# Comparative genomics of actinomycetes with a focus on natural product biosynthetic genes

**DOI:** 10.1186/1471-2164-14-611

**Published:** 2013-09-11

**Authors:** James R Doroghazi, William W Metcalf

**Affiliations:** 1 Institute for Genomic Biology, University of Illinois at Urbana-Champaign, Champaign, IL 61801, USA; 2 Department of Microbiology, University of Illinois at Urbana-Champaign, Champaign, IL 61801, USA

**Keywords:** Actinomycetes, Natural products, Genomics, Secondary metabolism

## Abstract

**Background:**

Actinomycetes are a diverse group of medically, industrially and ecologically important bacteria, studied as much for the diseases they cause as for the cures they hold. The genomes of actinomycetes revealed that these bacteria have a large number of natural product gene clusters, although many of these are difficult to tie to products in the laboratory. Large scale comparisons of these clusters are difficult to perform due to the presence of highly similar repeated domains in the most common biosynthetic machinery: polyketide synthases (PKSs) and nonribosomal peptide synthetases (NRPSs).

**Results:**

We have used comparative genomics to provide an overview of the genomic features of a set of 102 closed genomes from this important group of bacteria with a focus on natural product biosynthetic genes. We have focused on well-represented genera and determine the occurrence of gene cluster families therein. Conservation of natural product gene clusters within *Mycobacterium*, *Streptomyces* and *Frankia* suggest crucial roles for natural products in the biology of each genus. The abundance of natural product classes is also found to vary greatly between genera, revealing underlying patterns that are not yet understood.

**Conclusions:**

A large-scale analysis of natural product gene clusters presents a useful foundation for hypothesis formulation that is currently underutilized in the field. Such studies will be increasingly necessary to study the diversity and ecology of natural products as the number of genome sequences available continues to grow.

## Background

The class *Actinobacteria* is the largest within the phylum *Actinobacteria* and contains many bacteria relevant to human health and industry (see [1] for review). These bacteria are Gram-positive with genomic GC content generally over 55%. Some of them, such as the *Streptomyces*, were originally mistaken for fungi, as evidenced by the name of the group (*myces* is derived from the Greek word for fungus) and were once considered relatives of fungi based on morphology and life cycle. The existence of a life cycle involving multiple, distinct stages and morphologies has also made some actinomycetes, such as “*Streptomyces coelicolor*” A3(2), important model systems for studying differentiation and the signaling pathways involved therein.

The class *Actinobacteria*, or the actinomycetes, contains both the most deadly bacterial pathogen and the organisms that are the most important for antibiotic production. *Mycobacterium tuberculosis* is the second leading cause of death worldwide due to an infectious agent (after HIV/AIDS [2]), while the genus *Streptomyces* is the source of over half of the bioactive metabolites from bacteria [3]. The genus *Corynebacterium* contains deadly pathogens but also includes non-pathogens that are the leading producers of L-amino acids, which represent some of the most important microbial products in terms of both volume and value [4]. Numerous other pathogens and pharmaceutical producers, as well as ecologically and industrially important taxa are also found among this important microbial group.

Actinomycetes have historically been a leading source for natural product discovery [5]. These compounds, also called secondary metabolites, have a wide range of industrial uses, including as antineoplastic, antifungal, antimicrobial, herbicidal and plant growth promoting agents. They are also important components of iron-acquisition systems and signaling molecules important for development. Production of secondary metabolites may also be important adaptations to environments such as soil, and may aid competition for resources such as plant matter. Whatever their use, the genes that are responsible for production of individual secondary metabolites are almost always located together in the genome and are referred to as biosynthetic gene clusters. The co-location and horizontal transfer of these gene clusters is fascinating in and of itself, but is also a trait that aids in discovery, characterization and comparison of the genes responsible for secondary metabolite biosynthesis (see [6,7] for an overview and discussion of evolutionary implications).

Many researchers have voiced optimism that genome mining for novel secondary metabolites will result in a renaissance of discovery and fill the innovation gap that has left the pipelines at low levels [8-10]. The main reason for this is that *Streptomyces* and related genera, the traditional focus of discovery, rarely express their full inventory of chemical weapons when cultivated in the lab. For example, “*Streptomyces coelicolor*” A3(2) was a genetic workhorse for some 40 years before having its genome sequenced and was known to make only four secondary metabolites. The genome sequence revealed an additional 18 biosynthetic gene clusters [11]. Biosynthetic gene clusters which are present but not known to produce any secondary metabolites are referred to as cryptic clusters. There have been no systematic studies to date, however, on whether a cryptic biosynthetic gene cluster in one species is also likely to be cryptic in a second species, and therefore the fraction of undiscovered secondary metabolites based solely on genetic capacity may tend to overestimate the number of pathways that are cryptic. With this in mind, being able to classify and compare biosynthetic gene clusters, and thus systematically catalog the extent of natural product diversity, is an important first step towards a full exploitation of secondary metabolites in bacteria. This is, however, a difficult bioinformatics task for the two most common classes of natural products, type I polyketide synthases (PKS), and nonribosomal peptide synthetases (NPRS), due to the multiple similar domains present in both (see [12] for a review).

Currently, there are six actinomycete genera with sufficient numbers of completed genomes to allow an in-depth analysis of secondary metabolic diversity. We compared the genomes within these six, *Mycobacterium*, *Corynebacterium*, *Rhodococcus*, *Arthrobacter*, *Frankia*, and *Streptomyces*, in detail to determine the extent to which natural product gene clusters are conserved within each genus. We also present a broad, genome-scale comparison of complete genomes across the class *Actinobacteria*.

## Methods

All genomes were downloaded from NCBI on September 21, 2011. An attempt was made to include all species for which publicly available closed genomes were available within the order *Actinomycetales* as shown within NCBI taxonomy browser, although this taxonomic group has been re-ordered recently to compose the class *Actinobacteria*[1]. Plasmids were omitted from the analysis to prevent skewing long term evolutionary trends. Predicted proteins were used as annotated, and an all-v-all BLAST comparison was performed using BLAST v2.2.26+ [13].

### Phylogeny and whole genome comparisons

OrthoMCL version 2.0 with default settings was used for further analysis of BLAST results [14]. OrthoMCL similarity groups with “*S. coelicolor*” A3(2) genes annotated as ribosomal proteins were used for phylogenetic analysis. Only ribosomal protein genes in similarity groups containing a single gene from each species were used for this analysis. The complete list of genes used is: L1, L2, L3, L4, L5, L6, L7/L12, L9, L10, L11, L13, L14, L15, L16, L17, L18, L19, L20, L21, L22, L23, L24, L25p, L27, L29, L35, S1, S3, S5, S6, S7, S8, S9, S10, S11, S12, S13, S15, S17, S19, S20. The amino acid sequences of these genes were aligned with Clustal W 1.83 [15] and concatenated for phylogenetic analysis. The concatenated gene tree was made using FastTree 2.1.5 run with the Gamma20 model [16]. A NeighborNet network was created using the same data in the program SplitsTree 4.11.3 [17].

Groups of similar genes as output by OrthoMCL were parsed with custom Perl scripts to calculate pairwise genome similarity. Similarity was calculated as S_ij_/G_i_, where S_ij_ is the number of similar genes between genomes i and j, and G_i_ is the total number of genes in genome i. When multiple genes from the organisms being compared appeared in one similarity group, the count for number of similar genes was determined by whichever genome has fewer copies. Dividing by the total number of genes in only one genome means that there are two similarity measures presented for each pairwise comparison.

### Biosynthetic gene cluster discovery and comparison

Signature enzymes for major classes of secondary metabolites were found using profile Hidden Markov Models (pHMMs) and the program HMMER [18]. The pHMMs used are a mixture of those reported by Medema *et al*. [19] with the same cut-offs mentioned therein for PKS I, PKS II, PKS III, NRPS, indolocarbazoles, aerobactin-like siderophores, butyrolactones, aminoglycosides, and β-lactams, including screening for fatty acid synthases that are hit by the PKS models. New pHMMs were made for discovery of terpene synthases based on the sequences published in [20], lanthipeptides based on the required cyclase domain, see [21] for review, and thiazole-oxazole modified microcins, or TOMMs based on the YcaO domain [22]. The new pHMMs and alignments are presented in a stand-alone website (see Additional file 1). Phosphonates were found using a BLAST search and screening for sequences containing the EDK-X(5)-NS motif present in all verified PepM sequences (see [23] for review). Gene clusters were defined by extending six genes to either side of a significant pHMM hit (past the specified cut-off), joining additional hits within that window into the same cluster, and re-initiating the six gene count after encountering additional hits. The six gene extension was a practical choice; when we defined gene clusters with longer extensions the comparisons included more noise (divergent genomic neighborhoods not related to biosynthetic genes), and fewer genes in each cluster resulted in too little data for comparisons. This choice was made with future automation in mind. Similar gene clusters were found using an array of tools including phylogenetic comparisons and Mauve [24] alignments after concatenation of all gene clusters in each strain into one sequence. A website showing all gene clusters are included as Additional file 1. Gene cluster diagrams also include domain annotations, but these are not manually curated and some domains are incorrectly split in half. Gene annotation and domain names are available on mouseover.

## Results and discussion

**Figure 1:**
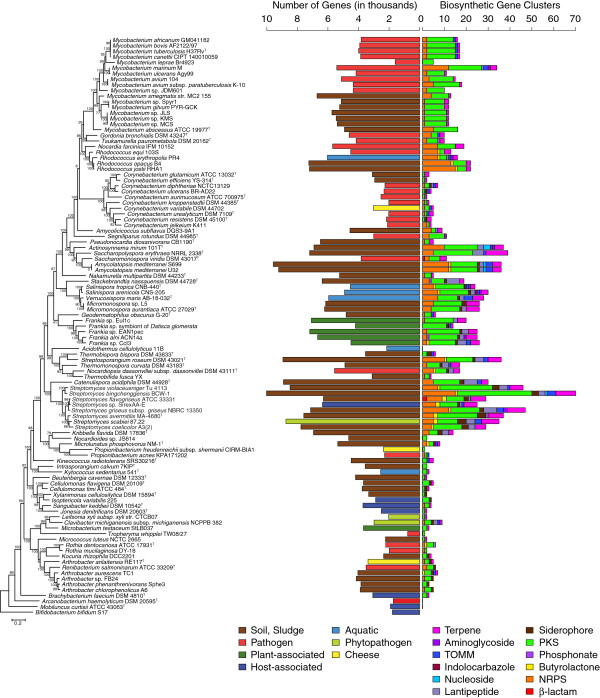
**Genome size, isolation source and number of secondary metabolite gene clusters.** The phylogenetic tree shown is calculated on concatenated ribosomal proteins and rooted with *Bifidobacterium bifidum* as an outgroup. The two bar plots are presented with species in the same order as the phylogenetic tree, representing genome size in thousands of genes on the left, colored by habitat, and number of secondary metabolite gene clusters on the right. Any combinations of cluster types found together count independently, e.g. an NRPS/PKS hybrid would be counted once as an NRPS and once as a PKS. The colors corresponding to habitat type and secondary metabolite class are shown in the key below the bar plots.

102 closed actinomycete genomes were grouped into seven broad categories according to isolation source, smear-ripened cheese being the most narrowly defined (Figure 1). The two most common isolation sources for actinomycetes are animal hosts and soil, although recently marine actinomycetes have garnered significant interest. Obligate pathogens, which by definition live in a well-defined and constant niche, tend to have undergone genome reduction, a trend not limited to actinomycetes [25]. Bacteria that dwell in soil, a very diverse and changing habitat, may benefit from a larger repertoire of genes that allows acclimation, response and adaptation to changing conditions and hence have much larger genomes.

**Figure 2:**
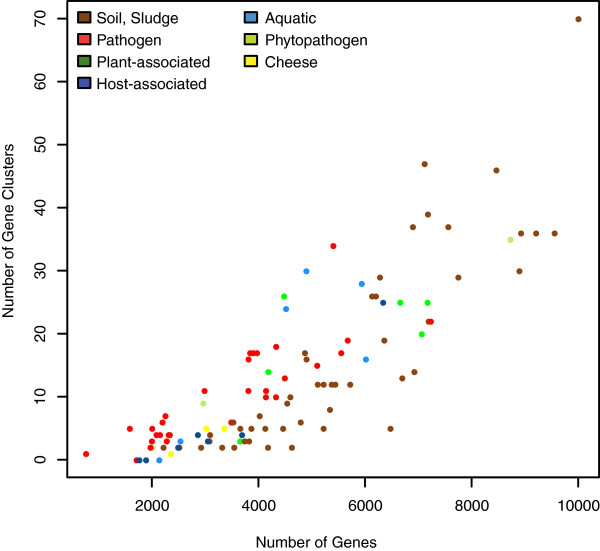
**Number of gene clusters per genome compared to genome size and habitat.** This compares the total number of gene clusters with the total number of genes. Each point is colored according to Figure 1. Note that the number of gene clusters for soil-isolated strains with genomes between 4–6000 genes declines before the number of secondary metabolite gene clusters from pathogens.

To provide context for the gene cluster comparisons, we constructed a phylogenetic tree using concatenated amino acid sequences from 41 ribosomal proteins shared by all strains (Figure 1). This is tree in good agreement with the phylogeny published by Gao and Gupta using 35 conserved genes from 98 actinobacterial genomes [26], although there are a couple of notable differences. In our tree *Nakamurella multipartita* DSM 44233^T^ is found outside of the *Pseudonocardiales*, where it was within *Pseudonocardiales* based on their tree. *Geodermatophilus obscurus* G-20^T^ was found to branch with *Frankia*, whereas their analysis suggested that it lay outside of the *Frankiales*. We also show that the groups they refer to as *Micrococcales* I and II group together, from *Leifsonia xyli* to *Arthrobacter chlorophenolicus* on our tree. Because it has already been shown that there can be extensive horizontal gene transfer within the actinomycetes [27,28], and that genome-based trees can differ from 16S and concatenated gene trees [29], we tested for recombination in the data set using the PHI test implemented in SplitsTree (p=1.0). A NeighborNet analysis was also not largely reticulate (Additional file 2), as one would expect for a data set impacted by homologous recombination. The secondary metabolite classes examined are also shown in Figure 1. While this is not an exhaustive list, it does cover all common secondary metabolites of actinomycetes. As might be expected, genome size and number of secondary metabolite biosynthetic gene clusters are positively correlated, as larger genomes can accommodate more gene clusters devoted to secondary metabolism (Figure 1 and Figure 2). This has also been noted in genomes of anaerobic microbes [30]. Interestingly, for genomes containing between 2000 and 6000 genes, pathogens tend to have a larger number of secondary metabolite biosynthetic gene clusters than free-living isolates from soil. This trend may not continue as more genomes from this order are sampled, however, as most of the pathogen genomes supporting this trend are from *Mycobacterium*. The same may be true with other patterns relating to isolation source.

**Figure 3:**
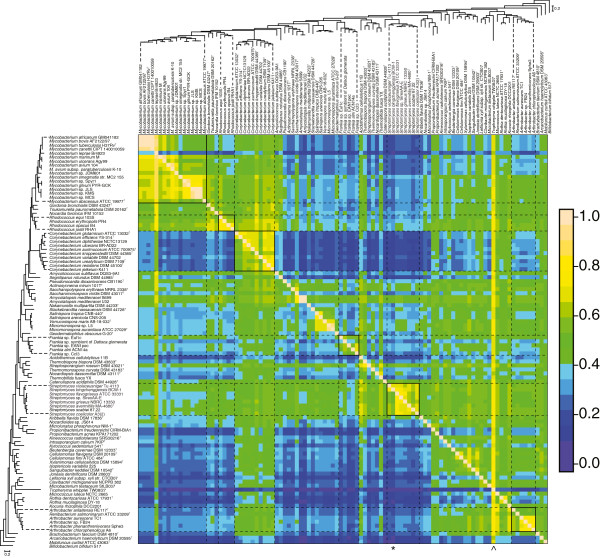
**Whole genome similarity.** The order for this comparison is the same as the phylogenetic tree in Figure 1, which has been shrunk and placed upon both axes for orientation. The heatmap legend is shown on the right. All comparisons between a genome and itself occur on a line stretching from the top left to the bottom right corners. The number of similar genes between two genomes is the numerator for each comparison and the genome represented by each column is used as the denominator for each comparison. The column divided by the size of *T. whipplei*, the smallest genome, is marked with ^ and the largest genome, *S. bingchenggensis*, is indicated with *.

To examine the overall similarity of the genomes between these organisms, we performed an all-vs-all BLAST search and grouped the results into sets of homologs using OrthoMCL. Two comparisons are shown in Figure 3. Both axes are ordered in the same way, based on the ribosomal protein tree. Each pairwise comparison is a tally of the homologs shared by two genomes. If multiple homologs were listed for each organism (e.g. *T. whipplei* has two copies of a gene and *S. bingchinggensis* has four) then the smaller number was counted for that single comparison. The total number of homologs for each pair of organisms was then divided by the total number of genes. This was done such that every vertical column is divided by the corresponding strain on the top, horizontal tree. For example, *Tropheryma whipplei* has only 783 protein coding genes due to reductive evolution as an intracellular pathogen. Therefore, *T. whipplei* shares nearly all its gene set with other strains (vertical column); while containing only a fraction of the genes present in other strains (horizontal row). In contrast, *S. bingchenggensis* has the largest number of protein-coding genes (10,022), so the many smaller genomes contain only a small fraction of the genes held by *S. bingchenggensis*, and this is reflected by a dark-colored vertical column.

Overall genome similarity clearly reflects the organismal phylogeny when distinguishing genera and large branches within a genus; however, the taxonomic level of genus is not uniformly applied. For example, *Salinispora, Verrucosispora*, and *Micromonospora* strains clearly show genomic similarities on the same degree as the other genera analyzed here and, thus, could be considered a single genus. The oldest of these genera, and therefore the one with precedence in naming, is *Micromonospora*[31]. *Verrucosispora* was described as a novel genus on the basis of a lack of arabinose in whole cell sugars, the presence of 10-methyl C_17:0_ fatty acids, and a 16S rRNA gene sequence not previously found in the family *Micromonosporaceae*[32]. The genus *Salinispora* was differentiated from other genera based largely on 16S rRNA gene diversity, a unique combination of fatty acid type and major menaquinones, and the requirement of sea water for growth [33]. It also appears that the genus *Arthrobacter*, which has long been divided into two groups, should be represented by two genera and *Renibacterium* should also remain separate. The case for *Arthrobacter* groups remaining in the same genus, however, was systematically considered and the two groups were determined to be members of the same genus with two “nuclei” [34]. A broader utilization of genomic data by the taxonomic community would assist in the creation of universal criteria for both species and genera definitions [35,36]. The genomes generated for research on natural products are very useful for improving actinobacterial systematics. Because taxonomy impacts both research focus and the interpretation of results, scientists with an interest in natural products should in turn not ignore the impact their data can have on taxonomy.

The whole genome comparisons also show a noticeable, but somewhat uneven, difference between rapid and slow-growing mycobacteria. It appears that the rate of genomic change leading to the branch containing *Mycobacterium leprae* and the *M. tuberculosis* strains has affected genomic content more than the change from rapid-growing nonpathogens to the slow-growing pathogens *Mycobacterium* sp. JDM601, *Mycobacterium avium* subsp. *paratuberculosis* K-10, *Mycobacterium avium* 104, *Mycobacterium ulcerans* Agy99 and *Mycobacterium marinum* M. In other words, the switch to pathogenicity itself did not require rapid genomic change because such rapid change is isolated to the *M. leprae* and *M. tuberculosis* branch of the tree. Unlike with *Mycobacterium* strains, the *Corynebacterium* isolates do not show such a large change between pathogens and nonpathogens. This is also reflected by what is known about the evolution of pathogenicity in *Corynebacterium*, as many pathogenicity factors appear to be acquired through recent horizontal gene transfer [37].

### Gene cluster diversity

**Figure 4:**
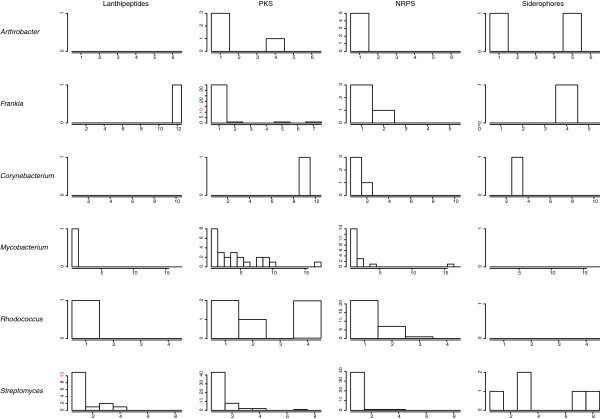
**Gene cluster conservation by class and genus.** Histograms showing the conservation of lanthipeptide, PKS, NRPS and NRPS-independent siderophores are shown for each genus. For example, in *Arthrobacter* there are three PKS gene clusters that are unique unto themselves and one type of PKS gene cluster that shows up four times. To emphasize the abundance of some classes in certain genera, the number 10 is highlighted in red on the y-axis when present.

Given the diversity of lifestyles and habitats of actinomycetes it should be expected that discrete genera use secondary metabolites differently. For many of the genera examined, the most conserved secondary metabolite clusters are siderophores, whether they are NRPS products or NRPS-independent. 41 out of 102 genomes contain at least one gene cluster for NRPS-independent siderophore biosynthesis (aerobactin-like), but 31/34 in the *Corynebacterium*, *Mycobacterium*, *Nocardia* group do not have this class of siderophores. The *Corynebacterium, Mycobacterium*, *Nocardia* group (from *Mycobacterium africanum* to *Segniliparus rotundus* DSM 44985 in Figure 1), all contain the gene cluster for mycolic acid, with the exception of *Corynebacterium kroppenstedtii* (see Additional file 1, Conserved Clusters)*.* In general, the genera with more pathogenic members, *Corynebacterium* and *Mycobacterium*, have higher proportions of conserved secondary metabolite gene clusters than the essentially saprophytic genera *Streptomyces* and *Rhodococcus* (Figure 4). This may be due to the increased homogeneity of environments inhabited by pathogens compared to free-living bacteria. This pattern based on host-association is broken with the *Frankia*, however, as *Frankia* species have almost no overlap in their secondary metabolic capabilities. All gene cluster families (GCFs) are shown in Additional file 3, and a stand-alone website is provided in Additional file 1 that contains all gene clusters found in the complete set of genomes. All conserved clusters mentioned are also present on the website provided under the “Conserved Clusters” link.

**Figure 5:**
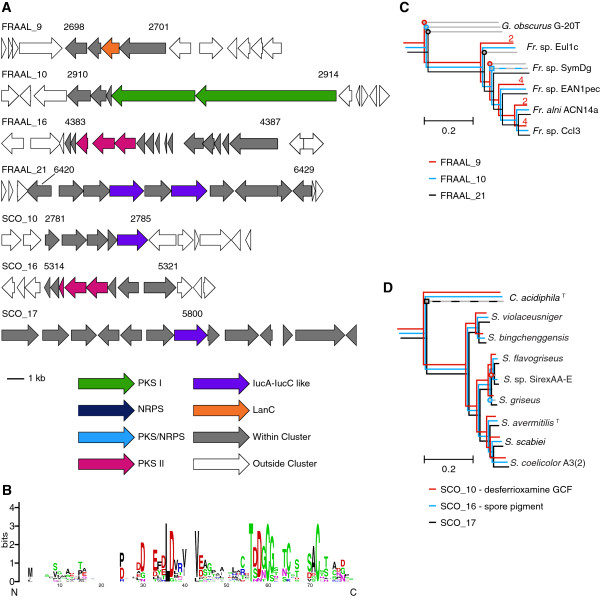
**Conserved gene clusters in *Streptomyces *and *Frankia. ***Four biosynthetic gene clusters were found to be well conserved in *Frankia* and three in *Streptomyces*. **(A)** The conservation of gene content was used to find putative gene cluster boundaries. The first and last conserved gene numbers are shown, along with the cluster name from our analysis. The standard six gene window extending from a pHMM hit are shown, with the exception of FRAAL_16 (*Frankia alni* ACN14a cluster 16) where additional genes are shown downstream of the original cluster due to continuing gene cluster content similarity. SCO_17 (“*Streptomyces coelicolor*” A3(2) cluster 17) occurs in a large, well conserved region, so no cluster boundaries were determined. **(B)** shows the sequence logo of all putative lanthipeptide precursors in *Frankia* clusters similar to FRAAL_9. **(C)** and **(D)** The occurrence of the well conserved gene clusters in *Frankia* and *Streptomyces* are shown on subtrees of the ribosomal protein phylogeny from Figure 1, along with the closest outgroup taxa. FRAAL_16 was found in all strains shown in **(C)** and is therefore not shown. Open circles show the start of branches lacking the relevant gene cluster. Open squares show the start of branches containing clusters with the same basic machinery but large scale changes compared to the other genomes. These branches are also shown with dashed lines. The number of lanthipeptide clusters similar to FRAAL_9 present in each *Frankia* genome is shown in red above the terminal branch on the tree.

One use for GCFs is the potential for cluster boundary delineation. Over evolutionary time natural product gene clusters will change their location on genomes and phylogenetic trees through horizontal gene transfer and genome rearrangements [6,7]. This mobility changes the surrounding genes, and if the GCF is found in enough genomic backgrounds, then the genes surrounding the cluster will change. The drop in gene content similarity is used to determine gene cluster boundaries shown in Figure 5. Knowing the genes involved in biosynthesis is essential for synthetic biologists and geneticists attempting to refactor pathways or to attempt heterologous expression of natural products in a new host.

Another use for GCFs is in correlating with molecular families through MS analyses. The basis for this work is that similar gene clusters should produce similar natural products [38]. The gene cluster families presented here can be correlated with the presence of such similar products, or molecular families, to uncover novel associations and find new natural products that would otherwise remain hidden in the analysis of a single sample.

#### *Mycobacterium*

**Figure 6:**
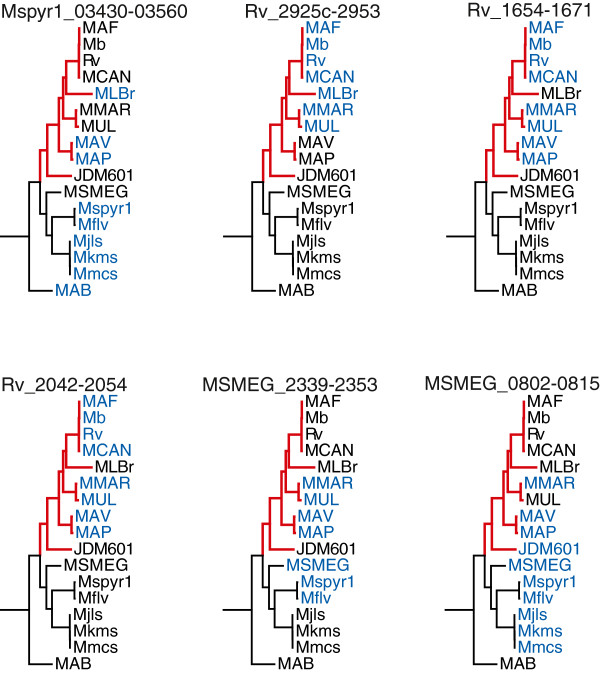
**Distribution of abundant gene clusters in *Mycobacterium. ***Six common secondary metabolite gene clusters with possibly informative phylogenetic distributions are shown from *Mycobacterium*. The tree is a subtree of that shown in Figure 1. Strains shown in blue contain the gene cluster indicated and strains shown in black do not. All strains above JDM601 are nonpathogenic or rarely opportunistic pathogens; branches leading to pathogenic strains are colored red. While none of these gene clusters have identical distribution patterns, the data suggest that some are important enough to be conserved for pathogenesis and others may be more useful in soil or aquatic habitats. Genome IDs used in this figure are: MAB, *Mycobacterium abscessus* ATCC 19977^T^; MSMEG, *Mycobacterium smegmatis* str. MC2 155; Mflv, *Mycobacterium gilvum* PYR-GCK; Mspyr1, *Mycobacterium* sp. Spyr1; Mjls, *Mycobacterium* sp. JLS; Mkms, *Mycobacterium* sp. KMS; Mmcs, *Mycobacterium* sp. MCS; JDM601, *Mycobacterium* sp. JDM601; MAP, *Mycobacterium avium* subsp. *Paratuberculosis* K-10; MAV, *Mycobacterium avium* 104; MUL, *Mycobacterium ulcerans* Agy99; MMAR, *Mycobacterium marinum* M; MLBr, *Mycobacterium leprae* Br4923; MCAN, *Mycobacterium canettii* CIPT 140010059; Rv, *Mycobacterium tuberculosis* H37Rv^T^; Mb, *Mycobacterium bovis* AF2122/97; and MAF, *Mycobacterium africanum* GM041182.

Within *Mycobacterium*, many of the PKS gene clusters are well conserved in large phylogenetic groups, Figure 6, which are largely accounted for by differences in the complicated cell wall of the mycobacteria. For example, the gene cluster for the production of mycolic acid is shared by all strains, whereas the genes for production of phthiocerol are only present in slow-growing, pathogenic strains. In contrast, the NRPS clusters, with one exception, are either unique or shared with only a single close relative. The single exception is the gene cluster for mycobactin synthesis, a characterized siderophore, which is found in all strains except *M. leprae*. Two scotochromogenic strains, *Mycobacterium gilvum* and *Mycobacterium* sp. Spyr1 (which is proposed as synonymous with *M. gilvum*[39]) share a lycopene cyclase not found in the other strains that is possibly the source of their coloration (Mflv_0944-0956, Mspyr1_50120-50240).

*Mycobacterium marinum* is a very unique genome with regards to natural products compared to other *Mycobacterium* genomes. It has seven NRPS clusters, two PKS clusters, and three hybrid PKS-NRPS clusters not found in other mycobacterial genomes completed to date. This is especially surprising given the very close relationship between *M. marinum* and *M. ulcerans*, which have an average nucleotide identity of >98% [40]. Stinear *et al.* has shown that these clusters are not found on a single genomic island, and some of them may represent recent duplication events followed by divergence [41]. The evolution of natural product gene clusters in this group has already been mapped out in detail, including a new genome sequence for *M. liflandii* not included in the present study [42].

#### *Corynebacterium*

*Corynebacterium* is not known for its ability to produce natural products of the kind investigated here, and their genomes have not held many surprises in these regards. The most conserved cluster is that for mycolic acid as discussed above. Unlike most bacteria in the *Corynebacterium*-*Nocardia-Mycobacterium* group examined here, three pathogenic strains, *Corynebacterium resistens* DSM 45100^T^, *Corynebacterium ulcerans* BR-AD22 and *Corynebacterium diphtheriae* NCTC13129, share an aerobactin-like non-NRPS siderophore gene cluster. The ratio of isoprenoid and terpenoid biosynthesis gene clusters to PKS and NRPS clusters is high in corynebacteria compared to other genera, but this may be due simply to low overall numbers. The importance of these compounds at least to some of these strains is highlighted by the presence of the discrete mevalonate and non-mevalonate pathways for isoprene biosynthesis in *Corynebacterium kroppenstedtii* DSM 44385^T^ and *Corynebacterium variabile* DSM 44702 [43]. Interestingly, the two mevalonate pathways seem to have reached *Corynebacterium* via different horizontal gene transfer routes, as they are only 54% similar to each other and more closely related to genes outside of the genus. The presence of two mevalonate pathways of different origins in *Actinobacteria* has been reported before, and these pathways are not unique to *Corynebacterium* among *Actinobacteria*[44].

#### *Arthrobacter*

The secondary metabolites in the *Arthrobacter* genomes examined here reveal little more than the divergence of *Renibacterium salmoninarum* ATCC 33209^T^ from both Group I and II arthrobacteria. Overall, these strains have very few secondary metabolite gene clusters. One NRPS independent, aerobactin-like siderophore cluster is shared among all strains except *Renibacterium*, and a type III PKS is shared by all Group I strains. *Arthrobacter arilaitensis* RE117^T^ and *Arthrobacter aurescens* TC1 also share a phytoene synthase gene cluster. The rest of the biosynthetic gene clusters present in this genus are unique to one strain.

#### *Rhodococcus*

The extent of secondary metabolite gene clusters revealed by *Rhodococcus* genome sequences was initially a surprise because no rhodococcal secondary metabolites were previously known [45]. In comparison with other actinomycete genomes, the *Rhodococcus* strains examined here have a skewed ratio of NRPS to PKS gene clusters. The average ratio of NRPS to PKS gene clusters for the entire data set is 0.45, but among rhodococcal genomes this ratio jumps to 2.8. In these four genomes there are only two PKS clusters that are found in only one strain, but each genome has at least four NRPS clusters that are not shared with any of the others. Despite the abundance of NRPS clusters, there are no conserved NRPS gene clusters; however, there are two conserved PKS clusters, one conserved phytoene synthase, which condenses two geranylgeranyl pyrophosphates to phytoene, one conserved lycopene cyclase, which cyclizes the ends of lycopene to the rings found in β-carotene, and a conserved butyrolactone biosynthetic gene cluster. The presence of a conserved butyrolactone biosynthetic gene cluster may indicate that a conserved cell-cell signaling pathway is important for the rhodococcal life cycle [46]. *Rhodococcus* strains are capable of differentiation and growth as either rods, cocci or hyphal filaments [47], but development has not been as well studied in this genus as in *Streptomyces*. The two strains from soil have larger genomes and more secondary metabolite biosynthetic gene clusters than *Rhodococcus erythropolis* PR4, a species isolated from a depth of 1,000 m in the Pacific Ocean south of Okinawa island, Japan, and *Rhodococcus equi* 103S, an equine pathogen.

#### *Streptomyces*

Based on solely genomic data, *Streptomyces* are the logical choice to mine for secondary metabolites. They have consistently high numbers of secondary metabolite biosynthetic gene clusters and a large variety of classes. Of course, streptomycetes have been the most heavily sampled historically, making rediscovery more likely when sampling from this genus. The eight genomes examined in this data set show a large diversity of gene clusters for secondary metabolism with little overlap between strains. The most common classes are PKS and NRPS, followed by terpenoids, aerobactin-like non-NRPS siderophores and lanthipeptides. All genomes contain the genes for butyrolactone biosynthesis, and in all but *Streptomyces griseus* at least one *afsA*, the central butyrolactone biosynthetic gene, homolog per genome is accompanied by a *tetR* family regulator immediately 5’ to *afsA* and in the opposite orientation (see Additional file 1, under Conserved Clusters). All eight genomes contain a non-NRPS aerobactin-like siderophore gene cluster similar to rhizobactin that is not currently tied to a product (SCO_17 in Figure 5). This gene cluster appears to be present in *Catenulispora acidiphila* as well, but significant changes to the gene cluster occurred between *C. acidiphila* and the most recent common ancestor of *Streptomyces*. All but *Streptomyces* sp. SirexAA-E contain the genes for the biosynthesis of the aerobactin-like siderophore desferrioxamine (nocardamine, SCO_10 in Figure 5). All streptomycetes, with the exception of *S. griseus,* contain the spore pigment type II PKS gene cluster. *S. griseus* contains a different spore pigment, produced instead by a type III PKS [48]. Interestingly, the lanthipeptide SapB, which was found to be required for aerial mycelia formation on rich media in “*S. coelicolor*” A3(2) and *S. griseus*[49], is only present in half of the strains.

Given the number of NRPS and PKS gene clusters in this genus, the amount of overlap with these clusters between genomes is very low. Unlike the abundance of NRPS clusters in *Rhodococcus* or PKS clusters in *Frankia* (discussed below), the ratio of NRPS to PKS clusters is also not heavily skewed in either direction and varies throughout the genus. While there has already been a significant amount of discovery of nonribosomal peptides and polyketides from *Streptomyces*, only a handful of terpenoids have been discovered from streptomycetes (see [20] for a review). Nevertheless, the number of terpene synthases present in these eight genomes comes close to those for PKS and NRPS biosynthesis, suggesting that a large diversity of terpenoids remain to be discovered in members of this genus.

#### *Frankia*

*Frankia* strains have a large number of secondary metabolite biosynthetic gene clusters, the vast majority of which are PKS clusters not shared with other strains. There are only four unique NRPS clusters within the genus, three of which occur only once and one that is shared by two strains. There are also two hybrid NRPS/PKS clusters, both unique. Out of the PKS clusters all but three sets of clusters are unique to one strain. Of the shared PKS clusters, one is a type II PKS shared by *Frankia* sp. CcI3 and *Frankia* sp. EuI1c, and one is a type II PKS conserved by all strains. The other cluster is a type I PKS that is conserved in all strains and duplicated in *Frankia* sp. EuI1c and *Frankia alni* ACN14a. There is only one type of lanthipeptide cluster found within the genus, but it is found either twice or four times in all genomes except FsymDg (*Frankia* symbiont of *Datisca glomerata,* Figure 5B). The sequence logo for the putative precursor peptides from these twelve lanthipeptide gene clusters show two conserved cysteine residues and a conserved threonine, along with a conserved LD motif that may be related to cleavage of the leader peptide. The conservation of cysteines, threonines and serines is biologically significant in lanthipeptides, as these residues are involved in lanthionine formation and cyclization that is central to lanthipeptide function (see [21] for review).

### Other genera

The marine actinomycetes in the genus *Salinispora* have been a recent focus of natural products research because they have been historically understudied and because they possess large numbers of secondary metabolite gene clusters [50]. Moreover, they have the genetic capacity to produce a diverse array of natural product classes, Figure 1. Of the twelve classes examined in this study, *Salinispora tropica* and *Salinispora arenicola* have gene clusters that involve seven and nine classes, respectively. Thus, of the complete genomes examined here, *S. arenicola* has the highest diversity of secondary metabolite classes.

The genomes of *Amycolatopsis mediterranei* U32 and S699 (AMED and RAM, respectively), *Actinosynnema mirum* 101T^T^ (Amir), *Pseudonocardia dioxanivorans* CB1190^T^ (Psed) and *Saccharopolyspora erythraea* NRRL 2338^T^ (SACE) also show a large number and diversity of secondary metabolite biosynthetic gene clusters. These strains were already known to produce rifamycin (AMED and RAM), nocardicin (Amir), and erythromycin (SACE). *Amycolatopsis* and *Saccharopolyspora* in particular are heavily researched, industrially important strains. *Saccharomonospora viridis* DSM 43017^T^, a pathogen that falls within the order *Pseudonocardiales*, has a smaller genome compared to its closest relatives in this analysis, a common theme among pathogens, and a corresponding large decrease in secondary metabolite biosynthetic gene clusters. The order *Streptosporangiales* also has significant potential for secondary metabolite production based on genome mining, although this is highly variable dependent on the genus examined.

## Conclusions

We have concerned ourselves here with the study of natural product genetic diversity throughout the actinomycetes because the resultant patterns and observations add depth and breadth to our understanding of their molecular biology and ecology. The work presented in this manuscript is our first step towards a systematic framework for studying natural products, a difficult bioinformatic task especially for PKS and NRPS systems. We have found patterns showing that some genera have higher prevalence of NRPS or PKS natural products compared to other genera. We have used multiple types of comparisons to group every gene cluster in each genus well-represented by complete genomes. Such gene cluster families are essential for determining cluster boundaries and as part of integrated data sets for novel natural product discovery. These groupings found conservation of the spore pigment and desferrioxamine class of siderophores in *Streptomyces*, along with mycolic acid, mycobactin and phthiocerol in *Mycobacterium*. When applied to less well-studied genera, analysis of conservation within phylogenetic groups is a first-step tool to form hypotheses about pathways that may be of similar importance. Our focus on the genomes available from *Frankia* has allowed us to generate hypotheses about the importance of several natural product gene cluster families that may relate to core aspects of the evolution and biology of *Frankia*. We also show that some mycobacterial natural product gene clusters with uncharacterized products are preferentially conserved on one of the other side of the fast or slow growing split that divides the genus. All conserved clusters are shown together on a stand-alone website, as well as the complete collection of all gene clusters found in these genomes. Our broad overview of actinomycete genomic diversity also reinforces the view that several genera within the *Actinobacteria* may be in need of new descriptions that take genomic diversity into account. It is our hope that this work will provide valuable leads in the field about yet unforeseen aspects of actinomycete biology and ecology.

## Competing interests

The authors declare that they have no competing interests.

## Authors' contributions

JRD designed and performed the research and wrote the manuscript. WWM guided the research and edited the manuscript. Both authors read and approved the final manuscript.

## Supplementary Material

Additional file 1 **A stand-alone website showing all natural product gene clusters analyzed in this study, along with separate files for conserved clusters mentioned in the text and pHMM files.** Use of the HTML files requires Javascript. Homologous genes are shown in the same color. All homologous genes on a page are highlighted upon mouseover of any of them. Mouseover also produces a description containing the locus tag and annotation for each gene. Mouseover for a domain box above the gene arrows shows the domain name. Clicking on a gene arrow produces a page with the amino acid sequence and a link to BLAST the nr protein database. Click here for file

Additional file 2 A NeighborNet analysis on concatenated ribosomal proteins. Click here for file

Additional file 3 List of genes grouped together within the genera of interest, gene range is separated by commas and gene groups are separated by semicolons. Click here for file
