# Supplementary material for: Comparative genomics of actinomycetes with a focus on natural product biosynthetic genes
Source: BMC Genomics. 2013 Sep 11;14:611. doi: 10.1186/1471-2164-14-611 (PMC3848822; doi:10.1186/1471-2164-14-611)
Supplement: Additional file 1 — A stand-alone website showing all natural product gene clusters analyzed in this study, along with separate files for conserved clusters mentioned in the text and pHMM files. Use of the HTML files requires Javascript. Homologous genes are shown in the same color. All homologous genes on a page are highlighted upon mouseover of any of them. Mouseover also produces a description containing the locus tag and annotation for each gene. Mouseover for a domain box above the gene arrows shows the domain name. Clicking on a gene arrow produces a page with the amino acid sequence and a link to BLAST the nr protein database. [file 1471-2164-14-611-S1.zip › website/allcomplete.html]

 

 

 
	 allcomplete 

 


 
 

 

 

 
 
   PFAAALMDRFGIRRVVVAALSAVAGGALLTTVMNASWQFTLFWGFLVGLGTGSLAMTFGATVTNRWFVQRRGLVTGMLSSGSVLGQLVFLPALSWTIDHY DWRPALITLALVALATAPLVRLTLRDHPADLGQRPYGSDAFVPKPPPVTGAARRTVTVLRDSARTRPFWLLAALFAICGASTNGIMWTHFAPAAHDHGMP VTTASTLLAVIGVFNVAGTIGSGWLTDRVDARRLLAVFFTLRGVLLVTLPLLMAATVNPPMMVFVVVFGLLDLATVPPVIALCRQFHGADSAIVFGWTNA AHQVGAALAALLGGVARDAFGSYDFVWVLLGAACAAAALLALVIQRPSAPRSTELRDTPVPSTADCATGR', 'http://blast.ncbi.nlm.nih.gov/Blast.cgi?PAGE=Proteins&PROGRAM=blastp&BLAST_PROGRAMS=blastp&QUERY=MPSPHAWPDCFTTCPVGQWYGRPASRRVTGMEDTTGHPRNAQGHRAWWVAAVAGLAIVVAGAFSTMPGILQGPLHREFHWSRGSIGLAASVNMVLYGVTA PFAAALMDRFGIRRVVVAALSAVAGGALLTTVMNASWQFTLFWGFLVGLGTGSLAMTFGATVTNRWFVQRRGLVTGMLSSGSVLGQLVFLPALSWTIDHY DWRPALITLALVALATAPLVRLTLRDHPADLGQRPYGSDAFVPKPPPVTGAARRTVTVLRDSARTRPFWLLAALFAICGASTNGIMWTHFAPAAHDHGMP VTTASTLLAVIGVFNVAGTIGSGWLTDRVDARRLLAVFFTLRGVLLVTLPLLMAATVNPPMMVFVVVFGLLDLATVPPVIALCRQFHGADSAIVFGWTNA AHQVGAALAALLGGVARDAFGSYDFVWVLLGAACAAAALLALVIQRPSAPRSTELRDTPVPSTADCATGR&LINK_LOC=protein&PAGE_TYPE=BlastSearch', 'BLAST this protein','SAV_1555')" />

 
 
   VRRVVPVFLGASSLSVVTELCQGLLPWIGRSCDSSDVQMNVLGAALGALAGAASVRAFRGGLRPLTSALRPTLLAFGSGILVCGVVGSCWITPVAVDPTS LRLAGGAEREAARQAVTAAFGDHERIVNVQVMPGWDGGADTLLIALETGSAELSGPDGEVFSADFSTPPEQPGPAGFPVAGAAGPRNADDALRTATSYAE ERFPWALSGTIATSTPVGARAESGWVVAWRGNTDGVVAPTRLDVRIDREGRVAALLARHVKDAPTTFPPRRVSEKRALAAAAAYYPGAVSGAVELLAVRR DGAWRAQWFVPVASAPVTAAGAPVTVDVVPVYVDAETGQVDDRPTPPHGLPTVLPPTAEESVETVELPDS', 'http://blast.ncbi.nlm.nih.gov/Blast.cgi?PAGE=Proteins&PROGRAM=blastp&BLAST_PROGRAMS=blastp&QUERY=MLEAVFKDQGAFVLVAAGAVSAAALLAYLGARKHTRRPWSYAGLAAALTAEVGVTLLLPSAGGESRRCVVVRDLGEPFATEQGLLNLAMFLVVGFLGVLA VRRVVPVFLGASSLSVVTELCQGLLPWIGRSCDSSDVQMNVLGAALGALAGAASVRAFRGGLRPLTSALRPTLLAFGSGILVCGVVGSCWITPVAVDPTS LRLAGGAEREAARQAVTAAFGDHERIVNVQVMPGWDGGADTLLIALETGSAELSGPDGEVFSADFSTPPEQPGPAGFPVAGAAGPRNADDALRTATSYAE ERFPWALSGTIATSTPVGARAESGWVVAWRGNTDGVVAPTRLDVRIDREGRVAALLARHVKDAPTTFPPRRVSEKRALAAAAAYYPGAVSGAVELLAVRR DGAWRAQWFVPVASAPVTAAGAPVTVDVVPVYVDAETGQVDDRPTPPHGLPTVLPPTAEESVETVELPDS&LINK_LOC=protein&PAGE_TYPE=BlastSearch', 'BLAST this protein','SACTE_5795')" />

 
 
   TAVTALIYPASLGQPAVEQLEAACAAGGTSFHGTGIEPGWAAEVLPLTMSTLFERIDSLLVQELLDYSSYDSVEMMFDIMGFGQAPDAPVPLSVPELAGS AFRAPLMLVADGLGAAVDDFVYDRQVAVAAEAFDIKVGRIEAGTVSAQRFSATAVIGGRPALTVEHITRVGSGQAPDWPTGRGWKVTVEGSPSMVLEAKI AVHGEDENDQGCLGTAMHAVHAIAPVCAAPAGIRTFLDLPLLNGRHVLGPGARPVIPAPADASS', 'http://blast.ncbi.nlm.nih.gov/Blast.cgi?PAGE=Proteins&PROGRAM=blastp&BLAST_PROGRAMS=blastp&QUERY=MLRVVVWATGTVGRHAVRAVAAHPDLELVGAFVYAEAKAGRDVGEIAGIGPVGVAATRDKDEVVALDADCVLYLAQGELDPAGALDDICRLLASGKNVIS TAVTALIYPASLGQPAVEQLEAACAAGGTSFHGTGIEPGWAAEVLPLTMSTLFERIDSLLVQELLDYSSYDSVEMMFDIMGFGQAPDAPVPLSVPELAGS AFRAPLMLVADGLGAAVDDFVYDRQVAVAAEAFDIKVGRIEAGTVSAQRFSATAVIGGRPALTVEHITRVGSGQAPDWPTGRGWKVTVEGSPSMVLEAKI AVHGEDENDQGCLGTAMHAVHAIAPVCAAPAGIRTFLDLPLLNGRHVLGPGARPVIPAPADASS&LINK_LOC=protein&PAGE_TYPE=BlastSearch', 'BLAST this protein','FraEuI1c_2679')" />

 
 
   DTAQLYPVSTLDRTEAKTAVATLSPTGWTPIGPALLKAADDLDGGTGSHRIVLITDGEDTCAPLDPCEVAREIAAKGVGLTIDTLGLVPNSKLSKQLSCI AEATGGTYTSVEHKEDLTDKVNQLVDRAADKVVTPVAVDGAADCAKAPTLKSGLFTDRAEFAQHRWYRVDVKPGQELRASVSLSADRQVNPDYGVLLRAV TVHNREIVRGEAAGTGRTDVVSTGLRYPKAESDDEDATAETVCLQVSHSFSPAAGVKTTPGLPLELTVDVVDGPDQAGDVASFGLGRGWWLLGALVLTGF LAGLLWGWLSRWRFAVWRTN', 'http://blast.ncbi.nlm.nih.gov/Blast.cgi?PAGE=Proteins&PROGRAM=blastp&BLAST_PROGRAMS=blastp&QUERY=MITRQRLAVGLCALLAALTAGFAFPAGAVADETTADAPKVDLVLDVSGSMRARDIDGGSRMAAAKQAFNEVLDATPEEVRLGIRTLGANYPGDDRKTGCK DTAQLYPVSTLDRTEAKTAVATLSPTGWTPIGPALLKAADDLDGGTGSHRIVLITDGEDTCAPLDPCEVAREIAAKGVGLTIDTLGLVPNSKLSKQLSCI AEATGGTYTSVEHKEDLTDKVNQLVDRAADKVVTPVAVDGAADCAKAPTLKSGLFTDRAEFAQHRWYRVDVKPGQELRASVSLSADRQVNPDYGVLLRAV TVHNREIVRGEAAGTGRTDVVSTGLRYPKAESDDEDATAETVCLQVSHSFSPAAGVKTTPGLPLELTVDVVDGPDQAGDVASFGLGRGWWLLGALVLTGF LAGLLWGWLSRWRFAVWRTN&LINK_LOC=protein&PAGE_TYPE=BlastSearch', 'BLAST this protein','SAV_2005')" />
   DTAQLYPVGPLDRTEAKTAVATLAPTGWTPIGPALLKAADDLEGGEGTKRIVLISDGEDTCAPLDPCEVAREIAAKGIGLTIDTLGLVPNAKLRVQLSCI AEATGGTYTSIEHRDELTDRVNQLVDRAADPVVVPKAVEGAEACAKAPTLTSGLYTDREEFARQRWYRVDVKPGQELRASVSVAADRDVNPDYGVLLRAV TAENREIVRGEAAGNGRTDVISTGLRYPKAEDEDDADEDAAETVCLQVTHSYAPASGVKTTPGLPLEITVDVVEAPDQAADVASFGLGRGWWLLGVLVLV GFLAGVLWGWLSRWRVAVWRTN', 'http://blast.ncbi.nlm.nih.gov/Blast.cgi?PAGE=Proteins&PROGRAM=blastp&BLAST_PROGRAMS=blastp&QUERY=MITRQRLATGAFVLLAVLTAGIAVPTTAVADEISATAPKVNLLLDVSGSMRAKDIDGQSRMSAAKQAFNEVLDATPKEVQLGIRTLGADYPGDDRKTGCK DTAQLYPVGPLDRTEAKTAVATLAPTGWTPIGPALLKAADDLEGGEGTKRIVLISDGEDTCAPLDPCEVAREIAAKGIGLTIDTLGLVPNAKLRVQLSCI AEATGGTYTSIEHRDELTDRVNQLVDRAADPVVVPKAVEGAEACAKAPTLTSGLYTDREEFARQRWYRVDVKPGQELRASVSVAADRDVNPDYGVLLRAV TAENREIVRGEAAGNGRTDVISTGLRYPKAEDEDDADEDAAETVCLQVTHSYAPASGVKTTPGLPLEITVDVVEAPDQAADVASFGLGRGWWLLGVLVLV GFLAGVLWGWLSRWRVAVWRTN&LINK_LOC=protein&PAGE_TYPE=BlastSearch', 'BLAST this protein','SCAB_18411')" />
   KDTAQLYPVGPLDRTEAKTAVATLSPTGWTPIGPALLKAADDLDGGDGSKRIVLISDGEDTCAPLDPCEVAREIAAKGIGLTIDTLGLVPNTKMRQQLSC IAEATGGTYTSVEHTDELTDKVNQLVDRAADPVVTPVATEGADACAKAPTLKSGLYTDREEFGQQRWYRVDVEPGQELRASVSVGADRAVNPSYGVLLRA VTVHGREIVRGEAAGNGRTDVLSTGLRYPKAKSDEDDAPAEAVCLQVTNSFSVASGVKTSPGLPLELTVDVVDGPGDSDDVAAFGLGRGWWLLGLLVLVG FVAGLLWGWLSRWRFAIWRTN', 'http://blast.ncbi.nlm.nih.gov/Blast.cgi?PAGE=Proteins&PROGRAM=blastp&BLAST_PROGRAMS=blastp&QUERY=MITRTRLAAGACALLAALAAGMAFPAGASAGEPTGEDAPKVDLVLDVSGSMRTRDIDGGTRMAAAKQAFNEVLDATPEEVRLGIRTLGADYPGDDRKTGC KDTAQLYPVGPLDRTEAKTAVATLSPTGWTPIGPALLKAADDLDGGDGSKRIVLISDGEDTCAPLDPCEVAREIAAKGIGLTIDTLGLVPNTKMRQQLSC IAEATGGTYTSVEHTDELTDKVNQLVDRAADPVVTPVATEGADACAKAPTLKSGLYTDREEFGQQRWYRVDVEPGQELRASVSVGADRAVNPSYGVLLRA VTVHGREIVRGEAAGNGRTDVLSTGLRYPKAKSDEDDAPAEAVCLQVTNSFSVASGVKTSPGLPLELTVDVVDGPGDSDDVAAFGLGRGWWLLGLLVLVG FVAGLLWGWLSRWRFAIWRTN&LINK_LOC=protein&PAGE_TYPE=BlastSearch', 'BLAST this protein','SCO_6225')" />

 
 
   GALLATTGLAGLLSLSGSSGARALAKDDRITVAVVTVSAIAVLALAARWSRRPGVRSVLLAVAAGTAFGVSSVFTKNVTVELEWGKPEFEGWASGLPSLA MIGLLAGAGVLLSQASYRGAGLAAPLATATVANPVVATAIGVTALGEHFRHGASGTLLALLAALVAGTGLVMLTVHGVETSGQPADGAPAPDPAGVKAAP RPGETDATDATGGPDGTDGASGTGGGHEGRGPDDRTGGPGAGGTGAGAARPRRLPRIPRPARGADDRLTPDRRSPGRRSRDQRRSTRGEAVLQDHR', 'http://blast.ncbi.nlm.nih.gov/Blast.cgi?PAGE=Proteins&PROGRAM=blastp&BLAST_PROGRAMS=blastp&QUERY=MTSLALSVLLALASAVCYAAGAILQEHVAATTPPRPYAALRRGRWWTAVVLNGTGAVLHVLALAYGPLSVVQPLGALTIVFALPMAAVFVRRPVGAAGWR GALLATTGLAGLLSLSGSSGARALAKDDRITVAVVTVSAIAVLALAARWSRRPGVRSVLLAVAAGTAFGVSSVFTKNVTVELEWGKPEFEGWASGLPSLA MIGLLAGAGVLLSQASYRGAGLAAPLATATVANPVVATAIGVTALGEHFRHGASGTLLALLAALVAGTGLVMLTVHGVETSGQPADGAPAPDPAGVKAAP RPGETDATDATGGPDGTDGASGTGGGHEGRGPDDRTGGPGAGGTGAGAARPRRLPRIPRPARGADDRLTPDRRSPGRRSRDQRRSTRGEAVLQDHR&LINK_LOC=protein&PAGE_TYPE=BlastSearch', 'BLAST this protein','SBI_02072')" />

 
 
   SGELRLEDIGDHATGGRWMIDNIAIRTKFYDDFFGDATTAGIRQVVILAAGLDTRAYRLPWPPGTVVYEIDQPAVIKFKTRALANLNAEPNAERHAVAVD LRNDWPTALKNAGFDPARPTAFSAEGLLSYLPPQGQDRLLDAITALSAPDSRLATQSPLVLDLAEEDEKKMRMKSAAEAWRERGFDLDLTELIYFDQRND VADYLAGSGWQVTTSTGKELFAAQGLPPFEDDHITRFADRRYISAVLK', 'http://blast.ncbi.nlm.nih.gov/Blast.cgi?PAGE=Proteins&PROGRAM=blastp&BLAST_PROGRAMS=blastp&QUERY=MARPMGKLPSNTRKCAQCAMAEALLEIAGQTINQKDLGRSGRMTRTDNDTWDLASSVGATATMIATARALASRAENPLINDPFAEPLVRAVGIDLFTRLA SGELRLEDIGDHATGGRWMIDNIAIRTKFYDDFFGDATTAGIRQVVILAAGLDTRAYRLPWPPGTVVYEIDQPAVIKFKTRALANLNAEPNAERHAVAVD LRNDWPTALKNAGFDPARPTAFSAEGLLSYLPPQGQDRLLDAITALSAPDSRLATQSPLVLDLAEEDEKKMRMKSAAEAWRERGFDLDLTELIYFDQRND VADYLAGSGWQVTTSTGKELFAAQGLPPFEDDHITRFADRRYISAVLK&LINK_LOC=protein&PAGE_TYPE=BlastSearch', 'BLAST this protein','MAF_34130')" />
   SGELRLEDIGDHATGGRWMIDNIAIRTKFYDDFFGDATTAGIRQVVILAAGLDTRAYRLPWPPGTVVYEIDQPAVIKFKTRALANLNAEPNAERHAVAVD LRNDWPTALKNAGFDPARPTAFSAEGLLSYLPPQGQDRLLDAITALSAPDSRLATQSPLVLDLAEEDEKKMRMKSAAEAWRERGFDLDLTELIYFDQRND VADYLAGSGWQVTTSTGKELFAAQGLPPFEDDHITRFADRRYISAVLK', 'http://blast.ncbi.nlm.nih.gov/Blast.cgi?PAGE=Proteins&PROGRAM=blastp&BLAST_PROGRAMS=blastp&QUERY=MARPMGKLPSNTRKCAQCAMAEALLEIAGQTINQKDLGRSGRMTRTDNDTWDLASSVGATATMIATARALASRAENPLINDPFAEPLVRAVGIDLFTRLA SGELRLEDIGDHATGGRWMIDNIAIRTKFYDDFFGDATTAGIRQVVILAAGLDTRAYRLPWPPGTVVYEIDQPAVIKFKTRALANLNAEPNAERHAVAVD LRNDWPTALKNAGFDPARPTAFSAEGLLSYLPPQGQDRLLDAITALSAPDSRLATQSPLVLDLAEEDEKKMRMKSAAEAWRERGFDLDLTELIYFDQRND VADYLAGSGWQVTTSTGKELFAAQGLPPFEDDHITRFADRRYISAVLK&LINK_LOC=protein&PAGE_TYPE=BlastSearch', 'BLAST this protein','Mb_3432')" />
   SGELRLEDIGDHATGGRWMIDNIAIRTKFYDDFFGDATTAGIRQVVILAAGLDTRAYRLPWPPGTVVYEIDQPAVIKFKTRALANLNAEPNAERHAVAVD LRNDWPTALKNAGFDPARPTAFSAEGLLSYLPPQGQDRLLDAITALSAPDSRLASQSPLVLDLAEEDEKKMRMKAAAEAWRERGFDLDLTELIYFDQRND VADYLAGSGWQVTTSTGKELFAAQGLPPFEDDHITRFADRRYISAVLK', 'http://blast.ncbi.nlm.nih.gov/Blast.cgi?PAGE=Proteins&PROGRAM=blastp&BLAST_PROGRAMS=blastp&QUERY=MARPMGNLPSNTRKCAQRAMAEALLEIAGQTINQKDLGRSGRMTRTDNDTWDLASSVGATATMIATARALASRAENPLINDPFAEPLVRAVGIDLFTRLA SGELRLEDIGDHATGGRWMIDNIAIRTKFYDDFFGDATTAGIRQVVILAAGLDTRAYRLPWPPGTVVYEIDQPAVIKFKTRALANLNAEPNAERHAVAVD LRNDWPTALKNAGFDPARPTAFSAEGLLSYLPPQGQDRLLDAITALSAPDSRLASQSPLVLDLAEEDEKKMRMKAAAEAWRERGFDLDLTELIYFDQRND VADYLAGSGWQVTTSTGKELFAAQGLPPFEDDHITRFADRRYISAVLK&LINK_LOC=protein&PAGE_TYPE=BlastSearch', 'BLAST this protein','MCAN_34231')" />
   SGELRLEDIGDHATGGRWMIDNIAIRTKFYDDFFGDATTAGIRQVVILAAGLDTRAYRLPWPPGTVVYEIDQPAVIKFKTRALANLNAEPNAERHAVAVD LRNDWPTALKNAGFDPARPTAFSAEGLLSYLPPQGQDRLLDAITALSAPDSRLATQSPLVLDLAEEDEKKMRMKSAAEAWRERGFDLDLTELIYFDQRND VADYLAGSGWQVTTSTGKELFAAQGLPPFADDHITRFADRRYISAVLK', 'http://blast.ncbi.nlm.nih.gov/Blast.cgi?PAGE=Proteins&PROGRAM=blastp&BLAST_PROGRAMS=blastp&QUERY=MARPMGKLPSNTRKCAQCAMAEALLEIAGQTINQKDLGRSGRMTRTDNDTWDLASSVGATATMIATARALASRAENPLINDPFAEPLVRAVGIDLFTRLA SGELRLEDIGDHATGGRWMIDNIAIRTKFYDDFFGDATTAGIRQVVILAAGLDTRAYRLPWPPGTVVYEIDQPAVIKFKTRALANLNAEPNAERHAVAVD LRNDWPTALKNAGFDPARPTAFSAEGLLSYLPPQGQDRLLDAITALSAPDSRLATQSPLVLDLAEEDEKKMRMKSAAEAWRERGFDLDLTELIYFDQRND VADYLAGSGWQVTTSTGKELFAAQGLPPFADDHITRFADRRYISAVLK&LINK_LOC=protein&PAGE_TYPE=BlastSearch', 'BLAST this protein','Rv_3399')" />

 
 
   TVLGCLLAAITYALGPLVATVFSNDALVDIVRWTSVVFVVNGAATQFRAELNRRLDFKMLALVDTVPVMVGLASAVGYVVFVNADYWALICQQLVTATSG LLLAVMTARWFPGLPNRNGSIRSLVSFGLGLFGTQGVAYFTRNVDNLSLGYVWGPSELGMYSRAYQLLMVPINQISAPLTRVAVPILTRVADQRERFDSF LRTGQLMGGVVLGVGYGILFGLADPIVNVVFGGAWDPMVPIFQALAVGGVFRALNQVTFWVFLAKGATGAQFRFYLVSQPLIVVSMLAGLPWGAVGVAVG HSVGYCMNWMISYWWCGRATGTSLGKLFVAGLKSIALFAAPVGLIGLAAVSVVTNPAVAIGIGLGLIGIWFGLLWLVSGFARETFSTGARAAQKVIAKKM PVN', 'http://blast.ncbi.nlm.nih.gov/Blast.cgi?PAGE=Proteins&PROGRAM=blastp&BLAST_PROGRAMS=blastp&QUERY=MTAGPKDAIDDHPGESSALASTAARGASYTIAGQLVRIVVMFAGTIVLARLLSPEDFGLVAIVASLVAFGELARDFGLSTAAAREKNLTKAQQSNLFWIN TVLGCLLAAITYALGPLVATVFSNDALVDIVRWTSVVFVVNGAATQFRAELNRRLDFKMLALVDTVPVMVGLASAVGYVVFVNADYWALICQQLVTATSG LLLAVMTARWFPGLPNRNGSIRSLVSFGLGLFGTQGVAYFTRNVDNLSLGYVWGPSELGMYSRAYQLLMVPINQISAPLTRVAVPILTRVADQRERFDSF LRTGQLMGGVVLGVGYGILFGLADPIVNVVFGGAWDPMVPIFQALAVGGVFRALNQVTFWVFLAKGATGAQFRFYLVSQPLIVVSMLAGLPWGAVGVAVG HSVGYCMNWMISYWWCGRATGTSLGKLFVAGLKSIALFAAPVGLIGLAAVSVVTNPAVAIGIGLGLIGIWFGLLWLVSGFARETFSTGARAAQKVIAKKM PVN&LINK_LOC=protein&PAGE_TYPE=BlastSearch', 'BLAST this protein','REQ_40610')" />

 
 
   SVLLLYAAYAWLNLYTTALIAAASVIGMLVGAEIPLLMELLQRIRRQAPGAAVADLFAADYVGALLGGLAFPFLLLPVFGQIRGSLLVGVLNAAAGLALV FVLFRDRLTRPARLLFTALTALVALVLGGSYVYADRFEATAQQALFAHPIVRHEQTRYQSIVLTESLSPFATPDVRLYLNGDLQFSSVDEYRYHEALVHP VLAGPRARVLVLGGGDGLALREILRYPDVAEVTLVELDPGMIHLARTEPRLLALNRHAFDDPRVRVINEDAFAWLRGQSGRFDAIIVDLPDPDQTSIAKL YTKEFYAMVAQVLAPGGTVAVQSGSPFFAPRSFWCIAATIRAGGLRVLPYHVDVPSFGDWGFVLATRDVEPVLRLDPPAPPRSIDAESLRAAAVFAPDRR DPGAPVSTLMHPVIVDLELREWR', 'http://blast.ncbi.nlm.nih.gov/Blast.cgi?PAGE=Proteins&PROGRAM=blastp&BLAST_PROGRAMS=blastp&QUERY=MRAGTRTRNPVAPSPVRARTARIALLLIAFVCAACGLVYELSLVTLGSYLLGDTAAQASITLSVMVFAMGIGALIAKPLRGHAAAAFVAVELALALLGGL SVLLLYAAYAWLNLYTTALIAAASVIGMLVGAEIPLLMELLQRIRRQAPGAAVADLFAADYVGALLGGLAFPFLLLPVFGQIRGSLLVGVLNAAAGLALV FVLFRDRLTRPARLLFTALTALVALVLGGSYVYADRFEATAQQALFAHPIVRHEQTRYQSIVLTESLSPFATPDVRLYLNGDLQFSSVDEYRYHEALVHP VLAGPRARVLVLGGGDGLALREILRYPDVAEVTLVELDPGMIHLARTEPRLLALNRHAFDDPRVRVINEDAFAWLRGQSGRFDAIIVDLPDPDQTSIAKL YTKEFYAMVAQVLAPGGTVAVQSGSPFFAPRSFWCIAATIRAGGLRVLPYHVDVPSFGDWGFVLATRDVEPVLRLDPPAPPRSIDAESLRAAAVFAPDRR DPGAPVSTLMHPVIVDLELREWR&LINK_LOC=protein&PAGE_TYPE=BlastSearch', 'BLAST this protein','NFA_34940')" />
   AVTLYVTFTFFGSSAIVLVLATALIGVLVGAEVPLLMTLLQSGRNDTDAESTGKVLANLNAADYAGALVGGLLWPFVLLPIAGMIRGAAITGIINLVAAA VVALILLRWQLSLRTRLFAVVALLVAAACIAVLLVRADGIETTSRQRLYTDPVVAAERSQYQEIVVTERGGDVRLFLDGDLQFSSIDEHRYTESLVYPAM ADDPKRVLILGGGDGLAAREVLRLPGVEEIVQVELDPAVIELANTRLSGLNQGALQDPRVHVVLDDAFRWLRDAPDSGFDAVIVDLPDPDTPALGRLYST EFYGLAAAALNPGGLMVVQSGSPYSTPDAYWRTTSTVASAGLAVTPYHVLVPSFGDWGYVLARRGPEPPALRLPRDVPELRFLDEPTLAASAIFPRDRPR RELEPSTLDRPRIVDDMRKGYER', 'http://blast.ncbi.nlm.nih.gov/Blast.cgi?PAGE=Proteins&PROGRAM=blastp&BLAST_PROGRAMS=blastp&QUERY=MTTTAAEPNTSTTRALGGRARAFLLAAVAACAACGLIYELALLTLSVSLTGGGITQTSLIVAGFVAALGVGALAAKPLLSTAAASFVVVEIVLGLVGGFS AVTLYVTFTFFGSSAIVLVLATALIGVLVGAEVPLLMTLLQSGRNDTDAESTGKVLANLNAADYAGALVGGLLWPFVLLPIAGMIRGAAITGIINLVAAA VVALILLRWQLSLRTRLFAVVALLVAAACIAVLLVRADGIETTSRQRLYTDPVVAAERSQYQEIVVTERGGDVRLFLDGDLQFSSIDEHRYTESLVYPAM ADDPKRVLILGGGDGLAAREVLRLPGVEEIVQVELDPAVIELANTRLSGLNQGALQDPRVHVVLDDAFRWLRDAPDSGFDAVIVDLPDPDTPALGRLYST EFYGLAAAALNPGGLMVVQSGSPYSTPDAYWRTTSTVASAGLAVTPYHVLVPSFGDWGYVLARRGPEPPALRLPRDVPELRFLDEPTLAASAIFPRDRPR RELEPSTLDRPRIVDDMRKGYER&LINK_LOC=protein&PAGE_TYPE=BlastSearch', 'BLAST this protein','RHA1_ro02313')" />
   AVTLYVTFTFFGSSALVLVLATALIGVLVGAEVPLLMTLLQSDRGDTDAESTGKVLANLNAADYAGALVGGLLWPFVLLPVAGMIRGAAITGIINLVAAA VVALILLRWQLSLRTRLLAVVALLVAAACIAVLLVRADGIETTSRQRLYTDPVVAAERSQYQEIVVTERGGDVRLFLDGDLQFSSVDEHRYTESLVYPAM ADDPKRVLILGGGDGLAAREVLRLPGVEDIVQVELDPAVIELANTRLSGLNQGALQNPRVHVVLDDAFRWLRDAPDSGFDAVIVDLPDPDTPALGRLYST EFYGLAAAALNPGGLMVVQSGSPYSTPDAYWRTTSTVASAGLAVTPYHVLVPSFGDWGYVLARRGPEPPALRLPHDAPELRFLDEQTLAASAIFPRDRPR RELEPSTLDRPRIVDDMRKGYER', 'http://blast.ncbi.nlm.nih.gov/Blast.cgi?PAGE=Proteins&PROGRAM=blastp&BLAST_PROGRAMS=blastp&QUERY=MNAPAVESDSRAARPLGPRARAFLLAAVAACAACGLIYELALLTLSVSLTGGGITQTSLIVAGFVAALGVGALAAKPLLSTAAASFVVVEIVLGLVGGFS AVTLYVTFTFFGSSALVLVLATALIGVLVGAEVPLLMTLLQSDRGDTDAESTGKVLANLNAADYAGALVGGLLWPFVLLPVAGMIRGAAITGIINLVAAA VVALILLRWQLSLRTRLLAVVALLVAAACIAVLLVRADGIETTSRQRLYTDPVVAAERSQYQEIVVTERGGDVRLFLDGDLQFSSVDEHRYTESLVYPAM ADDPKRVLILGGGDGLAAREVLRLPGVEDIVQVELDPAVIELANTRLSGLNQGALQNPRVHVVLDDAFRWLRDAPDSGFDAVIVDLPDPDTPALGRLYST EFYGLAAAALNPGGLMVVQSGSPYSTPDAYWRTTSTVASAGLAVTPYHVLVPSFGDWGYVLARRGPEPPALRLPHDAPELRFLDEQTLAASAIFPRDRPR RELEPSTLDRPRIVDDMRKGYER&LINK_LOC=protein&PAGE_TYPE=BlastSearch', 'BLAST this protein','ROP_20310')" />

 
 
   MRQLGDKIGAKLIAEEVGVPVAPWSRGAVETLETAIEAAGQIGYPLMLKATAGGGGRGIRVITNESELVDAYERTSQEAARAFGSGIVFLERLVTGARHV EVQVIADGQGTAWALGVRDCSIQRRNQKVIEESASPVLSAEQAAELKSSAERLAVRVGYRGAATVEFLYHPGDKQFAFLEVNTRLQVEHPITELTTGFDL VKAQLHVAGGGKLEGRPPAERGHAIEARLNAEDPDRDFAPSPGRIARLDLPAGPGIRVDTGVSEGDTIPADFDSMIAKVIAYGRDRDEALGRLRRAMAET SVVIEGGTTNKSFVLDLLDQPEVIDASADTGWIDRVRAEGRLVSHRNAAVALAAAAIDAYEDEERVERARLLSTASGGRPQVQHESGRPLDLKLRGVSYK VRVARVGAHRFRVGIEAGDGHGAQSQTADVVLDRFDRHTGQIAVNGSRFRLLTATHGSVHQVEVDGVTHRVSLDEGGVIRSPAPALVIATPLQVGAEVEA GAPVVVLESMKMETVLRAPFKARLKELAVTVGSQVETGAPLLRLEQIVEEDADEAAASASAQPTEAAELDLPAAPAEVPVRARVAQGQEDLRSLLLGFDV DPHDEDRILAGYVEARTTAVEQGLRPLSGELSLIEVFADLADLGRNRPAAAAEESNGESQLHSAHEYFHTYLQSLDVERAGLPESFQAALRKVLAHYGVT ELDRSPELEAAVFRIFLARQRSATDATVIAALMRAWLRELPPDELLREPTGLALERLIAATQVRFPNVADLARGVVYAWFGQPLLRRNRARVYAAIRKNL RYLDENPRAEDRDERVAEMVRSTEPLVRLLAQRLVHTELDNSAMLEVLTRRYYGNKGLTGVRTTEVGGNPFIVAEHDGSQLASAAVPFDRLGDALDGLAE LAASAGALDADIYLLWENQPDTDEAATALQRVISAHPLPQQVRRVTATVAGRRNSVMHHHFTFRRSTTGLTEERLIRGLHPFIAERMQLERLNKFDLTRL PSSDEDVYLYQCVARENPADDRLVAFTQVRDLTELREHDGRLVALPTAEDAIAACVDSIRRAQLRKPSNKRFSTNRVVVYLWPVSDIKRSELYLIAQRIL PSTVGAGLEEIELIGRQRSWKTGELIKIAVRVRFDTAGNPSLNVGEPTLSPIEPLDDYRLKVMRAASRGTVYPYELVDSLGDFTEHDLDEAHTLVPVDRP KGRNSAAMVAGVISTKTRRHRQGVTRVLLLGDPTKSLGALSEPECRRVIAALDLAEQMKVPVEWYALSSGARISMQSGTENMDWVAAALKRIVEFTQDGG EINVVVNGINVGAQPYWNAEATMLMHTRGVLVMTPDSAMVLTGKQALDFSGGVSAEDNFGIGGYDRVMGPNGQAQYWAPSLAAARDVLMSHYDHTYIAPG ETSPRRAGTDDPYDRDVSSFPHTFAGSDFTTVGEIFSAAANPDRKKAFDIRTVMRALSDQDHPVLERWAGMAGAETSVVQDVHLGGLPVCLVGIESRAVP RRGFPPTDGPDTYTAGTLFPQSSKKVARAINSASGNRPLVVLANLSGFDGSPESMRKLQLEYGAEIGRAIVNFEGPIVFCVISRYHGGAFVVFSKALNPN MTVLALEGSYASVLGGAPAAAVVFSGDVNSRTANDPRIRDLESRVTAASGTDRAALAAELDELRASVRAEKLGEVATEFDRVHSIQRAVQVGSVDEVIKA ADLRPKIIASIEARLG', 'http://blast.ncbi.nlm.nih.gov/Blast.cgi?PAGE=Proteins&PROGRAM=blastp&BLAST_PROGRAMS=blastp&QUERY=MRLIHAVRELSAQSATPIETVALHTDVDRDATFVREADIAFDLGPAAARPYLDLKVLEHALLESGADAAWVGWGFVAEDPAFAELCDRIGVTFIGPDAEA MRQLGDKIGAKLIAEEVGVPVAPWSRGAVETLETAIEAAGQIGYPLMLKATAGGGGRGIRVITNESELVDAYERTSQEAARAFGSGIVFLERLVTGARHV EVQVIADGQGTAWALGVRDCSIQRRNQKVIEESASPVLSAEQAAELKSSAERLAVRVGYRGAATVEFLYHPGDKQFAFLEVNTRLQVEHPITELTTGFDL VKAQLHVAGGGKLEGRPPAERGHAIEARLNAEDPDRDFAPSPGRIARLDLPAGPGIRVDTGVSEGDTIPADFDSMIAKVIAYGRDRDEALGRLRRAMAET SVVIEGGTTNKSFVLDLLDQPEVIDASADTGWIDRVRAEGRLVSHRNAAVALAAAAIDAYEDEERVERARLLSTASGGRPQVQHESGRPLDLKLRGVSYK VRVARVGAHRFRVGIEAGDGHGAQSQTADVVLDRFDRHTGQIAVNGSRFRLLTATHGSVHQVEVDGVTHRVSLDEGGVIRSPAPALVIATPLQVGAEVEA GAPVVVLESMKMETVLRAPFKARLKELAVTVGSQVETGAPLLRLEQIVEEDADEAAASASAQPTEAAELDLPAAPAEVPVRARVAQGQEDLRSLLLGFDV DPHDEDRILAGYVEARTTAVEQGLRPLSGELSLIEVFADLADLGRNRPAAAAEESNGESQLHSAHEYFHTYLQSLDVERAGLPESFQAALRKVLAHYGVT ELDRSPELEAAVFRIFLARQRSATDATVIAALMRAWLRELPPDELLREPTGLALERLIAATQVRFPNVADLARGVVYAWFGQPLLRRNRARVYAAIRKNL RYLDENPRAEDRDERVAEMVRSTEPLVRLLAQRLVHTELDNSAMLEVLTRRYYGNKGLTGVRTTEVGGNPFIVAEHDGSQLASAAVPFDRLGDALDGLAE LAASAGALDADIYLLWENQPDTDEAATALQRVISAHPLPQQVRRVTATVAGRRNSVMHHHFTFRRSTTGLTEERLIRGLHPFIAERMQLERLNKFDLTRL PSSDEDVYLYQCVARENPADDRLVAFTQVRDLTELREHDGRLVALPTAEDAIAACVDSIRRAQLRKPSNKRFSTNRVVVYLWPVSDIKRSELYLIAQRIL PSTVGAGLEEIELIGRQRSWKTGELIKIAVRVRFDTAGNPSLNVGEPTLSPIEPLDDYRLKVMRAASRGTVYPYELVDSLGDFTEHDLDEAHTLVPVDRP KGRNSAAMVAGVISTKTRRHRQGVTRVLLLGDPTKSLGALSEPECRRVIAALDLAEQMKVPVEWYALSSGARISMQSGTENMDWVAAALKRIVEFTQDGG EINVVVNGINVGAQPYWNAEATMLMHTRGVLVMTPDSAMVLTGKQALDFSGGVSAEDNFGIGGYDRVMGPNGQAQYWAPSLAAARDVLMSHYDHTYIAPG ETSPRRAGTDDPYDRDVSSFPHTFAGSDFTTVGEIFSAAANPDRKKAFDIRTVMRALSDQDHPVLERWAGMAGAETSVVQDVHLGGLPVCLVGIESRAVP RRGFPPTDGPDTYTAGTLFPQSSKKVARAINSASGNRPLVVLANLSGFDGSPESMRKLQLEYGAEIGRAIVNFEGPIVFCVISRYHGGAFVVFSKALNPN MTVLALEGSYASVLGGAPAAAVVFSGDVNSRTANDPRIRDLESRVTAASGTDRAALAAELDELRASVRAEKLGEVATEFDRVHSIQRAVQVGSVDEVIKA ADLRPKIIASIEARLG&LINK_LOC=protein&PAGE_TYPE=BlastSearch', 'BLAST this protein','Caci_4446')" />
   DSIRRARSRRPSDKRFNTNRIVVYVWPPSDITRTELEMIAERVLLMTADAELEEILFIAQQRSPQTGELSEIAVCVSFKTTGAELTVGEPSVEPVEPIDD YRLKVLRASNRKMMHPYELTDLLGDFVEYDLDDNCALVPVDRPKGHNTAAIVVGVATTPTRLHPQGVTRVVLLSDPTKSLGALSEPECRRVIAALNLAER RRLPLEWYALSSGARISMESGTENMDWVGAALKQIVEFTQAGGEINIVVTGITVGAQPYWNAEATMLMHTKGILVMIPDSTMVLTGKQAVDVSGGVSAED NFGIGGYDRVMGPNGQGQYWAPNLTAAVDMLMAYYNHTYVAPGEDGPRRAETNDPVDRDISDYPHSVAGSDFTSVGEIFSAEANPDRKKPFGIRPVMEAL SDQDHPVLERWKHMESAETAVVWDVHLGGIPVCLIGIESRAVPRHGFPPTDGPEIYTPGTLFPQSSKKVARAINAASGNRPLVVLANLSGFDGSPESMRK LQLEYGAEIGRAIVNFRGPIVFCVISRYHGGAFVVFSKALNPNMTVLALEGSFASVLGGAPAAAVVFAREVDARTAADPRVRGLEARVAAATGADHTALT AELDELRVSVHAEKHREIATEFDRRHTIQRAVEVGSVDAVIPPAELRPRVIEAIEASKPVGADPENEVR', 'http://blast.ncbi.nlm.nih.gov/Blast.cgi?PAGE=Proteins&PROGRAM=blastp&BLAST_PROGRAMS=blastp&QUERY=MTHHFTFRPSDAGMAEERLIRGLHPCIAQRMQLERLSKFDLTRLPSSDEDVYLYQCVARENPSDNRFVAFIQVRDLFREHDHDGQLVALPAAEDAVAACV DSIRRARSRRPSDKRFNTNRIVVYVWPPSDITRTELEMIAERVLLMTADAELEEILFIAQQRSPQTGELSEIAVCVSFKTTGAELTVGEPSVEPVEPIDD YRLKVLRASNRKMMHPYELTDLLGDFVEYDLDDNCALVPVDRPKGHNTAAIVVGVATTPTRLHPQGVTRVVLLSDPTKSLGALSEPECRRVIAALNLAER RRLPLEWYALSSGARISMESGTENMDWVGAALKQIVEFTQAGGEINIVVTGITVGAQPYWNAEATMLMHTKGILVMIPDSTMVLTGKQAVDVSGGVSAED NFGIGGYDRVMGPNGQGQYWAPNLTAAVDMLMAYYNHTYVAPGEDGPRRAETNDPVDRDISDYPHSVAGSDFTSVGEIFSAEANPDRKKPFGIRPVMEAL SDQDHPVLERWKHMESAETAVVWDVHLGGIPVCLIGIESRAVPRHGFPPTDGPEIYTPGTLFPQSSKKVARAINAASGNRPLVVLANLSGFDGSPESMRK LQLEYGAEIGRAIVNFRGPIVFCVISRYHGGAFVVFSKALNPNMTVLALEGSFASVLGGAPAAAVVFAREVDARTAADPRVRGLEARVAAATGADHTALT AELDELRVSVHAEKHREIATEFDRRHTIQRAVEVGSVDAVIPPAELRPRVIEAIEASKPVGADPENEVR&LINK_LOC=protein&PAGE_TYPE=BlastSearch', 'BLAST this protein','Francci3_0988')" />
   ERLGVTFIGPSSEAMRKLGDKIGAKRIAEEVGVPVAPWSRGPVESLDAALAAAAKIGYPLMLKAAAGGGGRGIRVIRDESELVNAFERTRQEAARAFGID VMFLERLITGARHVEVQVIADGQGTAWALGVRDCSVQRRNQKVIEESASPVLSPEQAANLKAAAERLIVAVGYRGAATVEFLYHPGDKQVTFLEVNTRLQ VEHPITEATTGFDLVKAQLHVASAGRLQGVRPVERGHAVEARLNAEDPDRDFMPCPGHITRLELPAGPGIRVDTGFSEGDTIPADFDSMIVKIIAYGRDR DGALGRLRRAIQETKVIIKGGVTNKSFLLDLLNRPELIDASADTGWIDRVGDGLVSHRHSAVALVAAAIDAYEEKEHVERQRLLLTAFGGRPQVQHDSAR PLDLKLRDVGYRARVARRGPCRFHVSLEAGAEVRTADVKIDRFDRHTGQIVVNGARYRLLADIHGPVHLIEVDGVTHRVSRDEGDVVRSPMPAMVIATPL EVGAEIEAGAQILVLESMKMETVLQAPFKARVREYFVSVGSQVEAGAPLLRLEPIAGAEVEDSPAAAAAGLDLPTAPEEVPARERTARGLEDLRALLLGF DLHDERQVLHDYLAARRAATEEGHQPLAKELEIIEIFADLAELSRNRPARMDVGDEGHLRNPHEHFHTYLRSLDVERAGLPEAFQAKLAKALGHYDVTDL KRSPELEAAVFRIFLARQPATAKVAAKVVAALLESWRWEPPPDKSVHKPVRLALERLVAATKGHFPVVADLALDVMFAWFQHQQLLRRAQERIRIFEHLL HLDAYPDAPDRTERIAEMVRSTEPLVPLLGQRLLRGDRDNTVILEVLTRRYYGNKDLTHIRATEYAGYRFWVAERAGSRLVSCAVGFDALDAALGGLAEL AGGERSVDADIYLSWENQPADSDVMAAALGEAASAHPLPPQVRRLTITITPEAAAR', 'http://blast.ncbi.nlm.nih.gov/Blast.cgi?PAGE=Proteins&PROGRAM=blastp&BLAST_PROGRAMS=blastp&QUERY=MFRCVAIVNRGEAAMRLIRAVREIVAETATAIETVALYTDVDRTATFVREADRAYCLGPAAARPYLDLRVLERALVETGADAAWVGWGFVAEDPAFEELC ERLGVTFIGPSSEAMRKLGDKIGAKRIAEEVGVPVAPWSRGPVESLDAALAAAAKIGYPLMLKAAAGGGGRGIRVIRDESELVNAFERTRQEAARAFGID VMFLERLITGARHVEVQVIADGQGTAWALGVRDCSVQRRNQKVIEESASPVLSPEQAANLKAAAERLIVAVGYRGAATVEFLYHPGDKQVTFLEVNTRLQ VEHPITEATTGFDLVKAQLHVASAGRLQGVRPVERGHAVEARLNAEDPDRDFMPCPGHITRLELPAGPGIRVDTGFSEGDTIPADFDSMIVKIIAYGRDR DGALGRLRRAIQETKVIIKGGVTNKSFLLDLLNRPELIDASADTGWIDRVGDGLVSHRHSAVALVAAAIDAYEEKEHVERQRLLLTAFGGRPQVQHDSAR PLDLKLRDVGYRARVARRGPCRFHVSLEAGAEVRTADVKIDRFDRHTGQIVVNGARYRLLADIHGPVHLIEVDGVTHRVSRDEGDVVRSPMPAMVIATPL EVGAEIEAGAQILVLESMKMETVLQAPFKARVREYFVSVGSQVEAGAPLLRLEPIAGAEVEDSPAAAAAGLDLPTAPEEVPARERTARGLEDLRALLLGF DLHDERQVLHDYLAARRAATEEGHQPLAKELEIIEIFADLAELSRNRPARMDVGDEGHLRNPHEHFHTYLRSLDVERAGLPEAFQAKLAKALGHYDVTDL KRSPELEAAVFRIFLARQPATAKVAAKVVAALLESWRWEPPPDKSVHKPVRLALERLVAATKGHFPVVADLALDVMFAWFQHQQLLRRAQERIRIFEHLL HLDAYPDAPDRTERIAEMVRSTEPLVPLLGQRLLRGDRDNTVILEVLTRRYYGNKDLTHIRATEYAGYRFWVAERAGSRLVSCAVGFDALDAALGGLAEL AGGERSVDADIYLSWENQPADSDVMAAALGEAASAHPLPPQVRRLTITITPEAAAR&LINK_LOC=protein&PAGE_TYPE=BlastSearch', 'BLAST this protein','Francci3_0989')" />
   CNQLGITFIGPPAESMRLLGDKVEAKLLAEKVDVPVAPWSGGPVATRADARRHAQAIGYPLIIKARSGGGGRGIRKVWSEDELELALERTQGEAQRSFGD PVVFIERLVTDARHVEVQVIADQHGNVWAPGVRDCSIQRKNQKVIEESSSPLLTAEQSNNLRAVSADLVRAAGYHGAATVEYLYQPEQKSFTFLEVNTRL QVEHPITEATTGIDLVKLQILVANGERLVGDCPPEFGHAVEARLNAEDAANGFAPAPGAVQLLKFPLGSGLRVDTGIAQGDVIPPDYDSMVAKVIAWGRD RPEALARLRTALRETTVVLQGGTTTKSFLLELLDRDEVISASADTGWLDRTGVGSVNTPTKVADVALIAAAIDVYDAEEKLEREAFLSSARGGRPRAGHA IGRKVELSYQGQAYGLTVTQVGAHRYRVDGDSPDVEVEVDRLSEFESRLVVGTRRHHVVSVAGRAQYLVEVDGISHQISQDEAGVVRAPAPAVVVAVPVS VGDEVEAGDTLVVLESMKMETAVRAPVAGRVREVLAMVNSQVDAGTALLRVEETAEESAAPKSPRVEFEVAAPASKGDVRTDALEQIDALGALLTGYDVS AKRAGVLLAGYEKLRAEIAGDADLVQAELALLNTFADICEIGRNRPTVAEEDGDERVHSPREHFHTYLHTLDVELEGLPESFTTRLSRALRHYDVTDLER TGELEEAVYRLFLAQQRMDNQVPVIAALLDRWLNDGNAPGRAPSGLGEVLDRLIIATQVRYPVIGNVARNVRFRFFDEPQIRKAREQVYDGVRGSLEYLA ENPGAPDYQERLEALVATPQSLTELLGQRIARKSTTVGPLLEVVTRRYYDIRTLEDVTAFERHGRRFVTGNFDLLGERLNLVSAVAEYAELPGALDEIGA VASENPENLVLDLYLSWTAPPAGPDTMSEDLRKALTALPLAATCRRVTVSVFGGTDVDVRKFTYRPDAGVLAEEKLIRDMHPLTGQRLDLWRLKNFDGTR LPASAETFLFNLVSRENPTDERLIALAEVRDITPVRNEDGQVIGVPAAERVLQSCLDGIRRAQATRGSKRKLDANRVVLYVWPKIDVPVEDLEAFARVAA PLTAGAGLEEITVIARLQGEPGTESREKALRFSYRTGAGVVVKVTEPPTEPLQPLDAYTQKVRSSRARGTVYPYELIPLLTGRDGSFVEHDLDESGQLVP VERPYGQNKAGLVVGLVTARTDRHPEGMTRVALFGDPTKALGTVAEAECARLVAGVDLAEKLGVPVEWFALSSGATISMDSGTENMDWVSRGLRRLITFT QDGGEVNVIVAGINVGAQPYWNAEATMLMHTKGILVMTPDSAMVLTGKNSLDYSGGVSAEDNFGIGGYDRVMGPNGEAQYWAPNLPAACEILFAHYDHAY VVAGERFPRRAQTSDPIGRDVRSFPHIHPSSDFTTVGDIFSAETNPERKKPFDIRTVMRSLVDQDHTVLERWADMADADTSVVFDAHLGGYPVSVIGIES RAIARKGWSPANGPDQWTAGTLFPQSSKKTARAINAASGSRPLVVLANLSGFDGSPDSLRHIQLEYGAEIGRAIVNFDGPIVFCVVSRYHGGAFVVFSGA LNDNMEVLAVEGSFASVLGGAPAAAVVFTREVNSRVAADPSIRELEANLAGAQNDAQQAHLRVELAAQQAAVRNEKLGEVAAEFEAVHNIQRAQRVGSVD AVIPAVELRPYIIGAVERGMRRAVEAGK', 'http://blast.ncbi.nlm.nih.gov/Blast.cgi?PAGE=Proteins&PROGRAM=blastp&BLAST_PROGRAMS=blastp&QUERY=MFKRIAIVNRGEAAVRLIRAVRELNAEHNYGIRTIALHTEAERRAMFVRQADEGVCLRTVKTGTAYLDHAELARALRESKADAVWVGWGFVAEDPAFVEV CNQLGITFIGPPAESMRLLGDKVEAKLLAEKVDVPVAPWSGGPVATRADARRHAQAIGYPLIIKARSGGGGRGIRKVWSEDELELALERTQGEAQRSFGD PVVFIERLVTDARHVEVQVIADQHGNVWAPGVRDCSIQRKNQKVIEESSSPLLTAEQSNNLRAVSADLVRAAGYHGAATVEYLYQPEQKSFTFLEVNTRL QVEHPITEATTGIDLVKLQILVANGERLVGDCPPEFGHAVEARLNAEDAANGFAPAPGAVQLLKFPLGSGLRVDTGIAQGDVIPPDYDSMVAKVIAWGRD RPEALARLRTALRETTVVLQGGTTTKSFLLELLDRDEVISASADTGWLDRTGVGSVNTPTKVADVALIAAAIDVYDAEEKLEREAFLSSARGGRPRAGHA IGRKVELSYQGQAYGLTVTQVGAHRYRVDGDSPDVEVEVDRLSEFESRLVVGTRRHHVVSVAGRAQYLVEVDGISHQISQDEAGVVRAPAPAVVVAVPVS VGDEVEAGDTLVVLESMKMETAVRAPVAGRVREVLAMVNSQVDAGTALLRVEETAEESAAPKSPRVEFEVAAPASKGDVRTDALEQIDALGALLTGYDVS AKRAGVLLAGYEKLRAEIAGDADLVQAELALLNTFADICEIGRNRPTVAEEDGDERVHSPREHFHTYLHTLDVELEGLPESFTTRLSRALRHYDVTDLER TGELEEAVYRLFLAQQRMDNQVPVIAALLDRWLNDGNAPGRAPSGLGEVLDRLIIATQVRYPVIGNVARNVRFRFFDEPQIRKAREQVYDGVRGSLEYLA ENPGAPDYQERLEALVATPQSLTELLGQRIARKSTTVGPLLEVVTRRYYDIRTLEDVTAFERHGRRFVTGNFDLLGERLNLVSAVAEYAELPGALDEIGA VASENPENLVLDLYLSWTAPPAGPDTMSEDLRKALTALPLAATCRRVTVSVFGGTDVDVRKFTYRPDAGVLAEEKLIRDMHPLTGQRLDLWRLKNFDGTR LPASAETFLFNLVSRENPTDERLIALAEVRDITPVRNEDGQVIGVPAAERVLQSCLDGIRRAQATRGSKRKLDANRVVLYVWPKIDVPVEDLEAFARVAA PLTAGAGLEEITVIARLQGEPGTESREKALRFSYRTGAGVVVKVTEPPTEPLQPLDAYTQKVRSSRARGTVYPYELIPLLTGRDGSFVEHDLDESGQLVP VERPYGQNKAGLVVGLVTARTDRHPEGMTRVALFGDPTKALGTVAEAECARLVAGVDLAEKLGVPVEWFALSSGATISMDSGTENMDWVSRGLRRLITFT QDGGEVNVIVAGINVGAQPYWNAEATMLMHTKGILVMTPDSAMVLTGKNSLDYSGGVSAEDNFGIGGYDRVMGPNGEAQYWAPNLPAACEILFAHYDHAY VVAGERFPRRAQTSDPIGRDVRSFPHIHPSSDFTTVGDIFSAETNPERKKPFDIRTVMRSLVDQDHTVLERWADMADADTSVVFDAHLGGYPVSVIGIES RAIARKGWSPANGPDQWTAGTLFPQSSKKTARAINAASGSRPLVVLANLSGFDGSPDSLRHIQLEYGAEIGRAIVNFDGPIVFCVVSRYHGGAFVVFSGA LNDNMEVLAVEGSFASVLGGAPAAAVVFTREVNSRVAADPSIRELEANLAGAQNDAQQAHLRVELAAQQAAVRNEKLGEVAAEFEAVHNIQRAQRVGSVD AVIPAVELRPYIIGAVERGMRRAVEAGK&LINK_LOC=protein&PAGE_TYPE=BlastSearch', 'BLAST this protein','ROP_41490')" />
   DRLGVTFIGPSAEAMRKLGDKIGAKLLAEEVGVPVAPWSRGPVEDLDAARAAAERIGYPLMLKATAGGGGRGIRVVSSEAELVDAYERTRSEAERAFGSG VVFLERLVTGARHVEVQVIADGQGTAWALGVRDCSVQRRNQKVIEESASPLLTPEQTEELKASAERLALAVGYRGAATVEFLYQPAEKLFAFLEVNTRLQ VEHPITEATTGMDLVKAQLHVAAGGTLDGQRPREIGHAVEARLNAEDPDRDFAPAPGRISLLNFPSGPGIRVDTGVSEGDTIPADFDSMIAKIIAYGRDR DEALARLRRAVADTDVLIEGGSTNKSFLLDLLDAPEVIDGSADTGWIDRMRAQGRLQATRHSGVALAVAAIDAYTEAEQAEQQHLLSTARGGRPQVRHTG SRAVELKLRGTSYRITVARTAPQRFRVEIEDGPTAHTVDVEIERHGTATGQLLVHGRRYRFVTGTHGPVHVVEIDGTVHRISRDEGGIVRSPAPALVVAA PVDVGSEVEAGAPVLVLESMKMETVLRAPFRAVLRERPVSVGSQVEAGAPLLRLEPLDTDEVDADAGGQREDLGIPPRVEAVDAEQRAERARAELRSRLL GYDLDLDDEARALDDYLAARAELVEAGRRPVKAEGELLRVFADLSELSRNKPVDGEGSASTRLHSPHEYFHTYLQSLDVDRAGLSEVFQQRLTRALAHYG VTDLEQGPELTEAVFRLFCVHQRAGGDIGVVTALLRQWLAEPAPEQSVRETIGHVLEHVVVATQVRFPVVADLARSVVFRWFAQPMLRRRRAELYANVRR HLRYLDAHPDAADRSERIAEMVSCSEPLVRVLGQRIHRQGADLGPLLEVILRRYYRDRSLANVQVRRVADFSCVTADYELDSERVRVVSTAADPQRVVDA LHAVSEAADDATDTGLVVDLYLSWSDEFADDDTAAAKLLATLTEVSLPDSVRRVTTTVAGRSGSVMHHHYTFRASASGLVEDRLIRGLHPLVAQRLQLRR FSEFDLTRLPSADEEVYLMRCVAPDNPSDERFVALAQVRDLTPLRDSQGRLVALPAVEGTLAACLDAIRAQQVRRPVKKRLDTNRVVIYVWPPIELTTEE LRAVANRVVPTTTGAGVEEVEFLGRRRDPETGATRDVAVLITHNPGSGVSVRMTTPSTEVIRPLDAYGQKVLRARRRNTVYPYELVELVAGKNGTFVEYD LDDEGELVPVDRPKGGNTAGIVVGVVTTPTKLYPEGVTRVVLLGDPTKSLGALAEPECARVIAALDLAERMRVPVEWFALSAGARIAMDSGTENMDWVAA ALKRIVHFTQDGGEINVVVAGINVGAQPYWNAEATMLMHTKGILVMTPDSAMVLTGKQSLDFSGGVSAEDNFGIGGYDRVMGPNGQAQYWAPNLAGACDV LLSHYEHTYVVPGERGPRRAETTDPRDRDVRDYPHNVVDSEFTTVGEIFSAEKNPDRKKPFDIRTVMRAVADADHPVLERWAGMADAETAVVQDARLGGW PVCLVGIESRSVPRRGFPPTDGPDTYTAGTLFPRSSKKVARAINAASGNRPLVVLANLSGFDGSPESMRKLQLEYGAEIGRAIVNFEGPIVFCVISRYHG GAFVVFSKALNPNMTVLAVEGSFASVLGGAPAAAVVFSGEVDARTAADERVRKLEERLTRADDEERAELATRLAEVKASVRAEKLGEVAAEFDNVHSIQR AVEVGSVDAVIPPESLRPELIAAVERGLRR', 'http://blast.ncbi.nlm.nih.gov/Blast.cgi?PAGE=Proteins&PROGRAM=blastp&BLAST_PROGRAMS=blastp&QUERY=MFRRVAIVNRGEAAMRLVHAVKEHNAEGGSRLETVAFYTDADREATFVREADHAYCLGPASTRPYLNLGILEQALRETEADAAWVGWGFVAEDPAFAELC DRLGVTFIGPSAEAMRKLGDKIGAKLLAEEVGVPVAPWSRGPVEDLDAARAAAERIGYPLMLKATAGGGGRGIRVVSSEAELVDAYERTRSEAERAFGSG VVFLERLVTGARHVEVQVIADGQGTAWALGVRDCSVQRRNQKVIEESASPLLTPEQTEELKASAERLALAVGYRGAATVEFLYQPAEKLFAFLEVNTRLQ VEHPITEATTGMDLVKAQLHVAAGGTLDGQRPREIGHAVEARLNAEDPDRDFAPAPGRISLLNFPSGPGIRVDTGVSEGDTIPADFDSMIAKIIAYGRDR DEALARLRRAVADTDVLIEGGSTNKSFLLDLLDAPEVIDGSADTGWIDRMRAQGRLQATRHSGVALAVAAIDAYTEAEQAEQQHLLSTARGGRPQVRHTG SRAVELKLRGTSYRITVARTAPQRFRVEIEDGPTAHTVDVEIERHGTATGQLLVHGRRYRFVTGTHGPVHVVEIDGTVHRISRDEGGIVRSPAPALVVAA PVDVGSEVEAGAPVLVLESMKMETVLRAPFRAVLRERPVSVGSQVEAGAPLLRLEPLDTDEVDADAGGQREDLGIPPRVEAVDAEQRAERARAELRSRLL GYDLDLDDEARALDDYLAARAELVEAGRRPVKAEGELLRVFADLSELSRNKPVDGEGSASTRLHSPHEYFHTYLQSLDVDRAGLSEVFQQRLTRALAHYG VTDLEQGPELTEAVFRLFCVHQRAGGDIGVVTALLRQWLAEPAPEQSVRETIGHVLEHVVVATQVRFPVVADLARSVVFRWFAQPMLRRRRAELYANVRR HLRYLDAHPDAADRSERIAEMVSCSEPLVRVLGQRIHRQGADLGPLLEVILRRYYRDRSLANVQVRRVADFSCVTADYELDSERVRVVSTAADPQRVVDA LHAVSEAADDATDTGLVVDLYLSWSDEFADDDTAAAKLLATLTEVSLPDSVRRVTTTVAGRSGSVMHHHYTFRASASGLVEDRLIRGLHPLVAQRLQLRR FSEFDLTRLPSADEEVYLMRCVAPDNPSDERFVALAQVRDLTPLRDSQGRLVALPAVEGTLAACLDAIRAQQVRRPVKKRLDTNRVVIYVWPPIELTTEE LRAVANRVVPTTTGAGVEEVEFLGRRRDPETGATRDVAVLITHNPGSGVSVRMTTPSTEVIRPLDAYGQKVLRARRRNTVYPYELVELVAGKNGTFVEYD LDDEGELVPVDRPKGGNTAGIVVGVVTTPTKLYPEGVTRVVLLGDPTKSLGALAEPECARVIAALDLAERMRVPVEWFALSAGARIAMDSGTENMDWVAA ALKRIVHFTQDGGEINVVVAGINVGAQPYWNAEATMLMHTKGILVMTPDSAMVLTGKQSLDFSGGVSAEDNFGIGGYDRVMGPNGQAQYWAPNLAGACDV LLSHYEHTYVVPGERGPRRAETTDPRDRDVRDYPHNVVDSEFTTVGEIFSAEKNPDRKKPFDIRTVMRAVADADHPVLERWAGMADAETAVVQDARLGGW PVCLVGIESRSVPRRGFPPTDGPDTYTAGTLFPRSSKKVARAINAASGNRPLVVLANLSGFDGSPESMRKLQLEYGAEIGRAIVNFEGPIVFCVISRYHG GAFVVFSKALNPNMTVLAVEGSFASVLGGAPAAAVVFSGEVDARTAADERVRKLEERLTRADDEERAELATRLAEVKASVRAEKLGEVAAEFDNVHSIQR AVEVGSVDAVIPPESLRPELIAAVERGLRR&LINK_LOC=protein&PAGE_TYPE=BlastSearch', 'BLAST this protein','Svir_23180')" />

 
 
   DATWLLPAPLPRRTLLTPALVLTTVVALVVGAVIGVAVTGHIAARPTGLLVLGAGGTTGAAAAVGVALLAFTCQRRPSTARFADGIGIAVGVGGLVLAAA LRATGEPGLPAVRLPPEAVLAVGAAALAAGLIAVGSTWRALESWPAAPIAGASITMGAYADVVYAVEPSYLTEMSARRFWRSRTRVRTTRLLPARRLPPL VAQDLIILRRKAARLWWPVGAAAVPVYLADGPAWLLIGVVVIGALAAASLTAESTHDDAANPAMLRLLGVTGRQVTAARLVVPAVVAGGWLAVTLAVLQA LGALTGPWWVLGLLAGPAVAAGAFQRARASAASIGTVLIDTPLGAFPAGMLLWLLNGIDVLAVLTLPISVGLVLLPAPEVLGWHWLLAQAVSSALGYALL VRRSGTTAGSF', 'http://blast.ncbi.nlm.nih.gov/Blast.cgi?PAGE=Proteins&PROGRAM=blastp&BLAST_PROGRAMS=blastp&QUERY=MTPIAAPARPASLDPAHARRWIRRRRIAAEGRADVGTVYAAILALLMAAALVGPPVAGVIWPPDMPGGGPTVTPFVGAVAALAAFFVVLRQLGPLSVSRS DATWLLPAPLPRRTLLTPALVLTTVVALVVGAVIGVAVTGHIAARPTGLLVLGAGGTTGAAAAVGVALLAFTCQRRPSTARFADGIGIAVGVGGLVLAAA LRATGEPGLPAVRLPPEAVLAVGAAALAAGLIAVGSTWRALESWPAAPIAGASITMGAYADVVYAVEPSYLTEMSARRFWRSRTRVRTTRLLPARRLPPL VAQDLIILRRKAARLWWPVGAAAVPVYLADGPAWLLIGVVVIGALAAASLTAESTHDDAANPAMLRLLGVTGRQVTAARLVVPAVVAGGWLAVTLAVLQA LGALTGPWWVLGLLAGPAVAAGAFQRARASAASIGTVLIDTPLGAFPAGMLLWLLNGIDVLAVLTLPISVGLVLLPAPEVLGWHWLLAQAVSSALGYALL VRRSGTTAGSF&LINK_LOC=protein&PAGE_TYPE=BlastSearch', 'BLAST this protein','Sare_4897')" />
   ATWLLPAPVPRRTLLAPALALTTTAALVVGAVIGVAVTGHIAARPTGLLVLGAGGATGAAAAVFVALLAIAGQHRPSVTRFADGAGIAVGAGSLVVAAAL SAHGEHRLPFPPVPPVAVLAAGVAALATGLILVGLTWRELESWPAAPIAEASINAGAYADVVYAVEPSYLTEMSARRFWRSRTRIRTTRLLRTGWLSPLI AQDLIILRRKAPRLWWSVGAAAVPVYLAEGPAWLLIGVVLIGALGAAGFTGESTHSDAANPAMLRLLGVTGRRVTAARLVVPAVIAGGWLAITLAVLQGL GSLTGPWWVLGLLAGPAVAVGAYQRAKASAAALGTILIDTPLGAFPAGMLLWLLNGIDVLAVLTLPVSVGLVMLPAPEVLGWPWLLAQVVSSALGCALLV RFSARATHLF', 'http://blast.ncbi.nlm.nih.gov/Blast.cgi?PAGE=Proteins&PROGRAM=blastp&BLAST_PROGRAMS=blastp&QUERY=MTPVAAPVRPASPATVDARRWVRRRRIAAEGRADVGTVYTVALAVLMAIVLVRQPVARAVWPPEAPGDPAVTPFIGAVAALAAFFVVLRQLGPLSVSRSD ATWLLPAPVPRRTLLAPALALTTTAALVVGAVIGVAVTGHIAARPTGLLVLGAGGATGAAAAVFVALLAIAGQHRPSVTRFADGAGIAVGAGSLVVAAAL SAHGEHRLPFPPVPPVAVLAAGVAALATGLILVGLTWRELESWPAAPIAEASINAGAYADVVYAVEPSYLTEMSARRFWRSRTRIRTTRLLRTGWLSPLI AQDLIILRRKAPRLWWSVGAAAVPVYLAEGPAWLLIGVVLIGALGAAGFTGESTHSDAANPAMLRLLGVTGRRVTAARLVVPAVIAGGWLAITLAVLQGL GSLTGPWWVLGLLAGPAVAVGAYQRAKASAAALGTILIDTPLGAFPAGMLLWLLNGIDVLAVLTLPVSVGLVMLPAPEVLGWPWLLAQVVSSALGCALLV RFSARATHLF&LINK_LOC=protein&PAGE_TYPE=BlastSearch', 'BLAST this protein','Strop_4422')" />

 
 
   SVYATFTPDQVAFVAGDVGARIVVLGGPADLARWEPVLDGLPGISKVVMLEGAPSGDRFLGWEEFLALGRARLAEDPASIEDRWRAVTADDTLTVLYTSG TTGNPKGVPLTHANVFFEVAATSRMVALPDRGTQISYLTYAHIAERVLSLYLPLFKISHTHFCTDLAQLGATLGQVKPVLFFGVPRVWEKMMARLQALLA TQPEEQQENVRNAMAAGLAHVEASQYGRTPSPEVQAAYEKADAALLSIIRSMIGLDNAAWLASAAAPMPLEVQRFFAGLGMRVIDVYGMTETTGAFTANA PDRFKLGTVGQAGPGVEVRIAEDGEIVTRSPANARGYLNRPEATAELLDEDGWLHTGDVGSIDEDGFVRIVDRKKELIITSGGENISPANIENYLKEHPL VGQALAYGDGRPYPVAVLTLDGEVAPGWAQGRGIEFTTLADLAEHPDVLKVVEAAVATANDKLARVQQVKRWRLLPVEWTAETEELTPSLKLKRRVIHAK YAEIIDGMYESS', 'http://blast.ncbi.nlm.nih.gov/Blast.cgi?PAGE=Proteins&PROGRAM=blastp&BLAST_PROGRAMS=blastp&QUERY=MAGVLEERAAIEREIAGRTVCEQLRETAERNPDAPAYSDPVEGGWATLTYAEARQRILEIAAGFVALGLRPGEAVALMMVNRSEHVLADLGAVHAGGVPC SVYATFTPDQVAFVAGDVGARIVVLGGPADLARWEPVLDGLPGISKVVMLEGAPSGDRFLGWEEFLALGRARLAEDPASIEDRWRAVTADDTLTVLYTSG TTGNPKGVPLTHANVFFEVAATSRMVALPDRGTQISYLTYAHIAERVLSLYLPLFKISHTHFCTDLAQLGATLGQVKPVLFFGVPRVWEKMMARLQALLA TQPEEQQENVRNAMAAGLAHVEASQYGRTPSPEVQAAYEKADAALLSIIRSMIGLDNAAWLASAAAPMPLEVQRFFAGLGMRVIDVYGMTETTGAFTANA PDRFKLGTVGQAGPGVEVRIAEDGEIVTRSPANARGYLNRPEATAELLDEDGWLHTGDVGSIDEDGFVRIVDRKKELIITSGGENISPANIENYLKEHPL VGQALAYGDGRPYPVAVLTLDGEVAPGWAQGRGIEFTTLADLAEHPDVLKVVEAAVATANDKLARVQQVKRWRLLPVEWTAETEELTPSLKLKRRVIHAK YAEIIDGMYESS&LINK_LOC=protein&PAGE_TYPE=BlastSearch', 'BLAST this protein','Sros_5953')" />
   TVYATFAPEQVAYVAKDVGAVVAVLGGPADLARWEPVLGELPRLRKIIMLEGAPSGDDRFLSWREFLAMGAEALAADPGAVEARWRAVTPDDVLTVLYTS GTTGHPKGVPLTHANVLYEVATTDRIVRLPFGGTQISYLTYAHIAERVLSLYLPLFKRTHIYFCPDMTQLAAVLGEVRPVMFFGVPRVWEKIASKLRALL TLQPAEQREKVAQAMAAGLAFIEAGQYGRTVTPEVRAAYGQADAAVLSGIRSLIGLDRAGWLATAAAPMPQDVQRFFAGLGLKILDVYGMTETTGAITSN TPTAYKLGTVGRAEPGVEVRIAEDGEILTRSPLNTRGYLNRPEATAELLDADGWLHTGDVGTMDEDGFLRIIDRKKELFITSGGENISPANIENHLKEHP LIGQALAYGEGRPYPIALLTLDPEVAPEWARARGISFGSLAELATHPDVLKEVEAGVEAANRRLARVQQVKRWRLLPDEWTPDSGELTPTLKLKRRRIHA KYADVIDDLYGGA', 'http://blast.ncbi.nlm.nih.gov/Blast.cgi?PAGE=Proteins&PROGRAM=blastp&BLAST_PROGRAMS=blastp&QUERY=MASVLDERAEIEREIAGRTVCDQLLKVAEEHPDDPAYSDRTGDGWATLTFAQARRRVLEIAAGFAALGVQPGDAIALMMPNRSEHVLADLGAVHARALPC TVYATFAPEQVAYVAKDVGAVVAVLGGPADLARWEPVLGELPRLRKIIMLEGAPSGDDRFLSWREFLAMGAEALAADPGAVEARWRAVTPDDVLTVLYTS GTTGHPKGVPLTHANVLYEVATTDRIVRLPFGGTQISYLTYAHIAERVLSLYLPLFKRTHIYFCPDMTQLAAVLGEVRPVMFFGVPRVWEKIASKLRALL TLQPAEQREKVAQAMAAGLAFIEAGQYGRTVTPEVRAAYGQADAAVLSGIRSLIGLDRAGWLATAAAPMPQDVQRFFAGLGLKILDVYGMTETTGAITSN TPTAYKLGTVGRAEPGVEVRIAEDGEILTRSPLNTRGYLNRPEATAELLDADGWLHTGDVGTMDEDGFLRIIDRKKELFITSGGENISPANIENHLKEHP LIGQALAYGEGRPYPIALLTLDPEVAPEWARARGISFGSLAELATHPDVLKEVEAGVEAANRRLARVQQVKRWRLLPDEWTPDSGELTPTLKLKRRRIHA KYADVIDDLYGGA&LINK_LOC=protein&PAGE_TYPE=BlastSearch', 'BLAST this protein','Tbis_1953')" />

 
 
   LIRLRVPELDRRPPGDLAARVTADSTLLQNAATEGLVMVANGLITALGAVVIMGVLHAGLLAVTLGVLLTVALLLLVVLPRIRAAVARAQSSVGAIGAAL DRVLGAARTVKANGAEARETERANAAVESAYLAGLRGARHVAVVKVLTGVAVQGAFLAVLGVGGALVAAGGLTAPDLIAFLLYVFSLAAPLAALVGGLSA LQQGLGAVGRIHEVAAMPVEDDVDLPVPETTRTAPPSVEFRDVGFAHPGRAQALRGVSFTADPGARTALVGPSGAGKSTSFALLQRFVEPDSGTILLDGV DITTIPRAHLRATIAHVEQDAPVLAGTLRENLLYAEPNATDAEVDRVLRVTMLKSFVDDLERGLDTEVGTRGVALSGGERQRLAIARALLRRPGVLLLDE ATAQLDARNETALREAVDRAALGCTVILIAHRLSTVTDAERIVVLEQGRVRASGTHAELVRGDDLYRELATTQLLAGEAGASGRADAPGEAMPAR', 'http://blast.ncbi.nlm.nih.gov/Blast.cgi?PAGE=Proteins&PROGRAM=blastp&BLAST_PROGRAMS=blastp&QUERY=MTAPTPGALRVLLAYARPHRGVLLASLALVLVASATGLAQPLVARDVLSGLGGDGGVLGPVLLLAALVVVGALLTGLQSWWQQRTAERVVREVRRDLVHR LIRLRVPELDRRPPGDLAARVTADSTLLQNAATEGLVMVANGLITALGAVVIMGVLHAGLLAVTLGVLLTVALLLLVVLPRIRAAVARAQSSVGAIGAAL DRVLGAARTVKANGAEARETERANAAVESAYLAGLRGARHVAVVKVLTGVAVQGAFLAVLGVGGALVAAGGLTAPDLIAFLLYVFSLAAPLAALVGGLSA LQQGLGAVGRIHEVAAMPVEDDVDLPVPETTRTAPPSVEFRDVGFAHPGRAQALRGVSFTADPGARTALVGPSGAGKSTSFALLQRFVEPDSGTILLDGV DITTIPRAHLRATIAHVEQDAPVLAGTLRENLLYAEPNATDAEVDRVLRVTMLKSFVDDLERGLDTEVGTRGVALSGGERQRLAIARALLRRPGVLLLDE ATAQLDARNETALREAVDRAALGCTVILIAHRLSTVTDAERIVVLEQGRVRASGTHAELVRGDDLYRELATTQLLAGEAGASGRADAPGEAMPAR&LINK_LOC=protein&PAGE_TYPE=BlastSearch', 'BLAST this protein','Amir_2498')" />
   IKPGDLMSRLVGDTTLLRQVTTQALVTSCTATIALVGALVMMALIDVVLLGVTLSSVVLMSASVRWSASRIGKATGQAQAAVGYMATLLDRDLGALRTVK ASGAEDHEIGLLADAAQTAFRRGLRVAAWQAGTGASAGMLMQASFLGVLGVGGARVAAGDLPIADLIAFLLYMFFLTQPVTTLVGAWGQFRAGGAAAERI QAVLRLDAEPTAARGRSSLDRRTTAGRGTNGGRGTNGGRRTTAGRRANGGHRDPATSGPMFRRGPVVVRRAALTRRAASATNTGPNRRAASATVAFDNVE FAYRQDLPPVHRGVSFTVPPGGMTAIVGPSGAGKSSIFLLLERFYDATAGRILVDGRDVRDWPLAQLRRSIGYVEQEAPVLAGSLRENLTLGLTGVDDED LHEALRLARLELFVAALPGGLDGWIGHRGGTLSGGERQRIAIARALLRRPRLLLLDEATSALDAGNELALRDTIAAAASTTTVLVVAHRLSTVVGARQII VMEAGRVRALGSHAQLLETDPLYRDLATTQLLVPTG', 'http://blast.ncbi.nlm.nih.gov/Blast.cgi?PAGE=Proteins&PROGRAM=blastp&BLAST_PROGRAMS=blastp&QUERY=MLRFTAGSRRVLLAGGVLTLLGTSMSLAQPIIAQHILARLTADQPVGRLLGVLTVAVLLGTAVSAGGFFLIESVGETLVRTVRIRLVERILRLRLSATDE IKPGDLMSRLVGDTTLLRQVTTQALVTSCTATIALVGALVMMALIDVVLLGVTLSSVVLMSASVRWSASRIGKATGQAQAAVGYMATLLDRDLGALRTVK ASGAEDHEIGLLADAAQTAFRRGLRVAAWQAGTGASAGMLMQASFLGVLGVGGARVAAGDLPIADLIAFLLYMFFLTQPVTTLVGAWGQFRAGGAAAERI QAVLRLDAEPTAARGRSSLDRRTTAGRGTNGGRGTNGGRRTTAGRRANGGHRDPATSGPMFRRGPVVVRRAALTRRAASATNTGPNRRAASATVAFDNVE FAYRQDLPPVHRGVSFTVPPGGMTAIVGPSGAGKSSIFLLLERFYDATAGRILVDGRDVRDWPLAQLRRSIGYVEQEAPVLAGSLRENLTLGLTGVDDED LHEALRLARLELFVAALPGGLDGWIGHRGGTLSGGERQRIAIARALLRRPRLLLLDEATSALDAGNELALRDTIAAAASTTTVLVVAHRLSTVVGARQII VMEAGRVRALGSHAQLLETDPLYRDLATTQLLVPTG&LINK_LOC=protein&PAGE_TYPE=BlastSearch', 'BLAST this protein','FRAAL_2909')" />
   RTVRRLLVTRILRMRPAAADRIPPADLLSRLVADTTLLRQVTTQAMVASVTAMLALLGSLVLMGLIDYVLLAVTLGAVVTMSVSVRWAAARIGAATGKAQ EAVAYLAMLLDRDLGAFQTVKAAGAEAHEIGLLTDAADTAWRRGLRVAGWQAASGASAGLLMQTSFLAVLGVGGARVAAGRLPLADLIAYLLYMFFLTQP VTTLVSAWGQLKVGGAAAERIQDVLRLPVEPEPATVGPVDGRAIAALAARARTRFGDRGHGPFPTGANGADRPRADPRRAGLCAAVAFEHVVFGYRPDLP LVHQGVTFTVPPGGTTAVVGPSGAGKSSLFALLERFHEVTSGRVLLDGRDVRDWPLAELRRAIGYVEQDSPVLAGTLRDNLTLGLSGVADERLWEVVALA RLEDLVQTLPGGLDGWIGHRGGTLSGGQRQRIAIGRAFLRRPRLLLLDEATSALDAVNEAALRTTLEAAARTTTVMVVAHRLSTVVNARQIVVLEAGRVR AVGTHSELLDADPTYRELATNQLLAASP', 'http://blast.ncbi.nlm.nih.gov/Blast.cgi?PAGE=Proteins&PROGRAM=blastp&BLAST_PROGRAMS=blastp&QUERY=MSASELAGPAPRHRPHNRLRLILWYTAGFRARLALGSVLTLAGTALSLSQPIVAQRILNRLARHQPTGGLLLLLAGAVVLGTAVGGVGYFFVESVGESLV RTVRRLLVTRILRMRPAAADRIPPADLLSRLVADTTLLRQVTTQAMVASVTAMLALLGSLVLMGLIDYVLLAVTLGAVVTMSVSVRWAAARIGAATGKAQ EAVAYLAMLLDRDLGAFQTVKAAGAEAHEIGLLTDAADTAWRRGLRVAGWQAASGASAGLLMQTSFLAVLGVGGARVAAGRLPLADLIAYLLYMFFLTQP VTTLVSAWGQLKVGGAAAERIQDVLRLPVEPEPATVGPVDGRAIAALAARARTRFGDRGHGPFPTGANGADRPRADPRRAGLCAAVAFEHVVFGYRPDLP LVHQGVTFTVPPGGTTAVVGPSGAGKSSLFALLERFHEVTSGRVLLDGRDVRDWPLAELRRAIGYVEQDSPVLAGTLRDNLTLGLSGVADERLWEVVALA RLEDLVQTLPGGLDGWIGHRGGTLSGGQRQRIAIGRAFLRRPRLLLLDEATSALDAVNEAALRTTLEAAARTTTVMVVAHRLSTVVNARQIVVLEAGRVR AVGTHSELLDADPTYRELATNQLLAASP&LINK_LOC=protein&PAGE_TYPE=BlastSearch', 'BLAST this protein','FraEuI1c_1555')" />
   QGVLLGLRTSLIGHLLRLRMRVFDTRRIGDLISRANTDTTVVREAVAYSFTALVTSVIGVVGAVALMIWLNPWLFLLVLGVVAVAGVVVLGALSRIRTVS ERGQASVGGMTADLERALAAIRTVRANRAEGREAARIGDHAAAAYQAGLRMAKLDSIIAPAMQLAVQGSVLVVLLVGGVLVARGSASLGSLVAFLLYATY LVMPLSQLIEAAATIQRGMGALSRVNAVFALPREGDDESVRGPLRTVAVVAPGGTRPPAPLSPLSSSRVSSSFSSRGETPNPRAPSIEFRDVWFGYAQTP VLRGVSFTVPSRGHVALVGPSGAGKSTILELVERFYEPDAGMILFAGHDVRAMPRATARGWVGLVEQNTPVLHGSLRDNITYAAPDATEEQIAAVLATAG LRDFTDRLPERLRTPVGDHGVLLSGGERQRVAIARALLAQPAVLLLDEPTAQLDSVNEQALTRAMSRIAAERALLVIAHRISTVRAADFILVLNEGRVVA QGSHEELMATSAFYRGLATGLPAGPASPASPAGPVSPPERGRAGTAGPPAPVR', 'http://blast.ncbi.nlm.nih.gov/Blast.cgi?PAGE=Proteins&PROGRAM=blastp&BLAST_PROGRAMS=blastp&QUERY=MTRAYVVEGEGRGAARGPASRGVTELWALLRGRRGAVAIATALTVVGTAVGVLQPLLVMRVIDAAQAGGVPGWLIGALLGLFVGQAVIDTAGHYLLERVG QGVLLGLRTSLIGHLLRLRMRVFDTRRIGDLISRANTDTTVVREAVAYSFTALVTSVIGVVGAVALMIWLNPWLFLLVLGVVAVAGVVVLGALSRIRTVS ERGQASVGGMTADLERALAAIRTVRANRAEGREAARIGDHAAAAYQAGLRMAKLDSIIAPAMQLAVQGSVLVVLLVGGVLVARGSASLGSLVAFLLYATY LVMPLSQLIEAAATIQRGMGALSRVNAVFALPREGDDESVRGPLRTVAVVAPGGTRPPAPLSPLSSSRVSSSFSSRGETPNPRAPSIEFRDVWFGYAQTP VLRGVSFTVPSRGHVALVGPSGAGKSTILELVERFYEPDAGMILFAGHDVRAMPRATARGWVGLVEQNTPVLHGSLRDNITYAAPDATEEQIAAVLATAG LRDFTDRLPERLRTPVGDHGVLLSGGERQRVAIARALLAQPAVLLLDEPTAQLDSVNEQALTRAMSRIAAERALLVIAHRISTVRAADFILVLNEGRVVA QGSHEELMATSAFYRGLATGLPAGPASPASPAGPVSPPERGRAGTAGPPAPVR&LINK_LOC=protein&PAGE_TYPE=BlastSearch', 'BLAST this protein','Francci3_2983')" />
   MAGRIVGAPVSYVESRSTADLVSRVGADSTLIQQTTVKALVDLVVVPLTVVAGIVLMLTIDVFLALVVIVLLSVAGLAEAGVFRRVVVDTETAQEHVAGL TGVVQRVLLAFRTVKASRSERREAESFDRNADSAYRASVKAVRTGALADTVAYAAVDFTFLVTLAVGVVRVSTGSVGVGDLVAILLYVVYIQEPVEALTN SAGKLSEGLAALRRITELLDAPQETDTAAAPARTEVPAVSNGSKPPASRRSVRFDQVWFGYGDQPVLRDVSIEAVPGLTVLVGSSGAGKTTLLSLAERFV EPERGAVLLDGADVRDLPLAELRGRVAYVQQEAPLLGATIGEAATYGVDDVDEERLRRILESVGLRTWIEGLPLGLDTEVGERGVQISGGQRQRLAVARA LARDSEVLLLDEATSQLDPLNEQNLVRSLTRDFRDRVVIAVTHRMPMAYQADQVIMLRHGSVHARGTHDDLLSDPHYRQLIASPSAGSPELAEQLTASGE DGRGEPGA', 'http://blast.ncbi.nlm.nih.gov/Blast.cgi?PAGE=Proteins&PROGRAM=blastp&BLAST_PROGRAMS=blastp&QUERY=MEPDAASRPTSSLRTLISRLAPFRGALTVVLLLELGTRAAALIQPLAARAVVDGVAARADLMWPIAVLGAVAIIGLCLNYAGFYQRGKLSERFVLGLRKA MAGRIVGAPVSYVESRSTADLVSRVGADSTLIQQTTVKALVDLVVVPLTVVAGIVLMLTIDVFLALVVIVLLSVAGLAEAGVFRRVVVDTETAQEHVAGL TGVVQRVLLAFRTVKASRSERREAESFDRNADSAYRASVKAVRTGALADTVAYAAVDFTFLVTLAVGVVRVSTGSVGVGDLVAILLYVVYIQEPVEALTN SAGKLSEGLAALRRITELLDAPQETDTAAAPARTEVPAVSNGSKPPASRRSVRFDQVWFGYGDQPVLRDVSIEAVPGLTVLVGSSGAGKTTLLSLAERFV EPERGAVLLDGADVRDLPLAELRGRVAYVQQEAPLLGATIGEAATYGVDDVDEERLRRILESVGLRTWIEGLPLGLDTEVGERGVQISGGQRQRLAVARA LARDSEVLLLDEATSQLDPLNEQNLVRSLTRDFRDRVVIAVTHRMPMAYQADQVIMLRHGSVHARGTHDDLLSDPHYRQLIASPSAGSPELAEQLTASGE DGRGEPGA&LINK_LOC=protein&PAGE_TYPE=BlastSearch', 'BLAST this protein','SACE_0601')" />
   GLVHRLIRLRVAELDRRAPGDLIARVTSDSTLLKSAATEGLIMTVNGVLTFAGALFMMAALDARLLGVTLLVLTLVGVVITVILPRIKAAVARSQSSVGA VGAVLDRTLGAARTVKANGAEGRETLAAEDAVDEAYAAGLVGARYSALVTMVGGAAIQTAFLVVLGVGGTFVANRTMSVSELIAFLLYVFFLASPVSQLV GGAAQLQQGLGAVGRIQEAAGLPVEDDIDAPPAAAPAHAAPPAVELTGVEFAYPGRAPALRGVGFTVPGGTQTALVGLSGAGKTTLFSLLQRFYEPTAGT IRIGGQDISALPRAEVRRRIAYVEQDSPVMAGSLRENLLYAAPSATPEELAEALAVTRLDALVARLPQGLDTPVGPRGVTLSGGERQRLAIARALLRRPQ VLLLDEATAQLDARNEQALSELVARAAGRCTVLLIAHRLSTVTDADQIVVLEHGGVRAVGTHHSLVDDDDLYRELAATQLLAAEPPA', 'http://blast.ncbi.nlm.nih.gov/Blast.cgi?PAGE=Proteins&PROGRAM=blastp&BLAST_PROGRAMS=blastp&QUERY=MTDDPTPTTPPGSLSALIGYARPHWRVLLLSLVLTLLASVSGLVQPKFAQAILDRLDDGAGVVAPVALLAVFLVAGALLTGLNAWLQQRTSERVVRQVRR GLVHRLIRLRVAELDRRAPGDLIARVTSDSTLLKSAATEGLIMTVNGVLTFAGALFMMAALDARLLGVTLLVLTLVGVVITVILPRIKAAVARSQSSVGA VGAVLDRTLGAARTVKANGAEGRETLAAEDAVDEAYAAGLVGARYSALVTMVGGAAIQTAFLVVLGVGGTFVANRTMSVSELIAFLLYVFFLASPVSQLV GGAAQLQQGLGAVGRIQEAAGLPVEDDIDAPPAAAPAHAAPPAVELTGVEFAYPGRAPALRGVGFTVPGGTQTALVGLSGAGKTTLFSLLQRFYEPTAGT IRIGGQDISALPRAEVRRRIAYVEQDSPVMAGSLRENLLYAAPSATPEELAEALAVTRLDALVARLPQGLDTPVGPRGVTLSGGERQRLAIARALLRRPQ VLLLDEATAQLDARNEQALSELVARAAGRCTVLLIAHRLSTVTDADQIVVLEHGGVRAVGTHHSLVDDDDLYRELAATQLLAAEPPA&LINK_LOC=protein&PAGE_TYPE=BlastSearch', 'BLAST this protein','SAV_7357')" />
   RLRVSEVDRLKPGDLLSRVTSDTTLLRAVFTDGVVETVSAVFMLVGAVVMMAIMDGLLLLITLTVLILVGSLVGLVMPRIRRASTQAQVAVGEMGAVLDR VLQAFRTVKASGAEDREIATVGAAAREARDRGVAVAWWTSIAGISAWVSAQLAFVAVLGVGGARVASGALEVSSLIAFLLYLFYLVAPIGQMVQGVTQMQ NGLAAVKRIREIEELPAEEAADAAGTVGTAPAGVSFEGVAFRYGDDRPVVHHDVSFTVPAGGMTALVGPSGAGKSTVFALLERFYEQQSGTISVDGRDIG QWPLGRLRASLGYVEQDAPVLDGSLRENLVFAAPEVGEAEIRRVLALTRLEDLVARLPEGLETKVGHRGIMLSGGERQRVAIARALLRRPRLLLLDEATS QLDAVNELRLREVIAEVARETTVLVIAHRLSTVTTADRIVVMEAGRVRAVGTHSELLDGDDLYRELAATQFLLPT', 'http://blast.ncbi.nlm.nih.gov/Blast.cgi?PAGE=Proteins&PROGRAM=blastp&BLAST_PROGRAMS=blastp&QUERY=MPETASWRLLFAYVRPHRRALLLGGVLSLVGSLAGLAMPLLAKVVIDAFGEQRSLVGPVLGLTAAVLLGAAVGALGRYVLERMGEGVVFSARRSLVDRML RLRVSEVDRLKPGDLLSRVTSDTTLLRAVFTDGVVETVSAVFMLVGAVVMMAIMDGLLLLITLTVLILVGSLVGLVMPRIRRASTQAQVAVGEMGAVLDR VLQAFRTVKASGAEDREIATVGAAAREARDRGVAVAWWTSIAGISAWVSAQLAFVAVLGVGGARVASGALEVSSLIAFLLYLFYLVAPIGQMVQGVTQMQ NGLAAVKRIREIEELPAEEAADAAGTVGTAPAGVSFEGVAFRYGDDRPVVHHDVSFTVPAGGMTALVGPSGAGKSTVFALLERFYEQQSGTISVDGRDIG QWPLGRLRASLGYVEQDAPVLDGSLRENLVFAAPEVGEAEIRRVLALTRLEDLVARLPEGLETKVGHRGIMLSGGERQRVAIARALLRRPRLLLLDEATS QLDAVNELRLREVIAEVARETTVLVIAHRLSTVTTADRIVVMEAGRVRAVGTHSELLDGDDLYRELAATQFLLPT&LINK_LOC=protein&PAGE_TYPE=BlastSearch', 'BLAST this protein','Sros_1772')" />
   LLLMGVLVVVTAGIGPLGEYVLRRTGESIVLTARRRLVSHLLRLRMSAVDQVEPGDLMSRVTSDTTLLRQVTTDSLVGTVTGGLTILATMTMMGVLDPVL LAVTLGVLLLAGTVVRVVMPRVSRASRDIQKSVGAMGAALERILGALRTVKASGAEHREEAAIHGAAEESWQASVRAAKWLAVAGHTAELALQVAFFTVL AAGGARVASGLIDVGTLIAFLMYVFTLVPSLQGLVGAAGQYQIGAAAVARIQEAERLPSEPQRPAAVLPAPGAAPAELRFRDVRFRYAPDLPPVHRGVTF TVPSRGMTAFVGPSGAGKTTLFSLIERFYEPTSGSVLLDGRDLGDWDLPELRAAIGYVEQDAPVLSGSLRDNLLLGAPGATGEEIRRVLRTARLDDLVAR LPKGLDTLVGHRGTKLSGGERQRVAIARALLRRPRLLLLDEATSQLDAVNEAALRDTVAEAARTTTVLVVAHRLSTVTLADRIVVIEAGTVRAVGTHDEL VVADPLYAELAATQFLTATG', 'http://blast.ncbi.nlm.nih.gov/Blast.cgi?PAGE=Proteins&PROGRAM=blastp&BLAST_PROGRAMS=blastp&QUERY=MDTQRLQPDAPAAVAGAAEPRVSRPGRLPPRDGGTSAPVPAPSDPSPAWRVLLRYMRPSWRWLALGGLLNLVSGAIGLALPLAAKQLVDDLGADRPLTDV LLLMGVLVVVTAGIGPLGEYVLRRTGESIVLTARRRLVSHLLRLRMSAVDQVEPGDLMSRVTSDTTLLRQVTTDSLVGTVTGGLTILATMTMMGVLDPVL LAVTLGVLLLAGTVVRVVMPRVSRASRDIQKSVGAMGAALERILGALRTVKASGAEHREEAAIHGAAEESWQASVRAAKWLAVAGHTAELALQVAFFTVL AAGGARVASGLIDVGTLIAFLMYVFTLVPSLQGLVGAAGQYQIGAAAVARIQEAERLPSEPQRPAAVLPAPGAAPAELRFRDVRFRYAPDLPPVHRGVTF TVPSRGMTAFVGPSGAGKTTLFSLIERFYEPTSGSVLLDGRDLGDWDLPELRAAIGYVEQDAPVLSGSLRDNLLLGAPGATGEEIRRVLRTARLDDLVAR LPKGLDTLVGHRGTKLSGGERQRVAIARALLRRPRLLLLDEATSQLDAVNEAALRDTVAEAARTTTVLVVAHRLSTVTLADRIVVIEAGTVRAVGTHDEL VVADPLYAELAATQFLTATG&LINK_LOC=protein&PAGE_TYPE=BlastSearch', 'BLAST this protein','Sros_7440')" />
   QQRTSEQVVRSIRRDLVFRLIRLKIPELDRRPPGDLVARVTSDSALVQNAATQGLVMITNGLLTVVGAIVLMGTVHLGLLGVTLAVLLGTGALLGLILPR IREAVARAQSSVGAIGAALDRSLGAARTVKANGAETRETARAEAAVQEAYQAGLTGAKYVALVQVLSGVAIQAAFLAVLGVGGALVASGDLDASQLIAFL LYVFYLASPIASLLGGLGMLQQGLGAIVRIEQVKHLPAEDDVDLPERAAPHTARSTPAPPVEFDGVHFAYPGRGPALRGISFTVPGATRTALVGLSGAGK TTMFALLQRFYEPRAGAIRIGGQDIALMSRAEVRQRIAYVEQESPVMAGTLGDNLRYAATGAGDEDIAEVLRLTRLDSLIARLPDGLDTEVGNRGVTLSG GERQRLAIARALLRRPEVLLLDEATAQLDASNEQALREAVDQAARRCTVILIAHRLSTVTDAEQIIVLEDGGIRAAGSHDDLVGADTLYRELAASQMLVP AVDDRPPGPDAQLTL', 'http://blast.ncbi.nlm.nih.gov/Blast.cgi?PAGE=Proteins&PROGRAM=blastp&BLAST_PROGRAMS=blastp&QUERY=MTTAHPMPPPQAPSPASDEPPAPGIRPLRALLQQARPYRFVLLGSLLLALAASGCGLVQPLVARSVLDALADDTVVTGSLIVLSVLVVAGAVLTGLQSWW QQRTSEQVVRSIRRDLVFRLIRLKIPELDRRPPGDLVARVTSDSALVQNAATQGLVMITNGLLTVVGAIVLMGTVHLGLLGVTLAVLLGTGALLGLILPR IREAVARAQSSVGAIGAALDRSLGAARTVKANGAETRETARAEAAVQEAYQAGLTGAKYVALVQVLSGVAIQAAFLAVLGVGGALVASGDLDASQLIAFL LYVFYLASPIASLLGGLGMLQQGLGAIVRIEQVKHLPAEDDVDLPERAAPHTARSTPAPPVEFDGVHFAYPGRGPALRGISFTVPGATRTALVGLSGAGK TTMFALLQRFYEPRAGAIRIGGQDIALMSRAEVRQRIAYVEQESPVMAGTLGDNLRYAATGAGDEDIAEVLRLTRLDSLIARLPDGLDTEVGNRGVTLSG GERQRLAIARALLRRPEVLLLDEATAQLDASNEQALREAVDQAARRCTVILIAHRLSTVTDAEQIIVLEDGGIRAAGSHDDLVGADTLYRELAASQMLVP AVDDRPPGPDAQLTL&LINK_LOC=protein&PAGE_TYPE=BlastSearch', 'BLAST this protein','Strvi_6296')" />

 
 
   ARIEYDLRNIVYEHLTAMSFPFYDRMQSGQLISRANSDIRAVQMYLAFAPYLLVQSGISLVSFAYMLAIDVPLAILAMLPMPLMFVASRRMQRSLFPVSW LIQARLADVATVVDENVNGVRVVKSFAAERSQLASLQKAATSVAWANVKDADLRARWTPLVQNLPRVGMAIVLLYGGYLVIHGELGVGAILAFNAYLLML QVPFQVIGNLIMLGQRSSAAAKRLYEVLDEKPEIADAPDAREITGPRGDVRFDDVTFGYGDGLDVLKGFSLQLRPGETVALVGRTGTGKSTAARLLPRFY DVRAGSVSVDGHDVRDLTLRSLRDTVGVVLDEPFLFSASVRDNIAYGRPDADFTDIERVARLAGAHEFITELSEGYDTVIGERGYTLSGGQRQRIAIART LLLNPPVLVLDDATSAIDVQVEQEIHAGLRSLFAGRTTLIVAHRLSTISLADRVVLLDGGRIVADGTHKELLASTPLYAEVLAQADETIEELGSVGR', 'http://blast.ncbi.nlm.nih.gov/Blast.cgi?PAGE=Proteins&PROGRAM=blastp&BLAST_PROGRAMS=blastp&QUERY=MNKTYPPPSAAIDPDPSRSWLWRAWPLVRAHRWMFSAALVMSGASMLVVVQVPRLIQDAVDNSIVSDRVPLSHYMWWLVGMSVIMLVVGYLSKQLLFRAA ARIEYDLRNIVYEHLTAMSFPFYDRMQSGQLISRANSDIRAVQMYLAFAPYLLVQSGISLVSFAYMLAIDVPLAILAMLPMPLMFVASRRMQRSLFPVSW LIQARLADVATVVDENVNGVRVVKSFAAERSQLASLQKAATSVAWANVKDADLRARWTPLVQNLPRVGMAIVLLYGGYLVIHGELGVGAILAFNAYLLML QVPFQVIGNLIMLGQRSSAAAKRLYEVLDEKPEIADAPDAREITGPRGDVRFDDVTFGYGDGLDVLKGFSLQLRPGETVALVGRTGTGKSTAARLLPRFY DVRAGSVSVDGHDVRDLTLRSLRDTVGVVLDEPFLFSASVRDNIAYGRPDADFTDIERVARLAGAHEFITELSEGYDTVIGERGYTLSGGQRQRIAIART LLLNPPVLVLDDATSAIDVQVEQEIHAGLRSLFAGRTTLIVAHRLSTISLADRVVLLDGGRIVADGTHKELLASTPLYAEVLAQADETIEELGSVGR&LINK_LOC=protein&PAGE_TYPE=BlastSearch', 'BLAST this protein','SAV_2365')" />

 
 
   NSDPVQVRYFGFEGTMSLGVAMMFAALAGALTAGLLGTVRILQLRARARRATGSR', 'http://blast.ncbi.nlm.nih.gov/Blast.cgi?PAGE=Proteins&PROGRAM=blastp&BLAST_PROGRAMS=blastp&QUERY=MSTPSQYDPARPNTPYDMTTAPTPPSGLPAIQPTPAADPSYPPDPSGPSFGGSDAVDPAAGAPRATPNSAAKKHSRTGAAWVALVVAAIVMIFLLIFILQ NSDPVQVRYFGFEGTMSLGVAMMFAALAGALTAGLLGTVRILQLRARARRATGSR&LINK_LOC=protein&PAGE_TYPE=BlastSearch', 'BLAST this protein','Namu_5112')" />

 
 
   EGEVPGLDEAGFVKAAEDAKANCPVSQALSGTTITLTASLVA', 'http://blast.ncbi.nlm.nih.gov/Blast.cgi?PAGE=Proteins&PROGRAM=blastp&BLAST_PROGRAMS=blastp&QUERY=MATTRQAHTVWEGNLLEGSGVVSLDSSGIGEYPVSWPSRAEQANGRTSPEELIAAAHSSCFSMALSNGLASAGNPPARLDTKAEVTFQPGTGITGIHLTV EGEVPGLDEAGFVKAAEDAKANCPVSQALSGTTITLTASLVA&LINK_LOC=protein&PAGE_TYPE=BlastSearch', 'BLAST this protein','SACTE_5283')" />
   TLRASVDGIDEERFSTIAQKAKDTCPVSRALAGTTITLHASLA', 'http://blast.ncbi.nlm.nih.gov/Blast.cgi?PAGE=Proteins&PROGRAM=blastp&BLAST_PROGRAMS=blastp&QUERY=MATRDATTHWTGGLNSGTGTLTLDSSNSARFTVSFPRRIGEPEGTTSPEELIAAAQASCLAMNLAGTLEKEGLTANSIDANASVTVEPSNGGLSINNVDV TLRASVDGIDEERFSTIAQKAKDTCPVSRALAGTTITLHASLA&LINK_LOC=protein&PAGE_TYPE=BlastSearch', 'BLAST this protein','Svir_37030')" />

 
 
   EEPTPAGYRQAVGLARRLAFVTADLIEVLIEARYVKEIE', 'http://blast.ncbi.nlm.nih.gov/Blast.cgi?PAGE=Proteins&PROGRAM=blastp&BLAST_PROGRAMS=blastp&QUERY=MSTAVAPKPPAQEWTPPLDADGLRLVLERFRAWEPLDIEEVFDDLDAAIGSQPPPVASAVALLGRLRRHLKQLSDITVADDSFPPSAEMTRLVERGVPLL EEPTPAGYRQAVGLARRLAFVTADLIEVLIEARYVKEIE&LINK_LOC=protein&PAGE_TYPE=BlastSearch', 'BLAST this protein','SGR_3850')" />

 
 
   RRTEDIGHEDFARTVAINLTGLFLGLKHVLKVMREQGHGRVLNTSSLFGIRANGLGSDYHASKHGVVGLTRNAGVEYGRYGITVNAMAPGTILTPMVENH LGALRQQAPEAIDKALDRIPAGRFGHPDEVAALAAFLLSDEASYINATVVTIDGGRSERA', 'http://blast.ncbi.nlm.nih.gov/Blast.cgi?PAGE=Proteins&PROGRAM=blastp&BLAST_PROGRAMS=blastp&QUERY=MQDRFTDKVALITGGGSGLGRATAVRLASEGARLVLVDINEQGLADSVVELGKAAPGTEVRTVVADVSQEADVARYVARSVEHHGRIDCFFNNAGVEPTQ RRTEDIGHEDFARTVAINLTGLFLGLKHVLKVMREQGHGRVLNTSSLFGIRANGLGSDYHASKHGVVGLTRNAGVEYGRYGITVNAMAPGTILTPMVENH LGALRQQAPEAIDKALDRIPAGRFGHPDEVAALAAFLLSDEASYINATVVTIDGGRSERA&LINK_LOC=protein&PAGE_TYPE=BlastSearch', 'BLAST this protein','SCAB_79721')" />

 
 
   RRWTDHAGEGLLGLALAIAVLLLRRWRIRAGSGN', 'http://blast.ncbi.nlm.nih.gov/Blast.cgi?PAGE=Proteins&PROGRAM=blastp&BLAST_PROGRAMS=blastp&QUERY=MGHVHPSHLVELALGHASGEADVGALRHAASCPRCREELLRLTRVVTAARGAEASDLPVPPPERVWQRIALEVLPETDRVPRLRESSAHGSADERVRGSQ RRWTDHAGEGLLGLALAIAVLLLRRWRIRAGSGN&LINK_LOC=protein&PAGE_TYPE=BlastSearch', 'BLAST this protein','SCO_0196')" />

 
 
   VLVDALSSRWSWYRPRSGGKVVWAVLPAEVQPPVQPSADTVRLERRSARPVPEPVRPVVLQDDPTLLRRVADRLRALDDWHLPVPRAAAWDRHLPSAEGG RPGRADRENAR', 'http://blast.ncbi.nlm.nih.gov/Blast.cgi?PAGE=Proteins&PROGRAM=blastp&BLAST_PROGRAMS=blastp&QUERY=MTVRALVEHFPATLAAVPAARAATTRALRQWHLPAVDLDAAELVVAELMANAAKASPDGETIALRVAVLSEGLLVEVWDSADALPILRAPAADGEGGRGL VLVDALSSRWSWYRPRSGGKVVWAVLPAEVQPPVQPSADTVRLERRSARPVPEPVRPVVLQDDPTLLRRVADRLRALDDWHLPVPRAAAWDRHLPSAEGG RPGRADRENAR&LINK_LOC=protein&PAGE_TYPE=BlastSearch', 'BLAST this protein','Franean1_1327')" />

 
 
   EFKNGVTIKAYFVDKDGAVSPTYTTTSKNLAGQDGRYSILMKPWTDAKGKVHTFEAIPGEQLRVWAVAPNGYTISHTESYPIGTSTKRDNAAWNLASGAN HVYNWSISLQELPTDWQWREPATRTDSSSKIQDDGGWVRGTIYWDQAHTWGATGYPQYNPGLGDVPAPNVEVVGSYVNDEVARRFDAWLGKNPKASQKDF QAAQQQILSDYQKETGQSGIAETVRGFTDANGNYLLQFNGIYGNSYSNAGISADKSLYGKVANSPTDGTWYQSQARSKHINAKYMYVFPTSATTQNVTMA SMQVPMFQNITDTDLTDSTGPVGAAGVLFNYSNVNFPMRPGTPKFDVVNYDNSNNPARPGDTAYTSATGLVPNTPYTVKWWDEQGNVVKEEKLITDGIGN LPKSTYTVDPKLATSQVYTASLLDKAGQEFAADSFIALPFKAVTPVGSVGDAYSGSIAQSIPAGAKVNYAASNLPPGLTLDPATGKITGTPTTAGTWDVA VVSTITTADGQTLKLNNTVPISITDVPLVNGVVGDAYVQKVEPTGLPEGSTYTLKSAENLPAGLSFNPATGTITGTPTVAGTADNVKLTYDITLPDGTVV KNHVDDVSLKIAPPIAPAQRFNATNEPDYQDTAVVQGAQILIPLPINKDGSAVPTGSQFVPGPNFPAWATLNADGSISAAPGTDVPAGPVTMQVVVVYPD GSNDLIDVVVDVKQAPPKAAPLAETNDPSYQPTTVVAGSAASVPAPLNSNGSLPPTGSTFTAGPDAPDWVKVNSDGSLDFTPPKGTAAGEYTVPVVVTYP DGSKETITTTINVTAPAPARQATTNEPVYDPITVLPGTTAEVPSPLNFDGTAPPAGTKFTGGNADGAKVPDWIQVNPDGSLTVKPGSNVKPGDYAVPITV TYPDGSTESIAATVTVSPTPPAPSVPQNRVYDPKYQDNSVVQGAEQNIPAPVDSTGLALPTGTKFALDAPAPGWVTIGEDGSLTAKPGTDVAPGDYPVSV VVTYPDGTTERVTTTIKVTPAPQPPAAPNDQTFNPVYADSTVLQGDTATLPAPLDNGQPLPEGTKFAPGADVPAWATVNPDGTITVNPTDSTPTGDTTIK VLVTYPDGTSETVTAAVKVAEVPAPPAPEGKLVDAYQPTYTPESVVQGVPHEVPAPLNTDGAAMPAGTKFTAAGDTPAWVTVNEDGSLQLKADENVAPGE YSVPIQVTYTDGTSEVVNVKVTVSAPSVTPDPEVPDNEANAPTYPDVSIQQGAERTIPTPLNADETSMPSGTTFEQTGDSYPWVTVNSDGTVSLAPGADV TPGDYPVTVKVTYPDGTTDTVTMKLSVTAKPADQTTDPTTNSVDAKYQQDNAVKAGDSSVVPVPTDANNSPLPAGTTFEGALLPDWAKVNSDGSISLNPP ADVTTSDVVLHVRAIYPDGSRDVVTVPVHVEGVQTPPALTQSSSYQPVYPTDTTAHAGSNDPVNIPAPQWADGKAPDGAKFALADGAPSWMTVNPDGSIT VKPGADVPAGDYLVPVVVTYPDGTNERINVPVAVTAPSKQQTYTPVYTQDTKVEAGKTVDIAAPSWENDLKPTQPVSFAATTGAPAGVTVNQDGSITFAA DEKLAAGTYVIPVEVTYADGSQEVVTVPVVVSAAPGTSVPPVVVDASTYQPTYGAPTTVKAGETATVSAPNWGDNKPNGDVKFTAGDTSPSWAAVNPDGS IDVKPGDNVPAGTYIVPVVVTYPDGTTEIVNVPVIVEAPAVPSKTNPELYQPVYPTNNSVEAGKELSVSPKWENDKSPAGPTTFSPGVAYPSWATVTPEG TVIANPPESVAPGTYYMPVVVTYPDGLKDYISVPIEVTNSGTTTPSVQTDNTRYHASYPTTTVNAGGEPVTTAPVWENRTVPGGGTTFTKGQNAPEWATV NPDGSITIQPGADVPPGNYVVPVVVKYPDGSEDIVYAPVSVGNPTVPSTTHDVDVYQPVYVTDTTVKPNDVHDIPAPKEASGAPFPAGTTFALGDQDPFS AKSINPQTGEIHMEVPSWKKPGTYYIPVVVTYPDGTTETVNVPFVVESTAAPEDPSASTAVPVYGSIQASPGKQITTLPPVFTDNGKTVDMPAGTTFTPG PGAPTDMKIGTDGAVTVTVPKDAQPGTLIRVPVIATVPGADPHTAWVEINVTPAPLNGVSPIWTGGGAAAGATVTVPNTGGPVQPGTTISTEGPGTATLN PNGSITVSVDENATPGSVIIVTVKDAEGKVIDTVQITVTSKPAETSTPGTTTPGTDAPGTTTPGTTAPGTTTPGTDAPGTTTPGTTAPGTTTPGTDAPGT TTPGTTTPGTDAPGTTAPGTTTPGTTAPGTTTPGTTAPGTTTPGTTAPGTTTPGTTTPGTTAPGTTTPGTDTPGTTTPGTETPSPEQPGNPDGSSIDPLD KGIIAGIIGAIAGGALGSSTPMGPRPGTTLPGSATPSQPGKPGKPGKPGTSKPNEPGKQGSSQPGKSNGSGKPGKPGTPGDSGANVTRPAQPSTGSNPQS PITNGVSGGNQGGREGIDANGSANNSSRSGQLAMTGLSGLAITAGIALAALLAGGALMLLRRRREED', 'http://blast.ncbi.nlm.nih.gov/Blast.cgi?PAGE=Proteins&PROGRAM=blastp&BLAST_PROGRAMS=blastp&QUERY=MPSTRSRHFRHRNHVAPAVRRFIFKDRMRAVLAAGAIVAVITSGTVVVQSFEPHDSHSTQISNTAVNEIASDGTWQGQYTVNGQIFVDRGGLQNFYNSGD EFKNGVTIKAYFVDKDGAVSPTYTTTSKNLAGQDGRYSILMKPWTDAKGKVHTFEAIPGEQLRVWAVAPNGYTISHTESYPIGTSTKRDNAAWNLASGAN HVYNWSISLQELPTDWQWREPATRTDSSSKIQDDGGWVRGTIYWDQAHTWGATGYPQYNPGLGDVPAPNVEVVGSYVNDEVARRFDAWLGKNPKASQKDF QAAQQQILSDYQKETGQSGIAETVRGFTDANGNYLLQFNGIYGNSYSNAGISADKSLYGKVANSPTDGTWYQSQARSKHINAKYMYVFPTSATTQNVTMA SMQVPMFQNITDTDLTDSTGPVGAAGVLFNYSNVNFPMRPGTPKFDVVNYDNSNNPARPGDTAYTSATGLVPNTPYTVKWWDEQGNVVKEEKLITDGIGN LPKSTYTVDPKLATSQVYTASLLDKAGQEFAADSFIALPFKAVTPVGSVGDAYSGSIAQSIPAGAKVNYAASNLPPGLTLDPATGKITGTPTTAGTWDVA VVSTITTADGQTLKLNNTVPISITDVPLVNGVVGDAYVQKVEPTGLPEGSTYTLKSAENLPAGLSFNPATGTITGTPTVAGTADNVKLTYDITLPDGTVV KNHVDDVSLKIAPPIAPAQRFNATNEPDYQDTAVVQGAQILIPLPINKDGSAVPTGSQFVPGPNFPAWATLNADGSISAAPGTDVPAGPVTMQVVVVYPD GSNDLIDVVVDVKQAPPKAAPLAETNDPSYQPTTVVAGSAASVPAPLNSNGSLPPTGSTFTAGPDAPDWVKVNSDGSLDFTPPKGTAAGEYTVPVVVTYP DGSKETITTTINVTAPAPARQATTNEPVYDPITVLPGTTAEVPSPLNFDGTAPPAGTKFTGGNADGAKVPDWIQVNPDGSLTVKPGSNVKPGDYAVPITV TYPDGSTESIAATVTVSPTPPAPSVPQNRVYDPKYQDNSVVQGAEQNIPAPVDSTGLALPTGTKFALDAPAPGWVTIGEDGSLTAKPGTDVAPGDYPVSV VVTYPDGTTERVTTTIKVTPAPQPPAAPNDQTFNPVYADSTVLQGDTATLPAPLDNGQPLPEGTKFAPGADVPAWATVNPDGTITVNPTDSTPTGDTTIK VLVTYPDGTSETVTAAVKVAEVPAPPAPEGKLVDAYQPTYTPESVVQGVPHEVPAPLNTDGAAMPAGTKFTAAGDTPAWVTVNEDGSLQLKADENVAPGE YSVPIQVTYTDGTSEVVNVKVTVSAPSVTPDPEVPDNEANAPTYPDVSIQQGAERTIPTPLNADETSMPSGTTFEQTGDSYPWVTVNSDGTVSLAPGADV TPGDYPVTVKVTYPDGTTDTVTMKLSVTAKPADQTTDPTTNSVDAKYQQDNAVKAGDSSVVPVPTDANNSPLPAGTTFEGALLPDWAKVNSDGSISLNPP ADVTTSDVVLHVRAIYPDGSRDVVTVPVHVEGVQTPPALTQSSSYQPVYPTDTTAHAGSNDPVNIPAPQWADGKAPDGAKFALADGAPSWMTVNPDGSIT VKPGADVPAGDYLVPVVVTYPDGTNERINVPVAVTAPSKQQTYTPVYTQDTKVEAGKTVDIAAPSWENDLKPTQPVSFAATTGAPAGVTVNQDGSITFAA DEKLAAGTYVIPVEVTYADGSQEVVTVPVVVSAAPGTSVPPVVVDASTYQPTYGAPTTVKAGETATVSAPNWGDNKPNGDVKFTAGDTSPSWAAVNPDGS IDVKPGDNVPAGTYIVPVVVTYPDGTTEIVNVPVIVEAPAVPSKTNPELYQPVYPTNNSVEAGKELSVSPKWENDKSPAGPTTFSPGVAYPSWATVTPEG TVIANPPESVAPGTYYMPVVVTYPDGLKDYISVPIEVTNSGTTTPSVQTDNTRYHASYPTTTVNAGGEPVTTAPVWENRTVPGGGTTFTKGQNAPEWATV NPDGSITIQPGADVPPGNYVVPVVVKYPDGSEDIVYAPVSVGNPTVPSTTHDVDVYQPVYVTDTTVKPNDVHDIPAPKEASGAPFPAGTTFALGDQDPFS AKSINPQTGEIHMEVPSWKKPGTYYIPVVVTYPDGTTETVNVPFVVESTAAPEDPSASTAVPVYGSIQASPGKQITTLPPVFTDNGKTVDMPAGTTFTPG PGAPTDMKIGTDGAVTVTVPKDAQPGTLIRVPVIATVPGADPHTAWVEINVTPAPLNGVSPIWTGGGAAAGATVTVPNTGGPVQPGTTISTEGPGTATLN PNGSITVSVDENATPGSVIIVTVKDAEGKVIDTVQITVTSKPAETSTPGTTTPGTDAPGTTTPGTTAPGTTTPGTDAPGTTTPGTTAPGTTTPGTDAPGT TTPGTTTPGTDAPGTTAPGTTTPGTTAPGTTTPGTTAPGTTTPGTTAPGTTTPGTTTPGTTAPGTTTPGTDTPGTTTPGTETPSPEQPGNPDGSSIDPLD KGIIAGIIGAIAGGALGSSTPMGPRPGTTLPGSATPSQPGKPGKPGKPGTSKPNEPGKQGSSQPGKSNGSGKPGKPGTPGDSGANVTRPAQPSTGSNPQS PITNGVSGGNQGGREGIDANGSANNSSRSGQLAMTGLSGLAITAGIALAALLAGGALMLLRRRREED&LINK_LOC=protein&PAGE_TYPE=BlastSearch', 'BLAST this protein','CRES_0606')" />

 
 
   RQFEGGFPPVLLRYYADMAAKQEDEIRPGMLGGSTLVTRSPIGVVGAIVPWNVPQAITFLKLAPALAAGCTVVLKPSPETVLDAFLMAEAAIAAGLPAGV LNIVPGGRELGAYLVAHPGIDKVSFTGSTGAGRAIARTCGELLRPVTLELGGKSAAVILDDADLAANLESFYAATLLNNGQICWLGTRVLAPRSRYTEIV DTITDLARSLAVGDPLSDTTQMGPLVSARQRHRVESYIDKGRSDGGRVTTGGRRPDNIDHGWFVEPTVFADVETGHTIAQEEIFGPVLSVIPYTDEAEAI AIANHSDYGLGGSIWTADPDRGAAFARNVASGTVGINGYSNDPTAPFGGIKDSGLGRELGPEGLASYQQLKSIYLDVR', 'http://blast.ncbi.nlm.nih.gov/Blast.cgi?PAGE=Proteins&PROGRAM=blastp&BLAST_PROGRAMS=blastp&QUERY=MTIDYTDLYIRGGWAKPSGASTIPVISPTTEQQIGSVPEGAETDIDDAVDAARAALTEWAAWSAAQRAEVLGRFADELEARAEDTSTRVSSQNGMPITLA RQFEGGFPPVLLRYYADMAAKQEDEIRPGMLGGSTLVTRSPIGVVGAIVPWNVPQAITFLKLAPALAAGCTVVLKPSPETVLDAFLMAEAAIAAGLPAGV LNIVPGGRELGAYLVAHPGIDKVSFTGSTGAGRAIARTCGELLRPVTLELGGKSAAVILDDADLAANLESFYAATLLNNGQICWLGTRVLAPRSRYTEIV DTITDLARSLAVGDPLSDTTQMGPLVSARQRHRVESYIDKGRSDGGRVTTGGRRPDNIDHGWFVEPTVFADVETGHTIAQEEIFGPVLSVIPYTDEAEAI AIANHSDYGLGGSIWTADPDRGAAFARNVASGTVGINGYSNDPTAPFGGIKDSGLGRELGPEGLASYQQLKSIYLDVR&LINK_LOC=protein&PAGE_TYPE=BlastSearch', 'BLAST this protein','ROP_45660')" />
   YSWSVLAQALGAMMVWDAAITVARDFGYEERRAGVLGPLLVRREPVGVVAAVVPWNVPQFVAAAKLAPALLAGCSVVLKPSPETPLDSYILAEIAAEAGL PEGALSILPADREISEYLVGHPGVDKVSFTGSVAAGKRVMEVASRHLTRVTLELGGKSAAVILPDADLEAAVAGIVPNAWMNNGQACVAQTRILAPRSHY DEIAERFAAAASALVVGDPLDPATQVGPLVARRQQKRSLDYIALGQREGAKVLTGGGRPKALDTGWYVEPTLFGDVGNGMRIAREEIFGPVICLLPYGDE EEAARIADDSDYGLSGSVWTADVGHGIDFARRIRTGTYSVNTFSLDMLGPFGGYKNSGLGREFGPEGFSAYLEHKMIHLPQGWDGETH', 'http://blast.ncbi.nlm.nih.gov/Blast.cgi?PAGE=Proteins&PROGRAM=blastp&BLAST_PROGRAMS=blastp&QUERY=MGDLIEHGQLFIGGTLTDPAGKDTIEVVSPHTEEVIGRVPHAARGDVDRAVAAARTAFDEGPWPRMSVAERIAVVTRIKDAIAVRHEEIARVISAQNGSP YSWSVLAQALGAMMVWDAAITVARDFGYEERRAGVLGPLLVRREPVGVVAAVVPWNVPQFVAAAKLAPALLAGCSVVLKPSPETPLDSYILAEIAAEAGL PEGALSILPADREISEYLVGHPGVDKVSFTGSVAAGKRVMEVASRHLTRVTLELGGKSAAVILPDADLEAAVAGIVPNAWMNNGQACVAQTRILAPRSHY DEIAERFAAAASALVVGDPLDPATQVGPLVARRQQKRSLDYIALGQREGAKVLTGGGRPKALDTGWYVEPTLFGDVGNGMRIAREEIFGPVICLLPYGDE EEAARIADDSDYGLSGSVWTADVGHGIDFARRIRTGTYSVNTFSLDMLGPFGGYKNSGLGREFGPEGFSAYLEHKMIHLPQGWDGETH&LINK_LOC=protein&PAGE_TYPE=BlastSearch', 'BLAST this protein','Strvi_7409')" />

 
 
   STDLDRAVRERLQRHGVAVREAVVDDLAGVAGRVVDTRPVPVPAGGRTLLLQHFLGYRVRSRRPVFDPRTVTLMDFRTPQPAGGVAFGYVLPTSPTEALV EYTEFSADLLTDAGYRARLGEHLERLRVGPHEVLEEERGVIPMTDARFPASPDPRVLRWGANSGSVRPSTGYSFSALQRQADDLAVQAVRGGPLRLPPAH RPRHLLMDSLVLQALSTGRVDGARFFVDLFARNPVQRVLGFLDGSTTPGEDLALMATSPRVAMLGTVLSRFPGAGGVVGLRR', 'http://blast.ncbi.nlm.nih.gov/Blast.cgi?PAGE=Proteins&PROGRAM=blastp&BLAST_PROGRAMS=blastp&QUERY=MPNPRGGAAAPALTVVGLGAAGASLAWRLAGTGVPFEVVEAPAGSGRRSQDRTWSTWGPPGGPGPFGPLVAASWPRVRVVGPDGTVVTADLGAWRYRMLR STDLDRAVRERLQRHGVAVREAVVDDLAGVAGRVVDTRPVPVPAGGRTLLLQHFLGYRVRSRRPVFDPRTVTLMDFRTPQPAGGVAFGYVLPTSPTEALV EYTEFSADLLTDAGYRARLGEHLERLRVGPHEVLEEERGVIPMTDARFPASPDPRVLRWGANSGSVRPSTGYSFSALQRQADDLAVQAVRGGPLRLPPAH RPRHLLMDSLVLQALSTGRVDGARFFVDLFARNPVQRVLGFLDGSTTPGEDLALMATSPRVAMLGTVLSRFPGAGGVVGLRR&LINK_LOC=protein&PAGE_TYPE=BlastSearch', 'BLAST this protein','Krad_0091')" />
   RSAPVYDRAAEAERRLDAVRISVPAGELHDDGERVTVRDPDGRDLVRAGWVLDSRPRRPKRPGRTSWLQHFRGWWLAADRPTFDPERAVLMDFRTPQPAR GVSFGYVLPVDDRFALVEYTEFGPALLDDAGYDAALRGYADLLGLDLTALRVREVEDGVIPMTDGPFESRPSPRVVRLGTAGGATRPSTGFTFSAMLRQA DQVAGAHAAGRPPVPAPAYPGRHLWMDAVALRALDRGHVGGVEFFERLFDRNPPERVLRFLDGLTSPAEDLAVMRSSPLLPMTGAVLGDAAGRLRARLRR ', 'http://blast.ncbi.nlm.nih.gov/Blast.cgi?PAGE=Proteins&PROGRAM=blastp&BLAST_PROGRAMS=blastp&QUERY=MWQRGRMHASFPVDVDLALVGGGGAASLVLAALDRHDVTGLRVAVVDPVHKRGQDRTWAFWGLPGDDLDPMLSASWSQVDVVTPAGRRVLSLDPLRYAML RSAPVYDRAAEAERRLDAVRISVPAGELHDDGERVTVRDPDGRDLVRAGWVLDSRPRRPKRPGRTSWLQHFRGWWLAADRPTFDPERAVLMDFRTPQPAR GVSFGYVLPVDDRFALVEYTEFGPALLDDAGYDAALRGYADLLGLDLTALRVREVEDGVIPMTDGPFESRPSPRVVRLGTAGGATRPSTGFTFSAMLRQA DQVAGAHAAGRPPVPAPAYPGRHLWMDAVALRALDRGHVGGVEFFERLFDRNPPERVLRFLDGLTSPAEDLAVMRSSPLLPMTGAVLGDAAGRLRARLRR &LINK_LOC=protein&PAGE_TYPE=BlastSearch', 'BLAST this protein','Micau_3285')" />
   RSAPVYDRAAEAERRLDVVRISVPAGELHDDGERVTVRDPDGRDLVRAGWVLDSRPRRPKRPGRTSWLQHFRGWWLAADRPTFDPERAVLMDFRTPQPQR GVSFGYVLPIDDRFALVEYTEFGPALLDDAGYDAALRGYADLLGLDLTALRVREVEDGVIPMTDGPFESRPSPRVVRLGTAGGATRPSTGFTFSAMLRQA DQVAGALAAGRPPVPAPAYPGRHLWMDAVALRALDRGHVGGVEFFERLFDRNPPERVLRFLDGVTSPAEDLAVMRSSPLLPMTGAVLGDAAGRLRARLRR ', 'http://blast.ncbi.nlm.nih.gov/Blast.cgi?PAGE=Proteins&PROGRAM=blastp&BLAST_PROGRAMS=blastp&QUERY=MWQRGRMHASFPVDVDLALVGGGGAASLVLAALDRHDVTGLRVAVVDPVHKRGQDRTWAFWGLPGDDLDPMLSASWSQVDVVTPAGRRVLSLDPLRYAML RSAPVYDRAAEAERRLDVVRISVPAGELHDDGERVTVRDPDGRDLVRAGWVLDSRPRRPKRPGRTSWLQHFRGWWLAADRPTFDPERAVLMDFRTPQPQR GVSFGYVLPIDDRFALVEYTEFGPALLDDAGYDAALRGYADLLGLDLTALRVREVEDGVIPMTDGPFESRPSPRVVRLGTAGGATRPSTGFTFSAMLRQA DQVAGALAAGRPPVPAPAYPGRHLWMDAVALRALDRGHVGGVEFFERLFDRNPPERVLRFLDGVTSPAEDLAVMRSSPLLPMTGAVLGDAAGRLRARLRR &LINK_LOC=protein&PAGE_TYPE=BlastSearch', 'BLAST this protein','ML5_5108')" />
   DAHVRASAGEHVDQLPVLVTEVVDGVEHAVVRGTCPGGPGGGERELTASWVFDSRPPRPAPRGRTHLLQHFRGWFVRTPDDAFDPASAVLMDLRPPQPAN GVAFGYVLPLSPREALVEYTEFGREALTTPEYERALEDYCGLLGLGDVEVTAAEQGVIPMTDARFRPRAGRRVFRVGTAGGATRPSTGYTFSGVRRQTAA VARALAQGRAPVPPVPHRRRHLAMDAVMLRALDTGRVRGAEFFAGLFAANRLGDVLAFLDGGSRLPRELAMGLSTPVAAMSLTSLDQAWYALRGVGARSL SRGPGPARRR', 'http://blast.ncbi.nlm.nih.gov/Blast.cgi?PAGE=Proteins&PROGRAM=blastp&BLAST_PROGRAMS=blastp&QUERY=MADYDVAIIGGGAAGLTLTHQLRGVNDRRGRPLRVALVEPPPGPHTPPPRTWCFWEPDGGPWDHLLAARWRDLSVVGPDGAVHDSPAAPYVYKMLRSADV DAHVRASAGEHVDQLPVLVTEVVDGVEHAVVRGTCPGGPGGGERELTASWVFDSRPPRPAPRGRTHLLQHFRGWFVRTPDDAFDPASAVLMDLRPPQPAN GVAFGYVLPLSPREALVEYTEFGREALTTPEYERALEDYCGLLGLGDVEVTAAEQGVIPMTDARFRPRAGRRVFRVGTAGGATRPSTGYTFSGVRRQTAA VARALAQGRAPVPPVPHRRRHLAMDAVMLRALDTGRVRGAEFFAGLFAANRLGDVLAFLDGGSRLPRELAMGLSTPVAAMSLTSLDQAWYALRGVGARSL SRGPGPARRR&LINK_LOC=protein&PAGE_TYPE=BlastSearch', 'BLAST this protein','Ndas_3568')" />
   GRFEQLVHSRLAARPTARLLRATAHEVRDTPYGAEVRCTGADGRPLTLRGRHVFDSRPAHGIPPHRTLLLQHFRGWFVRTGAPRFDPGVADLMDFRVPQP RHGLAFGYVLPLAPDRALVEYTEFSRTPLTTPAYEAALRHYTADVLRLGPLTVESAEQGVIPMTDARFPGRTGPAVFRIGAAGGATRPATGYTFAAVQRQ SRAIAEALRHGRADVPPPHGRRQLAMDAVLLRALDTGRVDGPRFFTDLFRRVPMERLLRFLDGGSTPWEEFGIGLRTPVGPMLRTAAELPFLARRETDRR ETARRDTGRSEESPR', 'http://blast.ncbi.nlm.nih.gov/Blast.cgi?PAGE=Proteins&PROGRAM=blastp&BLAST_PROGRAMS=blastp&QUERY=MSRTGAAQDAADGVVVVGGGAAGLCLAHWLGKDPDIPVTVVEAPDGPLRPAERTWCYWEAGPGEFEEAVVSSWTRLRVRGPDGSAVESEPAPLRYRMVRS GRFEQLVHSRLAARPTARLLRATAHEVRDTPYGAEVRCTGADGRPLTLRGRHVFDSRPAHGIPPHRTLLLQHFRGWFVRTGAPRFDPGVADLMDFRVPQP RHGLAFGYVLPLAPDRALVEYTEFSRTPLTTPAYEAALRHYTADVLRLGPLTVESAEQGVIPMTDARFPGRTGPAVFRIGAAGGATRPATGYTFAAVQRQ SRAIAEALRHGRADVPPPHGRRQLAMDAVLLRALDTGRVDGPRFFTDLFRRVPMERLLRFLDGGSTPWEEFGIGLRTPVGPMLRTAAELPFLARRETDRR ETARRDTGRSEESPR&LINK_LOC=protein&PAGE_TYPE=BlastSearch', 'BLAST this protein','SACTE_5151')" />
   DQAATAEHRLGAVRVAAPARAVEDDGDRVTVRAGGATVRASWVLDSRPRPPRRVGRTNWLQHFRGWWLEADRPLFDPGRAVLMDFRTPQPARGVSFGYLL PVTDRYALVEYTEFTPGLLTDAGYDAALAGYRDQLGLDPGRLRVREVENGVIPMTDGRFDLRPSPRVVRLGTAGGATRPSTGFTFAAMYRQAGQIAEALA AGRAPVPAPAYPRRHRWLDAVALRALDRGGVGGPDFFDRLFDRNPAERVLRFLDGTTSPAEEVALMGTTRLSPMVAATVGDAAARLRDRVVPWRRPTTWQ IPPPVTGATRRSPPQR', 'http://blast.ncbi.nlm.nih.gov/Blast.cgi?PAGE=Proteins&PROGRAM=blastp&BLAST_PROGRAMS=blastp&QUERY=MPTAPPVDVDLALVGGGGAGSLVLAALDRCGVRGLRVAVVDPVRRRGQDRTWAFWGRPDDRLDSLLAASWSQVEVATPGRRRVLDLAPLRYAMLRSAAVY DQAATAEHRLGAVRVAAPARAVEDDGDRVTVRAGGATVRASWVLDSRPRPPRRVGRTNWLQHFRGWWLEADRPLFDPGRAVLMDFRTPQPARGVSFGYLL PVTDRYALVEYTEFTPGLLTDAGYDAALAGYRDQLGLDPGRLRVREVENGVIPMTDGRFDLRPSPRVVRLGTAGGATRPSTGFTFAAMYRQAGQIAEALA AGRAPVPAPAYPRRHRWLDAVALRALDRGGVGGPDFFDRLFDRNPAERVLRFLDGTTSPAEEVALMGTTRLSPMVAATVGDAAARLRDRVVPWRRPTTWQ IPPPVTGATRRSPPQR&LINK_LOC=protein&PAGE_TYPE=BlastSearch', 'BLAST this protein','Sare_2557')" />
   ALMAHDLALSGGVQRVEATVEAVEGVPGGAEVYAYTAAGRPLPVRARWVFDSRPLGSLPAARTTLLQHFHGWFVRSDLPVFDPGTVELMDFRTPQPPRGL SFGYVLPTGRRQALVEYTEFSPAVLPRSAYEAALRHYTRDILHLPGLEIVSTETGVIPMTDAPFARQTAASVFRIGAAGGATRPSTGYTFAAVQRQTRAV AAALCRGRRPLPPPAHSARSRAMDAVLLRALDSGRIGGAAFFARLFSRVPMERLLRFLDGCTHLHEDLSIGVHTPVLPMLRSAAELPYLPRRPFPGP', 'http://blast.ncbi.nlm.nih.gov/Blast.cgi?PAGE=Proteins&PROGRAM=blastp&BLAST_PROGRAMS=blastp&QUERY=MLEADVAIVGAGAAGLSLAHRLACPVRGAPRISVVLLEAPPGPLRPPRRTWCFWERGPGRYDAAVTASWQRLRVRAPGGRPIEGDITPLRYKMIRSDDFE ALMAHDLALSGGVQRVEATVEAVEGVPGGAEVYAYTAAGRPLPVRARWVFDSRPLGSLPAARTTLLQHFHGWFVRSDLPVFDPGTVELMDFRTPQPPRGL SFGYVLPTGRRQALVEYTEFSPAVLPRSAYEAALRHYTRDILHLPGLEIVSTETGVIPMTDAPFARQTAASVFRIGAAGGATRPSTGYTFAAVQRQTRAV AAALCRGRRPLPPPAHSARSRAMDAVLLRALDSGRIGGAAFFARLFSRVPMERLLRFLDGCTHLHEDLSIGVHTPVLPMLRSAAELPYLPRRPFPGP&LINK_LOC=protein&PAGE_TYPE=BlastSearch', 'BLAST this protein','SAV_1021')" />
   ALVTGELARSDEVRRVEAAVETVDGVARGAEILARTGGGAPVAVRARWVFDSRPLGSLPAARTTLLQHFHGWYVRAERPVFDRGTVEFMDFRVPQPPGGL AFGYVLPTDSRRALVEYTEFSRAVLSPGAYDTALRRYTEEVLRLGDFAVVSTETGVIPMTDARFPRRSGPSVFRIGAAGGATRPSTGYTFSAIQRQTRAV AAALRAGRHPLPPPAHSARSRAMDAVLLRALDSGRVDGAAFFARLFARVPAERLLRFLDGETRLHEDLALGIRTPVLPMLRSAAELSWLPRRAAPPVGPA DTFPGRGPGRP', 'http://blast.ncbi.nlm.nih.gov/Blast.cgi?PAGE=Proteins&PROGRAM=blastp&BLAST_PROGRAMS=blastp&QUERY=MLEADVAIVGAGAAGLSLAHRLSRPAPGGPRLSVLLLDAPPGPSRPPVRTWCFWEAGPGPYDAALSAAWRRLRVRTPEGRLLDTDIAPLTYKMIRSDDFE ALVTGELARSDEVRRVEAAVETVDGVARGAEILARTGGGAPVAVRARWVFDSRPLGSLPAARTTLLQHFHGWYVRAERPVFDRGTVEFMDFRVPQPPGGL AFGYVLPTDSRRALVEYTEFSRAVLSPGAYDTALRRYTEEVLRLGDFAVVSTETGVIPMTDARFPRRSGPSVFRIGAAGGATRPSTGYTFSAIQRQTRAV AAALRAGRHPLPPPAHSARSRAMDAVLLRALDSGRVDGAAFFARLFARVPAERLLRFLDGETRLHEDLALGIRTPVLPMLRSAAELSWLPRRAAPPVGPA DTFPGRGPGRP&LINK_LOC=protein&PAGE_TYPE=BlastSearch', 'BLAST this protein','SCAB_5491')" />
   FERMVHGRLARTDGARLLRGTAESVRAVPAGTEVRCTLPGGRPLTLYARRVFDSRPLPELPPARTCLLQHFRGWFVHTRTDRFDPAVADLMDFRVPQPAH GLAFGYVLPLAPDRALVEYTEFSRAPLTTEAYESALGHYCRDILGLGELTVERTEQGVIPMTDARFPGRAGPAVYRIGTAGGATRPATGYTFAAVQRHSG AIAAALRDGHDRVPAPHGRRARAMDAVLLRALDTGRIDGPRFFTDLFRRVPAERLLRFLDGTTSLREEWGIGLRTPVRPMLRTAAEVPFLPRRSQPLART GGNNR', 'http://blast.ncbi.nlm.nih.gov/Blast.cgi?PAGE=Proteins&PROGRAM=blastp&BLAST_PROGRAMS=blastp&QUERY=MTPRSAQDSDVLVIGGGAAGLSLAHRLTENGTAPAMTLVEPPDGPLRPAERTWCYWGAAADGLEEAVGASWSVLRLHGADGGSVTVDPAPFTYRMVRSAD FERMVHGRLARTDGARLLRGTAESVRAVPAGTEVRCTLPGGRPLTLYARRVFDSRPLPELPPARTCLLQHFRGWFVHTRTDRFDPAVADLMDFRVPQPAH GLAFGYVLPLAPDRALVEYTEFSRAPLTTEAYESALGHYCRDILGLGELTVERTEQGVIPMTDARFPGRAGPAVYRIGTAGGATRPATGYTFAAVQRHSG AIAAALRDGHDRVPAPHGRRARAMDAVLLRALDTGRIDGPRFFTDLFRRVPAERLLRFLDGTTSLREEWGIGLRTPVRPMLRTAAEVPFLPRRSQPLART GGNNR&LINK_LOC=protein&PAGE_TYPE=BlastSearch', 'BLAST this protein','SCO_0191')" />
   ERLVHARLAAAPAARVVRATAHEVRDAPYGAEVRCTAADGRPLVLRGRHVFDSRPVRDTPPHRTLLLQHFRGWFVRTAVPRFDPGVADLMDFRVPQPGHG LAFGYVLPLAADRALVEYTEFSRAPLPTAAYEASLRQYTGDVLGLGRFTVESAEQGVIPMTDARFPRRAGRAVFRIGAAGGATRPATGYTFAAVQRQGRA VASALGRGRPDVPPPHGRRALAMDAVLLRALDTGRVDGPRFFTDLFRRVPMERLLRFLDGGSTPWEEFGIGLRTPVGPMLRTAAELPFLVRRATGRSEES L', 'http://blast.ncbi.nlm.nih.gov/Blast.cgi?PAGE=Proteins&PROGRAM=blastp&BLAST_PROGRAMS=blastp&QUERY=MTGHPQERVDDAVVVGGGAAGLCLAHWLTRTGTGVTLVEAPDGPLRPAERTWCYWEAGTGEFEEAVVASWRRLRVRGPDGAVVESDPAPLRYRMVRSGPF ERLVHARLAAAPAARVVRATAHEVRDAPYGAEVRCTAADGRPLVLRGRHVFDSRPVRDTPPHRTLLLQHFRGWFVRTAVPRFDPGVADLMDFRVPQPGHG LAFGYVLPLAADRALVEYTEFSRAPLPTAAYEASLRQYTGDVLGLGRFTVESAEQGVIPMTDARFPRRAGRAVFRIGAAGGATRPATGYTFAAVQRQGRA VASALGRGRPDVPPPHGRRALAMDAVLLRALDTGRVDGPRFFTDLFRRVPMERLLRFLDGGSTPWEEFGIGLRTPVGPMLRTAAELPFLVRRATGRSEES L&LINK_LOC=protein&PAGE_TYPE=BlastSearch', 'BLAST this protein','Sfla_0350')" />
   KMLRSDDFEALVHRRLSGAPGVRRVVATVSSVRDLPGSGAEVRVRDGGSERALVRGRYVFDSRPPRVLPSARTTLLQHFSGWFVRAERPVFDPAVPDLMD FRTSQPERGLSFGYVLPLDPHTALVEYTEFSPAPLTSEGYRRALDRYTREILGLGSFEVTAREHGVIPMTDGRFPRRVGRSVYRIGTAGGATRPSTGYTF AAIQRQSRAVAGRLRSGMPLRVPPAHGLRARTMDAVMLRALDTGRIDGPDFFGRLFRGVPAERLLTFLDGGSRWHEDLLIGVRTPVAPMLRTVAELPFVR RRPPQPLPPPPVRLEESSS', 'http://blast.ncbi.nlm.nih.gov/Blast.cgi?PAGE=Proteins&PROGRAM=blastp&BLAST_PROGRAMS=blastp&QUERY=MSAGKGAVPADVAEVDVVIVGAGAAGLSLAHHLCAPGGRLLSVVLVDAPPGRLRPPRRTWSFWEPESGPYDASLTASWSRLRVRAADGGVVVAGLPRLRY KMLRSDDFEALVHRRLSGAPGVRRVVATVSSVRDLPGSGAEVRVRDGGSERALVRGRYVFDSRPPRVLPSARTTLLQHFSGWFVRAERPVFDPAVPDLMD FRTSQPERGLSFGYVLPLDPHTALVEYTEFSPAPLTSEGYRRALDRYTREILGLGSFEVTAREHGVIPMTDGRFPRRVGRSVYRIGTAGGATRPSTGYTF AAIQRQSRAVAGRLRSGMPLRVPPAHGLRARTMDAVMLRALDTGRIDGPDFFGRLFRGVPAERLLTFLDGGSRWHEDLLIGVRTPVAPMLRTVAELPFVR RRPPQPLPPPPVRLEESSS&LINK_LOC=protein&PAGE_TYPE=BlastSearch', 'BLAST this protein','SGR_54t')" />
   ALVERRLARAPRLCRMEATASAVRDDPSGARGEVLTRGPGGERVLVTGRLVFDSRPPRRLPPARTTLLQHFTGWFVRTGRPVFDPATADLMDFRTPQPAR GLSFGYVLPLDPRTALVEYTEFSPAPLDTDGYRRALDHYTHDVLGIGPFDVTAREHGVIPMTDGRFPLRQGRSVYRIGTAGGATRPSTGYTFAAVQRQTR YIAGRLRSGRPLRVPTPYGRRARMMDAVLLRALDSGRVDGADFFDRLFRGVPGERLLRFMDGGSPPHEDLLIGLRTPVVPMLRTVVELPFRPRREHPAPP LAPSSTDTEESTP', 'http://blast.ncbi.nlm.nih.gov/Blast.cgi?PAGE=Proteins&PROGRAM=blastp&BLAST_PROGRAMS=blastp&QUERY=MPADFDVVIVGAGAAGMSLAYHLCAPGADVPVSVALVDAPPGPLRAPPRTWCFWEPPGGPYDQSLAASWPRLRIRAADGAATVARLSRLRYKMLCSDAFE ALVERRLARAPRLCRMEATASAVRDDPSGARGEVLTRGPGGERVLVTGRLVFDSRPPRRLPPARTTLLQHFTGWFVRTGRPVFDPATADLMDFRTPQPAR GLSFGYVLPLDPRTALVEYTEFSPAPLDTDGYRRALDHYTHDVLGIGPFDVTAREHGVIPMTDGRFPLRQGRSVYRIGTAGGATRPSTGYTFAAVQRQTR YIAGRLRSGRPLRVPTPYGRRARMMDAVLLRALDSGRVDGADFFDRLFRGVPGERLLRFMDGGSPPHEDLLIGLRTPVVPMLRTVVELPFRPRREHPAPP LAPSSTDTEESTP&LINK_LOC=protein&PAGE_TYPE=BlastSearch', 'BLAST this protein','SGR_6824')" />
   KMLRSDDFEALVHRRLSGAPGVRRVVATVSSVRDLPGSGAEVRVRDGGSERALVRGRYVFDSRPPRVLPSARTTLLQHFSGWFVRAERPVFDPAVPDLMD FRTSQPERGLSFGYVLPLDPHTALVEYTEFSPAPLTSEGYRRALDRYTREILGLGSFEVTAREHGVIPMTDGRFPRRVGRSVYRIGTAGGATRPSTGYTF AAIQRQSRAVAGRLRSGMPLRVPPAHGLRARTMDAVMLRALDTGRIDGPDFFGRLFRGVPAERLLTFLDGGSRWHEDLLIGVRTPVAPMLRTVAELPFVR RRPPQPLPPPPVRLEESSS', 'http://blast.ncbi.nlm.nih.gov/Blast.cgi?PAGE=Proteins&PROGRAM=blastp&BLAST_PROGRAMS=blastp&QUERY=MSAGKGAVPADVAEVDVVIVGAGAAGLSLAHHLCAPGGRLLSVVLVDAPPGRLRPPRRTWSFWEPESGPYDASLTASWSRLRVRAADGGVVVAGLPRLRY KMLRSDDFEALVHRRLSGAPGVRRVVATVSSVRDLPGSGAEVRVRDGGSERALVRGRYVFDSRPPRVLPSARTTLLQHFSGWFVRAERPVFDPAVPDLMD FRTSQPERGLSFGYVLPLDPHTALVEYTEFSPAPLTSEGYRRALDRYTREILGLGSFEVTAREHGVIPMTDGRFPRRVGRSVYRIGTAGGATRPSTGYTF AAIQRQSRAVAGRLRSGMPLRVPPAHGLRARTMDAVMLRALDTGRIDGPDFFGRLFRGVPAERLLTFLDGGSRWHEDLLIGVRTPVAPMLRTVAELPFVR RRPPQPLPPPPVRLEESSS&LINK_LOC=protein&PAGE_TYPE=BlastSearch', 'BLAST this protein','SGR_7085t')" />
   PVYDQAATAEQRLGAVRVAAPAEAVEDDGSRVTIHAGGSTLRASWVLDSRPRPPRRAGRTNWLQHFRGWWLEADRPLFDPGRAVLMDFRTPQPVRGVSFG YVLPVTDRYALVEYTEFTSGLLTDAGYDAALAGYRDRLGLDPGRLRVREVENGVIPMTDGPFDPRPSPRVVRLGTGGGATRPSTGFTFAAMHRQAGQIAE ALAAGRAPVPAPAYPRRHRWLDAVALRALDRGGVGGPDFFDRLFDRNPAERVLRFLDGTTSPAEEVALMSTTRPTPMVAATLGDAAARLRDRLVPWRRPA PGRVPPPVTGAPRSSHPAE', 'http://blast.ncbi.nlm.nih.gov/Blast.cgi?PAGE=Proteins&PROGRAM=blastp&BLAST_PROGRAMS=blastp&QUERY=MWQRVRVTTVDVDLALVGGGGAGSLVLAALDRWGVRGLRVAVIDPVRRRGQDRTWAFWGRPDDGLDPLLAASWSQVEVAVPGRRRVLDLAPLRYAMLRSA PVYDQAATAEQRLGAVRVAAPAEAVEDDGSRVTIHAGGSTLRASWVLDSRPRPPRRAGRTNWLQHFRGWWLEADRPLFDPGRAVLMDFRTPQPVRGVSFG YVLPVTDRYALVEYTEFTSGLLTDAGYDAALAGYRDRLGLDPGRLRVREVENGVIPMTDGPFDPRPSPRVVRLGTGGGATRPSTGFTFAAMHRQAGQIAE ALAAGRAPVPAPAYPRRHRWLDAVALRALDRGGVGGPDFFDRLFDRNPAERVLRFLDGTTSPAEEVALMSTTRPTPMVAATLGDAAARLRDRLVPWRRPA PGRVPPPVTGAPRSSHPAE&LINK_LOC=protein&PAGE_TYPE=BlastSearch', 'BLAST this protein','Strop_2408')" />
   RGSLQRVRTLHWTVTDIVDGPGEAVLRLRGADGAQRELAAGLVFDSRPLVGLPAEGVTLLQHFRGWFVRTDRPRFDSSAAVLMDLRVPQPERGVAFGYLL PFDARTALVEYTEFTREVCSDAAYDAALRDYTENLLHLGVFTVTGVEQGVIPMTSAHLPERVGHRVFRIGTAGGATRPATGYTFAGVQRQTRRIAAALVA GRFPVGYSAYPARHRFFDAVLLRGLDEGVVDGAEFFATLFRSNPLPRLLRFLDGSSRPWEELAIGLGTPVADMSTALWRQVRAGRRVASSLPR', 'http://blast.ncbi.nlm.nih.gov/Blast.cgi?PAGE=Proteins&PROGRAM=blastp&BLAST_PROGRAMS=blastp&QUERY=MERVDVAIVGAGAAGLSLAHRLDRVLPHRSVVLVETPEAALRSPERTWCFWEEETGEWDAAVCHRWHALAVQGPDGTTWRSRIHPLVYKMIRSRDFEALV RGSLQRVRTLHWTVTDIVDGPGEAVLRLRGADGAQRELAAGLVFDSRPLVGLPAEGVTLLQHFRGWFVRTDRPRFDSSAAVLMDLRVPQPERGVAFGYLL PFDARTALVEYTEFTREVCSDAAYDAALRDYTENLLHLGVFTVTGVEQGVIPMTSAHLPERVGHRVFRIGTAGGATRPATGYTFAGVQRQTRRIAAALVA GRFPVGYSAYPARHRFFDAVLLRGLDEGVVDGAEFFATLFRSNPLPRLLRFLDGSSRPWEELAIGLGTPVADMSTALWRQVRAGRRVASSLPR&LINK_LOC=protein&PAGE_TYPE=BlastSearch', 'BLAST this protein','Tfu_3088')" />
   RAAEAERLLGVTRVVASADTVTDHGDGVLVGGTDGPALRAGWVLDSRPRPPERPGRTNWLQHFRGWWLAADRPLFDPTRAVLMDFRTPQPTRGVSFGYVL PVSSRYALVEYTEFSPHLLTGAAYDRALGGYCDLLGLDPARFTVGEVENGVIPMTDGPFVARPSPRVVRLGTAGGATRPSTGFTFAAMYRQADQVARALA AGGPPVPGPAYPPRHRWMDAVALRALDRDLVDGPAFFARLFDRNPASRVLRFLDGATTVAEDLAVMNSTRLVPMAAAAVGDAAGRLRDRVVPSRRPAWRV PPPVVGSDP', 'http://blast.ncbi.nlm.nih.gov/Blast.cgi?PAGE=Proteins&PROGRAM=blastp&BLAST_PROGRAMS=blastp&QUERY=MGSVPLEVDLALLGGGGAASLVLAALARHGVRDLRIAVVDPVHRRGQDRTWAFWDRPGNDLDELLSASWSRVEVVTAERQRVLDVAPLRYAMLRSSPIYD RAAEAERLLGVTRVVASADTVTDHGDGVLVGGTDGPALRAGWVLDSRPRPPERPGRTNWLQHFRGWWLAADRPLFDPTRAVLMDFRTPQPTRGVSFGYVL PVSSRYALVEYTEFSPHLLTGAAYDRALGGYCDLLGLDPARFTVGEVENGVIPMTDGPFVARPSPRVVRLGTAGGATRPSTGFTFAAMYRQADQVARALA AGGPPVPGPAYPPRHRWMDAVALRALDRDLVDGPAFFARLFDRNPASRVLRFLDGATTVAEDLAVMNSTRLVPMAAAAVGDAAGRLRDRVVPSRRPAWRV PPPVVGSDP&LINK_LOC=protein&PAGE_TYPE=BlastSearch', 'BLAST this protein','VAB18032_21040')" />

 
 
   VGGGSGTGLIVPPGGSATVHGGVVRGWESGVALAEDAWDAPGRLVIEDVTFRENTTGLELQGLGTVAPDTDVTASRFVANGTGISALDLWGGVDLTVSAS RFTDNATALDVTSSTVAVADSVLAYNEQGVACDQSACRIEDSTLRDNGTAVSSIWYGTARVYRSTFVRNDIGVDSYWVLSIPNELVGNTFTANGTGVRFD SAHGVLTDNTFVRNDVGYEGRSEDGPPYFTAALVGNDFRRNGDGILSVVGESTLQANTAVANERWGIHAPGAVDLGGNTARGNGNEPQCVGVVCEGAGPA S', 'http://blast.ncbi.nlm.nih.gov/Blast.cgi?PAGE=Proteins&PROGRAM=blastp&BLAST_PROGRAMS=blastp&QUERY=MRVRSAVVRSALDVGSASLAAPPGRRRRRGLQTLTAAAAALVLGAGGSALAAPAAAAPVVGCGAVVEGEVTLGADLVCRNSPVGLTLLDGASLDLAGHRV VGGGSGTGLIVPPGGSATVHGGVVRGWESGVALAEDAWDAPGRLVIEDVTFRENTTGLELQGLGTVAPDTDVTASRFVANGTGISALDLWGGVDLTVSAS RFTDNATALDVTSSTVAVADSVLAYNEQGVACDQSACRIEDSTLRDNGTAVSSIWYGTARVYRSTFVRNDIGVDSYWVLSIPNELVGNTFTANGTGVRFD SAHGVLTDNTFVRNDVGYEGRSEDGPPYFTAALVGNDFRRNGDGILSVVGESTLQANTAVANERWGIHAPGAVDLGGNTARGNGNEPQCVGVVCEGAGPA S&LINK_LOC=protein&PAGE_TYPE=BlastSearch', 'BLAST this protein','Cfla_2896')" />

 
 
   WWRRRESLQAQAADTASTGSTTVATRHRTFLAPTSTAVTVVIAVLSIAAAGAACYDVYRIGDSGAQATWQGQFSTAPAPRGPGH', 'http://blast.ncbi.nlm.nih.gov/Blast.cgi?PAGE=Proteins&PROGRAM=blastp&BLAST_PROGRAMS=blastp&QUERY=MTTVNGLPAHILLVHAIVVLLPLAALLLVLSALWPAVRRKVAGPNAILAILVVVLVPITTDAGEWLERRVASTPLVRTHTELGDTALYVAIPVAVLALVV WWRRRESLQAQAADTASTGSTTVATRHRTFLAPTSTAVTVVIAVLSIAAAGAACYDVYRIGDSGAQATWQGQFSTAPAPRGPGH&LINK_LOC=protein&PAGE_TYPE=BlastSearch', 'BLAST this protein','AMED_3686')" />
   SWFSFFDREGSPRRPPVAVRRAATVVLAAIALGLAIGSTVEMILIGEAGSRAIWENSFSPTPLPATPTP', 'http://blast.ncbi.nlm.nih.gov/Blast.cgi?PAGE=Proteins&PROGRAM=blastp&BLAST_PROGRAMS=blastp&QUERY=MEINGLPLHPLLVHFVVVLVPLAAICTVLVPIWPAARRRLGIVTPLLALLNMVLVPIVTDAGEWLQKRVPSTPLIEQHAALGRMLYPCVIAVFVFAAVQW SWFSFFDREGSPRRPPVAVRRAATVVLAAIALGLAIGSTVEMILIGEAGSRAIWENSFSPTPLPATPTP&LINK_LOC=protein&PAGE_TYPE=BlastSearch', 'BLAST this protein','Lxx_12780')" />
   WWRRRESLQAQAADTASTGSTTVATRHRTFLAPTSTAVTVVIAVLSIAAAGAACYDVYRIGDSGAQATWQGQFSTAPAPRGPGH', 'http://blast.ncbi.nlm.nih.gov/Blast.cgi?PAGE=Proteins&PROGRAM=blastp&BLAST_PROGRAMS=blastp&QUERY=MTTVNGLPAHILLVHAIVVLLPLAALLLVLSALWPAVRRKVAGPNAILAILVVVLVPITTDAGEWLERRVASTPLVRTHTELGDTALYVAIPVAVLALVV WWRRRESLQAQAADTASTGSTTVATRHRTFLAPTSTAVTVVIAVLSIAAAGAACYDVYRIGDSGAQATWQGQFSTAPAPRGPGH&LINK_LOC=protein&PAGE_TYPE=BlastSearch', 'BLAST this protein','RAM_18770')" />

 
 
   ASDLVSNLKTQYPAGLVDACLNALPGAAMALLLGWGPVAAVVLAGVTWISSSGVIAKVLGDLGRLGNRETPVILSILVLEDLSMAVYLPILTALVAGTSL AAGSVTLAIALSVAGLVLLVAVRYGRHISRFVSSDDPEKLLLVVLGLTLLVAGVAQQLQVSAAVGAFLVGIALSGEVAEGAHGLLAPLRDLFAAVFFVFF GLHTDPASIPPVLLPALALAVVTAATKIATGYWAAKRAGVGTKGRWRAGGTLVARGEFSIVIAGLAVTAGIEPSLGPLATAYVLILVVLGPLTARYTEPV AAWFLRRRAPRPPSAEPLTASPAAEDRTGAAEPAAPQEAGRSARPAAGDQSSA', 'http://blast.ncbi.nlm.nih.gov/Blast.cgi?PAGE=Proteins&PROGRAM=blastp&BLAST_PROGRAMS=blastp&QUERY=MHLAAHLPAQPVVHLATQSESGAHSSAVFLIEFGAIILGLGLLGRLAGRLQFSPIPLYLLAGLAFGEGGLLPLGTSEEFVAIGAEIGVILLLLMLGLEYT ASDLVSNLKTQYPAGLVDACLNALPGAAMALLLGWGPVAAVVLAGVTWISSSGVIAKVLGDLGRLGNRETPVILSILVLEDLSMAVYLPILTALVAGTSL AAGSVTLAIALSVAGLVLLVAVRYGRHISRFVSSDDPEKLLLVVLGLTLLVAGVAQQLQVSAAVGAFLVGIALSGEVAEGAHGLLAPLRDLFAAVFFVFF GLHTDPASIPPVLLPALALAVVTAATKIATGYWAAKRAGVGTKGRWRAGGTLVARGEFSIVIAGLAVTAGIEPSLGPLATAYVLILVVLGPLTARYTEPV AAWFLRRRAPRPPSAEPLTASPAAEDRTGAAEPAAPQEAGRSARPAAGDQSSA&LINK_LOC=protein&PAGE_TYPE=BlastSearch', 'BLAST this protein','SCAB_86201')" />

 
 
   

 
 
   VVDYEATGRREYLDRATEIARDWVRDNPRGTAGVSPWAWAEHPVALRAPALVCLSAHVKDDWLAASLAEHAEILADATLYKGGHNHGLDQDIALLGIGCR FGEQRWTALALRRMTASARFAIDAQGVLREQAPRYGLYVHQRMRVAMEQIKDCGVGMPAELTRRWRSLEDYVAHATQPDGRLAPIGDSPADLRPKGISPR KDTVKVFTGGYVFGRTAWQDADSAYYSIRFGPGRALHGHEDHLGVSYHARRRDILVEAGFHSYEKTPYQRWTLSPQAHNVPVVVGADFREGTAARLLGEA AGRTRQSFRLADEAYGLTRTRSVLVEHGTDLMAVLDVVPRGRQVRPLWHLGPALSATAAGDGRVVLADGRWRASLIQLEMPACRPIGGQSVRSGEISPGY LKRIKVPTVVSPAAGALLTVIVPGAADPEISCSGGNVRVRTPGGTVSFTADVSTGLV', 'http://blast.ncbi.nlm.nih.gov/Blast.cgi?PAGE=Proteins&PROGRAM=blastp&BLAST_PROGRAMS=blastp&QUERY=MLDKKRGIIGLGALALTFLTFLAVLACGPVELSLADSRGRVTCQSGQFSTVTGTDAMRGRVAFVGLPPVELGRDVDWRADPYRNRSWALNLHTLRWMGRL VVDYEATGRREYLDRATEIARDWVRDNPRGTAGVSPWAWAEHPVALRAPALVCLSAHVKDDWLAASLAEHAEILADATLYKGGHNHGLDQDIALLGIGCR FGEQRWTALALRRMTASARFAIDAQGVLREQAPRYGLYVHQRMRVAMEQIKDCGVGMPAELTRRWRSLEDYVAHATQPDGRLAPIGDSPADLRPKGISPR KDTVKVFTGGYVFGRTAWQDADSAYYSIRFGPGRALHGHEDHLGVSYHARRRDILVEAGFHSYEKTPYQRWTLSPQAHNVPVVVGADFREGTAARLLGEA AGRTRQSFRLADEAYGLTRTRSVLVEHGTDLMAVLDVVPRGRQVRPLWHLGPALSATAAGDGRVVLADGRWRASLIQLEMPACRPIGGQSVRSGEISPGY LKRIKVPTVVSPAAGALLTVIVPGAADPEISCSGGNVRVRTPGGTVSFTADVSTGLV&LINK_LOC=protein&PAGE_TYPE=BlastSearch', 'BLAST this protein','Sros_3705')" />

 
 
   ILPRTVALSHNKLCTVSRTVELGTPVRTIIE', 'http://blast.ncbi.nlm.nih.gov/Blast.cgi?PAGE=Proteins&PROGRAM=blastp&BLAST_PROGRAMS=blastp&QUERY=MRVERVAEGRYLAHNDSGASIPIGGESAFSPVELLLAAIGGCTAVDTDVATSRRAEPTSFVVTVSGDKVSDEETGNRMKNLTVTFSVEFPEGEQGDQARA ILPRTVALSHNKLCTVSRTVELGTPVRTIIE&LINK_LOC=protein&PAGE_TYPE=BlastSearch', 'BLAST this protein','Svir_20740')" />

 
 
   IIDGGNSHFPDTIRRERALAEKGLHFVGVGVSGGEEGALWGPSIMPGGSAESYKHVGPMLEKISAKAPQDGSPCCAWISTDGSGHFVKMVHNGIEYADMQ VIGEAYDILRSVAGIEPAEQAEIFKKWNETDLNSYLIEITSEVLAQVDAKTGKPLVDVILDQAGQKGTGRWTVQAALDLGAPVSAIAESVFARALSSSDV KARAQAQQELPAGLIATTGVGAGDPEFVEDVRRALFASKLVAYAQGMDMIARAGQEYGWELHLDTIASLWRAGCIIRAELLGDIMAAYAGEVPANMLLAP AFKNLMEELVPSWRRVVAKATALGVPVPVFSSALSYYDGLRRDRLPAALIQGQRDFFGAHTYGRVDAEGAFHTMWSGDRSEVNAADIHVH', 'http://blast.ncbi.nlm.nih.gov/Blast.cgi?PAGE=Proteins&PROGRAM=blastp&BLAST_PROGRAMS=blastp&QUERY=MTENKKAQIGVTGLAVMGANLARNLARNGYTVALHNRSVEKTDALLSEHGADGDFIRTESLQDLVDSLESPRRILIMVKAGAPVDAVIEQLTPLLDEGDI IIDGGNSHFPDTIRRERALAEKGLHFVGVGVSGGEEGALWGPSIMPGGSAESYKHVGPMLEKISAKAPQDGSPCCAWISTDGSGHFVKMVHNGIEYADMQ VIGEAYDILRSVAGIEPAEQAEIFKKWNETDLNSYLIEITSEVLAQVDAKTGKPLVDVILDQAGQKGTGRWTVQAALDLGAPVSAIAESVFARALSSSDV KARAQAQQELPAGLIATTGVGAGDPEFVEDVRRALFASKLVAYAQGMDMIARAGQEYGWELHLDTIASLWRAGCIIRAELLGDIMAAYAGEVPANMLLAP AFKNLMEELVPSWRRVVAKATALGVPVPVFSSALSYYDGLRRDRLPAALIQGQRDFFGAHTYGRVDAEGAFHTMWSGDRSEVNAADIHVH&LINK_LOC=protein&PAGE_TYPE=BlastSearch', 'BLAST this protein','HMPREF0733_12158')" />
   IIDGGNSHFPDTIRRERALAEKGLHFVGVGVSGGEEGALWGPSMMPGGSAESYKHIGPMLEKIAAKAPQDGAPCCAWISTDGSGHFVKMVHNGIEYADMQ VIGEAYDLLRSVAGIEPAEQAEIFKTWNEGDLSSYLIEITAEVLAQVDAKTGKPLVDVIVDAAGQKGTGLWTAKAGLDLGSPVSAIAESVFARALSSSER EQREQAQRELPAGLIATTGIGAGDPEFVEDVRRALFASKLVAYAQGMDLLTRAGKEYGWDLKLDTIASLWRAGCIIRAELLGDIMAAYADEAPANMLLAP AFKNLMEELVPSWRRVVAKATALGIPVPVFASALSYYDGLRRDRLPASLIQGQRDFFGAHTYGRIDEEGAFHTMWSGDRSEVNAADIHVH', 'http://blast.ncbi.nlm.nih.gov/Blast.cgi?PAGE=Proteins&PROGRAM=blastp&BLAST_PROGRAMS=blastp&QUERY=MTENKKAQIGVTGLAVMGANLARNLARNGYTVALHNRSVEKTDALLAEHGADGDFIRTESLQELVDSLESPRRILIMVKAGAPVDAVIEQLTPLLDEGDI IIDGGNSHFPDTIRRERALAEKGLHFVGVGVSGGEEGALWGPSMMPGGSAESYKHIGPMLEKIAAKAPQDGAPCCAWISTDGSGHFVKMVHNGIEYADMQ VIGEAYDLLRSVAGIEPAEQAEIFKTWNEGDLSSYLIEITAEVLAQVDAKTGKPLVDVIVDAAGQKGTGLWTAKAGLDLGSPVSAIAESVFARALSSSER EQREQAQRELPAGLIATTGIGAGDPEFVEDVRRALFASKLVAYAQGMDLLTRAGKEYGWDLKLDTIASLWRAGCIIRAELLGDIMAAYADEAPANMLLAP AFKNLMEELVPSWRRVVAKATALGIPVPVFASALSYYDGLRRDRLPASLIQGQRDFFGAHTYGRIDEEGAFHTMWSGDRSEVNAADIHVH&LINK_LOC=protein&PAGE_TYPE=BlastSearch', 'BLAST this protein','RMDY18_07920')" />

 
 
   LITAITATGHHLLTLLVAVTITLGGFYSPDKAHFAFESIALMIFCTIATLAYLLGLWIHRNHQQRLNTQRAQQARRQQLTSLLHDTIAADLTSIIAQVEK LAITTPQRHDELKDIARTARNALDRTRQLLTTLNTHPNTAPTTSLPITLDTTTKRLRDHGFTVTTTTRLTTPVTMTLPNTALERVLSETATNIIKHATPH SAVAIDTTSDDEGVTITVTNHRIPVQAKPTGSIHLGLTSMSQTLHTLGGTLITRSDEQEWSTIVHLPFP', 'http://blast.ncbi.nlm.nih.gov/Blast.cgi?PAGE=Proteins&PROGRAM=blastp&BLAST_PROGRAMS=blastp&QUERY=MMSPMLIPPTNTAGSLKLMLAQRLPTTVILATLALIFSSASILNEYLITPLITVLIITQALSLIVAPRYPRISTGIYITAFLGALLAGHSTGVELFLGVF LITAITATGHHLLTLLVAVTITLGGFYSPDKAHFAFESIALMIFCTIATLAYLLGLWIHRNHQQRLNTQRAQQARRQQLTSLLHDTIAADLTSIIAQVEK LAITTPQRHDELKDIARTARNALDRTRQLLTTLNTHPNTAPTTSLPITLDTTTKRLRDHGFTVTTTTRLTTPVTMTLPNTALERVLSETATNIIKHATPH SAVAIDTTSDDEGVTITVTNHRIPVQAKPTGSIHLGLTSMSQTLHTLGGTLITRSDEQEWSTIVHLPFP&LINK_LOC=protein&PAGE_TYPE=BlastSearch', 'BLAST this protein','CRES_0450')" />

 
 
   TRWLSANIGEGLILDLRTSVFDHVQRMPVAFFTRTRTGALVSRLNNDVIGAQRAFSDTLSGVVGNMVTLLLTLVVMIGISWQITLLTLVLLPLFLLPARR VGGRLAKLRREAAAHNAAMSTQMTERFSAPGATLVKLFGRPADESAEFAARTRRVRDIGVRTAMVQVSFVTALTLVSALALALVYGLGGWFALHGRLDPG AVVALALLLTRLYAPLTALAGARVEVMSALVSFERVFEVLDLEPLIKEKPDAREVPDGPVSVEFDRVDFGYPAADKVSLASLEEVATLDTRGGVQVLHQL SFRAEPGQMVALVGSSGAGKSTIAQLLPRLYDADGGAVRLSGVDVRDLTADSIRATLGMVTQDGHLFHDSIRANLLLARPEATDEELWEVLRRARLEGLI ASLPDGLETVVGERGYRLSGGERQRLTIARLLLARPRVVILDEATAHLDATSEADVQEALGEALEGRTAMVIAHRLSTVRAADLILVVEDGRIVERGTHT ALLAAGGRYEELYRTQFEQPAPMGDGFAVDGASAPGTGTKVAVVDGASAPGTGAEVTALDGGPTAAEAPAT', 'http://blast.ncbi.nlm.nih.gov/Blast.cgi?PAGE=Proteins&PROGRAM=blastp&BLAST_PROGRAMS=blastp&QUERY=MDIEVTAWHSLYQASNASDDRRPFSRETLRRIGAFARPHRRRLQLFLVLSTVTAVLAVATPLLAGRVVDAIVHHSGQDTVLGLAGLIAAIAVAEAAIGLL TRWLSANIGEGLILDLRTSVFDHVQRMPVAFFTRTRTGALVSRLNNDVIGAQRAFSDTLSGVVGNMVTLLLTLVVMIGISWQITLLTLVLLPLFLLPARR VGGRLAKLRREAAAHNAAMSTQMTERFSAPGATLVKLFGRPADESAEFAARTRRVRDIGVRTAMVQVSFVTALTLVSALALALVYGLGGWFALHGRLDPG AVVALALLLTRLYAPLTALAGARVEVMSALVSFERVFEVLDLEPLIKEKPDAREVPDGPVSVEFDRVDFGYPAADKVSLASLEEVATLDTRGGVQVLHQL SFRAEPGQMVALVGSSGAGKSTIAQLLPRLYDADGGAVRLSGVDVRDLTADSIRATLGMVTQDGHLFHDSIRANLLLARPEATDEELWEVLRRARLEGLI ASLPDGLETVVGERGYRLSGGERQRLTIARLLLARPRVVILDEATAHLDATSEADVQEALGEALEGRTAMVIAHRLSTVRAADLILVVEDGRIVERGTHT ALLAAGGRYEELYRTQFEQPAPMGDGFAVDGASAPGTGTKVAVVDGASAPGTGAEVTALDGGPTAAEAPAT&LINK_LOC=protein&PAGE_TYPE=BlastSearch', 'BLAST this protein','Strvi_3078')" />

 
 
   AEQIGRPILSSRIALGSGVAGRDRVAIRMANIDATLAAVKKAAESRA', 'http://blast.ncbi.nlm.nih.gov/Blast.cgi?PAGE=Proteins&PROGRAM=blastp&BLAST_PROGRAMS=blastp&QUERY=MEQATIWVDAPPERVWKLISDPTRYGEWSLENQGGTWKSPPGPGAQFKGRNKRGLARWATTCTVTDYQAPSRFTFEVHESAMRWGYILEPRDGGTTVTEF AEQIGRPILSSRIALGSGVAGRDRVAIRMANIDATLAAVKKAAESRA&LINK_LOC=protein&PAGE_TYPE=BlastSearch', 'BLAST this protein','Caci_3553')" />

 
 
   TDAYRRVTADQFTAPSTHRSASVPYGLTLYLGLGVGAAAAAPVLGLLLGLHALGVALLTLGARLTALTLRAADRAVMILRGLPRGMLCPSCFERVPYPAY DCPRTTCRRRHADIRPGTFGLFRRRCACDERIPTLLMLMSRDARLSGHCVHEHCGKPMNPDAGHMPELILPLIGGRAAGKTQLMAAMVKALENTAENGGP AIRLADPESTANQRVLNEVLEIQGHTRPTQKTLPRAHSFVRGSGRAERLVHVFDTAGERFVNREETDALRYIREARTFVFVLDPMAVGAFWTRLDPAGPK VDRTLASTVDPEDVFSRSVQTVRTMGTRLDKARLAVAVSKRDLMTGQSALLPDRPDHSDTTRDWLCERLGLRNLLKTMDLEFGEVRFFCTAAVADEEGRV DPSIGVFVDWCLRE', 'http://blast.ncbi.nlm.nih.gov/Blast.cgi?PAGE=Proteins&PROGRAM=blastp&BLAST_PROGRAMS=blastp&QUERY=MSDILTFIIVPALYLALLLLYLVFVPVIAALRGAALVSELLWRYCRLLGGVLRLRTPEFVTIPPYRPADERAHRNYFFGPATRDLRQLLTLGRRLYVRTV TDAYRRVTADQFTAPSTHRSASVPYGLTLYLGLGVGAAAAAPVLGLLLGLHALGVALLTLGARLTALTLRAADRAVMILRGLPRGMLCPSCFERVPYPAY DCPRTTCRRRHADIRPGTFGLFRRRCACDERIPTLLMLMSRDARLSGHCVHEHCGKPMNPDAGHMPELILPLIGGRAAGKTQLMAAMVKALENTAENGGP AIRLADPESTANQRVLNEVLEIQGHTRPTQKTLPRAHSFVRGSGRAERLVHVFDTAGERFVNREETDALRYIREARTFVFVLDPMAVGAFWTRLDPAGPK VDRTLASTVDPEDVFSRSVQTVRTMGTRLDKARLAVAVSKRDLMTGQSALLPDRPDHSDTTRDWLCERLGLRNLLKTMDLEFGEVRFFCTAAVADEEGRV DPSIGVFVDWCLRE&LINK_LOC=protein&PAGE_TYPE=BlastSearch', 'BLAST this protein','SBI_00527')" />

 
 
   NAFTNAGLQFDDLKAMFITHLHTDHIVDYYNFFLSGGFLAPPGRAPVLVYGPGPAGGLPPSEVGNPNPATVNPANPTPGLAAATEALHRAFAYTSNIFIR DYGIDNVADLVKVTEIGLPPGSDYRNRAPKMSPFSVASDDNVSVTATLVSHYDVYPAFGFRFDLKKSGVSVTFSGDTTKSDNLITLAQGTDILVHEAVFS LDTAYFGNAFPPNYLVNSHTSAEQVGEVAAAAKPKQLILSHYAPDDLPDSQWLDKIKKNYSGMTTIARDGQVFAL', 'http://blast.ncbi.nlm.nih.gov/Blast.cgi?PAGE=Proteins&PROGRAM=blastp&BLAST_PROGRAMS=blastp&QUERY=MLLGMHQAGHVGTHERRAAATRRSALTAAGLAVVGAGVLGASACSPQKSPQPSSPRLPDNALITLGVAAGPPPTPSRVGISSVLKIGRDLYVIDCGLGSL NAFTNAGLQFDDLKAMFITHLHTDHIVDYYNFFLSGGFLAPPGRAPVLVYGPGPAGGLPPSEVGNPNPATVNPANPTPGLAAATEALHRAFAYTSNIFIR DYGIDNVADLVKVTEIGLPPGSDYRNRAPKMSPFSVASDDNVSVTATLVSHYDVYPAFGFRFDLKKSGVSVTFSGDTTKSDNLITLAQGTDILVHEAVFS LDTAYFGNAFPPNYLVNSHTSAEQVGEVAAAAKPKQLILSHYAPDDLPDSQWLDKIKKNYSGMTTIARDGQVFAL&LINK_LOC=protein&PAGE_TYPE=BlastSearch', 'BLAST this protein','MAF_38110')" />
   NAFTNAGLQFDDLKAMFITHLHTDHIVDYYNFFLSGGFLAPPGRAPVLVYGPGPAGGLPPSEVGNPNPATVNPANPTPGLAAATEALHRAFAYTSNIFIR DYGIDNVADLVKVTEIGLPPGSDYRNRAPKMSPFSVASDDNVSVTATLVSHYDVYPAFGFRFDLKKSGVSVTFSGDTTKSDNLITLAQGTDILVHEAVFS LDTAYFGNAFPPNYLVNSHISAEQVGEVAAAAKPKQLILSHYAPDDLPDSQWLDKIKKNYSGMTTIARDGQVFAL', 'http://blast.ncbi.nlm.nih.gov/Blast.cgi?PAGE=Proteins&PROGRAM=blastp&BLAST_PROGRAMS=blastp&QUERY=MLLGMHQAGHVGTHERRAAATRRSALTAAGLAVVGAGVLGASACSPQKSPQPSSPRLPDNALITLGVAAGPPPTPSRVGISSVLKIGRDLYVIDCGLGSL NAFTNAGLQFDDLKAMFITHLHTDHIVDYYNFFLSGGFLAPPGRAPVLVYGPGPAGGLPPSEVGNPNPATVNPANPTPGLAAATEALHRAFAYTSNIFIR DYGIDNVADLVKVTEIGLPPGSDYRNRAPKMSPFSVASDDNVSVTATLVSHYDVYPAFGFRFDLKKSGVSVTFSGDTTKSDNLITLAQGTDILVHEAVFS LDTAYFGNAFPPNYLVNSHISAEQVGEVAAAAKPKQLILSHYAPDDLPDSQWLDKIKKNYSGMTTIARDGQVFAL&LINK_LOC=protein&PAGE_TYPE=BlastSearch', 'BLAST this protein','Mb_3825')" />
   NAFTNAGLQFDDLKAMFITHLHTDHIVDYYNFFLSGGFLAPPGRAPVLVYGPGPAGGLPPSEVGNPNPATVNPANPTPGLAAATEALHRAFAYTSNIFIR DYGIDNVADLVKVTEIGLPPGSDYRNRAPKMSPFSVASDDNVSVTATLVSHYDVYPAFGFRFDLKKSGVSVTFSGDTTKSDNLITLAQGTDILVHEAVFS LDTAYFGNAFPPNYLVNSHTSAEQVGEVAAAAKPKQLILSHYAPDDLPDSQWLDKIKKNYSGMTTIARDGQVFAL', 'http://blast.ncbi.nlm.nih.gov/Blast.cgi?PAGE=Proteins&PROGRAM=blastp&BLAST_PROGRAMS=blastp&QUERY=MLLGMHQAGHVGTHERRAAATRRSALTAAGLAVVGAGVLGASACSPQKSPQPSSPRLPDNALITLGVAAGPPPTPSRVGISSVLKIGRDLYVIDCGLGSL NAFTNAGLQFDDLKAMFITHLHTDHIVDYYNFFLSGGFLAPPGRAPVLVYGPGPAGGLPPSEVGNPNPATVNPANPTPGLAAATEALHRAFAYTSNIFIR DYGIDNVADLVKVTEIGLPPGSDYRNRAPKMSPFSVASDDNVSVTATLVSHYDVYPAFGFRFDLKKSGVSVTFSGDTTKSDNLITLAQGTDILVHEAVFS LDTAYFGNAFPPNYLVNSHTSAEQVGEVAAAAKPKQLILSHYAPDDLPDSQWLDKIKKNYSGMTTIARDGQVFAL&LINK_LOC=protein&PAGE_TYPE=BlastSearch', 'BLAST this protein','MCAN_38161')" />
   CGLGSLNAFTNAGLQFNDLKAIFITHLHTDHIVDYYSFFLSGGFQAPPGRAPIPVYGPGPAGGLPPSEIGNKNPATVNPANPTPGLAATTEALHQAFAYS SNIFIRDYGIDNVQDLAKVIEIPVPAGSDYRNTAPKVSPFPVVSDDNVSVTTTLVPHYDVYPAFAFRFDVKDPAVSITFSGDTTKSDNLVALANGTDILV HEAMFSLDTAYYGNVFPPDYLPKSHTSAEQVGEVAAVVKPKHLILSHYAPDDLPDSQWLDKINQNYSGRTTVAKDGQIFAL', 'http://blast.ncbi.nlm.nih.gov/Blast.cgi?PAGE=Proteins&PROGRAM=blastp&BLAST_PROGRAMS=blastp&QUERY=MTQPKLHGMHQACDHSGASIVAAVSSRRSALTAAGLAVVGAGVLATSACGPGESPAPPSPAALPENALITLGVAAGPPPTPNKIGISSVLKIGGDLYLID CGLGSLNAFTNAGLQFNDLKAIFITHLHTDHIVDYYSFFLSGGFQAPPGRAPIPVYGPGPAGGLPPSEIGNKNPATVNPANPTPGLAATTEALHQAFAYS SNIFIRDYGIDNVQDLAKVIEIPVPAGSDYRNTAPKVSPFPVVSDDNVSVTTTLVPHYDVYPAFAFRFDVKDPAVSITFSGDTTKSDNLVALANGTDILV HEAMFSLDTAYYGNVFPPDYLPKSHTSAEQVGEVAAVVKPKHLILSHYAPDDLPDSQWLDKINQNYSGRTTVAKDGQIFAL&LINK_LOC=protein&PAGE_TYPE=BlastSearch', 'BLAST this protein','MMAR_5358')" />
   NAFTNAGLQFDDLKAMFITHLHTDHIVDYYNFFLSGGFLAPPGRAPVLVYGPGPAGGLPPSEVGNPNPATVNPANPTPGLAAATEALHRAFAYTSNIFIR DYGIDNVADLVKVTEIGLPPGSDYRNRAPKMSPFSVASDDNVSVTATLVSHYDVYPAFGFRFDLKKSGVSVTFSGDTTKSDNLITLAQGTDILVHEAVFS LDTAYFGNAFPPNYLVNSHTSAEQVGEVAAAAKPKQLILSHYAPDDLPDSQWLDKIKKNYSGMTTIARDGQVFAL', 'http://blast.ncbi.nlm.nih.gov/Blast.cgi?PAGE=Proteins&PROGRAM=blastp&BLAST_PROGRAMS=blastp&QUERY=MLLGMHQAGHVGTHERRAAATRRSALTAAGLAVVGAGVLGASACSPQKSPQPSSPRLPDNALITLGVAAGPPPTPSRVGISSVLKIGRDLYVIDCGLGSL NAFTNAGLQFDDLKAMFITHLHTDHIVDYYNFFLSGGFLAPPGRAPVLVYGPGPAGGLPPSEVGNPNPATVNPANPTPGLAAATEALHRAFAYTSNIFIR DYGIDNVADLVKVTEIGLPPGSDYRNRAPKMSPFSVASDDNVSVTATLVSHYDVYPAFGFRFDLKKSGVSVTFSGDTTKSDNLITLAQGTDILVHEAVFS LDTAYFGNAFPPNYLVNSHTSAEQVGEVAAAAKPKQLILSHYAPDDLPDSQWLDKIKKNYSGMTTIARDGQVFAL&LINK_LOC=protein&PAGE_TYPE=BlastSearch', 'BLAST this protein','Rv_3796')" />

 
 
   ALCVQRRRGGFLLVEAEAGMGKSALAAYLAFTRAWPAHFTRLAEGRDSATARRNLAAQLIARWKLTDAAPGGVLPEGAGTTGWLHGRLCDSATARDRDDP GVPVVLLIDGLDEAPVSVGGELPLGLPPNLPAGTVIVATTRPKSIRIPAGSRVVERIDVESEANRTDLLDYLTVVTARDPLLSEPLNRAQMDSHRFCRTL VERSGGVWIYALSILDQIRDHGRSPKGVDRLPASLAGYYAENVRRWQDELGDHLWETAGLPVLTTVTAIREPMSVARIAAWADVPPAMTRTILRGMLKPF LAVRRGGDPDLYVPRHQSLRDFCDGTSLLDSDDDGLRELAYDLAAATRAAHRRIAVAVEPSGPVGRRDWAVAGRYARTYLAEHAALGGQLDELITDPQFG LFVGVACLLRQRRHLTTAEAKKALAALELAAGHNNDDALWLRWIEVSARKMRADTFADRAASLVDETWHPIRAMWTGNSHRTLIGHTKTVRAVTAVPLRD GRTLLASAGDDGSIRLWDPIEGTPAGTLTGHTARVFALAVVPLRDGRTLLASAGDDGSIRLWDPIEGTPAGTLTGHTVGGFSLAVVPLTDGRTLLASAGA DKAVRLWDPVAGTLAGTLTGHTDWVRAVTAVPLPDGGTLLATAGDDRAVRLWDPIEGTPAGTLTGHTDWVNALTAVPLPDGGTLLASAGSDGSVRLWDPI TATLTGHTGRVNALAVVPLPDDGALLASAGNDGSVRLWDPIAATAVGTLTGHTAGVRAVTAVPLPDGGTLLATAGDDRAVRLWDPIEGTPAGTLTGHTDW VNALTAVPLPDGGTLLASAGSDGSVRLWDPITATLTGTLSSHTDWVRTLAAVPLPGGGILLASAGAEGSLRLWDPTEGTPAGILTGHTGWVRTLAAVPLP GGGTLLASAGNDGSVRLWDPIAATAVGALTGHTAGVNALTAVPLPDSRILLASAGDDGSVRLWNPVTAVAVGALTGHTEPVNAVATLALPDGRVLLASAG YDGSVRLWDPIAGTLAGILTGHTAGVNALTAVPLPDSRILLASAGDDGSVRLWNPVTAVAVGALTGHTEPVNAVATLALPDGRVLLASAGDDRAIILWQA LAGTVL', 'http://blast.ncbi.nlm.nih.gov/Blast.cgi?PAGE=Proteins&PROGRAM=blastp&BLAST_PROGRAMS=blastp&QUERY=MTQTPQHFSTASGDDRASQYDAQIKVGDLSAPGGGQAVGINYGTAVQQYFQGPFRLLRQAVIDLDPLPGDLRLVDPEEPTNPVGLFRGREKLIGKIDAFL ALCVQRRRGGFLLVEAEAGMGKSALAAYLAFTRAWPAHFTRLAEGRDSATARRNLAAQLIARWKLTDAAPGGVLPEGAGTTGWLHGRLCDSATARDRDDP GVPVVLLIDGLDEAPVSVGGELPLGLPPNLPAGTVIVATTRPKSIRIPAGSRVVERIDVESEANRTDLLDYLTVVTARDPLLSEPLNRAQMDSHRFCRTL VERSGGVWIYALSILDQIRDHGRSPKGVDRLPASLAGYYAENVRRWQDELGDHLWETAGLPVLTTVTAIREPMSVARIAAWADVPPAMTRTILRGMLKPF LAVRRGGDPDLYVPRHQSLRDFCDGTSLLDSDDDGLRELAYDLAAATRAAHRRIAVAVEPSGPVGRRDWAVAGRYARTYLAEHAALGGQLDELITDPQFG LFVGVACLLRQRRHLTTAEAKKALAALELAAGHNNDDALWLRWIEVSARKMRADTFADRAASLVDETWHPIRAMWTGNSHRTLIGHTKTVRAVTAVPLRD GRTLLASAGDDGSIRLWDPIEGTPAGTLTGHTARVFALAVVPLRDGRTLLASAGDDGSIRLWDPIEGTPAGTLTGHTVGGFSLAVVPLTDGRTLLASAGA DKAVRLWDPVAGTLAGTLTGHTDWVRAVTAVPLPDGGTLLATAGDDRAVRLWDPIEGTPAGTLTGHTDWVNALTAVPLPDGGTLLASAGSDGSVRLWDPI TATLTGHTGRVNALAVVPLPDDGALLASAGNDGSVRLWDPIAATAVGTLTGHTAGVRAVTAVPLPDGGTLLATAGDDRAVRLWDPIEGTPAGTLTGHTDW VNALTAVPLPDGGTLLASAGSDGSVRLWDPITATLTGTLSSHTDWVRTLAAVPLPGGGILLASAGAEGSLRLWDPTEGTPAGILTGHTGWVRTLAAVPLP GGGTLLASAGNDGSVRLWDPIAATAVGALTGHTAGVNALTAVPLPDSRILLASAGDDGSVRLWNPVTAVAVGALTGHTEPVNAVATLALPDGRVLLASAG YDGSVRLWDPIAGTLAGILTGHTAGVNALTAVPLPDSRILLASAGDDGSVRLWNPVTAVAVGALTGHTEPVNAVATLALPDGRVLLASAGDDRAIILWQA LAGTVL&LINK_LOC=protein&PAGE_TYPE=BlastSearch', 'BLAST this protein','FRAAL_6340')" />
   IYIDQIRAGIAHPTVDLRAEDWTPRWAIGAHFSPALFETLTGYFRKVTALACTTVNGDSVAIIGSIDGTVRACSLTTGQQYRPDPTDCPEVVTALACTTI NGDPVAIIGGTAGTLRIWNLITGIEHRRSLFGIRAVRALTCTTINGDPTAILVSDDETVWRCSLTNRFRNDLVRLTISKPITALTCATVEEQPVVITGSK SGNVRLHNLNANNEWRVTLPKRNCEVRALDFTTVNGDPLIVIGDYDGSLRILNLATREQTWLNVSEATEDPNSYPRARRKEVVSATSSEEDGSSIAVTID FKGTLRIWDLVAGTHYCPDLDRNTALWSPALCATVDGRSVIIVGSRDGKVRVWDSAIEQQTESKPISVEKVTAIACTVANSNPVAVTLNNRATVQVLDLA TAERLRLDIRGHSGHLTAVACTTVDGQPIAVIGANDGTVQAWNLTTRQSHCEPLTESNDLILAVACTTIGGQPVAVIGGNDGTVCVWNLGTGQQRRMVVS EEAGQITAVACTDINGELVVVIGCANGSLWVGNLATGQQYRPDFAKGVLPIRTLACTTVNSHAVVVLSGNEDSVQCWDLTTRRHLGPNQSSRVLPVRTLA CATVDNHPIAAICSNDGTVRVWDLQRHKQLTQPIRILDPVDCLAIAPDLSLVIAYNTEVMAFNPPQRWDWKEDT', 'http://blast.ncbi.nlm.nih.gov/Blast.cgi?PAGE=Proteins&PROGRAM=blastp&BLAST_PROGRAMS=blastp&QUERY=MTKFDDVRAIARAFVEYADSTWACDPEPYLCRHAAEFTAEASILAELLPQQDFLKNADPHRLVPLLRQELGGPAARLAISYLLHAGYHRNTPNPDQRALR IYIDQIRAGIAHPTVDLRAEDWTPRWAIGAHFSPALFETLTGYFRKVTALACTTVNGDSVAIIGSIDGTVRACSLTTGQQYRPDPTDCPEVVTALACTTI NGDPVAIIGGTAGTLRIWNLITGIEHRRSLFGIRAVRALTCTTINGDPTAILVSDDETVWRCSLTNRFRNDLVRLTISKPITALTCATVEEQPVVITGSK SGNVRLHNLNANNEWRVTLPKRNCEVRALDFTTVNGDPLIVIGDYDGSLRILNLATREQTWLNVSEATEDPNSYPRARRKEVVSATSSEEDGSSIAVTID FKGTLRIWDLVAGTHYCPDLDRNTALWSPALCATVDGRSVIIVGSRDGKVRVWDSAIEQQTESKPISVEKVTAIACTVANSNPVAVTLNNRATVQVLDLA TAERLRLDIRGHSGHLTAVACTTVDGQPIAVIGANDGTVQAWNLTTRQSHCEPLTESNDLILAVACTTIGGQPVAVIGGNDGTVCVWNLGTGQQRRMVVS EEAGQITAVACTDINGELVVVIGCANGSLWVGNLATGQQYRPDFAKGVLPIRTLACTTVNSHAVVVLSGNEDSVQCWDLTTRRHLGPNQSSRVLPVRTLA CATVDNHPIAAICSNDGTVRVWDLQRHKQLTQPIRILDPVDCLAIAPDLSLVIAYNTEVMAFNPPQRWDWKEDT&LINK_LOC=protein&PAGE_TYPE=BlastSearch', 'BLAST this protein','Snas_3598')" />
   LCHAGTAVRDLTHRQFGDRLWVAAAAGADRETWDGVFTQAFADTLMQIAKQRSVEGTADQHVFDPSAPYVSMAFVRSRVVENLVKLWPEKAASRAPDPWF HGAFSKGDGGVQGVPFFANPRFDPLARDAVTARQRISSGLHEYLDIAHFQDRVGTHFTGRRTLLTELGNWRADRDTNTPVRLIVGGAGVGKSSVLGVIVL TGHPHVAKRPEFHKQLAELRRRLPSEFSQEFTEPIAAVHARQLRTDDVVGSLMDQLYGQSGGPKAKPPDSTVGNFISWLRTTQHPPLLIVDAIDEAADSL DLIRRLIIPLTRTAVHFGAVRLLLALRTVDEQQRRLAKEIASIASAGDDRTIKLHDIDLSEPAELHRDLRRFLIETLETDDNRAWRQARVASVAQHVATQ LTRSPEGDWGAFLVAVLFSQYLLRQTEHDLDAALQTIPRTLPEVLELDLAMGNARRRQQRRAVLAALAHVKGAGMPSHLVHLLAQQVFGGGDNLDTATLL TDPQDLKVYLRTSVDTNNTTLFRLFHQALVDHLLAYPRRSAHEGLHT', 'http://blast.ncbi.nlm.nih.gov/Blast.cgi?PAGE=Proteins&PROGRAM=blastp&BLAST_PROGRAMS=blastp&QUERY=MATKRGESLARSLEALGSGYGVEHISEPTMAQLRRIDEFAFETKNLVRIIHVISHGEVDGQDQLHLVARNGFGYRTNMTSLCRSLADLAGEYGHILLVLD LCHAGTAVRDLTHRQFGDRLWVAAAAGADRETWDGVFTQAFADTLMQIAKQRSVEGTADQHVFDPSAPYVSMAFVRSRVVENLVKLWPEKAASRAPDPWF HGAFSKGDGGVQGVPFFANPRFDPLARDAVTARQRISSGLHEYLDIAHFQDRVGTHFTGRRTLLTELGNWRADRDTNTPVRLIVGGAGVGKSSVLGVIVL TGHPHVAKRPEFHKQLAELRRRLPSEFSQEFTEPIAAVHARQLRTDDVVGSLMDQLYGQSGGPKAKPPDSTVGNFISWLRTTQHPPLLIVDAIDEAADSL DLIRRLIIPLTRTAVHFGAVRLLLALRTVDEQQRRLAKEIASIASAGDDRTIKLHDIDLSEPAELHRDLRRFLIETLETDDNRAWRQARVASVAQHVATQ LTRSPEGDWGAFLVAVLFSQYLLRQTEHDLDAALQTIPRTLPEVLELDLAMGNARRRQQRRAVLAALAHVKGAGMPSHLVHLLAQQVFGGGDNLDTATLL TDPQDLKVYLRTSVDTNNTTLFRLFHQALVDHLLAYPRRSAHEGLHT&LINK_LOC=protein&PAGE_TYPE=BlastSearch', 'BLAST this protein','Snas_3599')" />

 
 
   QDHAAAAVVVGKYGTVEEPKGTPVFAWKGETLPEYWWCTEQMLRWPDGQGPNMILDDGGDATLLVHKGVAYEKTGVIPSINEDDPEYSEEYAVILDFLRA SLAADPQYWTRTAPSIIGVTEETTTGVNRLYQLAAAGELLFPAINVNDSVTKSKFDNKYGIRHSLIDGINRGTDVLIGGKVALVAGYGDVGKGAAESLRG QGARVVVAEIDPICALQALMDGFEVSTVDKAVERADLIITTTGNKDIITVAHMSRMKHQAILGNIGHFDNEIDMAGLARSGASKIEIKPQVDEWRFADGH TIIVLSAGRLLNLGNATGHPSFVMSTSFSNQVIAQLELFTKPNEWDKEVYRLPKHLDEKVAKIHVEALGGELTKLSKDQAEYLGVDVEGPFKPEHYRY', 'http://blast.ncbi.nlm.nih.gov/Blast.cgi?PAGE=Proteins&PROGRAM=blastp&BLAST_PROGRAMS=blastp&QUERY=MSTTTSTLSDLNLVADRSGDLDYKVASMALADFGRKEITLAEHEMPGLMALRREYAEVQPLAGARISGSLHMTVQTAVLIETLVELGADVRWASCNIFST QDHAAAAVVVGKYGTVEEPKGTPVFAWKGETLPEYWWCTEQMLRWPDGQGPNMILDDGGDATLLVHKGVAYEKTGVIPSINEDDPEYSEEYAVILDFLRA SLAADPQYWTRTAPSIIGVTEETTTGVNRLYQLAAAGELLFPAINVNDSVTKSKFDNKYGIRHSLIDGINRGTDVLIGGKVALVAGYGDVGKGAAESLRG QGARVVVAEIDPICALQALMDGFEVSTVDKAVERADLIITTTGNKDIITVAHMSRMKHQAILGNIGHFDNEIDMAGLARSGASKIEIKPQVDEWRFADGH TIIVLSAGRLLNLGNATGHPSFVMSTSFSNQVIAQLELFTKPNEWDKEVYRLPKHLDEKVAKIHVEALGGELTKLSKDQAEYLGVDVEGPFKPEHYRY&LINK_LOC=protein&PAGE_TYPE=BlastSearch', 'BLAST this protein','Namu_4257')" />

 
 
   FFNVGGVKGETKERLEAACARGSSSLYITGISPGWINTMVTAMTGVCRDVRKISILEAADCSVYESKETWEAMGISRTGTTPQIADMAKLWMMPAEEAVQ RMAHALEYTLDSVEFFLGYATASQTVDLGYMTIEKGTNAAVEVGWNGNLGGSTVIQMKFRWYLTKNLNEDWEFNDDQYRVVIDGEPGIDTRIRFIPPEHW GNHEWDTMTALPAVNAAFNVKAARPGILHLTDVGLPRAPAGQWHAARE', 'http://blast.ncbi.nlm.nih.gov/Blast.cgi?PAGE=Proteins&PROGRAM=blastp&BLAST_PROGRAMS=blastp&QUERY=MERLRVIQWTTGKVGKLTLRAILDDPRLDLVGVYAWSADKVGTDAGTLCGRPPCGVAATNDIDALIALKADTVVYTPFMADLDHVLPLLEAGMDVISTNL FFNVGGVKGETKERLEAACARGSSSLYITGISPGWINTMVTAMTGVCRDVRKISILEAADCSVYESKETWEAMGISRTGTTPQIADMAKLWMMPAEEAVQ RMAHALEYTLDSVEFFLGYATASQTVDLGYMTIEKGTNAAVEVGWNGNLGGSTVIQMKFRWYLTKNLNEDWEFNDDQYRVVIDGEPGIDTRIRFIPPEHW GNHEWDTMTALPAVNAAFNVKAARPGILHLTDVGLPRAPAGQWHAARE&LINK_LOC=protein&PAGE_TYPE=BlastSearch', 'BLAST this protein','Franean1_4269')" />

 
 
   PGRYRAKSQAGGHQVFDMVPVLPQGWTGEVTGAAVVPENECDTRTYVGLFPSPAIEGTPVACDIVDGSGTYTVRGVPAGSWYVRAATVRTGRVDPRPWAR QPRFLGPGEKVTVGPDEGTVKLDILLRAATILDLPILMALPELDNAGTSPAGALVAVGGQADAVS', 'http://blast.ncbi.nlm.nih.gov/Blast.cgi?PAGE=Proteins&PROGRAM=blastp&BLAST_PROGRAMS=blastp&QUERY=MDLRQLAIEQAIATMWNRYPEPLSLDEIADSAIMSKFHFSRVFRTMTGTSPGRFLSAIRVFKAKHLLLQTRMSVTEIAYQVGYNSLGTFTSRFSGSVGAS PGRYRAKSQAGGHQVFDMVPVLPQGWTGEVTGAAVVPENECDTRTYVGLFPSPAIEGTPVACDIVDGSGTYTVRGVPAGSWYVRAATVRTGRVDPRPWAR QPRFLGPGEKVTVGPDEGTVKLDILLRAATILDLPILMALPELDNAGTSPAGALVAVGGQADAVS&LINK_LOC=protein&PAGE_TYPE=BlastSearch', 'BLAST this protein','Caci_6355')" />
   LVNSNLSVTEVCMAVGYSSLGTFISQFTRLTGMSPRRFRSAMSVFGQVRLSELTDPAADRHCAPGPVGAVTGGPSGGGCAVLALYRSEEPDDVPLTYGVL ETNRLSRIRPLADGAYHAVALGFDKTSTLSDVLGDNGRQVEFVGTGGAPIVVRGGRTFRTFQVALRRPRPIDPPLQLTSSVLALAQRRRPQLLATGS', 'http://blast.ncbi.nlm.nih.gov/Blast.cgi?PAGE=Proteins&PROGRAM=blastp&BLAST_PROGRAMS=blastp&QUERY=MTTADQAWRQGGNCMDCAQFMTCSTLGVCLGNPTDAARLDSVTRAVEYMTEYLSEPQRLSDIAQAALLSPFHFHRVFRHVTSTTPARFLTALRMAQARNL LVNSNLSVTEVCMAVGYSSLGTFISQFTRLTGMSPRRFRSAMSVFGQVRLSELTDPAADRHCAPGPVGAVTGGPSGGGCAVLALYRSEEPDDVPLTYGVL ETNRLSRIRPLADGAYHAVALGFDKTSTLSDVLGDNGRQVEFVGTGGAPIVVRGGRTFRTFQVALRRPRPIDPPLQLTSSVLALAQRRRPQLLATGS&LINK_LOC=protein&PAGE_TYPE=BlastSearch', 'BLAST this protein','Micau_2516')" />
   LVNSNLSVTEVCMAVGYSSLGTFISQFTRLTGMSPRRFRSAMSVFGQVRLSELTDPAADRHCAPGPVGAVTGGPSGGGCAVLALYRSEEPDDVPLTYGVL ETNRLSRIRPLADGAYHAVALGFDKTSTLSDVLGDNGRQVEFVGTGGAPIVVRGGRTFRTFQVALRRPRPIDPPLQLTSSVLALAQRRRPQLLATGS', 'http://blast.ncbi.nlm.nih.gov/Blast.cgi?PAGE=Proteins&PROGRAM=blastp&BLAST_PROGRAMS=blastp&QUERY=MTTADQAWRQGGNCMDCAQFMTCSTLGVCLGNPTDAARLDSVTRAVEYMTEYLSEPQRLSDIAQAALLSPFHFHRVFRHVTSTTPARFLTALRMAQARNL LVNSNLSVTEVCMAVGYSSLGTFISQFTRLTGMSPRRFRSAMSVFGQVRLSELTDPAADRHCAPGPVGAVTGGPSGGGCAVLALYRSEEPDDVPLTYGVL ETNRLSRIRPLADGAYHAVALGFDKTSTLSDVLGDNGRQVEFVGTGGAPIVVRGGRTFRTFQVALRRPRPIDPPLQLTSSVLALAQRRRPQLLATGS&LINK_LOC=protein&PAGE_TYPE=BlastSearch', 'BLAST this protein','ML5_5847')" />
   SPTAYRRCGGIVHAMNPDVDEYHDRAEGVVAGTVSVQPPHRLDTVFLGLFRGRIPEGRPVRCVMEQGTGRFLLSKVPDGVWYLLCQGGASEPGSKDTVRF VGCYGPFEIRHGNVIDAQVQLKPVRTLDPPLLVDLPSARRSNSEGDGRWRPNRTGGLPLAA', 'http://blast.ncbi.nlm.nih.gov/Blast.cgi?PAGE=Proteins&PROGRAM=blastp&BLAST_PROGRAMS=blastp&QUERY=MGSHFEAAVERAIEMMRNNLGEQLTVDDMARAAMFSKFHFTRIFQRVTGVTPGRFLSALRLQRAKVLLLSTSMNVADISVQVGYNSIGTFSSRFTRSVGV SPTAYRRCGGIVHAMNPDVDEYHDRAEGVVAGTVSVQPPHRLDTVFLGLFRGRIPEGRPVRCVMEQGTGRFLLSKVPDGVWYLLCQGGASEPGSKDTVRF VGCYGPFEIRHGNVIDAQVQLKPVRTLDPPLLVDLPSARRSNSEGDGRWRPNRTGGLPLAA&LINK_LOC=protein&PAGE_TYPE=BlastSearch', 'BLAST this protein','Sare_2090')" />
   SPSKYRQLETVMPQLLTDDHRVLAGTTTMTVRGDISSPLKDRPVFAGLFPDRILEGRPIRYTILHQPGPYVLEDVPEGQWHLIAQSAAAGSEDAIDHPPG GDEALCIGRHGPITVQAGKSTEPADVQLKPMRTLDPPVLLALRDLVTATAARHTA', 'http://blast.ncbi.nlm.nih.gov/Blast.cgi?PAGE=Proteins&PROGRAM=blastp&BLAST_PROGRAMS=blastp&QUERY=MNDVIEQAVLRVVESMHDNLGDQITIDDMARTAMFSKFHFSRVFQRVTGLSPGRFLSAVRLREAKRLLTSTSLTVTDISHRVGYSSVGTFSSRFTSSVGV SPSKYRQLETVMPQLLTDDHRVLAGTTTMTVRGDISSPLKDRPVFAGLFPDRILEGRPIRYTILHQPGPYVLEDVPEGQWHLIAQSAAAGSEDAIDHPPG GDEALCIGRHGPITVQAGKSTEPADVQLKPMRTLDPPVLLALRDLVTATAARHTA&LINK_LOC=protein&PAGE_TYPE=BlastSearch', 'BLAST this protein','Sros_6995')" />
   LVNSNLSVTEVCLGVGYSSLGTFISQFTKLTGMSPRRFRAAMSVFGHIRLSELTDPSAERHFAPGPVGAVTGGPSAGGCAVLALYRSEEPDDVPVTYGVL ETNRLSHFRPLADGAYHAVALGFDNRSTLSDVLGDNGQQAQFVGTGDVPIVVRGGRTRRSFQIALRCPRPIDPPVQLTSALLALAQRRQPRLLATGS', 'http://blast.ncbi.nlm.nih.gov/Blast.cgi?PAGE=Proteins&PROGRAM=blastp&BLAST_PROGRAMS=blastp&QUERY=MTTADQLWRQRGNCLECAEVTRCSTLGMCLDNPTDAARLDSVTRAVEYMTAHFSEPQRLSDIAQAALLSPFHFHRVFRHVTSTTPARFLTALRMARARSM LVNSNLSVTEVCLGVGYSSLGTFISQFTKLTGMSPRRFRAAMSVFGHIRLSELTDPSAERHFAPGPVGAVTGGPSAGGCAVLALYRSEEPDDVPVTYGVL ETNRLSHFRPLADGAYHAVALGFDNRSTLSDVLGDNGQQAQFVGTGDVPIVVRGGRTRRSFQIALRCPRPIDPPVQLTSALLALAQRRQPRLLATGS&LINK_LOC=protein&PAGE_TYPE=BlastSearch', 'BLAST this protein','Strop_0600')" />
   SPTTYRRLGGFTPQVPVMDHAGPGSATGSVSGAIRSSASHGSTLIFAGLFPDRIPEGRPVRCTILRGPGRLRLDRVPEGEWYLLAHSVAGDDPEDALAKP EPACVGSVGPLVVRRGVVTHSRDLRLSPTRSIDPPVLLALLDIRRAAMHGQHGPELAGLAA', 'http://blast.ncbi.nlm.nih.gov/Blast.cgi?PAGE=Proteins&PROGRAM=blastp&BLAST_PROGRAMS=blastp&QUERY=MSHTVEKVAERVIALMHDDLSRQLTIDDMARTAMFSKFHFSRIFQKATGVTPARFLAALRLQRAKHLLVSTSMTVADISIQVGYTSVGTFSYRFSRSVGL SPTTYRRLGGFTPQVPVMDHAGPGSATGSVSGAIRSSASHGSTLIFAGLFPDRIPEGRPVRCTILRGPGRLRLDRVPEGEWYLLAHSVAGDDPEDALAKP EPACVGSVGPLVVRRGVVTHSRDLRLSPTRSIDPPVLLALLDIRRAAMHGQHGPELAGLAA&LINK_LOC=protein&PAGE_TYPE=BlastSearch', 'BLAST this protein','Strop_2693')" />

 
 
   YFMMRVRCDGGALSAAALRTLGQISTEFARDTADISDRQNVQYHWIEVENVPEIWRRLDDVGLQTTEACGDCPRVVLGSPLAGESLDEVLDPTWAIEEIV RRYIGKPDFADLPRKYKTAISGLQDVAHEINDVAFIGVNHPEHGPGLDLWVGGGLSTNPMLAQRVGAWVPLGEVPEVWAAVTSVFRDYGYRRLRAKARLK FLIKDWGIAKFREVLETEYLKRPLIDGPAPEPVKHPIDHVGVQRLKNGLNAVGVAPIAGRVSGTILTAVADLMARAGSDRIRFTPYQKLVILDIPDALLD DLIAGLDALGLQSRPSHWRRNLMACSGIEFCKLSFAETRVRAQHLVPELERRLEDINSQLDVPITVNINGCPNSCARIQIADIGFKGQMIDDGHGGSVEG FQVHLGGHLGLDAGFGRKLRQHKVTSDELGDYIDRVVRNFVKHRSEGERFAQWVIRAEEDDLR', 'http://blast.ncbi.nlm.nih.gov/Blast.cgi?PAGE=Proteins&PROGRAM=blastp&BLAST_PROGRAMS=blastp&QUERY=MSAKENPQMTTARPAKARNEGQWALGHREPLNANEELKKAGNPLDVRERIENIYAKQGFDSIDKTDLRGRFRWWGLYTQREQGYDGTWTGDDNIDKLEAK YFMMRVRCDGGALSAAALRTLGQISTEFARDTADISDRQNVQYHWIEVENVPEIWRRLDDVGLQTTEACGDCPRVVLGSPLAGESLDEVLDPTWAIEEIV RRYIGKPDFADLPRKYKTAISGLQDVAHEINDVAFIGVNHPEHGPGLDLWVGGGLSTNPMLAQRVGAWVPLGEVPEVWAAVTSVFRDYGYRRLRAKARLK FLIKDWGIAKFREVLETEYLKRPLIDGPAPEPVKHPIDHVGVQRLKNGLNAVGVAPIAGRVSGTILTAVADLMARAGSDRIRFTPYQKLVILDIPDALLD DLIAGLDALGLQSRPSHWRRNLMACSGIEFCKLSFAETRVRAQHLVPELERRLEDINSQLDVPITVNINGCPNSCARIQIADIGFKGQMIDDGHGGSVEG FQVHLGGHLGLDAGFGRKLRQHKVTSDELGDYIDRVVRNFVKHRSEGERFAQWVIRAEEDDLR&LINK_LOC=protein&PAGE_TYPE=BlastSearch', 'BLAST this protein','MCAN_24231')" />
   MLRVRSDGGALTAAALRTLGTISTEFARDTADISDRQNVQYHWIDVKDMPEIWRRLDEVGLQTTEACGDCPRVVLGSPLAGESLDEVIDGTPAVNEIVKR YIGKKEYSNLPRKFKTAISGLQDVVHEVNDVAFIGVNHPEHGPGFDLWVGGGLSTNPMLGQRVGAWVPLDEVPDVWEGVVSVFRDYGYRRLRAKARLKFL IKDWGVEKFRQVLEEEYLKRPLIDGPAPEPVTRPIDHVGVQKLKNGLNAVGVAPIAGRVSGTILTKVADLAEAAGSDRIRFTPYQKLIVLDVPDDKLDEL RAGLDALGLPSTPSHWRRNLMACTGIEFCKLSFAETRSRSQVLVPELEKRLEDINAQLDVPVTININGCPNSCARIQVADIGFKGQMVDDGDGPEEGFQV HLGGSLGLDSGFGRKLRQHKVVATELGDYIERVVRNFVKQREQGERFATWALRADDADLR', 'http://blast.ncbi.nlm.nih.gov/Blast.cgi?PAGE=Proteins&PROGRAM=blastp&BLAST_PROGRAMS=blastp&QUERY=MTTAKTPPAKPAKRPRGEGQWALGYREPLNPNEQSKKDDNPLNVRERIENIYAKNGFESIDKGDLRGRFRWWGLYTQRKPGYDGTWTGDENTDMLEDEYF MLRVRSDGGALTAAALRTLGTISTEFARDTADISDRQNVQYHWIDVKDMPEIWRRLDEVGLQTTEACGDCPRVVLGSPLAGESLDEVIDGTPAVNEIVKR YIGKKEYSNLPRKFKTAISGLQDVVHEVNDVAFIGVNHPEHGPGFDLWVGGGLSTNPMLGQRVGAWVPLDEVPDVWEGVVSVFRDYGYRRLRAKARLKFL IKDWGVEKFRQVLEEEYLKRPLIDGPAPEPVTRPIDHVGVQKLKNGLNAVGVAPIAGRVSGTILTKVADLAEAAGSDRIRFTPYQKLIVLDVPDDKLDEL RAGLDALGLPSTPSHWRRNLMACTGIEFCKLSFAETRSRSQVLVPELEKRLEDINAQLDVPVTININGCPNSCARIQVADIGFKGQMVDDGDGPEEGFQV HLGGSLGLDSGFGRKLRQHKVVATELGDYIERVVRNFVKQREQGERFATWALRADDADLR&LINK_LOC=protein&PAGE_TYPE=BlastSearch', 'BLAST this protein','Mflv_2686')" />
   MLRVRSDGGALTAKALRTLGEISTEFARDTADISDRQNVQYHWIDVRNMPEIWRRLDEVGLQTTEACGDCPRVVLGSPLAGESLDEVIDGTPAVDEIVKR YIGKPEYSNLPRKFKTAISGLQDVVHEVNDVAFIGVVHPEHGPGFDLWVGGGLSTNPMLGQRVGAWVPLDEVPDVWEGVVSVFRDYGYRRLRSKARLKFL IKDWGVEKFRQVLETEYLKRPLIDGPAPDPVTRPIDHVGVQKLKNGLNAVGVAPIAGRVSGTILSKVADLAEAAGSDRIRFTPYQKLIVLDVPDEKLDEL RAGLDALGLPSTPSHWRRNLMACTGIEFCKLSFAETRSRSQVLVPELEKRLEDINAQLDVPVTININGCPNSCARIQVADIGFKGQMVDDGDGPEEGFQV HLGGSLGLDSGFGRKLRQHKVLATELGDYIERVVRNFVKQREDGERFATWALRADEADLR', 'http://blast.ncbi.nlm.nih.gov/Blast.cgi?PAGE=Proteins&PROGRAM=blastp&BLAST_PROGRAMS=blastp&QUERY=MTETPTRPAKPVKRPRGEGQWALGYREPLNANEQSKKDDNPLNVRERIENIYAPGGFDTIDKGDLRGRFRWWGLYTQRKPGFDGTWTGDENTDMLEDEYF MLRVRSDGGALTAKALRTLGEISTEFARDTADISDRQNVQYHWIDVRNMPEIWRRLDEVGLQTTEACGDCPRVVLGSPLAGESLDEVIDGTPAVDEIVKR YIGKPEYSNLPRKFKTAISGLQDVVHEVNDVAFIGVVHPEHGPGFDLWVGGGLSTNPMLGQRVGAWVPLDEVPDVWEGVVSVFRDYGYRRLRSKARLKFL IKDWGVEKFRQVLETEYLKRPLIDGPAPDPVTRPIDHVGVQKLKNGLNAVGVAPIAGRVSGTILSKVADLAEAAGSDRIRFTPYQKLIVLDVPDEKLDEL RAGLDALGLPSTPSHWRRNLMACTGIEFCKLSFAETRSRSQVLVPELEKRLEDINAQLDVPVTININGCPNSCARIQVADIGFKGQMVDDGDGPEEGFQV HLGGSLGLDSGFGRKLRQHKVLATELGDYIERVVRNFVKQREDGERFATWALRADEADLR&LINK_LOC=protein&PAGE_TYPE=BlastSearch', 'BLAST this protein','Mjls_3487')" />
   MLRVRSDGGALTAKALRTLGEISTEFARDTADISDRQNVQYHWIDVRNMPEIWRRLDEVGLQTTEACGDCPRVVLGSPLAGESLDEVIDGTPAVNEIVKR YIGKPEYSNLPRKFKTAISGLQDVVHEVNDVAFIGVVHPEHGPGFDLWVGGGLSTNPMLGQRVGAWVPLDEVPDVWEGVVSVFRDYGYRRLRSKARLKFL IKDWGVEKFRQVLETEYLKRPLIDGPAPDPVTRPIDHVGVQKLKNGLNAVGVAPIAGRVSGTILSKVADLAEAAGSDRIRFTPYQKLIVLDVPDEKLEEL RAGLDALGLPSTPSHWRRNLMACTGIEFCKLSFAETRSRSQVLVPELEKRLEDINAQLDVPVTININGCPNSCARIQVADIGFKGQMVDDGDGPQEGFQV HLGGSLGLDSGFGRKLRQHKVLATELGDYIERVVRNFVKQREDGERFATWALRADEADLR', 'http://blast.ncbi.nlm.nih.gov/Blast.cgi?PAGE=Proteins&PROGRAM=blastp&BLAST_PROGRAMS=blastp&QUERY=MTETPTRPAKPVKRPRGEGQWALGYREPLNANEQSKKDDNPLNVRERIENIYAPGGFDTIDKGDLRGRFRWWGLYTQRKPGFDGTWTGDENTDMLEDEYF MLRVRSDGGALTAKALRTLGEISTEFARDTADISDRQNVQYHWIDVRNMPEIWRRLDEVGLQTTEACGDCPRVVLGSPLAGESLDEVIDGTPAVNEIVKR YIGKPEYSNLPRKFKTAISGLQDVVHEVNDVAFIGVVHPEHGPGFDLWVGGGLSTNPMLGQRVGAWVPLDEVPDVWEGVVSVFRDYGYRRLRSKARLKFL IKDWGVEKFRQVLETEYLKRPLIDGPAPDPVTRPIDHVGVQKLKNGLNAVGVAPIAGRVSGTILSKVADLAEAAGSDRIRFTPYQKLIVLDVPDEKLEEL RAGLDALGLPSTPSHWRRNLMACTGIEFCKLSFAETRSRSQVLVPELEKRLEDINAQLDVPVTININGCPNSCARIQVADIGFKGQMVDDGDGPQEGFQV HLGGSLGLDSGFGRKLRQHKVLATELGDYIERVVRNFVKQREDGERFATWALRADEADLR&LINK_LOC=protein&PAGE_TYPE=BlastSearch', 'BLAST this protein','Mkms_3537')" />
   MLRVRSDGGALTAKALRTLGEISTEFARDTADISDRQNVQYHWIDVRNMPEIWRRLDEVGLQTTEACGDCPRVVLGSPLAGESLDEVIDGTPAVNEIVKR YIGKPEYSNLPRKFKTAISGLQDVVHEVNDVAFIGVVHPEHGPGFDLWVGGGLSTNPMLGQRVGAWVPLDEVPDVWEGVVSVFRDYGYRRLRSKARLKFL IKDWGVEKFRQVLETEYLKRPLIDGPAPDPVTRPIDHVGVQKLKNGLNAVGVAPIAGRVSGTILSKVADLAEAAGSDRIRFTPYQKLIVLDVPDEKLEEL RAGLDALGLPSTPSHWRRNLMACTGIEFCKLSFAETRSRSQVLVPELEKRLEDINAQLDVPVTININGCPNSCARIQVADIGFKGQMVDDGDGPQEGFQV HLGGSLGLDSGFGRKLRQHKVLATELGDYIERVVRNFVKQREDGERFATWALRADEADLR', 'http://blast.ncbi.nlm.nih.gov/Blast.cgi?PAGE=Proteins&PROGRAM=blastp&BLAST_PROGRAMS=blastp&QUERY=MTETPTRPAKPVKRPRGEGQWALGYREPLNANEQSKKDDNPLNVRERIENIYAPGGFDTIDKGDLRGRFRWWGLYTQRKPGFDGTWTGDENTDMLEDEYF MLRVRSDGGALTAKALRTLGEISTEFARDTADISDRQNVQYHWIDVRNMPEIWRRLDEVGLQTTEACGDCPRVVLGSPLAGESLDEVIDGTPAVNEIVKR YIGKPEYSNLPRKFKTAISGLQDVVHEVNDVAFIGVVHPEHGPGFDLWVGGGLSTNPMLGQRVGAWVPLDEVPDVWEGVVSVFRDYGYRRLRSKARLKFL IKDWGVEKFRQVLETEYLKRPLIDGPAPDPVTRPIDHVGVQKLKNGLNAVGVAPIAGRVSGTILSKVADLAEAAGSDRIRFTPYQKLIVLDVPDEKLEEL RAGLDALGLPSTPSHWRRNLMACTGIEFCKLSFAETRSRSQVLVPELEKRLEDINAQLDVPVTININGCPNSCARIQVADIGFKGQMVDDGDGPQEGFQV HLGGSLGLDSGFGRKLRQHKVLATELGDYIERVVRNFVKQREDGERFATWALRADEADLR&LINK_LOC=protein&PAGE_TYPE=BlastSearch', 'BLAST this protein','Mmcs_3474')" />
   MLRVRSDGGALTAAALRTLGTISTEFARDTADISDRQNVQYHWIDVKDMPEIWRRLDEVGLQTTEACGDCPRVVLGSPLAGESLDEVIDGTPAVNEIVKR YIGKKEYSNLPRKFKTAISGLQDVVHEVNDVAFIGVNHPEHGPGFDLWVGGGLSTNPMLGQRVGAWVPLDEVPDVWEGVVSVFRDYGYRRLRAKARLKFL IKDWGVEKFRQVLEEEYLKRPLIDGPAPEPVTRPIDHVGVQKLKNGLNAVGVAPIAGRVSGTILTKVADLAEAAGSDRIRFTPYQKLIVLDVPDDKLDEL RAGLDALGLPSTPSHWRRNLMACTGIEFCKLSFAETRSRSQVLVPELEKRLEDINAQLDVPVTININGCPNSCARIQVADIGFKGQMVDDGDGPEEGFQV HLGGSLGLDSGFGRKLRQHKVVATELGDYIERVVRNFVKQREQGERFATWALRADDADLR', 'http://blast.ncbi.nlm.nih.gov/Blast.cgi?PAGE=Proteins&PROGRAM=blastp&BLAST_PROGRAMS=blastp&QUERY=MTTAKTPPAKPAKRPRGEGQWALGYREPLNPNEQSKKDDNPLNVRERIENIYAKNGFESIDKGDLRGRFRWWGLYTQRKPGYDGTWTGDENTDMLEDEYF MLRVRSDGGALTAAALRTLGTISTEFARDTADISDRQNVQYHWIDVKDMPEIWRRLDEVGLQTTEACGDCPRVVLGSPLAGESLDEVIDGTPAVNEIVKR YIGKKEYSNLPRKFKTAISGLQDVVHEVNDVAFIGVNHPEHGPGFDLWVGGGLSTNPMLGQRVGAWVPLDEVPDVWEGVVSVFRDYGYRRLRAKARLKFL IKDWGVEKFRQVLEEEYLKRPLIDGPAPEPVTRPIDHVGVQKLKNGLNAVGVAPIAGRVSGTILTKVADLAEAAGSDRIRFTPYQKLIVLDVPDDKLDEL RAGLDALGLPSTPSHWRRNLMACTGIEFCKLSFAETRSRSQVLVPELEKRLEDINAQLDVPVTININGCPNSCARIQVADIGFKGQMVDDGDGPEEGFQV HLGGSLGLDSGFGRKLRQHKVVATELGDYIERVVRNFVKQREQGERFATWALRADDADLR&LINK_LOC=protein&PAGE_TYPE=BlastSearch', 'BLAST this protein','Mspyr1_21230')" />

 
 
   

 
 
   TEEARHADTLGLKPWLPTLKYNFVRIDVREVSGRAFVFGEEPERY', 'http://blast.ncbi.nlm.nih.gov/Blast.cgi?PAGE=Proteins&PROGRAM=blastp&BLAST_PROGRAMS=blastp&QUERY=MMGIMSDPITILDSSDSLSRLSSESVGRLVVHRKDDLDIFPVNFVLDYSAEQPRVYFRTAEGTKLFSVNLNSDVLFEVDRFDDAEGWSVVLKGNAYVVRD TEEARHADTLGLKPWLPTLKYNFVRIDVREVSGRAFVFGEEPERY&LINK_LOC=protein&PAGE_TYPE=BlastSearch', 'BLAST this protein','DIP_1874')" />

 
 
   LHSVREADGDEVQQGLERTVAAMTHAATADADVEAAEGQGGWTSYDDKRDSSS', 'http://blast.ncbi.nlm.nih.gov/Blast.cgi?PAGE=Proteins&PROGRAM=blastp&BLAST_PROGRAMS=blastp&QUERY=MTEYRHSATADIPADELFAFLSHPENLPRYFPEMKVAEPTGGDSVHVEAEVHGNRVASEAWLHTDPATRSLKWGAEGPDDYHGELRIRDDGPASSEIIVT LHSVREADGDEVQQGLERTVAAMTHAATADADVEAAEGQGGWTSYDDKRDSSS&LINK_LOC=protein&PAGE_TYPE=BlastSearch', 'BLAST this protein','AMED_4477')" />
   LHSVREADGDEVQQGLERTVAAMTHAATADADVEAAEGQGGWTSYDDKRDSSS', 'http://blast.ncbi.nlm.nih.gov/Blast.cgi?PAGE=Proteins&PROGRAM=blastp&BLAST_PROGRAMS=blastp&QUERY=MTEYRHSATADIPADELFAFLSHPENLPRYFPEMKVAEPTGGDSVHVEAEVHGNRVASEAWLHTDPATRSLKWGAEGPDDYHGELRIRDDGPASSEIIVT LHSVREADGDEVQQGLERTVAAMTHAATADADVEAAEGQGGWTSYDDKRDSSS&LINK_LOC=protein&PAGE_TYPE=BlastSearch', 'BLAST this protein','RAM_22800')" />

 
 
   PAVLGLGAAWVLGTQHAVAVLWIGVVALVAMLILVRNLYGLFSLTVVGALLFGLVWFGTQDQQVAAAYLITLFMLVAAPRPVLELQRQRARGAAPESDAD QLARLTRVPGIVWVGLSLLVTLSCLALGGWWILRPVGG', 'http://blast.ncbi.nlm.nih.gov/Blast.cgi?PAGE=Proteins&PROGRAM=blastp&BLAST_PROGRAMS=blastp&QUERY=MNGIHSLWERMSAVSPAPAPWIVQVTAVVAVILVLEPHAWRLTRNVVTIVHEGAHLVVALLFGRTLKGVRLHSDTSGVAISSGKPTGLGVVLMTFAGYVG PAVLGLGAAWVLGTQHAVAVLWIGVVALVAMLILVRNLYGLFSLTVVGALLFGLVWFGTQDQQVAAAYLITLFMLVAAPRPVLELQRQRARGAAPESDAD QLARLTRVPGIVWVGLSLLVTLSCLALGGWWILRPVGG&LINK_LOC=protein&PAGE_TYPE=BlastSearch', 'BLAST this protein','RHA1_ro02398')" />
   PALLGLGAAWVLGTQHAVAVLWIGVVALAAMLILVRNLYGLFSLTVVGALLFGLVWFGTQDQQVGAAYLITLFMLVAAPRPVLELQRQRSRGAAPHSDAD QLARLTRVPGIVWVGLSLLVTLGCLALGGWWILRPVGG', 'http://blast.ncbi.nlm.nih.gov/Blast.cgi?PAGE=Proteins&PROGRAM=blastp&BLAST_PROGRAMS=blastp&QUERY=MNGMHSLWERVSAVSPAPAPWIVQITAVVAVILVLEPHAWRITRNVVTIVHEGAHLVVALLFGRTLKGVRLHSDTSGVAISSGKPTGLGVVLMTFAGYVG PALLGLGAAWVLGTQHAVAVLWIGVVALAAMLILVRNLYGLFSLTVVGALLFGLVWFGTQDQQVGAAYLITLFMLVAAPRPVLELQRQRSRGAAPHSDAD QLARLTRVPGIVWVGLSLLVTLGCLALGGWWILRPVGG&LINK_LOC=protein&PAGE_TYPE=BlastSearch', 'BLAST this protein','ROP_21100')" />
   LGLGGAWLTSAGYVAILITIVIALLFCMLLLIRNLFGVVSLLATGGATAALTWYAPPDVDAVLAYLAVWFLLFGGVRPIVELQRKRRHGRAPSSDADQLA RLTFLPGGFWVLLFLLVAGAALLGGAYLLVPLPLAFH', 'http://blast.ncbi.nlm.nih.gov/Blast.cgi?PAGE=Proteins&PROGRAM=blastp&BLAST_PROGRAMS=blastp&QUERY=MSEVWVTLTTPQAGPPLWIVLLAALAALAVVTWSASWQLSRGLITIAHEGGHALTALLTRRKLQGIRLHSDTSGVTLTRGRPTGPGMILTAAAGYVAPSL LGLGGAWLTSAGYVAILITIVIALLFCMLLLIRNLFGVVSLLATGGATAALTWYAPPDVDAVLAYLAVWFLLFGGVRPIVELQRKRRHGRAPSSDADQLA RLTFLPGGFWVLLFLLVAGAALLGGAYLLVPLPLAFH&LINK_LOC=protein&PAGE_TYPE=BlastSearch', 'BLAST this protein','Sros_6431')" />

 
 
   VTGALAMAATDLPMAGLRVSDPTTWSAKDWVSDAVPHLVYGLVTYAVVTAGEDRS', 'http://blast.ncbi.nlm.nih.gov/Blast.cgi?PAGE=Proteins&PROGRAM=blastp&BLAST_PROGRAMS=blastp&QUERY=MIQNLVRGGVAGAAGTTVLNAVTYADMAWRGRGASDAPAQVVEKLAQAVGHPVTGSGDTRDNRLTGLGALSGISVGCGTGVAVSALRRVGVRLPWWLGGV VTGALAMAATDLPMAGLRVSDPTTWSAKDWVSDAVPHLVYGLVTYAVVTAGEDRS&LINK_LOC=protein&PAGE_TYPE=BlastSearch', 'BLAST this protein','SACTE_5058')" />
   AAMAAGDIPLVALRLTDPRRWEASSWVADVVPHLAYGLTAAAVYRRLTRP', 'http://blast.ncbi.nlm.nih.gov/Blast.cgi?PAGE=Proteins&PROGRAM=blastp&BLAST_PROGRAMS=blastp&QUERY=MSLGAGIVAGAAGTTALDAVTYLDMAVRGRPASRLPSEAAAELADRAGADLGSGQAGESRREGVGALLGYAAGLGAGALYGLLAGDRRLPLPVAALGLSA AAMAAGDIPLVALRLTDPRRWEASSWVADVVPHLAYGLTAAAVYRRLTRP&LINK_LOC=protein&PAGE_TYPE=BlastSearch', 'BLAST this protein','Sros_3596')" />

 
 
   SAAATWNSSESNVKLEEASSGADFSYYEGNDPRGSHASTDGHGSGQIFLDYTQAQQNDSIRVVTHETGHVLGLPDNYSGPCSELMSGGGPGPSCTNRYPN ATERARVDELWAGGGAQAPGTGSQPAGGPSAAPSAPSAPSVAPGTGNQPAGGPSAALPVRPVGPGQGPWRPHLRTLWN', 'http://blast.ncbi.nlm.nih.gov/Blast.cgi?PAGE=Proteins&PROGRAM=blastp&BLAST_PROGRAMS=blastp&QUERY=MKSSRTSPRFLALALGLGLASSALAVAVPASAQTVATPSDSADTSVAGYDGSAEEAANNKAFFEAVLKSVAEKRAAQPSGQAVTLHYDASKAPSFRSQIA SAAATWNSSESNVKLEEASSGADFSYYEGNDPRGSHASTDGHGSGQIFLDYTQAQQNDSIRVVTHETGHVLGLPDNYSGPCSELMSGGGPGPSCTNRYPN ATERARVDELWAGGGAQAPGTGSQPAGGPSAAPSAPSAPSVAPGTGNQPAGGPSAALPVRPVGPGQGPWRPHLRTLWN&LINK_LOC=protein&PAGE_TYPE=BlastSearch', 'BLAST this protein','SAV_2939')" />

 
 
   LEHGPLGLTNSMLSMNGEQHKRYRALVQPSFLPANGKWWIDNWISETVDLLIDGLVHEGRAELNVDFCAAIPVLTITGSFGVPVEQALDIREALARDPQK VVDLLKPVIAARPEEPRDDLISVLVQAELTDEDGAKDRLTDREIDSFVLLLLGAGSGTTWKQMGTTLTTLLQRPELLEAVRADRSLLRPAIEEAIRWMPT DPMFSRWVMADTELAGVSIPAGSVVHLALGAANRDPARWDRPDEYDITRKFKPSLGFGQGSHICLGMHVARAEMTIAISALLDRLPNLRLDPDAEPPRFV GMYERGATAIPVVFDV', 'http://blast.ncbi.nlm.nih.gov/Blast.cgi?PAGE=Proteins&PROGRAM=blastp&BLAST_PROGRAMS=blastp&QUERY=MTAPALDRDRLRELFDLRSSYNAWAGGAYEDDPYPVWHRLREKGPVLPGVLHELTGSTDTMFFHGLPYPDCPHFTVFDYDSCMIAYRNPEVFASSPEPVD LEHGPLGLTNSMLSMNGEQHKRYRALVQPSFLPANGKWWIDNWISETVDLLIDGLVHEGRAELNVDFCAAIPVLTITGSFGVPVEQALDIREALARDPQK VVDLLKPVIAARPEEPRDDLISVLVQAELTDEDGAKDRLTDREIDSFVLLLLGAGSGTTWKQMGTTLTTLLQRPELLEAVRADRSLLRPAIEEAIRWMPT DPMFSRWVMADTELAGVSIPAGSVVHLALGAANRDPARWDRPDEYDITRKFKPSLGFGQGSHICLGMHVARAEMTIAISALLDRLPNLRLDPDAEPPRFV GMYERGATAIPVVFDV&LINK_LOC=protein&PAGE_TYPE=BlastSearch', 'BLAST this protein','MAP_2183c')" />

 
 
   LYQALVRWNRYRTYVSDPGDWLNWISAVLACAGAGLLVQDRVPFLPAAWPSWMVQLWLLVFGSLLILLGTAVTVAWIGGLLRDRRVWLVVGALGALTVVS GTLRGDPADALHGQAAWTLAFLVIAFASTRGGRHAPVPATSQAPAIGALVVLALAVGVLVVDGHEAGWVATAYAGVAVLGVSVRVVHLVRELAQLAESRQ QALTDELTGVGNRRALLRVLGGLVADGRSAALLLLDVDRFKEVNDRQGHHAGDDLLRRVVAATRRALPADAVLTRTGGDEFAVVLPDRDEASAVEVARAV HAAVVADAEIGLSVGVRSLPAGGFDPDRLLRQADTAMYAAKTAGGGVSVYDVDVDARLRDRAALAADLKALVAAGEERLRREVVVHYQPQLDVGTGRVVG AEALVRWQHPQRGLLPPVAFLDLVEEQGLMDDLTEHLLHRASADAAGWSHRGSPLRISVNLSASSLTHPGLLGLVDDVLERSGLAAGRLVLEVTETTLMA DPDLALAVTRELTRRGVQLSIDDYGTGYSSLAYLTDLPATELKLDRAFTARVLAEPRTAEIVEATVALAHRLGLRVVAEGVEDESTRAALGGLDVDESQG YLHSRPLPPAAFAAWLAAAGAAQVVGAAGSTDAPSR', 'http://blast.ncbi.nlm.nih.gov/Blast.cgi?PAGE=Proteins&PROGRAM=blastp&BLAST_PROGRAMS=blastp&QUERY=MTFSQPRPRTRVAAVCLAVLAVGSAFAPVGATASAGLLAVAHLTAVAVLALGARRRADDGVWRSFAVAGALVGVPPVLALVTGGRSLTPLGGCLVVAMPF LYQALVRWNRYRTYVSDPGDWLNWISAVLACAGAGLLVQDRVPFLPAAWPSWMVQLWLLVFGSLLILLGTAVTVAWIGGLLRDRRVWLVVGALGALTVVS GTLRGDPADALHGQAAWTLAFLVIAFASTRGGRHAPVPATSQAPAIGALVVLALAVGVLVVDGHEAGWVATAYAGVAVLGVSVRVVHLVRELAQLAESRQ QALTDELTGVGNRRALLRVLGGLVADGRSAALLLLDVDRFKEVNDRQGHHAGDDLLRRVVAATRRALPADAVLTRTGGDEFAVVLPDRDEASAVEVARAV HAAVVADAEIGLSVGVRSLPAGGFDPDRLLRQADTAMYAAKTAGGGVSVYDVDVDARLRDRAALAADLKALVAAGEERLRREVVVHYQPQLDVGTGRVVG AEALVRWQHPQRGLLPPVAFLDLVEEQGLMDDLTEHLLHRASADAAGWSHRGSPLRISVNLSASSLTHPGLLGLVDDVLERSGLAAGRLVLEVTETTLMA DPDLALAVTRELTRRGVQLSIDDYGTGYSSLAYLTDLPATELKLDRAFTARVLAEPRTAEIVEATVALAHRLGLRVVAEGVEDESTRAALGGLDVDESQG YLHSRPLPPAAFAAWLAAAGAAQVVGAAGSTDAPSR&LINK_LOC=protein&PAGE_TYPE=BlastSearch', 'BLAST this protein','Krad_0088')" />

 
 
   VKRRTDVVQVFPNDDALLRLVTAVLFELHDEWIAFPRRYLPEGSMSQLYPAELPEQAPALAHTTSTTAG', 'http://blast.ncbi.nlm.nih.gov/Blast.cgi?PAGE=Proteins&PROGRAM=blastp&BLAST_PROGRAMS=blastp&QUERY=MLGAAYQRCRVHFLRNVFSLINKEAAEMAAATIRTIFAQPTADAVRTQLDTVAGMLGKQFPKVKAMLLEAKDDLTAFAAFPERHGKKIQSTNPLERINRE VKRRTDVVQVFPNDDALLRLVTAVLFELHDEWIAFPRRYLPEGSMSQLYPAELPEQAPALAHTTSTTAG&LINK_LOC=protein&PAGE_TYPE=BlastSearch', 'BLAST this protein','SAV_104')" />

 
 
   GPIAPCYHAAVDAQTGAFDLLLGDAGPALVGDEIAGATVAQAQLAVSQLGRLHGPLLGDAALAEAPWLNRDSPLNQAMVNGLYAGFLDRYGEQIDPRSRM VCERLVAAFDGYLAQESSRGAIQGLVHGDYRLDNLLFGAAGAERPLTVVDWQTVSWGPALTDLAYFLGCALQPNERRAHYDDLLRTYHDALGPQAPITLE QVADEVRRQSFFGVMMAIVSSMLVERTDRGDRLFMTMLHRHCEHVLDTDALATLPDAAPPEPLCPGEQDEMAHEPTDEPLWSESWYADFVDAAQGLGGWF RLGRIANQQKAWVHALLCGPDMATVAVADIEVALPDDPWAVRTGDIELRHHATAPLQTYAVQLRARGQAYEDPSALLRGEPGDPVEVTMNLVWTTNGVPY RYRLTSRYEIPCTVAGTVTVDGTHYRVDAVPGQRDHSWGVRDWWSMDWMWSALHLDDGTHLHGVNIQLPGTPAFSVGYVQDSGGNLTELQAVNFREAFGS NGLPQNTTLILKPGDITANIDVRAHAPVRLNASDWRVSHFPRAWVAVVTTDGRSGVGWMEWNRNQV', 'http://blast.ncbi.nlm.nih.gov/Blast.cgi?PAGE=Proteins&PROGRAM=blastp&BLAST_PROGRAMS=blastp&QUERY=MNDEGDQNAGIIEEPGDLTASWLTEKIGAGDVTEFAVERIGTGQMSECYRVQLTYAVPGGPSLVVLKVAATDPMSRQTGQALGLYEREVRFYAEVAPRLG GPIAPCYHAAVDAQTGAFDLLLGDAGPALVGDEIAGATVAQAQLAVSQLGRLHGPLLGDAALAEAPWLNRDSPLNQAMVNGLYAGFLDRYGEQIDPRSRM VCERLVAAFDGYLAQESSRGAIQGLVHGDYRLDNLLFGAAGAERPLTVVDWQTVSWGPALTDLAYFLGCALQPNERRAHYDDLLRTYHDALGPQAPITLE QVADEVRRQSFFGVMMAIVSSMLVERTDRGDRLFMTMLHRHCEHVLDTDALATLPDAAPPEPLCPGEQDEMAHEPTDEPLWSESWYADFVDAAQGLGGWF RLGRIANQQKAWVHALLCGPDMATVAVADIEVALPDDPWAVRTGDIELRHHATAPLQTYAVQLRARGQAYEDPSALLRGEPGDPVEVTMNLVWTTNGVPY RYRLTSRYEIPCTVAGTVTVDGTHYRVDAVPGQRDHSWGVRDWWSMDWMWSALHLDDGTHLHGVNIQLPGTPAFSVGYVQDSGGNLTELQAVNFREAFGS NGLPQNTTLILKPGDITANIDVRAHAPVRLNASDWRVSHFPRAWVAVVTTDGRSGVGWMEWNRNQV&LINK_LOC=protein&PAGE_TYPE=BlastSearch', 'BLAST this protein','MMAR_3019')" />

 
 
   AAANMRLAQERIPATFVTDRSDKIKDMMREVFNRPVPERPGKYLDEKLPCVSCRSFFVIRAILHAWRNNIPYIILCADPQQTLTMESNVRKIVKNFYQIF GRDRTGEFFSGGLEELLFADERELPKIVFPFVALRDEYDPDRIVAELKAKGIYESSPFETHCTLFPLLNYYSFRNWGCMFYKLNASSYVRAVNRNKDYDR STYSVKFPRSVDVAEVEDRLRAIVFEIAAGEGDPGAHEEALVEVFKQLDAGEDAARYVARSFLDLRTVAADMGIRLD', 'http://blast.ncbi.nlm.nih.gov/Blast.cgi?PAGE=Proteins&PROGRAM=blastp&BLAST_PROGRAMS=blastp&QUERY=MVRCKICSLNAGHPGISLDNDGVCNLCSLDVAVEMFENFTYVSRTYEEFANSGPNERGPYDCLLMYSGGKDSTYMLDKFVNEYHKRVVSYTFEIPFESSH AAANMRLAQERIPATFVTDRSDKIKDMMREVFNRPVPERPGKYLDEKLPCVSCRSFFVIRAILHAWRNNIPYIILCADPQQTLTMESNVRKIVKNFYQIF GRDRTGEFFSGGLEELLFADERELPKIVFPFVALRDEYDPDRIVAELKAKGIYESSPFETHCTLFPLLNYYSFRNWGCMFYKLNASSYVRAVNRNKDYDR STYSVKFPRSVDVAEVEDRLRAIVFEIAAGEGDPGAHEEALVEVFKQLDAGEDAARYVARSFLDLRTVAADMGIRLD&LINK_LOC=protein&PAGE_TYPE=BlastSearch', 'BLAST this protein','Francci3_1977')" />

 
 
   ESVRVVTQYDVREDPRPAALARYGHWVGQWRTSVDRCRSHAQAVVDQANQRLACYWDAVRETHPQLSRLPRRPPGDWLPGRVELDRSWQQPDVWLLADDD SARTATSRALQILERQNTDRVDGRTAR', 'http://blast.ncbi.nlm.nih.gov/Blast.cgi?PAGE=Proteins&PROGRAM=blastp&BLAST_PROGRAMS=blastp&QUERY=MITSDSVNGVVRRGRLGRTARFAARLRGKRDGARGVPRIPLPEPPEEQRAELPPPIEPPAPELLITPYVMEVRTGVRRATEQMRSALIGREHALLSRVRA ESVRVVTQYDVREDPRPAALARYGHWVGQWRTSVDRCRSHAQAVVDQANQRLACYWDAVRETHPQLSRLPRRPPGDWLPGRVELDRSWQQPDVWLLADDD SARTATSRALQILERQNTDRVDGRTAR&LINK_LOC=protein&PAGE_TYPE=BlastSearch', 'BLAST this protein','Strvi_5361')" />

 
 
   
   

 
 
   TTDGESHELDSMVSLEIAGGPEAMSPRLARRTSAGRLATSIAHELRLSNVEVGSIATACSAGNYAIGHSFDAIRLGEADFALCGGADAVCRKTFTSFYRL RSIAPDFCRPFDKNRLGILTGEGAGILMLESLESATARGARIYGEVLGYGLSCDAHHPVAPHQGGIARCMELALANAGVKPAEVDLISAHGTGTHANDKT EAAAIRELFGEDTPRVVSMKSMLGHSMGAASALAAIGCSVAITEGFIPPTINHVETDPECAVDCVPNVAVEADLRIVQNNGWAFGGNNSVVLIGRYDGPR EVVA', 'http://blast.ncbi.nlm.nih.gov/Blast.cgi?PAGE=Proteins&PROGRAM=blastp&BLAST_PROGRAMS=blastp&QUERY=MITGCGVVSSLGTGIEEFTAALRAGRSNVRPITVFDTTGFEHNNGCEVIGFEPERWIHNVDVDSLGRASRFSTAAARLAVADSGIDLDVLREARCLLSVG TTDGESHELDSMVSLEIAGGPEAMSPRLARRTSAGRLATSIAHELRLSNVEVGSIATACSAGNYAIGHSFDAIRLGEADFALCGGADAVCRKTFTSFYRL RSIAPDFCRPFDKNRLGILTGEGAGILMLESLESATARGARIYGEVLGYGLSCDAHHPVAPHQGGIARCMELALANAGVKPAEVDLISAHGTGTHANDKT EAAAIRELFGEDTPRVVSMKSMLGHSMGAASALAAIGCSVAITEGFIPPTINHVETDPECAVDCVPNVAVEADLRIVQNNGWAFGGNNSVVLIGRYDGPR EVVA&LINK_LOC=protein&PAGE_TYPE=BlastSearch', 'BLAST this protein','AMED_4044')" />
   GTTMSGVQSLAEAQRALDLEGPQAIHRKLNIKAWPNMAAGQLALRWKLHGPLLTVSSACASANDAIGTAAQMIEAGRADVMIAGGTEHGLCEILYHAQTS YGMSQGTPDPTMAMLPFDRRRTGIVEGEGAAVLVLERLDRAVARGAHIHGMVRGYASLSDGHHPSSPDPSGRWEAEAIRRAHSDAGIGSDAVDAIVAHAT STPKGDTVEIAAINEVFGDRGDSLLVTSIKGSVGHTGAASAAMGVLVGLQAMEAGALPPVGGTAEVEDGPLFRVVVGEPAKADIGIVQVNAFGFGGQNSS LVVSRD', 'http://blast.ncbi.nlm.nih.gov/Blast.cgi?PAGE=Proteins&PROGRAM=blastp&BLAST_PROGRAMS=blastp&QUERY=MEQVAITGVGIVSPLGQSADDFTAAMQAGRVRIVQAPWADPDSGRYAWVSTIPDFDPTAWMDDRVVDGTDVFAQYAIAAAAQAVADHGAELDPLRTGVVL GTTMSGVQSLAEAQRALDLEGPQAIHRKLNIKAWPNMAAGQLALRWKLHGPLLTVSSACASANDAIGTAAQMIEAGRADVMIAGGTEHGLCEILYHAQTS YGMSQGTPDPTMAMLPFDRRRTGIVEGEGAAVLVLERLDRAVARGAHIHGMVRGYASLSDGHHPSSPDPSGRWEAEAIRRAHSDAGIGSDAVDAIVAHAT STPKGDTVEIAAINEVFGDRGDSLLVTSIKGSVGHTGAASAAMGVLVGLQAMEAGALPPVGGTAEVEDGPLFRVVVGEPAKADIGIVQVNAFGFGGQNSS LVVSRD&LINK_LOC=protein&PAGE_TYPE=BlastSearch', 'BLAST this protein','Psed_0508')" />
   HELDSMVSLEIAGGPEAMSPRLARRTSAGRLATSIAHELRLSNVEVGSIATACSAGNYAIGHSFDAIRLGEADFALCGGADAVCRKTFTSFYRLRSIAPD FCRPFDKNRLGILTGEGAGILMLESLESATARGARIYGEVLGYGLSCDAHHPVAPHQGGIARCMELALANAGVKPAEVDLISAHGTGTHANDKTEAAAIR ELFGEDTPRVVSMKSMLGHSMGAASALAAIGCSVAITEGFIPPTINHVETDPECAVDCVPNVAVEADLRIVQNNGWAFGGNNSVVLIGRYDGPREVVA', 'http://blast.ncbi.nlm.nih.gov/Blast.cgi?PAGE=Proteins&PROGRAM=blastp&BLAST_PROGRAMS=blastp&QUERY=MVSSLGTGIEEFTAALRAGRSNVRPITVFDTTGFEHNNGCEVIGFEPERWIHNVDVDSLGRASRFSTAAARLAVADSGIDLDVLREARCLLSVGTTDGES HELDSMVSLEIAGGPEAMSPRLARRTSAGRLATSIAHELRLSNVEVGSIATACSAGNYAIGHSFDAIRLGEADFALCGGADAVCRKTFTSFYRLRSIAPD FCRPFDKNRLGILTGEGAGILMLESLESATARGARIYGEVLGYGLSCDAHHPVAPHQGGIARCMELALANAGVKPAEVDLISAHGTGTHANDKTEAAAIR ELFGEDTPRVVSMKSMLGHSMGAASALAAIGCSVAITEGFIPPTINHVETDPECAVDCVPNVAVEADLRIVQNNGWAFGGNNSVVLIGRYDGPREVVA&LINK_LOC=protein&PAGE_TYPE=BlastSearch', 'BLAST this protein','RAM_20610')" />
   AAARMAVADAGLHEEDVRARRSLVSVGTTDGESRDLDQLVAAGLENGPEEYEPAVARRIPAGRLSAGIVRELELEDVEAVTIPTACAAGNYAVGYGYDAL RSGDVDIALCGGADAACRKTFTGFYRLGTIAPEVCRPFDKDREGILTGEGAGILLMESLDSALARGARIYAEVLGYGLACDALHPVAPDRDSIARTIRIA HRNAGITPDQVDFVSAHGTGTKANDITEAGAIRQVFGEDAMPRTVSIKSMIGHSMGAASAVSAAACALAITEGFIPPTVNHRETDPECGVDCVPNQAVET ELSVVQNNALAFGGNNAVLILGAYRSAA', 'http://blast.ncbi.nlm.nih.gov/Blast.cgi?PAGE=Proteins&PROGRAM=blastp&BLAST_PROGRAMS=blastp&QUERY=MNASINTDARGHGGPEDPRPEAAGRRVVITGLGVVTSIGIGVEAFTAGLRAGRSGVKPITAFDTTGFAHANGCEVTDFEPERWLDRQHPEELGRASQFAV AAARMAVADAGLHEEDVRARRSLVSVGTTDGESRDLDQLVAAGLENGPEEYEPAVARRIPAGRLSAGIVRELELEDVEAVTIPTACAAGNYAVGYGYDAL RSGDVDIALCGGADAACRKTFTGFYRLGTIAPEVCRPFDKDREGILTGEGAGILLMESLDSALARGARIYAEVLGYGLACDALHPVAPDRDSIARTIRIA HRNAGITPDQVDFVSAHGTGTKANDITEAGAIRQVFGEDAMPRTVSIKSMIGHSMGAASAVSAAACALAITEGFIPPTVNHRETDPECGVDCVPNQAVET ELSVVQNNALAFGGNNAVLILGAYRSAA&LINK_LOC=protein&PAGE_TYPE=BlastSearch', 'BLAST this protein','SAV_3665')" />
   DVPTLISVGTTDGESRDLDHLVEVEVDQGPEQMDAVIARRVPAGRLSAAIAQEFGLTRVEAVTLPTACAAGNYAIGYGFDAIRGGDVDFALCGGADALCR KTFTGFYRLGTIAPERCQPFDKDRKGILTGEGAGILLLESLESALARGARIYAEVLGYGLNCDAHHPVAPHQDSVARCMRIALENAEVKPEEVDFISAHG TGTKANDVTEARAIRQVFGEDDPPRTVSVKSMIGHTMGAASALAAIACSLALTEGFIPPTINHAETDPECALDVVPNRAVDADVKIVQNNGLAFGGNNAV VIFGKRDTDWS', 'http://blast.ncbi.nlm.nih.gov/Blast.cgi?PAGE=Proteins&PROGRAM=blastp&BLAST_PROGRAMS=blastp&QUERY=MQGEPARRVVITGLGTVSGIGVGTAEFLAGLRSGKSAAKPITAFDTEGFDHATACEITDFDPGEWIRHLDVRTLGRASQFSVSAARMAVADAGLTEHDLR DVPTLISVGTTDGESRDLDHLVEVEVDQGPEQMDAVIARRVPAGRLSAAIAQEFGLTRVEAVTLPTACAAGNYAIGYGFDAIRGGDVDFALCGGADALCR KTFTGFYRLGTIAPERCQPFDKDRKGILTGEGAGILLLESLESALARGARIYAEVLGYGLNCDAHHPVAPHQDSVARCMRIALENAEVKPEEVDFISAHG TGTKANDVTEARAIRQVFGEDDPPRTVSVKSMIGHTMGAASALAAIACSLALTEGFIPPTINHAETDPECALDVVPNRAVDADVKIVQNNGLAFGGNNAV VIFGKRDTDWS&LINK_LOC=protein&PAGE_TYPE=BlastSearch', 'BLAST this protein','SBI_06743')" />
   GTTDGEARELDQLVESELAVGMAGLDPTAVRRLPAHRLALAVVRELRLPQAVPSVLGTACAAGNYAIGDGFDAVRFGEADYALVGGSDALARRTFAGFSR LGLVASDRCRPFDAEREGLLTAEGAGVLLLETLESARARGARIYAELLGYGLNCDAHHPTAPDQESVARCMGLALADAGIKPEEVDLVSAHGTGTKTNDI TETRAIRELFGDRPPRTVALKSMLGHSMGAASALGAAASAVALAHGFIPPTVNHRSTDPECGIDCVPNHAVPAPLRIVVNNGLAFGGNNASVVLGRYEED QR', 'http://blast.ncbi.nlm.nih.gov/Blast.cgi?PAGE=Proteins&PROGRAM=blastp&BLAST_PROGRAMS=blastp&QUERY=MVLTGLGVLSSIGNGVTEFTRGLREGRCGAGEITLFDATGFRRQLAHEVKDFEPAQWIRHTPLEELGRASQFSVAATRLAVQDAGLDLEELRGRRGLVTI GTTDGEARELDQLVESELAVGMAGLDPTAVRRLPAHRLALAVVRELRLPQAVPSVLGTACAAGNYAIGDGFDAVRFGEADYALVGGSDALARRTFAGFSR LGLVASDRCRPFDAEREGLLTAEGAGVLLLETLESARARGARIYAELLGYGLNCDAHHPTAPDQESVARCMGLALADAGIKPEEVDLVSAHGTGTKTNDI TETRAIRELFGDRPPRTVALKSMLGHSMGAASALGAAASAVALAHGFIPPTVNHRSTDPECGIDCVPNHAVPAPLRIVVNNGLAFGGNNASVVLGRYEED QR&LINK_LOC=protein&PAGE_TYPE=BlastSearch', 'BLAST this protein','SBI_06796')" />
   AARAGSAMGTTLGESAVIEALTSQWVHGGLKDLDPALVRQAPAGRIATAVNAELGLTGEAHTIATACSASNYTLGYAYDMVRTGEAEFMISGGADSVNRW EHAGFYRLGALAESVCRPFDTHRSGILTAEGGVALFLEPLEGAVARGARIYAEVLGYGVNCDAKHPVAPDPTSIAECIRAAHRNAGVRPEDIDYICAHGT GTPTNDATEVKAVREVFGDELPPISSIKSMIGHTMGAASGFGALICCMALHDGFLPPTANVTETDPALGPGVDCVPGTGRAASPRIVQNHGFAFGGNNAI TILGRAA', 'http://blast.ncbi.nlm.nih.gov/Blast.cgi?PAGE=Proteins&PROGRAM=blastp&BLAST_PROGRAMS=blastp&QUERY=MPEGAPRRVVVTGLGAVSSLGTGARTFAKAIREGRSGVSPIRSFDASGFPYTFAGEVHDFEPERLLRRLRVDEWGRSSLFAASAARLALMDADIDPGQVA AARAGSAMGTTLGESAVIEALTSQWVHGGLKDLDPALVRQAPAGRIATAVNAELGLTGEAHTIATACSASNYTLGYAYDMVRTGEAEFMISGGADSVNRW EHAGFYRLGALAESVCRPFDTHRSGILTAEGGVALFLEPLEGAVARGARIYAEVLGYGVNCDAKHPVAPDPTSIAECIRAAHRNAGVRPEDIDYICAHGT GTPTNDATEVKAVREVFGDELPPISSIKSMIGHTMGAASGFGALICCMALHDGFLPPTANVTETDPALGPGVDCVPGTGRAASPRIVQNHGFAFGGNNAI TILGRAA&LINK_LOC=protein&PAGE_TYPE=BlastSearch', 'BLAST this protein','Sros_4188')" />
   LRDIPCLISVGTTDGESRDLDHLVEEEVDLGPERMDPTVARRVPAGRLSSAIAQEFGLTRAEAVTLPTACAAGNYAIGYGFDAIRGGDVDLALCGGADAL CRKTFTGFYRLGTIAPERCQPFDKDRKGILTGEGAGILVLESLESALARGARIYAEVLGYGLNCDADHPVAPNQDSVARCMRLALDNAHVKPEEVDFISA HGTGTKANDITEARAIRQVFGDAAPPRTVSIKSMIGHSMGAAGALASIACALALTEGFIPPTINHQETDPECGLDCVPNQAVDADLKVVQNNGLAFGGNN AVVIFGKSRADWS', 'http://blast.ncbi.nlm.nih.gov/Blast.cgi?PAGE=Proteins&PROGRAM=blastp&BLAST_PROGRAMS=blastp&QUERY=MRGEPGRRVVVTGLGAVSGIGIGAAEFLAGLRAGKSAAGPITAFDTEGFDRSTACEVKDFEPDRWIRNLDVRTLGRASRFSVAAARMAVADAGFAESESA LRDIPCLISVGTTDGESRDLDHLVEEEVDLGPERMDPTVARRVPAGRLSSAIAQEFGLTRAEAVTLPTACAAGNYAIGYGFDAIRGGDVDLALCGGADAL CRKTFTGFYRLGTIAPERCQPFDKDRKGILTGEGAGILVLESLESALARGARIYAEVLGYGLNCDADHPVAPNQDSVARCMRLALDNAHVKPEEVDFISA HGTGTKANDITEARAIRQVFGDAAPPRTVSIKSMIGHSMGAAGALASIACALALTEGFIPPTINHQETDPECGLDCVPNQAVDADLKVVQNNGLAFGGNN AVVIFGKSRADWS&LINK_LOC=protein&PAGE_TYPE=BlastSearch', 'BLAST this protein','Strvi_8201')" />
   DWRSLPGLISIGTTDGESRDLDHLVEAEITHGPEHMPPELARRVPAGRLSAAIAQELQLSDVEAVTIPTACAAGNYALGYGYDAVRSGDAEYALCGGADA MCRKTFSGFYRLGTIAPDRCRPFDVDRKGILTGEGAGVLVLESLESALARGARIYAEVLGYGLNCDAYHQVAPNQDSVGRCMQLALENAGVKADQVDLIS AHGTGTKANDVTEARAIREVFGSRPPRTISMKSMIGHTMGAASALSAIGCALAITEKFIPPTINHTTTDPECDVDCVPNVAIEAELKVVQNNGLAFGGNN AVVLLGRYDGVPA', 'http://blast.ncbi.nlm.nih.gov/Blast.cgi?PAGE=Proteins&PROGRAM=blastp&BLAST_PROGRAMS=blastp&QUERY=MAESGHASPRRVVITGLGVVSSIGMGVEEFRRGLRAGSSGAKPITVFDVEGFDHANGCEVEDFDPERWIRHLAVEQLGRATQYSVAAARMAIEDAGVSIA DWRSLPGLISIGTTDGESRDLDHLVEAEITHGPEHMPPELARRVPAGRLSAAIAQELQLSDVEAVTIPTACAAGNYALGYGYDAVRSGDAEYALCGGADA MCRKTFSGFYRLGTIAPDRCRPFDVDRKGILTGEGAGVLVLESLESALARGARIYAEVLGYGLNCDAYHQVAPNQDSVGRCMQLALENAGVKADQVDLIS AHGTGTKANDVTEARAIREVFGSRPPRTISMKSMIGHTMGAASALSAIGCALAITEKFIPPTINHTTTDPECDVDCVPNVAIEAELKVVQNNGLAFGGNN AVVLLGRYDGVPA&LINK_LOC=protein&PAGE_TYPE=BlastSearch', 'BLAST this protein','Strvi_8803')" />
   AVIGTTSGESVVLEALTARLVEAGFDAVPPQLVEQLPAGRLAHAVSEELGLRGDSLTLATACSASNYAIGYAYDLLVSGESDVVFAGGADSVCRWAHAGF YRLGALTEHACAPFDRDRSGIITGEGGAVLALETWEHAEARGAHVYAEVLGYGLNCDANHPVAPDRDSIAECIRIAHRNAGVTPEQVDYICAHGTGTPAN DLVEAQAVLEVFGANPPPISSIKSMIGHTMGAASGFGAIASALAIEQGFLPPTINWSHPDPQLTGIDPVPNEARPATVRLVQNNGFAFGGNNAIVLLGAA R', 'http://blast.ncbi.nlm.nih.gov/Blast.cgi?PAGE=Proteins&PROGRAM=blastp&BLAST_PROGRAMS=blastp&QUERY=MRRVVVTGLGPVSSIGTGVTAFGAALRAGRSGISQITSFDPSGFPYRNGGEVRDFDPAAWLHRLPTDRWGRSSQFAAAAARLAAEDAKLDLDAVDRTRAH AVIGTTSGESVVLEALTARLVEAGFDAVPPQLVEQLPAGRLAHAVSEELGLRGDSLTLATACSASNYAIGYAYDLLVSGESDVVFAGGADSVCRWAHAGF YRLGALTEHACAPFDRDRSGIITGEGGAVLALETWEHAEARGAHVYAEVLGYGLNCDANHPVAPDRDSIAECIRIAHRNAGVTPEQVDYICAHGTGTPAN DLVEAQAVLEVFGANPPPISSIKSMIGHTMGAASGFGAIASALAIEQGFLPPTINWSHPDPQLTGIDPVPNEARPATVRLVQNNGFAFGGNNAIVLLGAA R&LINK_LOC=protein&PAGE_TYPE=BlastSearch', 'BLAST this protein','VAB18032_07160')" />

 
 
   AGAILHLGTERHHERYLADALTGDLLGCFAMTETGHGSNVQALETTATYDPATGEFVVHTPTASARKDYIGNAARHGRLAAVFAQLVVGGQTLGVHCLLV PIRTDDGQPVTGVTISDCGPKLGLNGVDNGRIVFDQVRVPREALLDRYAQVDADGTYRSEIENPDRRFFTMLGTLVQGRVSVGGAAINASKVALTIAVRY AEQRRQFGAPGSPEEALLLDYRMHQRRLLPLLARTYALHFAQAELVDEFARIFGADTATGQDGGSAPDGGSRSDEHDRRALEAQAAGTKAVGTWHATETI QLCREACGGAGYLAENRLSTLKADTDVFTTFEGDNTVLLQLVAKGLLTDYRESFGKLDPLGTARFVAGQAVEIAVEKTALRGLIERLRDAVPNRSDAGDP DAGLRDDDYHSGLLRFREQHMLAGVARRLRAGIDAGDEAFEVFNRCQDHVIAAGRAHVERVVLEAFQRAVHAAPEGETRDRLKELYDLHALTTIENDRAW FMEHGRLSGPRSKAITALVNDLCGRVRPHASSLVDAFGVPGAAVDVPMVVRSLHE', 'http://blast.ncbi.nlm.nih.gov/Blast.cgi?PAGE=Proteins&PROGRAM=blastp&BLAST_PROGRAMS=blastp&QUERY=MTGLREYLDGPHKAARDVVRDGLATHAGLLDAGLRLSRDEYRDQVLATLREVAAQGHPVRGFPKEYGGQGDLGGFIAGFETLAFGDLSLMVKAGVQFGLF AGAILHLGTERHHERYLADALTGDLLGCFAMTETGHGSNVQALETTATYDPATGEFVVHTPTASARKDYIGNAARHGRLAAVFAQLVVGGQTLGVHCLLV PIRTDDGQPVTGVTISDCGPKLGLNGVDNGRIVFDQVRVPREALLDRYAQVDADGTYRSEIENPDRRFFTMLGTLVQGRVSVGGAAINASKVALTIAVRY AEQRRQFGAPGSPEEALLLDYRMHQRRLLPLLARTYALHFAQAELVDEFARIFGADTATGQDGGSAPDGGSRSDEHDRRALEAQAAGTKAVGTWHATETI QLCREACGGAGYLAENRLSTLKADTDVFTTFEGDNTVLLQLVAKGLLTDYRESFGKLDPLGTARFVAGQAVEIAVEKTALRGLIERLRDAVPNRSDAGDP DAGLRDDDYHSGLLRFREQHMLAGVARRLRAGIDAGDEAFEVFNRCQDHVIAAGRAHVERVVLEAFQRAVHAAPEGETRDRLKELYDLHALTTIENDRAW FMEHGRLSGPRSKAITALVNDLCGRVRPHASSLVDAFGVPGAAVDVPMVVRSLHE&LINK_LOC=protein&PAGE_TYPE=BlastSearch', 'BLAST this protein','Kfla_6493')" />
   VQWGLFGGAVENLGTERHHEQYVPKIIDLQLRGCFAMTETGHGSDVQSLETTATYDADTEEFIIDSPTPTARKDYIGGAAETATIAAVFAQLITQIDGEQ ANHGVHCFLVPIRDEDGNDLPGVTTSDCHYKGGLPGVDNGRITFDHVRIPRVNLLNRYGDVATDGTYSSPIDNPNRRFFTMIGTLIRGRVTVGGSAAAAA RVALDIATRYALQRRQFSAPDDDDTEVLIMDYLMHQRRLFPLIARSYALQFAQNELVSKCHDLQTTDSPDAEEQRELEARAAGLKAANTWHASRAIQEAR EACGGAGYMAENRLIALRADTDVFTTFEGDNHVLTQLVAKELLTAYADDIRSMSPVEWVRFAANAVGDRVMKRTAAEAIMQRIVDARQDNEEEGSLFNRG TQVKMFEDREDYLLSSVARRLQNKSNEMSAFDAFNAVQDHVLHAAQAHIDRVVLEAFVAGIESCTDEQARELLGVVCDLYALSVIEEDKAWYIEHRYLST ERAKAVTRGINDRCRVLRPHARTLVDGFGIPKPLRYAEMLHPENLPD', 'http://blast.ncbi.nlm.nih.gov/Blast.cgi?PAGE=Proteins&PROGRAM=blastp&BLAST_PROGRAMS=blastp&QUERY=MAPDMTNTAKHLRDALDGRFRDVKNEMRQQLTLEIFRPHYTPNTVIARAKVAEQMKFMAGAGAAEDSFRKEHGGTGNVGAAITMIEMLAMSDLSLMVKAG VQWGLFGGAVENLGTERHHEQYVPKIIDLQLRGCFAMTETGHGSDVQSLETTATYDADTEEFIIDSPTPTARKDYIGGAAETATIAAVFAQLITQIDGEQ ANHGVHCFLVPIRDEDGNDLPGVTTSDCHYKGGLPGVDNGRITFDHVRIPRVNLLNRYGDVATDGTYSSPIDNPNRRFFTMIGTLIRGRVTVGGSAAAAA RVALDIATRYALQRRQFSAPDDDDTEVLIMDYLMHQRRLFPLIARSYALQFAQNELVSKCHDLQTTDSPDAEEQRELEARAAGLKAANTWHASRAIQEAR EACGGAGYMAENRLIALRADTDVFTTFEGDNHVLTQLVAKELLTAYADDIRSMSPVEWVRFAANAVGDRVMKRTAAEAIMQRIVDARQDNEEEGSLFNRG TQVKMFEDREDYLLSSVARRLQNKSNEMSAFDAFNAVQDHVLHAAQAHIDRVVLEAFVAGIESCTDEQARELLGVVCDLYALSVIEEDKAWYIEHRYLST ERAKAVTRGINDRCRVLRPHARTLVDGFGIPKPLRYAEMLHPENLPD&LINK_LOC=protein&PAGE_TYPE=BlastSearch', 'BLAST this protein','MMAR_3631')" />
   VQFGLFGGAILHLGTGRHHDAYLPDLITGKLMGCFAMTETGHGSNVQALGTLATYDAATQEFVITTDGDQARKDYIGNAARHAELAVVFAQLEVGGESKG VHAFVVPVRTGGEVVPGVRIEDDGRKMGLNGVDNGRIRFDGVRVPREALLNRFADVTPDGVYESPIENPDRRFFTMLGTLVQGRVSVGGAGVNAAKVALT IATKYAVRRRQFDAAPDTEEQLLLDYGLHQRRLLPLLARTYALHFAQDVVRTHLHEVLSGIKDDAYERRQLESRAAGTKALGTWHATRVVQECREACGGA GYLAVNRFAALKSDSDIFTTFEGDNHVLLQLVAKGLLTHYASEFEDLDQLGMVRHVTGLAVETVIEKTSAHKLLERVRDLLPGGDEWDQEAGLRDSEYQL AMLRYREEHMLAGVARRLKRGIDQKRDPGAVFSQVQDHVIAVAHAHVERLVLEAFVDKLRAQPEGGNKVALGLLCDLFALSTIEADRAWFMEHGRLTVQR SKAISREVHDLCRKVRPLAVDLVDAWGIPPEMLRAPDLVG', 'http://blast.ncbi.nlm.nih.gov/Blast.cgi?PAGE=Proteins&PROGRAM=blastp&BLAST_PROGRAMS=blastp&QUERY=MQPEVDVRALTEVLDGEYAAIRDLVRTNLVTHASVLEEADELGVDAYRERVRELVVEMAATGQTGMGFPAKYGGGGDVGASIAAFETLAFGDLSVLVKVG VQFGLFGGAILHLGTGRHHDAYLPDLITGKLMGCFAMTETGHGSNVQALGTLATYDAATQEFVITTDGDQARKDYIGNAARHAELAVVFAQLEVGGESKG VHAFVVPVRTGGEVVPGVRIEDDGRKMGLNGVDNGRIRFDGVRVPREALLNRFADVTPDGVYESPIENPDRRFFTMLGTLVQGRVSVGGAGVNAAKVALT IATKYAVRRRQFDAAPDTEEQLLLDYGLHQRRLLPLLARTYALHFAQDVVRTHLHEVLSGIKDDAYERRQLESRAAGTKALGTWHATRVVQECREACGGA GYLAVNRFAALKSDSDIFTTFEGDNHVLLQLVAKGLLTHYASEFEDLDQLGMVRHVTGLAVETVIEKTSAHKLLERVRDLLPGGDEWDQEAGLRDSEYQL AMLRYREEHMLAGVARRLKRGIDQKRDPGAVFSQVQDHVIAVAHAHVERLVLEAFVDKLRAQPEGGNKVALGLLCDLFALSTIEADRAWFMEHGRLTVQR SKAISREVHDLCRKVRPLAVDLVDAWGIPPEMLRAPDLVG&LINK_LOC=protein&PAGE_TYPE=BlastSearch', 'BLAST this protein','Sfla_5665')" />

 
 
   SVASPALLGRLRSDLLECAGDRRLVLVVDDARWVDVPSLRFLSYLGARAERTPVLLAVALDPADRGSAADWLGSVSTSLETTLLRLAPLSEVATRRLVSV MLGTASSPEFAAACFDATGGNPLLLHEVLAEAAAGRSGSPEEALSGLSRRPPVRVMRQMVRRLQSLPRSSVAVARAVAVLGVAEDPVVVSRTADCPEAEV DDAVTVLVDAEVMTDGPALRFAQPMLRTAVYAGIPAFLRGRLHARAARALAETGAPSEAVAEQLVNVPPAMVPAAGAKELAEPRSRNRRASRHLRPVWGE NPVERAAAALEAARTLTLDCQFDRAIAILEAAIADVRDPALSPRLEATAVWIAQLSPSTRGRGRARLDTLERPHLAEKALLDQIWRGTPAEHIGRLTGDL LHRPRIPAGDSTGWSAGLVAAVTLAHFDRLAETRQAVLTLRDAASQTHTTLAGLDLCLRAAVDYQLGDIAGAGDLALQVLSGTDGQRPARMVQAFAGAVR AETLIAQGALDAAAAMVEEPGLIDDCGSDTIAVLPLLRARGHLRIAQDRLEEGLTDLLRGRRAAASLGLTPGAWVGWSSAVTALHRLGRTEEALFMAREE LAGARSCGAPAPLAAALRTLGLLTPGPAALPLVEEAAGLMEDFPGQLEKARTQLTYGIMLHRVGKRAAAREQLRMAAGTSGVHGAHVLNRRAIDELRSIE GDTTDRRSRTVLTPQQQRVAELAAEGMQNEEIAQKLFVTVKTVEWHLTQAYRKLGIESRTALSPALAATTLDYAKSA', 'http://blast.ncbi.nlm.nih.gov/Blast.cgi?PAGE=Proteins&PROGRAM=blastp&BLAST_PROGRAMS=blastp&QUERY=MVFDLFDELPHPLLERDVETQWIERKLRRLRGGHGGVLAIEGSAGTGRSSLLKLLYQRAAEAGFCVLYARGNEFEGDVPFGAVAQLLEPVLPARMVEESP SVASPALLGRLRSDLLECAGDRRLVLVVDDARWVDVPSLRFLSYLGARAERTPVLLAVALDPADRGSAADWLGSVSTSLETTLLRLAPLSEVATRRLVSV MLGTASSPEFAAACFDATGGNPLLLHEVLAEAAAGRSGSPEEALSGLSRRPPVRVMRQMVRRLQSLPRSSVAVARAVAVLGVAEDPVVVSRTADCPEAEV DDAVTVLVDAEVMTDGPALRFAQPMLRTAVYAGIPAFLRGRLHARAARALAETGAPSEAVAEQLVNVPPAMVPAAGAKELAEPRSRNRRASRHLRPVWGE NPVERAAAALEAARTLTLDCQFDRAIAILEAAIADVRDPALSPRLEATAVWIAQLSPSTRGRGRARLDTLERPHLAEKALLDQIWRGTPAEHIGRLTGDL LHRPRIPAGDSTGWSAGLVAAVTLAHFDRLAETRQAVLTLRDAASQTHTTLAGLDLCLRAAVDYQLGDIAGAGDLALQVLSGTDGQRPARMVQAFAGAVR AETLIAQGALDAAAAMVEEPGLIDDCGSDTIAVLPLLRARGHLRIAQDRLEEGLTDLLRGRRAAASLGLTPGAWVGWSSAVTALHRLGRTEEALFMAREE LAGARSCGAPAPLAAALRTLGLLTPGPAALPLVEEAAGLMEDFPGQLEKARTQLTYGIMLHRVGKRAAAREQLRMAAGTSGVHGAHVLNRRAIDELRSIE GDTTDRRSRTVLTPQQQRVAELAAEGMQNEEIAQKLFVTVKTVEWHLTQAYRKLGIESRTALSPALAATTLDYAKSA&LINK_LOC=protein&PAGE_TYPE=BlastSearch', 'BLAST this protein','Sare_4559')" />

 
 
   TALVTSAHTLYLARFALGVAEGGFFPGVIAYLTVWFPCAQRARAVATFLLAIPVANTVGLPLSGLIVGHVHMAGLPGWRAMFVIEALPALLLAPLLRRLL PDNPQRASWLTPEERAELSARLTEDTPAPTGRSSGAGWDLVLFAVVYGGLYFALYALQFFLPQLVASLAHGTATLTAATLAALPYGVAALAMLAWSHRSI DRSGAQAGHITLPTTAAGSAALGAALSPMSPIVTLSWLTIAVAGILAAMPAFWSRCTAALAGPRVAVAIATVNAVASLASFAGPYATGHLKDATGTYHLA LLTVAAVLAAAAACSLLLRHAGRTVCANDSEIMLHPSPATPFV', 'http://blast.ncbi.nlm.nih.gov/Blast.cgi?PAGE=Proteins&PROGRAM=blastp&BLAST_PROGRAMS=blastp&QUERY=MATIAASPTHNALGKAARRLLPLLFVLYVINFVDRANISVAALAMNADLRLSATAYGTAAGVFFLGYVLFQVPANAALARFGAGRTLTAVVLAWGVCSAA TALVTSAHTLYLARFALGVAEGGFFPGVIAYLTVWFPCAQRARAVATFLLAIPVANTVGLPLSGLIVGHVHMAGLPGWRAMFVIEALPALLLAPLLRRLL PDNPQRASWLTPEERAELSARLTEDTPAPTGRSSGAGWDLVLFAVVYGGLYFALYALQFFLPQLVASLAHGTATLTAATLAALPYGVAALAMLAWSHRSI DRSGAQAGHITLPTTAAGSAALGAALSPMSPIVTLSWLTIAVAGILAAMPAFWSRCTAALAGPRVAVAIATVNAVASLASFAGPYATGHLKDATGTYHLA LLTVAAVLAAAAACSLLLRHAGRTVCANDSEIMLHPSPATPFV&LINK_LOC=protein&PAGE_TYPE=BlastSearch', 'BLAST this protein','Mb_1699c')" />

 
 
   PVHHTVRADAGCTWGDVDHATVAFGMATPSGIVASTGVAGLTLGGGIGYLARRFGLTVDNLLGADVVLADGTFAHASESEHADLFWALRGGGGNFGVVTS FTFRCHDIGEHGVVIGGPVLYDLADTPDVLRWYRELLPSLPEELNGWFGLLTVPPAPPFPEQLWGRKACGIVWCYTGSHDKAEEVLEPVRSFGTPLLVGL QSMPYTALQGAFDALYPAGLQWYWRADVFHEISDEAIDIHLKHGEKLPTMHSSMHLYPIDGAASRVAPDAMAFPHRSGGWSGVIVGVDPAPEKAEAIAQW TRDYWEELHPTSAGGAYVNFMMEEGQDRVQASYRGNYDRLAAVKRRYDPENVFHINQNIRPK', 'http://blast.ncbi.nlm.nih.gov/Blast.cgi?PAGE=Proteins&PROGRAM=blastp&BLAST_PROGRAMS=blastp&QUERY=MTTTALPHEELAATLRGDLVTPADPGYDQARAVYNAMIDKRPAAIAYCRDAADVVACVRYGRAQGLDLAVRGGGHNAGGLGVADGALVIDLSRLRSTTVD PVHHTVRADAGCTWGDVDHATVAFGMATPSGIVASTGVAGLTLGGGIGYLARRFGLTVDNLLGADVVLADGTFAHASESEHADLFWALRGGGGNFGVVTS FTFRCHDIGEHGVVIGGPVLYDLADTPDVLRWYRELLPSLPEELNGWFGLLTVPPAPPFPEQLWGRKACGIVWCYTGSHDKAEEVLEPVRSFGTPLLVGL QSMPYTALQGAFDALYPAGLQWYWRADVFHEISDEAIDIHLKHGEKLPTMHSSMHLYPIDGAASRVAPDAMAFPHRSGGWSGVIVGVDPAPEKAEAIAQW TRDYWEELHPTSAGGAYVNFMMEEGQDRVQASYRGNYDRLAAVKRRYDPENVFHINQNIRPK&LINK_LOC=protein&PAGE_TYPE=BlastSearch', 'BLAST this protein','AMED_2696')" />
   ATWGDVDPVTSRHGLACPGGVVSTTGVGGFSLGGGIGWLSRAHGMTCDNLIGATLVTASGNVLTVSETEHADVLWGLRGGGGNFGVVAQFVLRLHPVNAV TAGVQSYPDTDAEAALKHFRAQMDNAPDHLASILDFSTDFTTGKTLVNVLACSTRTDSIGSNDVSALLDVRGVAAKPVVAVQHTLDYSTWQQALDYTAPY GWLNYWKSLFVTELTDEAIRRIALLGRSRPSTQTRLHLIRLGGYASRVPPESTAVVARDHPYIVHLMTTWTDPADTARCTAWAGQAFEILRPLGPASAYL NFVGDEGQDRIRATFGDAGYQRLAELKARLDPENRFTLNHNIEPAD', 'http://blast.ncbi.nlm.nih.gov/Blast.cgi?PAGE=Proteins&PROGRAM=blastp&BLAST_PROGRAMS=blastp&QUERY=MPGFSGEVIAPSDARYDEVRQVFNGMIDKRPRVVLQCRSTDDIVAAVRYAVAAGAEIAVRSGGHSVAGHSATDGGIVIDLTLMRGVRVDAQARIAYVDAG ATWGDVDPVTSRHGLACPGGVVSTTGVGGFSLGGGIGWLSRAHGMTCDNLIGATLVTASGNVLTVSETEHADVLWGLRGGGGNFGVVAQFVLRLHPVNAV TAGVQSYPDTDAEAALKHFRAQMDNAPDHLASILDFSTDFTTGKTLVNVLACSTRTDSIGSNDVSALLDVRGVAAKPVVAVQHTLDYSTWQQALDYTAPY GWLNYWKSLFVTELTDEAIRRIALLGRSRPSTQTRLHLIRLGGYASRVPPESTAVVARDHPYIVHLMTTWTDPADTARCTAWAGQAFEILRPLGPASAYL NFVGDEGQDRIRATFGDAGYQRLAELKARLDPENRFTLNHNIEPAD&LINK_LOC=protein&PAGE_TYPE=BlastSearch', 'BLAST this protein','JDM601_0487')" />
   PVHHTVRADAGCTWGDVDHATVAFGMATPSGIVASTGVAGLTLGGGIGYLARRFGLTVDNLLGADVVLADGTFAHASESEHADLFWALRGGGGNFGVVTS FTFRCHDIGEHGVVIGGPVLYDLADTPDVLRWYRELLPSLPEELNGWFGLLTVPPAPPFPEQLWGRKACGIVWCYTGSHDKAEEVLEPVRSFGTPLLVGL QSMPYTALQGAFDALYPAGLQWYWRADVFHEISDEAIDIHLKHGEKLPTMHSSMHLYPIDGAASRVAPDAMAFPHRSGGWSGVIVGVDPAPEKAEAIAQW TRDYWEELHPTSAGGAYVNFMMEEGQDRVQASYRGNYDRLAAVKRRYDPENVFHINQNIRPK', 'http://blast.ncbi.nlm.nih.gov/Blast.cgi?PAGE=Proteins&PROGRAM=blastp&BLAST_PROGRAMS=blastp&QUERY=MTTTALPHEELAATLRGDLVTPADPGYDQARAVYNAMIDKRPAAIAYCRDAADVVACVRYGRAQGLDLAVRGGGHNAGGLGVADGALVIDLSRLRSTTVD PVHHTVRADAGCTWGDVDHATVAFGMATPSGIVASTGVAGLTLGGGIGYLARRFGLTVDNLLGADVVLADGTFAHASESEHADLFWALRGGGGNFGVVTS FTFRCHDIGEHGVVIGGPVLYDLADTPDVLRWYRELLPSLPEELNGWFGLLTVPPAPPFPEQLWGRKACGIVWCYTGSHDKAEEVLEPVRSFGTPLLVGL QSMPYTALQGAFDALYPAGLQWYWRADVFHEISDEAIDIHLKHGEKLPTMHSSMHLYPIDGAASRVAPDAMAFPHRSGGWSGVIVGVDPAPEKAEAIAQW TRDYWEELHPTSAGGAYVNFMMEEGQDRVQASYRGNYDRLAAVKRRYDPENVFHINQNIRPK&LINK_LOC=protein&PAGE_TYPE=BlastSearch', 'BLAST this protein','RAM_13705')" />
   MNAVAVDPLTRTAHVEAGAQWHQVIAAGAPHGLAPLNGSSPLVGVVGYTLGGGLGPLGRQYGYAADHVTCIEVVTADGSILRVTRDRHPDLFWALRGGKG NFGIVTGLWFGLVPVRRLHGGGIYFPGEQAAQALHTWRQWTATVPEEMTSSVALLRLPDVPDVPEFLRGTFAAHVRIAFTGRAEEGERLVRPLRECGTPL ADTVGEMPYTAVADIHQDPVHPLPYHERNIVLRELGSDAMEALLRAAGAGSSCGDLMVEVRHLGGALSRPAEVPNAVGARDGAFTLATLSPPDSPDVVLG AMEPWGTGRRYLNFLAGADTAGSAAECFDPETLGRLARVKAAVDPGNLFRLNHNIAPLP', 'http://blast.ncbi.nlm.nih.gov/Blast.cgi?PAGE=Proteins&PROGRAM=blastp&BLAST_PROGRAMS=blastp&QUERY=MIVAGQTGHAGIAESALDGLVARLRGEVFTPRDAGYERELAGFNRIGRHRPDVIVAAQSAGDVSAAVSFAAREALPVAVQATGHGIAASARGGVLVSTRR MNAVAVDPLTRTAHVEAGAQWHQVIAAGAPHGLAPLNGSSPLVGVVGYTLGGGLGPLGRQYGYAADHVTCIEVVTADGSILRVTRDRHPDLFWALRGGKG NFGIVTGLWFGLVPVRRLHGGGIYFPGEQAAQALHTWRQWTATVPEEMTSSVALLRLPDVPDVPEFLRGTFAAHVRIAFTGRAEEGERLVRPLRECGTPL ADTVGEMPYTAVADIHQDPVHPLPYHERNIVLRELGSDAMEALLRAAGAGSSCGDLMVEVRHLGGALSRPAEVPNAVGARDGAFTLATLSPPDSPDVVLG AMEPWGTGRRYLNFLAGADTAGSAAECFDPETLGRLARVKAAVDPGNLFRLNHNIAPLP&LINK_LOC=protein&PAGE_TYPE=BlastSearch', 'BLAST this protein','SGR_4241')" />

 
 
   RRFGWVDITADWAPATEPPLNFSHTALRREARDFLHDLIADLVDLHDGLADNPAIWNLQARFPRV', 'http://blast.ncbi.nlm.nih.gov/Blast.cgi?PAGE=Proteins&PROGRAM=blastp&BLAST_PROGRAMS=blastp&QUERY=MIELGYSLSHRFPDPPQTDYRSAPVQALRHDLFCGDVYLADTAADRELSTAWGWLPVLDFAWALCDIVELLDRDPRGSRSATPVHAELDFTESTDRLRFE RRFGWVDITADWAPATEPPLNFSHTALRREARDFLHDLIADLVDLHDGLADNPAIWNLQARFPRV&LINK_LOC=protein&PAGE_TYPE=BlastSearch', 'BLAST this protein','Strvi_8805')" />

 
 
   VNPDGWLATAGHCVDPQSARDLIFEHAASDYVKQFPDSPEALNPVGAVQWLRKNARVEGETAEQGPEVSITVLYGSGAKVAATMNADVVDFRPIDKGDVA LLRVDRRNMPSSELTTDADVSIGTRILAVGYPKSTQDITDPSLDPTYKSGTVSKKSLRQTIPEYEIDAPISDGMSGGPTIELNGKVIGLNSFRPASESQP FNFVAPVAGLAALLAAKGVVPTLGPADLSYRKGLDLYYSGHYSAAIKEFDTALSMSPDFPGLVDLRTSAVNLRQQYGDVSVLRGSNLMWYIGLVVVLAVG GGVTFTVLRSRRQRSAASPVPDFQPIPDGWSPVGGGAEGAGEHAADAAAPADAEHLTAGEPHFCANCGAQHHHTEKFCPSCGEPIVIDASAHGKHEKS', 'http://blast.ncbi.nlm.nih.gov/Blast.cgi?PAGE=Proteins&PROGRAM=blastp&BLAST_PROGRAMS=blastp&QUERY=MVSRARPARLGSGPVGGRIFALLAVAALLMAGSPGIAYADDGRPQANPEERAAALIRPAVMYIVIGAYGWVRLPTGQRLSHYGDNPTKPFTAGWGCTAFT VNPDGWLATAGHCVDPQSARDLIFEHAASDYVKQFPDSPEALNPVGAVQWLRKNARVEGETAEQGPEVSITVLYGSGAKVAATMNADVVDFRPIDKGDVA LLRVDRRNMPSSELTTDADVSIGTRILAVGYPKSTQDITDPSLDPTYKSGTVSKKSLRQTIPEYEIDAPISDGMSGGPTIELNGKVIGLNSFRPASESQP FNFVAPVAGLAALLAAKGVVPTLGPADLSYRKGLDLYYSGHYSAAIKEFDTALSMSPDFPGLVDLRTSAVNLRQQYGDVSVLRGSNLMWYIGLVVVLAVG GGVTFTVLRSRRQRSAASPVPDFQPIPDGWSPVGGGAEGAGEHAADAAAPADAEHLTAGEPHFCANCGAQHHHTEKFCPSCGEPIVIDASAHGKHEKS&LINK_LOC=protein&PAGE_TYPE=BlastSearch', 'BLAST this protein','MMAR_0916')" />

 
 
   GGLWYAGELSGKPFTGFTSAINMHGGNEATLLSLYHVAFHWGSVIVPPGYNDPAVPAAYGNPYGASYPSGMDGEPPNERVLAAARFQGYNLATVTARLLN GARG', 'http://blast.ncbi.nlm.nih.gov/Blast.cgi?PAGE=Proteins&PROGRAM=blastp&BLAST_PROGRAMS=blastp&QUERY=MSVKVAVIYYSSTGNVHALAQAVREGAEKADAEVRFRRVDELAPDSAIDANPAWRAHADATKDVEPATHEDLKWADAYAFGTPVRFGDVASQLKQFIDST GGLWYAGELSGKPFTGFTSAINMHGGNEATLLSLYHVAFHWGSVIVPPGYNDPAVPAAYGNPYGASYPSGMDGEPPNERVLAAARFQGYNLATVTARLLN GARG&LINK_LOC=protein&PAGE_TYPE=BlastSearch', 'BLAST this protein','SACE_2881')" />
   ELWNQGRLVNKVVSSFTSTSTSHGGQESTLLALNNTFYHWGAIIVAPGYADPIQFSAANGNPYGTSHVSHDTTPPGEEKLDAIDFQARRAVEIATALNIG LKAL', 'http://blast.ncbi.nlm.nih.gov/Blast.cgi?PAGE=Proteins&PROGRAM=blastp&BLAST_PROGRAMS=blastp&QUERY=MTNVAVIYYSSTGHVHKMAEAAAVSAEKAGAEVRLRRVAETASESVVAASPEWSEHVAATGGIATATLEDLEWADVLLFGTPTRFGLPAAQLKQFIDSTG ELWNQGRLVNKVVSSFTSTSTSHGGQESTLLALNNTFYHWGAIIVAPGYADPIQFSAANGNPYGTSHVSHDTTPPGEEKLDAIDFQARRAVEIATALNIG LKAL&LINK_LOC=protein&PAGE_TYPE=BlastSearch', 'BLAST this protein','SCAB_12131')" />

 
 
   HLAVHGTVNDLAVSGARPQWLSAAFVIEEGFPIAELREIVADMSEAAVLSGVQIVTGDTKVVGKGAADGVYISTAGVGVIPEGRRLSPDLVRAGDKVLLS GTIGAHGMAVMLARGDLAIEADIASDTAPVNALVETLLEAAPSTRWMRDATRGGVGTVCNELAHACQLAVIVDEQSLPIEPQVLGACDMLGIDPLYVANE GKFVAVVAADEADAAVAALRAHPRGADATVVGEILAEPPGIVALRTSFGGSRIVDMLVGDPLPRIC', 'http://blast.ncbi.nlm.nih.gov/Blast.cgi?PAGE=Proteins&PROGRAM=blastp&BLAST_PROGRAMS=blastp&QUERY=MSTTEPATSAEREDRVLERIDKFRQRRPRLLDEVVTLAHGAGGKSSAALVDAVFVEAFRNEELEQLGDAAALTMPSGERLAFSTDSYVVQPLRFPGGSIG HLAVHGTVNDLAVSGARPQWLSAAFVIEEGFPIAELREIVADMSEAAVLSGVQIVTGDTKVVGKGAADGVYISTAGVGVIPEGRRLSPDLVRAGDKVLLS GTIGAHGMAVMLARGDLAIEADIASDTAPVNALVETLLEAAPSTRWMRDATRGGVGTVCNELAHACQLAVIVDEQSLPIEPQVLGACDMLGIDPLYVANE GKFVAVVAADEADAAVAALRAHPRGADATVVGEILAEPPGIVALRTSFGGSRIVDMLVGDPLPRIC&LINK_LOC=protein&PAGE_TYPE=BlastSearch', 'BLAST this protein','RHA1_ro04620')" />
   ERIERVRRRKARVREDRITLAHGAGGKATHTLIEAVFLEAFRNPLLEPLEDGAVVDGLAFTTDSYVVTPLFFPGGDIGDLAVNGTVNDLAMCGARPRYLS AAFIVEEGFPVADLRRITASMAAAARAADVWIVTGDTKVVENGKADGCYITTSGVGTVERPIRLSAAAARPGDVVIVSGPIGEHGVTVMLARGELDIQAD LTSDTAPLNTLTGRLLDACGDGQVRCLRDATRGGVATVVNEIAVASKVAVVLEEDAIPVCPAVRGACELLGIDPLYVACEGRMVAVVAQRSAEAALAALR SHPLGSEAAVIGRIGDDPPGLVLLKTSFGGTRIVDLLVGDPLPRIC', 'http://blast.ncbi.nlm.nih.gov/Blast.cgi?PAGE=Proteins&PROGRAM=blastp&BLAST_PROGRAMS=blastp&QUERY=MNDEEDSSLGEGTAARELREGTAPREMGPAGRAGSETRDPVVTALPGRKGPDSVVTAFPGRDSGEAVFPGTEGPDSRVTALPMERGPRGSTATSREQEVL ERIERVRRRKARVREDRITLAHGAGGKATHTLIEAVFLEAFRNPLLEPLEDGAVVDGLAFTTDSYVVTPLFFPGGDIGDLAVNGTVNDLAMCGARPRYLS AAFIVEEGFPVADLRRITASMAAAARAADVWIVTGDTKVVENGKADGCYITTSGVGTVERPIRLSAAAARPGDVVIVSGPIGEHGVTVMLARGELDIQAD LTSDTAPLNTLTGRLLDACGDGQVRCLRDATRGGVATVVNEIAVASKVAVVLEEDAIPVCPAVRGACELLGIDPLYVACEGRMVAVVAQRSAEAALAALR SHPLGSEAAVIGRIGDDPPGLVLLKTSFGGTRIVDLLVGDPLPRIC&LINK_LOC=protein&PAGE_TYPE=BlastSearch', 'BLAST this protein','Sros_3824')" />

 
 
   NQVQNSTSTLQAAAKTLKTDATIPGTAWAVDPKTNEIRVTADSTVKGAKWDTLTATVKSLGSGVATVKKSAGTFKTFVSGGDAIFGGGARCSLGFNVTAG DGSPAFLTAGHCGVAAAAWSDSQNGQPIATVDQATFPGEGDFSLVKYDDPNTQAPSEVNVGNGQTVQISQAAEATVGQQVLRMGSTTGLNDGNVTGLDAT VNYPEGTVTGLIQTDVCAEPGDSGGSLFTQDGSAIGLTSGGSGDCTVGGETFFQPVTTALEAVGATLGAGGAAGAGDESAAPGDEATDPAGEATDPAGEA TDPAGEATDPAGEATDPADQSGNDGAEQSGNDGADQSGNDGADDGSGLVSSN', 'http://blast.ncbi.nlm.nih.gov/Blast.cgi?PAGE=Proteins&PROGRAM=blastp&BLAST_PROGRAMS=blastp&QUERY=MSHKRIPKRKAAIAAGSVAALGAAALLLPNANASQTDSGDSTPRTFKSADVSDLASQLASQLGDAFAGSYYNADKQQLVINVVGDDSNVINQIRKAGAVP NQVQNSTSTLQAAAKTLKTDATIPGTAWAVDPKTNEIRVTADSTVKGAKWDTLTATVKSLGSGVATVKKSAGTFKTFVSGGDAIFGGGARCSLGFNVTAG DGSPAFLTAGHCGVAAAAWSDSQNGQPIATVDQATFPGEGDFSLVKYDDPNTQAPSEVNVGNGQTVQISQAAEATVGQQVLRMGSTTGLNDGNVTGLDAT VNYPEGTVTGLIQTDVCAEPGDSGGSLFTQDGSAIGLTSGGSGDCTVGGETFFQPVTTALEAVGATLGAGGAAGAGDESAAPGDEATDPAGEATDPAGEA TDPAGEATDPAGEATDPADQSGNDGAEQSGNDGADQSGNDGADDGSGLVSSN&LINK_LOC=protein&PAGE_TYPE=BlastSearch', 'BLAST this protein','SAV_7497')" />
   KGAATLKAKAAVPGTAWAIDPRTNKIQVTADSTVTGENWDTIESATKSLGTGMATIKKSAGTFKPFLEGGDAIFGGGARCSAGFNVVNAEGAPAFLTAGH CGVAEAEWSEEEGGAPIATVDAATATFPGAGDFALVNYNDPATQAASTVDLGNGQTVDIKAAGEAEVGLQVFRMGSTTGLADGQVTGLEATVNYPEGTVT GLIQTDVCAEPGDSGGSLFTQDGQAIGLTSGGSGDCTVGGETFFQPVTTALAAVGATLGDAGAGAGEQADGGAAAGAGEQADGGAAAGAGQEEGAAAAGA GEEEGAAAAGAGEEQGAAAGAGDDAGAGQQHDQGAGDQGGEQQQGAEQQGAEQQGVQR', 'http://blast.ncbi.nlm.nih.gov/Blast.cgi?PAGE=Proteins&PROGRAM=blastp&BLAST_PROGRAMS=blastp&QUERY=MAALGAAALILPNAMASQTDAKGAAPRTLAASDASDLASKLREALGDAFAGAYYDSAEKQLVINVIDGLQIDGDDNNVIIQAQSAGAKVREVDNSWSELQ KGAATLKAKAAVPGTAWAIDPRTNKIQVTADSTVTGENWDTIESATKSLGTGMATIKKSAGTFKPFLEGGDAIFGGGARCSAGFNVVNAEGAPAFLTAGH CGVAEAEWSEEEGGAPIATVDAATATFPGAGDFALVNYNDPATQAASTVDLGNGQTVDIKAAGEAEVGLQVFRMGSTTGLADGQVTGLEATVNYPEGTVT GLIQTDVCAEPGDSGGSLFTQDGQAIGLTSGGSGDCTVGGETFFQPVTTALAAVGATLGDAGAGAGEQADGGAAAGAGEQADGGAAAGAGQEEGAAAAGA GEEEGAAAAGAGEEQGAAAGAGDDAGAGQQHDQGAGDQGGEQQQGAEQQGAEQQGVQR&LINK_LOC=protein&PAGE_TYPE=BlastSearch', 'BLAST this protein','SCAB_8661')" />

 
 
   FTCRAWRGEPRAAEAKAADVRWFPLDALPEPVVPHERWVLDQLARGAVPPPVSEFGFTPDGRRPTVSS', 'http://blast.ncbi.nlm.nih.gov/Blast.cgi?PAGE=Proteins&PROGRAM=blastp&BLAST_PROGRAMS=blastp&QUERY=MIVTDRFRLVPAAYVLLLRTGPRGEEVLLQLRRGTGFMDGHWAAAAAGHVEEAESVTRAAVREAAEELGVVVDPADLVALTAMHRTRATGLPVDERVDFF FTCRAWRGEPRAAEAKAADVRWFPLDALPEPVVPHERWVLDQLARGAVPPPVSEFGFTPDGRRPTVSS&LINK_LOC=protein&PAGE_TYPE=BlastSearch', 'BLAST this protein','Psed_2127')" />
   PHAGGTDQYAGYLENEDGFEVELVADRDRSGESRTT', 'http://blast.ncbi.nlm.nih.gov/Blast.cgi?PAGE=Proteins&PROGRAM=blastp&BLAST_PROGRAMS=blastp&QUERY=MLHHIELWVPDLGRAVRSWGWLLGELGYQSFQEWSVGRSWALDGVYIVVEQSPAMTAPSHDRCRPGLNHLAFRVIGSERVDALVAAAPEHGWTLMFTDRH PHAGGTDQYAGYLENEDGFEVELVADRDRSGESRTT&LINK_LOC=protein&PAGE_TYPE=BlastSearch', 'BLAST this protein','Sros_0069')" />

 
 
   TPSKVRGPMAAGVTAVAAERSLGQLEALGARLDAAGPSAAVATWYVDVPTNKLVVESVGDTAAAADAVAAAGLPADAVTLATTEAPRTFVDVIGGNAYYI NASSRCSVGFAVEGGFVTAGHCGRAGASTSSPSGTFRGSSFPGNDYAWVQVASGNTPRGLVNNHSGGTVRVTGSQQAAVGSYVCRSGSTTGWRCGYVRAY NTTVRYAEGSVSGLIRTSVCAEPGDSGGSLVAGTQAQGVTSGGSGNCRYGGTTYFQPVNEILQAYGLRLVLG', 'http://blast.ncbi.nlm.nih.gov/Blast.cgi?PAGE=Proteins&PROGRAM=blastp&BLAST_PROGRAMS=blastp&QUERY=MATPTTLRRASAVATAALALLLAPLTTHGASAAAPSSDLPAGMVEALERDLGVPRAEAAQRLAFQKDAATKVRTLTQALGSTYAGAWVDPAAGTVHVAST TPSKVRGPMAAGVTAVAAERSLGQLEALGARLDAAGPSAAVATWYVDVPTNKLVVESVGDTAAAADAVAAAGLPADAVTLATTEAPRTFVDVIGGNAYYI NASSRCSVGFAVEGGFVTAGHCGRAGASTSSPSGTFRGSSFPGNDYAWVQVASGNTPRGLVNNHSGGTVRVTGSQQAAVGSYVCRSGSTTGWRCGYVRAY NTTVRYAEGSVSGLIRTSVCAEPGDSGGSLVAGTQAQGVTSGGSGNCRYGGTTYFQPVNEILQAYGLRLVLG&LINK_LOC=protein&PAGE_TYPE=BlastSearch', 'BLAST this protein','Cfla_2899')" />

 
 
   VDAEPEVLVATRCPGCGQPHALVVNRTVPPEGVQVAHFLVPAARMWDDVLHTCANQRLFCCESCVDAWLAETGQPKGSVLDLVTLWRLARGWYAGRLERG YRRREPDDAVAYFAEAGLTGPFWAATAGV', 'http://blast.ncbi.nlm.nih.gov/Blast.cgi?PAGE=Proteins&PROGRAM=blastp&BLAST_PROGRAMS=blastp&QUERY=MRDDDASWDEDVRLAVYRAFARHGRAPSGPELADAAGGSLAVAKQALHRLADAHHLVLDECEHVVLAHPFAAKSLGFSVMGAHTLWWGGCAWDSFAIPHL VDAEPEVLVATRCPGCGQPHALVVNRTVPPEGVQVAHFLVPAARMWDDVLHTCANQRLFCCESCVDAWLAETGQPKGSVLDLVTLWRLARGWYAGRLERG YRRREPDDAVAYFAEAGLTGPFWAATAGV&LINK_LOC=protein&PAGE_TYPE=BlastSearch', 'BLAST this protein','AMED_2325')" />
   VDAEPEVLVATRCPGCGQPHALVVNRTVPPEGVQVAHFLVPAARMWDDVLHTCANQRLFCCESCVDAWLAETGQPKGSVLDLVTLWRLARGWYAGRLERG YRRREPDDAVAYFAEAGLTGPFWAATAGV', 'http://blast.ncbi.nlm.nih.gov/Blast.cgi?PAGE=Proteins&PROGRAM=blastp&BLAST_PROGRAMS=blastp&QUERY=MRDDDASWDEDVRLAVYRAFARHGRAPSGPELADAAGGSLAVAKQALHRLADAHHLVLDECEHVVLAHPFAAKSLGFSVMGAHTLWWGGCAWDSFAIPHL VDAEPEVLVATRCPGCGQPHALVVNRTVPPEGVQVAHFLVPAARMWDDVLHTCANQRLFCCESCVDAWLAETGQPKGSVLDLVTLWRLARGWYAGRLERG YRRREPDDAVAYFAEAGLTGPFWAATAGV&LINK_LOC=protein&PAGE_TYPE=BlastSearch', 'BLAST this protein','RAM_11835')" />

 
 
   SDGALGAVVLADTRRLEDCFPAVDFFERRDINFIVAVNEFDGAYRYDTDEVRTALDLKPHIPVVHCDARDSHSATSVLVAFLRHLLLTATSRPGSIVPS', 'http://blast.ncbi.nlm.nih.gov/Blast.cgi?PAGE=Proteins&PROGRAM=blastp&BLAST_PROGRAMS=blastp&QUERY=MAYNDDSEAQFPAAIKFLIAGGFGVGKTTFVGAVSEIEPLSTEELITSASIGTDSLDGVDAKSTTTVAMDFGRITLDPERVLYLFGTPGQDRFWFMWDEL SDGALGAVVLADTRRLEDCFPAVDFFERRDINFIVAVNEFDGAYRYDTDEVRTALDLKPHIPVVHCDARDSHSATSVLVAFLRHLLLTATSRPGSIVPS&LINK_LOC=protein&PAGE_TYPE=BlastSearch', 'BLAST this protein','Snas_1148')" />

 
 
   PEAVLAAQQAAFAPVLAAALAPIDPAELGELAELARAAGQAAAANREGRPLLAGLAALPWPREDHLVIWHAAKVLREHRGDGHIAGLVLEGLRGIDALVI HAAYEGWPGELLRDSRRWDPPAWDASVASLRRRGWLTEAATPEVTPEVTPELTAAVTPELTAAVTPELTAEGRRRRRWIEDRTDQLAALAYAPIGDAGMD RMIELGAKVVAALGAAGLATRLRRPVAR', 'http://blast.ncbi.nlm.nih.gov/Blast.cgi?PAGE=Proteins&PROGRAM=blastp&BLAST_PROGRAMS=blastp&QUERY=MTTEGNLLTGHEADGGVDVARTRPALLAIEPTFGAGVLCPAVHAALDRLDLGLDDVAVVNLVTRAALLGPASADVVVATFYNVNPRLVESVIPAVWRKAS PEAVLAAQQAAFAPVLAAALAPIDPAELGELAELARAAGQAAAANREGRPLLAGLAALPWPREDHLVIWHAAKVLREHRGDGHIAGLVLEGLRGIDALVI HAAYEGWPGELLRDSRRWDPPAWDASVASLRRRGWLTEAATPEVTPEVTPELTAAVTPELTAAVTPELTAEGRRRRRWIEDRTDQLAALAYAPIGDAGMD RMIELGAKVVAALGAAGLATRLRRPVAR&LINK_LOC=protein&PAGE_TYPE=BlastSearch', 'BLAST this protein','FRAAL_4066')" />
   VAGLEEMVTLAASAAAGVSPGGRVLGAANQALPPPVEPHLALWQSCSTLRESRGDGHVAALVAAGLAPCEALVLFTADKGLDPAYMRRMRGWSEQEWKEA EAALVDRRLLTGGGLTVAGRELRAEVERWTDMAAAAPWQALGAGPTARLRDLMTPVVQRLSELNESMKVNPMALNPAEIVA', 'http://blast.ncbi.nlm.nih.gov/Blast.cgi?PAGE=Proteins&PROGRAM=blastp&BLAST_PROGRAMS=blastp&QUERY=MSREMWLRFEIYHDVVYAPEASLVAKELGCRGGWMGYFGTRSAPLGAASPEVVTAAFFNFHPQMVAKAVPQVWSVASPERFLQARLEIVDAALRRMLGAD VAGLEEMVTLAASAAAGVSPGGRVLGAANQALPPPVEPHLALWQSCSTLRESRGDGHVAALVAAGLAPCEALVLFTADKGLDPAYMRRMRGWSEQEWKEA EAALVDRRLLTGGGLTVAGRELRAEVERWTDMAAAAPWQALGAGPTARLRDLMTPVVQRLSELNESMKVNPMALNPAEIVA&LINK_LOC=protein&PAGE_TYPE=BlastSearch', 'BLAST this protein','Strop_1025')" />

 
 
   HLFGGLLADKSRQGGDLTVVSHNVGADNPDAVGTARALAAAGADVLALEELDPADRRTYERELATAYPHHTVLGTVGVWSRLPLSDTRPVDVAMDAGPLG DAKPAEVKLAYNRGLRTTVATDHGPLAVYVAHLGSVRVNPRAGLSSGQRDAGARSLGRAVAAERNERVVLLGDLNGTLDDRAYAGITSRMRSAQEVAGDG FGFSWPATFPVARIDQILVRGVEPESSWLLPATGSDHLPVAARVSW', 'http://blast.ncbi.nlm.nih.gov/Blast.cgi?PAGE=Proteins&PROGRAM=blastp&BLAST_PROGRAMS=blastp&QUERY=MNRRPPSAWTALRRSRALLAVGAAAGPWKRGRGLAVAALLLGLLLPLHASIPNGVGNLGSLVETFLPWFGLLIPVLLAGALLRRSLLAGAALLLPAVVWL HLFGGLLADKSRQGGDLTVVSHNVGADNPDAVGTARALAAAGADVLALEELDPADRRTYERELATAYPHHTVLGTVGVWSRLPLSDTRPVDVAMDAGPLG DAKPAEVKLAYNRGLRTTVATDHGPLAVYVAHLGSVRVNPRAGLSSGQRDAGARSLGRAVAAERNERVVLLGDLNGTLDDRAYAGITSRMRSAQEVAGDG FGFSWPATFPVARIDQILVRGVEPESSWLLPATGSDHLPVAARVSW&LINK_LOC=protein&PAGE_TYPE=BlastSearch', 'BLAST this protein','SGR_273')" />

 
 
   TVLDKVGKVYQKWRGTAVIEIKSGKMLAARGENVPLTAIDLTKLSRSDGLDPRMVRLENGQTRLIIVALLSWKGQPQQLLVASSTLSFPGVDLGPGRAIG VVDSTGHLLSSHGALESERARKQLTSFAKTAARKGRQHPVKAKEPGAGGYTGVSGHLAGGAAKEVRTVGGYATLAAPQAGGATTASSLGLTVVTEVDVSA KVSQITRPAFGVAAAGALLVIGGLAVVVLLGTVQRPLIRLFLESRRLTRGDLARPVSVPRAGEAARVGAALERLRGQLQGEPADVEGPAVRKGLGARALL AVCAVLLLVWSVPLMLLLNRADSAVKVPVQIVHDQNSRTITLSDRVRRALNEGHADLSAVAALIGDRTTPEDMKQVLERTMQDHDRYESVYVLGADGEIL ARAGHSPHHADGKGPSKQALEVTGEGRKKPVVTATAQAPGRGGAAVVGEFRIEFVNALLGRQGMGEVRVVDTKGRVIGGDSGYIAFEKAPSHLSSLLATK RAHATVQRGGTVRVAAVAPFSGGGAAKSLQWSLASWQPAAGLAIPEYSLQNRTVLAGLLGLTAAAACLGWLHIVVVRPLRALAQLAEALAAGDRKTVLFP RHHDEVGAVVRSLELIRQQLQEQPRKRDGGSRTPAGVGRN', 'http://blast.ncbi.nlm.nih.gov/Blast.cgi?PAGE=Proteins&PROGRAM=blastp&BLAST_PROGRAMS=blastp&QUERY=MAASRSKRRHKVRRRADMSLLGGIRPPIAVLSVLLLSLAGITALTLGKPDNALVPKAVLTSQQYVAEDGAIALRASLDESVTDLTSTATLFNEGRPVSAD TVLDKVGKVYQKWRGTAVIEIKSGKMLAARGENVPLTAIDLTKLSRSDGLDPRMVRLENGQTRLIIVALLSWKGQPQQLLVASSTLSFPGVDLGPGRAIG VVDSTGHLLSSHGALESERARKQLTSFAKTAARKGRQHPVKAKEPGAGGYTGVSGHLAGGAAKEVRTVGGYATLAAPQAGGATTASSLGLTVVTEVDVSA KVSQITRPAFGVAAAGALLVIGGLAVVVLLGTVQRPLIRLFLESRRLTRGDLARPVSVPRAGEAARVGAALERLRGQLQGEPADVEGPAVRKGLGARALL AVCAVLLLVWSVPLMLLLNRADSAVKVPVQIVHDQNSRTITLSDRVRRALNEGHADLSAVAALIGDRTTPEDMKQVLERTMQDHDRYESVYVLGADGEIL ARAGHSPHHADGKGPSKQALEVTGEGRKKPVVTATAQAPGRGGAAVVGEFRIEFVNALLGRQGMGEVRVVDTKGRVIGGDSGYIAFEKAPSHLSSLLATK RAHATVQRGGTVRVAAVAPFSGGGAAKSLQWSLASWQPAAGLAIPEYSLQNRTVLAGLLGLTAAAACLGWLHIVVVRPLRALAQLAEALAAGDRKTVLFP RHHDEVGAVVRSLELIRQQLQEQPRKRDGGSRTPAGVGRN&LINK_LOC=protein&PAGE_TYPE=BlastSearch', 'BLAST this protein','Strvi_3274')" />
   RREELTRMVQTRGETSDAELLNRLISGGRNISGVLIMDAGTRQVIAAKGAELPLDLLPAELPIGSTLAVTNADGPLMVYGIALDETRVVLATQPLTMRNL RLNPDAGHGIHALTPDGTISLMQGANAVEAAHLPAIFEGLVDAGSRQSRQVVVKEWSDRRLLVSSAPVGSSGLVIVSLLTTEVSTGTSLRKGLLLGLSLL AVGLLSFWIMRRSLFLPVRVLLSQAKADACGAITARRPKLRIREAHRIARALALTSGEQFPTDRRWRPTVLQGLSVALVIALLWPAAVVVLGLRAPAPTV PVQLMRDEENRAEEASSALGNLLDGGLVTVSRTSYGFNVQDLEQAGRRLDRELDTDDRLRALYLVDRDGKVLAGAGRRPLRTVEPLPGEIGIHLDRTVQR LPVVYAYNQMADGYSIVGEFDPDRLLGLVRRVSGRTYVVDAELRTVLDSEGFQAFQPLQGDLVREAAVEALPGGTVGRSRTADGKPALVAAAGLSAPGTV AHLEWAVVIEQDTSGLRLPELIEQRWTLLMAGAAVGVVLLTHVWQLYIFVRPLRRLANVADRMSEGNVELPVPPQRHDDIGAIAMCLEICRQVRHTGSAR FGGALRLRGAGADRTTVLPRVRSAAGTGRGTKG', 'http://blast.ncbi.nlm.nih.gov/Blast.cgi?PAGE=Proteins&PROGRAM=blastp&BLAST_PROGRAMS=blastp&QUERY=MRSGSAQGAPQQPIDGSVNDQAQLVAWRRRALEQQLPAAAFPAGTNAPSLVYLLATMFVVVAAVLGFAVNAQHGALPAVVDSQRDIVAKLASSIRLDAAA RREELTRMVQTRGETSDAELLNRLISGGRNISGVLIMDAGTRQVIAAKGAELPLDLLPAELPIGSTLAVTNADGPLMVYGIALDETRVVLATQPLTMRNL RLNPDAGHGIHALTPDGTISLMQGANAVEAAHLPAIFEGLVDAGSRQSRQVVVKEWSDRRLLVSSAPVGSSGLVIVSLLTTEVSTGTSLRKGLLLGLSLL AVGLLSFWIMRRSLFLPVRVLLSQAKADACGAITARRPKLRIREAHRIARALALTSGEQFPTDRRWRPTVLQGLSVALVIALLWPAAVVVLGLRAPAPTV PVQLMRDEENRAEEASSALGNLLDGGLVTVSRTSYGFNVQDLEQAGRRLDRELDTDDRLRALYLVDRDGKVLAGAGRRPLRTVEPLPGEIGIHLDRTVQR LPVVYAYNQMADGYSIVGEFDPDRLLGLVRRVSGRTYVVDAELRTVLDSEGFQAFQPLQGDLVREAAVEALPGGTVGRSRTADGKPALVAAAGLSAPGTV AHLEWAVVIEQDTSGLRLPELIEQRWTLLMAGAAVGVVLLTHVWQLYIFVRPLRRLANVADRMSEGNVELPVPPQRHDDIGAIAMCLEICRQVRHTGSAR FGGALRLRGAGADRTTVLPRVRSAAGTGRGTKG&LINK_LOC=protein&PAGE_TYPE=BlastSearch', 'BLAST this protein','VAB18032_05485')" />

 
 
   SALGYHGVSMEAIASRVGISAAALYRHYSSKYELFRDAVLNLGQQLVDCTAFADTADAPENSEDAREQLRLLAAALTDTSLANRESGGLYRWEARYLNED DQAALNAQMRTVHHRIQRPLQQLRPELISRERWMLSTATLSVIGSVVDHRGKLPAGQIREVLGEIVESVLAADLPELPELGAAAAPATTPVVSAAKYEAL LNESMRLFNKNGYRDTTMEDIAAAVGMPASGIYRYFSGKADILAAGFRRAADRLSADMAEILAAGGEPEDVLAALIDDYVTRSFDRPELEHVYYTERLNM TPADQKILRDLQRSAVESWVNVVMPVRPHWSAGQARFAVHAAMALVIDLGRLMGYRNSEQARAVVSTMVDLVLLGRYRLRTALPAR', 'http://blast.ncbi.nlm.nih.gov/Blast.cgi?PAGE=Proteins&PROGRAM=blastp&BLAST_PROGRAMS=blastp&QUERY=MHAPENSSTGQGAMALRNDTTASRLSMLRGWSMCVLDQMTPTYVNDRSPWVRAFDRIGGPVYRDHMARTPLGPTTSGSVAVRRRPKDRKAQIARASAEAF SALGYHGVSMEAIASRVGISAAALYRHYSSKYELFRDAVLNLGQQLVDCTAFADTADAPENSEDAREQLRLLAAALTDTSLANRESGGLYRWEARYLNED DQAALNAQMRTVHHRIQRPLQQLRPELISRERWMLSTATLSVIGSVVDHRGKLPAGQIREVLGEIVESVLAADLPELPELGAAAAPATTPVVSAAKYEAL LNESMRLFNKNGYRDTTMEDIAAAVGMPASGIYRYFSGKADILAAGFRRAADRLSADMAEILAAGGEPEDVLAALIDDYVTRSFDRPELEHVYYTERLNM TPADQKILRDLQRSAVESWVNVVMPVRPHWSAGQARFAVHAAMALVIDLGRLMGYRNSEQARAVVSTMVDLVLLGRYRLRTALPAR&LINK_LOC=protein&PAGE_TYPE=BlastSearch', 'BLAST this protein','MSMEG_0815')" />
   ASGGVYRWEARYLNHEDRRQLRAKFRHVIGRVDEAVQRERPLPDGRLRAVAALGAIGSITMHHTSIAQRRAEELLVESALRVAAADPAAPRPGARTVEVV AHPVPRTRRAEILAAAIPLFARDGFASVTNGQIAEAVGLAPSALYRHYSGKVDILAAACLQAAGLLAREVDQSLHEVSGPHDAIGALAATYVAYSFEYTA LNSVAEAELVGLPADLRRPLVLAQREHIAVWEQQLRLARPELDPRQARVLVHAGFGVAVEAGRGLRWQDGPDHRDAVTALVMGALGF', 'http://blast.ncbi.nlm.nih.gov/Blast.cgi?PAGE=Proteins&PROGRAM=blastp&BLAST_PROGRAMS=blastp&QUERY=MTADPAAATRPRNRKQLIVEAAGRIFSERGYHMASMEKIAAGVGITAAALYRHFPNKYALFAECADLMADRLVAAVDEVPSGAPSADVLTAVTRVTVAHR ASGGVYRWEARYLNHEDRRQLRAKFRHVIGRVDEAVQRERPLPDGRLRAVAALGAIGSITMHHTSIAQRRAEELLVESALRVAAADPAAPRPGARTVEVV AHPVPRTRRAEILAAAIPLFARDGFASVTNGQIAEAVGLAPSALYRHYSGKVDILAAACLQAAGLLAREVDQSLHEVSGPHDAIGALAATYVAYSFEYTA LNSVAEAELVGLPADLRRPLVLAQREHIAVWEQQLRLARPELDPRQARVLVHAGFGVAVEAGRGLRWQDGPDHRDAVTALVMGALGF&LINK_LOC=protein&PAGE_TYPE=BlastSearch', 'BLAST this protein','Sfla_5663')" />

 
 
   VEAGAGVIVVLGGDGTARVAAAACEDVPLLALSTGTNNAFPQMREATVAGLAAGLIATGQIDPDLVTHRVSVLEVVTKARREIALVDVCVSLSRHIGARA LWDPSSLTELYCTFAEPDGIGLSSVPGQLCPSPRSSPDGVALQLGPVGETPYVVHAPIAPGLVRPVGVRGWGLLRPGVRVELAAAGGVIALDGERELELK TGESAFVELKPDGPWVVDVRAAMAEAARKGLLRADTELGKERRR', 'http://blast.ncbi.nlm.nih.gov/Blast.cgi?PAGE=Proteins&PROGRAM=blastp&BLAST_PROGRAMS=blastp&QUERY=MGETVAGIVANPASGRDIRRLVAQASVFPTAEKANMVQRLLAAFAVTGLDRALVSTDLGGISAAVLRALGRGGSWPKVDFCEDDALTGTAADTTNAVRRM VEAGAGVIVVLGGDGTARVAAAACEDVPLLALSTGTNNAFPQMREATVAGLAAGLIATGQIDPDLVTHRVSVLEVVTKARREIALVDVCVSLSRHIGARA LWDPSSLTELYCTFAEPDGIGLSSVPGQLCPSPRSSPDGVALQLGPVGETPYVVHAPIAPGLVRPVGVRGWGLLRPGVRVELAAAGGVIALDGERELELK TGESAFVELKPDGPWVVDVRAAMAEAARKGLLRADTELGKERRR&LINK_LOC=protein&PAGE_TYPE=BlastSearch', 'BLAST this protein','AMED_0343')" />

 
 
   PDHPLRGATPRQLLSHTAGLVSERSETAPAPSLRRYVSSLAPEDRVAEPGTLFSYSNTGYAVAGRLVETVTGQSWWETVESHLCGSTGMELAPVLAPRPF PTADLVTGHAVDGAGRAHPVGFHIEPALAPAGGLAASATALAAFGRAFLDPEHAALDGEVAAPDVLREMGRSVSVADPFGLADGWGAGWSLHLAGDRLWY GHDGTLDGATCNLRVDPEGGSVVALTTNATTGVTAWEHVVRGLRELGVDVGHYRQPDAAAEGTATGPGPGRVTGRYGNGGLVAEVVPGRDGYRLVMSNGF EGALSTGPGGAFRVVSERHGGIALSGRFLADPAEPRRATALQYNGRTLRLSGLAVGPNA', 'http://blast.ncbi.nlm.nih.gov/Blast.cgi?PAGE=Proteins&PROGRAM=blastp&BLAST_PROGRAMS=blastp&QUERY=MPSDSHEENVVRDALSDLLRRTSHQAGVPGAQLAVYHRGELHTCATGVENIQTGRPVAPDSLFPMGSVTKTVTAALVMQLVEDGDVDLDAPVLDHLGGSS PDHPLRGATPRQLLSHTAGLVSERSETAPAPSLRRYVSSLAPEDRVAEPGTLFSYSNTGYAVAGRLVETVTGQSWWETVESHLCGSTGMELAPVLAPRPF PTADLVTGHAVDGAGRAHPVGFHIEPALAPAGGLAASATALAAFGRAFLDPEHAALDGEVAAPDVLREMGRSVSVADPFGLADGWGAGWSLHLAGDRLWY GHDGTLDGATCNLRVDPEGGSVVALTTNATTGVTAWEHVVRGLRELGVDVGHYRQPDAAAEGTATGPGPGRVTGRYGNGGLVAEVVPGRDGYRLVMSNGF EGALSTGPGGAFRVVSERHGGIALSGRFLADPAEPRRATALQYNGRTLRLSGLAVGPNA&LINK_LOC=protein&PAGE_TYPE=BlastSearch', 'BLAST this protein','Ndas_4308')" />

 
 
   AGRATGLNRGRGGSMHAADFGVGILGANAIVGAAAPIATGAAWAARCAGSDRVVVTYFGDGAVSQGVVLETFNMAALWRAPVIFVCENNGFATTTRTQDA VAGSITGRAEAFGIPAERVWGMDPEAVYAATARAVARARSGEGPTLLECETYRYDAHHTWEHAARPRYRTPEEVELGRSVDPLDIQGARIDAGVRARIDA EVDVLLDEAVRFALESPRPDPATALDFLYADGTTARAGAL', 'http://blast.ncbi.nlm.nih.gov/Blast.cgi?PAGE=Proteins&PROGRAM=blastp&BLAST_PROGRAMS=blastp&QUERY=MRSALPGSVAPASASAASPSPDPAALYRTVRLIRRFEERAVELVRAGEVFGGIHPYTGQEAIAAGTCGALRADDLITSTHRGHGHVLAKGADPARMMAEI AGRATGLNRGRGGSMHAADFGVGILGANAIVGAAAPIATGAAWAARCAGSDRVVVTYFGDGAVSQGVVLETFNMAALWRAPVIFVCENNGFATTTRTQDA VAGSITGRAEAFGIPAERVWGMDPEAVYAATARAVARARSGEGPTLLECETYRYDAHHTWEHAARPRYRTPEEVELGRSVDPLDIQGARIDAGVRARIDA EVDVLLDEAVRFALESPRPDPATALDFLYADGTTARAGAL&LINK_LOC=protein&PAGE_TYPE=BlastSearch', 'BLAST this protein','Caci_3259')" />
   GLGVLGANAIVGAAGSIATGAAWAYRRQGRDVVAASFFGDGAMNEGMLLEAFNLAALWQVPVVFVCENNGYATTMPVQSAVAGSITGRARAFGMPAFTVD GQDPEKVLAATSAAVARARSGRGPTFLECTTYRFDAHHTFEHRTRLNYRSTEELSTGRSRDPVEIQGARLAAELRAAVDAEIEALLDAAAGFALGSAHPD PAAALEFLYASPEVQR', 'http://blast.ncbi.nlm.nih.gov/Blast.cgi?PAGE=Proteins&PROGRAM=blastp&BLAST_PROGRAMS=blastp&QUERY=MTADQLYRTVRLIRRFEERAIGFVRSGEIVGGIHPYVGQEAIAAGVCAALRPDDVITSTHRGHGHVLAKGADPARMLAELMGRESGLNKGRGGSMHAADF GLGVLGANAIVGAAGSIATGAAWAYRRQGRDVVAASFFGDGAMNEGMLLEAFNLAALWQVPVVFVCENNGYATTMPVQSAVAGSITGRARAFGMPAFTVD GQDPEKVLAATSAAVARARSGRGPTFLECTTYRFDAHHTFEHRTRLNYRSTEELSTGRSRDPVEIQGARLAAELRAAVDAEIEALLDAAAGFALGSAHPD PAAALEFLYASPEVQR&LINK_LOC=protein&PAGE_TYPE=BlastSearch', 'BLAST this protein','Kfla_6063')" />
   ADFGVGVLGANAIVGAAGAILTGAVWERRRRGADIVGATFFGDGAVNEGMLLEAFNLAALWRIPVLFVCENNGYATTMPVDGAVAGTIAGRAAAFGMPAA TVDGQDPEAVREVTAAAVARMRAGGGPELVEARTYRFDAHHTFEHQVRLDYRPPEEVAEGRSRDPVDIAGARLDPAVRAEVDAAVEAELDAAVDYALAGP HPDPATALDHLYASGLTARTGGG', 'http://blast.ncbi.nlm.nih.gov/Blast.cgi?PAGE=Proteins&PROGRAM=blastp&BLAST_PROGRAMS=blastp&QUERY=MTGSDPLRLYRTVRLIRRFEERAIELVRAGEIVGGIHPYLGQEGIAAGVCAALDREDLVTGTHRGHGHVLAKGADPARMLAELCGRVTGLNRGRGGSMHA ADFGVGVLGANAIVGAAGAILTGAVWERRRRGADIVGATFFGDGAVNEGMLLEAFNLAALWRIPVLFVCENNGYATTMPVDGAVAGTIAGRAAAFGMPAA TVDGQDPEAVREVTAAAVARMRAGGGPELVEARTYRFDAHHTFEHQVRLDYRPPEEVAEGRSRDPVDIAGARLDPAVRAEVDAAVEAELDAAVDYALAGP HPDPATALDHLYASGLTARTGGG&LINK_LOC=protein&PAGE_TYPE=BlastSearch', 'BLAST this protein','Micau_3479')" />
   ADFGVGVLGANAIVGAAGAILTGAVWERRRRGADIVGATFFGDGAVNEGMLLEAFNLAALWRIPVLFVCENNGYATTMPVDGAVAGTIAGRAAAFGMPTA TVDGQDPEAVREVTAAAVVRMRAGGGPELVEARTYRFDAHHTFEHQVRLDYRPPEEVAEGRSRDPVDIAGARLDPAVRAEVDAAVEAELDAAVDYALAGP HPDPATALDHLYASGLTARTGGG', 'http://blast.ncbi.nlm.nih.gov/Blast.cgi?PAGE=Proteins&PROGRAM=blastp&BLAST_PROGRAMS=blastp&QUERY=MTGSDPLRLYRTVRLIRRFEERAIELVRAGEIVGGIHPYLGQEGIAAGVCAALDREDLVTGTHRGHGHVLAKGADPARMLAELCGRVTGLNRGRGGSMHA ADFGVGVLGANAIVGAAGAILTGAVWERRRRGADIVGATFFGDGAVNEGMLLEAFNLAALWRIPVLFVCENNGYATTMPVDGAVAGTIAGRAAAFGMPTA TVDGQDPEAVREVTAAAVVRMRAGGGPELVEARTYRFDAHHTFEHQVRLDYRPPEEVAEGRSRDPVDIAGARLDPAVRAEVDAAVEAELDAAVDYALAGP HPDPATALDHLYASGLTARTGGG&LINK_LOC=protein&PAGE_TYPE=BlastSearch', 'BLAST this protein','ML5_4912')" />
   ADFAVGVLGANAIVGAGGAIVTGAVWARRRRGEDLVGVSFLGDGAVNEGMLLEAFNLAALWRVPVLFVCENNGYATTMPVADSVAGSIPARAEAFGIRAS VVDGQDPAAVHATTAAALARMRAGGGPEFLEARTYRFDAHHTFEHTVRLDYRSAEEVERGRSRDPVRIAGSRLSATDRANVDADVEAVLDVAVAEALAAP EPDPATALEHLYASGLTARTGGG', 'http://blast.ncbi.nlm.nih.gov/Blast.cgi?PAGE=Proteins&PROGRAM=blastp&BLAST_PROGRAMS=blastp&QUERY=MTGVDPVRLYRTVRLIRRFEERAIELVRSGHIVGGIHPYIGQEGIAAGVCAALRPDDVVAGTHRGHGHVLAKGADPARMMAELCGRVTGLNRGRGGSMHA ADFAVGVLGANAIVGAGGAIVTGAVWARRRRGEDLVGVSFLGDGAVNEGMLLEAFNLAALWRVPVLFVCENNGYATTMPVADSVAGSIPARAEAFGIRAS VVDGQDPAAVHATTAAALARMRAGGGPEFLEARTYRFDAHHTFEHTVRLDYRSAEEVERGRSRDPVRIAGSRLSATDRANVDADVEAVLDVAVAEALAAP EPDPATALEHLYASGLTARTGGG&LINK_LOC=protein&PAGE_TYPE=BlastSearch', 'BLAST this protein','Sare_2670')" />
   ADFAVGVLGANAIVGAGGAIVTGAVWARRRRGDDLVGVSFLGDGAVNEGMLLEAFNLAALWRVPVLFVCENNGYATTMPVADAVAGSIPARAEAFGIRTS VVDGQDPAAVQATTAAALTRMRAGGGPEFLEAQTYRFDAHHTFEHAVRLDYRSVEEVERGRSRDPVRIAGSRLSATERAKVDADVEAVLDAAVAEALAAP EPDPATALEHLYASGLTARTGGG', 'http://blast.ncbi.nlm.nih.gov/Blast.cgi?PAGE=Proteins&PROGRAM=blastp&BLAST_PROGRAMS=blastp&QUERY=MTEVGSVRLYRTVRLIRRFEERAIELVRSGHIVGGIHPYVGQEGIAAGVCAALRPDDVVAGTHRGHGHVLAKGADPARMMAELCGRVTGLNRGRGGSMHA ADFAVGVLGANAIVGAGGAIVTGAVWARRRRGDDLVGVSFLGDGAVNEGMLLEAFNLAALWRVPVLFVCENNGYATTMPVADAVAGSIPARAEAFGIRTS VVDGQDPAAVQATTAAALTRMRAGGGPEFLEAQTYRFDAHHTFEHAVRLDYRSVEEVERGRSRDPVRIAGSRLSATERAKVDADVEAVLDAAVAEALAAP EPDPATALEHLYASGLTARTGGG&LINK_LOC=protein&PAGE_TYPE=BlastSearch', 'BLAST this protein','Strop_2487')" />

 
 
   PSENQVLERALRGLERLFQ', 'http://blast.ncbi.nlm.nih.gov/Blast.cgi?PAGE=Proteins&PROGRAM=blastp&BLAST_PROGRAMS=blastp&QUERY=MSFRRRRSNRVRPFTFTGGRTRSRHPLMVQTLVSTSEPGQEPPGTLMPESISIYKLCRETRSLAEVSAELNIPLGVTQVLVSDLAEQDLVYIHPTITGSS PSENQVLERALRGLERLFQ&LINK_LOC=protein&PAGE_TYPE=BlastSearch', 'BLAST this protein','Ndas_0491')" />

 
 
   LDALAAGNRAAGGTPTAGFIDVRDDRTLRLSCAGAMALPYTVIHFADPTKIPLEIVLAAAESAEGKLLTTVADLEEAAIVFDVLERGSDGILYPPRTADE VFALARLLEATTPQLELATLTVESIQHVGLGDRVCVDTCSHFEEDEGILVGSYSSGFILCCSETHPLPYMPTRPFRVNAGALHSYTLGPENRTSYLSEVG SGHALLAVGADGRTRRVVVGRAKLESRPLLEIRAHAEDGQLVSLTVQDDWHVRVLGPGGKVLNVTELRTGDELLGYLATDKRHVGLPIGEFCKES', 'http://blast.ncbi.nlm.nih.gov/Blast.cgi?PAGE=Proteins&PROGRAM=blastp&BLAST_PROGRAMS=blastp&QUERY=MRFAWIDLREVPGPQLQAVVDAAVHTRMAGVLATDAELLATLPPTVTRVLIPGNPATDTAKKPGAKETKETKGADDSKQTKATAGAGWDVLLRTFTTQDE LDALAAGNRAAGGTPTAGFIDVRDDRTLRLSCAGAMALPYTVIHFADPTKIPLEIVLAAAESAEGKLLTTVADLEEAAIVFDVLERGSDGILYPPRTADE VFALARLLEATTPQLELATLTVESIQHVGLGDRVCVDTCSHFEEDEGILVGSYSSGFILCCSETHPLPYMPTRPFRVNAGALHSYTLGPENRTSYLSEVG SGHALLAVGADGRTRRVVVGRAKLESRPLLEIRAHAEDGQLVSLTVQDDWHVRVLGPGGKVLNVTELRTGDELLGYLATDKRHVGLPIGEFCKES&LINK_LOC=protein&PAGE_TYPE=BlastSearch', 'BLAST this protein','SBI_01605')" />
   CDLLLRKFTTQDELDALAAENRGTTGTPVPRTPVAGFVDVRDDRTLRLSCVGAMALPYTVIHFADPTKIPLEIVLAAAESAEGKLVTVVGDLEEAAIVFD VLERGSDGILFTPRSADDVFALARLLEATTPQLEMSTLTVESIRHVGLGDRVCVDTCSHFEEDEGILVGSYSSGFVLCCSETHPLPYMPTRPFRVNAGAL HSYTLGPDNRTSYLSEVGSGSALLAVGADGRTRRVVVGRAKLESRPLLEIRTHAEDGRLVSLTVQDDWHVRVLGPGGKVLNVTELRAGDELLGYLAQDKR HVGLPIGEFCKEV', 'http://blast.ncbi.nlm.nih.gov/Blast.cgi?PAGE=Proteins&PROGRAM=blastp&BLAST_PROGRAMS=blastp&QUERY=MRFAWIDLREVPRPQLQAVVDAAVHARMAGVVSADAELLGTLPPTVTRVLASESRTAAPAKKPADKNAKGDRSDAGQGGGKDTQAADGAGPASGAPAGTG CDLLLRKFTTQDELDALAAENRGTTGTPVPRTPVAGFVDVRDDRTLRLSCVGAMALPYTVIHFADPTKIPLEIVLAAAESAEGKLVTVVGDLEEAAIVFD VLERGSDGILFTPRSADDVFALARLLEATTPQLEMSTLTVESIRHVGLGDRVCVDTCSHFEEDEGILVGSYSSGFVLCCSETHPLPYMPTRPFRVNAGAL HSYTLGPDNRTSYLSEVGSGSALLAVGADGRTRRVVVGRAKLESRPLLEIRTHAEDGRLVSLTVQDDWHVRVLGPGGKVLNVTELRAGDELLGYLAQDKR HVGLPIGEFCKEV&LINK_LOC=protein&PAGE_TYPE=BlastSearch', 'BLAST this protein','SCAB_69861')" />
   HGVTPAELALKHPEIEFGRFVEIIDAPTLEDACESSRTEKWSVLLFRDPTKIPLEIVIAAAARASGSMVTIAQDLEEAEILFGVLEHGSDGVMMAPKTVG DAAELKRIAEAGIPNLNLTELRVVETSHIGMGERACVDTTTHFGEDEGILVGSHSKGMILCVSETHPLPYMPTRPFRVNAGAIHSYTLGRDERTNYLSEL KTGSKLTAVDIKGNTRLVTVGRVKIETRPLISIDAEAPDGRRVNLILQDDWHVRVLGPGGTVLNSTELKPGDTVLGYLPVEDRHVGYPINEFCLEK', 'http://blast.ncbi.nlm.nih.gov/Blast.cgi?PAGE=Proteins&PROGRAM=blastp&BLAST_PROGRAMS=blastp&QUERY=MSSSPSPSPSSSSSSSASSSASSSPSSSSKLTWLDIRSVGEARAAIVQEALHHRVEALVADDPAHLADLPPTVAKVLLVVGKQIPEEFGEATVVVVDPSK HGVTPAELALKHPEIEFGRFVEIIDAPTLEDACESSRTEKWSVLLFRDPTKIPLEIVIAAAARASGSMVTIAQDLEEAEILFGVLEHGSDGVMMAPKTVG DAAELKRIAEAGIPNLNLTELRVVETSHIGMGERACVDTTTHFGEDEGILVGSHSKGMILCVSETHPLPYMPTRPFRVNAGAIHSYTLGRDERTNYLSEL KTGSKLTAVDIKGNTRLVTVGRVKIETRPLISIDAEAPDGRRVNLILQDDWHVRVLGPGGTVLNSTELKPGDTVLGYLPVEDRHVGYPINEFCLEK&LINK_LOC=protein&PAGE_TYPE=BlastSearch', 'BLAST this protein','SGR_4248')" />

 
 
   ADIDLMSGGRLRLGVGAGYNRVEYHAMGVDFASRGRRLTEQISYLRRLWSEELISFEGEFDQIDRANIVPRPTRRIPIWCGGFAEAAFRRAVALADGFVF GYGLDQAATDGWIRLRELLAIAGRPLDGFGAQFVLHAPGQPYTDRQITDGLLRLRAAGATHASLFTMGRGLTGVDRHIDYIAEIMKKAEVALR', 'http://blast.ncbi.nlm.nih.gov/Blast.cgi?PAGE=Proteins&PROGRAM=blastp&BLAST_PROGRAMS=blastp&QUERY=MRLGVVYPQIELEGDPSALARFARAAEGLGYDHLVLYDHVIGASHERRDPPIRGRYGERSPFHDPLTAFAYLAGLTERIEFVSGVLILPLRQTVLVARQT ADIDLMSGGRLRLGVGAGYNRVEYHAMGVDFASRGRRLTEQISYLRRLWSEELISFEGEFDQIDRANIVPRPTRRIPIWCGGFAEAAFRRAVALADGFVF GYGLDQAATDGWIRLRELLAIAGRPLDGFGAQFVLHAPGQPYTDRQITDGLLRLRAAGATHASLFTMGRGLTGVDRHIDYIAEIMKKAEVALR&LINK_LOC=protein&PAGE_TYPE=BlastSearch', 'BLAST this protein','Franean1_4755')" />
   IDLLSDGRLRLGVGIGWNTVEYEALGRPMQSRGERLDQQIALLRALWTHRSVSFEHAGERIVAAGINPRPKRPIPIWIGGTSPAAYRRAGRTGDGWMPRL QPGPELTAARMIVTKAAEAAGRPPEAIGMHGRVRYTDGGAVGVAAAARRWREARATHLAVSTLGAGFTSLSEHLDALTAIARTGLLDENMEGPNR', 'http://blast.ncbi.nlm.nih.gov/Blast.cgi?PAGE=Proteins&PROGRAM=blastp&BLAST_PROGRAMS=blastp&QUERY=MEIGVVLPQGELHGGPRALRDYASAAQELGFRHLLAYDHVLGADPAGHPNWQGVYDADDPFHEPLVLFGYLAAVCDLDLVTSVLVLPQRQTALVAKQAAE IDLLSDGRLRLGVGIGWNTVEYEALGRPMQSRGERLDQQIALLRALWTHRSVSFEHAGERIVAAGINPRPKRPIPIWIGGTSPAAYRRAGRTGDGWMPRL QPGPELTAARMIVTKAAEAAGRPPEAIGMHGRVRYTDGGAVGVAAAARRWREARATHLAVSTLGAGFTSLSEHLDALTAIARTGLLDENMEGPNR&LINK_LOC=protein&PAGE_TYPE=BlastSearch', 'BLAST this protein','Sare_3016')" />
   EIDLLSDGRLRLGVGIGWNTVEYEALGRPMQSRGERLDQQITLLRSLWTHRSVSFEHSGERIVAAGINPRPKRPIPIWIGGASPAAYRRAGRTGDGWMPR LQPGPELTAARMIVTKAAEAAGRPPEAIGMHGRVRYLDGGAVGVAAAARRWREAHATHLAVSTLGAGFTSLSEHLEALTAIARTGLLDENMEGPNR', 'http://blast.ncbi.nlm.nih.gov/Blast.cgi?PAGE=Proteins&PROGRAM=blastp&BLAST_PROGRAMS=blastp&QUERY=MMEIGVVLPQGELHGGPRALREYASVAQELGFRHLLAYDHVLGADPAGHPNWQGVYDADDPFHEPLVLFGYLAAVCDLDLVTSVLVLPQRQTALVAKQAA EIDLLSDGRLRLGVGIGWNTVEYEALGRPMQSRGERLDQQITLLRSLWTHRSVSFEHSGERIVAAGINPRPKRPIPIWIGGASPAAYRRAGRTGDGWMPR LQPGPELTAARMIVTKAAEAAGRPPEAIGMHGRVRYLDGGAVGVAAAARRWREAHATHLAVSTLGAGFTSLSEHLEALTAIARTGLLDENMEGPNR&LINK_LOC=protein&PAGE_TYPE=BlastSearch', 'BLAST this protein','Strop_4150')" />

 
 
   RRLLELGVDRVLTDAAELVPLLDSLRPKVAV', 'http://blast.ncbi.nlm.nih.gov/Blast.cgi?PAGE=Proteins&PROGRAM=blastp&BLAST_PROGRAMS=blastp&QUERY=MILGVAESDAHAVANRLIAMHLSQAGFEVVNLGTCTPLADFAAALRRHPTAEAVLIGSLNGHAYADLRDLPALRAEGELRCPVVVGGNLAVGVDRSADAR RRLLELGVDRVLTDAAELVPLLDSLRPKVAV&LINK_LOC=protein&PAGE_TYPE=BlastSearch', 'BLAST this protein','AMED_4797')" />
   RRLLELGVDRVLTDAAELVPLLDSLRPKVAV', 'http://blast.ncbi.nlm.nih.gov/Blast.cgi?PAGE=Proteins&PROGRAM=blastp&BLAST_PROGRAMS=blastp&QUERY=MILGVAESDAHAVANRLIAMHLSQAGFEVVNLGTCTPLADFAAALRRHPTAEAVLIGSLNGHAYADLRDLPALRAEGELRCPVVVGGNLAVGVDRSADAR RRLLELGVDRVLTDAAELVPLLDSLRPKVAV&LINK_LOC=protein&PAGE_TYPE=BlastSearch', 'BLAST this protein','RAM_24420')" />
   GSNKDGDERLRLMRLGVDHILDDADQIPLLLDLLHAARATPLTGV', 'http://blast.ncbi.nlm.nih.gov/Blast.cgi?PAGE=Proteins&PROGRAM=blastp&BLAST_PROGRAMS=blastp&QUERY=MTTRSSHRSVIIGVARSDAHAVANHLIAMRLRAAGFHVINLGVCTPLEDFAEAAAANPDAEAVVVGSLNGHAYEDLSDLPVLRAAGRLLCPVIVGGNLSV GSNKDGDERLRLMRLGVDHILDDADQIPLLLDLLHAARATPLTGV&LINK_LOC=protein&PAGE_TYPE=BlastSearch', 'BLAST this protein','Sare_2951')" />

 
 
   SGTHGKRISLAHSRIMMHQPSAGIGGTAVDIAIQAESLERMKRQSQEILAAETGHPVEQIAEDSDRDRWFTADEARDYGIVDRVVSSFAEIAPHTTAPRI GL', 'http://blast.ncbi.nlm.nih.gov/Blast.cgi?PAGE=Proteins&PROGRAM=blastp&BLAST_PROGRAMS=blastp&QUERY=MTTAALPDLNYRDLLADRLFRQRTILLTGEVDDAMAERACSELVLLAAADPKRDIVLYINSPGGSVFAGLAIYDTMKLVPNDVVTVAMGFAASMGQVLLC SGTHGKRISLAHSRIMMHQPSAGIGGTAVDIAIQAESLERMKRQSQEILAAETGHPVEQIAEDSDRDRWFTADEARDYGIVDRVVSSFAEIAPHTTAPRI GL&LINK_LOC=protein&PAGE_TYPE=BlastSearch', 'BLAST this protein','ROP_12300')" />
   LCAGTAGKRYSLPNSHVLLHQGSAGFGGTAADVEIYAGHLERVSARMTELVARHTGQPVEKVEKDSLRDRWFDAAEALEYGFIDHIVERMDDVSPLAGTT TEGQ', 'http://blast.ncbi.nlm.nih.gov/Blast.cgi?PAGE=Proteins&PROGRAM=blastp&BLAST_PROGRAMS=blastp&QUERY=MPSATETPPIGTADDQLSARLLRQRIIVLGTEVDDQVANRLCAQLLLLSAENSRDDISLYINSPGGSVSAGMAIYDTMRLIPNDVRTVAMGLAASMGQVL LCAGTAGKRYSLPNSHVLLHQGSAGFGGTAADVEIYAGHLERVSARMTELVARHTGQPVEKVEKDSLRDRWFDAAEALEYGFIDHIVERMDDVSPLAGTT TEGQ&LINK_LOC=protein&PAGE_TYPE=BlastSearch', 'BLAST this protein','Snas_0218')" />

 
 
   LVLAQRPRRVLEIGCGTGLLAHRLHQHLRGYLGTDVAPAAVQRLEKADLPRTAFVQAAAHETATARVRAAMDDAFGQGVTPDCVLLNSVTQCFPGLGYLA EVLRQALAVVADGGTVIVGDIRHSDLLTAHFTWLEQSRDPDVGADDLRTRIRAATAADEELSFSPRAVAAVLTTQGRPVRVSLHARTMEQDTELTRYRYD IVLHVGAGSPEVPAPVRRIPWSGQVGADLATTLRAASADDPIVVSGIPNALLNDAPTAATPHALRHAVRELDAAVLLDPHDPRLLAVAAPATGGLLALED LVGSGPATGPEAHEPLAGFVRRRLPEVLRDHLRRQVPGTRSPRIVVADDSDGGETP', 'http://blast.ncbi.nlm.nih.gov/Blast.cgi?PAGE=Proteins&PROGRAM=blastp&BLAST_PROGRAMS=blastp&QUERY=MTVDSMVSIVSAHPWVADARATPGDVITVWPEPAATRAGPEPGPLLREHLDHWAEVYDWVYQEAVGRHSDDLDLSGWRASDTGEPLPIEHMREWLSCTVG LVLAQRPRRVLEIGCGTGLLAHRLHQHLRGYLGTDVAPAAVQRLEKADLPRTAFVQAAAHETATARVRAAMDDAFGQGVTPDCVLLNSVTQCFPGLGYLA EVLRQALAVVADGGTVIVGDIRHSDLLTAHFTWLEQSRDPDVGADDLRTRIRAATAADEELSFSPRAVAAVLTTQGRPVRVSLHARTMEQDTELTRYRYD IVLHVGAGSPEVPAPVRRIPWSGQVGADLATTLRAASADDPIVVSGIPNALLNDAPTAATPHALRHAVRELDAAVLLDPHDPRLLAVAAPATGGLLALED LVGSGPATGPEAHEPLAGFVRRRLPEVLRDHLRRQVPGTRSPRIVVADDSDGGETP&LINK_LOC=protein&PAGE_TYPE=BlastSearch', 'BLAST this protein','Sare_2077')" />
   RPRRVLEIGCGTGLLAHRLHPHLHGYVGTDVAQTAVQRLGDADLPRTAFVQAAAHETGTARVRAAMDGALGRAVAPDCVLLNSVTQCFPGLGYLAEVLRQ ALAVVADGGTVIVGDIRHSDLLTAHFTWLEQARDPGLGGTDLRNRVRAAIVADEELSFSPRAVAAVLAAGDRPVRVSLHARTMEQDTELTRYRYDLVLHV GAGSSKVSAPVRTIPWSEQLGAALAGTLRAASADEPIVVSGIPNALLNDVPTAVTPHALRHAVRELDAAVLLDPEDPRLLAVAAPAAGGLLTVEDLVGSG SQIGPEAHEPLAGFVRRRLPEVLRDHLRRQAPGTRPPRIVVADDSDDRGTR', 'http://blast.ncbi.nlm.nih.gov/Blast.cgi?PAGE=Proteins&PROGRAM=blastp&BLAST_PROGRAMS=blastp&QUERY=MVALVAAHPWVAEARLTPGGAITVRPEPAATLAGPEPGPLLREHLDHWSEVYDWVYQEAVGRHSDDLDLSGWRASDTGQPLPIEHMREWLACTVGLVLAQ RPRRVLEIGCGTGLLAHRLHPHLHGYVGTDVAQTAVQRLGDADLPRTAFVQAAAHETGTARVRAAMDGALGRAVAPDCVLLNSVTQCFPGLGYLAEVLRQ ALAVVADGGTVIVGDIRHSDLLTAHFTWLEQARDPGLGGTDLRNRVRAAIVADEELSFSPRAVAAVLAAGDRPVRVSLHARTMEQDTELTRYRYDLVLHV GAGSSKVSAPVRTIPWSEQLGAALAGTLRAASADEPIVVSGIPNALLNDVPTAVTPHALRHAVRELDAAVLLDPEDPRLLAVAAPAAGGLLTVEDLVGSG SQIGPEAHEPLAGFVRRRLPEVLRDHLRRQAPGTRPPRIVVADDSDDRGTR&LINK_LOC=protein&PAGE_TYPE=BlastSearch', 'BLAST this protein','Strop_2649')" />

 
 
   PVVLIGHSLGGIVSVRYVQRAVGPVDALVLSGPVIGGNPAITALLDLDPIPDVPLDPAALSRDPAVGAAYAADPLVYHGPFHRESLQTLKDVVATIAAGP GLGDLPTLWIHGELDPLAPLAETRAAFERIGGSNLRQKVYPGALHEIFNETNSDEVLDDVVAFVREAVPAR', 'http://blast.ncbi.nlm.nih.gov/Blast.cgi?PAGE=Proteins&PROGRAM=blastp&BLAST_PROGRAMS=blastp&QUERY=MTSPGTITTLAGSHGALALHRWSAQQPSFVALLAHGYGEHAGRYDHVARRLSDAGGAVYAPDHIGHGRSEGERAHVELLEDIVTDLGTVAKHATAEHPGL PVVLIGHSLGGIVSVRYVQRAVGPVDALVLSGPVIGGNPAITALLDLDPIPDVPLDPAALSRDPAVGAAYAADPLVYHGPFHRESLQTLKDVVATIAAGP GLGDLPTLWIHGELDPLAPLAETRAAFERIGGSNLRQKVYPGALHEIFNETNSDEVLDDVVAFVREAVPAR&LINK_LOC=protein&PAGE_TYPE=BlastSearch', 'BLAST this protein','FsymDg_2592')" />

 
 
   HPVFAGTTGQSSGRAGTGAVWEVGWPRNPLFTGRDAELAVLRAELVGSGAAAVLPVALHGLGGVGKTQMAAEYCYRFGREYDLVWWVAAEEPATTLAGLV WLAERIGVAVAGAAEESVRALVALLGSGTRFARWLLVLDNAGAPGDLFGLLRAASASGGHVLVTSRDHRWSGVAQSVRVDVLPSMDAVALLRARVPGIGD RDAARIAESLGNLPLAVEQAGAFLAETSMRPGEYAGLLSTELQQLMSRGAPEGVRPVAATWTVTLHQLEDPTAVMLARLWAHFGPEPIPLDLVRPQVAVL LPAPLDRASADRIGWAETVGRLLALALVRRTDDNDAVVMHRLAGAVLREDTPADLRPVLRTAARRLLAHGRPDAWDRPDAWPRFALLYAHASAVELVDDD DPDSRAMISRLTRYLLSRGDLPSSRALAERALSRNREILGEDHPDTLTSAANLASTLGELGQGDYAAARELAEDVLARRRQVLGEDHPDTLTAMADLAVT LRSLGDYAAARGLAQDVLARRREVLGEDHPDTLTAGAGLAATLRELRDYSAARALEENVLARRREVLGEDHPEVLTAAGNLAATLGELGDWSAARRLAEV VLARRREILGENHPDTLYAAGNLAVILWAMGGYAAARELEEGVLARRREILGENHPHTLRAAGNLAVTLWSTGHYAAARRLGEATLARSREVVGSGHPIT LVVAMHLATAVRSRTMKRTSAIVLGLYACACLFLQAVSINPAEKTAGLLVSILLVWPRGDHWGLLPALGRVFLGIPAFLLAERYLSSSFVSGLYLGFIFV WLTETFGIILGQLRSYAVYRQYPGSRTIGKSRVRWWK', 'http://blast.ncbi.nlm.nih.gov/Blast.cgi?PAGE=Proteins&PROGRAM=blastp&BLAST_PROGRAMS=blastp&QUERY=MGGMLRGGNGGVAVRADRAAGGGVKAGLTGEQIAALADVFTDPGSARQVVREAGIPAGDLPWTTASPRVFWTAVATLLADGIVAGGPERLLTLAREQYPA HPVFAGTTGQSSGRAGTGAVWEVGWPRNPLFTGRDAELAVLRAELVGSGAAAVLPVALHGLGGVGKTQMAAEYCYRFGREYDLVWWVAAEEPATTLAGLV WLAERIGVAVAGAAEESVRALVALLGSGTRFARWLLVLDNAGAPGDLFGLLRAASASGGHVLVTSRDHRWSGVAQSVRVDVLPSMDAVALLRARVPGIGD RDAARIAESLGNLPLAVEQAGAFLAETSMRPGEYAGLLSTELQQLMSRGAPEGVRPVAATWTVTLHQLEDPTAVMLARLWAHFGPEPIPLDLVRPQVAVL LPAPLDRASADRIGWAETVGRLLALALVRRTDDNDAVVMHRLAGAVLREDTPADLRPVLRTAARRLLAHGRPDAWDRPDAWPRFALLYAHASAVELVDDD DPDSRAMISRLTRYLLSRGDLPSSRALAERALSRNREILGEDHPDTLTSAANLASTLGELGQGDYAAARELAEDVLARRRQVLGEDHPDTLTAMADLAVT LRSLGDYAAARGLAQDVLARRREVLGEDHPDTLTAGAGLAATLRELRDYSAARALEENVLARRREVLGEDHPEVLTAAGNLAATLGELGDWSAARRLAEV VLARRREILGENHPDTLYAAGNLAVILWAMGGYAAARELEEGVLARRREILGENHPHTLRAAGNLAVTLWSTGHYAAARRLGEATLARSREVVGSGHPIT LVVAMHLATAVRSRTMKRTSAIVLGLYACACLFLQAVSINPAEKTAGLLVSILLVWPRGDHWGLLPALGRVFLGIPAFLLAERYLSSSFVSGLYLGFIFV WLTETFGIILGQLRSYAVYRQYPGSRTIGKSRVRWWK&LINK_LOC=protein&PAGE_TYPE=BlastSearch', 'BLAST this protein','FraEuI1c_3916')" />
   LHGMGGVGKTQLALEYAHRHTGDYTLIWWIDAEQTTLLAEKIATLARPLGLPTTTVPETASGVLAALARRPGWLVVFDNAEHPTALAPWLPTGPGHVLIT SRNPAWEHLAATVDVDLLPRPESVALLTHQLPGLDPAVAGALADELGDLPLALAQAGAYLARTRTPPRDYLAQFRARRAEYLATGDPPLYAGRLDTCWSL SLQRLAADAPAAVWLLQACALLAPDPIPLALFPTLPLRRRRWWHRRHPPAAGQDVREAVAAAADYSLLRHHDSDGTLTVHRLVQAVIAGQLTDAQRRTLT DTTTRLLTAATPTHQADDPRSWPAWTALGPHLLHAHSQLTGPDDPHHLRTTTDQFCYQLYARGDYTAANTLALRLHRDALHHHSPDHPTTLAAAHTLATT YNALGHHQAARQLAQDTLARQRRVLGDNHPRTLTSANNLAAQMYAMGEHQAARQLAQDTLARQRRVLGNDHPRTLTSANNLATYLSAMGEHQAARQLDED TLARRRRVLGDNHPNTLTSAGNLAGRLGVLGEHQAARQLAQDTLARQRRVLGDNHPNTQQTERLVSWIDEQASRG', 'http://blast.ncbi.nlm.nih.gov/Blast.cgi?PAGE=Proteins&PROGRAM=blastp&BLAST_PROGRAMS=blastp&QUERY=MVALVVTVVLVRRYDWEPAGTILAVITPALTIALLAYGTPLVFTRPPSPEPDPAGAGPPPRVDGPPVGALPARNRLFTGRAGQLERIRRQLQAGPVAVTA LHGMGGVGKTQLALEYAHRHTGDYTLIWWIDAEQTTLLAEKIATLARPLGLPTTTVPETASGVLAALARRPGWLVVFDNAEHPTALAPWLPTGPGHVLIT SRNPAWEHLAATVDVDLLPRPESVALLTHQLPGLDPAVAGALADELGDLPLALAQAGAYLARTRTPPRDYLAQFRARRAEYLATGDPPLYAGRLDTCWSL SLQRLAADAPAAVWLLQACALLAPDPIPLALFPTLPLRRRRWWHRRHPPAAGQDVREAVAAAADYSLLRHHDSDGTLTVHRLVQAVIAGQLTDAQRRTLT DTTTRLLTAATPTHQADDPRSWPAWTALGPHLLHAHSQLTGPDDPHHLRTTTDQFCYQLYARGDYTAANTLALRLHRDALHHHSPDHPTTLAAAHTLATT YNALGHHQAARQLAQDTLARQRRVLGDNHPRTLTSANNLAAQMYAMGEHQAARQLAQDTLARQRRVLGNDHPRTLTSANNLATYLSAMGEHQAARQLDED TLARRRRVLGDNHPNTLTSAGNLAGRLGVLGEHQAARQLAQDTLARQRRVLGDNHPNTQQTERLVSWIDEQASRG&LINK_LOC=protein&PAGE_TYPE=BlastSearch', 'BLAST this protein','Franean1_4762')" />
   LWAALARRLMAALRHHGGFRDLRLRFMDTHSDRPGEVVLRGPNRRPGPQPPRSLADPTGRRIVLVLTDGLAPAWRAGAVQKALAIWGRHQPVAVLVTLPQ RLWHRTGLDPRRVRLRASGPWAGSARMDWEFAEAPIAGPAAEAPGGRRPVPVPVLEVADEWIAPWARFVAGEGPRWTEVAALLVPPRRSEAPAPRPECTR PPAGAAERVARFRVWASPEAFSLATRLAAVPLDLPVMYAVQRRTPPRTGPVHLAEFFMSGLVEPVPESGGNSFLFGQGVREELLASSTRQATEMASRIAA EFLAPHSGAARELLIHLSGGDVPPEPEVTGDNLHFREIEHVVLEALSGAHLRRARRIQHVIRTTRASSDQMIDPTARGEINPAEHNEGPVTVNIPGTPET PVPSQQGGVGGDGALRGGANLTAVSQGATTPDSGASLERGSSGPSTRPAVWGNMPPRNLVFTGREALLERLERDLRDGPTAVLPHALHGMGGVGKSQLAL EYVYRHAARYDVVWWIPAERPTQIAQALVELAQRLHLPVTVEAITAVPAVLEALRTGNPYGNWLLVFDNAESPESVQEFFPSSPAGGPSGSILVTSRNPQ WNTLAHPLEVDVFKRSESIQLLQRRNPDLPDAEADQLAEVLGDLPLAVEQASAWRAETGMPAAEYLRLFEEKRAELMSVSPPTHYEQTVATAWNVSLDHL ASKNPAALQLLQICAYFASEPVARSLFSGAAVEPIAPDLDRALTDPLRLGRAIREINRYSLAKIDHRNNSIQMHRLVQAVLIARMTEEQRERMRRGAHML LAANAPRDPGDPEHWGRFADLYPHVVVSRAVDSDSRNVRQMVINLAQYLYFWGDHEAARDFGQHAYDIWREKYGEGDQHTLLLCRDLWYVLWRMGRYQEA SDLSERMLAQVRELGADAEEELLSTLGQVAADRRARGDFRGSLEISEEVYERAVRAYGDEDPLTLIHSHNMAVALRASGQFHRAMELDQETYRRTVLLYG EETYDSLLSEMGLALDRREAGEYALAARMFEELVDKFRRVFGEMNPNTLRTVGRLAVSRRKAGDHPGALELSRPVRKALTERYGERSPDSLISSLSLSVD LRQTGALDEAMQLGQSTRELYEEVFGEDHPDTAAADVDLAITLRLLNEVDTARAINESALRRARAALGDTHPYVLIASANLASDMFAQGDAAAAAELDRE NLEVIKATLGETHPTALVVMGNLACDLRALRQNDKAETLHATAVKELQAKLGESHPACADTAAWRRGNCDADPMPL', 'http://blast.ncbi.nlm.nih.gov/Blast.cgi?PAGE=Proteins&PROGRAM=blastp&BLAST_PROGRAMS=blastp&QUERY=MLLPTDDPAPARSARWSGTHAGRRWPSPRPGAADGNPTAEVASALRPLRQWTRSTRHQELDEEATAERLAEAPGLPPAMSPGGERRWDAVLVVDAGPQMT LWAALARRLMAALRHHGGFRDLRLRFMDTHSDRPGEVVLRGPNRRPGPQPPRSLADPTGRRIVLVLTDGLAPAWRAGAVQKALAIWGRHQPVAVLVTLPQ RLWHRTGLDPRRVRLRASGPWAGSARMDWEFAEAPIAGPAAEAPGGRRPVPVPVLEVADEWIAPWARFVAGEGPRWTEVAALLVPPRRSEAPAPRPECTR PPAGAAERVARFRVWASPEAFSLATRLAAVPLDLPVMYAVQRRTPPRTGPVHLAEFFMSGLVEPVPESGGNSFLFGQGVREELLASSTRQATEMASRIAA EFLAPHSGAARELLIHLSGGDVPPEPEVTGDNLHFREIEHVVLEALSGAHLRRARRIQHVIRTTRASSDQMIDPTARGEINPAEHNEGPVTVNIPGTPET PVPSQQGGVGGDGALRGGANLTAVSQGATTPDSGASLERGSSGPSTRPAVWGNMPPRNLVFTGREALLERLERDLRDGPTAVLPHALHGMGGVGKSQLAL EYVYRHAARYDVVWWIPAERPTQIAQALVELAQRLHLPVTVEAITAVPAVLEALRTGNPYGNWLLVFDNAESPESVQEFFPSSPAGGPSGSILVTSRNPQ WNTLAHPLEVDVFKRSESIQLLQRRNPDLPDAEADQLAEVLGDLPLAVEQASAWRAETGMPAAEYLRLFEEKRAELMSVSPPTHYEQTVATAWNVSLDHL ASKNPAALQLLQICAYFASEPVARSLFSGAAVEPIAPDLDRALTDPLRLGRAIREINRYSLAKIDHRNNSIQMHRLVQAVLIARMTEEQRERMRRGAHML LAANAPRDPGDPEHWGRFADLYPHVVVSRAVDSDSRNVRQMVINLAQYLYFWGDHEAARDFGQHAYDIWREKYGEGDQHTLLLCRDLWYVLWRMGRYQEA SDLSERMLAQVRELGADAEEELLSTLGQVAADRRARGDFRGSLEISEEVYERAVRAYGDEDPLTLIHSHNMAVALRASGQFHRAMELDQETYRRTVLLYG EETYDSLLSEMGLALDRREAGEYALAARMFEELVDKFRRVFGEMNPNTLRTVGRLAVSRRKAGDHPGALELSRPVRKALTERYGERSPDSLISSLSLSVD LRQTGALDEAMQLGQSTRELYEEVFGEDHPDTAAADVDLAITLRLLNEVDTARAINESALRRARAALGDTHPYVLIASANLASDMFAQGDAAAAAELDRE NLEVIKATLGETHPTALVVMGNLACDLRALRQNDKAETLHATAVKELQAKLGESHPACADTAAWRRGNCDADPMPL&LINK_LOC=protein&PAGE_TYPE=BlastSearch', 'BLAST this protein','SBI_00665')" />

 
 
   FRLALATSAKRAHAEHFLDLLGGSTVADDWVTSAEVERTKPAPDLIDAAMHAVEGRSAVFVGDSAWDCYAAGRLGIPTLGVRTGGWAAAELFDAGASAVY DSLPQLRSALDHTPLAAAD', 'http://blast.ncbi.nlm.nih.gov/Blast.cgi?PAGE=Proteins&PROGRAM=blastp&BLAST_PROGRAMS=blastp&QUERY=MPDTAVFDIDGTLVDSNYQHALAWFRAFRRFDLTMPVWRLHRAIGMGGDQFVQHVAGERTEREHGDDLRKAHGDEFDQLVDEVRPLEGARDLLEEIRRRG FRLALATSAKRAHAEHFLDLLGGSTVADDWVTSAEVERTKPAPDLIDAAMHAVEGRSAVFVGDSAWDCYAAGRLGIPTLGVRTGGWAAAELFDAGASAVY DSLPQLRSALDHTPLAAAD&LINK_LOC=protein&PAGE_TYPE=BlastSearch', 'BLAST this protein','SACE_4385')" />

 
 
   GNDNWIVPVLVIGTVVLVLSFALTLLQQLLLTRVRIVVSIRMSATFLSHLLRLPIRFFDARSPGGLVTRVQLNSQLAGLVSGQLATAAISALTMVIFAAV LIVVSTPLALAAIAMAALNAIALVAVSRARIAVNQNLQQTLIQLSGYTFLGIGMIDGIKATGAQDEYFARWAGIQARAVNAQQRLGVLTQGLLAVPVLLA SLNVVVILGLGGMLVITQRLSLSELIAFHVLAASFFAPIGQVVSVASQFQNASAWLMQIDDVLQQPTAVREDEPVKVATGKLTGKVELKDITFGYVTTDP PLVENLSLVLEPGARVALVGVSGSGKSTIAGLVAGIHEPWSGQILFDGRPRAEVPRAVMTASLGKVDQSIMLFSATVAENIALFSEGVAATDIATAANDA CIADDIEAKSGGFAHVLSEGGNNLSGGQRQRLEIARVLASSPTILILDEATSALDTITEAQIDSNLRRRGCTCLIVAHRLSTIRDCDQILVLENGKVIES GTHDELIRHRGRYLDLVSHD', 'http://blast.ncbi.nlm.nih.gov/Blast.cgi?PAGE=Proteins&PROGRAM=blastp&BLAST_PROGRAMS=blastp&QUERY=MMPPSGRHRSSLVGPRITTITSATEFDEAYSKIALTFEPGASFGVHERPRRDPEFLRLMRLLGLSSRGVILAVITGLLITVPTTAAAVLTAIFVEEVLRA GNDNWIVPVLVIGTVVLVLSFALTLLQQLLLTRVRIVVSIRMSATFLSHLLRLPIRFFDARSPGGLVTRVQLNSQLAGLVSGQLATAAISALTMVIFAAV LIVVSTPLALAAIAMAALNAIALVAVSRARIAVNQNLQQTLIQLSGYTFLGIGMIDGIKATGAQDEYFARWAGIQARAVNAQQRLGVLTQGLLAVPVLLA SLNVVVILGLGGMLVITQRLSLSELIAFHVLAASFFAPIGQVVSVASQFQNASAWLMQIDDVLQQPTAVREDEPVKVATGKLTGKVELKDITFGYVTTDP PLVENLSLVLEPGARVALVGVSGSGKSTIAGLVAGIHEPWSGQILFDGRPRAEVPRAVMTASLGKVDQSIMLFSATVAENIALFSEGVAATDIATAANDA CIADDIEAKSGGFAHVLSEGGNNLSGGQRQRLEIARVLASSPTILILDEATSALDTITEAQIDSNLRRRGCTCLIVAHRLSTIRDCDQILVLENGKVIES GTHDELIRHRGRYLDLVSHD&LINK_LOC=protein&PAGE_TYPE=BlastSearch', 'BLAST this protein','AS9A_4262')" />
   RIVDPSSGRRWCSKKEIEANFRSFCVTGARGNTKVSEGQVSDDPSWKSLAVLLAGKAFLLLPILLGVILVTGVTVAVPQITGWLVKQLGGMDQASIIAVI VSLAFGFILVHLLNTAINSVASTTISKRLTEIIYYTLLRAPLSYFNIRPRGELLYRVSLIKKLESFVSGVLPKLFVGSVAGIGCLIYICVLDFRSFFLLL AATLIYLGAFNFSQRQIRKLSDEQNSEDSLANSVLVDSISSIQEVKAGGHEEKIFESWAAHNAKVVSLERKKLFLRGSISSLVNAIQTFLPVVIFLTNLF SSFGPDALGQAVQLQLLATVFLAQVTMIVEMGAEIGETSSALRRVDDLVNYMDAPMFSPDASSQFQLPVRLESVSFRYGAFAKDSVSDISIEIGEQRRIA VVGKTGCGKSTLAKLIGGLLLPTSGSIEANGVRLRSVSQSSFYDCVAYVPQDSSLRNATLRDNLSWGNEHTDADLVRCLELAQFRLDPNLFPQGLQTMLI NGGQNISGGQRQRICIARALLKEPRLLILDEATSGLDQRTEASLYESLSRLGCAFVTITHRLETIRDFDEIVVLDQGIVVEKGSFESLLEGGGLFSKMYF AYQESTKDGL', 'http://blast.ncbi.nlm.nih.gov/Blast.cgi?PAGE=Proteins&PROGRAM=blastp&BLAST_PROGRAMS=blastp&QUERY=MVRVRPSCQVAQSDCGLACVHMLIQSFGISASLRKMRVTYAPGRDGLSLRALTQILSDFGLNSKVVKCSYSNLSRVPLPAIIAWEPAHYVVLEKRLGDSW RIVDPSSGRRWCSKKEIEANFRSFCVTGARGNTKVSEGQVSDDPSWKSLAVLLAGKAFLLLPILLGVILVTGVTVAVPQITGWLVKQLGGMDQASIIAVI VSLAFGFILVHLLNTAINSVASTTISKRLTEIIYYTLLRAPLSYFNIRPRGELLYRVSLIKKLESFVSGVLPKLFVGSVAGIGCLIYICVLDFRSFFLLL AATLIYLGAFNFSQRQIRKLSDEQNSEDSLANSVLVDSISSIQEVKAGGHEEKIFESWAAHNAKVVSLERKKLFLRGSISSLVNAIQTFLPVVIFLTNLF SSFGPDALGQAVQLQLLATVFLAQVTMIVEMGAEIGETSSALRRVDDLVNYMDAPMFSPDASSQFQLPVRLESVSFRYGAFAKDSVSDISIEIGEQRRIA VVGKTGCGKSTLAKLIGGLLLPTSGSIEANGVRLRSVSQSSFYDCVAYVPQDSSLRNATLRDNLSWGNEHTDADLVRCLELAQFRLDPNLFPQGLQTMLI NGGQNISGGQRQRICIARALLKEPRLLILDEATSGLDQRTEASLYESLSRLGCAFVTITHRLETIRDFDEIVVLDQGIVVEKGSFESLLEGGGLFSKMYF AYQESTKDGL&LINK_LOC=protein&PAGE_TYPE=BlastSearch', 'BLAST this protein','DIP_0754')" />
   QIMDPGQGLRTISLEEAESSFSGSVLVPTPRDDFEKKSRNVFKDWQFHTFFTKQMFGQYALFILLLAITYSVTFAVPMVIQHVVDAQLKGEENVVVGITA GVIAGIAGYYLVSIARAATLASIVSSIGYKLLGDLFARLLKLPLTYFALRSPGDILYRLASVNILRDFLSSNLTEFVINIGTMIVILIFIAQQSGVVLLI TCGVLSVLAVIWIVTARPTSQALDAEYSHASETQVIELDAINTVATMKMTGSATSTFGKWRETYLRSLASMRRRMVLQQGVLGSAAAVVQLGGPLILLLS SLPLVNNGTLTLGQAIATEGISALLFSSISSVMFGIMNIAAVDRAVARITDIQRYEPEQDEGTVTDVIDSDIELEDVGFTYPGGMAPVLSEVSLTVPEEQ DIAIVGSSGSGKSTLAMLLCSLYTPVSGAIRVGGIDIRDYSLDALRSHIGYVPQQLQTRTGSLYDNLTAGLDDGRSREEIEANIYAMGILDFVKDLPLGF DTIMANGGENFSGGQRQRIAIATALLRYPRILVLDEATSALDTATERQVTELIAQYQCTKVVVAHRLSTIRNAQQIVVMEHGCVVQVGSHAELISVEGRY RDLYYQEEKSSHKEYTSPIAA', 'http://blast.ncbi.nlm.nih.gov/Blast.cgi?PAGE=Proteins&PROGRAM=blastp&BLAST_PROGRAMS=blastp&QUERY=MAFPAVLQTAQTECGLGVSASILQHYGRYQTVSDLRLSMEPGREGLNLLQVSNLLTDEGMEVGAYRASSLEQLRDIGKPVILHWNNSHYVVLVRMRHDRV QIMDPGQGLRTISLEEAESSFSGSVLVPTPRDDFEKKSRNVFKDWQFHTFFTKQMFGQYALFILLLAITYSVTFAVPMVIQHVVDAQLKGEENVVVGITA GVIAGIAGYYLVSIARAATLASIVSSIGYKLLGDLFARLLKLPLTYFALRSPGDILYRLASVNILRDFLSSNLTEFVINIGTMIVILIFIAQQSGVVLLI TCGVLSVLAVIWIVTARPTSQALDAEYSHASETQVIELDAINTVATMKMTGSATSTFGKWRETYLRSLASMRRRMVLQQGVLGSAAAVVQLGGPLILLLS SLPLVNNGTLTLGQAIATEGISALLFSSISSVMFGIMNIAAVDRAVARITDIQRYEPEQDEGTVTDVIDSDIELEDVGFTYPGGMAPVLSEVSLTVPEEQ DIAIVGSSGSGKSTLAMLLCSLYTPVSGAIRVGGIDIRDYSLDALRSHIGYVPQQLQTRTGSLYDNLTAGLDDGRSREEIEANIYAMGILDFVKDLPLGF DTIMANGGENFSGGQRQRIAIATALLRYPRILVLDEATSALDTATERQVTELIAQYQCTKVVVAHRLSTIRNAQQIVVMEHGCVVQVGSHAELISVEGRY RDLYYQEEKSSHKEYTSPIAA&LINK_LOC=protein&PAGE_TYPE=BlastSearch', 'BLAST this protein','HMPREF0733_11066')" />
   HIMDPSTGMRVMDLQEAEESFSGSILVPSPRKDFKKKSRSVFQDWQLGTFFTKKMFGQYALFLLLLIITYSVTFAVPMVIQTVVDAQLRGARNTVVETTV GVVVGVIGYYLVSIARAATLASIVSSIGYKLLGNMFDRLLRLPLTYFALRSPGDILYRLSSVNTLRNFLSTSLTEFVINIGTMIVILIFIAQQSSQVLII TFVVLAVLMLIWFVTARPTSQALDAEYAHASEAQVVELDAINTIAAMKMTGSSTPTYNTWREAYMQSLASMRRRMVLQQGVLGSAASVVQLGGPLILLLA SIPLVNDGTLTLGQAIATEGISALLFSSVASTMFGIMSIIAVDRAVARIVDIQRYEPEKSTGSVTDIADGDLDLENVNFTYPGGNKPVLTDISLRVPEGH DVALVGSSGSGKSTLAKLLCSLYSPVSGTIRVGGIDVKDYSLDELRSHIGYVPQQLQTRSGSLYDNLTAGLDDGRSREEIEESILAMGILDFVKDLPMGF DTIMASGGENFSGGQRQRIAIVTALLRYPRILVLDEATSALDTATERQVTELIAQYQCTKVVVAHRLSTVRNAENIVVMEDGHIIQSGRHDDLMMVEGRY RELYSQEEQSSQAGYIWAQWPPTKVSRHGFGLGMFTS', 'http://blast.ncbi.nlm.nih.gov/Blast.cgi?PAGE=Proteins&PROGRAM=blastp&BLAST_PROGRAMS=blastp&QUERY=MGFPAVLQAAKTECGLGVGASVLQHYGRYQTISDLRLIAEPGREGLSMRQVRDLLVEEGMEVKAFRAPSLEHLRDIGKPVILHWNNSHYVVLVRIRKDRV HIMDPSTGMRVMDLQEAEESFSGSILVPSPRKDFKKKSRSVFQDWQLGTFFTKKMFGQYALFLLLLIITYSVTFAVPMVIQTVVDAQLRGARNTVVETTV GVVVGVIGYYLVSIARAATLASIVSSIGYKLLGNMFDRLLRLPLTYFALRSPGDILYRLSSVNTLRNFLSTSLTEFVINIGTMIVILIFIAQQSSQVLII TFVVLAVLMLIWFVTARPTSQALDAEYAHASEAQVVELDAINTIAAMKMTGSSTPTYNTWREAYMQSLASMRRRMVLQQGVLGSAASVVQLGGPLILLLA SIPLVNDGTLTLGQAIATEGISALLFSSVASTMFGIMSIIAVDRAVARIVDIQRYEPEKSTGSVTDIADGDLDLENVNFTYPGGNKPVLTDISLRVPEGH DVALVGSSGSGKSTLAKLLCSLYSPVSGTIRVGGIDVKDYSLDELRSHIGYVPQQLQTRSGSLYDNLTAGLDDGRSREEIEESILAMGILDFVKDLPMGF DTIMASGGENFSGGQRQRIAIVTALLRYPRILVLDEATSALDTATERQVTELIAQYQCTKVVVAHRLSTVRNAENIVVMEDGHIIQSGRHDDLMMVEGRY RELYSQEEQSSQAGYIWAQWPPTKVSRHGFGLGMFTS&LINK_LOC=protein&PAGE_TYPE=BlastSearch', 'BLAST this protein','HMPREF0733_11070')" />
   TILDPAVGRRKLSATDFSSGFSGILIVGSPPFIREKAKAEPSPWLSILPVARANRGRIAMLMLLAMATVVTTLASPLAVSGAVSSLMENQSTPSMMALVL FLGVMATLLLINVINVLIAVSAAVAIGRDLAAKVFSSLLDLPFKYFALRNRGEILYRIHATNQIESFLTDEVARSVGSALTVTAAAIGLLWLSPPIGCLA ILVFSGMWLLLVVARRKTTQWADAEMHFESLANAVQVDSITAVSLIKTGGLKESTFHEWINPFDNGVRWRKKREFLEGLVQTAATFVQTLSPLVFTVAAL SGTIGQSMNLGGALGLQMLSGVFFSQLTVISQLAARWGTAISSARRIDDILSHSKDKIFYGNKPIELSGRVKLQNVDFAYTNLSPLAITGINFEIKPGEH VAFVGPSGSGKSSLAKLIVGLYSPTHGQILFNGMPLRYHEEASFRAQVAYVEQDSQLLNATIWDNIALGRSNVSFNDVRDAAMKASINDDIEGMPMGYDT FLTNGGDNISGGQRQRIALARALFGDPKVLVLDEATSGLDRASEQRVLKSLEGMCQTRITIAHRLDTIRSADRIFVMDKGKIIASGDHNMLLRECELYAE LYKEQGY', 'http://blast.ncbi.nlm.nih.gov/Blast.cgi?PAGE=Proteins&PROGRAM=blastp&BLAST_PROGRAMS=blastp&QUERY=MVRRIKPLHQVTQTECGLVACVMLMKALGSPITLREAREKYEVGRDGLTIRQIRDLLTNQGLAPRVLKVPDSAIFDIPTPAIAYWKKSHFVMVESVGSSF TILDPAVGRRKLSATDFSSGFSGILIVGSPPFIREKAKAEPSPWLSILPVARANRGRIAMLMLLAMATVVTTLASPLAVSGAVSSLMENQSTPSMMALVL FLGVMATLLLINVINVLIAVSAAVAIGRDLAAKVFSSLLDLPFKYFALRNRGEILYRIHATNQIESFLTDEVARSVGSALTVTAAAIGLLWLSPPIGCLA ILVFSGMWLLLVVARRKTTQWADAEMHFESLANAVQVDSITAVSLIKTGGLKESTFHEWINPFDNGVRWRKKREFLEGLVQTAATFVQTLSPLVFTVAAL SGTIGQSMNLGGALGLQMLSGVFFSQLTVISQLAARWGTAISSARRIDDILSHSKDKIFYGNKPIELSGRVKLQNVDFAYTNLSPLAITGINFEIKPGEH VAFVGPSGSGKSSLAKLIVGLYSPTHGQILFNGMPLRYHEEASFRAQVAYVEQDSQLLNATIWDNIALGRSNVSFNDVRDAAMKASINDDIEGMPMGYDT FLTNGGDNISGGQRQRIALARALFGDPKVLVLDEATSGLDRASEQRVLKSLEGMCQTRITIAHRLDTIRSADRIFVMDKGKIIASGDHNMLLRECELYAE LYKEQGY&LINK_LOC=protein&PAGE_TYPE=BlastSearch', 'BLAST this protein','HMPREF0733_11079')" />
   ASRVPVPAVVFLDGRHYAVLEGVRRGRAWINDPAVGRLPLTPAEFATRANGPVLVATPTPEFEPGGERWPFARAVAQRIRPYAPMILAGALLGGVAAVPT LLASLTMRAVLNRVLILGDLAWRPALLAILVGGALLAALAGWAQQRIFATALAAMATRFSSRYLTALLRLPGEFFHRRHLSGLVNRTQMNDGLAIALSER VVLPAAAAVTVAGYGLFIGYLAPRLLVVMSLAMAAQIVLLNVVRRRAGPHQQRLLFEQLRRDSTAHSGLKMIESVKADGAERWLFASWARSAGRTLDAAN ALASATLGVMALSAAVGPAALAGLALVGAHDVAAGRIDLGTLLTAQLVGGAMLAPLGLLVGLGSEIQVTRVHVSVIDDALAATPHPARATVLAPDAPPEP AQPARLSGRIEFDRVSFGYDRHRPPLVRDISFTVEPGQWVALVGVSGSGKSTLARLAVGAAEPWSGAVLLDGRPRETYSRRTLAASIGYVEQQLRLFEGN LRDNLTLWDPTLPEERLRAALVDARIDRLVEQRGGLDHGAVNEHARNLSGGEQQRLELARALAADPPLLVLDEATSALDATTEFAVLEALRRRGVTCMFI AHRISTVRDADLILVLDRGEIVQRGTHAELIDTDGVYRRLATDGRSA', 'http://blast.ncbi.nlm.nih.gov/Blast.cgi?PAGE=Proteins&PROGRAM=blastp&BLAST_PROGRAMS=blastp&QUERY=MVVLADSQATTPPAEEPQPQPRRQRAVRTVETPQMEDADCGAACLSIILTHHGRRVPLAELRDRCGVGRDGMTASGFARAAAGYGLKVTGRWIETSDLDM ASRVPVPAVVFLDGRHYAVLEGVRRGRAWINDPAVGRLPLTPAEFATRANGPVLVATPTPEFEPGGERWPFARAVAQRIRPYAPMILAGALLGGVAAVPT LLASLTMRAVLNRVLILGDLAWRPALLAILVGGALLAALAGWAQQRIFATALAAMATRFSSRYLTALLRLPGEFFHRRHLSGLVNRTQMNDGLAIALSER VVLPAAAAVTVAGYGLFIGYLAPRLLVVMSLAMAAQIVLLNVVRRRAGPHQQRLLFEQLRRDSTAHSGLKMIESVKADGAERWLFASWARSAGRTLDAAN ALASATLGVMALSAAVGPAALAGLALVGAHDVAAGRIDLGTLLTAQLVGGAMLAPLGLLVGLGSEIQVTRVHVSVIDDALAATPHPARATVLAPDAPPEP AQPARLSGRIEFDRVSFGYDRHRPPLVRDISFTVEPGQWVALVGVSGSGKSTLARLAVGAAEPWSGAVLLDGRPRETYSRRTLAASIGYVEQQLRLFEGN LRDNLTLWDPTLPEERLRAALVDARIDRLVEQRGGLDHGAVNEHARNLSGGEQQRLELARALAADPPLLVLDEATSALDATTEFAVLEALRRRGVTCMFI AHRISTVRDADLILVLDRGEIVQRGTHAELIDTDGVYRRLATDGRSA&LINK_LOC=protein&PAGE_TYPE=BlastSearch', 'BLAST this protein','VAB18032_20065')" />

 
 
   LALRVNAPQPAGTPWDILLVSAGSGVLSRAVALRPATSWNAQTLTTLMPLRYQDANWWLRARTAGDIGGARLALDDVRRRLERGGIEVSLDQACGRGDFT PLARVSLTAVIDDDVSFDPVVNTAPGVSLHPRWLADLRARAYRHSRDGRDADQ', 'http://blast.ncbi.nlm.nih.gov/Blast.cgi?PAGE=Proteins&PROGRAM=blastp&BLAST_PROGRAMS=blastp&QUERY=MRQRKFRACGRPKVVRSGGFPSTQNGNPRPVNVFELATAPFGWGSAIRGKRFFHPDGVLAGGVVERVAPAGRGLPIPPSRIVARLSKATGTPGALPDFIG LALRVNAPQPAGTPWDILLVSAGSGVLSRAVALRPATSWNAQTLTTLMPLRYQDANWWLRARTAGDIGGARLALDDVRRRLERGGIEVSLDQACGRGDFT PLARVSLTAVIDDDVSFDPVVNTAPGVSLHPRWLADLRARAYRHSRDGRDADQ&LINK_LOC=protein&PAGE_TYPE=BlastSearch', 'BLAST this protein','MAP_3066c')" />
   VALRPATSWNAQTLTTLMPLRYQDANWWLRARTASDIGGAGLALDDVRRRLERGGIEVSLDQACGRGDFTPLARVSLTAVIDDDVSFDPVVNTAPGVSLH PRWLADLRARAYRHSRDGRDADQ', 'http://blast.ncbi.nlm.nih.gov/Blast.cgi?PAGE=Proteins&PROGRAM=blastp&BLAST_PROGRAMS=blastp&QUERY=MNVFELATAPFGWGSAIRGKRFFHPDGVLAGGVAERVAPAGRGLPIPPSRIVARLSKATGTPGALPDFIGLALRVSAPQPAGTPWDILLVSAGSGVLSRA VALRPATSWNAQTLTTLMPLRYQDANWWLRARTASDIGGAGLALDDVRRRLERGGIEVSLDQACGRGDFTPLARVSLTAVIDDDVSFDPVVNTAPGVSLH PRWLADLRARAYRHSRDGRDADQ&LINK_LOC=protein&PAGE_TYPE=BlastSearch', 'BLAST this protein','MAV_3881')" />

 
 
   EEVTTRTSMLANLMAQSTGDELGCRIIVNGEVRDEQVQSREQGSVACKVKSA', 'http://blast.ncbi.nlm.nih.gov/Blast.cgi?PAGE=Proteins&PROGRAM=blastp&BLAST_PROGRAMS=blastp&QUERY=MAFPRGLGAFGRKVIRHGWLVVVIAVVASLASVSVMKVRALSAPSGPVVSPKGAPKLPSFAPKHVVYMVFGESAGTGTLTYLDVDSRPHRVDFTTLPWTH EEVTTRTSMLANLMAQSTGDELGCRIIVNGEVRDEQVQSREQGSVACKVKSA&LINK_LOC=protein&PAGE_TYPE=BlastSearch', 'BLAST this protein','MAB_2036')" />

 
 
   VSDAAKASRLSSKPYGTFAPKGDAPKSKSDKDSKVTITITDQSGAVPRDCTVDYVNVADGTSDEIELGTNCKGSVSLPPGQYDFLSWAGGESGDVQGVTT AKVKNSPLDVAIDGTKSKEVTYKVDQAAQLGEQSVKVSMMPEKGDGLIVGVDAPAGRKQFVIPTGKSDHTIGIQATPHLVGTDNANPYTYDLDFYQTDGI PAKPVFTARDGDLAKVTRTFDGLATTEPTNGCNWSYRKGASHFSYCQPREQKWQTQRTEYLTPGKDITWHADTVIGDYASEKYVQASSTSTFKAGPSERV TGQAPLSFNVGGSGTQPLVVRDGDKLYANMPFLDNADSKEYIVDYNSIKGTAVLKRDGKEVARKDLKVSGFELTLPKKDSGRYTLSVKSTHSDSYTPLAT KSAVKWEFKSKPTTKPVPLPVSAVAFDAEGVSNGYADVSKPQKFTLDYQAQQGAKDTKLKKLVFEVSYDDGKTWTKVDTKVDGDSATGELKHPADATFVS VRATATDDAGNTVTHSTVHTHGLK', 'http://blast.ncbi.nlm.nih.gov/Blast.cgi?PAGE=Proteins&PROGRAM=blastp&BLAST_PROGRAMS=blastp&QUERY=MLAAGGAFAFAETDNAAEASPSPPDSRVVTLPTGDIVSVSPDGGMVWRPAKDREDIGLINPPAHDGSGDVVAIPTDRVDDIKNGKEDPRRYNVSELLRSG VSDAAKASRLSSKPYGTFAPKGDAPKSKSDKDSKVTITITDQSGAVPRDCTVDYVNVADGTSDEIELGTNCKGSVSLPPGQYDFLSWAGGESGDVQGVTT AKVKNSPLDVAIDGTKSKEVTYKVDQAAQLGEQSVKVSMMPEKGDGLIVGVDAPAGRKQFVIPTGKSDHTIGIQATPHLVGTDNANPYTYDLDFYQTDGI PAKPVFTARDGDLAKVTRTFDGLATTEPTNGCNWSYRKGASHFSYCQPREQKWQTQRTEYLTPGKDITWHADTVIGDYASEKYVQASSTSTFKAGPSERV TGQAPLSFNVGGSGTQPLVVRDGDKLYANMPFLDNADSKEYIVDYNSIKGTAVLKRDGKEVARKDLKVSGFELTLPKKDSGRYTLSVKSTHSDSYTPLAT KSAVKWEFKSKPTTKPVPLPVSAVAFDAEGVSNGYADVSKPQKFTLDYQAQQGAKDTKLKKLVFEVSYDDGKTWTKVDTKVDGDSATGELKHPADATFVS VRATATDDAGNTVTHSTVHTHGLK&LINK_LOC=protein&PAGE_TYPE=BlastSearch', 'BLAST this protein','Snas_3481')" />

 
 
   VEDCLYLNVTTPRRGGRNLPVMVWLHGGSFTTGAGAIYDARALAARGDVVVVTPNYRLGPFGFLAMPSLTAESPGSQSGNYGIQDQQAALRWVQRNAAAF GGDPRNVTIFGGSAGGASVCVNLTSPTAAGLFHRAIAQSFSCASELASKQQAEAAGVRLATGFGCPDPATAAACMRTKPVRELTLSWPGGFPVVGGPELP LHPPEALRRDRFSHVPLIMGNTRDEMRLYVGLEFEARGNPVTPQLFEQRVRETFGGAADRVLARYPLSAYPTPAIALSTVVTDAGNTLATCDHLAGYRLA SARPRPVPVYAYQFADRTAPVPVEIPGLDEGAMHATELPYLFTGVFGEPLTGPQRDLSNRMIDYWTAFARTGDPNRAGLPAWPEHRPDAGANVLTLDLAT SGGTRLTDVARASNCAFWNSIGFGDGPREPR', 'http://blast.ncbi.nlm.nih.gov/Blast.cgi?PAGE=Proteins&PROGRAM=blastp&BLAST_PROGRAMS=blastp&QUERY=MWRRRLLVLLGVAGLLSGAVPAIADPVGRPDAVRTAEGPVRGVVERDARVFRGIPFAAPPVGELRWRPPQPVRPWREPLDATEPGAACAQPSDFGLPESF VEDCLYLNVTTPRRGGRNLPVMVWLHGGSFTTGAGAIYDARALAARGDVVVVTPNYRLGPFGFLAMPSLTAESPGSQSGNYGIQDQQAALRWVQRNAAAF GGDPRNVTIFGGSAGGASVCVNLTSPTAAGLFHRAIAQSFSCASELASKQQAEAAGVRLATGFGCPDPATAAACMRTKPVRELTLSWPGGFPVVGGPELP LHPPEALRRDRFSHVPLIMGNTRDEMRLYVGLEFEARGNPVTPQLFEQRVRETFGGAADRVLARYPLSAYPTPAIALSTVVTDAGNTLATCDHLAGYRLA SARPRPVPVYAYQFADRTAPVPVEIPGLDEGAMHATELPYLFTGVFGEPLTGPQRDLSNRMIDYWTAFARTGDPNRAGLPAWPEHRPDAGANVLTLDLAT SGGTRLTDVARASNCAFWNSIGFGDGPREPR&LINK_LOC=protein&PAGE_TYPE=BlastSearch', 'BLAST this protein','Amir_4606')" />

 
 
   RTQDIQVLGRSQNGTAFTSLRARAGYAFSPSGNQNTVTIPVDGRVADLQLKFFANTGAPGGQIAEVQVLGTAAPNPDLTVSALSWSPTSPSETDEITVRG TVRNAGSAASPATTVNVSLGGVVVGSAPVGPLVAGASAPVSVGVGKRPQGSYTVSALVDPTDTVVESDNTNNSRTTASPLVVGQSPGPDLEVRSISSSPA NPAVGAAVTFTVAVHNRGTSAVSAGTVTRLTVGSTTLNGTTPAIAAGATANVAVSGSWTAASGGATLTATADATNQVAETNETNNTFARSIVVGRGAAVP YTELEAENAAYRGTLLKSDAERTFGHTNFATESSGRESVRLNATGEYVEFTSTAPSNSVVVRNSIPDAPGGGGREATISLYADGQFVRKLDLSSKHSWLY GNTDSPEGLTNTPGGDARRLFDESHALLTRTYPAGTKFRLQRDASDNASFYIIDLIDLEQVAAPSSQPSGCVSITTYGAVANDGIDDTAAIQRAVTANQT GQIDCVWIPAGQWRQEQKILTDDPLDRGQWNQVGIRDVTIRGAGMWHSQLYTLTPPHEAGGINHPHEGNFGFDIDENTQISDIAIFGSGTIRGGDGNHEG GVALNGRFGKDTKISNVWIEHANVGVWAGRDFDNIQELWNPGDGVEFTGMRIRNTYADGINFANGTRNSTVYNSSFRNTGDDALAVWSSKYVKNQSVDIG HDNSFRNNTIQLPWRANGIAIYGGYGNKIENNLISDTMNYPAIMLATDHDPLPFSGQTLIANNGLYRTGGAFWNEDQEFGAITLFPQNLPIPGVTIRDTD IIDSTYDGIQFKTGGGVMQDVKIENVRIDKSNNGSGILAMGGARGNATLTNVTITNSRDGHVLIEPGSQFTVAGTPNGARAKR', 'http://blast.ncbi.nlm.nih.gov/Blast.cgi?PAGE=Proteins&PROGRAM=blastp&BLAST_PROGRAMS=blastp&QUERY=MRWKRRTGQSITGLLIAGLVSVGLLPVAASAAEATDLARGKPVAASGSHGGYPAANANDGKVDSYWESNGLPADLTVKLGADADLESVVIKLNPDPIWGG RTQDIQVLGRSQNGTAFTSLRARAGYAFSPSGNQNTVTIPVDGRVADLQLKFFANTGAPGGQIAEVQVLGTAAPNPDLTVSALSWSPTSPSETDEITVRG TVRNAGSAASPATTVNVSLGGVVVGSAPVGPLVAGASAPVSVGVGKRPQGSYTVSALVDPTDTVVESDNTNNSRTTASPLVVGQSPGPDLEVRSISSSPA NPAVGAAVTFTVAVHNRGTSAVSAGTVTRLTVGSTTLNGTTPAIAAGATANVAVSGSWTAASGGATLTATADATNQVAETNETNNTFARSIVVGRGAAVP YTELEAENAAYRGTLLKSDAERTFGHTNFATESSGRESVRLNATGEYVEFTSTAPSNSVVVRNSIPDAPGGGGREATISLYADGQFVRKLDLSSKHSWLY GNTDSPEGLTNTPGGDARRLFDESHALLTRTYPAGTKFRLQRDASDNASFYIIDLIDLEQVAAPSSQPSGCVSITTYGAVANDGIDDTAAIQRAVTANQT GQIDCVWIPAGQWRQEQKILTDDPLDRGQWNQVGIRDVTIRGAGMWHSQLYTLTPPHEAGGINHPHEGNFGFDIDENTQISDIAIFGSGTIRGGDGNHEG GVALNGRFGKDTKISNVWIEHANVGVWAGRDFDNIQELWNPGDGVEFTGMRIRNTYADGINFANGTRNSTVYNSSFRNTGDDALAVWSSKYVKNQSVDIG HDNSFRNNTIQLPWRANGIAIYGGYGNKIENNLISDTMNYPAIMLATDHDPLPFSGQTLIANNGLYRTGGAFWNEDQEFGAITLFPQNLPIPGVTIRDTD IIDSTYDGIQFKTGGGVMQDVKIENVRIDKSNNGSGILAMGGARGNATLTNVTITNSRDGHVLIEPGSQFTVAGTPNGARAKR&LINK_LOC=protein&PAGE_TYPE=BlastSearch', 'BLAST this protein','SGR_474')" />
   RTQTLSLQGSADGTSFATLKNSATYTFAPGAANEVTIAFPATLTRFVRVNITANTGWPAAQLSELEVRGAGESSADLAAGKTLTASSSNGSQTPANANDG NRGSYWASRDGQFPQWIQADLGALLGVDRVVLRLPDGWAARSQTLKLQGSANGTDFTDLTASKAYRFDAAGGQSATIAFDATTTRYVRVLVTANTGAGAA QLSEFEVYGPATGDTRAPTAPTNLAFTEPASGQIRLTWKASTDDKGVTGYDVYANNTLLTSVAGDVTTYTDTRPAGQDVSYFVRAKDAAGNVSANSNTVT RRGETGDTQAPTAPSALAYTEPSSGSIKLTWGASTDDKGVTGYDVYANNVLRGSVAGNVLTYTDTQPASTTVSYVVRAKDAAGNVSGDSNTVTRNGQSGS ASNLAVNKPITASSVVHTFVAANANDNSTSTYWEGAGGSYPNTLTVKLGSNADTENVVIKLNPDSSWGPRTQRIEVLGREQSSSSLNTLVAAKDYAFGPA SGNTVTIPVSARVADVQLKFTANSGSGAGQVAEFQVLGAPAANPDLQVTGITAAPAAPVETDEVTLTATVRNAGALAAPASKVELRLGGTKVATASVGPL AAGASTQVSASIGARNAGSYVLSAVADPAGEVIEQNETNNSYTSASPLVVKPVSSSDLVASAVTTSPSAASAGETVTFSVALKNQGTVASAAGAHGITLT LVDSKGATVKTLTGNHTGAIAAGATTAPVTLGTWTAANGSYTVRTVIAADANELPVKRENNTSTQALFVGRGANMPYDMYEAEDGTAGGAAKTVGPNRTI GDVAGEASGRKAVTLDRTGDYVEFTTRAATNTLVTRFSIPDAPGGGGIDSTLNVYVDGVFLKAIDLTSKYAWLYGNEAAPGNSPGSGAPRHIYDEANTML GRTVPAGSKIRLQKDSANTTTYAIDFINLEQVAPVANPNPATYTVPAGFTHQDVQNALDKVRMDTTGTLVGVYLPAGQYTTASKFQVYGKAVKVVGAGPW YTRFNAPSTQDNTDVGFRAEASAKGSSFANFAYFGNYTSRIDGPGKVFDFSNVSDIVIDNIWNEHMVCLYWGANTDSVTIKNSRIRNMFADGVNMTNGST DNLVTNNEARATGDDSFALFSAIDAGGADMKNNVYENLTSILTWRAAGVAVYGGYDNTFRNIHIADTLVYSGITVSSLDFGYPMNGFGTGPTRIENVSIV RAGGHFWGSQTFPGIWLFSASKVFQGIRINNVDIVDPTYSGIMFQTNYVGGQPQFPIKDTVLTDVSISGARKSGDAFDAKSGFGLWANEMPEAGQGPAVG EVTFNGLKLSDNAQDIRNTTSTFKIINNP', 'http://blast.ncbi.nlm.nih.gov/Blast.cgi?PAGE=Proteins&PROGRAM=blastp&BLAST_PROGRAMS=blastp&QUERY=MRAQRWRWRAISALVTTSLLMIGVPSLAAAAADGPNLAAGRAAAASSAHAEYGASNITDGNRSTYWESAGGGLPQWVQADLGSGARVDEVKLTLPAGWET RTQTLSLQGSADGTSFATLKNSATYTFAPGAANEVTIAFPATLTRFVRVNITANTGWPAAQLSELEVRGAGESSADLAAGKTLTASSSNGSQTPANANDG NRGSYWASRDGQFPQWIQADLGALLGVDRVVLRLPDGWAARSQTLKLQGSANGTDFTDLTASKAYRFDAAGGQSATIAFDATTTRYVRVLVTANTGAGAA QLSEFEVYGPATGDTRAPTAPTNLAFTEPASGQIRLTWKASTDDKGVTGYDVYANNTLLTSVAGDVTTYTDTRPAGQDVSYFVRAKDAAGNVSANSNTVT RRGETGDTQAPTAPSALAYTEPSSGSIKLTWGASTDDKGVTGYDVYANNVLRGSVAGNVLTYTDTQPASTTVSYVVRAKDAAGNVSGDSNTVTRNGQSGS ASNLAVNKPITASSVVHTFVAANANDNSTSTYWEGAGGSYPNTLTVKLGSNADTENVVIKLNPDSSWGPRTQRIEVLGREQSSSSLNTLVAAKDYAFGPA SGNTVTIPVSARVADVQLKFTANSGSGAGQVAEFQVLGAPAANPDLQVTGITAAPAAPVETDEVTLTATVRNAGALAAPASKVELRLGGTKVATASVGPL AAGASTQVSASIGARNAGSYVLSAVADPAGEVIEQNETNNSYTSASPLVVKPVSSSDLVASAVTTSPSAASAGETVTFSVALKNQGTVASAAGAHGITLT LVDSKGATVKTLTGNHTGAIAAGATTAPVTLGTWTAANGSYTVRTVIAADANELPVKRENNTSTQALFVGRGANMPYDMYEAEDGTAGGAAKTVGPNRTI GDVAGEASGRKAVTLDRTGDYVEFTTRAATNTLVTRFSIPDAPGGGGIDSTLNVYVDGVFLKAIDLTSKYAWLYGNEAAPGNSPGSGAPRHIYDEANTML GRTVPAGSKIRLQKDSANTTTYAIDFINLEQVAPVANPNPATYTVPAGFTHQDVQNALDKVRMDTTGTLVGVYLPAGQYTTASKFQVYGKAVKVVGAGPW YTRFNAPSTQDNTDVGFRAEASAKGSSFANFAYFGNYTSRIDGPGKVFDFSNVSDIVIDNIWNEHMVCLYWGANTDSVTIKNSRIRNMFADGVNMTNGST DNLVTNNEARATGDDSFALFSAIDAGGADMKNNVYENLTSILTWRAAGVAVYGGYDNTFRNIHIADTLVYSGITVSSLDFGYPMNGFGTGPTRIENVSIV RAGGHFWGSQTFPGIWLFSASKVFQGIRINNVDIVDPTYSGIMFQTNYVGGQPQFPIKDTVLTDVSISGARKSGDAFDAKSGFGLWANEMPEAGQGPAVG EVTFNGLKLSDNAQDIRNTTSTFKIINNP&LINK_LOC=protein&PAGE_TYPE=BlastSearch', 'BLAST this protein','SGR_6622')" />

 
 
   DEIVAFEIPGDVAVLLHRGTWHAGPFFDGPRMAFFNLELTDTNAADHDTSQLDVRLGVECWFDRG', 'http://blast.ncbi.nlm.nih.gov/Blast.cgi?PAGE=Proteins&PROGRAM=blastp&BLAST_PROGRAMS=blastp&QUERY=MALTRHAVATVPITENAFAPYGQVLAAQPDSTRTTAAEAALDLSRGTPRFYVMELTHGRTTFTRITRHRQVTQVLAAVGGGAWWMAVARGEGPAEVAPAL DEIVAFEIPGDVAVLLHRGTWHAGPFFDGPRMAFFNLELTDTNAADHDTSQLDVRLGVECWFDRG&LINK_LOC=protein&PAGE_TYPE=BlastSearch', 'BLAST this protein','FraEuI1c_4382')" />

 
 
   
   
   
   
   

 
 
   TPGQICSAIGALDEARRGWPSQVTEDTAEGVGALDHEEVRERMSGNLCRCGAYGGIVEAVLEAAGAEGGAR', 'http://blast.ncbi.nlm.nih.gov/Blast.cgi?PAGE=Proteins&PROGRAM=blastp&BLAST_PROGRAMS=blastp&QUERY=MRINGAAHALTVDNRTTLLDVLRERLGLTGTKKGCDHGQCGACTVLMDGERVNSCLMFAVAAEGREIVSIEGVADLTDGAELHPVQRAFLNHDGLQCGYC TPGQICSAIGALDEARRGWPSQVTEDTAEGVGALDHEEVRERMSGNLCRCGAYGGIVEAVLEAAGAEGGAR&LINK_LOC=protein&PAGE_TYPE=BlastSearch', 'BLAST this protein','SBI_08953')" />

 
 
   EFLLRAPENLDLLDIRPQDRPGAEQPEALRRALLESVGADPEADTPVARLTGAEAQKALRTAYRRGLTRVAVRDVGALDPVEHMPAVGRQLADLAGAALE AALAVARAQAHERWDEALVEQVRLAVIGMGKCGARELNYISDVDVVYVVETRPAESGEVPDVSTATEIATELAHVLAHTVMATGPEPALWEVDANLRPEG KDGPLVRTLDSHVRYYKRWAKSWEFQALLKARCVAGDRELGERYEDAIAPFIWSSAEREGFVESVQAMRRRVTENIPEADRDRQIKLGPGGLRDVEFTVQ LLQLVHGRGDETVRTRATTDSLTALSSGGYIGRKDAEEFGRNYRWLRLLEHRIQLFRLRRTHLMPRDERELAVIARSVDGAREGSHPTAARLVEQWSALK RSVRSMHERIFYRPMLAALANAPADTTLTTDSARDRLAALGYQDTRAAMRHIEALSRGVSRRAEILRTLLPVLLDWLAEGVDPDAGLLNLRRVCESLGTT PWFLRLLRDSNAAAERLCHILSSSRYVSDLLEVSPEATAWLGDDSTLATRSLEDLGTEMRSQLSRHREPADAIRAVRQIRRREQLRIALADTSGVADVEA TVRGLSDVDQATVMAALHLAQKEWETENGEPAPAHVLVIAMGRQGGREIGFGSDADVMYAYEPAEGAEPGAAQAAAESVLARMVKLLKQPCTPPIVAERV LEIDNDLRPEGRSGAKVRSVASYAEYYERWAETWEAQALTRARYMAGSEEVAHAFFRVADRHRYPAEFTDRQLVDIRRMKARVENERMPRGADPLRQVKL GRGGLSDVEWLVQLLQLQHAHEVPELRTTSTLPALHAAVAAELVGPEDASVLEHAWKLATDIRNGNVLRSGRASDSVPSRRADLEAVARWIGYEPGSATQ LEEDYLRITRQSRSVFERLFYGR', 'http://blast.ncbi.nlm.nih.gov/Blast.cgi?PAGE=Proteins&PROGRAM=blastp&BLAST_PROGRAMS=blastp&QUERY=MSDTAGTARERAERISAKDLIAIGFADVTNARRWLGFGELDAVDLAALLTGLSHAASPDVALQLIVRLIEQHPQIAERINGDPADAETMYRLLGASEALG EFLLRAPENLDLLDIRPQDRPGAEQPEALRRALLESVGADPEADTPVARLTGAEAQKALRTAYRRGLTRVAVRDVGALDPVEHMPAVGRQLADLAGAALE AALAVARAQAHERWDEALVEQVRLAVIGMGKCGARELNYISDVDVVYVVETRPAESGEVPDVSTATEIATELAHVLAHTVMATGPEPALWEVDANLRPEG KDGPLVRTLDSHVRYYKRWAKSWEFQALLKARCVAGDRELGERYEDAIAPFIWSSAEREGFVESVQAMRRRVTENIPEADRDRQIKLGPGGLRDVEFTVQ LLQLVHGRGDETVRTRATTDSLTALSSGGYIGRKDAEEFGRNYRWLRLLEHRIQLFRLRRTHLMPRDERELAVIARSVDGAREGSHPTAARLVEQWSALK RSVRSMHERIFYRPMLAALANAPADTTLTTDSARDRLAALGYQDTRAAMRHIEALSRGVSRRAEILRTLLPVLLDWLAEGVDPDAGLLNLRRVCESLGTT PWFLRLLRDSNAAAERLCHILSSSRYVSDLLEVSPEATAWLGDDSTLATRSLEDLGTEMRSQLSRHREPADAIRAVRQIRRREQLRIALADTSGVADVEA TVRGLSDVDQATVMAALHLAQKEWETENGEPAPAHVLVIAMGRQGGREIGFGSDADVMYAYEPAEGAEPGAAQAAAESVLARMVKLLKQPCTPPIVAERV LEIDNDLRPEGRSGAKVRSVASYAEYYERWAETWEAQALTRARYMAGSEEVAHAFFRVADRHRYPAEFTDRQLVDIRRMKARVENERMPRGADPLRQVKL GRGGLSDVEWLVQLLQLQHAHEVPELRTTSTLPALHAAVAAELVGPEDASVLEHAWKLATDIRNGNVLRSGRASDSVPSRRADLEAVARWIGYEPGSATQ LEEDYLRITRQSRSVFERLFYGR&LINK_LOC=protein&PAGE_TYPE=BlastSearch', 'BLAST this protein','KRH_10900')" />
   RELAQDSTLRGRLLGVLGASPALADHLIANPSRWRLLRRSDGVVPDAPARRKPEPLPTGQELAEELFAVLDAHTDERLLQTRLRGVYRDWQLRLAARDVA ATVEDEPVLPLMAVAEHLSDMADAALAALLEFAKRKIVPPHEQAPKIAVIAMGKHGARELNYVSDVDVIFVAEPAGQISDRIAAELMRACSYVSFVVDAG LRPEGRHGALTRTLESHRKYYTNWAKPWEFQALLKARPAVGDMALGQAWFDALSPMVWKVAEHEDFVDEVRAMRRRVEESVPPPLREREIKLGRGSLRDV EFAVQLLQLVHGGPDPNLRLRATIPALAALAAGGYISRDDAANLTASYEFLRLLEHRLQMKQMQRTHTLPEDSDEEAMRWLARAAHLRPDGQHDALGVLR ECVRHERRRVLRLHSKFFYQPLLAAVSHRPQLGLSEQGAVRQLRALGYARPEAAFGHLKALTTGHRRANQIQGVLLPTLLEWLGETPDPDGGLLSYRKIS EAFADHPWYLRTLRDETAAAKRLMRVLGVSAFVPELMLRAPEVLRLYADGPHGPRLLSTNTDEVTSALLTSSAKHKDLHAAVSAARGARRYELARVASAD LLGVMELPSVCWALSRAWAATINAALAAAVRATTPEGEAPPAQIAVIGMGRLGGGELGYGSDADVLFVCEPRAGADEHEAIKWATGVAERVRSLLAAPSP DPSLHVDVDLRPEGRGGPLVCTLAAYDRYYKTRAQAWEAQALLRAHQIAGDQELGVKFLHLVDPVRYPPGGIGSEAVREIRRIKARVDTERLPRGADPTT HTKLGRGGLADVEWAVQLIQLRHAGAVPSLHNTSTMQTLAAIEAENLLPAEDVAQLREAWVTATKARNALVLVKGKSIDQLPGPGPVLAAVASAAGWADW EDSGAFLDNYLRVTRRAHNVVVRVLESETA', 'http://blast.ncbi.nlm.nih.gov/Blast.cgi?PAGE=Proteins&PROGRAM=blastp&BLAST_PROGRAMS=blastp&QUERY=MPERTGVPTLARLGLVATSAQEDVARLGWADEASVGLLWALSRAANPDCALRALSRLADALDSAEAEGPAGPGSPTGCAGSQSPTGCAGSQSPTGWAELG RELAQDSTLRGRLLGVLGASPALADHLIANPSRWRLLRRSDGVVPDAPARRKPEPLPTGQELAEELFAVLDAHTDERLLQTRLRGVYRDWQLRLAARDVA ATVEDEPVLPLMAVAEHLSDMADAALAALLEFAKRKIVPPHEQAPKIAVIAMGKHGARELNYVSDVDVIFVAEPAGQISDRIAAELMRACSYVSFVVDAG LRPEGRHGALTRTLESHRKYYTNWAKPWEFQALLKARPAVGDMALGQAWFDALSPMVWKVAEHEDFVDEVRAMRRRVEESVPPPLREREIKLGRGSLRDV EFAVQLLQLVHGGPDPNLRLRATIPALAALAAGGYISRDDAANLTASYEFLRLLEHRLQMKQMQRTHTLPEDSDEEAMRWLARAAHLRPDGQHDALGVLR ECVRHERRRVLRLHSKFFYQPLLAAVSHRPQLGLSEQGAVRQLRALGYARPEAAFGHLKALTTGHRRANQIQGVLLPTLLEWLGETPDPDGGLLSYRKIS EAFADHPWYLRTLRDETAAAKRLMRVLGVSAFVPELMLRAPEVLRLYADGPHGPRLLSTNTDEVTSALLTSSAKHKDLHAAVSAARGARRYELARVASAD LLGVMELPSVCWALSRAWAATINAALAAAVRATTPEGEAPPAQIAVIGMGRLGGGELGYGSDADVLFVCEPRAGADEHEAIKWATGVAERVRSLLAAPSP DPSLHVDVDLRPEGRGGPLVCTLAAYDRYYKTRAQAWEAQALLRAHQIAGDQELGVKFLHLVDPVRYPPGGIGSEAVREIRRIKARVDTERLPRGADPTT HTKLGRGGLADVEWAVQLIQLRHAGAVPSLHNTSTMQTLAAIEAENLLPAEDVAQLREAWVTATKARNALVLVKGKSIDQLPGPGPVLAAVASAAGWADW EDSGAFLDNYLRVTRRAHNVVVRVLESETA&LINK_LOC=protein&PAGE_TYPE=BlastSearch', 'BLAST this protein','Srot_2760')" />
   EHLARHPGDWRVLSGDGAIQAPDPRDLRDSLLRAVGADPADPAPTAADASTETLVALRAVYRRHLLHLAGRDLTGVLDVGEVAAELADLASAALEAGLAV ARTEVPEADSCRLAVIGMGKCGGRELNYVSDVDVIFVAEPRPGHDETEALRAASRLASGLMRACSASTPEGALWEVDAALRPEGKAGPLVRTLASHRAYY ERWAKTWEFQALLKARPVAGDMELGADYIDAIAPLVWKAAAGEEFVEDIQAMRRRVEEHLYKRTEETDRQLKLGPGGLRDVEFAVQLLQLVHGRADETLR SRGTLEALSLLSEGGYVGRDDAAGLASAYRFLRNVEHLLQLHRLRRTHTVPDDPADLRRLGRALGLRTDPVGEFTRLWRRHARTVRTLHEKLFYRPLLRA VARLPEEEARLTPEAARTRLVALGFTDPAGALRHIQALTSGVSRRAAIQRTLLPVMLGWFASAPDPDAGLLGFRQVSDALGTTPWYLRLLRDDVTVAERM AKVLASSRYATDLLLRAPEAVAILGNDAELAPRPFEALLNEALAAVRRREDDDRDAHASAEDAVAAVRGLRRRELFRISVGDLLGLIDVRTVGEALTDIA TVTIEAALQAAINKIEMERRGPLPTRITVVAMGRFGGRELGYGSDADVMFVHDPLPGADERDAQNAAHAVAEEMRRLLSRPAPDPPLQIDPNLRPEGKAG PLVRTLASYEAYYSRWSSPWEAQALLRADPVIGDAELGHRFRALIDPIRWPRDGISDAALREIRRLKARMESERLPHGVERRLHTKLGPGGLSDVEWVAQ LLQLQHAHEVAGLRTTSTLAALDAAVEAGLLDPADGEVLSSAWKLATRIRGTMMLVRGKASDLLPTDHHRERTAVTRVLGYPGTGDLLEDYRRHARRARR VMERVFYGISD', 'http://blast.ncbi.nlm.nih.gov/Blast.cgi?PAGE=Proteins&PROGRAM=blastp&BLAST_PROGRAMS=blastp&QUERY=MSEPWTSERRPSSLTGRLARLGFTDAGRAERLLAEAAAEGVTVTDAMLDALGGAADPDLALSSLLRLLGELDAPERAALQADEGLRRRLPAVLGVSAALG EHLARHPGDWRVLSGDGAIQAPDPRDLRDSLLRAVGADPADPAPTAADASTETLVALRAVYRRHLLHLAGRDLTGVLDVGEVAAELADLASAALEAGLAV ARTEVPEADSCRLAVIGMGKCGGRELNYVSDVDVIFVAEPRPGHDETEALRAASRLASGLMRACSASTPEGALWEVDAALRPEGKAGPLVRTLASHRAYY ERWAKTWEFQALLKARPVAGDMELGADYIDAIAPLVWKAAAGEEFVEDIQAMRRRVEEHLYKRTEETDRQLKLGPGGLRDVEFAVQLLQLVHGRADETLR SRGTLEALSLLSEGGYVGRDDAAGLASAYRFLRNVEHLLQLHRLRRTHTVPDDPADLRRLGRALGLRTDPVGEFTRLWRRHARTVRTLHEKLFYRPLLRA VARLPEEEARLTPEAARTRLVALGFTDPAGALRHIQALTSGVSRRAAIQRTLLPVMLGWFASAPDPDAGLLGFRQVSDALGTTPWYLRLLRDDVTVAERM AKVLASSRYATDLLLRAPEAVAILGNDAELAPRPFEALLNEALAAVRRREDDDRDAHASAEDAVAAVRGLRRRELFRISVGDLLGLIDVRTVGEALTDIA TVTIEAALQAAINKIEMERRGPLPTRITVVAMGRFGGRELGYGSDADVMFVHDPLPGADERDAQNAAHAVAEEMRRLLSRPAPDPPLQIDPNLRPEGKAG PLVRTLASYEAYYSRWSSPWEAQALLRADPVIGDAELGHRFRALIDPIRWPRDGISDAALREIRRLKARMESERLPHGVERRLHTKLGPGGLSDVEWVAQ LLQLQHAHEVAGLRTTSTLAALDAAVEAGLLDPADGEVLSSAWKLATRIRGTMMLVRGKASDLLPTDHHRERTAVTRVLGYPGTGDLLEDYRRHARRARR VMERVFYGISD&LINK_LOC=protein&PAGE_TYPE=BlastSearch', 'BLAST this protein','Tcur_3105')" />

 
 
   IADLVVNHTSVEHPWFQRARQSRDDPFHDFYVWRDDEPPDTSDQVVFPDQEKGVWTFNEPTGEWYLHKFYKEQPDLNVANPRVRDEVAKVMGFWLQLGLS GFRVDAVPFFLETAGTDGEALPEPHEYLRDLRSVVGRRTGDGVLLGEVNLPYEQQLEFFGGTDGDELTMQFDFIGMQALYLSLARADAGPLATALAGRPA LDPDSQWATFVRNHDELTLDKLTDDERQEVFAAFGPEERMQVYGRGLRRRLPPMLDGDPRRVRMVYSLLFSLPGTPVLFYGEEIGMGEDLDAEGRLAVRT PMQWTSGRNGGFSTADPDRLPGPVVDGGFAPEFVNVADQRRDEDSLLSFTKLLIRRYRESPELGWGGFELLEQPHREVLAHLCSWDDGALVAVHNLGPEP RTVPLMLPGCDAGHRLEDLLVTQTTPVGEDGGVELTLDGYGYRWLRVVGPDDRRLV', 'http://blast.ncbi.nlm.nih.gov/Blast.cgi?PAGE=Proteins&PROGRAM=blastp&BLAST_PROGRAMS=blastp&QUERY=MRITDTADLWWKNAVVYCLDVETYMDWNGDGCGDLPGLAQRIDHLADLGVTCLWLMPFYPTAERDDGYDITDFYGVDRRLGTHGDLVEVIRTAQDRGMRV IADLVVNHTSVEHPWFQRARQSRDDPFHDFYVWRDDEPPDTSDQVVFPDQEKGVWTFNEPTGEWYLHKFYKEQPDLNVANPRVRDEVAKVMGFWLQLGLS GFRVDAVPFFLETAGTDGEALPEPHEYLRDLRSVVGRRTGDGVLLGEVNLPYEQQLEFFGGTDGDELTMQFDFIGMQALYLSLARADAGPLATALAGRPA LDPDSQWATFVRNHDELTLDKLTDDERQEVFAAFGPEERMQVYGRGLRRRLPPMLDGDPRRVRMVYSLLFSLPGTPVLFYGEEIGMGEDLDAEGRLAVRT PMQWTSGRNGGFSTADPDRLPGPVVDGGFAPEFVNVADQRRDEDSLLSFTKLLIRRYRESPELGWGGFELLEQPHREVLAHLCSWDDGALVAVHNLGPEP RTVPLMLPGCDAGHRLEDLLVTQTTPVGEDGGVELTLDGYGYRWLRVVGPDDRRLV&LINK_LOC=protein&PAGE_TYPE=BlastSearch', 'BLAST this protein','Gobs_3079')" />
   IADLVVNHTSDEHPWFKEARSSRDSAKRDWYIWKDDPDPVDPKDLVFPDKEDSIWELDEKTGQYYLHSFYRFQPDLNVANPEVRDEIARVLGFWMELGLS GFRVDAVPFLIEGIGPDPHEFLADLRAFMNRRDGTSILLGEVNLPYPDLMEYFGDGNGDQVTMCFDFIGMQRFHLSMARQNPQALAESLRERPAPPDDSH WATFVRNHDELTLDKLTEAEKHEVFDAFGPDKDMQVYDRGLRRRLPPMLGGDQRRLKLAYSVLFSLPGTPVLFYGEEIGLGENLEAEGRMAVRIPMQWSR SGGFSPDPKTAVPTPEGEYGPRHVNVDDQRRDPDSMLKWITMLVERYRESPELAWGRYEVLDAGDEAVLAHRADAEGGTVIAVHNFADREAEVELVLRDL DHCEVLTDLLVDGTLDLPSDGRVKFALEPYGTRWLRAAVPDAPLGNSSAKSH', 'http://blast.ncbi.nlm.nih.gov/Blast.cgi?PAGE=Proteins&PROGRAM=blastp&BLAST_PROGRAMS=blastp&QUERY=MRLTYTADLWWKNAVVYCLDVETYKDGNGDGIGDFRGLTQQIDHLDRLGVTCIWLMPFFPTPNRDDGYDITDFYSVDPRLGTLGDFVEFMRTARDRGIRV IADLVVNHTSDEHPWFKEARSSRDSAKRDWYIWKDDPDPVDPKDLVFPDKEDSIWELDEKTGQYYLHSFYRFQPDLNVANPEVRDEIARVLGFWMELGLS GFRVDAVPFLIEGIGPDPHEFLADLRAFMNRRDGTSILLGEVNLPYPDLMEYFGDGNGDQVTMCFDFIGMQRFHLSMARQNPQALAESLRERPAPPDDSH WATFVRNHDELTLDKLTEAEKHEVFDAFGPDKDMQVYDRGLRRRLPPMLGGDQRRLKLAYSVLFSLPGTPVLFYGEEIGLGENLEAEGRMAVRIPMQWSR SGGFSPDPKTAVPTPEGEYGPRHVNVDDQRRDPDSMLKWITMLVERYRESPELAWGRYEVLDAGDEAVLAHRADAEGGTVIAVHNFADREAEVELVLRDL DHCEVLTDLLVDGTLDLPSDGRVKFALEPYGTRWLRAAVPDAPLGNSSAKSH&LINK_LOC=protein&PAGE_TYPE=BlastSearch', 'BLAST this protein','Sros_8808')" />

 
 
   RVTVELPGQAAVPVTPQQLEGDERDQAWQRIAAAQPRIAKYQSKSERQYPVIRLRPRRP', 'http://blast.ncbi.nlm.nih.gov/Blast.cgi?PAGE=Proteins&PROGRAM=blastp&BLAST_PROGRAMS=blastp&QUERY=MTQNEQMSGRRPRKPGTPGAFSRWMQRTTNARMNRKIRNGKGQFMGMDVLILNTVGRRSGQPRETPLAWFPEGENGWLVVASGGGSQHPDWHANLTAHPD RVTVELPGQAAVPVTPQQLEGDERDQAWQRIAAAQPRIAKYQSKSERQYPVIRLRPRRP&LINK_LOC=protein&PAGE_TYPE=BlastSearch', 'BLAST this protein','Kfla_3191')" />
   TAAPRFAQYQVKTDRELPIIRLIPRSPD', 'http://blast.ncbi.nlm.nih.gov/Blast.cgi?PAGE=Proteins&PROGRAM=blastp&BLAST_PROGRAMS=blastp&QUERY=MKRIRRKGGGKLMGMNALVLTTTGRKSGEPRESPVGWFPGQDGSWIIVASAAGAAKNPAWYYNLSAHPDKAQVQIGSRKVDVVAEQLHGEERIGAWRQIT TAAPRFAQYQVKTDRELPIIRLIPRSPD&LINK_LOC=protein&PAGE_TYPE=BlastSearch', 'BLAST this protein','SBI_01539')" />

 
 
   EPIGVTDLRDEQGYPSLDSEAVVIDSDKTRLVTSEIEPSIRLYDESGRNPVKLDMPKRLLVKPAGRAPDRNLTLEGLTLQPGGQTLIASMEGPLEGDGKD TAGHTLVRLQSWHRGQGQAAKDFKIAAQYGYPIDKNPDNANKDLGISEIAATPDGRLLVLERGHSPQGNSARLYLADLSEATDVTDTENLPGSAHLVKKT LLADLVTCPDLGAPAKQPQHNKLLDNIEGMTVTGHTPDGKLNLLLVSDDNANVVQITRLYALTAKLPSSGS', 'http://blast.ncbi.nlm.nih.gov/Blast.cgi?PAGE=Proteins&PROGRAM=blastp&BLAST_PROGRAMS=blastp&QUERY=MRRHLRIALATATAALALCSSLGVAGGSATARAQNDDHKCSDNVDVDGFSDGLDNRLYKKRYVGNLSGLAVDSDGSIAAVSDRSLLFRLKVQSQDGNPSA EPIGVTDLRDEQGYPSLDSEAVVIDSDKTRLVTSEIEPSIRLYDESGRNPVKLDMPKRLLVKPAGRAPDRNLTLEGLTLQPGGQTLIASMEGPLEGDGKD TAGHTLVRLQSWHRGQGQAAKDFKIAAQYGYPIDKNPDNANKDLGISEIAATPDGRLLVLERGHSPQGNSARLYLADLSEATDVTDTENLPGSAHLVKKT LLADLVTCPDLGAPAKQPQHNKLLDNIEGMTVTGHTPDGKLNLLLVSDDNANVVQITRLYALTAKLPSSGS&LINK_LOC=protein&PAGE_TYPE=BlastSearch', 'BLAST this protein','SBI_06944')" />

 
 
   VAYVDNDPIVNTYANVLLRGSSATSIALADLRDPRAVLDHPEVRQVIDFDEPVALLLVAIVHFLTEAEEPERVLATLRDGLPTGSFLVLSHATGDFADRS AAQAVYDNATATLNLRSRAEVEGFFGGFTLVEPGLAEVPFWRPDAPPPTRPGEIGYYGGVARKTG', 'http://blast.ncbi.nlm.nih.gov/Blast.cgi?PAGE=Proteins&PROGRAM=blastp&BLAST_PROGRAMS=blastp&QUERY=MTENGFRAEEIDTSRPHPARIYDYLLGGKNNYEVDREAGDRLAAAAPEVWTGVRANRDFLRRAVRYVVGSGIRQILDIGTGLPSSPNVHEIARELAPDVR VAYVDNDPIVNTYANVLLRGSSATSIALADLRDPRAVLDHPEVRQVIDFDEPVALLLVAIVHFLTEAEEPERVLATLRDGLPTGSFLVLSHATGDFADRS AAQAVYDNATATLNLRSRAEVEGFFGGFTLVEPGLAEVPFWRPDAPPPTRPGEIGYYGGVARKTG&LINK_LOC=protein&PAGE_TYPE=BlastSearch', 'BLAST this protein','Strvi_3263')" />

 
 
   AVARSILEIGRFCRQNGIDAQYENTGILQVATDTKQLARLELQIKRAQRAGVTSFRLLDREQAQERIGSPTVLGALKVSGALLNPYRLVRGLARVVQEQG VLLHEQTPAQTIEKVDGKWRITTPHGTITANEVIVATEAMQGAFPELYARQMPVWNYLLVTEPLTDEQLGRVPWPGREGVANSLSFSTAARLTADNRVLW AGGLWYMFGNRDTDPRHRRNDDAFDKLGASFREFFPQWRDVRFSHGNGGLISWSHSFIPQFGRTDSGMVYGHGYTGSGIAASHTGGKILRDLVLRRKTEF TELAFVTVNQPKFLPAPFGDKGGEFFIWRQRVGDRLPLLLPYKSALAPNRFFARRTGSPERATAGRSSD', 'http://blast.ncbi.nlm.nih.gov/Blast.cgi?PAGE=Proteins&PROGRAM=blastp&BLAST_PROGRAMS=blastp&QUERY=MAQLSTEVYWHSTEPTEVGPPLDGDASCDVCIVGAGYTGLWAAHFLKQAEPGMSVRVVEAKFAGEGASGMNAGFVQMTAGKVLRRMLWYYGAEKAGGVYR AVARSILEIGRFCRQNGIDAQYENTGILQVATDTKQLARLELQIKRAQRAGVTSFRLLDREQAQERIGSPTVLGALKVSGALLNPYRLVRGLARVVQEQG VLLHEQTPAQTIEKVDGKWRITTPHGTITANEVIVATEAMQGAFPELYARQMPVWNYLLVTEPLTDEQLGRVPWPGREGVANSLSFSTAARLTADNRVLW AGGLWYMFGNRDTDPRHRRNDDAFDKLGASFREFFPQWRDVRFSHGNGGLISWSHSFIPQFGRTDSGMVYGHGYTGSGIAASHTGGKILRDLVLRRKTEF TELAFVTVNQPKFLPAPFGDKGGEFFIWRQRVGDRLPLLLPYKSALAPNRFFARRTGSPERATAGRSSD&LINK_LOC=protein&PAGE_TYPE=BlastSearch', 'BLAST this protein','VAB18032_07100')" />

 
 
   VLDGIERLIDP', 'http://blast.ncbi.nlm.nih.gov/Blast.cgi?PAGE=Proteins&PROGRAM=blastp&BLAST_PROGRAMS=blastp&QUERY=MDGPNALALNERLLAALADGGVAAANAARSAYLLIVYVLGAIALEAAEPHEPGTTEAERIAARRDAFAAVPVEHYPRTASQIDVLAAYVTTEQFSWGLDR VLDGIERLIDP&LINK_LOC=protein&PAGE_TYPE=BlastSearch', 'BLAST this protein','Francci3_1975')" />

 
 
   QDVRDMEPGHPA', 'http://blast.ncbi.nlm.nih.gov/Blast.cgi?PAGE=Proteins&PROGRAM=blastp&BLAST_PROGRAMS=blastp&QUERY=MGPSHCPRERPAQATHLAVHLLDVADRELSELFGELTGDEVDKFARCRWSGGPFGMPVLAGPRAWFAGPVMTTAEVGDHTAFVIDPVAGKFHGPLRQLGF QDVRDMEPGHPA&LINK_LOC=protein&PAGE_TYPE=BlastSearch', 'BLAST this protein','Franean1_2400')" />
   RRNHTFRTARDATHLAVHVFDHDHLDVAELFGSQTSDKVHKFDRCSWHPGPHRLPILDDAAAWFAGEILDRFSLGDHVGHLLAPVDGSPPRQLESWVSFG DLRHLEPGHEA', 'http://blast.ncbi.nlm.nih.gov/Blast.cgi?PAGE=Proteins&PROGRAM=blastp&BLAST_PROGRAMS=blastp&QUERY=MAAAQRRLGWLSPCCQTIRHTGRPRRGATAGRCGWFRDGPTGKTEQMADPRTDPFEKLVALLNYPMFVVTTQSDGTPAGCLVGFASQASIHPPRFLVGLS RRNHTFRTARDATHLAVHVFDHDHLDVAELFGSQTSDKVHKFDRCSWHPGPHRLPILDDAAAWFAGEILDRFSLGDHVGHLLAPVDGSPPRQLESWVSFG DLRHLEPGHEA&LINK_LOC=protein&PAGE_TYPE=BlastSearch', 'BLAST this protein','MAP_1426c')" />
   WHPGPHRLPILDDAAAWFAGEILDRFSLGDHVGHLLAPVDGSPPRQLESWVSFGDVRHLEPGHEA', 'http://blast.ncbi.nlm.nih.gov/Blast.cgi?PAGE=Proteins&PROGRAM=blastp&BLAST_PROGRAMS=blastp&QUERY=MADPRTDPFEKLVALLNYPMFVVTTQSDGTPAGCLVGFASQASIHPPRFLVGLSRRNHTFRTARDATHLAVHVFDHDHLDVAELFGSQTSDKVDKFDRCS WHPGPHRLPILDDAAAWFAGEILDRFSLGDHVGHLLAPVDGSPPRQLESWVSFGDVRHLEPGHEA&LINK_LOC=protein&PAGE_TYPE=BlastSearch', 'BLAST this protein','MAV_3049')" />

 
 
   

 
 
   WMHQARAEGLGGAEVQELFRASFEEAYGDGSG', 'http://blast.ncbi.nlm.nih.gov/Blast.cgi?PAGE=Proteins&PROGRAM=blastp&BLAST_PROGRAMS=blastp&QUERY=MPVVVFRIDRRSGVATYLQIVRQVEQALRMGALEEGDRLPTAAQVAATTKVNPNTTLKAYRELERAGLAEVRQGAGTFITRSLAQPGSGPDWPLRSALNE WMHQARAEGLGGAEVQELFRASFEEAYGDGSG&LINK_LOC=protein&PAGE_TYPE=BlastSearch', 'BLAST this protein','SACTE_4656')" />

 
 
   

 
 
   FVEGVVWLIVLRLLLGMAVGADYPIATSLMTEFAPRKYRGPLLGAFVTMWFVGAAAAYVVGEVLARTAGDDAWRWMLASAAFPAVLIVVARVGTPESPRW LVSKGKIDKANEVLLKVYGPGVTVADLPEEEESNVGVKELLRSGYGKRMAFITLFWTCSVIPLFAVYAFAPAILGALKLEGDAAHIGSAVITILFMVGCV VALFLVNRMGRRPLLIHSFVWSGLALLLLGIFPGAPSAIIMVLFAAYAVLIGGSQILQWVYPNELFPTEVRGSAVGLASSLSRIGAAIGTFLVPLSLSSL GIGVTMLIAAGITLFGAVISQMWAPETRGLSLSESAGLGQAAKSPAPAEAISV', 'http://blast.ncbi.nlm.nih.gov/Blast.cgi?PAGE=Proteins&PROGRAM=blastp&BLAST_PROGRAMS=blastp&QUERY=MTTNPLDDAPLSTFHKKLAVFSSGGPFLDGYALSIIGVAMVQISGQWNLSSAEQGLIAASTLIGILLGAFAGGWLTDRFGREVLFTLDLVAIIACSVAQF FVEGVVWLIVLRLLLGMAVGADYPIATSLMTEFAPRKYRGPLLGAFVTMWFVGAAAAYVVGEVLARTAGDDAWRWMLASAAFPAVLIVVARVGTPESPRW LVSKGKIDKANEVLLKVYGPGVTVADLPEEEESNVGVKELLRSGYGKRMAFITLFWTCSVIPLFAVYAFAPAILGALKLEGDAAHIGSAVITILFMVGCV VALFLVNRMGRRPLLIHSFVWSGLALLLLGIFPGAPSAIIMVLFAAYAVLIGGSQILQWVYPNELFPTEVRGSAVGLASSLSRIGAAIGTFLVPLSLSSL GIGVTMLIAAGITLFGAVISQMWAPETRGLSLSESAGLGQAAKSPAPAEAISV&LINK_LOC=protein&PAGE_TYPE=BlastSearch', 'BLAST this protein','ROP_24160')" />

 
 
   PGAFRVRVLARGEVTERLDPFLSPVRPLIED', 'http://blast.ncbi.nlm.nih.gov/Blast.cgi?PAGE=Proteins&PROGRAM=blastp&BLAST_PROGRAMS=blastp&QUERY=MYEFHGWFGIAESPEEADTGTLEQGIAELRERVEALDWSTGEAVLRAHNGEWFVRADRLVNRRRDEAEELDALVAFIARRFPGGWGLLYERSDDLPSPPG PGAFRVRVLARGEVTERLDPFLSPVRPLIED&LINK_LOC=protein&PAGE_TYPE=BlastSearch', 'BLAST this protein','SAV_3204')" />

 
 
   ESVYLTSLGMNAVALPVLVEMGIPRITAQAVIIAVSTFLSYFGHRHFSFRRSAADTQDDASNAERT', 'http://blast.ncbi.nlm.nih.gov/Blast.cgi?PAGE=Proteins&PROGRAM=blastp&BLAST_PROGRAMS=blastp&QUERY=MTEFDLPPEPAPAGPLIRLFRDQRVAFLVVGGINTVVGFAIFVACSESVGQFVDHRFGKVAGSLVTLGITHVLSVLFAFVMHRRFVFHVRGHVLRDLARF ESVYLTSLGMNAVALPVLVEMGIPRITAQAVIIAVSTFLSYFGHRHFSFRRSAADTQDDASNAERT&LINK_LOC=protein&PAGE_TYPE=BlastSearch', 'BLAST this protein','MMAR_2337')" />

 
 
   TGNDHAREFGLPTKNPKAAADIVVDGWTETIDLGRIQDDNGIEKWFGTVAATGFDSLVNDRANRMRWPHGRMRYYIAMLAELSRLRPLPFRLVLDGTEEI VADLTLADFGNTRSYGGGLLICPNADHSDGLLDITMAQSDSRTKLLRLFPTIFKGAHVELDEVSTTRAKTVHVECPGINVYADGDFACPLPAEISAVPAA LQVLRPRHG', 'http://blast.ncbi.nlm.nih.gov/Blast.cgi?PAGE=Proteins&PROGRAM=blastp&BLAST_PROGRAMS=blastp&QUERY=MSAGQLRRHEIGKVTALTNPLSGHGAAVKAAHGAIARLKHRGVDVVEIVGGDAHDARHLLAAAVAKGTDAVMVTGGDGVVSNALQVLAGTDIPLGIIPAG TGNDHAREFGLPTKNPKAAADIVVDGWTETIDLGRIQDDNGIEKWFGTVAATGFDSLVNDRANRMRWPHGRMRYYIAMLAELSRLRPLPFRLVLDGTEEI VADLTLADFGNTRSYGGGLLICPNADHSDGLLDITMAQSDSRTKLLRLFPTIFKGAHVELDEVSTTRAKTVHVECPGINVYADGDFACPLPAEISAVPAA LQVLRPRHG&LINK_LOC=protein&PAGE_TYPE=BlastSearch', 'BLAST this protein','MAF_22630')" />
   AGTGNDHAREFGIPTKDPEAAADIIVDGWAETVDLGRIRADNGFDKWFGTVAATGFDSLVTDRANRMRWPHGRLRYYVAMLAELSQLRLLPFRLVLDGAQ EIDAEITLAAFGNTRSYGGGMRICPAADHADGLLDITMVHEASRAKLVRLFPTVMTGTHVELEQVSTVRAKSIHVECPGINVYADGDFACPLPAEISAGS GRAADPAGRSLTHAPAQPGHLAGVAAVAECLERLVPGRPQHGIQLGGQRVGALGHHRAQPHLPDHDVAVGAHRPAPQTQLRGVAEAVAVDSGAGVLGDLE PQHGPGAQRVGQLGAGAHRTDPVGDRAQLSGHRRLGRPGQVGLGPQGRRQRPLDMRAHRRRRGVHVHHASRHVGELVRRSHFLLLRFHEGRLPQRPVDPA SRAACANLRFFLCRACLLCPVIPMLR', 'http://blast.ncbi.nlm.nih.gov/Blast.cgi?PAGE=Proteins&PROGRAM=blastp&BLAST_PROGRAMS=blastp&QUERY=MTSPAARLRRYEIGKVIALTNPVSGHGAAVAAAQRAIARLHRRGVEVVEIIGDDAQDARHLVGAALDKGADAVMVTGGDGVFSNALQVLAGTDIPAGIVP AGTGNDHAREFGIPTKDPEAAADIIVDGWAETVDLGRIRADNGFDKWFGTVAATGFDSLVTDRANRMRWPHGRLRYYVAMLAELSQLRLLPFRLVLDGAQ EIDAEITLAAFGNTRSYGGGMRICPAADHADGLLDITMVHEASRAKLVRLFPTVMTGTHVELEQVSTVRAKSIHVECPGINVYADGDFACPLPAEISAGS GRAADPAGRSLTHAPAQPGHLAGVAAVAECLERLVPGRPQHGIQLGGQRVGALGHHRAQPHLPDHDVAVGAHRPAPQTQLRGVAEAVAVDSGAGVLGDLE PQHGPGAQRVGQLGAGAHRTDPVGDRAQLSGHRRLGRPGQVGLGPQGRRQRPLDMRAHRRRRGVHVHHASRHVGELVRRSHFLLLRFHEGRLPQRPVDPA SRAACANLRFFLCRACLLCPVIPMLR&LINK_LOC=protein&PAGE_TYPE=BlastSearch', 'BLAST this protein','MAP_2005')" />
   AGTGNDHAREFGIPTKDPEAAADIIVDGWAETVDLGRIRADNGFDKWFGTVAATGFDSLVTDRANRMRWPHGRLRYYVAMLAELSQLRLLPFRLVLDGAR EIDADITLAAFGNTRSYGGGMRICPAADHADGLLDITMVHEASRAKLVRLFPTVMKGTHVELEQVSTARAKSIHVECPGINVYADGDFACPLPAEISAVP GALRILRAGP', 'http://blast.ncbi.nlm.nih.gov/Blast.cgi?PAGE=Proteins&PROGRAM=blastp&BLAST_PROGRAMS=blastp&QUERY=MTSPAARLRRYEIGKVIALTNPASGHGAAVAAAQRAIARLHRRGVEVVEIIGDDAQDARHLVGAALDKGADAVMVTGGDGVFSNALQVLAGTDIPAGIVP AGTGNDHAREFGIPTKDPEAAADIIVDGWAETVDLGRIRADNGFDKWFGTVAATGFDSLVTDRANRMRWPHGRLRYYVAMLAELSQLRLLPFRLVLDGAR EIDADITLAAFGNTRSYGGGMRICPAADHADGLLDITMVHEASRAKLVRLFPTVMKGTHVELEQVSTARAKSIHVECPGINVYADGDFACPLPAEISAVP GALRILRAGP&LINK_LOC=protein&PAGE_TYPE=BlastSearch', 'BLAST this protein','MAV_2184')" />
   TGNDHAREFGLPTKNPKAAADIVVDGWTETIDLGRIQDDNGIEKWFGTVAATGFDSLVNDRANRMRWPHGRMRYYIAMLAELSRLRPLPFRLVLDGTEEI VADLTLADFGNTRSYGGGLLICPNADHSDGLLDITMAQSDSRTKLLRLFPTIFKGAHVELDEVSTTRAKTVHVECPGINVYADGDFACPLPAEISAVPAA LQVLRPRHG', 'http://blast.ncbi.nlm.nih.gov/Blast.cgi?PAGE=Proteins&PROGRAM=blastp&BLAST_PROGRAMS=blastp&QUERY=MSAGQLRRHEIGKVTALTNPLSGHGAAVKAAHGAIARLKHRGVDVVEIVGGDAHDARHLLAAAVAKGTDAVMVTGGDGVVSNALQVLAGTDIPLGIIPAG TGNDHAREFGLPTKNPKAAADIVVDGWTETIDLGRIQDDNGIEKWFGTVAATGFDSLVNDRANRMRWPHGRMRYYIAMLAELSRLRPLPFRLVLDGTEEI VADLTLADFGNTRSYGGGLLICPNADHSDGLLDITMAQSDSRTKLLRLFPTIFKGAHVELDEVSTTRAKTVHVECPGINVYADGDFACPLPAEISAVPAA LQVLRPRHG&LINK_LOC=protein&PAGE_TYPE=BlastSearch', 'BLAST this protein','Mb_2276')" />
   TGNDHAREFGLPTKNPKAAADIVVDGWTETIDLGRIQDDNGIEKWFGTVAATGFDSLVNDRANRMRWPHGRMRYYIAMLAELSRLRPLPFRLVLDGTEEI VADLTLADFGNTRSYGGGLLICPNADHSDGLLDITMAQSDSRTKLLRLFPTIFKGAHVELDEVSTTRAKTVHVECPGINVYADGDFACPLPAEISAVPAA LQVLRPRHG', 'http://blast.ncbi.nlm.nih.gov/Blast.cgi?PAGE=Proteins&PROGRAM=blastp&BLAST_PROGRAMS=blastp&QUERY=MSAGQLRRHEIGKVTALTNPLSGHGAAVKAAHGAIARLKHRGVDVVEIVGGDAHDARHLLAAAVAKGTDAVMVTGGDGVVSNALQVLAGTDIPLGIIPAG TGNDHAREFGLPTKNPKAAADIVVDGWTETIDLGRIQDDNGIEKWFGTVAATGFDSLVNDRANRMRWPHGRMRYYIAMLAELSRLRPLPFRLVLDGTEEI VADLTLADFGNTRSYGGGLLICPNADHSDGLLDITMAQSDSRTKLLRLFPTIFKGAHVELDEVSTTRAKTVHVECPGINVYADGDFACPLPAEISAVPAA LQVLRPRHG&LINK_LOC=protein&PAGE_TYPE=BlastSearch', 'BLAST this protein','MCAN_22741')" />
   IPTGDPEAAADVVVDGVSDHVDLGRISGADGTVRWFGTVMAAGFDSLVTDRTNRMRWPHGRMRYNLAMVAEISKLRLLPFRLSFDGDEISTQLTLAAFGN TRSYGGGMKICPGADPRDGLLDVTMVASASRTRLIRLFPTVFKGTHVNLDEVSTRRARTITVDSPGINSYADGEYVCPLPVEVSAVPKALKILRPA', 'http://blast.ncbi.nlm.nih.gov/Blast.cgi?PAGE=Proteins&PROGRAM=blastp&BLAST_PROGRAMS=blastp&QUERY=MTRVTVLTNPASGHGSASHAAERAITRLHRRGVDVVAIAGRDAVHARQLVEGALERDMDALVVVGGDGIISLALQVLAQTDIPLGVIPAGTGNDHAREFG IPTGDPEAAADVVVDGVSDHVDLGRISGADGTVRWFGTVMAAGFDSLVTDRTNRMRWPHGRMRYNLAMVAEISKLRLLPFRLSFDGDEISTQLTLAAFGN TRSYGGGMKICPGADPRDGLLDVTMVASASRTRLIRLFPTVFKGTHVNLDEVSTRRARTITVDSPGINSYADGEYVCPLPVEVSAVPKALKILRPA&LINK_LOC=protein&PAGE_TYPE=BlastSearch', 'BLAST this protein','Mflv_2771')" />
   PLGIIPAGTGNDHAREFEIPTKDAEAAADIVVDGWTETIDLGRIQAGSGKDKCDKWFGTLAATGFDSLVTDRANRMTWPHGRLRYYIAMLVELSQLRPLP FRLVLDGTEEIETDLTLATFGNTRSYGGGMLMCPNADRTDGLLDITMVRSGSRSRFLRLFPTVVKGTHVELDEVTTARAKSIDVECPGINVYADGDYACP LPANISAVAGALQILRPNDR', 'http://blast.ncbi.nlm.nih.gov/Blast.cgi?PAGE=Proteins&PROGRAM=blastp&BLAST_PROGRAMS=blastp&QUERY=MTPENPVNPLAGQLRQRSISKVTALTNPLSGHGAAVQAAQHAIARLHHRGVEVIEIVGENAEDARYLLAAAVEKGTDAVVVTGGDGVISNALQVLAETDV PLGIIPAGTGNDHAREFEIPTKDAEAAADIVVDGWTETIDLGRIQAGSGKDKCDKWFGTLAATGFDSLVTDRANRMTWPHGRLRYYIAMLVELSQLRPLP FRLVLDGTEEIETDLTLATFGNTRSYGGGMLMCPNADRTDGLLDITMVRSGSRSRFLRLFPTVVKGTHVELDEVTTARAKSIDVECPGINVYADGDYACP LPANISAVAGALQILRPNDR&LINK_LOC=protein&PAGE_TYPE=BlastSearch', 'BLAST this protein','MMAR_3345')" />
   IPTGDPEAAADVVVDGVSDHVDLGRISGADGTVRWFGTVMAAGFDSLVTDRTNRMRWPHGRMRYNLAMVAEISKLRLLPFRLSFDGDEISTQLTLAAFGN TRSYGGGMKICPGADPRDGLLDVTMVASASRTRLIRLFPTVFKGTHVNLDEVSTRRARTITVDSPGINSYADGEYVCPLPVEVSAVPKALKILRPA', 'http://blast.ncbi.nlm.nih.gov/Blast.cgi?PAGE=Proteins&PROGRAM=blastp&BLAST_PROGRAMS=blastp&QUERY=MTRVTVLTNPASGHGSASHAAERAITRLHRRGVDVVAIAGRDAVHARQLVEGALERDMDALVVVGGDGIISLALQVLAQTDIPLGVIPAGTGNDHAREFG IPTGDPEAAADVVVDGVSDHVDLGRISGADGTVRWFGTVMAAGFDSLVTDRTNRMRWPHGRMRYNLAMVAEISKLRLLPFRLSFDGDEISTQLTLAAFGN TRSYGGGMKICPGADPRDGLLDVTMVASASRTRLIRLFPTVFKGTHVNLDEVSTRRARTITVDSPGINSYADGEYVCPLPVEVSAVPKALKILRPA&LINK_LOC=protein&PAGE_TYPE=BlastSearch', 'BLAST this protein','Mspyr1_22110')" />
   GNDHAREFEIPTKDAEAAADIVVDGWTETIDLGPIQAGSGKDKCDKWFGTLAATGFDSLVTDRANRMTWPHGRLRYYIAMLVELSQLRPLPFRLVLDGTE EIETDLTLATFGNTRSYGGGMLMCPNADRTDDLLDITMVRSGPRSSFLRLFPTVVKGTHVELDEVTTARAKSIDVECPGINVYADGDYACPLPANISAVA GALQILRPNDR', 'http://blast.ncbi.nlm.nih.gov/Blast.cgi?PAGE=Proteins&PROGRAM=blastp&BLAST_PROGRAMS=blastp&QUERY=MARQLRQRSISKVTALTNPLSGHGAAVQAAQHAIARLHHRGVEVIEIVGENAEDARYLLAAAVEKGTDAVVVTGGDGVISNALQVLAETDVPLGIIPAGT GNDHAREFEIPTKDAEAAADIVVDGWTETIDLGPIQAGSGKDKCDKWFGTLAATGFDSLVTDRANRMTWPHGRLRYYIAMLVELSQLRPLPFRLVLDGTE EIETDLTLATFGNTRSYGGGMLMCPNADRTDDLLDITMVRSGPRSSFLRLFPTVVKGTHVELDEVTTARAKSIDVECPGINVYADGDYACPLPANISAVA GALQILRPNDR&LINK_LOC=protein&PAGE_TYPE=BlastSearch', 'BLAST this protein','MUL_1297')" />
   VDLARITTATGEHRWFGAVLAAGFDAIVNERANRMRWPRGPRRYDLAILVELARLRPRRYTLRLDGETHELDAALVAVGNCASYGGGMRICPDADPTDGL LDIVVGGRFNRRMLIREKPNIYHGTHIHHPLVRSYRARTVELAAPDITTYADGERCLPLPITITATPAALHLLLP', 'http://blast.ncbi.nlm.nih.gov/Blast.cgi?PAGE=Proteins&PROGRAM=blastp&BLAST_PROGRAMS=blastp&QUERY=MLDRLSAGGRPVRVLDAYTRGQAEAVCHEAVADGAAALVAVGGDGTVHVALQAVAGTRVPFGAVPAGTGNDFAVETGFPADPLTAVDTIAAALRDGRSRP VDLARITTATGEHRWFGAVLAAGFDAIVNERANRMRWPRGPRRYDLAILVELARLRPRRYTLRLDGETHELDAALVAVGNCASYGGGMRICPDADPTDGL LDIVVGGRFNRRMLIREKPNIYHGTHIHHPLVRSYRARTVELAAPDITTYADGERCLPLPITITATPAALHLLLP&LINK_LOC=protein&PAGE_TYPE=BlastSearch', 'BLAST this protein','VAB18032_22705')" />

 
 
   ELRLAPNTVSGLVGQLIEGGLVAKRADPTDRRVAQLTVTPLGHDKLAVWRGAHEKRIGGALDRLEPEERADVVRALTALDHLVDHLRAS', 'http://blast.ncbi.nlm.nih.gov/Blast.cgi?PAGE=Proteins&PROGRAM=blastp&BLAST_PROGRAMS=blastp&QUERY=MDFPARGGPDVPARVPTTVRPPSDRPTERIHLGVGHNARRYPSRMSDEARTLTDVVARLRRALRTSIRSEWPWDALPMAQVELLMALAERSPSRVGDLAA ELRLAPNTVSGLVGQLIEGGLVAKRADPTDRRVAQLTVTPLGHDKLAVWRGAHEKRIGGALDRLEPEERADVVRALTALDHLVDHLRAS&LINK_LOC=protein&PAGE_TYPE=BlastSearch', 'BLAST this protein','Amir_1308')" />
   ARSQVERWRDRRNLAVAAAFDGLSAEDRAAIESAIPALARLAGALHPDRAEDSDQEVAR', 'http://blast.ncbi.nlm.nih.gov/Blast.cgi?PAGE=Proteins&PROGRAM=blastp&BLAST_PROGRAMS=blastp&QUERY=MAQNEAPAEQLFAAIGLLRRHTRRRVGRPWPETPLTGSQVELVRLLRRRPGTSVADAAAALGLAANTVSTLVRQLTDAGLIERVRDESDRRVARLALTDE ARSQVERWRDRRNLAVAAAFDGLSAEDRAAIESAIPALARLAGALHPDRAEDSDQEVAR&LINK_LOC=protein&PAGE_TYPE=BlastSearch', 'BLAST this protein','ROP_48810')" />

 
 
   RGLMHYGNDTSTVTEVVGEYNPTFICAAGAAIDQIKTGGVEKVVLARSLEVFADEDWKPEAVWQQLYQQNPAGHSFTISLPQDETSIVGNSPELIAGVRR GQLTSHPLAGSAPRLANRTEDQMAAAVLAGSAKNLAEHAFVVQHISQALRSVAKDLIVPSLPDLLPTGQMWHLGTLIRASLYKGLGSLDAALAIHPTPAI CGSPTAVAQALISELEPEDRGFYGGLVGWMNQDGEGEWALLLRSAMLTGNRAKLHAGAGIVADSDPFDEHSETAAKFRTMLCALGVDREGSNR', 'http://blast.ncbi.nlm.nih.gov/Blast.cgi?PAGE=Proteins&PROGRAM=blastp&BLAST_PROGRAMS=blastp&QUERY=MKTQLLESHHEVIANEDPSAPAPRTEAATFRFASYGRALIGHGTKAILDVERARRLGLGPAARQLLAEQPQGSILAGVVPFDRSADPYLFVPQNVEDLPM RGLMHYGNDTSTVTEVVGEYNPTFICAAGAAIDQIKTGGVEKVVLARSLEVFADEDWKPEAVWQQLYQQNPAGHSFTISLPQDETSIVGNSPELIAGVRR GQLTSHPLAGSAPRLANRTEDQMAAAVLAGSAKNLAEHAFVVQHISQALRSVAKDLIVPSLPDLLPTGQMWHLGTLIRASLYKGLGSLDAALAIHPTPAI CGSPTAVAQALISELEPEDRGFYGGLVGWMNQDGEGEWALLLRSAMLTGNRAKLHAGAGIVADSDPFDEHSETAAKFRTMLCALGVDREGSNR&LINK_LOC=protein&PAGE_TYPE=BlastSearch', 'BLAST this protein','AARI_32900')" />
   APSPDTHLNRVHAAVRALSSTTLQKVVLARAVELRASSPVDSSALLARLVHSDPNRNGFAINLSAAGGPWQGCHLIGASPEVLISRSGTTITCHPLAGSA PRDRDAELDRAHAANLRASSKDLAEHAFVVDQIRATLTPLCRELRTPRQPTLTSTRELWHLGTPIRGELRSASTTALDLALALHPTPAVCGTPTPAAQAF IRETEGDRGFYAGALGWTDAAGDGEWMVTIRCVTLASDGVTLTAHAGGGIVAESEPHEELDETTSKLRTIFDAFGVEQ', 'http://blast.ncbi.nlm.nih.gov/Blast.cgi?PAGE=Proteins&PROGRAM=blastp&BLAST_PROGRAMS=blastp&QUERY=MQAQVISQPARDFHGCLEPSSFVLSRPHGTVIAMGTAATFDCANSAAHALRSGKISAIAGALPFDPEHPAALTAPHDLLRYNTPLDPREVALPRLSVTGF APSPDTHLNRVHAAVRALSSTTLQKVVLARAVELRASSPVDSSALLARLVHSDPNRNGFAINLSAAGGPWQGCHLIGASPEVLISRSGTTITCHPLAGSA PRDRDAELDRAHAANLRASSKDLAEHAFVVDQIRATLTPLCRELRTPRQPTLTSTRELWHLGTPIRGELRSASTTALDLALALHPTPAVCGTPTPAAQAF IRETEGDRGFYAGALGWTDAAGDGEWMVTIRCVTLASDGVTLTAHAGGGIVAESEPHEELDETTSKLRTIFDAFGVEQ&LINK_LOC=protein&PAGE_TYPE=BlastSearch', 'BLAST this protein','AS9A_0137')" />
   PESFPPPDSPAYRRAVGQAVYEINVGHLDKVVLARRMTVEHAHPVDRDALFAHLAADNPQAFTYRVDLPGSSTFLGASPELVLRCQDGVATSVPLAGSAP RCPVTAPDHELINHRRRRQLLHSPKNLQEHSLVSRRVAEVFRAHAAEVTVPTGPGVVETPVIMHLASTITGRLHEGISPVELAYALHPTPAVCGWPTQQA AALINELESSDRGMYAGLVGWVDAEGNSEWALALRGGLVRDNGASGSTTTAFAGAGIVAGSDPDLEHAETDTKFRTFTRALSRTLAPTTI', 'http://blast.ncbi.nlm.nih.gov/Blast.cgi?PAGE=Proteins&PROGRAM=blastp&BLAST_PROGRAMS=blastp&QUERY=MIFEMSTPGYHLRTSGVRHRITPSDWSGPAVARAVHEALDIARTCSSDATTADGAPLVVGAIPFDTSTPAVLYVPERAQWRDPTTATTADQTIRPTPADA PESFPPPDSPAYRRAVGQAVYEINVGHLDKVVLARRMTVEHAHPVDRDALFAHLAADNPQAFTYRVDLPGSSTFLGASPELVLRCQDGVATSVPLAGSAP RCPVTAPDHELINHRRRRQLLHSPKNLQEHSLVSRRVAEVFRAHAAEVTVPTGPGVVETPVIMHLASTITGRLHEGISPVELAYALHPTPAVCGWPTQQA AALINELESSDRGMYAGLVGWVDAEGNSEWALALRGGLVRDNGASGSTTTAFAGAGIVAGSDPDLEHAETDTKFRTFTRALSRTLAPTTI&LINK_LOC=protein&PAGE_TYPE=BlastSearch', 'BLAST this protein','CVAR_2834')" />
   ADRAQSVPARLPMRRSPVQLLGARELPDPGGYLENVNQAIARLRGGQNAAKVVLGRWLDLETATPLDPLAVLGSLATTSRGAVRLFAVPAPAVPEGDSEP AILLGASPELLVSRRGRLVRSTPMAGSVPRSSDPATDALRAHALLDSVKDGDEHRYVADAVAAALRPLCDGLVVDGPKLMRTDTVWHLATEIHGRLSDPN LDLSALHLAQLLQPTPAVGGTPTDWALDVIAELEGARSCVAGAVGWVDAAGDGSYAVGIRSGLLSGRRLRLFAGAGIVAASDPESELMETEAKLATMLGG LGLSTAAVKGMIR', 'http://blast.ncbi.nlm.nih.gov/Blast.cgi?PAGE=Proteins&PROGRAM=blastp&BLAST_PROGRAMS=blastp&QUERY=MTATVPFATEPFAERLEPAVQPAIPPGMPLVFGSTDRLLQGRTTDRHWWSARSNDTAYADRIIADLQAESRPDPGASPATAIGTLSFRPDAPALFFPLAA ADRAQSVPARLPMRRSPVQLLGARELPDPGGYLENVNQAIARLRGGQNAAKVVLGRWLDLETATPLDPLAVLGSLATTSRGAVRLFAVPAPAVPEGDSEP AILLGASPELLVSRRGRLVRSTPMAGSVPRSSDPATDALRAHALLDSVKDGDEHRYVADAVAAALRPLCDGLVVDGPKLMRTDTVWHLATEIHGRLSDPN LDLSALHLAQLLQPTPAVGGTPTDWALDVIAELEGARSCVAGAVGWVDAAGDGSYAVGIRSGLLSGRRLRLFAGAGIVAASDPESELMETEAKLATMLGG LGLSTAAVKGMIR&LINK_LOC=protein&PAGE_TYPE=BlastSearch', 'BLAST this protein','MLP_27440')" />
   LFRDRSPLTAGGPPSGRRPRPDPPRIPGGGSLPDPPSRERSSRTAAEAPSSGPPPRHRLREEPSADDYARLVGLAVEHLSNEGLDKVVLARTLLLDLAEP LDPGRVLARLTADNPGAFNFAVAAGAFGSGEVFGEGSGGTDGRDRDPLRRPAPVLVGASPELLVARSGRRVRSHPLAGSAPRHPDPEADRERAEALLASA KDLREHAFVVEAIAERLRPLCSRLDVPEAPSLTATATMWHLGTPLSGLLREPLPSALELAAVLHPTPAVCGTPAEAAARAIGAWEPFDRGLYAGAVGWCD ADGDGEWAVTLRCAEIAGTRARLFAGAGIVADSVPAEEAAETGAKFRTMLNALGLGPVGPGSAGPDPGASGPGALDPAARVPPP', 'http://blast.ncbi.nlm.nih.gov/Blast.cgi?PAGE=Proteins&PROGRAM=blastp&BLAST_PROGRAMS=blastp&QUERY=MAPEPRGSLPGPFVLSTPDHTLTADGAPGRSRPLSGGGADLAKEAEAALRDARAAGWDDPRVVGALPFRTGAPAALMAADVVRTGPRPSGAPGGGSRRGP LFRDRSPLTAGGPPSGRRPRPDPPRIPGGGSLPDPPSRERSSRTAAEAPSSGPPPRHRLREEPSADDYARLVGLAVEHLSNEGLDKVVLARTLLLDLAEP LDPGRVLARLTADNPGAFNFAVAAGAFGSGEVFGEGSGGTDGRDRDPLRRPAPVLVGASPELLVARSGRRVRSHPLAGSAPRHPDPEADRERAEALLASA KDLREHAFVVEAIAERLRPLCSRLDVPEAPSLTATATMWHLGTPLSGLLREPLPSALELAAVLHPTPAVCGTPAEAAARAIGAWEPFDRGLYAGAVGWCD ADGDGEWAVTLRCAEIAGTRARLFAGAGIVADSVPAEEAAETGAKFRTMLNALGLGPVGPGSAGPDPGASGPGALDPAARVPPP&LINK_LOC=protein&PAGE_TYPE=BlastSearch', 'BLAST this protein','Ndas_1026')" />
   RLPAEDEHLARVRTALHRLRDDADPLRKVVLARMLRLRTHDRLDPEALLRNLVAADPAGNGYCVDLSAAGSASAGQWLVGSSPEVLIRKEGRAISCHPLA GSARRLDDPLADWESGRTLGESAKNLDEHRYVVDAIRDRLAPLCTELDVPERPTLTHTPQLWHLGTPIRGMLADTSMTALDLALAVHPTPAICGTPTRDA YETISALEGDRGFYAGAVGWCDERGDGEWMVTIRCARIDADGTGITAYAGGGIVADSVPEDELAETATKFTTILDALGVTA', 'http://blast.ncbi.nlm.nih.gov/Blast.cgi?PAGE=Proteins&PROGRAM=blastp&BLAST_PROGRAMS=blastp&QUERY=MPAPVVAAITPHRAGPVAVNPFVLSRPQGSIRAEGVLRHFDDAIEAADALRAGDVTALTGALPFDPDTPAAFVEPGLLWRSDKPWRSVATGDLPHCEVEQ RLPAEDEHLARVRTALHRLRDDADPLRKVVLARMLRLRTHDRLDPEALLRNLVAADPAGNGYCVDLSAAGSASAGQWLVGSSPEVLIRKEGRAISCHPLA GSARRLDDPLADWESGRTLGESAKNLDEHRYVVDAIRDRLAPLCTELDVPERPTLTHTPQLWHLGTPIRGMLADTSMTALDLALAVHPTPAICGTPTRDA YETISALEGDRGFYAGAVGWCDERGDGEWMVTIRCARIDADGTGITAYAGGGIVADSVPEDELAETATKFTTILDALGVTA&LINK_LOC=protein&PAGE_TYPE=BlastSearch', 'BLAST this protein','REQ_08130')" />
   SRVAGFEPSAQIHTDRVRGAIERLRAGELDKVVLARSLTIEAESTILPAALAEKLIALDHAHNGFCVDLSPAGGKYRGRSLVGSTPEVLIERRGDVVTCH PLAGSIARHPNSDQDEANAEQLRASTKDLAEHAFVVDSISAILGPLCTSFNAPSAPELLRTPQLWHLGTKIEGVLADPSLTSLELAMALHPTPAICGTPT GAAREHITATEGDRGFYAGAVGWCDRTGDGEWMVAIRCAEIAADGLSARAYAGGGIVASSDPEIELRETSTKFRTLLSAFDLAEHQL', 'http://blast.ncbi.nlm.nih.gov/Blast.cgi?PAGE=Proteins&PROGRAM=blastp&BLAST_PROGRAMS=blastp&QUERY=MSMSALAEVSEFPLRNNRTDDETDSVFLLSRRQTSIRTSGVVEEFDSAFDAAEALKTGRIDSVVGALPFSPETASALTAPLRFTRVRGDLYHSNVPALPT SRVAGFEPSAQIHTDRVRGAIERLRAGELDKVVLARSLTIEAESTILPAALAEKLIALDHAHNGFCVDLSPAGGKYRGRSLVGSTPEVLIERRGDVVTCH PLAGSIARHPNSDQDEANAEQLRASTKDLAEHAFVVDSISAILGPLCTSFNAPSAPELLRTPQLWHLGTKIEGVLADPSLTSLELAMALHPTPAICGTPT GAAREHITATEGDRGFYAGAVGWCDRTGDGEWMVAIRCAEIAADGLSARAYAGGGIVASSDPEIELRETSTKFRTLLSAFDLAEHQL&LINK_LOC=protein&PAGE_TYPE=BlastSearch', 'BLAST this protein','RER_26950')" />
   VPPEDEHLDRVAAAISVLREPEAALHKVVLARSVVLHAEEPLHPHTLLARLVANDLGGNGFSVDLGAAGTGWSGRHLVGSSPEVLIRRRGDTVTCHPLAG SAPRHADPEIDRQTAENLVSSEKNLREHALVVDALRASLEPFCTELDIPDRPTLTSTPQLWHLGTPVAGRLRDRSVTALDLAVAVHPTPAVCGTPTGAAR ALIGELEGDRGFYAGAVGWADADGDGEWMVTIRCAQIDADGTTVTAYAGGGIVAASEPDDELAETTTKLGTVLAALGLPR', 'http://blast.ncbi.nlm.nih.gov/Blast.cgi?PAGE=Proteins&PROGRAM=blastp&BLAST_PROGRAMS=blastp&QUERY=MESTAVAESTETPADLPPGAPFVLSRPHGTVIADGIRTGFDSARDAAAALKSGTVTSVAGALAFDPSHPAALVAPQTLRHHPGRWTPRPQTVPHVHAGAS VPPEDEHLDRVAAAISVLREPEAALHKVVLARSVVLHAEEPLHPHTLLARLVANDLGGNGFSVDLGAAGTGWSGRHLVGSSPEVLIRRRGDTVTCHPLAG SAPRHADPEIDRQTAENLVSSEKNLREHALVVDALRASLEPFCTELDIPDRPTLTSTPQLWHLGTPVAGRLRDRSVTALDLAVAVHPTPAVCGTPTGAAR ALIGELEGDRGFYAGAVGWADADGDGEWMVTIRCAQIDADGTTVTAYAGGGIVAASEPDDELAETTTKLGTVLAALGLPR&LINK_LOC=protein&PAGE_TYPE=BlastSearch', 'BLAST this protein','ROP_48860')" />
   SVRCAPPLTSDPLIALPASAPAAGDWRIRPVPEPETYGKGVAAAVERMWRGEFSKVVLARTLELTAKGPLDLPAMLQRLARRDPAGYTFALPTGPGRTLI GASPELLVSRHGKQVVANPLAGSTPRSADLAEDVRRAATLLESAKDLHEHAVVVDAVHQALAPYCTDLTVPARPTLIRTATMWHLSTTVTGTLASPDASA LELACALHPTPAVCGTPTSTARQVIAETEPFDRGFFTGMVGWGNADGDGEWVVTIRCADAEERTLRLYAGAGIVAASEPEAEAAETAAKFRTFLSAVGAE L', 'http://blast.ncbi.nlm.nih.gov/Blast.cgi?PAGE=Proteins&PROGRAM=blastp&BLAST_PROGRAMS=blastp&QUERY=MSTASQVATHTAPADPAHPAVGAATSLLDAYTPGDHFLATPRRTLLARGPGHRVPHDERPLTARVDATLAAAVATGQESPLVMGAIPFDHTAPAALHVPE SVRCAPPLTSDPLIALPASAPAAGDWRIRPVPEPETYGKGVAAAVERMWRGEFSKVVLARTLELTAKGPLDLPAMLQRLARRDPAGYTFALPTGPGRTLI GASPELLVSRHGKQVVANPLAGSTPRSADLAEDVRRAATLLESAKDLHEHAVVVDAVHQALAPYCTDLTVPARPTLIRTATMWHLSTTVTGTLASPDASA LELACALHPTPAVCGTPTSTARQVIAETEPFDRGFFTGMVGWGNADGDGEWVVTIRCADAEERTLRLYAGAGIVAASEPEAEAAETAAKFRTFLSAVGAE L&LINK_LOC=protein&PAGE_TYPE=BlastSearch', 'BLAST this protein','SGR_6733')" />
   LAGAEAAPTHSHDTPALCEPPPQHYRDAVATALADIDATELSKVVLARSLRIGLDAPVDIPAVLRQLRHRQRRGYVFACPLPEQRDLVGGSPELLVSRNG DVVTAHPLAGSRPRSGDPRVDTARGAELLASEKDRREHAVVVDAIARSLDPYCAELSAPSTPEIVSTGHMLHLGTRVTGRLRDPLPSALSLAAALHPTPA VCGTPTATAREAIARLEGFDRGFYSGMVGWCDSAGNGEWAVTIRCAEIGARDVRLFAGAGIVAGSDPAAELAETEAKLQTMLSALGHTGKVVDERAVHAM A', 'http://blast.ncbi.nlm.nih.gov/Blast.cgi?PAGE=Proteins&PROGRAM=blastp&BLAST_PROGRAMS=blastp&QUERY=MSTVAPRFADTPLSPLWSVHMHLVDEYRPGSSFFFDTGSRSLLTHGSAIVLPGRGGLAPARADTVLRALGGDQLLVGAVPFSDTEPAQLLVPDVYRWGPG LAGAEAAPTHSHDTPALCEPPPQHYRDAVATALADIDATELSKVVLARSLRIGLDAPVDIPAVLRQLRHRQRRGYVFACPLPEQRDLVGGSPELLVSRNG DVVTAHPLAGSRPRSGDPRVDTARGAELLASEKDRREHAVVVDAIARSLDPYCAELSAPSTPEIVSTGHMLHLGTRVTGRLRDPLPSALSLAAALHPTPA VCGTPTATAREAIARLEGFDRGFYSGMVGWCDSAGNGEWAVTIRCAEIGARDVRLFAGAGIVAGSDPAAELAETEAKLQTMLSALGHTGKVVDERAVHAM A&LINK_LOC=protein&PAGE_TYPE=BlastSearch', 'BLAST this protein','Snas_4515')" />
   ARRPRNGTWTATPVPDPAAHTAAVKRAVALLASPDSTRLRKVVLARCLRLTSSHDVDVAAVLANLAQANPGGFTFAADLPAPDSRPRTLVGASPELLVAK HGRAVVSRPLAGSRPRDTDPTRDRERGAALLASAKDRAEHAVVVEAVVEALRPYCRSLDVPDEPQPVTTPTMWHLATQVTGELRDPRTPVHVLAQALHPT PAVCGYPTDQAYAAIAELEPFDRGFYTGAVGYTDAAGDGEWVVTIRCADIHGATAELFAGGGIMPDSDPEAELAETTAKFRTLLLALGADQLR', 'http://blast.ncbi.nlm.nih.gov/Blast.cgi?PAGE=Proteins&PROGRAM=blastp&BLAST_PROGRAMS=blastp&QUERY=MPRQCVATPAATVTELLDAYRPRDFFFTSPSGSLLGQGVLTTVTGVDDLSDALHATAAEPGRHAVAVGALPFNRTAPARLVIPATVQRGPAIMPGTGATR ARRPRNGTWTATPVPDPAAHTAAVKRAVALLASPDSTRLRKVVLARCLRLTSSHDVDVAAVLANLAQANPGGFTFAADLPAPDSRPRTLVGASPELLVAK HGRAVVSRPLAGSRPRDTDPTRDRERGAALLASAKDRAEHAVVVEAVVEALRPYCRSLDVPDEPQPVTTPTMWHLATQVTGELRDPRTPVHVLAQALHPT PAVCGYPTDQAYAAIAELEPFDRGFYTGAVGYTDAAGDGEWVVTIRCADIHGATAELFAGGGIMPDSDPEAELAETTAKFRTLLLALGADQLR&LINK_LOC=protein&PAGE_TYPE=BlastSearch', 'BLAST this protein','Tfu_1872')" />

 
 
   AMCREISPELGITLPNRASPQQEGFVAAVDAESGREFDSSAVNIMRVTHGQIFPAIAKIRASTKNTLVRQLADLANDTVLDHITVLEKTGLVEYDDVTFK QTGPAKLPREKVTPPPPQPGERVLVLKPRPDLNVNTASPTPAPSPAAP', 'http://blast.ncbi.nlm.nih.gov/Blast.cgi?PAGE=Proteins&PROGRAM=blastp&BLAST_PROGRAMS=blastp&QUERY=MRLSRSRIGVVVLFGAMTLTLTALAFPAVLGLDKADGNQNRVVASTKFGPLTEADRDFVVKVRAAGLWEYPLGEMVMERGTTKAMKTAGEHLVVGHAGLD AMCREISPELGITLPNRASPQQEGFVAAVDAESGREFDSSAVNIMRVTHGQIFPAIAKIRASTKNTLVRQLADLANDTVLDHITVLEKTGLVEYDDVTFK QTGPAKLPREKVTPPPPQPGERVLVLKPRPDLNVNTASPTPAPSPAAP&LINK_LOC=protein&PAGE_TYPE=BlastSearch', 'BLAST this protein','SCO_5311')" />

 
 
   RRMNEMHRKYAITAEDFTAVSVETTLGQLTAAREYGWRELTEHEVRGVVEFERVQALHMNIRDVPATVAEMVAFRDDYFDRQVRFAPANRRLAESALRLL ATVVPAHIAPRVEKVLMSLTDPRIVAALGYDYPDAAYRERVHRATREHAAQDATDKRAPKVVLDLIASVYPDGYEIDRLGTHLRAEEASGPRDRNAPTPM CPV', 'http://blast.ncbi.nlm.nih.gov/Blast.cgi?PAGE=Proteins&PROGRAM=blastp&BLAST_PROGRAMS=blastp&QUERY=MSTSSHDRDPYALRDRILALDPVENAREIAHLFHRDFQRALLPQIATGFFATISPPRMTRILIRTGELEFRNRKRLVDQMLIHHEMLTHGFVPGRGRDAL RRMNEMHRKYAITAEDFTAVSVETTLGQLTAAREYGWRELTEHEVRGVVEFERVQALHMNIRDVPATVAEMVAFRDDYFDRQVRFAPANRRLAESALRLL ATVVPAHIAPRVEKVLMSLTDPRIVAALGYDYPDAAYRERVHRATREHAAQDATDKRAPKVVLDLIASVYPDGYEIDRLGTHLRAEEASGPRDRNAPTPM CPV&LINK_LOC=protein&PAGE_TYPE=BlastSearch', 'BLAST this protein','FRAAL_2985')" />
   DAYRYVLACFDLAPLRWCAAYAWRAPTDAERDASHTFYLALAERMGIPDVPPDRREFEAWTEDFERAHFTFTPEAHALWAATRDLLAGRVPGVLGPLAGS AADSLLDEGLRRALGVGRPAMPVRAATHTALRLRAATGRAGRRIRGSHRPAGMSMVQ', 'http://blast.ncbi.nlm.nih.gov/Blast.cgi?PAGE=Proteins&PROGRAM=blastp&BLAST_PROGRAMS=blastp&QUERY=MTPSPTALRQAEEIYRRVAFDTFAQDLKMGLNLGFCRTFAVPEIARLLTATGRMTRHTRARAKATGELMYRMFRHGLDGEEGERTVAALKSLHAPWAISD DAYRYVLACFDLAPLRWCAAYAWRAPTDAERDASHTFYLALAERMGIPDVPPDRREFEAWTEDFERAHFTFTPEAHALWAATRDLLAGRVPGVLGPLAGS AADSLLDEGLRRALGVGRPAMPVRAATHTALRLRAATGRAGRRIRGSHRPAGMSMVQ&LINK_LOC=protein&PAGE_TYPE=BlastSearch', 'BLAST this protein','Strvi_5126')" />

 
 
   HADHRVSGQAPHD', 'http://blast.ncbi.nlm.nih.gov/Blast.cgi?PAGE=Proteins&PROGRAM=blastp&BLAST_PROGRAMS=blastp&QUERY=MSSASGAHLRAPDGARLAEATGVFAMLSDATRLHLLWLLAQGESDVGSLADRCEASRTAVSQHLAKLRLAGLVDTRRAGRHIYYSLADGHLRRLVVEALS HADHRVSGQAPHD&LINK_LOC=protein&PAGE_TYPE=BlastSearch', 'BLAST this protein','SCAB_84461')" />

 
 
   IARFLGRAASTISRELRRNAATRAGRLDYRASVAQWKAELMAPRPKTAKLVVNERLRD', 'http://blast.ncbi.nlm.nih.gov/Blast.cgi?PAGE=Proteins&PROGRAM=blastp&BLAST_PROGRAMS=blastp&QUERY=MGRPAGWMKELTGRAPMKSPEKPSRRRDVERLFWGEIAKGLCIEDAAIAVGVSQAAGSRWFRERGGMSTFVIVPLAGRYLSFEEQEEIALLRIQGAGVRE IARFLGRAASTISRELRRNAATRAGRLDYRASVAQWKAELMAPRPKTAKLVVNERLRD&LINK_LOC=protein&PAGE_TYPE=BlastSearch', 'BLAST this protein','SACE_4954')" />

 
 
   CDAIRAVAKGERVIDPQLVLAALDDKPQPLTPRELEVLLLASQGEGSAQIAARLYLSVGTVRNYLTSVVTKLNARNRVDAIRIAREAGWL', 'http://blast.ncbi.nlm.nih.gov/Blast.cgi?PAGE=Proteins&PROGRAM=blastp&BLAST_PROGRAMS=blastp&QUERY=MLRKALVALLEFEPDIEVVAEFSSGAEILPRAKELRPDVAVLDIDLPGVDGLTAAGELHTAVPECRTMMLTSLGRPGNLRRALAAHVSGFLLKDSTPDKL CDAIRAVAKGERVIDPQLVLAALDDKPQPLTPRELEVLLLASQGEGSAQIAARLYLSVGTVRNYLTSVVTKLNARNRVDAIRIAREAGWL&LINK_LOC=protein&PAGE_TYPE=BlastSearch', 'BLAST this protein','SBI_06838')" />
   FLLKDAPPDRLAGAVRDVAAGKRVIDPQLALAAFDNGENPLTPRETEVLRMAADGAEAADIASRLFLSVGTVRNYLTTVVTKLNARNRVDAIRVARESGW L', 'http://blast.ncbi.nlm.nih.gov/Blast.cgi?PAGE=Proteins&PROGRAM=blastp&BLAST_PROGRAMS=blastp&QUERY=MIRILLAEDMHMVRGALVALLTLEDDLTVVAEVERGDRILPTALECVPDVAVIDVDLPGLDGLSAAAQLAQQLPSCRTLILTSLGRPGTLRKALAAQVGG FLLKDAPPDRLAGAVRDVAAGKRVIDPQLALAAFDNGENPLTPRETEVLRMAADGAEAADIASRLFLSVGTVRNYLTTVVTKLNARNRVDAIRVARESGW L&LINK_LOC=protein&PAGE_TYPE=BlastSearch', 'BLAST this protein','SCAB_63121')" />
   FIMKDAPPGELTEAIRTVAAGRRYIDGQLALAAWDSGECPLTERELEVLRIAASGSGVEEIAATLFLTTGTVRNYLASAVSKLNARNRIDAIRIAHASDW L', 'http://blast.ncbi.nlm.nih.gov/Blast.cgi?PAGE=Proteins&PROGRAM=blastp&BLAST_PROGRAMS=blastp&QUERY=MLRVMLAEDMHIVRAAIVALLDLEEDIRVVADVASGDEIMRVARAYRPDVAIIDIDLPVVDGITAARMIHQELPETRTLILTSLGRPGTLRKALTAKVGG FIMKDAPPGELTEAIRTVAAGRRYIDGQLALAAWDSGECPLTERELEVLRIAASGSGVEEIAATLFLTTGTVRNYLASAVSKLNARNRIDAIRIAHASDW L&LINK_LOC=protein&PAGE_TYPE=BlastSearch', 'BLAST this protein','Tcur_2770')" />
   FLPKDAPPTRLADAIRRVHRGERVIDSEIVVAAFHHGPNPLTERERDVLTHVAKGSDVSDVAAKLYLAPGTVRNYLSAIMSKIGARNRVDAIRIARDRGW ISA', 'http://blast.ncbi.nlm.nih.gov/Blast.cgi?PAGE=Proteins&PROGRAM=blastp&BLAST_PROGRAMS=blastp&QUERY=MIRILLAEDMHMVRGALVALLSLEPDLEVVAEVAEGTAILPNALTHRPDVAVLDIGLPGIDGLAAAGALSQQLPTCRTIMLTSHADSANIARAMLIGVSG FLPKDAPPTRLADAIRRVHRGERVIDSEIVVAAFHHGPNPLTERERDVLTHVAKGSDVSDVAAKLYLAPGTVRNYLSAIMSKIGARNRVDAIRIARDRGW ISA&LINK_LOC=protein&PAGE_TYPE=BlastSearch', 'BLAST this protein','VAB18032_20025')" />

 
 
   ESESDCLRDRRAEAAAFAARSGGAFSGDDVIDRVVAPIIYRVIFLPWTLSEIDAHAYVDEL', 'http://blast.ncbi.nlm.nih.gov/Blast.cgi?PAGE=Proteins&PROGRAM=blastp&BLAST_PROGRAMS=blastp&QUERY=MLASIRKAVEELIAEGGSDAITIPMVAERAGVNHSSIYRRWGDARTMINDLATYRLDPGRGLPDTGDVRVDLAAWARELVSHYSIPVNAAILRGGAAAAG ESESDCLRDRRAEAAAFAARSGGAFSGDDVIDRVVAPIIYRVIFLPWTLSEIDAHAYVDEL&LINK_LOC=protein&PAGE_TYPE=BlastSearch', 'BLAST this protein','SBI_09316')" />

 
 
   
   ASCPNQRFVLVGYSQGANVVDNSIGISSAGAVVGSPIVATLPAALEPRVSAVLLFGNPIRAIGKSVTGTYQSRTIDFCAAGDPVCENGGGDVGAHLGYRA NADAAAAFAATKI', 'http://blast.ncbi.nlm.nih.gov/Blast.cgi?PAGE=Proteins&PROGRAM=blastp&BLAST_PROGRAMS=blastp&QUERY=MRIRLYLAAIPLVGGAGLAAVATPTATAAACTDIDVVSARGTFEPGTLGFIVGDPVYAALQKKVAGKSLSSYKVNYPADLSPTSAAQGNADLVNHVRSQA ASCPNQRFVLVGYSQGANVVDNSIGISSAGAVVGSPIVATLPAALEPRVSAVLLFGNPIRAIGKSVTGTYQSRTIDFCAAGDPVCENGGGDVGAHLGYRA NADAAAAFAATKI&LINK_LOC=protein&PAGE_TYPE=BlastSearch', 'BLAST this protein','SCAB_78931')" />

 
 
   EFAWTAVLALAKDLPRLRDQQHEQRWQLPDARLVAGSRLLLLGLGRIGSAVAELAAAFGVRVTAITRTARPSPLAGTVLPPHVLRAAAADTDHLVNTLPS NPGTRGLVDRQVIEALPDDAVVVNVGRADTVDTAALVDRLRAGRLRGAVLDVHDEEPLPPNSPLWSVPRLFVTPHGAYRFPEEEHAVAKVFLAEFAAWSA AVAPADQGAAGCPYLARTEIA', 'http://blast.ncbi.nlm.nih.gov/Blast.cgi?PAGE=Proteins&PROGRAM=blastp&BLAST_PROGRAMS=blastp&QUERY=MNPRVCVYHPSMGPAITRALQAAHPGVRVDVASDLRTDPPQPETIDVLVANRLPDGLLSRCTRLSWLHLTGTGTDHLAAAGAPPGLRVTTSATVPVRAVA EFAWTAVLALAKDLPRLRDQQHEQRWQLPDARLVAGSRLLLLGLGRIGSAVAELAAAFGVRVTAITRTARPSPLAGTVLPPHVLRAAAADTDHLVNTLPS NPGTRGLVDRQVIEALPDDAVVVNVGRADTVDTAALVDRLRAGRLRGAVLDVHDEEPLPPNSPLWSVPRLFVTPHGAYRFPEEEHAVAKVFLAEFAAWSA AVAPADQGAAGCPYLARTEIA&LINK_LOC=protein&PAGE_TYPE=BlastSearch', 'BLAST this protein','VAB18032_22240')" />

 
 
   GEGKTLTCVLPAYLNALAGKGTHVVTVNDYLAKRDSEWMGRVHRFLGLDVGVILSQMTPLERRDAYNADITYGTNNEFGFDYLRDNMTHSLDELVQRGHA FAIVDEVDSILIDEARTPLIISGPADGASNWYTEFARIVPLMEKDTHYEVDIRKRTIGVHELGVEFVEDQLGIDNLYEAANSPLVSYLNNAIKAKELFTR DKDYIVREGEVLIVDEFTGRVLMGRRYNEGMHQAIEAKERVEIKAENQTLATITLQNYFRLYDKLAGMTGTAQTEAAELNEIYKLGVVSIPTNRPMVRKD QSDLIYKTEEAKYIAVVDDVVERYEKGQPVLIGTTSVERSEYLSRQFTKRRVPHNVLNAKYHEQEAAIIAEAGRRGAITVATNMAGRGTDIVLGGNPDFL ADKHLREQGLDPVETPDEYQAAWDETLQKFKDAAETEAKEVQEAGGLYVLGTERHESRRIDNQLRGRSGRQGDPGESRFYLSLQDELMRRFNGAALETIL TRMNVPDDVPIEAKMVTNAIKSAQTQVEQQNFEVRKNVLKYDEVMNQQRKVIYAERRRILDGEDLQPQIQEMITDTIAAYVDGATADGYHEDWDFDALWT ALKTLYPVSLKPEELIASGEYGEADELSPEDLKAALLEDAKKAYKAREAEIDGLAGEGSMRQLERNILLSVIDRKWREHLYEMDYLKEGIGLRAMAQRDP LVEYQREGYDMFTAMLDGLKEESVGFLFNINVEVQQPDGAAVAPQEAPEGLAEFASAAAQAAHGELRAKGLEESTPELTYSGPAEDGSAQSRHENGAASA SGGGTRRERREAARNAGRTSKPAKSRRKR', 'http://blast.ncbi.nlm.nih.gov/Blast.cgi?PAGE=Proteins&PROGRAM=blastp&BLAST_PROGRAMS=blastp&QUERY=MLSKLLRLGEGRMVKRLKGVADYVNTLSDDIEKLSDAELQAKTGEFKGRLEKGETLDDLMPEAFAVAREASWRVLSQRHFDVQVMGGAALHAGNIAEMKT GEGKTLTCVLPAYLNALAGKGTHVVTVNDYLAKRDSEWMGRVHRFLGLDVGVILSQMTPLERRDAYNADITYGTNNEFGFDYLRDNMTHSLDELVQRGHA FAIVDEVDSILIDEARTPLIISGPADGASNWYTEFARIVPLMEKDTHYEVDIRKRTIGVHELGVEFVEDQLGIDNLYEAANSPLVSYLNNAIKAKELFTR DKDYIVREGEVLIVDEFTGRVLMGRRYNEGMHQAIEAKERVEIKAENQTLATITLQNYFRLYDKLAGMTGTAQTEAAELNEIYKLGVVSIPTNRPMVRKD QSDLIYKTEEAKYIAVVDDVVERYEKGQPVLIGTTSVERSEYLSRQFTKRRVPHNVLNAKYHEQEAAIIAEAGRRGAITVATNMAGRGTDIVLGGNPDFL ADKHLREQGLDPVETPDEYQAAWDETLQKFKDAAETEAKEVQEAGGLYVLGTERHESRRIDNQLRGRSGRQGDPGESRFYLSLQDELMRRFNGAALETIL TRMNVPDDVPIEAKMVTNAIKSAQTQVEQQNFEVRKNVLKYDEVMNQQRKVIYAERRRILDGEDLQPQIQEMITDTIAAYVDGATADGYHEDWDFDALWT ALKTLYPVSLKPEELIASGEYGEADELSPEDLKAALLEDAKKAYKAREAEIDGLAGEGSMRQLERNILLSVIDRKWREHLYEMDYLKEGIGLRAMAQRDP LVEYQREGYDMFTAMLDGLKEESVGFLFNINVEVQQPDGAAVAPQEAPEGLAEFASAAAQAAHGELRAKGLEESTPELTYSGPAEDGSAQSRHENGAASA SGGGTRRERREAARNAGRTSKPAKSRRKR&LINK_LOC=protein&PAGE_TYPE=BlastSearch', 'BLAST this protein','MAB_3580c')" />

 
 
   
   

 
 
   DTFLWMEPPDHTRLRGLVTKGFTARRVAGLRPRIEELVDELIDAALDAGEFDFIETVAYPLPLTMICEILGVPTEDHPLVQKWSQALSRAFDPDLHMTPE ALAARNAALPEFAGYFRQLVDERRRHPGDDLISSLAAVEDHGDRLTADELLGTCITLIIAGHETTVNLVGNGALALLRHPDQLDLLRQRPELIPRAVDEL LRYDSPIHMNTRAAKRELVVGGRTFAPGEGVVALIACANRDPAAYDEPDRLDVTRFHGDRPVSRHLSFSLGHHYCLGAPLALLEMEIFLAAWVDRVGTAE ILTDRPTYKPNILIRGLADLPVRFRAPER', 'http://blast.ncbi.nlm.nih.gov/Blast.cgi?PAGE=Proteins&PROGRAM=blastp&BLAST_PROGRAMS=blastp&QUERY=MDVATTQAATAQGGRGSQATTPVSPVTPSLLDELFDPANRADPYSRYDHLRDAAPLHMSDFGLHVATRYVDCVKILQSADWGHDKEAEQLHPTIPASAFP DTFLWMEPPDHTRLRGLVTKGFTARRVAGLRPRIEELVDELIDAALDAGEFDFIETVAYPLPLTMICEILGVPTEDHPLVQKWSQALSRAFDPDLHMTPE ALAARNAALPEFAGYFRQLVDERRRHPGDDLISSLAAVEDHGDRLTADELLGTCITLIIAGHETTVNLVGNGALALLRHPDQLDLLRQRPELIPRAVDEL LRYDSPIHMNTRAAKRELVVGGRTFAPGEGVVALIACANRDPAAYDEPDRLDVTRFHGDRPVSRHLSFSLGHHYCLGAPLALLEMEIFLAAWVDRVGTAE ILTDRPTYKPNILIRGLADLPVRFRAPER&LINK_LOC=protein&PAGE_TYPE=BlastSearch', 'BLAST this protein','FsymDg_0408')" />
   MAPFGPPHTPRRLHAMRGELTRIVGELIDGFEGRDRIDLVDHFSYPFPVTVICRLLGVPREDEPRFHVWAETLAASLDPDPDADAAERRRITRQARTELA MYLSELMEERRRAPRDDMLSDLVNGQGPEGRMSRVELLSTATLLLVAGHETTVNLITNGMLTLLRNPDVLARLRKAPHLVVPLVEELLRFDPPVQMLPQR TPLSEIDIAGVTIPKGASVWLLLASGNRDPQRFLDPDRFDPQRRDNQHLGFGSGIHSCFGAPLARLEAQIALTELARRLDRPRLLEDPPTYRRNAVLRGP RHLPIALEGLRPKASPNGSSNGSNGSR', 'http://blast.ncbi.nlm.nih.gov/Blast.cgi?PAGE=Proteins&PROGRAM=blastp&BLAST_PROGRAMS=blastp&QUERY=MTQTQGSLARQITDYAHRADPYPIYAELRKTPVVHDEEGPYIVSTYWEIHGLLHDPRLSSEARNLDPEAAPELAEEDDPELPPSFLRLDPPEHDRLRRLA MAPFGPPHTPRRLHAMRGELTRIVGELIDGFEGRDRIDLVDHFSYPFPVTVICRLLGVPREDEPRFHVWAETLAASLDPDPDADAAERRRITRQARTELA MYLSELMEERRRAPRDDMLSDLVNGQGPEGRMSRVELLSTATLLLVAGHETTVNLITNGMLTLLRNPDVLARLRKAPHLVVPLVEELLRFDPPVQMLPQR TPLSEIDIAGVTIPKGASVWLLLASGNRDPQRFLDPDRFDPQRRDNQHLGFGSGIHSCFGAPLARLEAQIALTELARRLDRPRLLEDPPTYRRNAVLRGP RHLPIALEGLRPKASPNGSSNGSNGSR&LINK_LOC=protein&PAGE_TYPE=BlastSearch', 'BLAST this protein','Strvi_9074')" />

 
 
   GIAEPNFEAIGKAKPDLIVVDGTSINNNPPVIEALRAIAPTAFVGYAGGDWRYTFKTLAKVVNKQDEAKQVLADYDAKVSTVKKGLGKYQGETFSIVRWQ GSSAALILNELPPGRALLDLGLKRPKNQDKDGRGHSEPVSRENLAEIDADWMFFGTLGGSSVDNRNAEGGVDLEAAKKALSEAEDAPGFTSLKAYKDDQI ILVDGSLWTSTGGPLLMNKIVADVDRALVEEQR', 'http://blast.ncbi.nlm.nih.gov/Blast.cgi?PAGE=Proteins&PROGRAM=blastp&BLAST_PROGRAMS=blastp&QUERY=MSLRSSAVALSLALLVLLGGCAWSSAQAPAASGETRVVKDAEETEITVPVEPQRIVALSEPTLDGLLALGITPIGTVSGRGQSKVPGYLSAEAGEIPLLG GIAEPNFEAIGKAKPDLIVVDGTSINNNPPVIEALRAIAPTAFVGYAGGDWRYTFKTLAKVVNKQDEAKQVLADYDAKVSTVKKGLGKYQGETFSIVRWQ GSSAALILNELPPGRALLDLGLKRPKNQDKDGRGHSEPVSRENLAEIDADWMFFGTLGGSSVDNRNAEGGVDLEAAKKALSEAEDAPGFTSLKAYKDDQI ILVDGSLWTSTGGPLLMNKIVADVDRALVEEQR&LINK_LOC=protein&PAGE_TYPE=BlastSearch', 'BLAST this protein','MLP_27420')" />
   QGLDKPPAYLADKVEGIDVVGNLLQPVMDKVVAAKPDLILAGDMQDEQVLKQLREITPATLVTMAPTDDWKLFFRGVGNAVNKLDDANEFISGHEASAKA AGDKLGANKGAEVSIVRWNPDGPSWMENKQFASGVALEMGLKRPASQNKDGNAHTPSLSLEKINEIDGDWLFLSTLTSDGEKALKDVQSKPAYKELGAVK DGHAVTVDGSVWSTRGGPLAADVVLADYVKALSAE', 'http://blast.ncbi.nlm.nih.gov/Blast.cgi?PAGE=Proteins&PROGRAM=blastp&BLAST_PROGRAMS=blastp&QUERY=MILHHRPTRTAARRAARAVATLGAAALLLTACGTDSDSGKSSGDKAGKADSASSATRTVKDATGKAVEIPAEPKRIVTLTQEDLDAVLALDIKPVGITNG QGLDKPPAYLADKVEGIDVVGNLLQPVMDKVVAAKPDLILAGDMQDEQVLKQLREITPATLVTMAPTDDWKLFFRGVGNAVNKLDDANEFISGHEASAKA AGDKLGANKGAEVSIVRWNPDGPSWMENKQFASGVALEMGLKRPASQNKDGNAHTPSLSLEKINEIDGDWLFLSTLTSDGEKALKDVQSKPAYKELGAVK DGHAVTVDGSVWSTRGGPLAADVVLADYVKALSAE&LINK_LOC=protein&PAGE_TYPE=BlastSearch', 'BLAST this protein','SGR_6741')" />

 
 
   EEWGLLDALHPGRLDLGLGRAGWHPPAPVAGDAEATGDAPAGPAGPVEPDEPPAPYPRLRGSDLFALHRLLLPLNPLNSPDYLDQVTEVVALLDGTRRAG DLRVHAVPGHQAEVAVWVLGRTAGESAEVAGRLGLPYATNYHASPSTTADSVAAYRKAFRASETLARPYVIVTADVVVGPDDESARRAAVGHDQWSLANR VGEPTVFPSPEEASAFPWEPADRELVADLGRSRLVGTAEHVATGLRELRDRFDADELLVTTTTFAQADRLRSYELLAEQWRLPRAD', 'http://blast.ncbi.nlm.nih.gov/Blast.cgi?PAGE=Proteins&PROGRAM=blastp&BLAST_PROGRAMS=blastp&QUERY=MTGTAPQPRPASDVPLGVLDLAPISAGESVADALRNSLGLARHTDRLGYHRYWVTEHHVNPSTAGLSSTLLTALVADATTRIRVGSGSLQLGHRTALSVA EEWGLLDALHPGRLDLGLGRAGWHPPAPVAGDAEATGDAPAGPAGPVEPDEPPAPYPRLRGSDLFALHRLLLPLNPLNSPDYLDQVTEVVALLDGTRRAG DLRVHAVPGHQAEVAVWVLGRTAGESAEVAGRLGLPYATNYHASPSTTADSVAAYRKAFRASETLARPYVIVTADVVVGPDDESARRAAVGHDQWSLANR VGEPTVFPSPEEASAFPWEPADRELVADLGRSRLVGTAEHVATGLRELRDRFDADELLVTTTTFAQADRLRSYELLAEQWRLPRAD&LINK_LOC=protein&PAGE_TYPE=BlastSearch', 'BLAST this protein','Franean1_3465')" />
   IDLGVGRSAQRRGVKPRGPAKSLAPQPAREWREIDGVVIPPPFDLRTLLSGGRVQATMAILQQPEAVAPDFAEQVGDILALLAGTYRAEGFDVHAVPGEN AALTPWIFGSSKGRSAQVAGALGLPFVASYHLTPATALEAIETYRDAFVPSAALQRPYVVVSADIVVAQDSATARRLASSYGHWVYSIRAGGGAAPYPDP DDCAALTDDQLAVVSDRLATQFVGNPDEVAERMLALQRVAGADELVVTSVTHGHQDRLRSHELIARRWGLWS', 'http://blast.ncbi.nlm.nih.gov/Blast.cgi?PAGE=Proteins&PROGRAM=blastp&BLAST_PROGRAMS=blastp&QUERY=MIPLSILDLSPISAGSTAATALRNTIELAQHAERWGYRRFWVAEHHFVAVASASPAVLIGQIAAATDRIRVGSAAVQLSHTTAAAVVESFGMLDAFHPGR IDLGVGRSAQRRGVKPRGPAKSLAPQPAREWREIDGVVIPPPFDLRTLLSGGRVQATMAILQQPEAVAPDFAEQVGDILALLAGTYRAEGFDVHAVPGEN AALTPWIFGSSKGRSAQVAGALGLPFVASYHLTPATALEAIETYRDAFVPSAALQRPYVVVSADIVVAQDSATARRLASSYGHWVYSIRAGGGAAPYPDP DDCAALTDDQLAVVSDRLATQFVGNPDEVAERMLALQRVAGADELVVTSVTHGHQDRLRSHELIARRWGLWS&LINK_LOC=protein&PAGE_TYPE=BlastSearch', 'BLAST this protein','MMAR_0702')" />
   RLDLGIGRSGHRRAQFGAEAGAPKGAHPVDDAATGRTTVRDGVVVPPPFDPRGLGDRRRYLAGLAALHLPGAEPLDFAEQVAEIRALLDGTYVTADGVPL HAVPGEGARVQLWVFGSSGGESAELAGRLGLPFAAAYHTAPGTALEAVAAYRAAFRPSVQLAEPYVVVSADVVVAEDDAAARHLASTYGHWVHSIRAGGG AAEILDPDTAAPLTPDQRRLVEDRLATQFVGSPATVVERLGALQRATGANELLVTSVTYDHAARLASHRLLAEAWGLLPAARDGHARTGAAVTP', 'http://blast.ncbi.nlm.nih.gov/Blast.cgi?PAGE=Proteins&PROGRAM=blastp&BLAST_PROGRAMS=blastp&QUERY=MSVPLSVLDLAPVSAGSSPRQALRNTVELARKAEEWGYHRYWLAEHHFVAVASASPITLIALVAAATSRIRVGSGAVQLGHHTSASIVEGFGTVDALHPG RLDLGIGRSGHRRAQFGAEAGAPKGAHPVDDAATGRTTVRDGVVVPPPFDPRGLGDRRRYLAGLAALHLPGAEPLDFAEQVAEIRALLDGTYVTADGVPL HAVPGEGARVQLWVFGSSGGESAELAGRLGLPFAAAYHTAPGTALEAVAAYRAAFRPSVQLAEPYVVVSADVVVAEDDAAARHLASTYGHWVHSIRAGGG AAEILDPDTAAPLTPDQRRLVEDRLATQFVGSPATVVERLGALQRATGANELLVTSVTYDHAARLASHRLLAEAWGLLPAARDGHARTGAAVTP&LINK_LOC=protein&PAGE_TYPE=BlastSearch', 'BLAST this protein','NFA_17410')" />
   RLDLGLGRSGQRRAEALKAVAERNNLPAPPRRETEIRDGVVIPAPFDPSGLLLSPRLTAAQSVLQQPGALSPAFDQQVDDILALLGGTYSTPDGIELHAS PGEGADVEVWVFGSSAGPSAELAGRLGLPFGANYHVSPSTTIEAVEAYRAAFRPSPNLQKPYVVVSADAVVAEDDATAKELASTFAHWTHSIRSGHGAIP YPDPAHTAPLGEAERAAVEDRLITQFVGAPATVADRLDSLRRLTDADELVITSVTHDHGDRLRSYELLAAEWGLPGLLAA', 'http://blast.ncbi.nlm.nih.gov/Blast.cgi?PAGE=Proteins&PROGRAM=blastp&BLAST_PROGRAMS=blastp&QUERY=MTTPLSVLDLSPVSEGTTARTALRNTVDLARHAEAWGYKRYWVAEHHFVGVASSSPAVLVGILAAATSTLRVGAAAVQLGHTTAAAVVEAFGTVDALYPG RLDLGLGRSGQRRAEALKAVAERNNLPAPPRRETEIRDGVVIPAPFDPSGLLLSPRLTAAQSVLQQPGALSPAFDQQVDDILALLGGTYSTPDGIELHAS PGEGADVEVWVFGSSAGPSAELAGRLGLPFGANYHVSPSTTIEAVEAYRAAFRPSPNLQKPYVVVSADAVVAEDDATAKELASTFAHWTHSIRSGHGAIP YPDPAHTAPLGEAERAAVEDRLITQFVGAPATVADRLDSLRRLTDADELVITSVTHDHGDRLRSYELLAAEWGLPGLLAA&LINK_LOC=protein&PAGE_TYPE=BlastSearch', 'BLAST this protein','RHA1_ro02216')" />
   RLDLGLGRSGQRRAEALKAVAERTKQPAPPRRETEIRDGVVIPAPFDPSGLLLSPRLTAAQSVLQQPGALSPAFDQQVDDILALLGGTYRTPDGIELHAS PGERADVDVWVFGSSAGPSAELAGRLGLPFGANYHVSPSTTIEAVEAYRAAFRPSAYLQRPYVVVSADTVVAEDDATAQELASTFGHWTHSIRSGHGAIP YPDPAHTAALGDTERALVEDRLITQFVGAPATVADRLDSLRRLTDADELVITSVTHDHTDRLRSYELLAAEWGLPGLLAA', 'http://blast.ncbi.nlm.nih.gov/Blast.cgi?PAGE=Proteins&PROGRAM=blastp&BLAST_PROGRAMS=blastp&QUERY=MTTPLSVLDLSPVSEGATARTALRNTVDLAQHAERWGYKRYWVAEHHFVGVASSSPAVLVGILAAATSTLRVGAAAVQLGHTTAAAVVEAFGTVDALYPG RLDLGLGRSGQRRAEALKAVAERTKQPAPPRRETEIRDGVVIPAPFDPSGLLLSPRLTAAQSVLQQPGALSPAFDQQVDDILALLGGTYRTPDGIELHAS PGERADVDVWVFGSSAGPSAELAGRLGLPFGANYHVSPSTTIEAVEAYRAAFRPSAYLQRPYVVVSADTVVAEDDATAQELASTFGHWTHSIRSGHGAIP YPDPAHTAALGDTERALVEDRLITQFVGAPATVADRLDSLRRLTDADELVITSVTHDHTDRLRSYELLAAEWGLPGLLAA&LINK_LOC=protein&PAGE_TYPE=BlastSearch', 'BLAST this protein','ROP_19300')" />

 
 
   PREVYQAINTTGTAHVLELARERSTPLVHVSTAYVCGERDGRILESQLDVGQRFGNPYEESKLAAEQLVRKAAAESLPTAVIRPSVVVGAARTGAVRDFK NLYVVLKLLSEGRIGSIPGYFDACVDLVPVDHVAALITAVADDFDRASDRTLHAVGSSVRMRHISDVLAEYPSFHVPRYIAPANFDPAMLSDLERAYWHR VMSLYESYFRRQQHFDDTVAAGFRGPKRLPSGPNHLRRIIDYAVRAGYLGAPLPGVTEALSRVNQR', 'http://blast.ncbi.nlm.nih.gov/Blast.cgi?PAGE=Proteins&PROGRAM=blastp&BLAST_PROGRAMS=blastp&QUERY=MSKPKRILVTGAAGLVGAEVCARLLKAGHRVSGLVHHTRVLIANSGRGVASSTDGRAGTVRLVTGDVTVPRLGLDDDTWTDLANGLDLIVHSAAITDFGH PREVYQAINTTGTAHVLELARERSTPLVHVSTAYVCGERDGRILESQLDVGQRFGNPYEESKLAAEQLVRKAAAESLPTAVIRPSVVVGAARTGAVRDFK NLYVVLKLLSEGRIGSIPGYFDACVDLVPVDHVAALITAVADDFDRASDRTLHAVGSSVRMRHISDVLAEYPSFHVPRYIAPANFDPAMLSDLERAYWHR VMSLYESYFRRQQHFDDTVAAGFRGPKRLPSGPNHLRRIIDYAVRAGYLGAPLPGVTEALSRVNQR&LINK_LOC=protein&PAGE_TYPE=BlastSearch', 'BLAST this protein','Sare_3013')" />
   TVALDGPEQDYQALNVTATANTVELAQRWDVPLVHVSTAYVCGRRGGRILEDELDVGQEFSNGYEHSKLRAEKLVRSAPGLRWSVVRPAIVTGSADGGAV RDYKNLYTLVKLIVEGKLRRLPGRYDATLSLVPVDYTADVTVSATLRMQSDEAAIRGRTFHATGNGVISLREVSNVFAEYPSFHVATFVPAASFSIEDLD SFERDYYQRLGAQYVCYFDRVRAFDDSATRTLLGMQPPDTGQEYLRTLLDYCLEAGYLGRALPSVEEILQDESSSSEGGARTADRAGTNELVR', 'http://blast.ncbi.nlm.nih.gov/Blast.cgi?PAGE=Proteins&PROGRAM=blastp&BLAST_PROGRAMS=blastp&QUERY=MNPAQKTRGRAILVTGAAGLVGAEVVDRLSKHLPVIATTHRNPRIVRNDGCGVDAVAYTPGHDAPVRQLAVDVRLPDFGLDADLLESLSVEVGGIVHCAA TVALDGPEQDYQALNVTATANTVELAQRWDVPLVHVSTAYVCGRRGGRILEDELDVGQEFSNGYEHSKLRAEKLVRSAPGLRWSVVRPAIVTGSADGGAV RDYKNLYTLVKLIVEGKLRRLPGRYDATLSLVPVDYTADVTVSATLRMQSDEAAIRGRTFHATGNGVISLREVSNVFAEYPSFHVATFVPAASFSIEDLD SFERDYYQRLGAQYVCYFDRVRAFDDSATRTLLGMQPPDTGQEYLRTLLDYCLEAGYLGRALPSVEEILQDESSSSEGGARTADRAGTNELVR&LINK_LOC=protein&PAGE_TYPE=BlastSearch', 'BLAST this protein','Srot_1339')" />
   PREVYQAINTTGTAHVLELARERSTPLVHVSTAYVCGERDGMILESQLDVGQRFGNPYEESKLAAEQLVRKAAAESLPTVVIRPSVVVGAARTGAVRDFK NLYVVLKLLSEGRIGSIPGYFDACVDLVPVDHVAALITAVVDDFDRARDRTLHAVGSSVRMRHISDVLAEYPSFHVPRYIAPANFDPAMLSDLERAYWHR VMSLYESYFRRQQHFDDTVAAGFRGHKRLPSGPNHLRRIIDYAVRADYLGAPLPGVAEALSRVNQR', 'http://blast.ncbi.nlm.nih.gov/Blast.cgi?PAGE=Proteins&PROGRAM=blastp&BLAST_PROGRAMS=blastp&QUERY=MSTPKRILVTGAAGLVGAEVCARLLKAGHRVSGLVHHTRVLIANNGRAVASATDDRAGTVRLVTGDVTVPRLGLDDDTWTDLANGLDLIVHSAAITDFGH PREVYQAINTTGTAHVLELARERSTPLVHVSTAYVCGERDGMILESQLDVGQRFGNPYEESKLAAEQLVRKAAAESLPTVVIRPSVVVGAARTGAVRDFK NLYVVLKLLSEGRIGSIPGYFDACVDLVPVDHVAALITAVVDDFDRARDRTLHAVGSSVRMRHISDVLAEYPSFHVPRYIAPANFDPAMLSDLERAYWHR VMSLYESYFRRQQHFDDTVAAGFRGHKRLPSGPNHLRRIIDYAVRADYLGAPLPGVAEALSRVNQR&LINK_LOC=protein&PAGE_TYPE=BlastSearch', 'BLAST this protein','Strop_4147')" />

 
 
   STVDGGIVVDLSRFSGIDVRPDGRAAIGAGARLGPIAATLAAHGRVLPAGSCETVGIAGLTLGGGVGLVDRKHGLTCDHLEAARIVTADGRVRTVSRAAE PDLFWALRGGGGGNFGIVTGFTFRTVPSADVATFKLTFPPGTQAALLAAWQEWLPGTPDELWSGVNIDAGTAITNGTFLGREARLKELLDDLVRRVGTPP AEREARVTDHLAAMRSFDDHEGRPGAVAARAAYVGTSRMLLRPVTDPAAVVEVLTRAPRVGTLIDSAGGAIARVGARETAFPHRSALASFQFLHGATPED GGEAEARRALGAVRDGLGPEFGATGYVNYLDPEMPDWARAYYGVNLARLRAVARKYDPDGLFAFPQGLSGPRPIVHKGGS', 'http://blast.ncbi.nlm.nih.gov/Blast.cgi?PAGE=Proteins&PROGRAM=blastp&BLAST_PROGRAMS=blastp&QUERY=MDRRAFLRISGAVLAASLAGWPPPDDWQRLRERLSGPLFRPGDPGYPEAKQGFFTMYDDRVPVAVVGAARVEDVQAAIGFAARHRLPVAARSGGHSYPGY STVDGGIVVDLSRFSGIDVRPDGRAAIGAGARLGPIAATLAAHGRVLPAGSCETVGIAGLTLGGGVGLVDRKHGLTCDHLEAARIVTADGRVRTVSRAAE PDLFWALRGGGGGNFGIVTGFTFRTVPSADVATFKLTFPPGTQAALLAAWQEWLPGTPDELWSGVNIDAGTAITNGTFLGREARLKELLDDLVRRVGTPP AEREARVTDHLAAMRSFDDHEGRPGAVAARAAYVGTSRMLLRPVTDPAAVVEVLTRAPRVGTLIDSAGGAIARVGARETAFPHRSALASFQFLHGATPED GGEAEARRALGAVRDGLGPEFGATGYVNYLDPEMPDWARAYYGVNLARLRAVARKYDPDGLFAFPQGLSGPRPIVHKGGS&LINK_LOC=protein&PAGE_TYPE=BlastSearch', 'BLAST this protein','AMED_5316')" />
   PRGGGHSYIGASTAVGAMVIDLRGLPGGVNFDAGSGTVTVPAGVSLTDVHQVLAGAGRAIPTGSCPTVGVGGLALGGGMGADSRHAGLTCDALRSATVVL PSGETVTASAEDHPDLFWALRGGGGGNFGVTTAMTFETFPVADSDVVRVDFTPSSAAQVLAGWQTWLAAADRDTWGLVDMSVSAAQANCHVLATCPAGAG RGVADAIKSAVGAQPTGIETKTFNHMELVKYLAGGSSASSPRGFVAGSDVIGTVNSAAAQAIVAAVGKWPPASGRASALVDTLTGAVGDIDPTATAFPWR RQSAVVQWYVETPSSGQTAAATKWINSAHQAVQQFSVGGYVNYLEANTAPSRYFGSNLSRLTQIRQRYDPSRLMYSGLNF', 'http://blast.ncbi.nlm.nih.gov/Blast.cgi?PAGE=Proteins&PROGRAM=blastp&BLAST_PROGRAMS=blastp&QUERY=MISRQTLLRGAAAATAAGLGSALFGSARAVAEPVSGWSALASSIGGRVLLPSNGASFTSGKQVFNSLYNNSNPAAVVTVTSQADVEKAVAFAAANKLKIA PRGGGHSYIGASTAVGAMVIDLRGLPGGVNFDAGSGTVTVPAGVSLTDVHQVLAGAGRAIPTGSCPTVGVGGLALGGGMGADSRHAGLTCDALRSATVVL PSGETVTASAEDHPDLFWALRGGGGGNFGVTTAMTFETFPVADSDVVRVDFTPSSAAQVLAGWQTWLAAADRDTWGLVDMSVSAAQANCHVLATCPAGAG RGVADAIKSAVGAQPTGIETKTFNHMELVKYLAGGSSASSPRGFVAGSDVIGTVNSAAAQAIVAAVGKWPPASGRASALVDTLTGAVGDIDPTATAFPWR RQSAVVQWYVETPSSGQTAAATKWINSAHQAVQQFSVGGYVNYLEANTAPSRYFGSNLSRLTQIRQRYDPSRLMYSGLNF&LINK_LOC=protein&PAGE_TYPE=BlastSearch', 'BLAST this protein','MMAR_1179')" />
   IVVDLSRFSGIDVRPDGRAAIGAGARLGPIAATLAAHGRVLPAGSCETVGIAGLTLGGGVGLVDRKHGLTCDHLEAARIVTADGRVRTVSRAAEPDLFWA LRGGGGGNFGIVTGFTFRTVPSADVATFKLTFPPGTQAALLAAWQEWLPGTPDELWSGVNIDAGTAITNGTFLGREARLKELLDDLVRRVGTPPAEREAR VTDHLAAMRSFDDHEGRPGAVAARAAYVGTSRMLLRPVTDPAAVVEVLTRAPRVGTLIDSAGGAIARVGARETAFPHRSALASFQFLHGATPEDGGEAEA RRALGAVRDGLGPEFGATGYVNYLDPEMPDWARAYYGVNLARLRAVARKYDPDGLFAFPQGLSGPRPIVHKGGS', 'http://blast.ncbi.nlm.nih.gov/Blast.cgi?PAGE=Proteins&PROGRAM=blastp&BLAST_PROGRAMS=blastp&QUERY=MRISGAVLAASLAGWPPPDDWQRLRERLSGPLFRPGDPGYPEAKQGFFTMYDDRVPVAVVGAARVEDVQAAIGFAARHRLPVAARSGGHSYPGYSTVDGG IVVDLSRFSGIDVRPDGRAAIGAGARLGPIAATLAAHGRVLPAGSCETVGIAGLTLGGGVGLVDRKHGLTCDHLEAARIVTADGRVRTVSRAAEPDLFWA LRGGGGGNFGIVTGFTFRTVPSADVATFKLTFPPGTQAALLAAWQEWLPGTPDELWSGVNIDAGTAITNGTFLGREARLKELLDDLVRRVGTPPAEREAR VTDHLAAMRSFDDHEGRPGAVAARAAYVGTSRMLLRPVTDPAAVVEVLTRAPRVGTLIDSAGGAIARVGARETAFPHRSALASFQFLHGATPEDGGEAEA RRALGAVRDGLGPEFGATGYVNYLDPEMPDWARAYYGVNLARLRAVARKYDPDGLFAFPQGLSGPRPIVHKGGS&LINK_LOC=protein&PAGE_TYPE=BlastSearch', 'BLAST this protein','RAM_27080')" />
   GATVTAGPGTRIGPLAEVLARHGRVVPVGWCPMVAVAGASMGGGFGPLGRYYGLGCDHLVGAEVVLADGRIVRTSETTEPDLLWALRGAGAGNFGAVTSL TFRTRPAVPAVHFAAWWKPEDGAAVIDAWQRWAPTAPSRVNAELILRCWPDPDEPATLSVFGLIVGASPRAAAERVAELADLVGISPERVTYTELTAEEL PNHHTFAGEPTSHNKLGGRPGDAEPGVRFVKSEFFDAAVPLDAIADLVDGLLRDRVASQQREFEFIPWGGAIGEPAPGDTAFVHRSPRFLVEHSVQAYGS AELKRASHEWVTASKATLHRWGNGHVYQNYPEPDLPDWDIAYYGDNLHRLHAVKAAYDPDGVFRYEQSLRADGF', 'http://blast.ncbi.nlm.nih.gov/Blast.cgi?PAGE=Proteins&PROGRAM=blastp&BLAST_PROGRAMS=blastp&QUERY=MKTVSFGQLGDRLHGRLILPGEPDFDSVRKWFIGRFTETVPQAVARCADTRDVAEAVAFARAKDIPFALRSGAHSFAEYSMSEGLVIDLDGMDEVRVSPD GATVTAGPGTRIGPLAEVLARHGRVVPVGWCPMVAVAGASMGGGFGPLGRYYGLGCDHLVGAEVVLADGRIVRTSETTEPDLLWALRGAGAGNFGAVTSL TFRTRPAVPAVHFAAWWKPEDGAAVIDAWQRWAPTAPSRVNAELILRCWPDPDEPATLSVFGLIVGASPRAAAERVAELADLVGISPERVTYTELTAEEL PNHHTFAGEPTSHNKLGGRPGDAEPGVRFVKSEFFDAAVPLDAIADLVDGLLRDRVASQQREFEFIPWGGAIGEPAPGDTAFVHRSPRFLVEHSVQAYGS AELKRASHEWVTASKATLHRWGNGHVYQNYPEPDLPDWDIAYYGDNLHRLHAVKAAYDPDGVFRYEQSLRADGF&LINK_LOC=protein&PAGE_TYPE=BlastSearch', 'BLAST this protein','Snas_3488')" />

 
 
   VARTAGDPAEMRAWAAQDRMAVVNAMSADEHPTQALTDLTTLQGHFGGIEGLRVLYVGEGNNTASALALALTRYPGVSLELRTPPGYGLAPYYRERAADQ AGRHGATFRERHDMDDLPADQDAVYTSRWQTTGTSKPDPDWREIFAPFQVSAALWETSPKAVFLHDLPAHRGDEVTAEVLDGPASIAFAQAENKMHSAMA VLEWCRP', 'http://blast.ncbi.nlm.nih.gov/Blast.cgi?PAGE=Proteins&PROGRAM=blastp&BLAST_PROGRAMS=blastp&QUERY=MAPVRHLISIDDLSDTDLYSLVLRGAAFSAGTAGRPAPLAGDVVGIYFRKTSTRTRTAFSSGALRLGAQIIAYGPDDLQLNTGETSEDTGRVMSGMLDVL VARTAGDPAEMRAWAAQDRMAVVNAMSADEHPTQALTDLTTLQGHFGGIEGLRVLYVGEGNNTASALALALTRYPGVSLELRTPPGYGLAPYYRERAADQ AGRHGATFRERHDMDDLPADQDAVYTSRWQTTGTSKPDPDWREIFAPFQVSAALWETSPKAVFLHDLPAHRGDEVTAEVLDGPASIAFAQAENKMHSAMA VLEWCRP&LINK_LOC=protein&PAGE_TYPE=BlastSearch', 'BLAST this protein','SAV_3641')" />
   SHRFGSLRGLHLVYLGEGNNTAAALAHGLALIPGAALTLATPAGYGLDPAVLVRAQQTAKEWGSKVVEVHRVDDIDGDADVVYTTQWQTTGTSKSDPDWR TAFTPFAVDERLMARWPDAFFMHDLPARRGEEVTSGVLDGPRSLAWDQAQMKLASAMAVLDWCLT', 'http://blast.ncbi.nlm.nih.gov/Blast.cgi?PAGE=Proteins&PROGRAM=blastp&BLAST_PROGRAMS=blastp&QUERY=MVGTLFSHTSTRTRTAFTSGAIRLGGTVIAYGPNDLQLATGEPLSDTGRILGGMLDALVVRDSVGENDLRELAGTASIPVINAMVREEHPTQGLTDIALL SHRFGSLRGLHLVYLGEGNNTAAALAHGLALIPGAALTLATPAGYGLDPAVLVRAQQTAKEWGSKVVEVHRVDDIDGDADVVYTTQWQTTGTSKSDPDWR TAFTPFAVDERLMARWPDAFFMHDLPARRGEEVTSGVLDGPRSLAWDQAQMKLASAMAVLDWCLT&LINK_LOC=protein&PAGE_TYPE=BlastSearch', 'BLAST this protein','SBI_06059')" />
   VLVARTAGPEHELRAYAAQRRMAVVNAMSQGEHPTQALADLTTLLRRFGRIEDLHVIYVGEGNNTASALALALSRFPGTRLTLRTPPGYGVAPEYLERAA VSAKRSGARIEERHDMADLPAADVVYTTRWQTTGTVKPTADWREVFAPFQVTEKTMASSPDAVFMHDLPAHRGEEVTADVLDGPASIAFDQAENKYHSAR AVLEWCAADRSSER', 'http://blast.ncbi.nlm.nih.gov/Blast.cgi?PAGE=Proteins&PROGRAM=blastp&BLAST_PROGRAMS=blastp&QUERY=MPQAERRHLISIDDLSDEELRHIALRGAEFSAGSADDARPLADTVVGVLFRKTSTRTRTAFSAGALRLGARLITYGPGDLQENTGETVEDSAAVLSRMID VLVARTAGPEHELRAYAAQRRMAVVNAMSQGEHPTQALADLTTLLRRFGRIEDLHVIYVGEGNNTASALALALSRFPGTRLTLRTPPGYGVAPEYLERAA VSAKRSGARIEERHDMADLPAADVVYTTRWQTTGTVKPTADWREVFAPFQVTEKTMASSPDAVFMHDLPAHRGEEVTADVLDGPASIAFDQAENKYHSAR AVLEWCAADRSSER&LINK_LOC=protein&PAGE_TYPE=BlastSearch', 'BLAST this protein','Sfla_0093')" />

 
 
   ASQASGRVEEVRSAPIDTALAAALDTDDPQRVAEIVALLDELARHLTPQALTRLRG', 'http://blast.ncbi.nlm.nih.gov/Blast.cgi?PAGE=Proteins&PROGRAM=blastp&BLAST_PROGRAMS=blastp&QUERY=MMYLGSMNGVELFLLGRTLMKIGEEALPTEGIGRHSTSVRTVLIVVSDVRAHPGSAVGEIATRTGLPQSAVSAAVARLREAGAITTEPDSRDRRRLLIQS ASQASGRVEEVRSAPIDTALAAALDTDDPQRVAEIVALLDELARHLTPQALTRLRG&LINK_LOC=protein&PAGE_TYPE=BlastSearch', 'BLAST this protein','Sros_0079')" />

 
 
   CRYGETSLCTAGYDETGFTRPGAFADHLVVPARLLHPLADDADLRAAALLEPAAVVAAAVRAGTPEPGERIAVLGAGTLGLLAVQLLAAVSPAELTVIDP REARAAQALDFGAGEARTPAESEEVRGHYDLVVETAGAPSTAADACLLARRGGRVVLTGMFTPGAVGIDPVHLSLSQLTVRSVFGAPSAAWSYAVRAFTA GLLDPAPLITHEFPLDRFADAVALVGSGDPETGKVLLRP', 'http://blast.ncbi.nlm.nih.gov/Blast.cgi?PAGE=Proteins&PROGRAM=blastp&BLAST_PROGRAMS=blastp&QUERY=MTSTSRSLTVDRPGSHRLDEGPPPEPGPGEVRVRVAAAGICMSDREVYDGHRDPAYVRYPVVPGHEWSGTVDALGEGVDPALLGRRTVAEGFRACGRCER CRYGETSLCTAGYDETGFTRPGAFADHLVVPARLLHPLADDADLRAAALLEPAAVVAAAVRAGTPEPGERIAVLGAGTLGLLAVQLLAAVSPAELTVIDP REARAAQALDFGAGEARTPAESEEVRGHYDLVVETAGAPSTAADACLLARRGGRVVLTGMFTPGAVGIDPVHLSLSQLTVRSVFGAPSAAWSYAVRAFTA GLLDPAPLITHEFPLDRFADAVALVGSGDPETGKVLLRP&LINK_LOC=protein&PAGE_TYPE=BlastSearch', 'BLAST this protein','Sfla_4421')" />

 
 
   RRVCFRVGEQWPARFVLFAEGRTVKRVGVVVDHVAIDPWGMGVLHNDLRQALKARATGHEPFGAELVEQPIDVAESEASPSGLAYQGRARDYWQERLDLI ADTLAGRTPGTRPAAKPGAPVFRSARLYSPRAAQAAQSIAAATGVSAASTFLLAFGTAVCAAEQSAGVGLFAISANRTTLQSARSVRKATMTLPVLIRAE QGPIRPALADCAAQQLRGHRFANADPRVTERMCHEILGDHYETAVAYPRFSYLAAPFGTENGTPGRITFGEPRSLGARFMLTVAQHRRGARLDLEWSEES GWGALAADLLMRTENLVIQGAASL', 'http://blast.ncbi.nlm.nih.gov/Blast.cgi?PAGE=Proteins&PROGRAM=blastp&BLAST_PROGRAMS=blastp&QUERY=MTGAESGRSGGLTLSQHEWVQWIPADSDTSFEDNIWTVVPADGVPLDRAAQAVRDVLLRHEGLRSLVARTAGTQLVEAVDERIGEAVAIADAAAPSDDGW RRVCFRVGEQWPARFVLFAEGRTVKRVGVVVDHVAIDPWGMGVLHNDLRQALKARATGHEPFGAELVEQPIDVAESEASPSGLAYQGRARDYWQERLDLI ADTLAGRTPGTRPAAKPGAPVFRSARLYSPRAAQAAQSIAAATGVSAASTFLLAFGTAVCAAEQSAGVGLFAISANRTTLQSARSVRKATMTLPVLIRAE QGPIRPALADCAAQQLRGHRFANADPRVTERMCHEILGDHYETAVAYPRFSYLAAPFGTENGTPGRITFGEPRSLGARFMLTVAQHRRGARLDLEWSEES GWGALAADLLMRTENLVIQGAASL&LINK_LOC=protein&PAGE_TYPE=BlastSearch', 'BLAST this protein','Caci_5072')" />

 
 
   IDVEVDNLRKVRDGLADPSTVAELGARLAGCEPLPVLGLRVSSGLATTFAYFARRIHPDVRLLTHGGSELADGLHAARRAGAGWLVAVILPRYPAEAVQA LRSARELGLRTAVITDRPDVPFEADVILDAPVGERLVFDSHAAPLALAMVLVEAMADAAPLRTQARLEEYERMIDQAGVFVDRPNAPA', 'http://blast.ncbi.nlm.nih.gov/Blast.cgi?PAGE=Proteins&PROGRAM=blastp&BLAST_PROGRAMS=blastp&QUERY=MPVDDQAVGGDVGDALDRLLEGRRLSPVQRRIARYLDDNLAEAIFLSSVELADRAGVSQPSVTRFAMVLGFAGYPELRQALRPFVVGDRSPSRANDLQTA IDVEVDNLRKVRDGLADPSTVAELGARLAGCEPLPVLGLRVSSGLATTFAYFARRIHPDVRLLTHGGSELADGLHAARRAGAGWLVAVILPRYPAEAVQA LRSARELGLRTAVITDRPDVPFEADVILDAPVGERLVFDSHAAPLALAMVLVEAMADAAPLRTQARLEEYERMIDQAGVFVDRPNAPA&LINK_LOC=protein&PAGE_TYPE=BlastSearch', 'BLAST this protein','Sros_1774')" />

 
 
   LDDAGMVPQKLNVPREQVAYVDVTLTSDRLENLTVVDTPGMSSVNTSISNEANKFLFDAPIADDIDPDSQSALSGAEAIIYTFTQSVREDDLQALEAFRT MSSRLSSNPINSLGLFNKVDKLAPTPSGDPWPVAEPLSRSQSQLMRRVVSDVVPVVGLLAETTEAGRLTAADCEALRKLAELPQEERTVLLAAASLFSTR ECPVSPEQRERLLRLLDLYGINFAIAHLVANPSMATGELVRLLFAASGFPRLRQTLEQAFRLRSDAIKAGWGLARLETLASTTASPQEREMLRDAIERVI QDPQYHRLRLIEVAAQVTTGTVELPSDMESEIARLALSSEPSHVLGLPANDHEALVKAALAANNRWRAFAVGGASPAQARVAHVVARGFHLLAQQLRSPR TAH', 'http://blast.ncbi.nlm.nih.gov/Blast.cgi?PAGE=Proteins&PROGRAM=blastp&BLAST_PROGRAMS=blastp&QUERY=MASGPLSTRVAALCAELSPRLSPRSRAEVDGVRARIGQPLRVAIAGRLKAGKSTLVNALIGRRVAPTEVGECTRLVTQFRYGTSDRVDVVKRDGTRVSLP LDDAGMVPQKLNVPREQVAYVDVTLTSDRLENLTVVDTPGMSSVNTSISNEANKFLFDAPIADDIDPDSQSALSGAEAIIYTFTQSVREDDLQALEAFRT MSSRLSSNPINSLGLFNKVDKLAPTPSGDPWPVAEPLSRSQSQLMRRVVSDVVPVVGLLAETTEAGRLTAADCEALRKLAELPQEERTVLLAAASLFSTR ECPVSPEQRERLLRLLDLYGINFAIAHLVANPSMATGELVRLLFAASGFPRLRQTLEQAFRLRSDAIKAGWGLARLETLASTTASPQEREMLRDAIERVI QDPQYHRLRLIEVAAQVTTGTVELPSDMESEIARLALSSEPSHVLGLPANDHEALVKAALAANNRWRAFAVGGASPAQARVAHVVARGFHLLAQQLRSPR TAH&LINK_LOC=protein&PAGE_TYPE=BlastSearch', 'BLAST this protein','Snas_3727')" />

 
 
   DRRTWDRILGINLTGAFLGIKAAAPSLRRNGGGVIVNIASTSGVGGTAMYAPYVASKWAIRGLTKTAALELGRDHIRVNAIHPGVIATPFITEPAAGSDA PISDFYSAEPFAIPRLGEPADITRALLFLTSDEASFATGSEFVIDGGLLLGPALRHEAV', 'http://blast.ncbi.nlm.nih.gov/Blast.cgi?PAGE=Proteins&PROGRAM=blastp&BLAST_PROGRAMS=blastp&QUERY=MGRFDHRSVLVTGGTGGQGASHIRAFHAEGADVVIADIDENRGHALADELGTRALYVRLDVTDENSWNAAVQASEEHFGPLSVLVNNAGVQNPAAPIETT DRRTWDRILGINLTGAFLGIKAAAPSLRRNGGGVIVNIASTSGVGGTAMYAPYVASKWAIRGLTKTAALELGRDHIRVNAIHPGVIATPFITEPAAGSDA PISDFYSAEPFAIPRLGEPADITRALLFLTSDEASFATGSEFVIDGGLLLGPALRHEAV&LINK_LOC=protein&PAGE_TYPE=BlastSearch', 'BLAST this protein','SGR_48t')" />
   DRRTWDRILGINLTGAFLGIKAAAPSLRRNGGGVIVNIASTSGVGGTAMYAPYVASKWAIRGLTKTAALELGRDHIRVNAIHPGVIATPFITEPAAGSDA PISDFYSAEPFAIPRLGEPADITRALLFLTSDEASFATGSEFVIDGGLLLGPALRHEAV', 'http://blast.ncbi.nlm.nih.gov/Blast.cgi?PAGE=Proteins&PROGRAM=blastp&BLAST_PROGRAMS=blastp&QUERY=MGRFDHRSVLVTGGTGGQGASHIRAFHAEGADVVIADIDENRGHALADELGTRALYVRLDVTDENSWNAAVQASEEHFGPLSVLVNNAGVQNPAAPIETT DRRTWDRILGINLTGAFLGIKAAAPSLRRNGGGVIVNIASTSGVGGTAMYAPYVASKWAIRGLTKTAALELGRDHIRVNAIHPGVIATPFITEPAAGSDA PISDFYSAEPFAIPRLGEPADITRALLFLTSDEASFATGSEFVIDGGLLLGPALRHEAV&LINK_LOC=protein&PAGE_TYPE=BlastSearch', 'BLAST this protein','SGR_7091t')" />
   HGVESLGEEEWSTVVDVCQRGTWLGMRALAPLMRLAGGGSIVNLSSVFALIGTGAAVAYHAAKGAIVSMTRTAALEFAPQRIRVNAVSPGIVRTPLTDGL PAGFVDRIAADTPLGRPGAPAEIAAAVHFLLSDAASYITGTNLVVDGGLTAR', 'http://blast.ncbi.nlm.nih.gov/Blast.cgi?PAGE=Proteins&PROGRAM=blastp&BLAST_PROGRAMS=blastp&QUERY=MGRPGLSGRVAVVTGAAGGIGAAVARTLVADGAGVVLADVDVAGAEKVARRLREEGHPGRALAARLDVTSPADWQAVCRTACRAFGRPQLLVNNAGVHGL HGVESLGEEEWSTVVDVCQRGTWLGMRALAPLMRLAGGGSIVNLSSVFALIGTGAAVAYHAAKGAIVSMTRTAALEFAPQRIRVNAVSPGIVRTPLTDGL PAGFVDRIAADTPLGRPGAPAEIAAAVHFLLSDAASYITGTNLVVDGGLTAR&LINK_LOC=protein&PAGE_TYPE=BlastSearch', 'BLAST this protein','Tcur_0214')" />

 
 
   TLVGLTGENAVLGARRLRFAGEDLAAFTEARWRAVRGRRIGLVLQDALVSLDPLRTVGAEIAEVLRTHRVVPRAEIDARMLKLLDDVGVPEPARRARQYP HELSGGLRQRALIASAIAAGPALVLADEPTTALDVTVQAQVLDLLRQHKIDGAALLLISHDLAVVGGLADDIAVMYAGHIVEHGPAEQILGDPRHPYTRA LLDAVPVTHTKGTRLAPAASRGPLPGPAGCPYADRCPRADDRCRERLPARAVPAGAHHRHDVLCWHPFEALSVEVLSVEVLAAEVLAAEVLAAEVPPARA LSAGAPSFGAPSPGPATDTTATAEDWANTSGTEGAAATSGTAGAAGVVPTAALGAAVASPRPAAPGDATGVGIPPRPRVGAAATGEALLEVEGVSRRFRS PDGTWRDAVRDVSLRLHAGETLGVVGESGSGKTTVARIVLGQLEPDTGTVHFAGQPWSGLRERARRARRRRIQAIYQDPLGSFDPRYRVERIIGEAVAIG GVPGDADPARASAPRGAARRARVVELLDQVGLPADVLRRRPLELSGGQRQRVAIARALAPGPDLIVCDEPVSALDVSIQAQILDLLAGLQHDLGVALLFI SHDLGVIHHASDRIIVMKDGQVVETGDVTTVFARPAHAYTRQLLAAVPRPVGGRAAPPGPADAGPAPGAAPAAGSLSVVSR', 'http://blast.ncbi.nlm.nih.gov/Blast.cgi?PAGE=Proteins&PROGRAM=blastp&BLAST_PROGRAMS=blastp&QUERY=MTGPAGNISPAGSTSLAGKEGKEGKKRKTDKAGRVGRVGRVGRVDSALAESLLVVDGLDVTFTTGGAAVHAVRDVSFTLDAGRCLALVGESGSGKSVTAR TLVGLTGENAVLGARRLRFAGEDLAAFTEARWRAVRGRRIGLVLQDALVSLDPLRTVGAEIAEVLRTHRVVPRAEIDARMLKLLDDVGVPEPARRARQYP HELSGGLRQRALIASAIAAGPALVLADEPTTALDVTVQAQVLDLLRQHKIDGAALLLISHDLAVVGGLADDIAVMYAGHIVEHGPAEQILGDPRHPYTRA LLDAVPVTHTKGTRLAPAASRGPLPGPAGCPYADRCPRADDRCRERLPARAVPAGAHHRHDVLCWHPFEALSVEVLSVEVLAAEVLAAEVLAAEVPPARA LSAGAPSFGAPSPGPATDTTATAEDWANTSGTEGAAATSGTAGAAGVVPTAALGAAVASPRPAAPGDATGVGIPPRPRVGAAATGEALLEVEGVSRRFRS PDGTWRDAVRDVSLRLHAGETLGVVGESGSGKTTVARIVLGQLEPDTGTVHFAGQPWSGLRERARRARRRRIQAIYQDPLGSFDPRYRVERIIGEAVAIG GVPGDADPARASAPRGAARRARVVELLDQVGLPADVLRRRPLELSGGQRQRVAIARALAPGPDLIVCDEPVSALDVSIQAQILDLLAGLQHDLGVALLFI SHDLGVIHHASDRIIVMKDGQVVETGDVTTVFARPAHAYTRQLLAAVPRPVGGRAAPPGPADAGPAPGAAPAAGSLSVVSR&LINK_LOC=protein&PAGE_TYPE=BlastSearch', 'BLAST this protein','FsymDg_2622')" />
   EMRPVRANHIAMVFQDALSALNPVWTVGFQLGELFRIHRGMSRSDARKRAIELLDLVKIPAAKQRVDEYPHQFSGGMRQRVMIAMALALDPEILIADEPT TALDVTVQAQIMRLLAEIQAERQMGLILITHDLGVVADVADDVTVMYAGRAVEQATIHEIFEKPAHPYTKALLKSIPRLDVKGQRLEVIAGLPPVLTNIP PGCSFAPRCGYAQDVCRTDDPELLQFTGERFAACHFSSEVMNDE', 'http://blast.ncbi.nlm.nih.gov/Blast.cgi?PAGE=Proteins&PROGRAM=blastp&BLAST_PROGRAMS=blastp&QUERY=MTTVEIAKKDVLAGLDPSAPLLRVKNLQVEFNTREGKAKAVNGVNFTLEEGQTLAIVGESGSGKSVTSQAIMGILDIPPARIADGEVLFRGVDLLKLKAK EMRPVRANHIAMVFQDALSALNPVWTVGFQLGELFRIHRGMSRSDARKRAIELLDLVKIPAAKQRVDEYPHQFSGGMRQRVMIAMALALDPEILIADEPT TALDVTVQAQIMRLLAEIQAERQMGLILITHDLGVVADVADDVTVMYAGRAVEQATIHEIFEKPAHPYTKALLKSIPRLDVKGQRLEVIAGLPPVLTNIP PGCSFAPRCGYAQDVCRTDDPELLQFTGERFAACHFSSEVMNDE&LINK_LOC=protein&PAGE_TYPE=BlastSearch', 'BLAST this protein','Snas_4141')" />

 
 
   VDVFQTGSGTSSNMNTNEVIATLATERLGREVHPNDHVNASQSSNDVFPSSIHIAATAAVTADLIPALVHLAESLGRKSAEFAEVVKSGRTHLMDATPVT LGQEFGGYAAQIRYGVERLRASLPRLAELPLGGTAVGTGINTPPGFSAAVIAEVAEATGLPLTEARDHFEAQGARDGLVETSGQLRTIAVSLTKISNDLR WMASGPRTGLAEIALPDLQPGSSIMPGKVNPVIPEAVLMVAAQVTGNDATVATAGAAGNFELNVMLPVIAKNLLESVRLLANASRLLADRTVDGITADVE RARAYAESSPSVVTPLNKYIGYEEAAKVAKKSLKDGTTIRETVLASGYVERGDLTMEQLDEALDVLRMTRP', 'http://blast.ncbi.nlm.nih.gov/Blast.cgi?PAGE=Proteins&PROGRAM=blastp&BLAST_PROGRAMS=blastp&QUERY=MVDTSGEQPRDSGFRIEHDSMGEVRVPADAKWRAQTQRAVENFPVSGQRLERAHIEALARIKGAAAKVNAELKVLDPDIAAAIQEAAAEVASGRWDAHFP VDVFQTGSGTSSNMNTNEVIATLATERLGREVHPNDHVNASQSSNDVFPSSIHIAATAAVTADLIPALVHLAESLGRKSAEFAEVVKSGRTHLMDATPVT LGQEFGGYAAQIRYGVERLRASLPRLAELPLGGTAVGTGINTPPGFSAAVIAEVAEATGLPLTEARDHFEAQGARDGLVETSGQLRTIAVSLTKISNDLR WMASGPRTGLAEIALPDLQPGSSIMPGKVNPVIPEAVLMVAAQVTGNDATVATAGAAGNFELNVMLPVIAKNLLESVRLLANASRLLADRTVDGITADVE RARAYAESSPSVVTPLNKYIGYEEAAKVAKKSLKDGTTIRETVLASGYVERGDLTMEQLDEALDVLRMTRP&LINK_LOC=protein&PAGE_TYPE=BlastSearch', 'BLAST this protein','SGR_2491')" />

 
 
   VHIGVLDGTDVFYVAKVDSSHPVRMVSAVGKRLPAHCTAVGKVLLAALPRQRLDELYASVRLTAMTDNSITNRRALRRELDTIAADGGVAREYCESNDAV ACVAAPVRDQSGEVVAALSISAPILRWNEETEPALRELACEGARLLSERLGHRPAG', 'http://blast.ncbi.nlm.nih.gov/Blast.cgi?PAGE=Proteins&PROGRAM=blastp&BLAST_PROGRAMS=blastp&QUERY=MARAVPAVERAFDVLELFLDEHELSAPEITAKLGLPRTTVHELVGTLAERGYLTPAGGGSNRFRLGVRGFQLGSAYAERLDLAREGRVVAETVAERCSET VHIGVLDGTDVFYVAKVDSSHPVRMVSAVGKRLPAHCTAVGKVLLAALPRQRLDELYASVRLTAMTDNSITNRRALRRELDTIAADGGVAREYCESNDAV ACVAAPVRDQSGEVVAALSISAPILRWNEETEPALRELACEGARLLSERLGHRPAG&LINK_LOC=protein&PAGE_TYPE=BlastSearch', 'BLAST this protein','SACE_3534')" />

 
 
   HWRERLSAVAYANWRLLLRHPWMLQTPRGRLLGPHLIAKYEYELSAVDGLGLSDVEMDSTVSLVNSYAEGAASQAAAVSETERATGMTDGQWWSTHGPLL AAFTRDSDYPLASRVGSTVGREHRTVYSSDHEFAFGLERVLDGVRVLVESRSARGAEGS', 'http://blast.ncbi.nlm.nih.gov/Blast.cgi?PAGE=Proteins&PROGRAM=blastp&BLAST_PROGRAMS=blastp&QUERY=MPTQYTKSGDPARSLALLWRTQEPVSRRNKPDLSVDRIVAVASEMADNEGLAALSMRKVAERLGVGTMSLYTYVPGKGELVDLMLDAAYADMYADGERPE HWRERLSAVAYANWRLLLRHPWMLQTPRGRLLGPHLIAKYEYELSAVDGLGLSDVEMDSTVSLVNSYAEGAASQAAAVSETERATGMTDGQWWSTHGPLL AAFTRDSDYPLASRVGSTVGREHRTVYSSDHEFAFGLERVLDGVRVLVESRSARGAEGS&LINK_LOC=protein&PAGE_TYPE=BlastSearch', 'BLAST this protein','Ndas_4687')" />

 
 
   IPGRAEFVRVDVTRADSRFDGAFDLVIADRLVNRFVRAELRSALRTLSAAVRPGGKMRLSYRLGLYERDEAVLSEAARRGVLSTVFDEAEFDVDYSAAAE WLGTVLPPHGDIPTHALVDFYVARGREHRIRTGELDELAAQDVPRGLRYETAHLPVPGQGDDFLLQLTRLA', 'http://blast.ncbi.nlm.nih.gov/Blast.cgi?PAGE=Proteins&PROGRAM=blastp&BLAST_PROGRAMS=blastp&QUERY=MPPPTGVAAHGRTSVIADHEITNSKAGAWDRLGSAYWNRNYDGGPNPAACAQYLEGLAAGERVLLVGASTVALARAVLDAGAELVVADFSAVMLAELENL IPGRAEFVRVDVTRADSRFDGAFDLVIADRLVNRFVRAELRSALRTLSAAVRPGGKMRLSYRLGLYERDEAVLSEAARRGVLSTVFDEAEFDVDYSAAAE WLGTVLPPHGDIPTHALVDFYVARGREHRIRTGELDELAAQDVPRGLRYETAHLPVPGQGDDFLLQLTRLA&LINK_LOC=protein&PAGE_TYPE=BlastSearch', 'BLAST this protein','FRAAL_6373')" />

 
 
   PPVRDLDRLAHWARTLRIPPRLLWFDLPGQPRSTPAAVMVASAEPSRPVPVAGGTLDMIRSAALAFRAADRQLGSGRLYPVVVRFLQTEVAPHLVGDQYP PSAIFSAAASLTDMAGWLAYDDNRGDLAAQHFVQAFGLATAAGDQALSAQTLVSQSQLALENDRPDDAVRLAMAGLALTPDDPQCGALRSRLHVMTARGN ALAGSSAASLAALRDAEQELARAIPTGDEWLSPFDEAALAAEAAICLRDLNDWTAAEREALRVLELRTPDRVRSRAFTNLTLATVHLGRGDLDATCEAGN RVLDAATRLDSDRVVDHLRTLGRRLEPHLGLPVVDDLLGRVATALPAGQLA', 'http://blast.ncbi.nlm.nih.gov/Blast.cgi?PAGE=Proteins&PROGRAM=blastp&BLAST_PROGRAMS=blastp&QUERY=MDTASSRYCRSCGNRLARDNTNPVCARCDRQSRLAPDTAPAVPEQFWHTDHLRDAFTAQHIGRVSVAYRLNPHHPRIISQERLARWLSMTQAQVSRIETG PPVRDLDRLAHWARTLRIPPRLLWFDLPGQPRSTPAAVMVASAEPSRPVPVAGGTLDMIRSAALAFRAADRQLGSGRLYPVVVRFLQTEVAPHLVGDQYP PSAIFSAAASLTDMAGWLAYDDNRGDLAAQHFVQAFGLATAAGDQALSAQTLVSQSQLALENDRPDDAVRLAMAGLALTPDDPQCGALRSRLHVMTARGN ALAGSSAASLAALRDAEQELARAIPTGDEWLSPFDEAALAAEAAICLRDLNDWTAAEREALRVLELRTPDRVRSRAFTNLTLATVHLGRGDLDATCEAGN RVLDAATRLDSDRVVDHLRTLGRRLEPHLGLPVVDDLLGRVATALPAGQLA&LINK_LOC=protein&PAGE_TYPE=BlastSearch', 'BLAST this protein','FraEuI1c_0875')" />

 
 
   

 
 
   PRRARIIDNDH', 'http://blast.ncbi.nlm.nih.gov/Blast.cgi?PAGE=Proteins&PROGRAM=blastp&BLAST_PROGRAMS=blastp&QUERY=MKREVEYKWRLSELMAARGLHNTTDLIPLLAERGITLSRPQVYRLVNQKPERVALQVIAAICDIFSCGPEDLITVTAADVRARKTGTSAPNVVDLNRTVR PRRARIIDNDH&LINK_LOC=protein&PAGE_TYPE=BlastSearch', 'BLAST this protein','Arth_2201')" />
   YRPVRARITRPHEQ', 'http://blast.ncbi.nlm.nih.gov/Blast.cgi?PAGE=Proteins&PROGRAM=blastp&BLAST_PROGRAMS=blastp&QUERY=MNRQIDYQFRVKELMARAGMRNSRDLVAPLRERGITLSESQIYRLVGQNPDRISFQVLAALCDIFKVEANEILTYTATDARTQRRRTAVGDGTDVPLLAA YRPVRARITRPHEQ&LINK_LOC=protein&PAGE_TYPE=BlastSearch', 'BLAST this protein','Asphe3_19230')" />

 
 
   SALRPRLLGIADTLLDGVLDAGGPADLVDRFTAPLSAEATCELVGVPYADRAVFLDWFTAFAVGGDTGRSRELVTAYTDGLVAERRARPRADLVTALVRA EPELTRGELLEVVGGVLLAGDGVATQLANCAYVLLAHPEHARLLREEPELLPLAAQELLRRVPFPPGAACARYATEDVELGGALIRAGDAVVPASAPGAD ALDFRREAGPAPTRHHCLGPGVVAELLEVALTALLHRVPTVRLAAEEVLDWRRDLLVRRVVRLPVTW', 'http://blast.ncbi.nlm.nih.gov/Blast.cgi?PAGE=Proteins&PROGRAM=blastp&BLAST_PROGRAMS=blastp&QUERY=MPAAPTLPHPSGPPVRLEPPGERSGLTSVSVPFGAEAWLVTRREDISALQDDHRFTSAAESRPPLLPGRAVPPARERDDGDRARLRRVLADCQVPAGPDL SALRPRLLGIADTLLDGVLDAGGPADLVDRFTAPLSAEATCELVGVPYADRAVFLDWFTAFAVGGDTGRSRELVTAYTDGLVAERRARPRADLVTALVRA EPELTRGELLEVVGGVLLAGDGVATQLANCAYVLLAHPEHARLLREEPELLPLAAQELLRRVPFPPGAACARYATEDVELGGALIRAGDAVVPASAPGAD ALDFRREAGPAPTRHHCLGPGVVAELLEVALTALLHRVPTVRLAAEEVLDWRRDLLVRRVVRLPVTW&LINK_LOC=protein&PAGE_TYPE=BlastSearch', 'BLAST this protein','Amir_2491')" />

 
 
   IEVEIEVLGARLHTATAPKAGEHQVGPQTRPVARRTIA', 'http://blast.ncbi.nlm.nih.gov/Blast.cgi?PAGE=Proteins&PROGRAM=blastp&BLAST_PROGRAMS=blastp&QUERY=MDTGPIPNHPTSEPVRTVYGYISVEHTDEAVIERLRTRLTEHARAVGMSLTEIFIDRCVPPGRVVRPGLNVLLDTILRSGGDVLVIEVDHLSSLPAVRRA IEVEIEVLGARLHTATAPKAGEHQVGPQTRPVARRTIA&LINK_LOC=protein&PAGE_TYPE=BlastSearch', 'BLAST this protein','FraEuI1c_0876')" />

 
 
   AIHGLVRDKEWELLEHGEWFIRLAVEVGAAPGWPVPLHAEIVYDLAPRGLTVTHEVRNEGDRPIGFGVGAHPYLRIGDVPTDELTLTLAASRVRPYVAEE QLPFGEEIDVEGTDYDLRTGRIVGGLDLDTAFGGLTPAADGRHHHLLSHGDTTVDLWADADFRWVQVFTPSDYPGRGRAIAIEPMTCPADALNSGTDLIT LEPGASWRGSWGIQVDIR', 'http://blast.ncbi.nlm.nih.gov/Blast.cgi?PAGE=Proteins&PROGRAM=blastp&BLAST_PROGRAMS=blastp&QUERY=MAIGSVDRSAGIVRPMANPTGEQFELTRGNARAVVTEIGAGLRAFEINKVPYLETFEEDAEPPKAAGQVLLPWPNRTKGARWIFDGEPQELEVTEEARGN AIHGLVRDKEWELLEHGEWFIRLAVEVGAAPGWPVPLHAEIVYDLAPRGLTVTHEVRNEGDRPIGFGVGAHPYLRIGDVPTDELTLTLAASRVRPYVAEE QLPFGEEIDVEGTDYDLRTGRIVGGLDLDTAFGGLTPAADGRHHHLLSHGDTTVDLWADADFRWVQVFTPSDYPGRGRAIAIEPMTCPADALNSGTDLIT LEPGASWRGSWGIQVDIR&LINK_LOC=protein&PAGE_TYPE=BlastSearch', 'BLAST this protein','Svir_33650')" />

 
 
   GTITGRAQPGNPAPRLFRLPADRALINRMGFNNPGADVAAGHLAAARSGPVRIPIAANIGKTKVVPVEDAVEDYRYSARLLGPHADFVVVNVSSPNTPGL RDLQAVDALRPILAAVQAEVSVPVLVKIAPDLADDDIDAVADLAVELGLAGIVATNTTISRDGLTTPADEVAAMGAGGLSGAPLGDRSLTVLRRLYGRVG GRVTLVSAGGIETAEQAWERICAGADLLQGYTGFIYGGPLWVSQIHAGLAERVRAQGLRSIADAVGSDGNRPATD', 'http://blast.ncbi.nlm.nih.gov/Blast.cgi?PAGE=Proteins&PROGRAM=blastp&BLAST_PROGRAMS=blastp&QUERY=MLRRALHTVNGAVYPLLLKLMFLLSPERIHGIAFGAISLTGRVTPLRQMAAKVLAPHDPVLHQRVFGVDFPAPFGLAAGFDKSAHAVNAWGQLGFGFAEI GTITGRAQPGNPAPRLFRLPADRALINRMGFNNPGADVAAGHLAAARSGPVRIPIAANIGKTKVVPVEDAVEDYRYSARLLGPHADFVVVNVSSPNTPGL RDLQAVDALRPILAAVQAEVSVPVLVKIAPDLADDDIDAVADLAVELGLAGIVATNTTISRDGLTTPADEVAAMGAGGLSGAPLGDRSLTVLRRLYGRVG GRVTLVSAGGIETAEQAWERICAGADLLQGYTGFIYGGPLWVSQIHAGLAERVRAQGLRSIADAVGSDGNRPATD&LINK_LOC=protein&PAGE_TYPE=BlastSearch', 'BLAST this protein','Gbro_2435')" />
   NPAPRLFRLPDDRALLNRMGFNNQGAGALATRLARHHADVPIGVNIGKTKTTPAEDAVADYRASARLLAPLADYLVVNVSSPNTPGLRDLQAVEALRPIL AAVRAETTRPVLVKIAPDLADTDLDAVADLAVELGLAGIVATNTTVSRAGLATPGVADLGPGGISGPPVARRALEVLRRLYARVGDRLVLISVGGIETAD DAWERITAGASLLQGYTGFIYGGGLWARNIHHGLADRLRAGGFASLSEAVGSAAR', 'http://blast.ncbi.nlm.nih.gov/Blast.cgi?PAGE=Proteins&PROGRAM=blastp&BLAST_PROGRAMS=blastp&QUERY=MGLYGALRRALFLAPPERSHTFAFAALRGATALSPTRRVLRRRLAPHDPILASTVFGVRFPGPLGLAAGFDKDGVGLKAWGALGFGYAEVGTVTAAPQPG NPAPRLFRLPDDRALLNRMGFNNQGAGALATRLARHHADVPIGVNIGKTKTTPAEDAVADYRASARLLAPLADYLVVNVSSPNTPGLRDLQAVEALRPIL AAVRAETTRPVLVKIAPDLADTDLDAVADLAVELGLAGIVATNTTVSRAGLATPGVADLGPGGISGPPVARRALEVLRRLYARVGDRLVLISVGGIETAD DAWERITAGASLLQGYTGFIYGGGLWARNIHHGLADRLRAGGFASLSEAVGSAAR&LINK_LOC=protein&PAGE_TYPE=BlastSearch', 'BLAST this protein','JDM601_1756')" />

 
 
   TRRPADAEPVLSFDQERLWLESQLLPPTAYNVHGRRRLAGPVDVAALEAGVAAIVARHDALRARFPVIDGQPVQIVDPPDAQWRLETARTDSLAAALRLA DRQAETAFDLALGPLFRCLLVAVDGSDGSGGGTNGATDAEPEYVLSVTVHHIVADAWSIGLFVRELLALYAAGGDPERAGLPELTVQYPDFAVWQRAHIA GEELTGQLAYWRDHLAGAPPVLAMPVSRRLAVPGGGIERARAELTGAESAALGDLRRKHGVTTFMVLLAALGATLGRWSGRRDVVVGVPIAGRADAGTHA LIGFFVNTLPVRVDLRGDPSFGDLLARVRQAALDGYANADAPFDVLVKELQAPRDPRQTPLFQALLNVIGPPEAETVAGIAVEPLELPALPSKFDLALTA QERGGRLGFDLAFNADRYHGSMMRELLAQVVALVRDALEDPGRAVSELGGAPGAAADSSDASGSADGNATAAAWTPHLAVDRFAQQADRVAVVGADGEHG YRWLARAADRVAAFLGAREAEPGRVGIARHPTAAFVATVLGCLKAGLEFTVTEPAAGVPASFLGLSQLLDTEETEGLSTLFEDLKEPLPAAESEATTHER DDWAVARFGFSRDDRFAAPATSPGLLVSALSSALSAGATLVMTELTPAGGVAELGDWLRSQAVSVLYTAPPLIRALAAADLRLPTLRFALVDNAGDFLPH DVEAAALLSPDCRCVSLYRVGQDGRPVAVYAVPADFTVASAPLRVPLGTGAVGLPHPSGRPAAIGEIAEIRADGRRTGDLGRWRADGVLEYTGLAGADPG QDLAEAASALRDVAEVRDALVTEQVGDEGDAIVVAYLVGPDPDAGTSGIRRYLISRLPEWLIPGALVVVGALPLTAEGDHDVALLPRTDPGAATEVYVAP RTPMEQQLVDVMAALLAVDRIGVHDTFFELGGFSLLATRLTSRIRDLFDVELSLRDVFEAPTVEGLAQLILRAQSEAFGGEDLEGLLAEITAAD', 'http://blast.ncbi.nlm.nih.gov/Blast.cgi?PAGE=Proteins&PROGRAM=blastp&BLAST_PROGRAMS=blastp&QUERY=MAHVSQAHDQAGAAGAVPPRTPYEEAVAAIWRDILGRPDVGALDDFFSLDATSLQAIQVVSRIRKTLGVDIPVKDVFQEPTVAALAARVQAESASRRSAL TRRPADAEPVLSFDQERLWLESQLLPPTAYNVHGRRRLAGPVDVAALEAGVAAIVARHDALRARFPVIDGQPVQIVDPPDAQWRLETARTDSLAAALRLA DRQAETAFDLALGPLFRCLLVAVDGSDGSGGGTNGATDAEPEYVLSVTVHHIVADAWSIGLFVRELLALYAAGGDPERAGLPELTVQYPDFAVWQRAHIA GEELTGQLAYWRDHLAGAPPVLAMPVSRRLAVPGGGIERARAELTGAESAALGDLRRKHGVTTFMVLLAALGATLGRWSGRRDVVVGVPIAGRADAGTHA LIGFFVNTLPVRVDLRGDPSFGDLLARVRQAALDGYANADAPFDVLVKELQAPRDPRQTPLFQALLNVIGPPEAETVAGIAVEPLELPALPSKFDLALTA QERGGRLGFDLAFNADRYHGSMMRELLAQVVALVRDALEDPGRAVSELGGAPGAAADSSDASGSADGNATAAAWTPHLAVDRFAQQADRVAVVGADGEHG YRWLARAADRVAAFLGAREAEPGRVGIARHPTAAFVATVLGCLKAGLEFTVTEPAAGVPASFLGLSQLLDTEETEGLSTLFEDLKEPLPAAESEATTHER DDWAVARFGFSRDDRFAAPATSPGLLVSALSSALSAGATLVMTELTPAGGVAELGDWLRSQAVSVLYTAPPLIRALAAADLRLPTLRFALVDNAGDFLPH DVEAAALLSPDCRCVSLYRVGQDGRPVAVYAVPADFTVASAPLRVPLGTGAVGLPHPSGRPAAIGEIAEIRADGRRTGDLGRWRADGVLEYTGLAGADPG QDLAEAASALRDVAEVRDALVTEQVGDEGDAIVVAYLVGPDPDAGTSGIRRYLISRLPEWLIPGALVVVGALPLTAEGDHDVALLPRTDPGAATEVYVAP RTPMEQQLVDVMAALLAVDRIGVHDTFFELGGFSLLATRLTSRIRDLFDVELSLRDVFEAPTVEGLAQLILRAQSEAFGGEDLEGLLAEITAAD&LINK_LOC=protein&PAGE_TYPE=BlastSearch', 'BLAST this protein','Caci_3433')" />

 
 
   IVRGSFRMNVSVDGATAEVHDRIRGRRGSFDRAMSALALLDTAARKRRLSGGSPLGLGIDCVVVSSNFHQIEDFCTAIAPRFPELESIAFGAVIPEGLAS REGFAQHELLSDSQVELLGNPEYGEHLRSLAPPSVRVATTDNLALQMHPDRIRGGSFFPVLQIEPDGAARAMAAYEGTVGNVLTDSPEELWRRAVERWHD PFVVETLAPVRTMEQWAEATRKIDYHFGSEADRARIDRRPEFVPTGPAARVPRQDSAQRL', 'http://blast.ncbi.nlm.nih.gov/Blast.cgi?PAGE=Proteins&PROGRAM=blastp&BLAST_PROGRAMS=blastp&QUERY=MKIPDRPLEVVWDITYACPLRCSHCYSESGRRPARQLDHGQMLRAADAVIAMKPYGVCIAGGEPLLVKGVFEVIDRLSAAGMEVSLFTGGWSLRPEMIDD IVRGSFRMNVSVDGATAEVHDRIRGRRGSFDRAMSALALLDTAARKRRLSGGSPLGLGIDCVVVSSNFHQIEDFCTAIAPRFPELESIAFGAVIPEGLAS REGFAQHELLSDSQVELLGNPEYGEHLRSLAPPSVRVATTDNLALQMHPDRIRGGSFFPVLQIEPDGAARAMAAYEGTVGNVLTDSPEELWRRAVERWHD PFVVETLAPVRTMEQWAEATRKIDYHFGSEADRARIDRRPEFVPTGPAARVPRQDSAQRL&LINK_LOC=protein&PAGE_TYPE=BlastSearch', 'BLAST this protein','Sros_7439')" />

 
 
   KGTLFMQPGGPGNSGVDYVRNNYAGLPAKLRESFDVFGYDVRGVGRSSALTCFDDARYTKAVTDAKGVPGPDAFGPALTEAAEFDAACQTNSGSLLPYVG TEYVARDIDLLRQALGEEQLTYYGRSFGSYIGTVYAAMFPKRVRALTLDGAYDPYKYAYRPYAYDRDQYLALDGSMSRFLDWCASDQPVCGFGDGDPRGA FEQLKKDLDANPVTTASGGRANGYTLVYRLMFNINEGKVIWPSLGAALKKAQARDNTSFLLRPPSPASFDFLGPNVVVECVDKDYPKSLHKLEWNVESNA AAAPLLGPAMAFGPPTYDHQHATACVQWPAETPSRYDGSFRAKGSAPILVLGTTGDPDTPYEHAVALSHQLDNAALVTFEAEGHTAFGRSACATDAVIDY LVDLKVPARGTTCADETQPPSSTPKTAPPGTTLGELRNGVNERVDSLGKVG', 'http://blast.ncbi.nlm.nih.gov/Blast.cgi?PAGE=Proteins&PROGRAM=blastp&BLAST_PROGRAMS=blastp&QUERY=MTTFHHAPRRFSRLRIVGAALTALVLAGTGTATQAMAAQDTAQKPQSAYTPPPVPTLAWSDCQGGFECANADVPLDYRQPQGTKITLAVVRKKAADQTKR KGTLFMQPGGPGNSGVDYVRNNYAGLPAKLRESFDVFGYDVRGVGRSSALTCFDDARYTKAVTDAKGVPGPDAFGPALTEAAEFDAACQTNSGSLLPYVG TEYVARDIDLLRQALGEEQLTYYGRSFGSYIGTVYAAMFPKRVRALTLDGAYDPYKYAYRPYAYDRDQYLALDGSMSRFLDWCASDQPVCGFGDGDPRGA FEQLKKDLDANPVTTASGGRANGYTLVYRLMFNINEGKVIWPSLGAALKKAQARDNTSFLLRPPSPASFDFLGPNVVVECVDKDYPKSLHKLEWNVESNA AAAPLLGPAMAFGPPTYDHQHATACVQWPAETPSRYDGSFRAKGSAPILVLGTTGDPDTPYEHAVALSHQLDNAALVTFEAEGHTAFGRSACATDAVIDY LVDLKVPARGTTCADETQPPSSTPKTAPPGTTLGELRNGVNERVDSLGKVG&LINK_LOC=protein&PAGE_TYPE=BlastSearch', 'BLAST this protein','SCAB_43901')" />

 
 
   DIDGRYRSGGFAYLGPGFNPGSPAGGASGSGGSGSAK', 'http://blast.ncbi.nlm.nih.gov/Blast.cgi?PAGE=Proteins&PROGRAM=blastp&BLAST_PROGRAMS=blastp&QUERY=MSRFSARVRLAPILLLVLAATACGGGVRDYIADTYALQNTAGDAKTYTSNDPVGTTVSNIVAEEEPAARKADGGSEYLRYDDDIVTVSAGPGGGSLVRVE DIDGRYRSGGFAYLGPGFNPGSPAGGASGSGGSGSAK&LINK_LOC=protein&PAGE_TYPE=BlastSearch', 'BLAST this protein','RHA1_ro02315')" />
   DIDGRYRSGGYAYLGPGFNPGSPAGGAGGSGGSGSAK', 'http://blast.ncbi.nlm.nih.gov/Blast.cgi?PAGE=Proteins&PROGRAM=blastp&BLAST_PROGRAMS=blastp&QUERY=MSRFGTHVRLAPMLLLVLAATACGGGVRDYIADTYALQNTAGDAKTYTSKDPVGTTVSTIVAEEPPAARKADGGNEYLRYDDDIVTVSGAPGGGSLVRVE DIDGRYRSGGYAYLGPGFNPGSPAGGAGGSGGSGSAK&LINK_LOC=protein&PAGE_TYPE=BlastSearch', 'BLAST this protein','ROP_20330')" />

 
 
   LPSYKDANWSRGTASGGGLYPIGVYWLSGPSGPLLPGAYHYSPGHHAMQRLVVGDPTGEVRAAVGDEALTADTDQFLVLGIKFWQNAFKYNSFSYHAVTM DVGTVLQTWRMWAGARGLRIDPLLWFDEQRLSRLLGVSTEDEGLFAVVPVRWDAPSAPTAEPATERLTEPPNERPTEPPIQVRRTDQERSRTVLTFDTIR RVHAATIEHATQRPDRLALEAARAHAPDERREAATLPEPRPLQATVRAALHARRSSFGRFSAQRTIAADQLSAVLAAAAAGAALECDVTKPGGAELVKLY AFVSHVDQIAPASYEYDPQEGALRMVKPGAPGSFLQRNYFLANYNLEQAAAVLVPSVRTHAVLDAVGDRGIRLVNALVGAVAQAVYTASAAAGIACGVAL GFDTISYIEELDLHQAGEIPLLTMMIGAERPRPADFRHDFGPLGPVPGSVR', 'http://blast.ncbi.nlm.nih.gov/Blast.cgi?PAGE=Proteins&PROGRAM=blastp&BLAST_PROGRAMS=blastp&QUERY=MGFAHEYATAVAWRGRVLMEPADFVPNWADKPRRAKYYPGALGFPLPDTEDEAAASVQKGLFDPAGSQPFTLSLLGGMLRDSYGLIGRRLGVQANTDLAA LPSYKDANWSRGTASGGGLYPIGVYWLSGPSGPLLPGAYHYSPGHHAMQRLVVGDPTGEVRAAVGDEALTADTDQFLVLGIKFWQNAFKYNSFSYHAVTM DVGTVLQTWRMWAGARGLRIDPLLWFDEQRLSRLLGVSTEDEGLFAVVPVRWDAPSAPTAEPATERLTEPPNERPTEPPIQVRRTDQERSRTVLTFDTIR RVHAATIEHATQRPDRLALEAARAHAPDERREAATLPEPRPLQATVRAALHARRSSFGRFSAQRTIAADQLSAVLAAAAAGAALECDVTKPGGAELVKLY AFVSHVDQIAPASYEYDPQEGALRMVKPGAPGSFLQRNYFLANYNLEQAAAVLVPSVRTHAVLDAVGDRGIRLVNALVGAVAQAVYTASAAAGIACGVAL GFDTISYIEELDLHQAGEIPLLTMMIGAERPRPADFRHDFGPLGPVPGSVR&LINK_LOC=protein&PAGE_TYPE=BlastSearch', 'BLAST this protein','Caci_3691')" />
   SFQWSRNTASGGSLYPVNVYRYSPGDSHLPAGLYLFNPITCQWQQLRADSPRGERSRSAGETLLVTVEFWRSAFKYGDFAYQATSVDVGIVVAALVSQLD AAVGPVAIDWSPDELALSEFLGSDPLDEAIYCTITLPNSSPSDTVTAGAPSAVLQTAARLAHSGTMPVRFPTTVALQKQRLREMQSMRAPFSTERIAAPP PAIIKRGSSSFGRYSGAPIDVGVLTRMVQRGRATAASLLGTPPETDYSSGIQAAALCVNVMDLAHSLIADSETYPAAAPARPCPQLPELLRNTYLLKNYD PLRSSAVLVLCADLQRVTTTYGASGYRWACAEVGAFCHAVYAVAAQERVSVGAVLGFDAQYQRNYLGLADNLIPVLNILVGVDRPHARWRNSLL', 'http://blast.ncbi.nlm.nih.gov/Blast.cgi?PAGE=Proteins&PROGRAM=blastp&BLAST_PROGRAMS=blastp&QUERY=MDRHTADFWATTDNAATEYTQLILERKEHGMVFPAQGPFWNHQPYPAKIVPDAPRFQLHTHAMSPTDIAIAQALEDSLIRTHLRAEVDCNSPTRTRSEAQ SFQWSRNTASGGSLYPVNVYRYSPGDSHLPAGLYLFNPITCQWQQLRADSPRGERSRSAGETLLVTVEFWRSAFKYGDFAYQATSVDVGIVVAALVSQLD AAVGPVAIDWSPDELALSEFLGSDPLDEAIYCTITLPNSSPSDTVTAGAPSAVLQTAARLAHSGTMPVRFPTTVALQKQRLREMQSMRAPFSTERIAAPP PAIIKRGSSSFGRYSGAPIDVGVLTRMVQRGRATAASLLGTPPETDYSSGIQAAALCVNVMDLAHSLIADSETYPAAAPARPCPQLPELLRNTYLLKNYD PLRSSAVLVLCADLQRVTTTYGASGYRWACAEVGAFCHAVYAVAAQERVSVGAVLGFDAQYQRNYLGLADNLIPVLNILVGVDRPHARWRNSLL&LINK_LOC=protein&PAGE_TYPE=BlastSearch', 'BLAST this protein','CULC22_01775')" />
   SDLGVLPSYAYANWHRGAASGGGLYPCSVYWVAGPGAGVTPGVYYYAHARHAMQRLLGGDVSARVNAAVAPPHPASQFLIVGVKYWQNAFKYNNFSYHVV SMDLGTLLQSWRLWAGAQGRQIRPVLWFDQAAVADLLGLAPDDETLFAAVPLTWAAPAAPAALAPTSAPTRRREPATVRVRHRDQERSQTLLTFDTLRQV SRSTAASVDRPATGALAPAAAHPTPPGGTRLPLPAAAPLTLSVEAALTARRSSFGRFLHNRPMAAEQLAALLRATTASTVPSEIDGPADRPLTRIYAFVN AVANVPAGGYVYDPQEHSLVAVTSGPPGAFLQRNYTLANYNLEQAAVVLVLTVRTHAVLDATGDRGYNLVNATIGAMAQTFYTVAAALHLGAGVALGFDG ISYVEELGLADSDEFPLLIMLAGEERGQLGDYRYELR', 'http://blast.ncbi.nlm.nih.gov/Blast.cgi?PAGE=Proteins&PROGRAM=blastp&BLAST_PROGRAMS=blastp&QUERY=MTSENPGLAHAYATAILRRGREPMPPADFTPNWADAPRRGKYYPRASAFGLPAPPSAGVDLDAALHGPGDADEPFTLPLLAGLLHHSYGLLGRRLGIQAN SDLGVLPSYAYANWHRGAASGGGLYPCSVYWVAGPGAGVTPGVYYYAHARHAMQRLLGGDVSARVNAAVAPPHPASQFLIVGVKYWQNAFKYNNFSYHVV SMDLGTLLQSWRLWAGAQGRQIRPVLWFDQAAVADLLGLAPDDETLFAAVPLTWAAPAAPAALAPTSAPTRRREPATVRVRHRDQERSQTLLTFDTLRQV SRSTAASVDRPATGALAPAAAHPTPPGGTRLPLPAAAPLTLSVEAALTARRSSFGRFLHNRPMAAEQLAALLRATTASTVPSEIDGPADRPLTRIYAFVN AVANVPAGGYVYDPQEHSLVAVTSGPPGAFLQRNYTLANYNLEQAAVVLVLTVRTHAVLDATGDRGYNLVNATIGAMAQTFYTVAAALHLGAGVALGFDG ISYVEELGLADSDEFPLLIMLAGEERGQLGDYRYELR&LINK_LOC=protein&PAGE_TYPE=BlastSearch', 'BLAST this protein','Sare_2589')" />
   YGLTGRRLGVQANTDLDALPFYPLANWSRGSASGGGLYPVGIHWVSGPSGPVPPGVHYYSTRHHSMQRLLTGDVSDRVRAALGEDAPGPETDQYLVLSIK YWQNSFKYNSFSFHAVSMDLGACVQTWRMWAAARGLAVEPALWFDEERLAELLGVDPEAEGIFAVVPLKWAGPQPPSGRPAAEGPVAVRRGDVERSRTVL TFDALVRMQAATSADAAARPGPGALAPAAAHPVDRALAEVALPPARALDADVRTALRARRSSFGRFDAQRPVTADQLAACLAAASAGSRIGGDTGDVRLT RLYAFVNHVEGVEPGSYAYDPERRGLRRVKEGRPGEFLQRNYFLSNYNLEQAGAVLVPTVRTTAVLDAVGDRGYRLVNATIGAVAQSVYTAAAAMDLGCG VALGFDNISYIEELGLEGTGEAPLLIMMIGNERPAPADFRYEIA', 'http://blast.ncbi.nlm.nih.gov/Blast.cgi?PAGE=Proteins&PROGRAM=blastp&BLAST_PROGRAMS=blastp&QUERY=MTASPLHPPAATKELAMGYAHEYADAIMHRGRVPMDPADYVPNWQDGPRKAKYHPGADGFPLPDASYPAGATLDRGLFPEGGPAEGEFDLVALSGMLRDS YGLTGRRLGVQANTDLDALPFYPLANWSRGSASGGGLYPVGIHWVSGPSGPVPPGVHYYSTRHHSMQRLLTGDVSDRVRAALGEDAPGPETDQYLVLSIK YWQNSFKYNSFSFHAVSMDLGACVQTWRMWAAARGLAVEPALWFDEERLAELLGVDPEAEGIFAVVPLKWAGPQPPSGRPAAEGPVAVRRGDVERSRTVL TFDALVRMQAATSADAAARPGPGALAPAAAHPVDRALAEVALPPARALDADVRTALRARRSSFGRFDAQRPVTADQLAACLAAASAGSRIGGDTGDVRLT RLYAFVNHVEGVEPGSYAYDPERRGLRRVKEGRPGEFLQRNYFLSNYNLEQAGAVLVPTVRTTAVLDAVGDRGYRLVNATIGAVAQSVYTAAAAMDLGCG VALGFDNISYIEELGLEGTGEAPLLIMMIGNERPAPADFRYEIA&LINK_LOC=protein&PAGE_TYPE=BlastSearch', 'BLAST this protein','SGR_4411')" />
   SDLGVLPSYAYANWYRGAASGGGLYPCSVYWVAGPGAGVTPGVYYYAHARHAMQRLLGGDVSARVNAAVAPPHHASQFLIVGVKYWQNAFKYNNFSYHVV SMDLGTLLQAWRLWAGAQGRQIRPVLWFDQAAVADLLGLAPDDETLFAAVPLTWAEPTPPASLPAGPTAPAGRGEPATVRVRHHDQERSRTLLDFDALRR VSRSTAASVERPAIGALASAAAHPAPPGGTRVPLPTAAPLTLPVEAALTARRSSFGRFLRNRPMAAEHLAALLEATAASMVPSEIEGPADRPLARIYAFV NAVAAVPAGGYAYDPQEHSLILVTSGPPGAFLQRNYTLTNYNLEQAAVVLVATVRTHAVLDATGDRGYNLANATIGAMAQTFYTVAAALCLGAGVALGFD GVSYVEELGLADSDEFPLLIMLAGEERGQLGNYRYELR', 'http://blast.ncbi.nlm.nih.gov/Blast.cgi?PAGE=Proteins&PROGRAM=blastp&BLAST_PROGRAMS=blastp&QUERY=MTSENPGSAHAYATAILHRSRAPMPPADFAPNWADAPRRGKYYPQASAFGLPTPPSTGVDLDTALRGPGDVDEPFTLPLLAGLLYHSYGLLGRRLGIQAN SDLGVLPSYAYANWYRGAASGGGLYPCSVYWVAGPGAGVTPGVYYYAHARHAMQRLLGGDVSARVNAAVAPPHHASQFLIVGVKYWQNAFKYNNFSYHVV SMDLGTLLQAWRLWAGAQGRQIRPVLWFDQAAVADLLGLAPDDETLFAAVPLTWAEPTPPASLPAGPTAPAGRGEPATVRVRHHDQERSRTLLDFDALRR VSRSTAASVERPAIGALASAAAHPAPPGGTRVPLPTAAPLTLPVEAALTARRSSFGRFLRNRPMAAEHLAALLEATAASMVPSEIEGPADRPLARIYAFV NAVAAVPAGGYAYDPQEHSLILVTSGPPGAFLQRNYTLTNYNLEQAAVVLVATVRTHAVLDATGDRGYNLANATIGAMAQTFYTVAAALCLGAGVALGFD GVSYVEELGLADSDEFPLLIMLAGEERGQLGNYRYELR&LINK_LOC=protein&PAGE_TYPE=BlastSearch', 'BLAST this protein','Strop_2434')" />
   NQDVAKRAHFEHAVWGRGTASGGGMYPVEVHLVAGASAPLLPGVYHYDTGRHVLQRLLVGDVSSNVRAGLAGPEDTDSFLVASIRFWKNSFKYNSFCYHV VTQDLGALLGSWELLASALGVPLRRLLCFDPEPMDRLLGFDSDEQSVFAVVPLSWHTDTGAGDPAVPTRAGDGEVTPATVQVGHAAHERSQRVRRFPAVT AVHRAAATAPAVDPAAVLDAAPRPLPGDPVALPEPRLDLLDRPLSAVLTGRSSSFGRFRRPPELTVAELGTTLAFAAAGRPHPADAGLPADAAALTRLWV FANHLDGLPAGGYAYCARRHALLPAASEPDIGMSAFLQQQYFLTNYTMGQVGAVLAVSGRLDAVLDAYGPRGYRILNAEIGAVAQRVYCATTAQRVGCGA VLGFDNVSMDTALGLDGSDERTVLFLLLGRNSDAVADLEYRL', 'http://blast.ncbi.nlm.nih.gov/Blast.cgi?PAGE=Proteins&PROGRAM=blastp&BLAST_PROGRAMS=blastp&QUERY=MQLPARDIVREYTEAVFRRARVPMEPLNFEVDWGDQPSRHTAYPAARTVPLPTRLPELATLRDLFTGASPSAPTGWSMTRLAALLRLSYGVLDRRMTVNW NQDVAKRAHFEHAVWGRGTASGGGMYPVEVHLVAGASAPLLPGVYHYDTGRHVLQRLLVGDVSSNVRAGLAGPEDTDSFLVASIRFWKNSFKYNSFCYHV VTQDLGALLGSWELLASALGVPLRRLLCFDPEPMDRLLGFDSDEQSVFAVVPLSWHTDTGAGDPAVPTRAGDGEVTPATVQVGHAAHERSQRVRRFPAVT AVHRAAATAPAVDPAAVLDAAPRPLPGDPVALPEPRLDLLDRPLSAVLTGRSSSFGRFRRPPELTVAELGTTLAFAAAGRPHPADAGLPADAAALTRLWV FANHLDGLPAGGYAYCARRHALLPAASEPDIGMSAFLQQQYFLTNYTMGQVGAVLAVSGRLDAVLDAYGPRGYRILNAEIGAVAQRVYCATTAQRVGCGA VLGFDNVSMDTALGLDGSDERTVLFLLLGRNSDAVADLEYRL&LINK_LOC=protein&PAGE_TYPE=BlastSearch', 'BLAST this protein','VAB18032_21130')" />

 
 
   SDVETGEVLLAWGPDWVLTVRHGDVTRLDGVRSRLEHSPDLMRHGPAAAVFVVADHIVDIYTAVDLELNDDLIRVEQATFADGGQVDVSDIYALKREILE ARGAVHPLVKPVRQLIAADELVPSPLNPYFSDVQDHLLRADDNIMDYDQSLTDILQAHLAMVSVKQGEDARQISAWAALAVIPTIVGAIYGMNFDHMPEL HTAWGYPVVLLLTAAAVVLLYWRFRRSGWL', 'http://blast.ncbi.nlm.nih.gov/Blast.cgi?PAGE=Proteins&PROGRAM=blastp&BLAST_PROGRAMS=blastp&QUERY=MIVDKAVYAGGRRHECPDAAAGLADARRRNEAGGPESERAFVWVGLFEPTGDEVREFTELFAMHPLMVEDIVTGRQRPKLDVVDDTAMLVFRTLAYFEEN SDVETGEVLLAWGPDWVLTVRHGDVTRLDGVRSRLEHSPDLMRHGPAAAVFVVADHIVDIYTAVDLELNDDLIRVEQATFADGGQVDVSDIYALKREILE ARGAVHPLVKPVRQLIAADELVPSPLNPYFSDVQDHLLRADDNIMDYDQSLTDILQAHLAMVSVKQGEDARQISAWAALAVIPTIVGAIYGMNFDHMPEL HTAWGYPVVLLLTAAAVVLLYWRFRRSGWL&LINK_LOC=protein&PAGE_TYPE=BlastSearch', 'BLAST this protein','Ksed_13880')" />

 
 
   MLRAPENLTVLFDRTSLTETITGTPVPVEPDAATVEAMTREFLRVLGLLPVVLGRGEHALGVAGFGLLHGMLVQALRLTARVVDPGGVLSLKHLIPEDVY QRLEEIPAVEAEPASLIDAHLACARLFLPTAHRLHEKTGADWPTAMAEALETHLRKTVDERFAALTTWR', 'http://blast.ncbi.nlm.nih.gov/Blast.cgi?PAGE=Proteins&PROGRAM=blastp&BLAST_PROGRAMS=blastp&QUERY=MKYPALIDRLTAAFSAEPRVLGVFLIGSRGRGTADDHSDVDLLLCAEPEHHTGLCDDMPQLVAEATETVHSQRVGTLPVFTYIAAGWLRFDISVASPAEL MLRAPENLTVLFDRTSLTETITGTPVPVEPDAATVEAMTREFLRVLGLLPVVLGRGEHALGVAGFGLLHGMLVQALRLTARVVDPGGVLSLKHLIPEDVY QRLEEIPAVEAEPASLIDAHLACARLFLPTAHRLHEKTGADWPTAMAEALETHLRKTVDERFAALTTWR&LINK_LOC=protein&PAGE_TYPE=BlastSearch', 'BLAST this protein','Snas_1130')" />

 
 
   LRTVLEEYASGAAALRRSESQLQQLRELLADADAACAGEDPAAATGVDALFHRAVVRASGNDLLVEVYDHLGTALTASLGGLPWDAGHAAEHARLHRRLV DAIEARDTGGARDAAAAIVRLTRDHETDASRTAGER', 'http://blast.ncbi.nlm.nih.gov/Blast.cgi?PAGE=Proteins&PROGRAM=blastp&BLAST_PROGRAMS=blastp&QUERY=MKRISAPRRTASLSAQLVDSLRSHIESGGWPVGTRIPPEHALIEELGVGRSTLREAIGALVHLGLLEPRAGDGTYVCSSSELQSVMVRRASSAQRDKVLE LRTVLEEYASGAAALRRSESQLQQLRELLADADAACAGEDPAAATGVDALFHRAVVRASGNDLLVEVYDHLGTALTASLGGLPWDAGHAAEHARLHRRLV DAIEARDTGGARDAAAAIVRLTRDHETDASRTAGER&LINK_LOC=protein&PAGE_TYPE=BlastSearch', 'BLAST this protein','SCO_7702')" />

 
 
   DVAPITIGEDCQFGPGVQLLTPTHPVEPEPRRDKLEAAKPITIGDNVWLGGGVVVCPGVTIGDNSVIGAGAVVTRDVPPNAVAVGNPARVVREIGVEG', 'http://blast.ncbi.nlm.nih.gov/Blast.cgi?PAGE=Proteins&PROGRAM=blastp&BLAST_PROGRAMS=blastp&QUERY=MPRDYFAGDPRTNRERMLAGELYIADDPESERIARRALALLEEYRVAFTASRDEEARRLLAEVLGEVGEGVVVKPPLFVDYGENIRIGARTFVNYNLTAL DVAPITIGEDCQFGPGVQLLTPTHPVEPEPRRDKLEAAKPITIGDNVWLGGGVVVCPGVTIGDNSVIGAGAVVTRDVPPNAVAVGNPARVVREIGVEG&LINK_LOC=protein&PAGE_TYPE=BlastSearch', 'BLAST this protein','Amir_4253')" />
   TVGADVQMGPNVQLLTPTHPIDPEQRRAKWEAAQPITIGDNVWLGGGVIVCPGVAIGENTVVGAGAVVTKDLPANVVAVGNPARVIRRIGEAEA', 'http://blast.ncbi.nlm.nih.gov/Blast.cgi?PAGE=Proteins&PROGRAM=blastp&BLAST_PROGRAMS=blastp&QUERY=MSDETSRSQKEAMLSGELYIADDPELAAEARHAAVLSERFNATSAADPEARRAVLAELIGELGEDVEVRPPLRVDYGYQITLGRGTFINFGAVLLDVARI TVGADVQMGPNVQLLTPTHPIDPEQRRAKWEAAQPITIGDNVWLGGGVIVCPGVAIGENTVVGAGAVVTKDLPANVVAVGNPARVIRRIGEAEA&LINK_LOC=protein&PAGE_TYPE=BlastSearch', 'BLAST this protein','SGR_2400')" />

 
 
   EADWLDGMLQLAGAQLALYRRHRWLLDVSHQPPGPGPETLAWFDNFLRILEPVRCAPTAKFEAIGMMTGVVSLFVRAEAAAGSFSFAGVDLAAYPHLAAA FCQPPATEPPSNLFERTLRSLLTGLLAAPVAGPQA', 'http://blast.ncbi.nlm.nih.gov/Blast.cgi?PAGE=Proteins&PROGRAM=blastp&BLAST_PROGRAMS=blastp&QUERY=MADIVDGFHSDPVRANAWVLEHLAARAASSVDRTVTGQVTRWASRAVADRDGLAVVSMRAVAAALGTSAGSLYRYLSSRGDLLDLMSDRVVGELRPYPPA EADWLDGMLQLAGAQLALYRRHRWLLDVSHQPPGPGPETLAWFDNFLRILEPVRCAPTAKFEAIGMMTGVVSLFVRAEAAAGSFSFAGVDLAAYPHLAAA FCQPPATEPPSNLFERTLRSLLTGLLAAPVAGPQA&LINK_LOC=protein&PAGE_TYPE=BlastSearch', 'BLAST this protein','Franean1_5781')" />
   HLALQSRDAHVRHPWLADLNDRRGEVLGPHAIDYLDHALGILAPAPGTAGQKLEAVGLLGGLAVLFARRANGAAGGAAHGGDPAARAAHLAAVAAEGRHP HLLAALTSAGPPPADRDALFVTLLRRLLPAMLDQGAA', 'http://blast.ncbi.nlm.nih.gov/Blast.cgi?PAGE=Proteins&PROGRAM=blastp&BLAST_PROGRAMS=blastp&QUERY=MADRETRPTTVWSRPQRGARGPAPERSRTQITAAALALADAEGLAAVSMRALAQRLGTGPASLYRYVGSRDELLDLMADAVAGELDLSGSSGGDWLDALV HLALQSRDAHVRHPWLADLNDRRGEVLGPHAIDYLDHALGILAPAPGTAGQKLEAVGLLGGLAVLFARRANGAAGGAAHGGDPAARAAHLAAVAAEGRHP HLLAALTSAGPPPADRDALFVTLLRRLLPAMLDQGAA&LINK_LOC=protein&PAGE_TYPE=BlastSearch', 'BLAST this protein','SGR_2601')" />

 
 
   ASPQAQEAFYRIVDTLTRLGTSGAPLAVHPVSEPAQALLAALGDTLLPVLDAVEPAVREASAALGELVDATSPLLPAAAGAAAGTLLALTPLIRAAADVL RDSSPELRRVADALTAALTPLLPLQVALAERAAAAGAGVVRAALPPLADLTEAAATTAKAARPLLIAGEERLVAGLEQIQPLLVATASRTGRAARAVAVR VSAAVSPAADQGVVVAVTPV', 'http://blast.ncbi.nlm.nih.gov/Blast.cgi?PAGE=Proteins&PROGRAM=blastp&BLAST_PROGRAMS=blastp&QUERY=MAGRPRARMPGWCLPGGPDGACWTRHRARRGDPMSPIPSLPSRDDVVRMARSTTDITGQLLDTAGNITKIAINTFDPRDGSGAEALRVLRQTTAELAEAS ASPQAQEAFYRIVDTLTRLGTSGAPLAVHPVSEPAQALLAALGDTLLPVLDAVEPAVREASAALGELVDATSPLLPAAAGAAAGTLLALTPLIRAAADVL RDSSPELRRVADALTAALTPLLPLQVALAERAAAAGAGVVRAALPPLADLTEAAATTAKAARPLLIAGEERLVAGLEQIQPLLVATASRTGRAARAVAVR VSAAVSPAADQGVVVAVTPV&LINK_LOC=protein&PAGE_TYPE=BlastSearch', 'BLAST this protein','SAV_5779')" />

 
 
   AGLSVVALGALLLCTRYDAPAWTLFGCAAVAGTMPNMAAMARARWTHALKGSDRLHTAYSLESVLDELTFVVGPALSVALSTALFPEAGPLAAGVLLILG VLMFVPQKRTEPPVVRPTAGHPRSGSAIRGGALRLLALTLAAGGAIVGTVDVVSVSFAEQQGSPAGAGLVLSVYAAGSALAGLVFGTLKLRTPLPRLLVI GTAGTALTTLPLLLVGDIVSLSAAVFLPGLFFAPTMIVVMGLVERTVPPAALTEGMTWAITGLSVGVASGAAVSGAIVDRFGPGGGFTVAVAAGTAALLT ALCARRPLERSLRERTDRDALGPIEAPPGAPDRSAH', 'http://blast.ncbi.nlm.nih.gov/Blast.cgi?PAGE=Proteins&PROGRAM=blastp&BLAST_PROGRAMS=blastp&QUERY=MTARPKGRPHDCPGGPRIANPYGALFRTPGSRAFTAAGLVARMPLSMAGIGVIAMVSQARGDYGLAGAVSATFTLSMALCGPRISRAVDRRGQSRVLPPV AGLSVVALGALLLCTRYDAPAWTLFGCAAVAGTMPNMAAMARARWTHALKGSDRLHTAYSLESVLDELTFVVGPALSVALSTALFPEAGPLAAGVLLILG VLMFVPQKRTEPPVVRPTAGHPRSGSAIRGGALRLLALTLAAGGAIVGTVDVVSVSFAEQQGSPAGAGLVLSVYAAGSALAGLVFGTLKLRTPLPRLLVI GTAGTALTTLPLLLVGDIVSLSAAVFLPGLFFAPTMIVVMGLVERTVPPAALTEGMTWAITGLSVGVASGAAVSGAIVDRFGPGGGFTVAVAAGTAALLT ALCARRPLERSLRERTDRDALGPIEAPPGAPDRSAH&LINK_LOC=protein&PAGE_TYPE=BlastSearch', 'BLAST this protein','SGR_6837')" />

 
 
   TPEEPATQPLPVMVFIHGGGYILGSSATPIYDGAALARRGCVYVSVNYRLGALGCLDLSSLSTPQITLDSNVYLRDLVLALRWVHDNIAEFGGDPGNVTI FGESAGAHITATLLAVPAAKGLFARAISESPAAGMVRSREVAAEFAARFANLIGARTQDAANALMQASPAQLVEAQHHLIRQGMRKRLGAFPIGPVFGDD YLPMDPVEAMRSGRVHAVPLIVGTNAEEGRLFTRFLGMLPTNEPMVEELLSGMKPADRERITAAYPNYPAPSACIQLGGDFAFSSAAWQIAEAHGANAPT YLYRYDYAPRTLRWSGFGATHATELFAVFDIYRTRFGALLTAAADRRAALRVSNEVQRRWRCFSQIGVPGDDWPAYTQDDRAVLVFDRRCRIEFDPHQHR RIAWDGFSLAN', 'http://blast.ncbi.nlm.nih.gov/Blast.cgi?PAGE=Proteins&PROGRAM=blastp&BLAST_PROGRAMS=blastp&QUERY=MALESATVGSMHERTVRARTATGIVEGFTRDGVHRWRSIPYARAPVGSLRFRAPQPAQPWPGVRHCHTFANCAPQQRRYTVMGIGRYQTRSEDCLTLNVV TPEEPATQPLPVMVFIHGGGYILGSSATPIYDGAALARRGCVYVSVNYRLGALGCLDLSSLSTPQITLDSNVYLRDLVLALRWVHDNIAEFGGDPGNVTI FGESAGAHITATLLAVPAAKGLFARAISESPAAGMVRSREVAAEFAARFANLIGARTQDAANALMQASPAQLVEAQHHLIRQGMRKRLGAFPIGPVFGDD YLPMDPVEAMRSGRVHAVPLIVGTNAEEGRLFTRFLGMLPTNEPMVEELLSGMKPADRERITAAYPNYPAPSACIQLGGDFAFSSAAWQIAEAHGANAPT YLYRYDYAPRTLRWSGFGATHATELFAVFDIYRTRFGALLTAAADRRAALRVSNEVQRRWRCFSQIGVPGDDWPAYTQDDRAVLVFDRRCRIEFDPHQHR RIAWDGFSLAN&LINK_LOC=protein&PAGE_TYPE=BlastSearch', 'BLAST this protein','MAF_20600')" />
   TPEEPATQPLPVMVFIHGGGYILGSSATPIYDGAALARRGCVYVSVNYRLGALGCLDLSSLSTPQITLDSNVYLRDLVLALRWVHDNIAEFGGDPGNVTI FGESAGAHITATLLAVPAAKGLFARAISESPAAGMVRSREVAAEFAARFANLIGARTQDAANALMQASPAQLVEAQHHLIRQGMRKRLGAFPIGPVFGDD YLPMDPVEAMRSGRVHAVPLIVGTNAEEGRLFTRFLGMLPTNEPMVEELLSGMKPADRERITAAYPNYPAPSACIQLGGDFAFSSAAWQIAEAHGANAPT YLYRYDYAPRTLRWSGFGATHATELFAVFDIYRTRFGALLTAAADRRAALRVSNEVQRRWRCFSQIGVPGDDWPAYTQDDRAVLVFDRRCRIEFDPHQHR RIAWDGFSLAN', 'http://blast.ncbi.nlm.nih.gov/Blast.cgi?PAGE=Proteins&PROGRAM=blastp&BLAST_PROGRAMS=blastp&QUERY=MALESATVGSMHERTVRARTATGIVEGFTRDGVHRWRSIPYARAPVGSLRFRAPQPAQPWPGVRHCHTFANCAPQQRRYTVMGIGRYQTRSEDCLTLNVV TPEEPATQPLPVMVFIHGGGYILGSSATPIYDGAALARRGCVYVSVNYRLGALGCLDLSSLSTPQITLDSNVYLRDLVLALRWVHDNIAEFGGDPGNVTI FGESAGAHITATLLAVPAAKGLFARAISESPAAGMVRSREVAAEFAARFANLIGARTQDAANALMQASPAQLVEAQHHLIRQGMRKRLGAFPIGPVFGDD YLPMDPVEAMRSGRVHAVPLIVGTNAEEGRLFTRFLGMLPTNEPMVEELLSGMKPADRERITAAYPNYPAPSACIQLGGDFAFSSAAWQIAEAHGANAPT YLYRYDYAPRTLRWSGFGATHATELFAVFDIYRTRFGALLTAAADRRAALRVSNEVQRRWRCFSQIGVPGDDWPAYTQDDRAVLVFDRRCRIEFDPHQHR RIAWDGFSLAN&LINK_LOC=protein&PAGE_TYPE=BlastSearch', 'BLAST this protein','Mb_2071c')" />
   TPEEPATQPLPVMVFIHGGGYILGSSATPIYDGAALARRGCVYVSVNYRLGALGCLDLSSLSTPQITLDSNVYLRDLVLALRWVHDNIAEFGGDPGNVTI FGGSAGAHITATLLAVPAAKGLFARAISESPAAGMVRSREVAAEFAARFANLIGARTQDAANALMQASPAQLVEAQHHLIRQGMRKRLGAFPIGPVFGDD YLPMDPVEAMRSGRVHAVPLIVGTNAEEGRLFTRFLGMLPTNEPMVEELLSGMKPADRERITAAYPNYPAPSACIQLGGDFAFSSAAWQIAEAHGANAPT YLYRYDYAPRTLRWSGFGATHATELFAVFDIYRTRFGALLTAAADRRAALRVSNEVQRRWRCFSQIGVPGDDWPAYTQDDRAVLVFDRRCRIEFDPHQHR RIAWDGFSLAN', 'http://blast.ncbi.nlm.nih.gov/Blast.cgi?PAGE=Proteins&PROGRAM=blastp&BLAST_PROGRAMS=blastp&QUERY=MALESATVGSMHERTVRARTATGIVEGFTRDGVHRWRSIPYARAPVGSLRFRAPQPAQPWPGVRHCHTFANCAPQQRRYTVMGIGRYQTRSEDCLTLNVV TPEEPATQPLPVMVFIHGGGYILGSSATPIYDGAALARRGCVYVSVNYRLGALGCLDLSSLSTPQITLDSNVYLRDLVLALRWVHDNIAEFGGDPGNVTI FGGSAGAHITATLLAVPAAKGLFARAISESPAAGMVRSREVAAEFAARFANLIGARTQDAANALMQASPAQLVEAQHHLIRQGMRKRLGAFPIGPVFGDD YLPMDPVEAMRSGRVHAVPLIVGTNAEEGRLFTRFLGMLPTNEPMVEELLSGMKPADRERITAAYPNYPAPSACIQLGGDFAFSSAAWQIAEAHGANAPT YLYRYDYAPRTLRWSGFGATHATELFAVFDIYRTRFGALLTAAADRRAALRVSNEVQRRWRCFSQIGVPGDDWPAYTQDDRAVLVFDRRCRIEFDPHQHR RIAWDGFSLAN&LINK_LOC=protein&PAGE_TYPE=BlastSearch', 'BLAST this protein','MCAN_20681')" />
   PVMVFIHGGGYILGSSATPIYDGAALARRGCVYVSVNYRLGALGCLDLSSLSTPDIAIDGNLYLRDLVMALQWIQDNIAEFGGDPDNVTIFGESAGAHIT ATLLAVPAAKGLFARAISESPAAGMVRPREIAAEFATRFARLLGARKHDAASALMRATAAQLVQTQHRLIDQGMQKRLGAFPIGPTFGDDCLPLDPVEAM RAGQSHKVPLIVGTNADEGRLFTRFLKMLPTNKAMIDELLADTEPATRERITAAYPNYPESAACIQLGGDFAFNAAAWQIAEAQGAHAPTYLYRYDYAPR TLRWSGFGATHATELFAVFDIYRTRFGALLTAAADRRHALRVSDEVIRRWRSFSQTGTPGEDWPAYTHTRRAVMVIDRKSRVEIDPHQHRRLAWDGFSLA R', 'http://blast.ncbi.nlm.nih.gov/Blast.cgi?PAGE=Proteins&PROGRAM=blastp&BLAST_PROGRAMS=blastp&QUERY=MHERTVRARTATGIVEGFTRDGVHRWRSIPYARAPIGPLRFRAPRPPQPWSGVRHCHGFTHCSPQQRRYTMLGLGKYQPMSEDCLTLNVVAPEAPSHRPL PVMVFIHGGGYILGSSATPIYDGAALARRGCVYVSVNYRLGALGCLDLSSLSTPDIAIDGNLYLRDLVMALQWIQDNIAEFGGDPDNVTIFGESAGAHIT ATLLAVPAAKGLFARAISESPAAGMVRPREIAAEFATRFARLLGARKHDAASALMRATAAQLVQTQHRLIDQGMQKRLGAFPIGPTFGDDCLPLDPVEAM RAGQSHKVPLIVGTNADEGRLFTRFLKMLPTNKAMIDELLADTEPATRERITAAYPNYPESAACIQLGGDFAFNAAAWQIAEAQGAHAPTYLYRYDYAPR TLRWSGFGATHATELFAVFDIYRTRFGALLTAAADRRHALRVSDEVIRRWRSFSQTGTPGEDWPAYTHTRRAVMVIDRKSRVEIDPHQHRRLAWDGFSLA R&LINK_LOC=protein&PAGE_TYPE=BlastSearch', 'BLAST this protein','MMAR_3020')" />
   PVMVFIHGGGYILGSSATPIYDGAALARRGCVYVSVNYRLGALGCLDLSSLSTPDIAIDGNLYLRDLVMALQWIQDNIAEFGGDPDNVTIFGESAGAHIT ATLLAVPAAKGLFARAISESPAAGMVRPREIAAEFATRFARLLGARKHDAASALMRATAAQLVQTQHRLIDQGMQKRLGAFPIGPTFGDDCLPLDPVEAM RAGQSHKVPLIVGTNADEGRLFTRFLKMLPTNKAMIDELLADTEPATRERITAAYPNYPESAACIQLGGDFAFNAAAWQIAEAQGVHAPTYLYRYDYAPR TLRWSGFGATHATELFAVFDIYRTRFGALLTAAADRRHALRVSDEVIRRWRSFSQTGTPGEDWPAYTHTRRAVMVIDRKSRVEIDPHRHRRLAWDGFSLA R', 'http://blast.ncbi.nlm.nih.gov/Blast.cgi?PAGE=Proteins&PROGRAM=blastp&BLAST_PROGRAMS=blastp&QUERY=MHERTVRARTATGVVEGFTRDGVHRWRSIPYARAPIGLLRFRAPRPPQPWSGVRHCHGFTHCSPQQRRYTMLGLGKYQPMSEDCLTLNVVAPEAPTHRPL PVMVFIHGGGYILGSSATPIYDGAALARRGCVYVSVNYRLGALGCLDLSSLSTPDIAIDGNLYLRDLVMALQWIQDNIAEFGGDPDNVTIFGESAGAHIT ATLLAVPAAKGLFARAISESPAAGMVRPREIAAEFATRFARLLGARKHDAASALMRATAAQLVQTQHRLIDQGMQKRLGAFPIGPTFGDDCLPLDPVEAM RAGQSHKVPLIVGTNADEGRLFTRFLKMLPTNKAMIDELLADTEPATRERITAAYPNYPESAACIQLGGDFAFNAAAWQIAEAQGVHAPTYLYRYDYAPR TLRWSGFGATHATELFAVFDIYRTRFGALLTAAADRRHALRVSDEVIRRWRSFSQTGTPGEDWPAYTHTRRAVMVIDRKSRVEIDPHRHRRLAWDGFSLA R&LINK_LOC=protein&PAGE_TYPE=BlastSearch', 'BLAST this protein','MUL_2261')" />
   TPEEPATQPLPVMVFIHGGGYILGSSATPIYDGAALARRGCVYVSVNYRLGALGCLDLSSLSTPQITLDSNVYLRDLVLALRWVHDNIAEFGGDPGNVTI FGESAGAHITATLLAVPAAKGLFARAISESPAAGMVRSREVAAEFAARFANLIGARTQDAANALMQASPAQLVEAQHHLIRQGMRKRLGAFPIGPVFGDD YLPMDPVEAMRSGRVHAVPLIVGTNAEEGRLFTRFLGMLPTNEPMVEELLSGMKPADRERITAAYPNYPAPSACIQLGGDFAFSSAAWQIAEAHGANAPT YLYRYDYAPRTLRWSGFGATHATELFAVFDIYRTRFGALLTAAADRRAALRVSNEVQRRWRCFSQIGVPGDDWPAYTQDDRAVLVFDRRCRIEFDPHQHR RIAWDGFSLAN', 'http://blast.ncbi.nlm.nih.gov/Blast.cgi?PAGE=Proteins&PROGRAM=blastp&BLAST_PROGRAMS=blastp&QUERY=MALESATVGSMHERTVRARTATGIVEGFTRDGVHRWRSIPYARAPVGSLRFRAPQPAQPWPGVRHCHTFANCAPQQRRYTVMGIGRYQTRSEDCLTLNVV TPEEPATQPLPVMVFIHGGGYILGSSATPIYDGAALARRGCVYVSVNYRLGALGCLDLSSLSTPQITLDSNVYLRDLVLALRWVHDNIAEFGGDPGNVTI FGESAGAHITATLLAVPAAKGLFARAISESPAAGMVRSREVAAEFAARFANLIGARTQDAANALMQASPAQLVEAQHHLIRQGMRKRLGAFPIGPVFGDD YLPMDPVEAMRSGRVHAVPLIVGTNAEEGRLFTRFLGMLPTNEPMVEELLSGMKPADRERITAAYPNYPAPSACIQLGGDFAFSSAAWQIAEAHGANAPT YLYRYDYAPRTLRWSGFGATHATELFAVFDIYRTRFGALLTAAADRRAALRVSNEVQRRWRCFSQIGVPGDDWPAYTQDDRAVLVFDRRCRIEFDPHQHR RIAWDGFSLAN&LINK_LOC=protein&PAGE_TYPE=BlastSearch', 'BLAST this protein','Rv_2045c')" />
   HSGSPGYDARRIARDGDVVVVTLNYRVGIEGFARVDGAPANRGLLDQVAALEWVRENITAFGGDPGRVTVFGESAGAGSIASLLAMPSASGLFRRAIAQS VPGTYFSDELAKDIAAAIAAEAGLRPTAADLSTVDPRQLPAAGEALAATMRQYEDRWGPVVHTLTPFSPVVDGEVLPTTPWQALAAGTARDVELIVGHNS EEFRLFVLLSGQLGKITDGEARAALRRFGPGPDAEQAYRTGFPDASPGELYERVMSDWLFHMPSLHLAEAQLTGGGRAHVYELTWPAPGNGGVLGACHGL DIPLLFGTFDADLGSLLFAGTEPSPEAEALSSRFRASWTAFARTGDPGWPTYDTERRLVQVLDAAPEVIPYPEETSRRLWERHTFPALPLIQ', 'http://blast.ncbi.nlm.nih.gov/Blast.cgi?PAGE=Proteins&PROGRAM=blastp&BLAST_PROGRAMS=blastp&QUERY=MRGRLEGGLAVFRGVPFAEPPVGDARFAAPRPVRAWDGTRDAFAFGPPPPQETGIQGRAALLDAPTGDDWLTVNVWTPDPDPGARRPVMVWIYGGAYKLG HSGSPGYDARRIARDGDVVVVTLNYRVGIEGFARVDGAPANRGLLDQVAALEWVRENITAFGGDPGRVTVFGESAGAGSIASLLAMPSASGLFRRAIAQS VPGTYFSDELAKDIAAAIAAEAGLRPTAADLSTVDPRQLPAAGEALAATMRQYEDRWGPVVHTLTPFSPVVDGEVLPTTPWQALAAGTARDVELIVGHNS EEFRLFVLLSGQLGKITDGEARAALRRFGPGPDAEQAYRTGFPDASPGELYERVMSDWLFHMPSLHLAEAQLTGGGRAHVYELTWPAPGNGGVLGACHGL DIPLLFGTFDADLGSLLFAGTEPSPEAEALSSRFRASWTAFARTGDPGWPTYDTERRLVQVLDAAPEVIPYPEETSRRLWERHTFPALPLIQ&LINK_LOC=protein&PAGE_TYPE=BlastSearch', 'BLAST this protein','SAV_7363')" />
   GRGTTGLPVLVWIHGGSLVHGSSAVPVYDGSAFARDGVVLVSVNYRLGVEGFGVLPDAPANRGLLDQLAALEWVRDNIAAFGGDPDRVTVAGESAGAVSV AALLASPRSAGLLRRAVLQSGAPAALPPAAARGTTELIAKRLGVPATAAALAAVAPEALLTAQTEVTGGGNPLTGRHSFQLVVDGELLPHDPVEALHAGA SAGIDLLMGTNTEEYRLWFVPGGLTERIGALRLRLALLKFRVPGATARVYRANRPGAAPGEILGALATDLLLRVPLNRLADARTGAPGATYLYEFGWPTP VQRLGACHALELGFVFDTLAHPDTMALTGPDAPQELADAMHRAWVDFATDGDPGWPSWDARRPVTVFGPGAPELVLAPRDDELRGWRPYRR', 'http://blast.ncbi.nlm.nih.gov/Blast.cgi?PAGE=Proteins&PROGRAM=blastp&BLAST_PROGRAMS=blastp&QUERY=MADITAGTSPRALTVHGTVRGAVERGVAVFRGIPYAAEPVGALRFRAPAPPEPWTGARETVAYGPTAPKRPYAPPLDRLLPDPAVPGDGCLNLNVWTPSP GRGTTGLPVLVWIHGGSLVHGSSAVPVYDGSAFARDGVVLVSVNYRLGVEGFGVLPDAPANRGLLDQLAALEWVRDNIAAFGGDPDRVTVAGESAGAVSV AALLASPRSAGLLRRAVLQSGAPAALPPAAARGTTELIAKRLGVPATAAALAAVAPEALLTAQTEVTGGGNPLTGRHSFQLVVDGELLPHDPVEALHAGA SAGIDLLMGTNTEEYRLWFVPGGLTERIGALRLRLALLKFRVPGATARVYRANRPGAAPGEILGALATDLLLRVPLNRLADARTGAPGATYLYEFGWPTP VQRLGACHALELGFVFDTLAHPDTMALTGPDAPQELADAMHRAWVDFATDGDPGWPSWDARRPVTVFGPGAPELVLAPRDDELRGWRPYRR&LINK_LOC=protein&PAGE_TYPE=BlastSearch', 'BLAST this protein','SGR_2081')" />

 
 
   LREQDIDTYGHLLAEAGAGDRVTGADLTAVAAGLKLPPSRHYLHAEGGLMVDGRRYLAAVRGRCRDQGVDWQQGCTVREVREEGRTTVIQTDEATLHADR VVIAAGAWSSAEGLAASTGIRPQRGQMVVLRTDEKLPSILSSAYYLAPGVGDDILVGATEEDAGFADQVTAQGIGQLLRFATAAMPSLADSVPVELRAGL RPVSDTGRPLAGAVPGHERIFISAGHAGHGLLSARASAKGMAAGLLSADWDALPYRMCPTHARGGAA', 'http://blast.ncbi.nlm.nih.gov/Blast.cgi?PAGE=Proteins&PROGRAM=blastp&BLAST_PROGRAMS=blastp&QUERY=MLSFPSSTPDFAIVGGGIVGACLAEELSSQGASVLVLDAGAEPGHATHRAAGVAVPSLRYLDDHEFYSWLRTAKLDLNADVARLEPQFGTFSVVRPILRA LREQDIDTYGHLLAEAGAGDRVTGADLTAVAAGLKLPPSRHYLHAEGGLMVDGRRYLAAVRGRCRDQGVDWQQGCTVREVREEGRTTVIQTDEATLHADR VVIAAGAWSSAEGLAASTGIRPQRGQMVVLRTDEKLPSILSSAYYLAPGVGDDILVGATEEDAGFADQVTAQGIGQLLRFATAAMPSLADSVPVELRAGL RPVSDTGRPLAGAVPGHERIFISAGHAGHGLLSARASAKGMAAGLLSADWDALPYRMCPTHARGGAA&LINK_LOC=protein&PAGE_TYPE=BlastSearch', 'BLAST this protein','Sare_3277')" />
   REQDIETYGHLLAEAGAGARVTGDDLATVATGLKLPPSRHYLHAEGGLMIDGRRYLDAVLGRCREQGVTWQQGRRVREVREDGHRVMIRTDQETLHADRV VIAAGAWSSGTGLAESTGIRPQRGQMVVLRTDEKLSSILSSAYYLAPGVGDDILVGATEEDAGFADHVTAQGIGALLRFATTAMPGLAASVPLELRAGLR PVSDTGRPLAGALPGHDRIFISAGHAGHGLLSARTTAKGMAAGLLGDDWDALPYRMCPTHAQGGTA', 'http://blast.ncbi.nlm.nih.gov/Blast.cgi?PAGE=Proteins&PROGRAM=blastp&BLAST_PROGRAMS=blastp&QUERY=MLSLPSTPDFAIVGGGIVGACLAEELSRQGASVLVLDAGAEPGHATHRAAGVAVPSLRYLDDHEFYSWLRGAKLDLDADVARLEPEFGTFSVVRPILRAL REQDIETYGHLLAEAGAGARVTGDDLATVATGLKLPPSRHYLHAEGGLMIDGRRYLDAVLGRCREQGVTWQQGRRVREVREDGHRVMIRTDQETLHADRV VIAAGAWSSGTGLAESTGIRPQRGQMVVLRTDEKLSSILSSAYYLAPGVGDDILVGATEEDAGFADHVTAQGIGALLRFATTAMPGLAASVPLELRAGLR PVSDTGRPLAGALPGHDRIFISAGHAGHGLLSARTTAKGMAAGLLGDDWDALPYRMCPTHAQGGTA&LINK_LOC=protein&PAGE_TYPE=BlastSearch', 'BLAST this protein','Strop_3051')" />

 
 
   LEPSVLAAQIDQLSGWSDDSDDLSGQKGIAAFTDSEWRAWGKDDDGNGHASPRDPADAIMALGRRDCSLAEKVTGLRTEGRVNGDLVELTLAAYAVGPDA VTGAGRVPAAAKTYLAEVQALVPRYEALDREDSADGSGGPAGALLTAPVSPLTITSPFGSRQHPLTGVTKLHTGVDFAAPQGAQVVAARRGRVVFAALTS AYGNRIVIDHGTIQGKRVETTYSHLSALETSVGQTVEAGAPIGRVGSTGLSTGPHLHFEVILDGYYTDPRPWVVANGV', 'http://blast.ncbi.nlm.nih.gov/Blast.cgi?PAGE=Proteins&PROGRAM=blastp&BLAST_PROGRAMS=blastp&QUERY=MNDERLTVAPTADDTDDPRQEPERAEGSERGPGRKRRGPGPVTLLLLPALVTVTGVAAFLALGGLPDPWPDDSDAKASTAADIDPSYVPWLRKAASACTV LEPSVLAAQIDQLSGWSDDSDDLSGQKGIAAFTDSEWRAWGKDDDGNGHASPRDPADAIMALGRRDCSLAEKVTGLRTEGRVNGDLVELTLAAYAVGPDA VTGAGRVPAAAKTYLAEVQALVPRYEALDREDSADGSGGPAGALLTAPVSPLTITSPFGSRQHPLTGVTKLHTGVDFAAPQGAQVVAARRGRVVFAALTS AYGNRIVIDHGTIQGKRVETTYSHLSALETSVGQTVEAGAPIGRVGSTGLSTGPHLHFEVILDGYYTDPRPWVVANGV&LINK_LOC=protein&PAGE_TYPE=BlastSearch', 'BLAST this protein','SCO_0543')" />

 
 
   VAAVLRGLGWTVLRFWEHVPAQDTADRIIVAVERARESASRAGHPRHADVDGSQ', 'http://blast.ncbi.nlm.nih.gov/Blast.cgi?PAGE=Proteins&PROGRAM=blastp&BLAST_PROGRAMS=blastp&QUERY=MSRASSDTSRRVMRSNMRRDTAPELAVRRILHAKGMRYRVDFRVVRETRSRADIAFTRQRIAVFIDGCFWHSCPEHLHLPKANADYWIPKLARNVERDAE VAAVLRGLGWTVLRFWEHVPAQDTADRIIVAVERARESASRAGHPRHADVDGSQ&LINK_LOC=protein&PAGE_TYPE=BlastSearch', 'BLAST this protein','CMM_1970')" />
   VVRVWEHDDPKTVAGQVAELVASRRTLRRGQRLPAAAPSTSEADRT', 'http://blast.ncbi.nlm.nih.gov/Blast.cgi?PAGE=Proteins&PROGRAM=blastp&BLAST_PROGRAMS=blastp&QUERY=MQLQRTRDTAPELALRRRLHGMGLRYRVDVQPLRDLRRRADLVFGPSKVAVFVDGCFWHGCPVHGNPRPAANTWYWPDKIAGNKARDTDTDRRLQEAGWA VVRVWEHDDPKTVAGQVAELVASRRTLRRGQRLPAAAPSTSEADRT&LINK_LOC=protein&PAGE_TYPE=BlastSearch', 'BLAST this protein','FraEuI1c_6952')" />

 
 
   GTAAAVVPVVKQRRSVGMSDVARASAGMNQLVEADDRQGGHASLATAALEARAKVLELQHRNASERVRRALYALAAEFTTVAAWSCIDLRDLDKARTYLH ESSTFAGLSQDPPTAMRVWVNMAMLAYQRKNWPEQLAAAQAANASPAARRDPFFGSMGRVRLALAHSSLGDLRAARRSLGAAQENYRKAAEDERPRWTAF YGPAELNHLAAIILNHNGEPAEAEAMAHRSLAKIPAEFQRNRALATCQLALAQLRQGEPEQATATAATVFTIMEGAPLPGRMRTLIGDFHRDLFRLAPST TYARDWADRMRDEWSRT', 'http://blast.ncbi.nlm.nih.gov/Blast.cgi?PAGE=Proteins&PROGRAM=blastp&BLAST_PROGRAMS=blastp&QUERY=MGGNLVLQDRLDQLGLTQEELAARLNAALQEITGRPGDISSRTVRNLLNGSSRRPIGRTCAALERVFGCPVADLGFSAPSSMQHPPEGPVRRRDFIASTT GTAAAVVPVVKQRRSVGMSDVARASAGMNQLVEADDRQGGHASLATAALEARAKVLELQHRNASERVRRALYALAAEFTTVAAWSCIDLRDLDKARTYLH ESSTFAGLSQDPPTAMRVWVNMAMLAYQRKNWPEQLAAAQAANASPAARRDPFFGSMGRVRLALAHSSLGDLRAARRSLGAAQENYRKAAEDERPRWTAF YGPAELNHLAAIILNHNGEPAEAEAMAHRSLAKIPAEFQRNRALATCQLALAQLRQGEPEQATATAATVFTIMEGAPLPGRMRTLIGDFHRDLFRLAPST TYARDWADRMRDEWSRT&LINK_LOC=protein&PAGE_TYPE=BlastSearch', 'BLAST this protein','SGR_3852')" />

 
 
   PLSPLLEVELLRSGVVGKLSLWETLTLHADRLGLDRAEFEELTAAAARQAETLGTIHATWAASAFPAEAADQSD', 'http://blast.ncbi.nlm.nih.gov/Blast.cgi?PAGE=Proteins&PROGRAM=blastp&BLAST_PROGRAMS=blastp&QUERY=MDHTVLTDAEQDLWTTYLNDHATGATGALERLEMMAEEYTDLPHHDDLQTLTGQITQERERLLELISQLGAERSGLKTALAAAGEKVGRLKPNQHLGSRS PLSPLLEVELLRSGVVGKLSLWETLTLHADRLGLDRAEFEELTAAAARQAETLGTIHATWAASAFPAEAADQSD&LINK_LOC=protein&PAGE_TYPE=BlastSearch', 'BLAST this protein','Ksed_00530')" />

 
 
   AEYERTSARSREIAAGFSLDDRWTHPVVGEVTLRFVYLFLIEELARHTGHGDILREQLTAR', 'http://blast.ncbi.nlm.nih.gov/Blast.cgi?PAGE=Proteins&PROGRAM=blastp&BLAST_PROGRAMS=blastp&QUERY=MYALPEKTTGPERELLEAMLDRGRLALVENARGLSEADARRRLVPSLTTPIALVKHAAVAERRWFQWLIAGLDEAEIDGPSTPGDPSFVVTEEETVDDVI AEYERTSARSREIAAGFSLDDRWTHPVVGEVTLRFVYLFLIEELARHTGHGDILREQLTAR&LINK_LOC=protein&PAGE_TYPE=BlastSearch', 'BLAST this protein','AMED_5038')" />
   EHTSWEEDLATYQEQCEHSRRVAAVRSFDDCGVWRGKQVSMRSVYLHMIQEYA', 'http://blast.ncbi.nlm.nih.gov/Blast.cgi?PAGE=Proteins&PROGRAM=blastp&BLAST_PROGRAMS=blastp&QUERY=MADEPEPPARTRPTYLQDERELLDGWLEFHRSTLLAKCDGLSDDLRGARPVATSLLSLHGLVRHLAETERNWFSRILEAKPDLGRIWYDPAVEGSPLVPL EHTSWEEDLATYQEQCEHSRRVAAVRSFDDCGVWRGKQVSMRSVYLHMIQEYA&LINK_LOC=protein&PAGE_TYPE=BlastSearch', 'BLAST this protein','Franean1_3880')" />
   AEYERTSARSREIAAGFSLDDRWTHPVVGEVTLRFVYLFLIEELARHTGHGDILREQLTAR', 'http://blast.ncbi.nlm.nih.gov/Blast.cgi?PAGE=Proteins&PROGRAM=blastp&BLAST_PROGRAMS=blastp&QUERY=MYALPEKTTGPERELLEAMLDRGRLALVENARGLSEADARRRLVPSLTTPIALVKHAAVAERRWFQWLIAGLDEAEIDGPSTPGDPSFVVTEEETVDDVI AEYERTSARSREIAAGFSLDDRWTHPVVGEVTLRFVYLFLIEELARHTGHGDILREQLTAR&LINK_LOC=protein&PAGE_TYPE=BlastSearch', 'BLAST this protein','RAM_25650')" />
   GVHAADAEKDFVFFQAEVKACDVAAAGHDLDETFMSSYGVTLSLRWVYMLMIQEYARHNGHADFLRERTDGATGD', 'http://blast.ncbi.nlm.nih.gov/Blast.cgi?PAGE=Proteins&PROGRAM=blastp&BLAST_PROGRAMS=blastp&QUERY=MTWTAPSVKRTQQLGDLGTVTERQMLEGWLNWHRETLQAKCAGLEPAQLARTTVDPSDLTLLGLVRHMAEVERWWFRRSFAGEAIGDVFTGPSDGNEGLG GVHAADAEKDFVFFQAEVKACDVAAAGHDLDETFMSSYGVTLSLRWVYMLMIQEYARHNGHADFLRERTDGATGD&LINK_LOC=protein&PAGE_TYPE=BlastSearch', 'BLAST this protein','Sfla_6325')" />

 
 
   FGSADPKTSPAWIHDLARSAVGRVPALHDRDAGHRPVAELADLPEDVPAGTAAYVITTSGSTGIPKAVVASRANLASMVDGRNYDFDDGDLVTFSAFRLT WDGSLLKTLWALCTGGTSVLPDSRELMDAEAVAALARTWQTTHLVATPSFYRLLLPHLTPLRDRLRLVTLAGEALPGTLVEQHRAVLPGVPLSNEYGPTE TTVSCLAHPVRDVPASIAPIGRPLGASTAYVLDAKLVEVPYGAVGDLYVGGPQISEGYASRPAATAARFVADPFAARPGARMYHTGDLARVDPHGDIEFC GRFDGQVKVRGARVERHAVEAVLESHPAIHQAVVLATADEHGETVLTAFWVPAPAATVLPTPRDLIAHCAERLVAQAVPDRFLALGALPLAPSSKVDEAA LRRLLPSGGGGVA', 'http://blast.ncbi.nlm.nih.gov/Blast.cgi?PAGE=Proteins&PROGRAM=blastp&BLAST_PROGRAMS=blastp&QUERY=MTGILRQCARVPGRIAIVVGDRALTYRELDSASEALARQLAAVGVRPGQVVLIHQRQSVETVVGMIAALRLGAAWCVIEPGHPVGQLRALLGDIDCGAVV FGSADPKTSPAWIHDLARSAVGRVPALHDRDAGHRPVAELADLPEDVPAGTAAYVITTSGSTGIPKAVVASRANLASMVDGRNYDFDDGDLVTFSAFRLT WDGSLLKTLWALCTGGTSVLPDSRELMDAEAVAALARTWQTTHLVATPSFYRLLLPHLTPLRDRLRLVTLAGEALPGTLVEQHRAVLPGVPLSNEYGPTE TTVSCLAHPVRDVPASIAPIGRPLGASTAYVLDAKLVEVPYGAVGDLYVGGPQISEGYASRPAATAARFVADPFAARPGARMYHTGDLARVDPHGDIEFC GRFDGQVKVRGARVERHAVEAVLESHPAIHQAVVLATADEHGETVLTAFWVPAPAATVLPTPRDLIAHCAERLVAQAVPDRFLALGALPLAPSSKVDEAA LRRLLPSGGGGVA&LINK_LOC=protein&PAGE_TYPE=BlastSearch', 'BLAST this protein','RHA1_ro02211')" />
   LGDIDCGAIVFGSADPKTAPEAVEALARSAGGRVPALYDRDRGPAPEDEPAAELPGDVPAAAPAYVITTSGSTGTPKAVVASRANLASMVDGRDYDYEEG DLVTFSAFRLTWDGSLLKTLWALCTGGTSVLPAAHELMDAEAVAALARTWRATHLVATPSFYRLLLPHLTPLRERLRLVTLAGEALPGVLVAQHRAVLPG VPLSNEYGPTETTVSCLAHPVRDVPASIAPIGRPLGASTAYVLDAKLVEVPYGAVGDLYVGGPQISEGYASRPAATAARFVADPFAERPGARMYHTGDLA RVDPHGDIEFCGRFDGQVKVRGARVERHAVEAALESHPAIHQAVVLATADEHGETALTAFWVPAAAATLLPTPRDLIAHCAERLVAQAVPDRFLALGALP LAPSSKVDEAALRRLLPSGGGGVA', 'http://blast.ncbi.nlm.nih.gov/Blast.cgi?PAGE=Proteins&PROGRAM=blastp&BLAST_PROGRAMS=blastp&QUERY=MTDVVASGGVTSGILRQCRRVPGRTAIVIGDRSLTYRELDSVSEALARRLAALGVRPGQVVLIHQRQSVETVVAMIAALRLGAAWCVIEPGHPARQLRAL LGDIDCGAIVFGSADPKTAPEAVEALARSAGGRVPALYDRDRGPAPEDEPAAELPGDVPAAAPAYVITTSGSTGTPKAVVASRANLASMVDGRDYDYEEG DLVTFSAFRLTWDGSLLKTLWALCTGGTSVLPAAHELMDAEAVAALARTWRATHLVATPSFYRLLLPHLTPLRERLRLVTLAGEALPGVLVAQHRAVLPG VPLSNEYGPTETTVSCLAHPVRDVPASIAPIGRPLGASTAYVLDAKLVEVPYGAVGDLYVGGPQISEGYASRPAATAARFVADPFAERPGARMYHTGDLA RVDPHGDIEFCGRFDGQVKVRGARVERHAVEAALESHPAIHQAVVLATADEHGETALTAFWVPAAAATLLPTPRDLIAHCAERLVAQAVPDRFLALGALP LAPSSKVDEAALRRLLPSGGGGVA&LINK_LOC=protein&PAGE_TYPE=BlastSearch', 'BLAST this protein','ROP_19250')" />

 
 
   VARYLASELGCYVVMPDYRAAPQVCHPVAGEECYDAYLWVRGSGTAHGWDGDRVSVGGPSAGGHLSLGVALTAIERGAPSPAALSVEFAPVDLTLPNAQR TSPRSRPVVGKALMDLVMNTYFAGADTSLPLVSPALHPALDRLPPTLVITAEHDTLRDEADRFAGALERAGVEVVHRVFEGVDHGFIYHRPAGPARAAIS LIGEHLGSAFRRPR', 'http://blast.ncbi.nlm.nih.gov/Blast.cgi?PAGE=Proteins&PROGRAM=blastp&BLAST_PROGRAMS=blastp&QUERY=MKRDPRIAWRPALAARALQLTYPAMNRFLLLPPEVEFATKPVAAPATVRIPTRHGDIGALVFSPTSEDIESQLADGRSPPVHMLIHGGGFITRYPLGEGN VARYLASELGCYVVMPDYRAAPQVCHPVAGEECYDAYLWVRGSGTAHGWDGDRVSVGGPSAGGHLSLGVALTAIERGAPSPAALSVEFAPVDLTLPNAQR TSPRSRPVVGKALMDLVMNTYFAGADTSLPLVSPALHPALDRLPPTLVITAEHDTLRDEADRFAGALERAGVEVVHRVFEGVDHGFIYHRPAGPARAAIS LIGEHLGSAFRRPR&LINK_LOC=protein&PAGE_TYPE=BlastSearch', 'BLAST this protein','Ndas_1312')" />

 
 
   DADVAAYRRTVEINLISALTWVQAAWHGWLGEHGGSIVNVSSIAAELAVPDGTAYGTAKAALNHFTRQMANELAPAVRVNGVASGTVLTDFTRANLEGRE DKVIGSIPLGRLGEAADVGAAVAFLLSDEASWITGHTLVVDGGRLLHNR', 'http://blast.ncbi.nlm.nih.gov/Blast.cgi?PAGE=Proteins&PROGRAM=blastp&BLAST_PROGRAMS=blastp&QUERY=MASLKGKTALVTGGRTGLGFGTATALVERGAKVVITSRNEDALRKAAAELGEERAVAVAGDANDPEHQRIAVATAVDRFGSLDLLVNNVGGTERTARRLV DADVAAYRRTVEINLISALTWVQAAWHGWLGEHGGSIVNVSSIAAELAVPDGTAYGTAKAALNHFTRQMANELAPAVRVNGVASGTVLTDFTRANLEGRE DKVIGSIPLGRLGEAADVGAAVAFLLSDEASWITGHTLVVDGGRLLHNR&LINK_LOC=protein&PAGE_TYPE=BlastSearch', 'BLAST this protein','SACTE_5208')" />
   AVDRFGSLDLLVNNVGGTERTARRLVDVDLASYRRTVEINLMSALTWVQTAWHGWLGEHGGAIVNISSIAGELAVPDGTAYGTAKAALNHFTRQMANELA PAVRVNGVASGTVLTDFTRANVEGREDKVIGSIPLRRLGEPADVGAAVAFLLSDEASWITGHTLVVDGGRLLHNR', 'http://blast.ncbi.nlm.nih.gov/Blast.cgi?PAGE=Proteins&PROGRAM=blastp&BLAST_PROGRAMS=blastp&QUERY=MKRREKCRRLTRRNNRRRRSREGTQTVASLEGKTALVTGGRTGLGFGIATALVERGANVVITSRTEDELRKAAAALGEERALGVAGDARDPEHQRIAVET AVDRFGSLDLLVNNVGGTERTARRLVDVDLASYRRTVEINLMSALTWVQTAWHGWLGEHGGAIVNISSIAGELAVPDGTAYGTAKAALNHFTRQMANELA PAVRVNGVASGTVLTDFTRANVEGREDKVIGSIPLRRLGEPADVGAAVAFLLSDEASWITGHTLVVDGGRLLHNR&LINK_LOC=protein&PAGE_TYPE=BlastSearch', 'BLAST this protein','SAV_2370')" />

 
 
   RMLGLPFIAVMPASTSPEKIAQIEFQGGRCHLVDDPSKVVVEARWLAEDSGGRFMDQFTYAERATDWRGNNNIAESIYAQLALERHPVPAWIVVGAGTGG TSATIGRYARYRRLPTKLCVVDPENSAFYPAWQAGDWSVRTDRGSRIEGIGRPTVEASFLPSVVDRMVQVPDAASLAAMRAGSAVLGRRVGGSTGTNLWG AFGLIAQMRAAGRTGSVVTLICDPGDRYVDTYYSDEWVVAQGLDLAPHLATIDRFLTDGTWPTA', 'http://blast.ncbi.nlm.nih.gov/Blast.cgi?PAGE=Proteins&PROGRAM=blastp&BLAST_PROGRAMS=blastp&QUERY=MTHLDRCDEASRRWVTEAIATVEADANRSADTHLLPFPLPREWGIDLYLKDESVHPTGSLKHRLARSLFLYGLCNGWIGPDTTIVEASSGSTAVSEAYFA RMLGLPFIAVMPASTSPEKIAQIEFQGGRCHLVDDPSKVVVEARWLAEDSGGRFMDQFTYAERATDWRGNNNIAESIYAQLALERHPVPAWIVVGAGTGG TSATIGRYARYRRLPTKLCVVDPENSAFYPAWQAGDWSVRTDRGSRIEGIGRPTVEASFLPSVVDRMVQVPDAASLAAMRAGSAVLGRRVGGSTGTNLWG AFGLIAQMRAAGRTGSVVTLICDPGDRYVDTYYSDEWVVAQGLDLAPHLATIDRFLTDGTWPTA&LINK_LOC=protein&PAGE_TYPE=BlastSearch', 'BLAST this protein','Micau_0352')" />
   RMLGLPFIAVMPASTSPEKIAQIEFQGGRCHLVDDPSKVVVEARWLAEDSGGRFMDQFTYAERATDWRGNNNIAESIYAQLALERHPVPAWIVVGAGTGG TSATIGRYARYRRLPTKLCVVDPENSAFYPAWQAGDWSVRTDRGSRIEGIGRPTVEASFLPSVVDRMVQVPDAASLAAMRAGSAVLGRRVGGSTGTNLWG AFGLIAQMRAAGRTGSVVTLICDPGDRYVDTYYSDEWVAAQGLDLAPHLATIDRFLTDATWPAA', 'http://blast.ncbi.nlm.nih.gov/Blast.cgi?PAGE=Proteins&PROGRAM=blastp&BLAST_PROGRAMS=blastp&QUERY=MTHLDRCDEASRRWVTEAIATVEADANRSADTHLLPFPLPREWGIDLYLKDESVHPTGSLKHRLARSLFLYGLCNGWIGPDTTIVEASSGSTAVSEAYFA RMLGLPFIAVMPASTSPEKIAQIEFQGGRCHLVDDPSKVVVEARWLAEDSGGRFMDQFTYAERATDWRGNNNIAESIYAQLALERHPVPAWIVVGAGTGG TSATIGRYARYRRLPTKLCVVDPENSAFYPAWQAGDWSVRTDRGSRIEGIGRPTVEASFLPSVVDRMVQVPDAASLAAMRAGSAVLGRRVGGSTGTNLWG AFGLIAQMRAAGRTGSVVTLICDPGDRYVDTYYSDEWVAAQGLDLAPHLATIDRFLTDATWPAA&LINK_LOC=protein&PAGE_TYPE=BlastSearch', 'BLAST this protein','ML5_0328')" />
   RMLDLPFIAVMPASTSPEKIAQIEFQGGRCHLVDDPAKVVIEARWLAEDSGGHYMDQFTYAERATDWRGNNNIAESIYAQLSLERHPVPAWIVVGAGTGG TSATIGRYARYRRLPTKLCVVDPENSAFYPAWQAADWSVRTGQGSRIEGIGRPTVEPSFLPSVVDRMMRVPDAASLAAMRSASAVLGRQVGGSTGTNLWG AFGLIAELLAAGRTGSVVTLICDAGDRYADTYYSDEWVSAQGLDLAPHLATIDRFLTDGSWPS', 'http://blast.ncbi.nlm.nih.gov/Blast.cgi?PAGE=Proteins&PROGRAM=blastp&BLAST_PROGRAMS=blastp&QUERY=MTQLDRCDEASRRWVTEAIAAVEADANRSADTHLLPFPLPRRWGIDLYLKDESVHPTGSLKHRLARSLFLYGLCNGWIGPDTTIVEASSGSTAVSEAYFA RMLDLPFIAVMPASTSPEKIAQIEFQGGRCHLVDDPAKVVIEARWLAEDSGGHYMDQFTYAERATDWRGNNNIAESIYAQLSLERHPVPAWIVVGAGTGG TSATIGRYARYRRLPTKLCVVDPENSAFYPAWQAADWSVRTGQGSRIEGIGRPTVEPSFLPSVVDRMMRVPDAASLAAMRSASAVLGRQVGGSTGTNLWG AFGLIAELLAAGRTGSVVTLICDAGDRYADTYYSDEWVSAQGLDLAPHLATIDRFLTDGSWPS&LINK_LOC=protein&PAGE_TYPE=BlastSearch', 'BLAST this protein','VAB18032_05940')" />

 
 
   
   
   
   

 
 
   GDEMVAEAWHRAGSGRSGIYDVTVRVGDTVIAEFRGRSRTVPGLAAPPPAPA', 'http://blast.ncbi.nlm.nih.gov/Blast.cgi?PAGE=Proteins&PROGRAM=blastp&BLAST_PROGRAMS=blastp&QUERY=MPAPENSAADALRVARDAAAAMEHADVAAREAGVRLVDVGPGRAVTALTVTEKHLNGHGICHGGYVFLLADAAFAYACNSFGVSTVAAGADVAFLRPAAS GDEMVAEAWHRAGSGRSGIYDVTVRVGDTVIAEFRGRSRTVPGLAAPPPAPA&LINK_LOC=protein&PAGE_TYPE=BlastSearch', 'BLAST this protein','Psed_6637')" />

 
 
   HATDDADRVRALLTAPETSVITLTVTEKAHHRRADGRGLDLAAPEIAADLTSPAPSTVVGRLAAGLALRHRTTGAPVNVVSCDNIADNGAVLAGLVRDYA EAANRPGTAAFLAWLDTAVAFPSTVVDRIVPATTEDDRATAARALGLRDEATVAGEPYRQWVLQDAFTADRPAWERDGALLVPDVRPYQVTKLRLLNGSH SALAHLGAAAGCTTIREVLHTAWGERFVRAFCAEVAPTLPEGGPDPAGYADDLVTRFRNPAVRHLLRQIGSDSSLKVPERWLDPLRALDRAPLLELALAG WVASTRPGDDDLPVHGTTDPLSDELAACWHDRPAHPELVARLLRLVGAPDLAERTDLTTAVAQRLPALRAGRVEF', 'http://blast.ncbi.nlm.nih.gov/Blast.cgi?PAGE=Proteins&PROGRAM=blastp&BLAST_PROGRAMS=blastp&QUERY=MTPVDRLGLAALDRLPAHARPAADPRELPTRALHLGLGAFHRAHQAVYTEHAGGWGIAAIAPRSRDVVAALRAQDCLYSVVERSPERPSARVVGSITEAL HATDDADRVRALLTAPETSVITLTVTEKAHHRRADGRGLDLAAPEIAADLTSPAPSTVVGRLAAGLALRHRTTGAPVNVVSCDNIADNGAVLAGLVRDYA EAANRPGTAAFLAWLDTAVAFPSTVVDRIVPATTEDDRATAARALGLRDEATVAGEPYRQWVLQDAFTADRPAWERDGALLVPDVRPYQVTKLRLLNGSH SALAHLGAAAGCTTIREVLHTAWGERFVRAFCAEVAPTLPEGGPDPAGYADDLVTRFRNPAVRHLLRQIGSDSSLKVPERWLDPLRALDRAPLLELALAG WVASTRPGDDDLPVHGTTDPLSDELAACWHDRPAHPELVARLLRLVGAPDLAERTDLTTAVAQRLPALRAGRVEF&LINK_LOC=protein&PAGE_TYPE=BlastSearch', 'BLAST this protein','Amir_5059')" />
   VAEQDPESVVRAIADPATKIVTITVTEHGYTVSPETGSLDLDSDAVRRDLREVRTPRTTIGQIARGLALRASTHAAPVTILSCDNLLSNGRQTEKLVREF VGTFAPTLRDDLLSWMDSSVTFPNSMVDRIVPSGSDRYNETALAHLGVRDTIAVPAEPFTMWVMEERFAAGRPAWEHGGALFTDDVEPYELMKVRLLNGT HSLIACLGALDGRATIPESVAQPFVEAAARRILCDEYLPTVTVPESVDTDDYIRQLFSRWSNTALGHRTSQVGSDGSVKLAQRIPIPALEHLRRGTVPQY LSLTVAAYLCCIAPPPGFDPGPHARSMTDPARARLAGMADNAASTTEFVRSVFVDGGLFPSTLASHTEFVSRIADFVDIIVRHGPAVAAGEASDPVPDLR TITSLRSSATI', 'http://blast.ncbi.nlm.nih.gov/Blast.cgi?PAGE=Proteins&PROGRAM=blastp&BLAST_PROGRAMS=blastp&QUERY=MSEPTLSRRNVPAAALIHRRVPEGTGIVHLGLGNFHRAHQAVYTALAMEREPGPWGILGVASRSSTVADAMNAQDLLYGVVEISPDGSRVSVPGSHTGTL VAEQDPESVVRAIADPATKIVTITVTEHGYTVSPETGSLDLDSDAVRRDLREVRTPRTTIGQIARGLALRASTHAAPVTILSCDNLLSNGRQTEKLVREF VGTFAPTLRDDLLSWMDSSVTFPNSMVDRIVPSGSDRYNETALAHLGVRDTIAVPAEPFTMWVMEERFAAGRPAWEHGGALFTDDVEPYELMKVRLLNGT HSLIACLGALDGRATIPESVAQPFVEAAARRILCDEYLPTVTVPESVDTDDYIRQLFSRWSNTALGHRTSQVGSDGSVKLAQRIPIPALEHLRRGTVPQY LSLTVAAYLCCIAPPPGFDPGPHARSMTDPARARLAGMADNAASTTEFVRSVFVDGGLFPSTLASHTEFVSRIADFVDIIVRHGPAVAAGEASDPVPDLR TITSLRSSATI&LINK_LOC=protein&PAGE_TYPE=BlastSearch', 'BLAST this protein','RHA1_ro02788')" />
   VAEQDPESVIRAIADPATKIVTITVTEHGYTVSPETGSLDLDSDAVRRDLAEVRTPRTTIGQIARGLALRASTHAAPITILSCDNLLSNGRQTERLVREF VGTFAPPLRDDMLSWMTSSVTFPNSMVDRIVPSGADRYNEAALAHLGVRDTIAVPAEPFTMWVMEERFAAGRPVWEHGGALFTDDVEPYELMKVRLLNGT HSLIAYLGALDGRATIPESVAQPFVEAAARRILCDEYLPTVTVPESVDTVDYIRQLFSRWSNTALGHRTSQVGSDGSVKLAQRIPIPALEHLGRGTVPQY LSLTVAAYLCCIAPLPGFDPGPHARSMTDPARPRLAGMADNAASTTEFVRSVFVDGGLFPSTLASHTEFVSRIADFVDIIVRHGPAVAAAEASDPDLGTI TSLQSRATI', 'http://blast.ncbi.nlm.nih.gov/Blast.cgi?PAGE=Proteins&PROGRAM=blastp&BLAST_PROGRAMS=blastp&QUERY=MSEPTLSRRHVPSAALIHRRVPEGTGIVHLGLGNFHRAHQAVYTALAMEREPGPWGILGVASRSRAVADAMNAQDLLYSVVEISPEGSRVSVPGSHTGTL VAEQDPESVIRAIADPATKIVTITVTEHGYTVSPETGSLDLDSDAVRRDLAEVRTPRTTIGQIARGLALRASTHAAPITILSCDNLLSNGRQTERLVREF VGTFAPPLRDDMLSWMTSSVTFPNSMVDRIVPSGADRYNEAALAHLGVRDTIAVPAEPFTMWVMEERFAAGRPVWEHGGALFTDDVEPYELMKVRLLNGT HSLIAYLGALDGRATIPESVAQPFVEAAARRILCDEYLPTVTVPESVDTVDYIRQLFSRWSNTALGHRTSQVGSDGSVKLAQRIPIPALEHLGRGTVPQY LSLTVAAYLCCIAPLPGFDPGPHARSMTDPARPRLAGMADNAASTTEFVRSVFVDGGLFPSTLASHTEFVSRIADFVDIIVRHGPAVAAAEASDPDLGTI TSLQSRATI&LINK_LOC=protein&PAGE_TYPE=BlastSearch', 'BLAST this protein','ROP_24050')" />

 
 
   VHEARRQPRPPPVDRPVPRQLPAAPGRLFTGRSRELAELDAAVAGPGSLPVVALGGAGGTGKTWLALHWAHHNVDRFPDGQLYVDLRGFDPGGTPVSPGA ALRGFLDALGVRAPSFPTDPAAQVGLYRSLVAGLRVLVVLDNARDLAQVEPLVPGGASATVLVTSRHKLPGLVTTHGARSLRVDVLGPAASRDLLGRHLG AERVAAEPEAVDAVVRTCAGLPLALGLVAARASTEPDLPLRELARELGRSASSPLDALELDDTGLRSTFSWSYRDLPADAARALRLLGACHCPDLDAAAV AALTGGDLDGAGRALAVLGRVHLARRSPDGRFHTHDLVRAYAADLAAANDGPAAVDAALERLVEHLLGRVASAMDVVAPYERLHRPTGGGAPPGFGYPEA LAWLDAERHNLVALAARGTPEQTGRLSTSLHRYFVVGAHFADALAVHGHALDRAHTGEARSNALRHLGAVHRWLGDYDRALAYNEDAAALAGRLGDRRLE HAALNNIGIIHERTGRHAEALARYERVLRYAEETDDRFARGTVHHNLGSLYQRMADYPHALEHYREALAVAGSADDHNLRGFTHNDLGRLHVRLGQQARA REHLEQALALAVEHNDRSLQTEVLNALGELHHALGDVPRALGHHRDALVLAEGVGRADEIARAEQGILRCEGPRT', 'http://blast.ncbi.nlm.nih.gov/Blast.cgi?PAGE=Proteins&PROGRAM=blastp&BLAST_PROGRAMS=blastp&QUERY=MTGAPRRAGGVSPALVRTLSDLSRELELLRGRAARGSGKAKVSLAELAAQVGVPKSTMHTYVTGSTLAPADVLDRVVIALGATPAEQSLWGEAWFRVAES VHEARRQPRPPPVDRPVPRQLPAAPGRLFTGRSRELAELDAAVAGPGSLPVVALGGAGGTGKTWLALHWAHHNVDRFPDGQLYVDLRGFDPGGTPVSPGA ALRGFLDALGVRAPSFPTDPAAQVGLYRSLVAGLRVLVVLDNARDLAQVEPLVPGGASATVLVTSRHKLPGLVTTHGARSLRVDVLGPAASRDLLGRHLG AERVAAEPEAVDAVVRTCAGLPLALGLVAARASTEPDLPLRELARELGRSASSPLDALELDDTGLRSTFSWSYRDLPADAARALRLLGACHCPDLDAAAV AALTGGDLDGAGRALAVLGRVHLARRSPDGRFHTHDLVRAYAADLAAANDGPAAVDAALERLVEHLLGRVASAMDVVAPYERLHRPTGGGAPPGFGYPEA LAWLDAERHNLVALAARGTPEQTGRLSTSLHRYFVVGAHFADALAVHGHALDRAHTGEARSNALRHLGAVHRWLGDYDRALAYNEDAAALAGRLGDRRLE HAALNNIGIIHERTGRHAEALARYERVLRYAEETDDRFARGTVHHNLGSLYQRMADYPHALEHYREALAVAGSADDHNLRGFTHNDLGRLHVRLGQQARA REHLEQALALAVEHNDRSLQTEVLNALGELHHALGDVPRALGHHRDALVLAEGVGRADEIARAEQGILRCEGPRT&LINK_LOC=protein&PAGE_TYPE=BlastSearch', 'BLAST this protein','Amir_0976')" />

 
 
   TWWNVSLGIQVWHHLEHLLLLLQVLAGANLLGKAAPTSLIQLLIPRVELHLFYNTLVTVPMVVAMYLHTRANRPDNAAARCACAPKD', 'http://blast.ncbi.nlm.nih.gov/Blast.cgi?PAGE=Proteins&PROGRAM=blastp&BLAST_PROGRAMS=blastp&QUERY=MAISTGAPPRTLRGGLDSLNSRHHQLGLRLFLFIVIAHWAEHLVQAYQIYVMGWPIPEARGVLGMPFPWLVTSEWMHYGYALVMMAGLFLLRSGFTGRSR TWWNVSLGIQVWHHLEHLLLLLQVLAGANLLGKAAPTSLIQLLIPRVELHLFYNTLVTVPMVVAMYLHTRANRPDNAAARCACAPKD&LINK_LOC=protein&PAGE_TYPE=BlastSearch', 'BLAST this protein','SGR_615')" />

 
 
   EHLVDPLDTLKRLRPKLKPDGVVVASIPNIRYLPALGKVVFRRDFPQEDFGIFDRTHLRFFTRSSMVRMFEDAGFSMQRIKGINPFLGPFGALFVALSLG HFADGVFLQYACVATPAS', 'http://blast.ncbi.nlm.nih.gov/Blast.cgi?PAGE=Proteins&PROGRAM=blastp&BLAST_PROGRAMS=blastp&QUERY=MRILPETTSVTEYYGHKRPEMLRFLPADAQCVLELGCGEGVFGAIVKQSTGAEVWGLEYNEEAAQRAGELLDHVLAGDASTRIAELPDNYFDAVVCNDVL EHLVDPLDTLKRLRPKLKPDGVVVASIPNIRYLPALGKVVFRRDFPQEDFGIFDRTHLRFFTRSSMVRMFEDAGFSMQRIKGINPFLGPFGALFVALSLG HFADGVFLQYACVATPAS&LINK_LOC=protein&PAGE_TYPE=BlastSearch', 'BLAST this protein','MMAR_2339')" />
   ERLVDPTATLKQLRRKLTSEGVVVAAVPNIRFLPALSKVLFRKDFPQEDFGTFDRTYIRFFTRRSLVRLFKTSGFGVRRIEGINAWNGTIGVALAVLTLG YFADGRYLQYACIASPSASKPTS', 'http://blast.ncbi.nlm.nih.gov/Blast.cgi?PAGE=Proteins&PROGRAM=blastp&BLAST_PROGRAMS=blastp&QUERY=MHIARESKSVTEYYRPTQTEILRFVPTTAQRILDLHCGQGTLGATLKERTGAEVWGIESDAQTAQQASAAIDRVLVGTVAERIAELPDNHFDVIVCNDVL ERLVDPTATLKQLRRKLTSEGVVVAAVPNIRFLPALSKVLFRKDFPQEDFGTFDRTYIRFFTRRSLVRLFKTSGFGVRRIEGINAWNGTIGVALAVLTLG YFADGRYLQYACIASPSASKPTS&LINK_LOC=protein&PAGE_TYPE=BlastSearch', 'BLAST this protein','MMAR_2350')" />

 
 
   SSGFEVLAELIESKTGIAFPEYLRLGVFEPLGMESTALEGPAGHGAVSTVDDLARFAEELLEPRFLAAETLSAATAVQFPDLAGIVPGYGMQRPNEWGLG FEIRGNKAPHWTGSENSPATFGHFGQSGTFLWVDPNVRVATVVLTDRDFGDWAKPLWPELSDDILSEISDL', 'http://blast.ncbi.nlm.nih.gov/Blast.cgi?PAGE=Proteins&PROGRAM=blastp&BLAST_PROGRAMS=blastp&QUERY=MQSLEKLGSWPVPHVSAAVVSPRGTLASTGPTDRVFKLASVTKLLVAYGSLIAIEEGAIGLDEEAGPEGSTVRHLLSHASGLAFGEKRTQAKLATKRIYS SSGFEVLAELIESKTGIAFPEYLRLGVFEPLGMESTALEGPAGHGAVSTVDDLARFAEELLEPRFLAAETLSAATAVQFPDLAGIVPGYGMQRPNEWGLG FEIRGNKAPHWTGSENSPATFGHFGQSGTFLWVDPNVRVATVVLTDRDFGDWAKPLWPELSDDILSEISDL&LINK_LOC=protein&PAGE_TYPE=BlastSearch', 'BLAST this protein','AS9A_1220')" />
   SAGFEVLAETVEAATEIAFGDYLAAAVFEPLGMASSELAGPAGHGARSTVADLGAFAGELLDPRLVSSQTAHAARTVQFPGLDGFVPGYGRHRPNDWGLG FEIRAHKSPHWTGSTNSPATFGHFGQSGTFLWVDPELAAACVVLTDRDFGAWAKPLWSEFNDSVVSALSD', 'http://blast.ncbi.nlm.nih.gov/Blast.cgi?PAGE=Proteins&PROGRAM=blastp&BLAST_PROGRAMS=blastp&QUERY=MSVLESLAAWPVDHVSAAVITTDGVAETFGDPDRVYPLASVTKLLVAEAILVAVEEGAVELDEEAGPPGATIAHLLAHASGLAFATRDVEAGVGEKRIYS SAGFEVLAETVEAATEIAFGDYLAAAVFEPLGMASSELAGPAGHGARSTVADLGAFAGELLDPRLVSSQTAHAARTVQFPGLDGFVPGYGRHRPNDWGLG FEIRAHKSPHWTGSTNSPATFGHFGQSGTFLWVDPELAAACVVLTDRDFGAWAKPLWSEFNDSVVSALSD&LINK_LOC=protein&PAGE_TYPE=BlastSearch', 'BLAST this protein','Gbro_3127')" />
   SAGFEVLADFVEAETGIAFPDYVAEAVFQPLGMATAALVGPAGHGAHAGIDDLSRFAAELLRPTLVSPQMLAEATAVQFPGLAGLLPGYGPQRPNDWGLG FEIKNGKSPHWTGAGNSPATYGHFGQSGTFLWVDPALDAACVVLTDRAFGDWAKPLWTDLSDAVATELRGRGR', 'http://blast.ncbi.nlm.nih.gov/Blast.cgi?PAGE=Proteins&PROGRAM=blastp&BLAST_PROGRAMS=blastp&QUERY=MQTLDRIGQWPVDNASAAVVDGSRGVAHFGDGQRIYPLASVTKLLCAYATLVAVEEGAVELDQPAGPPGATVRHLLAHASGLAFGENQVQAEPGAKRIYS SAGFEVLADFVEAETGIAFPDYVAEAVFQPLGMATAALVGPAGHGAHAGIDDLSRFAAELLRPTLVSPQMLAEATAVQFPGLAGLLPGYGPQRPNDWGLG FEIKNGKSPHWTGAGNSPATYGHFGQSGTFLWVDPALDAACVVLTDRAFGDWAKPLWTDLSDAVATELRGRGR&LINK_LOC=protein&PAGE_TYPE=BlastSearch', 'BLAST this protein','REQ_29000')" />
   PVGSKRIYSSAGFEVLAELVSRETEIEFADYLADAVFAPLGMISAALVGPAGHGAQASADDLAKFAAELLSPTLISTQTFAEATTVQFPGLNGILPGYGS QRPNGWGLGFEIRGDKRPHWTGLENSAATFGHFGQSGTFLWVDPTIDTACVVLTDRDFGDWAKPLWTELSDGVVGEVSR', 'http://blast.ncbi.nlm.nih.gov/Blast.cgi?PAGE=Proteins&PROGRAM=blastp&BLAST_PROGRAMS=blastp&QUERY=MPSTHSLDVIQQWPVDNAAAVVLSGKNPGEGDRIRGFSGDQSHVFPLASVTKLLCAYAILVAVEEGAVELDGPAGPEGSTVRHLLAHASGLAFAERVVQA PVGSKRIYSSAGFEVLAELVSRETEIEFADYLADAVFAPLGMISAALVGPAGHGAQASADDLAKFAAELLSPTLISTQTFAEATTVQFPGLNGILPGYGS QRPNGWGLGFEIRGDKRPHWTGLENSAATFGHFGQSGTFLWVDPTIDTACVVLTDRDFGDWAKPLWTELSDGVVGEVSR&LINK_LOC=protein&PAGE_TYPE=BlastSearch', 'BLAST this protein','RER_36770')" />
   SSAGYEVLADFLTAETSIDFADYVAESVFAPLSMGASALVGPAGHGAQASAGDLGRFAVELFRPALISPQTFADATSVQFPGLDGILPGYGSQRPNDWGL GFEIRSGKSPHWTGTGNSPRTFGHFGQSGTFLWVDPAAGLACVALTDRDFGDWAKPLWTELSDGILSERRRR', 'http://blast.ncbi.nlm.nih.gov/Blast.cgi?PAGE=Proteins&PROGRAM=blastp&BLAST_PROGRAMS=blastp&QUERY=MQSLDQIARWPVDNAAAVVLSYDEGVIGEYGDQQRVFPLASVTKLLCAYAVLVATEEGAVELDQPAGPEGSTVRHLLAHASGLAFDANRVQTAPERKRIY SSAGYEVLADFLTAETSIDFADYVAESVFAPLSMGASALVGPAGHGAQASAGDLGRFAVELFRPALISPQTFADATSVQFPGLDGILPGYGSQRPNDWGL GFEIRSGKSPHWTGTGNSPRTFGHFGQSGTFLWVDPAAGLACVALTDRDFGDWAKPLWTELSDGILSERRRR&LINK_LOC=protein&PAGE_TYPE=BlastSearch', 'BLAST this protein','RHA1_ro01204')" />
   SSAGYEVLADFLTAETSIDFADYVAESVFAPLSMSASALVGPAGHGARASAGDLGRFAAELFRPALISSQTFADATSVQFPGLDGILPGYGSQRPNDWGL GFEIRSGKSPHWTGTGNSPQTFGHFGQSGTFLWVDPAAGLACVALTDRDFGDWAKPVWTGLSDGILSERER', 'http://blast.ncbi.nlm.nih.gov/Blast.cgi?PAGE=Proteins&PROGRAM=blastp&BLAST_PROGRAMS=blastp&QUERY=MQSLDQIARWPVDNAAAVVLSRDDGVIGEYGDQQRVFPLASVTKLLCAYAVLVATEEGAVELDQPAGPEGSTVRHLLAHASGLAFDTDRVQTAPGRKRIY SSAGYEVLADFLTAETSIDFADYVAESVFAPLSMSASALVGPAGHGARASAGDLGRFAAELFRPALISSQTFADATSVQFPGLDGILPGYGSQRPNDWGL GFEIRSGKSPHWTGTGNSPQTFGHFGQSGTFLWVDPAAGLACVALTDRDFGDWAKPVWTGLSDGILSERER&LINK_LOC=protein&PAGE_TYPE=BlastSearch', 'BLAST this protein','ROP_09270')" />
   LYSNAGFEVLGDHIAKATDIPFAEYLRQAVLEPLGMTRTSLDGSPARDGVSTVADLVRFAAEVQAPRLLDPRTVAEAMTVQYPGTKGVLPGYGHQNPNDW GLGFEIRDAKSPHWTGTSSSPGTFGHFGQSGTFLWIDPVARVACAALTDRAFGPWAAEAWTPFTDAVLTELRAGRA', 'http://blast.ncbi.nlm.nih.gov/Blast.cgi?PAGE=Proteins&PROGRAM=blastp&BLAST_PROGRAMS=blastp&QUERY=MSLQSLALIENWPVSTAAAAVVRADGTVLAAHGPVDHRFPLASVTKPLAAYAVLVAYEEGAVELDEPAGPPGSTVRHLLAHTSGLAFDEHRVTSAPGERR LYSNAGFEVLGDHIAKATDIPFAEYLRQAVLEPLGMTRTSLDGSPARDGVSTVADLVRFAAEVQAPRLLDPRTVAEAMTVQYPGTKGVLPGYGHQNPNDW GLGFEIRDAKSPHWTGTSSSPGTFGHFGQSGTFLWIDPVARVACAALTDRAFGPWAAEAWTPFTDAVLTELRAGRA&LINK_LOC=protein&PAGE_TYPE=BlastSearch', 'BLAST this protein','SAV_5791')" />
   SNAGVEMLAEQVARETGIGFHEYTAEAVFDPLGMRDVSFTGSAAWGAGAHCRDLARFAGELLAPRLLAPELLVEATNVQFTGLDGVLVGYGNMKPNDWGL GFELRGAKSPHWTGAGNSPKTFGHFGASGTFLWVDPNAQLACVVLTDRPFQDGGWAKPLWPGFSDAVLAEFAGTSVPA', 'http://blast.ncbi.nlm.nih.gov/Blast.cgi?PAGE=Proteins&PROGRAM=blastp&BLAST_PROGRAMS=blastp&QUERY=MTTALSLLDAWPVDHVAATVIAPSGVVAEHGEVERVFPLASVTKILSGYAALLAVQEGALGLDDPAGPPGSTLRHLLAHASGLPFDSRVPFARPGSRRIY SNAGVEMLAEQVARETGIGFHEYTAEAVFDPLGMRDVSFTGSAAWGAGAHCRDLARFAGELLAPRLLAPELLVEATNVQFTGLDGVLVGYGNMKPNDWGL GFELRGAKSPHWTGAGNSPKTFGHFGASGTFLWVDPNAQLACVVLTDRPFQDGGWAKPLWPGFSDAVLAEFAGTSVPA&LINK_LOC=protein&PAGE_TYPE=BlastSearch', 'BLAST this protein','Srot_2741')" />
   EVLGDHIAKATDIPFPEYLRQAVLEPLGMTATELAGSPAKDGVSTVDDLVRFAAELQAPRLLAAETLAEASSVVHPGLTGVLPGYGHQRPNDWGLGFEIR DGKSPHWTGASSSPRTFGHFGQSGTFLWVDPDARAACVALADRAFGPWAVEAWPTLTDAILAELG', 'http://blast.ncbi.nlm.nih.gov/Blast.cgi?PAGE=Proteins&PROGRAM=blastp&BLAST_PROGRAMS=blastp&QUERY=MIENWPVPTAAAAVVRADGTLAGSYGPTGHRFPLASVTKPLAAYAALLAVEEGAVELDEPAGPEGSTVRHLLAHTSGLAFDEHRSVAAPGNRRLYSNAGF EVLGDHIAKATDIPFPEYLRQAVLEPLGMTATELAGSPAKDGVSTVDDLVRFAAELQAPRLLAAETLAEASSVVHPGLTGVLPGYGHQRPNDWGLGFEIR DGKSPHWTGASSSPRTFGHFGQSGTFLWVDPDARAACVALADRAFGPWAVEAWPTLTDAILAELG&LINK_LOC=protein&PAGE_TYPE=BlastSearch', 'BLAST this protein','Strvi_7538')" />
   SSYGYELVARLVEEETGIGFPDYLREAVFGPLGMTRSSLPGPAGHGARSTVADLTRFAAEVAAPTLLDPRTVGGACTVHFPGLAGFVPGYGKFIQNTWGL GFEIKGDKRPHWTGTRNSPRTVGHFGQAGTYLWIDPDLQLAAVILTDRPFGAWAKPLWSEFNDRLISQELQGV', 'http://blast.ncbi.nlm.nih.gov/Blast.cgi?PAGE=Proteins&PROGRAM=blastp&BLAST_PROGRAMS=blastp&QUERY=MSLAELAQWPVENAAGALLRTDDARTVDGYGDLDAEYELASVTKLLVAYGILVAIEEEAIRLDQPVGPPGSTVEHLLAHASGLAFDSDEVVAAPATQRIY SSYGYELVARLVEEETGIGFPDYLREAVFGPLGMTRSSLPGPAGHGARSTVADLTRFAAEVAAPTLLDPRTVGGACTVHFPGLAGFVPGYGKFIQNTWGL GFEIKGDKRPHWTGTRNSPRTVGHFGQAGTYLWIDPDLQLAAVILTDRPFGAWAKPLWSEFNDRLISQELQGV&LINK_LOC=protein&PAGE_TYPE=BlastSearch', 'BLAST this protein','Tpau_2729')" />

 
 
   AHAGRDTGSGGETDDGSRRHTPTASPTAVGPNHLADPVVRSNGEIDPGSNPYWAQSDVILKTGKPLTSLTVELRVAHTGGVLTTGSWSTLPTDDMKVSVR SEKNALVYRWTLRDGATVPAGRHVFAGQYNHAEGDRGIAEDSYSASGNGPSGAFAVRGAFREAK', 'http://blast.ncbi.nlm.nih.gov/Blast.cgi?PAGE=Proteins&PROGRAM=blastp&BLAST_PROGRAMS=blastp&QUERY=MIDHRERHDRIDVAAELHTAAESHRPDRARMLARVTAGMAGDERRHTGRGAGAGRPWTRVAGPVAGLAAAVAAAGIVVLATEPAQRPQTVRTSSEPAVPP AHAGRDTGSGGETDDGSRRHTPTASPTAVGPNHLADPVVRSNGEIDPGSNPYWAQSDVILKTGKPLTSLTVELRVAHTGGVLTTGSWSTLPTDDMKVSVR SEKNALVYRWTLRDGATVPAGRHVFAGQYNHAEGDRGIAEDSYSASGNGPSGAFAVRGAFREAK&LINK_LOC=protein&PAGE_TYPE=BlastSearch', 'BLAST this protein','SBI_08134')" />
   PPAGAGADRRPGEEPGAGSQSDAERHTPSAYPTAAGPNHLAEPTVRSKGAINTHSNPYWAQSDITLTIGRPLTSLTVELRVADTGDVLSTGSWSSLPTGD VASATRVEDGVLVYRWTLRKGATVRAGRYVFAGQYNHAEGDRDTGRDAYSASGNGASGAFAVRGDFPRFTVGD', 'http://blast.ncbi.nlm.nih.gov/Blast.cgi?PAGE=Proteins&PROGRAM=blastp&BLAST_PROGRAMS=blastp&QUERY=MIEHRERLEGRDVVAELRTAAASHEPDRIRMLARVTAGMAADGRDRSRGARTSPRRPWAPRLVGPVAGLAAAVAATGIAVVATDGAERPQTVRTSSEPAA PPAGAGADRRPGEEPGAGSQSDAERHTPSAYPTAAGPNHLAEPTVRSKGAINTHSNPYWAQSDITLTIGRPLTSLTVELRVADTGDVLSTGSWSSLPTGD VASATRVEDGVLVYRWTLRKGATVRAGRYVFAGQYNHAEGDRDTGRDAYSASGNGASGAFAVRGDFPRFTVGD&LINK_LOC=protein&PAGE_TYPE=BlastSearch', 'BLAST this protein','Strvi_6955')" />

 
 
   EGRYYFAVTIPADFSADLVSAGGDAPRSARIDVTYDDANSFLVTSLGRTAMERIRAAVSATAGEQAVDAVLVGLTQAHDGLAQASDGAVQLRGATHELAT GATTLADGAHALRDGAGRAADGAADLEDGTAQVRDGAATTADAAGRLADGSAQVADGVHAAVAQVDALAASAEQVPAQLGSLTAYLTARAQGGDAQAAQI LSGLAATASSLPDAATLDAATQSLHELDAGARGVADGSAALRDGAAQLAAGAADVRDGAAALADGTADVAAGATDLSDGADRLTDGTGGIEEGASTLADA LTQGTAAVPSDPDGTREARAAAIAEPVALEATDRAAAEGFGEGIAPFFLPLALFLGGVVTWMLLRPVPPRALATPARGARAALAGFAPAFVMGVAQVVAL LTVLRVGLGLTPSHPLGALAFTVLVVAAFLALQQMLLALLGTAAGRVATLALLVLQLASAGGTYPVETSPAFFRALHPLLPMSYGVDGLRALLTGNPDGR LWTAVAYLVTLLVGSLAVTSWRAGRMRTWTLSRLHPALTL', 'http://blast.ncbi.nlm.nih.gov/Blast.cgi?PAGE=Proteins&PROGRAM=blastp&BLAST_PROGRAMS=blastp&QUERY=MLSLTAGTELRRFRRGTLPRLAVVAMIVVPLLYGVLYLWAFWNPTGHLDRIPVALVDADAGAVRDGTPVDAGGDLVERLVDEKRLDWVVTDAADAAAGVD EGRYYFAVTIPADFSADLVSAGGDAPRSARIDVTYDDANSFLVTSLGRTAMERIRAAVSATAGEQAVDAVLVGLTQAHDGLAQASDGAVQLRGATHELAT GATTLADGAHALRDGAGRAADGAADLEDGTAQVRDGAATTADAAGRLADGSAQVADGVHAAVAQVDALAASAEQVPAQLGSLTAYLTARAQGGDAQAAQI LSGLAATASSLPDAATLDAATQSLHELDAGARGVADGSAALRDGAAQLAAGAADVRDGAAALADGTADVAAGATDLSDGADRLTDGTGGIEEGASTLADA LTQGTAAVPSDPDGTREARAAAIAEPVALEATDRAAAEGFGEGIAPFFLPLALFLGGVVTWMLLRPVPPRALATPARGARAALAGFAPAFVMGVAQVVAL LTVLRVGLGLTPSHPLGALAFTVLVVAAFLALQQMLLALLGTAAGRVATLALLVLQLASAGGTYPVETSPAFFRALHPLLPMSYGVDGLRALLTGNPDGR LWTAVAYLVTLLVGSLAVTSWRAGRMRTWTLSRLHPALTL&LINK_LOC=protein&PAGE_TYPE=BlastSearch', 'BLAST this protein','Celf_2273')" />
   DHGKYYFMLELPPDFSEAVASPVTGQSKKAELIAVYNDANNYISSNIGRTAVDQVLTAVSTRISGQAVNQVLSVVLSSGSGIKQAADGARLLADGAAQVD DGAAQLSAAIDKATDPILAVGKAASAVGGDTEQLQQAAVALRQANAQIGGIAKAQDGAAASLAGVIDQLSASDDPAARSSAETLRGVQNQLHEHQFTPQI RQQLTDGENAAISLTETLRSPGSPLRSALDQLGGKGRDLTDKLAQLRAGAERLKSGTAQVRGGSSELAAKLADGAGQVPNWTPQQKDAIADTIGGPVHLR ASHLNAAPNFGTGMAPFFLTLALFFGAIVLWMVFRPLQNRAIAAQAPAFRVALASYLPAALIGISQAVVLYCVVRFGLGMRAAHPVAMLAFMALVSFAFV ALTQSVNALLGPAVGRVLVMALLMVQLVSAGGMYPVETTSKPFQALHRYDPMTYGVNGLRQLVLGGVDSRLWQAVLTLAAIMAGALAVSSVCARRNRLWN ASRLIPAIKM', 'http://blast.ncbi.nlm.nih.gov/Blast.cgi?PAGE=Proteins&PROGRAM=blastp&BLAST_PROGRAMS=blastp&QUERY=MLAGLAFGSEIKRFGRSRLTRAAIVVLALLPLVYGALYLWAYWDPFGHVNKMPVALVNADRGAVVSGQRINVGDEIAKSLTQDGGLEWHVMSHEQARGGV DHGKYYFMLELPPDFSEAVASPVTGQSKKAELIAVYNDANNYISSNIGRTAVDQVLTAVSTRISGQAVNQVLSVVLSSGSGIKQAADGARLLADGAAQVD DGAAQLSAAIDKATDPILAVGKAASAVGGDTEQLQQAAVALRQANAQIGGIAKAQDGAAASLAGVIDQLSASDDPAARSSAETLRGVQNQLHEHQFTPQI RQQLTDGENAAISLTETLRSPGSPLRSALDQLGGKGRDLTDKLAQLRAGAERLKSGTAQVRGGSSELAAKLADGAGQVPNWTPQQKDAIADTIGGPVHLR ASHLNAAPNFGTGMAPFFLTLALFFGAIVLWMVFRPLQNRAIAAQAPAFRVALASYLPAALIGISQAVVLYCVVRFGLGMRAAHPVAMLAFMALVSFAFV ALTQSVNALLGPAVGRVLVMALLMVQLVSAGGMYPVETTSKPFQALHRYDPMTYGVNGLRQLVLGGVDSRLWQAVLTLAAIMAGALAVSSVCARRNRLWN ASRLIPAIKM&LINK_LOC=protein&PAGE_TYPE=BlastSearch', 'BLAST this protein','Srot_0304')" />

 
 
   FLGSWAAWRKAAPDRMFVLNTPMLERNEDHVPDDEVRSLLRRGAGGEFDAHFRKLAERLVLLGVPDTVLVLGWEMNGTTYTHRCGPDPAAWKAYWTRIVT AMRSVPGQEFRFDFAPSRGRDAVPWTECYPGDDVVDIIGMDSYDQPPARTFEEQVNEPYGLRKQVDFAAEHGKPISFPEWGLFRNGDNPRYMRGMLEWME RHKPVYQTITDYCPHGVWQCAENPESSRVYREMLSAGPDDGGPSAPAPTPTPETTRTSEAPVPDEPEPETGTEAPGWCVSLPLSDLFGPWARDHEFCAGS ', 'http://blast.ncbi.nlm.nih.gov/Blast.cgi?PAGE=Proteins&PROGRAM=blastp&BLAST_PROGRAMS=blastp&QUERY=MPAQRRLINFGIGTAVLGLLVGGVLVADRNGADSAAGKGADASLPPVAQGPTAVGAYLDYGPQGVRRMAELSRWLGGTELRVGHSYLPGDVWENIEGRPG FLGSWAAWRKAAPDRMFVLNTPMLERNEDHVPDDEVRSLLRRGAGGEFDAHFRKLAERLVLLGVPDTVLVLGWEMNGTTYTHRCGPDPAAWKAYWTRIVT AMRSVPGQEFRFDFAPSRGRDAVPWTECYPGDDVVDIIGMDSYDQPPARTFEEQVNEPYGLRKQVDFAAEHGKPISFPEWGLFRNGDNPRYMRGMLEWME RHKPVYQTITDYCPHGVWQCAENPESSRVYREMLSAGPDDGGPSAPAPTPTPETTRTSEAPVPDEPEPETGTEAPGWCVSLPLSDLFGPWARDHEFCAGS &LINK_LOC=protein&PAGE_TYPE=BlastSearch', 'BLAST this protein','SACTE_0311')" />
   LASWAAWRKGADDRMLVLNTPMLERNEERLSDEEVRSLLREGAQGRFDEHFRRLAERLVLLGVPDTVIVLGWEMNGTTYTHRCGPDPVAWKAYWNRIVTA MRSVSGQKFRFDFAPSRGLDAVPWTDCYPGDDVVDIIGMDSYDQPPALTFDEQVSEPYGLQKQVDFAAEHGKPISFPEWGLFRNGDNPEYMRRMLEWMDL HKPLYQTITDYCPHGVWQCADNPDSSKVFREMLSGLPDAPGPSAPAAEPSAPLTTPVPEEPAPTVPEPDVNDPAWCFTVPLGDWFGQWLADREICARG', 'http://blast.ncbi.nlm.nih.gov/Blast.cgi?PAGE=Proteins&PROGRAM=blastp&BLAST_PROGRAMS=blastp&QUERY=MPVQRRLTTFGIGATALCLLVGGAFLADHDAADTQTGKGAAQGAQEPVGTTAVGAYLDYGPQGVQRMGELSRWLGGTELRVGHTYLPGDLWENIEGRPGF LASWAAWRKGADDRMLVLNTPMLERNEERLSDEEVRSLLREGAQGRFDEHFRRLAERLVLLGVPDTVIVLGWEMNGTTYTHRCGPDPVAWKAYWNRIVTA MRSVSGQKFRFDFAPSRGLDAVPWTDCYPGDDVVDIIGMDSYDQPPALTFDEQVSEPYGLQKQVDFAAEHGKPISFPEWGLFRNGDNPEYMRRMLEWMDL HKPLYQTITDYCPHGVWQCADNPDSSKVFREMLSGLPDAPGPSAPAAEPSAPLTTPVPEEPAPTVPEPDVNDPAWCFTVPLGDWFGQWLADREICARG&LINK_LOC=protein&PAGE_TYPE=BlastSearch', 'BLAST this protein','Sfla_5960')" />
   DAWADWRQADPARMFVLNVPMLERNEERVPDAEVRTLLRAGAAGEYDQHFSRLAERLVELGVPDTVIVLGWEMNGTTYTHRCGPDPASWKAYWQRIVTAM RAVPGQDFRFDFTPSRGRDAVPWTECYPGDDVVDIIGMDSYDQPPGETFDDQVTDPYGLQKHVDFAAEHGKPISFPEWGLFRNGDNPEYMRRMLEWIDRH KPLYQTVTDYCPHGVWQCKSNPRSSRVFRTMLTEMAAPAAPTPSPAPTPTPTPPPTAAPTVPAPSPTVEPSVAPSPAPPAPTEPAAGAPTVREWCVPVPF GDWLGPWLREREICFRY', 'http://blast.ncbi.nlm.nih.gov/Blast.cgi?PAGE=Proteins&PROGRAM=blastp&BLAST_PROGRAMS=blastp&QUERY=MPAKRRLIVSCIAAAALSLLAGGGIAGQSPPGADSVDGAAPGAPRAASSTAHGAYLDYGPAGVSRMAELSRWLGGAELRVGHTYLPGDLWVNIEGAPDFL DAWADWRQADPARMFVLNVPMLERNEERVPDAEVRTLLRAGAAGEYDQHFSRLAERLVELGVPDTVIVLGWEMNGTTYTHRCGPDPASWKAYWQRIVTAM RAVPGQDFRFDFTPSRGRDAVPWTECYPGDDVVDIIGMDSYDQPPGETFDDQVTDPYGLQKHVDFAAEHGKPISFPEWGLFRNGDNPEYMRRMLEWIDRH KPLYQTVTDYCPHGVWQCKSNPRSSRVFRTMLTEMAAPAAPTPSPAPTPTPTPPPTAAPTVPAPSPTVEPSVAPSPAPPAPTEPAAGAPTVREWCVPVPF GDWLGPWLREREICFRY&LINK_LOC=protein&PAGE_TYPE=BlastSearch', 'BLAST this protein','SGR_530')" />

 
 
   AEFDFADVFTLSDDALVLSQRRFHYLAELAG', 'http://blast.ncbi.nlm.nih.gov/Blast.cgi?PAGE=Proteins&PROGRAM=blastp&BLAST_PROGRAMS=blastp&QUERY=MVGDLPSLTASAATTPCGVDHVRLLYRYLDTGEFDGYASLLDENVQIRGVGAAPAYGREAAAEAARAAPPALHELYKIIAAADSVVVTGRRLARDPGTGR AEFDFADVFTLSDDALVLSQRRFHYLAELAG&LINK_LOC=protein&PAGE_TYPE=BlastSearch', 'BLAST this protein','AMED_4808')" />
   AEFDFADVFTLSDDALVLSQRRFHYLAELAG', 'http://blast.ncbi.nlm.nih.gov/Blast.cgi?PAGE=Proteins&PROGRAM=blastp&BLAST_PROGRAMS=blastp&QUERY=MVGDLPSLTASAATTPCGVDHVRLLYRYLDTGEFDGYASLLDENVQIRGVGAAPAYGREAAAEAARAAPPALHELYKIIAAADSVVVTGRRLARDPGTGR AEFDFADVFTLSDDALVLSQRRFHYLAELAG&LINK_LOC=protein&PAGE_TYPE=BlastSearch', 'BLAST this protein','RAM_24475')" />

 
 
   ARGFDHESPMSPYVDDGPWPGVLDEVPAVFRRYVDDPLFTDESGMPVVTACLWRTGDDDRWRAGAIDFPEDGEDSDGADWLFQLLVTDAPESYQEWAEDH FEVSVDLEAVRHVLALRPLTDEVIAALAPERVPAELAADIAEIGYPVGPGE', 'http://blast.ncbi.nlm.nih.gov/Blast.cgi?PAGE=Proteins&PROGRAM=blastp&BLAST_PROGRAMS=blastp&QUERY=MSRRMWSRVPRGCGTTCGGLRRVRLDVPMSVHDVARRLPDIPTLIDRCRALAMLDAILCPEWDHRWHGYDARWSPTEAMASMRDGSGGEYSVVFAEAGAY ARGFDHESPMSPYVDDGPWPGVLDEVPAVFRRYVDDPLFTDESGMPVVTACLWRTGDDDRWRAGAIDFPEDGEDSDGADWLFQLLVTDAPESYQEWAEDH FEVSVDLEAVRHVLALRPLTDEVIAALAPERVPAELAADIAEIGYPVGPGE&LINK_LOC=protein&PAGE_TYPE=BlastSearch', 'BLAST this protein','SGR_1349')" />

 
 
   AEPAAYLSWLKKLAREHRVYKHPYYREFIRDEATSADLRTYVMQESVVDGRFDDLLAMMQVGTSGAAKMEIAQNFWDEMGNGKPEEVHTHLFNKIFEVFE IPADELERSLTANALLSGNLAVLLCRYRQYYPEAVGFLGMTEWLVPDRFVQVVHAWERLGLPDIGIVYHRLHITVDSQHAAGWFHNVVIPAAESEHMRRA IARGTLWRLNSSARYLDERMPAVAV', 'http://blast.ncbi.nlm.nih.gov/Blast.cgi?PAGE=Proteins&PROGRAM=blastp&BLAST_PROGRAMS=blastp&QUERY=MSSWLPRNPAELDKAVTGAERDAILDAIRSLNDDARLGDSEAFYAQQRLLARIYGLHTLIPEGPSAEGSVLLHTITRLLEEATIEAEDGYIEPGLIDQAP AEPAAYLSWLKKLAREHRVYKHPYYREFIRDEATSADLRTYVMQESVVDGRFDDLLAMMQVGTSGAAKMEIAQNFWDEMGNGKPEEVHTHLFNKIFEVFE IPADELERSLTANALLSGNLAVLLCRYRQYYPEAVGFLGMTEWLVPDRFVQVVHAWERLGLPDIGIVYHRLHITVDSQHAAGWFHNVVIPAAESEHMRRA IARGTLWRLNSSARYLDERMPAVAV&LINK_LOC=protein&PAGE_TYPE=BlastSearch', 'BLAST this protein','SAV_3163')" />
   LENAMMADLRGKMDQSALASVPQDPETFLPWYRNFISSHNASNHPFYRDFLEDRASAEDIRFYLAQETSLDPRFDDILSFLTVGTNGSEKMELVSNLWDE MGNGNSADVHTAVFAKTLTDAGVSKEFIDSNIMLESLVCGNVSAALALSRRHCYKAFGYFGVTEYLTPRRFRSYIVGCKRLGMPKSAYIYHDQHIQIDAR HGPSWFKNILLPSIAREPRCASEIVLGTVMRLETSTGYLDALQAKLELAR', 'http://blast.ncbi.nlm.nih.gov/Blast.cgi?PAGE=Proteins&PROGRAM=blastp&BLAST_PROGRAMS=blastp&QUERY=MSTTPELHSGTDLRELVISLLAGDEDAATVNARATDMVYRAEIRTVAENLAHAAFIRGDRAALHTAHWVLGDIYDRCFSLPPIDQVDSLLTEILDDIRSV LENAMMADLRGKMDQSALASVPQDPETFLPWYRNFISSHNASNHPFYRDFLEDRASAEDIRFYLAQETSLDPRFDDILSFLTVGTNGSEKMELVSNLWDE MGNGNSADVHTAVFAKTLTDAGVSKEFIDSNIMLESLVCGNVSAALALSRRHCYKAFGYFGVTEYLTPRRFRSYIVGCKRLGMPKSAYIYHDQHIQIDAR HGPSWFKNILLPSIAREPRCASEIVLGTVMRLETSTGYLDALQAKLELAR&LINK_LOC=protein&PAGE_TYPE=BlastSearch', 'BLAST this protein','Strvi_4643')" />

 
 
   QDDILVTTPLARARHPEHVLPVTVRLDCSGEPTFTDVLRRTREVLLDALAHRNLDASTLSAIAGPPQLVVSVSDTPPAMVDGAQLQLWWADDGSRAALHY RSPWFTATTAQRLLHQLTLLLAGAAAQDSTRLSDLPMLSPADQELLDRLPRPGATPVTAVPAQLFAEVAATHPDATAVRTRNTSISYRDLDVRATKLAHH LRQAGAGPGSVVGVCVGRGIDLPLSFLAVLKSGAVYLPLDPRHPAERLRDILDAADATLVVSTASEAVAVAAHTGPLLLLDRDEAAIAAQPDTPVTDLPD PAELAYLIYTSGSTGRPKGVEVTQAGLANLAVTLREEFAVGPQDRVSLFSSAAFDASVWEMTMAFAGGATLGVLTTAEAAPAEIAAEIRELGLTVGTYPP TLLRALTPDDLGDPRLVVSAGEQCDTDLAAAWAPGRRFVNAYGPTETTVCATFTDVAAPVTDAPAIGTALPNLAVHLLDHRLRPVPVGAPGEIFVAGVGL ARGYRRQPALTAAAFLPDPHGPAGSRMYRTGDLGRFGNDGRLYHLGRVDHQVKLRGFRVELGEIEHHLMAQPMVRDAAVVMRRDGDHDRLVAYLTVDEAR TDPVALRGALRAALSRALPEYMLPAAYVVLDAMPLNSSGKIDRAALPESSGEAPAGPVEQWQTPDEQRIAGLWRDALGVAQVRRTDSFFELGGHSLVAAQ MIGSVRQAYQLNRLPMRLLFEKPVLADFAAAVARL', 'http://blast.ncbi.nlm.nih.gov/Blast.cgi?PAGE=Proteins&PROGRAM=blastp&BLAST_PROGRAMS=blastp&QUERY=MTEALTSAGPTSRPGTPAELSDDPRHVEYWRRHLRGAPPRLGLPYDRPPVSAPPASPASCELTLPPTVGRAVAVLARRERASTLMVVTAAMAAWASRLTG QDDILVTTPLARARHPEHVLPVTVRLDCSGEPTFTDVLRRTREVLLDALAHRNLDASTLSAIAGPPQLVVSVSDTPPAMVDGAQLQLWWADDGSRAALHY RSPWFTATTAQRLLHQLTLLLAGAAAQDSTRLSDLPMLSPADQELLDRLPRPGATPVTAVPAQLFAEVAATHPDATAVRTRNTSISYRDLDVRATKLAHH LRQAGAGPGSVVGVCVGRGIDLPLSFLAVLKSGAVYLPLDPRHPAERLRDILDAADATLVVSTASEAVAVAAHTGPLLLLDRDEAAIAAQPDTPVTDLPD PAELAYLIYTSGSTGRPKGVEVTQAGLANLAVTLREEFAVGPQDRVSLFSSAAFDASVWEMTMAFAGGATLGVLTTAEAAPAEIAAEIRELGLTVGTYPP TLLRALTPDDLGDPRLVVSAGEQCDTDLAAAWAPGRRFVNAYGPTETTVCATFTDVAAPVTDAPAIGTALPNLAVHLLDHRLRPVPVGAPGEIFVAGVGL ARGYRRQPALTAAAFLPDPHGPAGSRMYRTGDLGRFGNDGRLYHLGRVDHQVKLRGFRVELGEIEHHLMAQPMVRDAAVVMRRDGDHDRLVAYLTVDEAR TDPVALRGALRAALSRALPEYMLPAAYVVLDAMPLNSSGKIDRAALPESSGEAPAGPVEQWQTPDEQRIAGLWRDALGVAQVRRTDSFFELGGHSLVAAQ MIGSVRQAYQLNRLPMRLLFEKPVLADFAAAVARL&LINK_LOC=protein&PAGE_TYPE=BlastSearch', 'BLAST this protein','VAB18032_22210')" />

 
 
   VRAGRIIESRDYTDPVGFARAFGQLDTLAAALAG', 'http://blast.ncbi.nlm.nih.gov/Blast.cgi?PAGE=Proteins&PROGRAM=blastp&BLAST_PROGRAMS=blastp&QUERY=MPRLVLGDRSQLEPLIALYAEPTDVRHPFAPFPTEPLRTRDDLRRHFGGATTEADGIETFAAVERTVHATADPEVVIGEFHYVGRAHARDFDVACVFVLR VRAGRIIESRDYTDPVGFARAFGQLDTLAAALAG&LINK_LOC=protein&PAGE_TYPE=BlastSearch', 'BLAST this protein','FRAAL_2918')" />
   VVGAVAPTGRPVTVPGILVIRVRDGQIVHTRDYMDGLGVAKATDRLPDLVAALGG', 'http://blast.ncbi.nlm.nih.gov/Blast.cgi?PAGE=Proteins&PROGRAM=blastp&BLAST_PROGRAMS=blastp&QUERY=MPETTAATPEPAVGPREIVERFHRAMLKKSADDLADLYAADARHEFPFLFPGLPACFNGREEIRAGYRAMWGATPVEAETIRDVVIHETAEAGTVVVEQT VVGAVAPTGRPVTVPGILVIRVRDGQIVHTRDYMDGLGVAKATDRLPDLVAALGG&LINK_LOC=protein&PAGE_TYPE=BlastSearch', 'BLAST this protein','SBI_07563')" />

 
 
   VAEDASRVRTGVGLRVMATLYSTVISLLRLDGHDSIAAALRFHERAPGRASLLVSTAHKRL', 'http://blast.ncbi.nlm.nih.gov/Blast.cgi?PAGE=Proteins&PROGRAM=blastp&BLAST_PROGRAMS=blastp&QUERY=MRPNTVTECGHGRRVTRTTKVLQLAPEDCGFPHARQLVQVRRTRTVYPAKGSGNKPKKSVEIIYLLCSLNHGDAPTHSLASWAQSHWRIENALHWVRDVT VAEDASRVRTGVGLRVMATLYSTVISLLRLDGHDSIAAALRFHERAPGRASLLVSTAHKRL&LINK_LOC=protein&PAGE_TYPE=BlastSearch', 'BLAST this protein','CVAR_2843')" />
   VLAALDADALDTALGAWAAAATTPPAGTRRRLAVDGKTLRGSRTPDSPGRHLLAALDHTSGVVLGQVAVDAKSNEIPALPVLLADLDLTDVIVTADALHT QRQTASWLVSRHAHYILTVKANQPALYAQLAALPWRRVKTAARTVERGHGRRERRTVKTTEVRAGLLFPHAVQAVQVTRRRQPLADGPATTEIVYLVTSL PTHQASPTLLATYAREHWLVENRLHWVRDVTFGEDLSQVRTGHAPQVMASLRNLAIAILRLTGATNIAQAIRHHARRPERPLETIKSLAC', 'http://blast.ncbi.nlm.nih.gov/Blast.cgi?PAGE=Proteins&PROGRAM=blastp&BLAST_PROGRAMS=blastp&QUERY=MPAAPVLPPAPVLDRLAAVGAGNQPPSPAGLLAVFNQLPDPRKPRGRRHSLAAVLTLATCAVLAGARSFTAIGEWSADAGQAVAGLLGVSRVPEESTFRR VLAALDADALDTALGAWAAAATTPPAGTRRRLAVDGKTLRGSRTPDSPGRHLLAALDHTSGVVLGQVAVDAKSNEIPALPVLLADLDLTDVIVTADALHT QRQTASWLVSRHAHYILTVKANQPALYAQLAALPWRRVKTAARTVERGHGRRERRTVKTTEVRAGLLFPHAVQAVQVTRRRQPLADGPATTEIVYLVTSL PTHQASPTLLATYAREHWLVENRLHWVRDVTFGEDLSQVRTGHAPQVMASLRNLAIAILRLTGATNIAQAIRHHARRPERPLETIKSLAC&LINK_LOC=protein&PAGE_TYPE=BlastSearch', 'BLAST this protein','Francci3_2121')" />
   TQVVTAKLICATLKSHYLMIVKSNQAKILARITALPWAEVPAAATDDSRGHGRVKTRTLQIITAARGIGFPYAKQIIRITRERLITATDQRSVEVVYAIC SLPFEHARPTAIMTWMRQHCGIENSLHWIRDVTFDEDRHRAHTGNGAQVLATLRDTAINLHRLNGADNIAEACRITALTANRRLDLLNPQFPSSQAC', 'http://blast.ncbi.nlm.nih.gov/Blast.cgi?PAGE=Proteins&PROGRAM=blastp&BLAST_PROGRAMS=blastp&QUERY=MSRLDPADLNARMGSYFTAHVASSDPSGLVPIALDGKMLRGALRAKATATHLVSVFAHRARLVLGQLAVAEKSNEIPCVCALLTLLPDNLRWLVTVDAMH TQVVTAKLICATLKSHYLMIVKSNQAKILARITALPWAEVPAAATDDSRGHGRVKTRTLQIITAARGIGFPYAKQIIRITRERLITATDQRSVEVVYAIC SLPFEHARPTAIMTWMRQHCGIENSLHWIRDVTFDEDRHRAHTGNGAQVLATLRDTAINLHRLNGADNIAEACRITALTANRRLDLLNPQFPSSQAC&LINK_LOC=protein&PAGE_TYPE=BlastSearch', 'BLAST this protein','MUL_0370')" />
   THLVSVFAHRARLVLGQLAVAEKSNEIPCVCALLTLLPGSLRWLVTVDAMHTQVVTAKLICATLKSHYLMIVKSNQAKILARITALPWAEVPAAATDDSR GHGRVETRTLQIITAARGIGFPYAKQIIRITRERLITATDQRSVEVVYAICSLPFEHARPTAIMTWMRQHCGIENSLHWIHDVTFDEDRHRAHTGNGAQV LATLRNTAINLHRLNGADNIAEACRITALTANRRLDLLNPQFPSSQAC', 'http://blast.ncbi.nlm.nih.gov/Blast.cgi?PAGE=Proteins&PROGRAM=blastp&BLAST_PROGRAMS=blastp&QUERY=MALLAIAVLATAARMRGYAGFATWAATASDDVLAQLRVRFRRPSEKTFRAVLSRLDPADLNARMGSYFTAHVASSDPSGLVPIALDGKMLRGALRAKATA THLVSVFAHRARLVLGQLAVAEKSNEIPCVCALLTLLPGSLRWLVTVDAMHTQVVTAKLICATLKSHYLMIVKSNQAKILARITALPWAEVPAAATDDSR GHGRVETRTLQIITAARGIGFPYAKQIIRITRERLITATDQRSVEVVYAICSLPFEHARPTAIMTWMRQHCGIENSLHWIHDVTFDEDRHRAHTGNGAQV LATLRNTAINLHRLNGADNIAEACRITALTANRRLDLLNPQFPSSQAC&LINK_LOC=protein&PAGE_TYPE=BlastSearch', 'BLAST this protein','MUL_4975')" />

 
 
   PADPPRGPGHPPPRPARAEPAPPQPVPPSKPTESVTQQLPTASATQRIATPPPAPSTFERATRPIRLAPPGAPAAPPPMPPHPPAPPSPARPPSQSSPTP PTPELPAASAPAASEGEEQPKSRGLVERMIDATRKLLPGRAETDSASASASASDSDSGSSTGTGTGELPSTNRLPLKPGARTIGVAAYQLGLTVDGHELI SDVSFTTRPGSLIAVVGPSRARNSSLAGLLARTRPLSDGVLTVDGHDVAAEPESMRSRIGVVTRDNRVHPRLTVEQALGYAARMRLPPDTSADNRRRVVN QVLDEVELTAQRATRVAKLTPDERRCAAMAIELITRPSLLVVDEPSAGLNPAQEMHVLAMLRRQADLGCVVVVASMPLAHLNMCDQVLLLTPAGTLAFAG PPVQIESTMGTASWPDIFARVSADPQAAHQSFQNRLRASVSPTPPSVLEPERRPAELTFGAQVRLILRRQVRVFLASRLYLVFLALLPFALGALTLLIPG DSGLDRPPPGSGNPHEAVEILAALSFAAVLMGTALTVRDLVSERQIFRREQAVGLSASAYLIGKIIMFGLVAAVQAAILTAIVLLIKGQPVHGAALLPNP GVEIYASVAATTIVSAIIGLTLSTLGSSLREVLPLVVPVILASLLFAGGLVPLVGTWGFDQIAWFVPAHWGFAATASTVDLHRVDVLATHNEVWAHYAGW WAFDIGMLVTFGVVAAGLARYRLRAPGVPADHGIAHSRS', 'http://blast.ncbi.nlm.nih.gov/Blast.cgi?PAGE=Proteins&PROGRAM=blastp&BLAST_PROGRAMS=blastp&QUERY=MRYVFTPGRDALVGYGNKFDIRLDGPAPFDTFDRPTPELALRFAGNRWVAIDQSRNGMFVDGARLATVDIRDGLTITVGDPHHGTPLRFQVTAAAGARSL PADPPRGPGHPPPRPARAEPAPPQPVPPSKPTESVTQQLPTASATQRIATPPPAPSTFERATRPIRLAPPGAPAAPPPMPPHPPAPPSPARPPSQSSPTP PTPELPAASAPAASEGEEQPKSRGLVERMIDATRKLLPGRAETDSASASASASDSDSGSSTGTGTGELPSTNRLPLKPGARTIGVAAYQLGLTVDGHELI SDVSFTTRPGSLIAVVGPSRARNSSLAGLLARTRPLSDGVLTVDGHDVAAEPESMRSRIGVVTRDNRVHPRLTVEQALGYAARMRLPPDTSADNRRRVVN QVLDEVELTAQRATRVAKLTPDERRCAAMAIELITRPSLLVVDEPSAGLNPAQEMHVLAMLRRQADLGCVVVVASMPLAHLNMCDQVLLLTPAGTLAFAG PPVQIESTMGTASWPDIFARVSADPQAAHQSFQNRLRASVSPTPPSVLEPERRPAELTFGAQVRLILRRQVRVFLASRLYLVFLALLPFALGALTLLIPG DSGLDRPPPGSGNPHEAVEILAALSFAAVLMGTALTVRDLVSERQIFRREQAVGLSASAYLIGKIIMFGLVAAVQAAILTAIVLLIKGQPVHGAALLPNP GVEIYASVAATTIVSAIIGLTLSTLGSSLREVLPLVVPVILASLLFAGGLVPLVGTWGFDQIAWFVPAHWGFAATASTVDLHRVDVLATHNEVWAHYAGW WAFDIGMLVTFGVVAAGLARYRLRAPGVPADHGIAHSRS&LINK_LOC=protein&PAGE_TYPE=BlastSearch', 'BLAST this protein','MMAR_3628')" />

 
 
   

 
 
   AVPTWRLRQIVAHDPELGDLILRAFLIRRSLLIGSGAGLRIIGSRYSPDTRRLREFAARNRLPHRWIDLEEDEEAEALVRNLGMAPDETPVVILGGTRVL RNPGNAELAREIGLPVPSAPEDVFDLVIVGAGPAGLAAAVYGASEGLRTVVLDAVATGGQAGTSSCIENYLGFPAGISGGELAERAVIQAKKFGAHLGVP AEATSLKRRDGHYTVGLRDEPPVEGRTVVIATGARYRKLGVPRLEDFEGNGVYYAATLAEVAFCRGEPVVVVGGGNSAGQATIFLARHADSVRLLVRADD LAQDMSRYLIDRIESHPKVEVMLRTEVRELVGAHALEAVVAEDTRTGERLRIDTRAMFVFIGADPCTTWLAPEIALDDRGFVLTGFDDGLPLETSMPGVF AVGDVRSQSVKRVASAVGEGSMAIRLVHEHFTRRQASQ', 'http://blast.ncbi.nlm.nih.gov/Blast.cgi?PAGE=Proteins&PROGRAM=blastp&BLAST_PROGRAMS=blastp&QUERY=MDAFAETPDHHGAFPRLSDEQIARLAPHGVRRPTRPGDILFREGEDCPDFFVILAGKAAVIQDERVVRVHGPGRFLGELGLLTGQKAFTTSVVCEAGEVL AVPTWRLRQIVAHDPELGDLILRAFLIRRSLLIGSGAGLRIIGSRYSPDTRRLREFAARNRLPHRWIDLEEDEEAEALVRNLGMAPDETPVVILGGTRVL RNPGNAELAREIGLPVPSAPEDVFDLVIVGAGPAGLAAAVYGASEGLRTVVLDAVATGGQAGTSSCIENYLGFPAGISGGELAERAVIQAKKFGAHLGVP AEATSLKRRDGHYTVGLRDEPPVEGRTVVIATGARYRKLGVPRLEDFEGNGVYYAATLAEVAFCRGEPVVVVGGGNSAGQATIFLARHADSVRLLVRADD LAQDMSRYLIDRIESHPKVEVMLRTEVRELVGAHALEAVVAEDTRTGERLRIDTRAMFVFIGADPCTTWLAPEIALDDRGFVLTGFDDGLPLETSMPGVF AVGDVRSQSVKRVASAVGEGSMAIRLVHEHFTRRQASQ&LINK_LOC=protein&PAGE_TYPE=BlastSearch', 'BLAST this protein','Sros_4028')" />

 
 
   RDRIVSDATGETVCEAYEGGELTAAEIIDRVQRACGISAKVLLVTLQKEQSLVSGRTARDPDDAALGAAMGARCPDTAPCDAGLAGFAAQIAQGATDLKS YSASDFMRQPGTHWIAYSPNPDCGGTDLTIANEATAALYNYTPYQPNPAALEAAWGTGDSCSSYGNRNFALYWALWFG', 'http://blast.ncbi.nlm.nih.gov/Blast.cgi?PAGE=Proteins&PROGRAM=blastp&BLAST_PROGRAMS=blastp&QUERY=MLRRRRITVGALVALGILTIVGLLVAAVVPLLGLASAWNTEVRKERIDLSSFDPGHLIDDADFYDGNAMTADEIQRFLDDQVGQCRTDTCLNVLRTTLPA RDRIVSDATGETVCEAYEGGELTAAEIIDRVQRACGISAKVLLVTLQKEQSLVSGRTARDPDDAALGAAMGARCPDTAPCDAGLAGFAAQIAQGATDLKS YSASDFMRQPGTHWIAYSPNPDCGGTDLTIANEATAALYNYTPYQPNPAALEAAWGTGDSCSSYGNRNFALYWALWFG&LINK_LOC=protein&PAGE_TYPE=BlastSearch', 'BLAST this protein','MTES_3428')" />

 
 
   PGGTAVVELAQASGLPLMADLDPLNAHTVGLGETIAAALDAGSRRILVALGGSASTDGGTGLLTALGARFLDPAGHPLPPGGGSLRSLATVDLTSLRAAP PDGVHCLVDVDAPLLGSTGAAAVFGPQKGAGAAEITLLDAGLARLVECMEQAGAAAGLATLPGAGAAGGTAYGLAAALGATLVPGATTIAEHAGLPAALA GADLLITGEGRFDHTSRAGKVVGAVSTLAAGAGLPLVVVAGQIAARPPMGGDAVALTDLAGGTAAAMAHPVHWLTQAGALLARRNSDGVTRAVIRQP', 'http://blast.ncbi.nlm.nih.gov/Blast.cgi?PAGE=Proteins&PROGRAM=blastp&BLAST_PROGRAMS=blastp&QUERY=MIPSPDAVPIPPTAPSGSPLRIVIAPDSFKGSLSATEAATALARGWRTVRPDDVVLTVPIADGGEGTLDVFSAAVPEAVRNTLDVTGPDGRRVTAGWLAL PGGTAVVELAQASGLPLMADLDPLNAHTVGLGETIAAALDAGSRRILVALGGSASTDGGTGLLTALGARFLDPAGHPLPPGGGSLRSLATVDLTSLRAAP PDGVHCLVDVDAPLLGSTGAAAVFGPQKGAGAAEITLLDAGLARLVECMEQAGAAAGLATLPGAGAAGGTAYGLAAALGATLVPGATTIAEHAGLPAALA GADLLITGEGRFDHTSRAGKVVGAVSTLAAGAGLPLVVVAGQIAARPPMGGDAVALTDLAGGTAAAMAHPVHWLTQAGALLARRNSDGVTRAVIRQP&LINK_LOC=protein&PAGE_TYPE=BlastSearch', 'BLAST this protein','Franean1_5723')" />

 
 
   DIGAYAEYKCLPETASLALKPVNKTYEEAVAAVDGATTALFFLRDKAKVRTGQKVLVNGASGSVGTYAVQLAKSFGAEVTGVCGPHNTELVKSLGADNVI DYTTEDFADDTEAYDVVFDAVGRNSFARCKGSLTEHGCYVPTSGLNNNLLQLWTSIRGGRKVVTGMSVRKNDALAYIKQLLEADTLRIVIDRSYPLERIV EAHRYVDTGHKSGNVVITVA', 'http://blast.ncbi.nlm.nih.gov/Blast.cgi?PAGE=Proteins&PROGRAM=blastp&BLAST_PROGRAMS=blastp&QUERY=MKAIIHTRFGGPEVLELAQVPKPTPKDGEVLVKVHATTVTTAECKMRRGEPLWGRLILGLRRPRRKLRTLGLELAGEIEAVGKDVRRFRPGDQVFGFTGF DIGAYAEYKCLPETASLALKPVNKTYEEAVAAVDGATTALFFLRDKAKVRTGQKVLVNGASGSVGTYAVQLAKSFGAEVTGVCGPHNTELVKSLGADNVI DYTTEDFADDTEAYDVVFDAVGRNSFARCKGSLTEHGCYVPTSGLNNNLLQLWTSIRGGRKVVTGMSVRKNDALAYIKQLLEADTLRIVIDRSYPLERIV EAHRYVDTGHKSGNVVITVA&LINK_LOC=protein&PAGE_TYPE=BlastSearch', 'BLAST this protein','RHA1_ro00434')" />
   GAGANAEYKCLSERASITTMPDNVAYAQAAAAVDGFTTAWHFLHDLAKVQPGQRVLVIGASGSIGTYAIQLAKYLGAVVHGVCSGRNAKLVESLGADRVF DYTVEDFTTSGERYSAIFDTVGRSSFARCRSVLAPRGCYLPTTGLVVNAMLTAGTALTTGPRVRTGMSVGKQAALDALRGLLGQDRLQVVIDRIYPMAEI VEAHRYVDEGHKVGNVVIDVVGDGRRNEP', 'http://blast.ncbi.nlm.nih.gov/Blast.cgi?PAGE=Proteins&PROGRAM=blastp&BLAST_PROGRAMS=blastp&QUERY=MKAVMFDAYGPPDVLTHVDVPTPVPRADQVLIRVHATTVTSAEVGMRRGEPRWGRVIIGLRRPRRGVRVLGLEFAGDVTAVGPAVRRLRAGDRVFGFTSF GAGANAEYKCLSERASITTMPDNVAYAQAAAAVDGFTTAWHFLHDLAKVQPGQRVLVIGASGSIGTYAIQLAKYLGAVVHGVCSGRNAKLVESLGADRVF DYTVEDFTTSGERYSAIFDTVGRSSFARCRSVLAPRGCYLPTTGLVVNAMLTAGTALTTGPRVRTGMSVGKQAALDALRGLLGQDRLQVVIDRIYPMAEI VEAHRYVDEGHKVGNVVIDVVGDGRRNEP&LINK_LOC=protein&PAGE_TYPE=BlastSearch', 'BLAST this protein','Sare_2400')" />

 
 
   FLVLRARYTDDVVEAAVNDGVDQLVLLGAGFDTTALRRVSNSTVKIFEVDAPTTQADKRAVMERLRPVQSPNPIVWVPCDFEHDTLREKLLDSGFDPTRP SLVVWLGVTAYLTREALDATLADLASLCAPGSRLVFDYLDTDVVAGDSTSLRARLWTQAAARFGEPYLTGLTATGADALLAAHGFTCEAHLTTRELLQHY ASADVGRSSVDGRAAITTAQRS', 'http://blast.ncbi.nlm.nih.gov/Blast.cgi?PAGE=Proteins&PROGRAM=blastp&BLAST_PROGRAMS=blastp&QUERY=MPRISRDGSGGDSVIVGYQRFGDHRVKSRRLDRRASFTAQSCAAQRAAETLQPPDRRLLDDPYSRYFIHSPLLRVCLIHPLAARAFIEVLKSVFGAAGHF FLVLRARYTDDVVEAAVNDGVDQLVLLGAGFDTTALRRVSNSTVKIFEVDAPTTQADKRAVMERLRPVQSPNPIVWVPCDFEHDTLREKLLDSGFDPTRP SLVVWLGVTAYLTREALDATLADLASLCAPGSRLVFDYLDTDVVAGDSTSLRARLWTQAAARFGEPYLTGLTATGADALLAAHGFTCEAHLTTRELLQHY ASADVGRSSVDGRAAITTAQRS&LINK_LOC=protein&PAGE_TYPE=BlastSearch', 'BLAST this protein','MAB_2209c')" />

 
 
   ELTQSYANEVVQVEWPALRAGGPVGGQGWELLSELQGEVERVPTRTDREENSRIEASTQLWNVYQARQDRLNASGNGVSAIVWFAILVGSAMSVGLMFMF GGPGVYSYAVIVSMLSGAIALMLFAIYQLQDPFSGGASVGPDAYVAALTRLARSG', 'http://blast.ncbi.nlm.nih.gov/Blast.cgi?PAGE=Proteins&PROGRAM=blastp&BLAST_PROGRAMS=blastp&QUERY=MSLFFSGLFWVLGLMMVTAVLAVILRKARQRFGREANNEVAGQVFTIVGGVNVVIAAFVLISLFDATDKADENTYQEANALVAVRWASESLAEPARSRVE ELTQSYANEVVQVEWPALRAGGPVGGQGWELLSELQGEVERVPTRTDREENSRIEASTQLWNVYQARQDRLNASGNGVSAIVWFAILVGSAMSVGLMFMF GGPGVYSYAVIVSMLSGAIALMLFAIYQLQDPFSGGASVGPDAYVAALTRLARSG&LINK_LOC=protein&PAGE_TYPE=BlastSearch', 'BLAST this protein','Kfla_4243')" />

 
 
   VRAISGVTYVSLADADGSVVISTRPGDVGVDARQTQDRSWTGLVDTSSGKAVEAHVPILGSEGGITGTAIVGIEYPSIWTRLLQSAPNLLVYLAVASLLG GVGSILLARRVKRQTLGMEAREILDLVRQREAMLRGLKEGVIAFDAQGKVVLMSDSAHQLLDIPRGSAGRSVRDLGLDDRLQSVLTSGTTESDQLVLVGD RLLVLNTVLIESKGRTIGSVTTFRDRTELRSVEEELDVTKTSTDALRAHIHEFDNQLHTISGLIQLGEYDEVVGYVEGLTMERAQVSAAVTDKIADVGTA ALVIAKIGAAAQGLVTVELTSDTHLDSVDRRLGRDLVTVVGNLVDNAVDAVGEMPEASRRKVRLSIRGRGTAVEVRVEDAGPGIAPENRDGVFGQGWSTK GQSSDGHGFGLALVRLTCRRRGGDVTVSSRSTEGIEWTVFTAVLHDGEGA', 'http://blast.ncbi.nlm.nih.gov/Blast.cgi?PAGE=Proteins&PROGRAM=blastp&BLAST_PROGRAMS=blastp&QUERY=MSSGNEQAERERKSPLRERLFRRRHTLATQLLELQLLIVVAVLVCVTALSLAQSSASFQREEGRRALSAAESLAANPAVRELLPQAQTRLRSGLQGSVDS VRAISGVTYVSLADADGSVVISTRPGDVGVDARQTQDRSWTGLVDTSSGKAVEAHVPILGSEGGITGTAIVGIEYPSIWTRLLQSAPNLLVYLAVASLLG GVGSILLARRVKRQTLGMEAREILDLVRQREAMLRGLKEGVIAFDAQGKVVLMSDSAHQLLDIPRGSAGRSVRDLGLDDRLQSVLTSGTTESDQLVLVGD RLLVLNTVLIESKGRTIGSVTTFRDRTELRSVEEELDVTKTSTDALRAHIHEFDNQLHTISGLIQLGEYDEVVGYVEGLTMERAQVSAAVTDKIADVGTA ALVIAKIGAAAQGLVTVELTSDTHLDSVDRRLGRDLVTVVGNLVDNAVDAVGEMPEASRRKVRLSIRGRGTAVEVRVEDAGPGIAPENRDGVFGQGWSTK GQSSDGHGFGLALVRLTCRRRGGDVTVSSRSTEGIEWTVFTAVLHDGEGA&LINK_LOC=protein&PAGE_TYPE=BlastSearch', 'BLAST this protein','RER_13840')" />

 
 
   LTVRLRPLSSRGRWAGREPFGIGAGRNAAHPGPVAVVTRARLRPTRALSFWRAVPPVVAELGGAPGLRLALGIGEAPVGLQGTFSIWDSATALTDFAYRS TAHRQAIRDTVPKHWYAEELFARFAVLELAGRYQGRTP', 'http://blast.ncbi.nlm.nih.gov/Blast.cgi?PAGE=Proteins&PROGRAM=blastp&BLAST_PROGRAMS=blastp&QUERY=MHLAGLVTQVATPVVDLRIWGVRSVTGALGRMAYGGVGLRRLPGLRFGKLMGTGSARTFTPRDADPRHWALLTVWPDIDAADAAGSSRFIRSWSEASHEE LTVRLRPLSSRGRWAGREPFGIGAGRNAAHPGPVAVVTRARLRPTRALSFWRAVPPVVAELGGAPGLRLALGIGEAPVGLQGTFSIWDSATALTDFAYRS TAHRQAIRDTVPKHWYAEELFARFAVLELAGRYQGRTP&LINK_LOC=protein&PAGE_TYPE=BlastSearch', 'BLAST this protein','Gbro_2549')" />
   EEVPRRNTGGPVAVLTRASIRPSRLVPFYRSVPQVERLLGEQAGCLASVGVGEWPLARQATFSLWRDSHAVRDFAYRGQAHREAIGQTRAEGWYSEELFA RFVPYGSEGTWNGTDPLARYGGRGG', 'http://blast.ncbi.nlm.nih.gov/Blast.cgi?PAGE=Proteins&PROGRAM=blastp&BLAST_PROGRAMS=blastp&QUERY=MDLGSMRYMAFDRPVLAATPGLRFWRLLGSGRGTSMSLGADLRRWALLAVWSEERALEDFLETSPVAARWRDQARESWQVRLAPLASRGRWGGVEPFGPI EEVPRRNTGGPVAVLTRASIRPSRLVPFYRSVPQVERLLGEQAGCLASVGVGEWPLARQATFSLWRDSHAVRDFAYRGQAHREAIGQTRAEGWYSEELFA RFVPYGSEGTWNGTDPLARYGGRGG&LINK_LOC=protein&PAGE_TYPE=BlastSearch', 'BLAST this protein','Sros_3830')" />

 
 
   TLREFGGLQSYPSRSKDPDPVDYSTGSVGIGATAPIWGAFARRYIDTVFASAGVGRQYSLVGDAELDEGAVWEAILDPSVQHLGEIVWIVDMNRQSLDRV VPNIAAGRLESMFSAAGWQVITVRFGGLLEELFTRPGGQALRRRILDMPNPEYQRLLRCAAAELRERLPGSDPGADAITALVDGLDDATLIAAIRNLGGH DLNTLRDAYAQIDDTRPTVIIAYTIKGHGLPTEGHPQNHSSLLSEEQYAELAQTLGKDPTRPWASFEDASPAARLCAAAAARLRRDSVPLGAPPTVPVDL GRTPPAVSTTQAALGRALLDLVREAPEVAKRVVTVSPDVSSSTNLAGWLNKVGVWSIDERRNWFDDDRETIMHWREKPTGQHMELGIAETNLVGLIGELG ATWSRWGQPLFPIGVLYDPFVERALEPWSYGIYAGGQSILVGTPSGVTLAAEGGAHQSIKTPSIGLEQPGCVSFEPAFAVEVEWTLMDCLSRLGRPGGSS SYLRLSTRPVLQELAAVPADPAARARRRRHVVAGGYTLRHADKPAVTLVAVGALVSESLAGADRLAQLGVAAEVVCVTSPGLLYEALQARHGLGEAPSWI LDQIFPAERAAPMVTVLDGHPHTLAFLATLHRVPVKSLGVSRFGQAGSLDAVYKYHGIDAESIVRAALDLVQ', 'http://blast.ncbi.nlm.nih.gov/Blast.cgi?PAGE=Proteins&PROGRAM=blastp&BLAST_PROGRAMS=blastp&QUERY=MTITGNATRASGGDPLEQVAEQVLWLSTAMIHHANRVRPNPSGMKVGGHQASSASMVSIMTSLWFEQLQPGDRVSVKPHASPVLHAINYLLGELDEKYLT TLREFGGLQSYPSRSKDPDPVDYSTGSVGIGATAPIWGAFARRYIDTVFASAGVGRQYSLVGDAELDEGAVWEAILDPSVQHLGEIVWIVDMNRQSLDRV VPNIAAGRLESMFSAAGWQVITVRFGGLLEELFTRPGGQALRRRILDMPNPEYQRLLRCAAAELRERLPGSDPGADAITALVDGLDDATLIAAIRNLGGH DLNTLRDAYAQIDDTRPTVIIAYTIKGHGLPTEGHPQNHSSLLSEEQYAELAQTLGKDPTRPWASFEDASPAARLCAAAAARLRRDSVPLGAPPTVPVDL GRTPPAVSTTQAALGRALLDLVREAPEVAKRVVTVSPDVSSSTNLAGWLNKVGVWSIDERRNWFDDDRETIMHWREKPTGQHMELGIAETNLVGLIGELG ATWSRWGQPLFPIGVLYDPFVERALEPWSYGIYAGGQSILVGTPSGVTLAAEGGAHQSIKTPSIGLEQPGCVSFEPAFAVEVEWTLMDCLSRLGRPGGSS SYLRLSTRPVLQELAAVPADPAARARRRRHVVAGGYTLRHADKPAVTLVAVGALVSESLAGADRLAQLGVAAEVVCVTSPGLLYEALQARHGLGEAPSWI LDQIFPAERAAPMVTVLDGHPHTLAFLATLHRVPVKSLGVSRFGQAGSLDAVYKYHGIDAESIVRAALDLVQ&LINK_LOC=protein&PAGE_TYPE=BlastSearch', 'BLAST this protein','Srot_1317')" />

 
 
   DSNGGGGTIGGGDTQWMTAGGGILHIETPPEHLVMSGGLFHGVQLWVNLPRDNKMAAPRYQDITGQKVALLSSPDGGALVRVIAGDVDGHHGPGSTYTPI SLVHATIAPGASLTLPWNPEFNALAYVLAGEGLVGSERRPIHMGQTAVYGRGDTLTIAAADTQDSRTKAFEVFLLGGKPIREPVAMAGPFVMNTKAEVLQ AFEDFQAGRLGSVPAAHETLD', 'http://blast.ncbi.nlm.nih.gov/Blast.cgi?PAGE=Proteins&PROGRAM=blastp&BLAST_PROGRAMS=blastp&QUERY=MPAVTVDNILALPRIDAPAPGAVDRPVRSLTTAPVGYEGEGFPVRRAFAGIDLPALDPFIHMDQMGEVDYAPGEPKGTPWHPHRGFETVTYMIDGIMEHQ DSNGGGGTIGGGDTQWMTAGGGILHIETPPEHLVMSGGLFHGVQLWVNLPRDNKMAAPRYQDITGQKVALLSSPDGGALVRVIAGDVDGHHGPGSTYTPI SLVHATIAPGASLTLPWNPEFNALAYVLAGEGLVGSERRPIHMGQTAVYGRGDTLTIAAADTQDSRTKAFEVFLLGGKPIREPVAMAGPFVMNTKAEVLQ AFEDFQAGRLGSVPAAHETLD&LINK_LOC=protein&PAGE_TYPE=BlastSearch', 'BLAST this protein','RHA1_ro04228')" />
   DSNGGGGTIGGGDTQWMTAGGGILHIETPPEHLVTSGGLFHGVQLWVNLPRDNKMAAPRYQDITGAKVALLSSPDGGALVRVIAGDVDGHHGPGSTYTPI SLVHATVAPGASLTLPWNPEFNALAYVLAGDGLAGSERRPIRMGQTAVFGRGDTLTIAAADTQDSRTKSFEVFVLGGKPIREPVAMAGPFVMNTRAEVLQ AFEDFQAGRLGSVPAAHETLA', 'http://blast.ncbi.nlm.nih.gov/Blast.cgi?PAGE=Proteins&PROGRAM=blastp&BLAST_PROGRAMS=blastp&QUERY=MPAVTVDNILALPRIDAPAPGAVDRPVRSLTTAPVGYEGEGFPVRRAFAGIDLPALDPFIHMDQMGEVDYAPGEPKGTPWHPHRGFETVTYMIDGIMEHQ DSNGGGGTIGGGDTQWMTAGGGILHIETPPEHLVTSGGLFHGVQLWVNLPRDNKMAAPRYQDITGAKVALLSSPDGGALVRVIAGDVDGHHGPGSTYTPI SLVHATVAPGASLTLPWNPEFNALAYVLAGDGLAGSERRPIRMGQTAVFGRGDTLTIAAADTQDSRTKSFEVFVLGGKPIREPVAMAGPFVMNTRAEVLQ AFEDFQAGRLGSVPAAHETLA&LINK_LOC=protein&PAGE_TYPE=BlastSearch', 'BLAST this protein','ROP_41530')" />

 
 
   AVGVVLFGASHAPGNERYWEAALAVFSVFVSWTIVHTVFTLKYARLYYLGTPGGIDFNEPDPPQYSDFAYLAFTIGMTFQVSDTDLQTKEIRRAALRHAW MSFPLGAVIIATSINLVSGLAK', 'http://blast.ncbi.nlm.nih.gov/Blast.cgi?PAGE=Proteins&PROGRAM=blastp&BLAST_PROGRAMS=blastp&QUERY=MRPDPGPRSVPPAFHASARLKLFVAVAAGALACVVAALGGAGRTSPLIGWDVLAVVFGGWTWRVVWRLDPALTRTHAQEENPSRDLADALLIGASLASLV AVGVVLFGASHAPGNERYWEAALAVFSVFVSWTIVHTVFTLKYARLYYLGTPGGIDFNEPDPPQYSDFAYLAFTIGMTFQVSDTDLQTKEIRRAALRHAW MSFPLGAVIIATSINLVSGLAK&LINK_LOC=protein&PAGE_TYPE=BlastSearch', 'BLAST this protein','Caci_3416')" />

 
 
   FYEEPAQRVCGVPWPANAAAFYCTERATLVFPLTGGWIEGRTDLYPLKVAAHEYGHHLQSLTGVRGDYEARVRSGRAPARQAELGRRYELQADCLSGVFL GSVRGSLSRTEQDWQALSDALRASGDDGDYRTHGTGANRLRWFGRGYRAVSPAACDTWTATPAQVS', 'http://blast.ncbi.nlm.nih.gov/Blast.cgi?PAGE=Proteins&PROGRAM=blastp&BLAST_PROGRAMS=blastp&QUERY=MRFLPPGAGPLRPAALTRPALLVTVCLALLPSGVAQAAPPGSPLLRVGKPAAASCPEPPIIDGGMPRTREYLTAAMRCMDRLWSARFARAGLRFRKPAVR FYEEPAQRVCGVPWPANAAAFYCTERATLVFPLTGGWIEGRTDLYPLKVAAHEYGHHLQSLTGVRGDYEARVRSGRAPARQAELGRRYELQADCLSGVFL GSVRGSLSRTEQDWQALSDALRASGDDGDYRTHGTGANRLRWFGRGYRAVSPAACDTWTATPAQVS&LINK_LOC=protein&PAGE_TYPE=BlastSearch', 'BLAST this protein','Sros_4034')" />

 
 
   LRTGATLPDPRLEALRRFTVAVLDHRGAVPDADLDALLAAGWQARHALDVVLGVGTYTISTFANRLTEAPLDEPLAAYAWQPAA', 'http://blast.ncbi.nlm.nih.gov/Blast.cgi?PAGE=Proteins&PROGRAM=blastp&BLAST_PROGRAMS=blastp&QUERY=MSRFTAYEPDTAPAAARPVMAGVRRSLGHLPAAVSLMAGSPELLKGFLAANAAFEATDLDPVAREVVVLTVATRNGCHLCVAMHTATLVRHGAAPELIEA LRTGATLPDPRLEALRRFTVAVLDHRGAVPDADLDALLAAGWQARHALDVVLGVGTYTISTFANRLTEAPLDEPLAAYAWQPAA&LINK_LOC=protein&PAGE_TYPE=BlastSearch', 'BLAST this protein','Micau_0344')" />
   LRTGATLPDPRLEALRRFTAAVLDHRGAVPDADLDALLAAGWQARHALDVVLGVGTYTISTFANRLTEAPLDEPLAAYAWQPAA', 'http://blast.ncbi.nlm.nih.gov/Blast.cgi?PAGE=Proteins&PROGRAM=blastp&BLAST_PROGRAMS=blastp&QUERY=MSRFTAYEPDTAPAAARPVMAGVRRSLGHLPAAVSLMAGSPELLKGFLAANAAFEATDLDPVAREVVVLTVATRNGCHLCVAMHTATLVRHGAAPELIGA LRTGATLPDPRLEALRRFTAAVLDHRGAVPDADLDALLAAGWQARHALDVVLGVGTYTISTFANRLTEAPLDEPLAAYAWQPAA&LINK_LOC=protein&PAGE_TYPE=BlastSearch', 'BLAST this protein','ML5_0320')" />

 
 
   RYLSELEQDGIAVIPTRWVSRGEDVTIDTLMTQTGWDHVVIKPTVSAGSWRTFRVSRSGPSTSATHFVRHGHEATSATGVAPRRDTEPETLLRDLVSTHH VCVQPFLSSILAHGELSFVFLGGKLSHAVRKRVARDGGWWAHERFGGRNEVIQPTPAEREWAYCVYEALENRYGPLIFGRVDGLRDEHRVLRLLECELVI PRLLLTEGNAYDTYAKTIARAVGG', 'http://blast.ncbi.nlm.nih.gov/Blast.cgi?PAGE=Proteins&PROGRAM=blastp&BLAST_PROGRAMS=blastp&QUERY=MKAVTTGRPIAYLHLDSPHAEYPQIVTALADYGVTAVAVHLDNVHTVDWTAFDGVNLRMCRGFHERPDFLDRIRRLYLELDGKIPILNSMRVAVGTLDKR RYLSELEQDGIAVIPTRWVSRGEDVTIDTLMTQTGWDHVVIKPTVSAGSWRTFRVSRSGPSTSATHFVRHGHEATSATGVAPRRDTEPETLLRDLVSTHH VCVQPFLSSILAHGELSFVFLGGKLSHAVRKRVARDGGWWAHERFGGRNEVIQPTPAEREWAYCVYEALENRYGPLIFGRVDGLRDEHRVLRLLECELVI PRLLLTEGNAYDTYAKTIARAVGG&LINK_LOC=protein&PAGE_TYPE=BlastSearch', 'BLAST this protein','Francci3_0994')" />

 
 
   VLNDRQTGYPLACLESSIISATRTAASAALAADWLTRDRARPARLGIVGTGLIARYVHAYLAAAGWSFDDVGVFDLNPAHADGFSGYLRAAGQPSVTVHE SAEDLIRGSDLVVFATVAGEPHVHDPAWFGHNPLVLHVSLRDLSPEVILASTNVVDDVEHCLKANTSVHLAEQRTGDRDFLSGTLHDVMTGKASLPKDRP LVFSPFGLGVLDLAVGKHVYDQLRVSGDLRTVPEFFFELDRYGKR', 'http://blast.ncbi.nlm.nih.gov/Blast.cgi?PAGE=Proteins&PROGRAM=blastp&BLAST_PROGRAMS=blastp&QUERY=MSTSTSVPEFAVISGAQVHEVLHGQEKLVIDLIESAYRLHGDGDTVNPPSYFLRFPDRPSSRIIALPASVGGDIAVDGLKWISSFPENVASGLPRASAIL VLNDRQTGYPLACLESSIISATRTAASAALAADWLTRDRARPARLGIVGTGLIARYVHAYLAAAGWSFDDVGVFDLNPAHADGFSGYLRAAGQPSVTVHE SAEDLIRGSDLVVFATVAGEPHVHDPAWFGHNPLVLHVSLRDLSPEVILASTNVVDDVEHCLKANTSVHLAEQRTGDRDFLSGTLHDVMTGKASLPKDRP LVFSPFGLGVLDLAVGKHVYDQLRVSGDLRTVPEFFFELDRYGKR&LINK_LOC=protein&PAGE_TYPE=BlastSearch', 'BLAST this protein','AMED_4040')" />
   ILNDHDTGYPFACMEASVISASRTAASAASAADWLSRGRGRPTRVGFFGVGLIARYIHSYLAATGWSFDEIGVHDLSADSAGGFRSYLEQSGTSARVTVH DTAEQLIRSSDLVVFATIAGEPHVSDVSWFDHNPLVLHVSLRDLAPEVLLASTNVVDDVEHCLKASTSPHLAEQLTGSRDFLLGTLADVMAGRVTVPADR PVVFSPFGLGVLDLAVGKAVYDEVAAAGELQVVEDFFTELRRYG', 'http://blast.ncbi.nlm.nih.gov/Blast.cgi?PAGE=Proteins&PROGRAM=blastp&BLAST_PROGRAMS=blastp&QUERY=MSQQLTVPPFAVVPGAQVQSALHGREKEIVELVEATYRLHGAGDSVNPPSYFLRFPDRPSARIIALPASIGGDVRVDGIKWISSFPDNVSAGIPRASAVL ILNDHDTGYPFACMEASVISASRTAASAASAADWLSRGRGRPTRVGFFGVGLIARYIHSYLAATGWSFDEIGVHDLSADSAGGFRSYLEQSGTSARVTVH DTAEQLIRSSDLVVFATIAGEPHVSDVSWFDHNPLVLHVSLRDLAPEVLLASTNVVDDVEHCLKASTSPHLAEQLTGSRDFLLGTLADVMAGRVTVPADR PVVFSPFGLGVLDLAVGKAVYDEVAAAGELQVVEDFFTELRRYG&LINK_LOC=protein&PAGE_TYPE=BlastSearch', 'BLAST this protein','Gobs_3075')" />
   NTDNGYAYAFIEASRISAARTAASAALAVRVLHGAPTSIGVVGSGPIAQTTLHFIKSLYTTAVPVKIHDLNSELVARIVTRHGPECSVVSLEEALGCDLV LLATSAGTPYVPMSVRFQPNQLVLNISLRDLHPETIRTANNVFDDVEHCLKANTTPHLLEQLNGNRNFITGTLAEFICSEKQLDPDHPTVFSPFGLGILD LAVAQSLYEHVQISGDGLRVPDFHGDMKR', 'http://blast.ncbi.nlm.nih.gov/Blast.cgi?PAGE=Proteins&PROGRAM=blastp&BLAST_PROGRAMS=blastp&QUERY=MTKYGEMLIIDAKLVETFLKGRESFLIDLVERVYRAHHAGNTVCPDSYFLRFPDTPRDRIIALPSYINDQTCVSGIKWISSFPENVDHGLQRASAVIVLN NTDNGYAYAFIEASRISAARTAASAALAVRVLHGAPTSIGVVGSGPIAQTTLHFIKSLYTTAVPVKIHDLNSELVARIVTRHGPECSVVSLEEALGCDLV LLATSAGTPYVPMSVRFQPNQLVLNISLRDLHPETIRTANNVFDDVEHCLKANTTPHLLEQLNGNRNFITGTLAEFICSEKQLDPDHPTVFSPFGLGILD LAVAQSLYEHVQISGDGLRVPDFHGDMKR&LINK_LOC=protein&PAGE_TYPE=BlastSearch', 'BLAST this protein','PPA_1289')" />
   VLNDRQTGYPLACLESSIISATRTAASAALAADWLTRDRARPARLGIVGTGLIARYVHAYLAAAGWSFDDVGVFDLNPAHADGFSGYLRAAGQPSVTVHE SAEDLIRGSDLVVFATVAGEPHVHDPAWFGHNPLVLHVSLRDLSPEVILASTNVVDDVEHCLKANTSVHLAEQRTGDRDFLSGTLHDVMTGKASLPKDRP LVFSPFGLGVLDLAVGKHVYDQLRVSGDLRTVPEFFFELDRYGKR', 'http://blast.ncbi.nlm.nih.gov/Blast.cgi?PAGE=Proteins&PROGRAM=blastp&BLAST_PROGRAMS=blastp&QUERY=MSTSTSVPEFAVISGAQVHEVLHGQEKLVIDLIESAYRLHGDGDTVNPPSYFLRFPDRPSSRIIALPASVGGDIAVDGLKWISSFPENVASGLPRASAIL VLNDRQTGYPLACLESSIISATRTAASAALAADWLTRDRARPARLGIVGTGLIARYVHAYLAAAGWSFDDVGVFDLNPAHADGFSGYLRAAGQPSVTVHE SAEDLIRGSDLVVFATVAGEPHVHDPAWFGHNPLVLHVSLRDLSPEVILASTNVVDDVEHCLKANTSVHLAEQRTGDRDFLSGTLHDVMTGKASLPKDRP LVFSPFGLGVLDLAVGKHVYDQLRVSGDLRTVPEFFFELDRYGKR&LINK_LOC=protein&PAGE_TYPE=BlastSearch', 'BLAST this protein','RAM_20590')" />
   GHPVAFVEGAVISAKRTAASAALAARELTADNPPAAALLIGCGVINLEILRFLAAALPDLREAALYDTDPARAEAFAARCAEEVPGVKARPVTDLAAALG EHRLVSLATTAATPHMDLSACGPDTTVLHISLRDLTAEAVLGAVNVVDDADHVCRERTSLDLAQQATGNRDFVAAPIGALLRGTATLRRTADRPVVYSPF GLGVLDLALAEFVREEADRRGLGVRVEDFLPAFA', 'http://blast.ncbi.nlm.nih.gov/Blast.cgi?PAGE=Proteins&PROGRAM=blastp&BLAST_PROGRAMS=blastp&QUERY=MLILRRADVTDVLSGRETEIIDLVAETYRLHDEGQTSLPHSTFLRFPEEHHSRDRIIGLPAYRGGERPVAGMKWISSFPGNVAAGTDRASAAVVLNSLGN GHPVAFVEGAVISAKRTAASAALAARELTADNPPAAALLIGCGVINLEILRFLAAALPDLREAALYDTDPARAEAFAARCAEEVPGVKARPVTDLAAALG EHRLVSLATTAATPHMDLSACGPDTTVLHISLRDLTAEAVLGAVNVVDDADHVCRERTSLDLAQQATGNRDFVAAPIGALLRGTATLRRTADRPVVYSPF GLGVLDLALAEFVREEADRRGLGVRVEDFLPAFA&LINK_LOC=protein&PAGE_TYPE=BlastSearch', 'BLAST this protein','SAV_3645')" />
   PVALVEGSTISARRTAASAALAAASLPAEDGQETGVALIGCGVINFEVLRFLLVVRPELTDVTVFDLDAGRAEEFRERCASEFGGAGRVVRVADSAEEAL GTHRLVSLATTATVPHLDLRACRPGTLVLHVSLRDVTVEGVLDAVNVVDDPDHVCRAATSVHLAETRSGSRSFIDSTLGELLRKTDAGHRDPRRITLFSP FGLGVLDLALAGLVLRRARAAGVGTEITGFLPEPRATGSG', 'http://blast.ncbi.nlm.nih.gov/Blast.cgi?PAGE=Proteins&PROGRAM=blastp&BLAST_PROGRAMS=blastp&QUERY=MLTLGNGDVRRLLDGREREVLDAVREAYLRHADEETSLPHSVFLRFPDDTRNRIIGLPAYLGGPEPLAGMKWIASFPGNVADGLERASAAIIVNSMRTGQ PVALVEGSTISARRTAASAALAAASLPAEDGQETGVALIGCGVINFEVLRFLLVVRPELTDVTVFDLDAGRAEEFRERCASEFGGAGRVVRVADSAEEAL GTHRLVSLATTATVPHLDLRACRPGTLVLHVSLRDVTVEGVLDAVNVVDDPDHVCRAATSVHLAETRSGSRSFIDSTLGELLRKTDAGHRDPRRITLFSP FGLGVLDLALAGLVLRRARAAGVGTEITGFLPEPRATGSG&LINK_LOC=protein&PAGE_TYPE=BlastSearch', 'BLAST this protein','Sfla_0080')" />
   AVLLLNDPDTGYPAACLESSVISATRTAASAVLAAETLGGGRSARRVGVVGTGLIARHVWKFLRDLDWQIDGFTLYDLDPAAARSFGEELTAEGAKEFTV ADDVAQAFTECDLVILTTVAGEPHIHDPKLLDHAPLVLHLSLRDLAPEMILAAQNITDDTEHAVRERTSLHLTEQAENGRDFITGTLADVLLGRVEVDRS RPVVFSPFGLGVLDLAVGSWVHSRAQEAGAGRVVNDFFASTTTK', 'http://blast.ncbi.nlm.nih.gov/Blast.cgi?PAGE=Proteins&PROGRAM=blastp&BLAST_PROGRAMS=blastp&QUERY=MSVRTPDSPPSLIFIPEAAVAAQIEGHRERCVDLVRRAYLTHDNGDSVNPQSGFLRLPDHPKSRIISLPAHLGGEFGVSGLKWISSFPDNPRTYGIPRAS AVLLLNDPDTGYPAACLESSVISATRTAASAVLAAETLGGGRSARRVGVVGTGLIARHVWKFLRDLDWQIDGFTLYDLDPAAARSFGEELTAEGAKEFTV ADDVAQAFTECDLVILTTVAGEPHIHDPKLLDHAPLVLHLSLRDLAPEMILAAQNITDDTEHAVRERTSLHLTEQAENGRDFITGTLADVLLGRVEVDRS RPVVFSPFGLGVLDLAVGSWVHSRAQEAGAGRVVNDFFASTTTK&LINK_LOC=protein&PAGE_TYPE=BlastSearch', 'BLAST this protein','SGR_2591')" />
   DTQTGYPFACLESSIISAARTAASAALAAVELSRRRGVRPRRAGFFGVGLIARYLHTYLVAGGLEFEAIGVHDLVAEHAEGFKGYLERTERGPITIYDKP EDLIRSCDLVVFATVAGEPHVLDPSWFEHNPLVLHISLRDLSPEIILSSYNVVDDVEHCMKANTSPHLAEQRVGNRDFVAGTLYDVLTDRITPPADKPVI FSPFGLGVLDLAVARYVYDQVKAKGELQTVPDFFYELKRYG', 'http://blast.ncbi.nlm.nih.gov/Blast.cgi?PAGE=Proteins&PROGRAM=blastp&BLAST_PROGRAMS=blastp&QUERY=MSQVPSFAVISGAQVHQAVSGREEQIIRIVEAAYRLHGQGRTVNPDSYFLRFPDRPSSRIIALPASVGGEVDVHGIKWIASFPENVAAGIPRASAVLILN DTQTGYPFACLESSIISAARTAASAALAAVELSRRRGVRPRRAGFFGVGLIARYLHTYLVAGGLEFEAIGVHDLVAEHAEGFKGYLERTERGPITIYDKP EDLIRSCDLVVFATVAGEPHVLDPSWFEHNPLVLHISLRDLSPEIILSSYNVVDDVEHCMKANTSPHLAEQRVGNRDFVAGTLYDVLTDRITPPADKPVI FSPFGLGVLDLAVARYVYDQVKAKGELQTVPDFFYELKRYG&LINK_LOC=protein&PAGE_TYPE=BlastSearch', 'BLAST this protein','Tcur_1883')" />

 
 
   EAVPGSAPDETPPPAGGA', 'http://blast.ncbi.nlm.nih.gov/Blast.cgi?PAGE=Proteins&PROGRAM=blastp&BLAST_PROGRAMS=blastp&QUERY=MRRTTTNVLLVLASVAVFAVALALGTGKGEFGGTDATATEQIEESAPDYEPWFEPLWTQPGGEVESGLFALQAALGAGLLGFALGTFRERRKHLGDRAPG EAVPGSAPDETPPPAGGA&LINK_LOC=protein&PAGE_TYPE=BlastSearch', 'BLAST this protein','Amir_3590')" />

 
 
   HELETLARRQSELEDQELDLMEQREQKQAAAEADARDLADKRTALAEIEKRRDEALAAIDAELAAERSTREGISVDIPEDLRKLYEKIRRTKPIAAALLR QRRCESCRLEQSGAELADLRAADESDVVRCDNCGAILVRTEESGL', 'http://blast.ncbi.nlm.nih.gov/Blast.cgi?PAGE=Proteins&PROGRAM=blastp&BLAST_PROGRAMS=blastp&QUERY=MRANPADQRRLLDLQQADTSLTQLAHRRANLPEEAEIVTLRQQVNELADRAGSNEATVGDLDRDIAKVEREIDQVRRRADTDRERQASGKLGPKELEGIA HELETLARRQSELEDQELDLMEQREQKQAAAEADARDLADKRTALAEIEKRRDEALAAIDAELAAERSTREGISVDIPEDLRKLYEKIRRTKPIAAALLR QRRCESCRLEQSGAELADLRAADESDVVRCDNCGAILVRTEESGL&LINK_LOC=protein&PAGE_TYPE=BlastSearch', 'BLAST this protein','Snas_4816')" />
   QAEIASLQRRQSDLEEVVLEIMERREEAEAKAAGLRAEREAAERELAEVTKRRDEAWRQIDEESGTTSAARTEVAKEIPEDLLALYEKLRGQFGGVGAAA LHRGRCQGCHLALNTVDLNRIRAAAEDEVVRCEECRRILIRTPESGL', 'http://blast.ncbi.nlm.nih.gov/Blast.cgi?PAGE=Proteins&PROGRAM=blastp&BLAST_PROGRAMS=blastp&QUERY=MKAAPQAQQRLLKLQELDTALDRLAHRRRTLPEKAEIERLQARLAELRDAIVAAETEVGDLDREQKKAEQDVEQVRTRARRDQERLDSGMITSAKELSSL QAEIASLQRRQSDLEEVVLEIMERREEAEAKAAGLRAEREAAERELAEVTKRRDEAWRQIDEESGTTSAARTEVAKEIPEDLLALYEKLRGQFGGVGAAA LHRGRCQGCHLALNTVDLNRIRAAAEDEVVRCEECRRILIRTPESGL&LINK_LOC=protein&PAGE_TYPE=BlastSearch', 'BLAST this protein','Tcur_1674')" />

 
 
   VVDTFAIVIRYFGGIKLGAGGLVRAYTAGVEQAVAAATLLRRTELAVARIEVPPAEVGLAENAVRVWAAAHAATVEPTQYTSRSALLTVLVPPERFAELS ADTARWSSGRRTVEDAGRRTADVPF', 'http://blast.ncbi.nlm.nih.gov/Blast.cgi?PAGE=Proteins&PROGRAM=blastp&BLAST_PROGRAMS=blastp&QUERY=MSRPRLRGMSAPLVLSADVETELVEKRSRFLTRLHRVESVEQADALLRTARAEHPDARHHCTALVLAETPERAEMHRSNDDGEPAGTAGMPMLQSLLHAH VVDTFAIVIRYFGGIKLGAGGLVRAYTAGVEQAVAAATLLRRTELAVARIEVPPAEVGLAENAVRVWAAAHAATVEPTQYTSRSALLTVLVPPERFAELS ADTARWSSGRRTVEDAGRRTADVPF&LINK_LOC=protein&PAGE_TYPE=BlastSearch', 'BLAST this protein','Bfae_24840')" />

 
 
   QVTRTRDQLEPAVAADAARVNAALAEIRTDPRINDAIVCSSPAVHRDLVRGATGGRIRRQLAAYAQRLAAKSETMSFFGPINYGRVDAESNAQTTLSWAG HQEIRVRQAHCAARVNDAVQAMIIDDDSLVTHLVPIRKTTTRPPRGHDPSAELIRAVDGTRTLAEIAAAGGDEVTRTVGLFRTAVAKGVLTHSWCPPATT VDPLRWTLGRIDDAGAEVTSRAVAVRSLLVKVLDLLDGYPWTPPERKLALQAEVEALLPTGGPTHRSRFYNDRVIIHEAAVGTAQLEVRGQLAHDLNAAV APVLDLLAHEAELTRVCTNRAVARRLGPGRTPLLQALRVCADMSIEYGGRLSAKLARVVGELGADASELDVAGRLPTVPAPTAPVLCSIDVLIGTADLAA YDPDTTPLVLGDIHDAALLTPWALQFHPDSTRLLAERDTAVRRALGDQIAVNVISRRTTGLPPLEFPGVVLELGGTATAGRHRIGLDELWLDSDGDQVTL RSTTFPGRSLLFHNGELDTAVHTALALPRIRRAVLPALPHVPRLRWGNAVFSRRRWMLQSSEVQASLRGQRDLDLLLGAARLVDERGLPSRFFVKSPAER KPVYVDTASPDLLKGMARLAATDDHLAVTEALPAPTDAWLRDGELRFASELRCVYLRGGGR', 'http://blast.ncbi.nlm.nih.gov/Blast.cgi?PAGE=Proteins&PROGRAM=blastp&BLAST_PROGRAMS=blastp&QUERY=MTVPTRAGPADPSWELLPVLVLRSAGFPWQLIESLAYRQTTTLLGELATHEQRAAEVASRLRPSARLTRGQQSKLRNLRPLPPSDVFDGAWLAEWNDVTG QVTRTRDQLEPAVAADAARVNAALAEIRTDPRINDAIVCSSPAVHRDLVRGATGGRIRRQLAAYAQRLAAKSETMSFFGPINYGRVDAESNAQTTLSWAG HQEIRVRQAHCAARVNDAVQAMIIDDDSLVTHLVPIRKTTTRPPRGHDPSAELIRAVDGTRTLAEIAAAGGDEVTRTVGLFRTAVAKGVLTHSWCPPATT VDPLRWTLGRIDDAGAEVTSRAVAVRSLLVKVLDLLDGYPWTPPERKLALQAEVEALLPTGGPTHRSRFYNDRVIIHEAAVGTAQLEVRGQLAHDLNAAV APVLDLLAHEAELTRVCTNRAVARRLGPGRTPLLQALRVCADMSIEYGGRLSAKLARVVGELGADASELDVAGRLPTVPAPTAPVLCSIDVLIGTADLAA YDPDTTPLVLGDIHDAALLTPWALQFHPDSTRLLAERDTAVRRALGDQIAVNVISRRTTGLPPLEFPGVVLELGGTATAGRHRIGLDELWLDSDGDQVTL RSTTFPGRSLLFHNGELDTAVHTALALPRIRRAVLPALPHVPRLRWGNAVFSRRRWMLQSSEVQASLRGQRDLDLLLGAARLVDERGLPSRFFVKSPAER KPVYVDTASPDLLKGMARLAATDDHLAVTEALPAPTDAWLRDGELRFASELRCVYLRGGGR&LINK_LOC=protein&PAGE_TYPE=BlastSearch', 'BLAST this protein','Sare_3272')" />
   EVTRTRDQLEPTVAADAARVNTALAEIRTDPRINDAIVCSSPAVHRDLVRGATGGRIRRQLAAYAQRLAAKSETMSFFGPINYGRLDAGSEAPTTLSWAG HREIRVRQAHCAARVNDAVQTMIIDDESLVTYLVPIRKTTVRPPRRDDPSAELIRAVDGDRTIAELAAACGDEVTSTVGLFRTAVAKGVLTHSWCPPATT VDPLRWTLERIDEAGPELASRAAAVRSLLVKVLDLLDSYPWTPPERKLAIQAEVEALLPVGGPTHRSRFYNDRVIIHEAAVGTAQLELRGQLAHDLRAAA APVLDLLAHEAELTRVQTNRAVARRLGPGRTPLLRALRACADMSIEYGGQLSTELARVVGEAGPEVSELDVAGLLPTVPAPTAPILCSIDVLIGTANLAD YDPSTTPLVLGDIHDAALLTPWALQFHPDAATLLAERDAAVRRALGDQIAVNVISRRTTGLPPLEFPGVVLELGGSATAGRDRIGLDELWLDSDGEQVVL RGASFPGRSLLLHNGELDTAVHTALALPRIRRAVLPALPYLPRLRWGNAVFSRRRWLVPSAEVQALLRGQRDVDLLLGAARLVTERALPNRFFVKSPAER KPVYVDTASPDLLKGMARLAATTEHLAITEALPAPTDAWLRDGELRFASELRCVYLRGGGQ', 'http://blast.ncbi.nlm.nih.gov/Blast.cgi?PAGE=Proteins&PROGRAM=blastp&BLAST_PROGRAMS=blastp&QUERY=MTVPTRSGPTDPSWELLPVLVLRSAGFPWQLIESLTYRQTAALLGELATLERRAAEVASRVRASARLTRGQQSKLRNVRPLPPSDVFASDWLAEWNDVAS EVTRTRDQLEPTVAADAARVNTALAEIRTDPRINDAIVCSSPAVHRDLVRGATGGRIRRQLAAYAQRLAAKSETMSFFGPINYGRLDAGSEAPTTLSWAG HREIRVRQAHCAARVNDAVQTMIIDDESLVTYLVPIRKTTVRPPRRDDPSAELIRAVDGDRTIAELAAACGDEVTSTVGLFRTAVAKGVLTHSWCPPATT VDPLRWTLERIDEAGPELASRAAAVRSLLVKVLDLLDSYPWTPPERKLAIQAEVEALLPVGGPTHRSRFYNDRVIIHEAAVGTAQLELRGQLAHDLRAAA APVLDLLAHEAELTRVQTNRAVARRLGPGRTPLLRALRACADMSIEYGGQLSTELARVVGEAGPEVSELDVAGLLPTVPAPTAPILCSIDVLIGTANLAD YDPSTTPLVLGDIHDAALLTPWALQFHPDAATLLAERDAAVRRALGDQIAVNVISRRTTGLPPLEFPGVVLELGGSATAGRDRIGLDELWLDSDGEQVVL RGASFPGRSLLLHNGELDTAVHTALALPRIRRAVLPALPYLPRLRWGNAVFSRRRWLVPSAEVQALLRGQRDVDLLLGAARLVTERALPNRFFVKSPAER KPVYVDTASPDLLKGMARLAATTEHLAITEALPAPTDAWLRDGELRFASELRCVYLRGGGQ&LINK_LOC=protein&PAGE_TYPE=BlastSearch', 'BLAST this protein','Strop_3046')" />

 
 
   TVGYQIGYPVALAGATIVLNTPGLGFAIGSSNGIDLGLLPELSLGLNAGTNVELAGDIIPSQELDIDLEPGGITTVPILEGQEFDGSAAVVRMQGVHGSI SGAIGPVTIRPYAMAVTENGDTVMTYGVPQKLN', 'http://blast.ncbi.nlm.nih.gov/Blast.cgi?PAGE=Proteins&PROGRAM=blastp&BLAST_PROGRAMS=blastp&QUERY=MTDISTSRFGYKRFTRTVAVAGIAALAMVMGNGSASAGVDNSSSVIDARGNRIEVVQGDTQYQTVPPLDGVPTSVEFFHNGYAGVAITGPNAEEFEGTQL TVGYQIGYPVALAGATIVLNTPGLGFAIGSSNGIDLGLLPELSLGLNAGTNVELAGDIIPSQELDIDLEPGGITTVPILEGQEFDGSAAVVRMQGVHGSI SGAIGPVTIRPYAMAVTENGDTVMTYGVPQKLN&LINK_LOC=protein&PAGE_TYPE=BlastSearch', 'BLAST this protein','ROP_39490')" />

 
 
   DAQNSLAELAALAETAGAMVLDAVFQRRDKPDPATYIGSGKALELRDIVLESGADTVVCDGELSPGQLIHLEDVVKVKVVDRTALILDIFAQHAKSREGK AQVSLAQMQYMLPRLRGWGQSLSRQMGGGGSSGGGGMATRGPGETKIETDRRRIREKMAKMRREIAEMKTGREIKRQERKRNKVPSVAIAGYTNAGKSSL LNRLTGAGVLVENALFATLDPTVRRAETPSGRLYTLADTVGFVRHLPHHLVEAFRSTMEEVGDSDLILHVVDGAHPVPEEQLAAVREVIRDVGAVDVREI VVVNKADAADPEVLQRLLRNEKYAIAVSARTGAGIDELLALIDAELPRPSVEIEALVPYVQGALVSRVHAEGEVVSEEHTSEGTLLKARVHEELAAELST FALAAH', 'http://blast.ncbi.nlm.nih.gov/Blast.cgi?PAGE=Proteins&PROGRAM=blastp&BLAST_PROGRAMS=blastp&QUERY=MTSSSSLPQDARDAQSATDTASESLTESLRADALMEEDVAWSHAIDTERDGEQLDRSERAALRRVAGLSTELEDVTEVEYRQLRLERVVLVGVWTSGTVT DAQNSLAELAALAETAGAMVLDAVFQRRDKPDPATYIGSGKALELRDIVLESGADTVVCDGELSPGQLIHLEDVVKVKVVDRTALILDIFAQHAKSREGK AQVSLAQMQYMLPRLRGWGQSLSRQMGGGGSSGGGGMATRGPGETKIETDRRRIREKMAKMRREIAEMKTGREIKRQERKRNKVPSVAIAGYTNAGKSSL LNRLTGAGVLVENALFATLDPTVRRAETPSGRLYTLADTVGFVRHLPHHLVEAFRSTMEEVGDSDLILHVVDGAHPVPEEQLAAVREVIRDVGAVDVREI VVVNKADAADPEVLQRLLRNEKYAIAVSARTGAGIDELLALIDAELPRPSVEIEALVPYVQGALVSRVHAEGEVVSEEHTSEGTLLKARVHEELAAELST FALAAH&LINK_LOC=protein&PAGE_TYPE=BlastSearch', 'BLAST this protein','SACTE_4958')" />
   ELAALAETAGALVLDGVVQRRDKPDAATYIGSGKANELRDIVLETGADTVICDGELSPGQLIHLEDVVKVKVIDRTALILDIFAQHAKSREGKAQVALAQ MQYMLPRLRGWGQSLSRQMGGGKGGGLATRGPGETKIETDRRRIREKMAKMRREIADMKTGREIKRQERRRNKVPSVAIAGYTNAGKSSLLNRLTGAGVL VENALFATLDPTVRRAETPSGRLYTLADTVGFVRHLPHHLVEAFRSTMEEVGDSDLILHVVDGSHPVPEEQLAAVREVIRDVGATGVPEIVVINKADAAD PLVLQRLMRNEKRSIAVSARTGQGIAELLALIDNELPRPSVEIEALVPYTHGKLVARAHTEGEVISEEHTPEGTLLKARVHEELAADLAPYVPAPLA', 'http://blast.ncbi.nlm.nih.gov/Blast.cgi?PAGE=Proteins&PROGRAM=blastp&BLAST_PROGRAMS=blastp&QUERY=MTSSSSPSQDAQSFAHTYPEGLRADALMEEDVAWSQEIDGDRDGDQFDRSERAALRRVAGLSTELEDVTEVEYRQLRLERVVLVGVWTSGTIQDSENSLA ELAALAETAGALVLDGVVQRRDKPDAATYIGSGKANELRDIVLETGADTVICDGELSPGQLIHLEDVVKVKVIDRTALILDIFAQHAKSREGKAQVALAQ MQYMLPRLRGWGQSLSRQMGGGKGGGLATRGPGETKIETDRRRIREKMAKMRREIADMKTGREIKRQERRRNKVPSVAIAGYTNAGKSSLLNRLTGAGVL VENALFATLDPTVRRAETPSGRLYTLADTVGFVRHLPHHLVEAFRSTMEEVGDSDLILHVVDGSHPVPEEQLAAVREVIRDVGATGVPEIVVINKADAAD PLVLQRLMRNEKRSIAVSARTGQGIAELLALIDNELPRPSVEIEALVPYTHGKLVARAHTEGEVISEEHTPEGTLLKARVHEELAADLAPYVPAPLA&LINK_LOC=protein&PAGE_TYPE=BlastSearch', 'BLAST this protein','SAV_2470')" />
   AALAETAGALVLDGVIQRRDKPDPATYIGSGKARELRDIVIESGADTVVCDGELSPGQLIHLEDVVKVKVVDRTALILDIFAQHAKSREGKAQVSLAQMQ YMLPRLRGWGQSLSRQMGGGGSGAAGGGGMATRGPGETKIETDRRRIREKMAKMRREIAEMKTGRDIKRQERKRHKVPSVAIAGYTNAGKSSLLNRLTGA GVLVENALFATLDPTVRRAETPSGRLYTLADTVGFVRHLPHHLVEAFRSTMEEVGDADLIVHVVDGSHPMPEEQLAAVREVIRDVGAVDVPEIVVINKAD MADPLVLQRLLRVEKRALVVSARTGQGIDELLGLIDNELPRPQVEIEALVPYTQGKLVARTHAEGEVISEEHTPEGTLLKARVHEELAAELRRYVPAGSA QG', 'http://blast.ncbi.nlm.nih.gov/Blast.cgi?PAGE=Proteins&PROGRAM=blastp&BLAST_PROGRAMS=blastp&QUERY=MTHSSSLPQDRQRIAFAESLRADALMEEDVAWSHEIDQERDGDQYDRSERAALRRVVGLSTELEDVTEVEYRQLRLERVVLVGVWTSGTMQDAENSLSEL AALAETAGALVLDGVIQRRDKPDPATYIGSGKARELRDIVIESGADTVVCDGELSPGQLIHLEDVVKVKVVDRTALILDIFAQHAKSREGKAQVSLAQMQ YMLPRLRGWGQSLSRQMGGGGSGAAGGGGMATRGPGETKIETDRRRIREKMAKMRREIAEMKTGRDIKRQERKRHKVPSVAIAGYTNAGKSSLLNRLTGA GVLVENALFATLDPTVRRAETPSGRLYTLADTVGFVRHLPHHLVEAFRSTMEEVGDADLIVHVVDGSHPMPEEQLAAVREVIRDVGAVDVPEIVVINKAD MADPLVLQRLLRVEKRALVVSARTGQGIDELLGLIDNELPRPQVEIEALVPYTQGKLVARTHAEGEVISEEHTPEGTLLKARVHEELAAELRRYVPAGSA QG&LINK_LOC=protein&PAGE_TYPE=BlastSearch', 'BLAST this protein','SBI_03318')" />
   AELAALAETAGALVLDGVIQRRDKPDAATYIGSGKANELRDVVLESGADTVICDGELSPGQLIHLEDVVKVKVIDRTALILDIFAQHAKSREGKAQVALA QMQYMLPRLRGWGQSLSRQMGGGKGGGLATRGPGETKIETDRRRIREKMAKMRREIAEMKTGREIKRQERRRNKVPSVAIAGYTNAGKSSLLNRLTGAGV LVENALFATLDPTVRRAETPSGRLYTLADTVGFVRHLPHHLVEAFRSTMEEVGDADLILHVVDGSHPAPEEQLAAVREVIRDVGATKVPEIVVINKADAA DPLTLQRLLRVEKRSIAVSARSGQGIQELLALIDNELPRPSVEIEALVPYTHGKLVARTHTEGEVISEEHTPEGTLLKARVHEELAADLAPYVPAATV', 'http://blast.ncbi.nlm.nih.gov/Blast.cgi?PAGE=Proteins&PROGRAM=blastp&BLAST_PROGRAMS=blastp&QUERY=MTSSSSPSQAAQSAFAQNNPENLRADALMEEDVAWSFEIDGERDGDQFDRSDRAALRRVAGLSTELEDVTEVEYRQLRLERVVLVGVWTTGTVRDAENSL AELAALAETAGALVLDGVIQRRDKPDAATYIGSGKANELRDVVLESGADTVICDGELSPGQLIHLEDVVKVKVIDRTALILDIFAQHAKSREGKAQVALA QMQYMLPRLRGWGQSLSRQMGGGKGGGLATRGPGETKIETDRRRIREKMAKMRREIAEMKTGREIKRQERRRNKVPSVAIAGYTNAGKSSLLNRLTGAGV LVENALFATLDPTVRRAETPSGRLYTLADTVGFVRHLPHHLVEAFRSTMEEVGDADLILHVVDGSHPAPEEQLAAVREVIRDVGATKVPEIVVINKADAA DPLTLQRLLRVEKRSIAVSARSGQGIQELLALIDNELPRPSVEIEALVPYTHGKLVARTHTEGEVISEEHTPEGTLLKARVHEELAADLAPYVPAATV&LINK_LOC=protein&PAGE_TYPE=BlastSearch', 'BLAST this protein','SCAB_24721')" />
   ELAALAETAGALVLDGVVQRRDKPDAATYIGSGKAEELRDVVLHTGADTVICDGELSPGQLIHLEDVVKVKVIDRTALILDIFAQHAKSREGKAQVALAQ MQYMLPRLRGWGQSLSRQMGGGKGGGLATRGPGETKIETDRRRIREKMAKMRREIAEMKTGREIKRQERKRHKVPSVAIAGYTNAGKSSLLNRLTGAGVL VENALFATLDPTVRRAETPSGRLYTLADTVGFVRHLPHHLVEAFRSTMEEVGDSDLILHVVDGSHPVPEEQLAAVREVIRDVGATDVPEIVVINKADMAD PLVLQRLLRIEKRAIAVSARTGQNIDQLLALIDNELPRPSVEIEALVPYTHGKLVARAHDEGEVISEEHTPEGTLLKVRVHEELAAELAPYVPAPLA', 'http://blast.ncbi.nlm.nih.gov/Blast.cgi?PAGE=Proteins&PROGRAM=blastp&BLAST_PROGRAMS=blastp&QUERY=MTSSSSPSQDTKRLAQTYPEGLRADALMEEDVAWNRGNDSQWDGEQFDRSDRAALRRVAGLSTELEDVTEVEYRQLRLERVVLVGVWTSGTVQDAENSLA ELAALAETAGALVLDGVVQRRDKPDAATYIGSGKAEELRDVVLHTGADTVICDGELSPGQLIHLEDVVKVKVIDRTALILDIFAQHAKSREGKAQVALAQ MQYMLPRLRGWGQSLSRQMGGGKGGGLATRGPGETKIETDRRRIREKMAKMRREIAEMKTGREIKRQERKRHKVPSVAIAGYTNAGKSSLLNRLTGAGVL VENALFATLDPTVRRAETPSGRLYTLADTVGFVRHLPHHLVEAFRSTMEEVGDSDLILHVVDGSHPVPEEQLAAVREVIRDVGATDVPEIVVINKADMAD PLVLQRLLRIEKRAIAVSARTGQNIDQLLALIDNELPRPSVEIEALVPYTHGKLVARAHDEGEVISEEHTPEGTLLKVRVHEELAAELAPYVPAPLA&LINK_LOC=protein&PAGE_TYPE=BlastSearch', 'BLAST this protein','SCO_5796')" />
   ISLAELAALAETAGAQVLDAVFQRRDKPDPATYIGSGKALELRDIVLESGADTVVCDGELSPGQLIHLEDVVKVKVVDRTALILDIFAQHAKSREGKAQV SLAQMQYMLPRLRGWGQSLSRQMGGGGSSGGGGMATRGPGETKIETDRRRIREKMAKMRREIAEMKTGREIKRQERKRNKVPSVAIAGYTNAGKSSLLNR LTGAGVLVENALFATLDPTVRRAETPSGRLYTLADTVGFVRHLPHHLVEAFRSTMEEVGDSDLILHVVDGAHPVPEEQLAAVRQVIREVGAVDVPEIVVI NKADAADPLVLQRLLRTEKHAIAVSARTGAGIDELLALIDAELPRPSVEIEALVPYTQGALVSRVHAEGEVLSEEHTPEGTILKARVHEELSADLGMFAP VAH', 'http://blast.ncbi.nlm.nih.gov/Blast.cgi?PAGE=Proteins&PROGRAM=blastp&BLAST_PROGRAMS=blastp&QUERY=MTSSSSLPQDAQSATDANSESFTESLRADALMEEDVAWSHEIDGERDGDQLDRSERAALRRVAGLSTELEDVTEVEYRQLRLERVVLVGVWTSGTVRDAE ISLAELAALAETAGAQVLDAVFQRRDKPDPATYIGSGKALELRDIVLESGADTVVCDGELSPGQLIHLEDVVKVKVVDRTALILDIFAQHAKSREGKAQV SLAQMQYMLPRLRGWGQSLSRQMGGGGSSGGGGMATRGPGETKIETDRRRIREKMAKMRREIAEMKTGREIKRQERKRNKVPSVAIAGYTNAGKSSLLNR LTGAGVLVENALFATLDPTVRRAETPSGRLYTLADTVGFVRHLPHHLVEAFRSTMEEVGDSDLILHVVDGAHPVPEEQLAAVRQVIREVGAVDVPEIVVI NKADAADPLVLQRLLRTEKHAIAVSARTGAGIDELLALIDAELPRPSVEIEALVPYTQGALVSRVHAEGEVLSEEHTPEGTILKARVHEELSADLGMFAP VAH&LINK_LOC=protein&PAGE_TYPE=BlastSearch', 'BLAST this protein','Sfla_1562')" />
   DAEISLAELAALAETAGAQVLDAVYQRRDKPDPATYIGSGKALELRDIVLESGADTVVCDGELSPGQLIHLEDVVKVKVVDRTALILDIFAQHAKSREGK AQVSLAQMQYMLPRLRGWGQSLSRQMGGGGSSGGGGMATRGPGETKIETDRRRIREKMAKMRREIAEMKTGREIKRQERKRNKVPSVAIAGYTNAGKSSL LNRLTGAGVLVENALFATLDPTVRRAETPSGRVYTLADTVGFVRHLPHHLVEAFRSTMEEVGESDLILHVVDGSHPVPEEQLAAVREVIRDVGAVDVREI VVINKADAADPLVLQRLLRNEKHAIAVSARTGAGIDELLALIDTELPRPSVEIEVLVPYIQGALVSRVHAEGEVLSEEHTAEGTLLKAQVHEELAAELGT FVPAAH', 'http://blast.ncbi.nlm.nih.gov/Blast.cgi?PAGE=Proteins&PROGRAM=blastp&BLAST_PROGRAMS=blastp&QUERY=MTSSSSLPQDAQDARSATENTTESLTESLRADALMEEDVAWSHEIDGARDGDQLDRSERAALRRVAGLSTELEDVTEVEYRQLRLERVVLVGVWTSGTVR DAEISLAELAALAETAGAQVLDAVYQRRDKPDPATYIGSGKALELRDIVLESGADTVVCDGELSPGQLIHLEDVVKVKVVDRTALILDIFAQHAKSREGK AQVSLAQMQYMLPRLRGWGQSLSRQMGGGGSSGGGGMATRGPGETKIETDRRRIREKMAKMRREIAEMKTGREIKRQERKRNKVPSVAIAGYTNAGKSSL LNRLTGAGVLVENALFATLDPTVRRAETPSGRVYTLADTVGFVRHLPHHLVEAFRSTMEEVGESDLILHVVDGSHPVPEEQLAAVREVIRDVGAVDVREI VVINKADAADPLVLQRLLRNEKHAIAVSARTGAGIDELLALIDTELPRPSVEIEVLVPYIQGALVSRVHAEGEVLSEEHTAEGTLLKAQVHEELAAELGT FVPAAH&LINK_LOC=protein&PAGE_TYPE=BlastSearch', 'BLAST this protein','SGR_1724')" />
   LAETAGALVLDGVIQRRNKPDPATYIGSGKAEELRDIVVESGADTVVCDGELSPGQLIHLEDVVKVKVVDRTALILDIFAQHAKSREGKAQVSLAQMQYM LPRLRGWGQSLSRQMGGGGSGSSGGGMATRGPGETKIETDRRRIREKMAKMRREIAEMKTGRDIKRQERRRHKVPSVAIAGYTNAGKSSLLNRLTGAGVL VENALFATLDPTVRRAETPSGRLYTLADTVGFVRHLPHHLVEAFRSTMEEVGDADLIVHVVDGSHPAPEEQLAAVREVIRDVGAVDVPEIVVINKADLAD PLVVQRLLRMERRAMAVSARSGLGIDELLAVIDEELPRPQVEIEVLLPYTHGKLVARTHVEGEVLSEEHTPEGTLLKARVHEELAAELRRFVPAAAAGQH ', 'http://blast.ncbi.nlm.nih.gov/Blast.cgi?PAGE=Proteins&PROGRAM=blastp&BLAST_PROGRAMS=blastp&QUERY=MTHSSSLPQDRQRPAESLRADALMEEDVAWSHEIDEERDGDQYDRSDRAALRRVVGLSTELEDITEVEYRQLRLERVVLVGVWTSGTAQDAENSLAELAA LAETAGALVLDGVIQRRNKPDPATYIGSGKAEELRDIVVESGADTVVCDGELSPGQLIHLEDVVKVKVVDRTALILDIFAQHAKSREGKAQVSLAQMQYM LPRLRGWGQSLSRQMGGGGSGSSGGGMATRGPGETKIETDRRRIREKMAKMRREIAEMKTGRDIKRQERRRHKVPSVAIAGYTNAGKSSLLNRLTGAGVL VENALFATLDPTVRRAETPSGRLYTLADTVGFVRHLPHHLVEAFRSTMEEVGDADLIVHVVDGSHPAPEEQLAAVREVIRDVGAVDVPEIVVINKADLAD PLVVQRLLRMERRAMAVSARSGLGIDELLAVIDEELPRPQVEIEVLLPYTHGKLVARTHVEGEVLSEEHTPEGTLLKARVHEELAAELRRFVPAAAAGQH &LINK_LOC=protein&PAGE_TYPE=BlastSearch', 'BLAST this protein','Strvi_2123')" />

 
 
   TVATVGGSLVLGLLLALLLNQRLAGRGIARTVAFAPYVLSGIAVGMLWLFIFDPRYGLMSTVLAWIGLNSPDWYNDGPWALAMVIIVYLWKNIGYVALIY LAGLQAVPQDLRDAAALDGASSRRTLVSIVLPLLGPTTFFLSVTTLLSSLQSFDIIHAMTKGGPLGSTTTLMYQIYQESFVSGRAGYASAVATILFGILL VVTLVQLKFVERKVHY', 'http://blast.ncbi.nlm.nih.gov/Blast.cgi?PAGE=Proteins&PROGRAM=blastp&BLAST_PROGRAMS=blastp&QUERY=MTSAALDAPPALSAPPVPPPVRRRPRARRWMSYLTFVALAGPNVALLVIFVYKPLLQSFQYSTLQWNIGSPTARNVGLGNYVDWFQDPRTPEILTTTAIF TVATVGGSLVLGLLLALLLNQRLAGRGIARTVAFAPYVLSGIAVGMLWLFIFDPRYGLMSTVLAWIGLNSPDWYNDGPWALAMVIIVYLWKNIGYVALIY LAGLQAVPQDLRDAAALDGASSRRTLVSIVLPLLGPTTFFLSVTTLLSSLQSFDIIHAMTKGGPLGSTTTLMYQIYQESFVSGRAGYASAVATILFGILL VVTLVQLKFVERKVHY&LINK_LOC=protein&PAGE_TYPE=BlastSearch', 'BLAST this protein','Sked_18860')" />

 
 
   GHVQQLDAVNSRLLAGLTARVPDLIRGGSDRDGIAFLDVDDTIREVHGYAKQAAAYGYSKVFGLNAMLAVCSTPLTAPVIVASALRRGNTASGKGSARML TRAAATARQAGVSGRLMGRADSAFYRHDLVTAMIKAKMWFSVTARKNTAVKTAITRIPEQAWVPIKYPNAVWEADDTVPGGGYWVSDAEVAETSFVAFSS KKKAQQVPCRLVVRRVRRLQPTASDGTVQGELFAAHRHHAFITNSTLTTIAADQHHRDHAIIEQVIAELKDGPLAHLPSGRYGANAAWLAHAVIAFNLAR AIGVLAGKAHARARWATLRAQLINLPARIATTARTMILHLPRDWAWADQWQQTFDGATGPPPMSLL', 'http://blast.ncbi.nlm.nih.gov/Blast.cgi?PAGE=Proteins&PROGRAM=blastp&BLAST_PROGRAMS=blastp&QUERY=MKACHTVRPVFDDPNLVGCAGLVPALLLGESAGLHELLGEHVSVDCPNPVVKSAGVIAGMLTGADSIDDLNVVRHGGMPRLLGGTRAPSTYGTYLRSFTH GHVQQLDAVNSRLLAGLTARVPDLIRGGSDRDGIAFLDVDDTIREVHGYAKQAAAYGYSKVFGLNAMLAVCSTPLTAPVIVASALRRGNTASGKGSARML TRAAATARQAGVSGRLMGRADSAFYRHDLVTAMIKAKMWFSVTARKNTAVKTAITRIPEQAWVPIKYPNAVWEADDTVPGGGYWVSDAEVAETSFVAFSS KKKAQQVPCRLVVRRVRRLQPTASDGTVQGELFAAHRHHAFITNSTLTTIAADQHHRDHAIIEQVIAELKDGPLAHLPSGRYGANAAWLAHAVIAFNLAR AIGVLAGKAHARARWATLRAQLINLPARIATTARTMILHLPRDWAWADQWQQTFDGATGPPPMSLL&LINK_LOC=protein&PAGE_TYPE=BlastSearch', 'BLAST this protein','MLP_17810')" />

 
 
   AVRSLFGIGEARHSTVGIVLAAVSLAVMPVLSWAQRRAGRELGSLSAVADSKQTLLCTYLSAVLLAGLVLNSAFGWSWADSVAALVIAAIAVKEGREAWR GEACCAAPPRDAANCCEAERDGAPGETAL', 'http://blast.ncbi.nlm.nih.gov/Blast.cgi?PAGE=Proteins&PROGRAM=blastp&BLAST_PROGRAMS=blastp&QUERY=MAADRALTAHRREILSRRIRWFVTATISYNIVEAIVALAEGSRVSSSALIGFGLDSVIEVSSAAAVAWQFAGRDPEAREKVALRVIAFSFFALAAFVTVD AVRSLFGIGEARHSTVGIVLAAVSLAVMPVLSWAQRRAGRELGSLSAVADSKQTLLCTYLSAVLLAGLVLNSAFGWSWADSVAALVIAAIAVKEGREAWR GEACCAAPPRDAANCCEAERDGAPGETAL&LINK_LOC=protein&PAGE_TYPE=BlastSearch', 'BLAST this protein','Mjls_4415')" />
   AVRSLFGIGEARHSTVGIVLAAVSLAVMPVLSWAQRRAGRELGSLSAVADSKQTLLCTYLSAVLLAGLVLNSAFGWSWADSVAALVIAAIAVKEGREAWR GEACCAAPPRDAANCCEAERDGAPGETAL', 'http://blast.ncbi.nlm.nih.gov/Blast.cgi?PAGE=Proteins&PROGRAM=blastp&BLAST_PROGRAMS=blastp&QUERY=MAADRALTAHRREILSRRIRWFVTATISYNIVEAIVALAEGSRVSSSALIGFGLDSVIEVSSAAAVAWQFAGRDPEAREKVALRVIAFSFFALAAFVTVD AVRSLFGIGEARHSTVGIVLAAVSLAVMPVLSWAQRRAGRELGSLSAVADSKQTLLCTYLSAVLLAGLVLNSAFGWSWADSVAALVIAAIAVKEGREAWR GEACCAAPPRDAANCCEAERDGAPGETAL&LINK_LOC=protein&PAGE_TYPE=BlastSearch', 'BLAST this protein','Mkms_4254')" />
   AVRSLFGIGEARHSTVGIVLAAVSLAVMPVLSWAQRRAGRELGSLSAVADSKQTLLCTYLSAVLLAGLVLNSAFGWSWADSVAALVIAAIAVKEGREAWR GEACCAAPPRDAANCCEAERDGAPGETAL', 'http://blast.ncbi.nlm.nih.gov/Blast.cgi?PAGE=Proteins&PROGRAM=blastp&BLAST_PROGRAMS=blastp&QUERY=MAADRALTAHRREILSRRIRWFVTATISYNIVEAIVALAEGSRVSSSALIGFGLDSVIEVSSAAAVAWQFAGRDPEAREKVALRVIAFSFFALAAFVTVD AVRSLFGIGEARHSTVGIVLAAVSLAVMPVLSWAQRRAGRELGSLSAVADSKQTLLCTYLSAVLLAGLVLNSAFGWSWADSVAALVIAAIAVKEGREAWR GEACCAAPPRDAANCCEAERDGAPGETAL&LINK_LOC=protein&PAGE_TYPE=BlastSearch', 'BLAST this protein','Mmcs_4188')" />

 
 
   GGDTETDLKGETQRVRFAEGQYMNAGPARIPQHHVTMDYCRELGVPLEPFVNQNADALLYYTGDTALSNQAVTHRAAKADVYGYVSELLSKATDAGALDG ELSAEDKEALTEFLRDFGDLGNSTGDPAAAGLYLGGSGRRGFDVEPAAGLQSGTVSPAPGLHDVIASGIGREFSFEFGWDQAVMMFQPVGGMDRIAYALE RAVGPNRIRYGSTVTGIRTTPAGADVDYTDAAGVPRTATADFVVNTIPPHIAARIPHNLGPAVTTALQTPVPSPTGKIGLEYTRRWWEDDLRIYGGITNT DLDLAHVWYPSSGFHSDRGVVVGYYNYGQDALDYAALPHSARRQRAVVNGARIHGDVYGRDIASSFSVSWSATRFSEAGWVGWDDQEGPAYRTLLAPQGN VYFAGDHLSHAIAWQHGAMTSARATVTALHQRVMSA', 'http://blast.ncbi.nlm.nih.gov/Blast.cgi?PAGE=Proteins&PROGRAM=blastp&BLAST_PROGRAMS=blastp&QUERY=MPVSRRNFLRGVGATGGAGAMFGAMGALGLAPAAVAAPTAAFTPPRPSDFTLTGRSARNVLVLGAGIAGLTTAYELGKAGYRVTLLEGRARPGGRNWTVR GGDTETDLKGETQRVRFAEGQYMNAGPARIPQHHVTMDYCRELGVPLEPFVNQNADALLYYTGDTALSNQAVTHRAAKADVYGYVSELLSKATDAGALDG ELSAEDKEALTEFLRDFGDLGNSTGDPAAAGLYLGGSGRRGFDVEPAAGLQSGTVSPAPGLHDVIASGIGREFSFEFGWDQAVMMFQPVGGMDRIAYALE RAVGPNRIRYGSTVTGIRTTPAGADVDYTDAAGVPRTATADFVVNTIPPHIAARIPHNLGPAVTTALQTPVPSPTGKIGLEYTRRWWEDDLRIYGGITNT DLDLAHVWYPSSGFHSDRGVVVGYYNYGQDALDYAALPHSARRQRAVVNGARIHGDVYGRDIASSFSVSWSATRFSEAGWVGWDDQEGPAYRTLLAPQGN VYFAGDHLSHAIAWQHGAMTSARATVTALHQRVMSA&LINK_LOC=protein&PAGE_TYPE=BlastSearch', 'BLAST this protein','Krad_0085')" />
   FQTLFSDDHAYLPSSGGYVRVGDAHETLVKEFVAGLPNRQYRENTLLFGAWLDATIRAVAPRQFYDGMHNEIGAELLDLIDGIDLTPYRCGSSGNRIDLH AFYADHPLIRSLCPPHLERFLDDVLDETGSNIIRLRSGMDMLPRRLAAQIRGPISTGREVVGIEIQQDSVLLHVRHGAKTIATSCDYVVCTIPFTVLRAM RLVGFDQDKLDIVHQTKYWPATKVALHCREAFWKKDGISGGASFTGQHVRQTYYPPVDGDPALGAVLLASYSIGPDAEALGRLSEAERNALIIRELSEMH PELRAPGMILGVASRAWGRHRWSLGAATVRWGQGAALRESERREAVRPQRRLFFAGEHCSSKPAWIEGAIESAIDAAHEIEWHELRADREFATTRSSRLG KLA', 'http://blast.ncbi.nlm.nih.gov/Blast.cgi?PAGE=Proteins&PROGRAM=blastp&BLAST_PROGRAMS=blastp&QUERY=MAALRNSGGSESAINGQKRVTVIGAGIAGLVTAYELERLGHRVEIIEASPDVGGRIDTHRFAVDGRPGPFAELGAMRIPAGHRLTRHYIAELGLQDQVHR FQTLFSDDHAYLPSSGGYVRVGDAHETLVKEFVAGLPNRQYRENTLLFGAWLDATIRAVAPRQFYDGMHNEIGAELLDLIDGIDLTPYRCGSSGNRIDLH AFYADHPLIRSLCPPHLERFLDDVLDETGSNIIRLRSGMDMLPRRLAAQIRGPISTGREVVGIEIQQDSVLLHVRHGAKTIATSCDYVVCTIPFTVLRAM RLVGFDQDKLDIVHQTKYWPATKVALHCREAFWKKDGISGGASFTGQHVRQTYYPPVDGDPALGAVLLASYSIGPDAEALGRLSEAERNALIIRELSEMH PELRAPGMILGVASRAWGRHRWSLGAATVRWGQGAALRESERREAVRPQRRLFFAGEHCSSKPAWIEGAIESAIDAAHEIEWHELRADREFATTRSSRLG KLA&LINK_LOC=protein&PAGE_TYPE=BlastSearch', 'BLAST this protein','Sare_2329')" />

 
 
   QFSQFLHGEQGAMVCAAKIVEVVPDLDAKFYAATQTMDEARHVEAFSRFLQEKIGLVYPINKHLTALLDDTLRDSRWDMPYLGMQVLIEGLALAAFGVLR DMAAPESLAKQVLAYVMQDEARHVAFGRISLKDYYSALTEAEREEREEFVVDACYLMRDRFRGEEVFETLGMDVKACAEWVDTSPLMIQFRSHLFSRIVP IVKDIGLWGGKVQRAFRDMGVLDMAGSDIEALMKADEDQAEALDKAHAEMAERAIEVDQVIAAGAS', 'http://blast.ncbi.nlm.nih.gov/Blast.cgi?PAGE=Proteins&PROGRAM=blastp&BLAST_PROGRAMS=blastp&QUERY=MTTRDKYTDVPTPYSWEVPSAGDARFTWEYDEGRARLLSLYQKGKDKQWDAQSRIDWGQDVDPMNAIALPDEFHPLFGSPMWNAADEARRSEMRQHFQAW QFSQFLHGEQGAMVCAAKIVEVVPDLDAKFYAATQTMDEARHVEAFSRFLQEKIGLVYPINKHLTALLDDTLRDSRWDMPYLGMQVLIEGLALAAFGVLR DMAAPESLAKQVLAYVMQDEARHVAFGRISLKDYYSALTEAEREEREEFVVDACYLMRDRFRGEEVFETLGMDVKACAEWVDTSPLMIQFRSHLFSRIVP IVKDIGLWGGKVQRAFRDMGVLDMAGSDIEALMKADEDQAEALDKAHAEMAERAIEVDQVIAAGAS&LINK_LOC=protein&PAGE_TYPE=BlastSearch', 'BLAST this protein','MMAR_0574')" />
   QFRWEYQAWMCSQFLHGEQGALVTTARLVETVPDMDTKTYAASQVADEARHVEAFARYMDEKLGTVYPVNPGLGTLLHDVLSESRWDIVYLGMQVVMEGL AITGLRLASSGFGDPLIRQITKMVASDEARHIAFGVTSLTGMYEQATSAEMREREDFLLESIRLMSRRFMLREVWERMELDVAKGLHFARTNPMMATYRQ LLFQQVIHVLRQLGLLSERVKGLLVAENLVRPEALTSR', 'http://blast.ncbi.nlm.nih.gov/Blast.cgi?PAGE=Proteins&PROGRAM=blastp&BLAST_PROGRAMS=blastp&QUERY=MTAPPDEAADSPFFVLDAVTADLMKVGSLTQACLNWDYGKRDSRVWRLYEKNKAGQWNAATDIDWDHDVRFGAELTEENGARLAGFVVGEGSPVPRELLT QFRWEYQAWMCSQFLHGEQGALVTTARLVETVPDMDTKTYAASQVADEARHVEAFARYMDEKLGTVYPVNPGLGTLLHDVLSESRWDIVYLGMQVVMEGL AITGLRLASSGFGDPLIRQITKMVASDEARHIAFGVTSLTGMYEQATSAEMREREDFLLESIRLMSRRFMLREVWERMELDVAKGLHFARTNPMMATYRQ LLFQQVIHVLRQLGLLSERVKGLLVAENLVRPEALTSR&LINK_LOC=protein&PAGE_TYPE=BlastSearch', 'BLAST this protein','SBI_09252')" />
   MVVAARLVEIVSGLDSKLYATTQAVDEARHVEVFSRYLGEKIQQPYPISEPLELLLTDILEDNRWDVISLGMQIMVEALAMAAFRLAHSTFHDPLIKQIT GLVARDEARHVSFGVLSLRGLYDQMTTRERTEREELVLESAALIRRRFLLGDIWERMEVPMADGLDFAARNELMVAYRQAIFSRVGRALDQIGLMTPRVR EGLVGLDLIQFVEGRRGG', 'http://blast.ncbi.nlm.nih.gov/Blast.cgi?PAGE=Proteins&PROGRAM=blastp&BLAST_PROGRAMS=blastp&QUERY=MSELVVRSTQDARLNWDYESSDSRTESLYQRAKQAQWSVDEIDWHLDVPFGAPLPDDSAFAMAGFAASPLAARGRPAWDEFRWELQAWMVSQFLHGEQAA MVVAARLVEIVSGLDSKLYATTQAVDEARHVEVFSRYLGEKIQQPYPISEPLELLLTDILEDNRWDVISLGMQIMVEALAMAAFRLAHSTFHDPLIKQIT GLVARDEARHVSFGVLSLRGLYDQMTTRERTEREELVLESAALIRRRFLLGDIWERMEVPMADGLDFAARNELMVAYRQAIFSRVGRALDQIGLMTPRVR EGLVGLDLIQFVEGRRGG&LINK_LOC=protein&PAGE_TYPE=BlastSearch', 'BLAST this protein','VAB18032_06985')" />

 
 
   VAATLMRALLSLVVAMVAGYAFGFRITGGLGYAMAFLFIALLLCLAVTLGADAVGSRAKSVQGASHLLFVPQLLLFMLSTGLAPEQTFPAWLQPFVRNQP VSQFAETLRGLAAGRVVLSNATAALAWCAGMVLVFGAITLRMQRRG', 'http://blast.ncbi.nlm.nih.gov/Blast.cgi?PAGE=Proteins&PROGRAM=blastp&BLAST_PROGRAMS=blastp&QUERY=MSVLAALTERSLKSAARDGEMIFEIVSPAAYLAGFTVALHGLIDTGRISYSQYLLPAVVVQSMIVVGLLTADRAARDHLFGFGERMRTLPIAAAATVTAR VAATLMRALLSLVVAMVAGYAFGFRITGGLGYAMAFLFIALLLCLAVTLGADAVGSRAKSVQGASHLLFVPQLLLFMLSTGLAPEQTFPAWLQPFVRNQP VSQFAETLRGLAAGRVVLSNATAALAWCAGMVLVFGAITLRMQRRG&LINK_LOC=protein&PAGE_TYPE=BlastSearch', 'BLAST this protein','MAP_1237c')" />
   RSATDSVQGINRRFRAMPIPSSTPLAARMTASMYRCCIALAVSVGCGYVIGFRFEAGVLGILGFVGLALLIGAALAIIGDLIGVATQNPEATAPMMLIPQ LTLGLASVGLQPVEQFPDWIQGFVRNQPLSQWVYALQALAGDSTDAAPDPTVAVLGSAVAWAVGCIVVALTLHVWVSRRRRS', 'http://blast.ncbi.nlm.nih.gov/Blast.cgi?PAGE=Proteins&PROGRAM=blastp&BLAST_PROGRAMS=blastp&QUERY=MTAQSTVKTAPRRAAIPQAVRPRVSAIQQWWVLTVRMIIPTLRNGELATQVVGSIVFTVGYYLPLKQMMGAVQPLSSYAQYLTPLIVLQAIWFAAISAAF RSATDSVQGINRRFRAMPIPSSTPLAARMTASMYRCCIALAVSVGCGYVIGFRFEAGVLGILGFVGLALLIGAALAIIGDLIGVATQNPEATAPMMLIPQ LTLGLASVGLQPVEQFPDWIQGFVRNQPLSQWVYALQALAGDSTDAAPDPTVAVLGSAVAWAVGCIVVALTLHVWVSRRRRS&LINK_LOC=protein&PAGE_TYPE=BlastSearch', 'BLAST this protein','Mflv_3388')" />
   RFDSMPIPAFTPLAARMSAGLYRCAVGTFAALVSGHIIGFRFHGGLVNAVLFCVLLLAIGLVLSFLADLLGSNSKNPEATSQWLMLPQLIFGLISVGIQP AENFPEWIQPIVRNQPISQFIYALRALGGDTTPGAGEVSWPVIGPSVAWLVGVMVIMVPVSVMLLRRRS', 'http://blast.ncbi.nlm.nih.gov/Blast.cgi?PAGE=Proteins&PROGRAM=blastp&BLAST_PROGRAMS=blastp&QUERY=MTAALTDPRPEPRTVGQWWVLTNRLVSPTLRNGEVATALVASVVFTVGWYIPLNNILGPRSGMSSYAQFLMPLVALQGISFAAITGALRAATDSVKGINR RFDSMPIPAFTPLAARMSAGLYRCAVGTFAALVSGHIIGFRFHGGLVNAVLFCVLLLAIGLVLSFLADLLGSNSKNPEATSQWLMLPQLIFGLISVGIQP AENFPEWIQPIVRNQPISQFIYALRALGGDTTPGAGEVSWPVIGPSVAWLVGVMVIMVPVSVMLLRRRS&LINK_LOC=protein&PAGE_TYPE=BlastSearch', 'BLAST this protein','Mjls_2859')" />
   RFDSMPIPAFTPLAARMSAGLYRCAVGTFAALVSGHIIGFRFHGGLVNAVLFCVLLLAIGLVLSFLADLLGSNSKNPEATSQWLMLPQLIFGLISVGIQP AENFPEWIQPIVRNQPISQFIYALRALGGDTTPGAGEVSWPVIGPSVAWLVGVMVIMVPVSVMLLRRRS', 'http://blast.ncbi.nlm.nih.gov/Blast.cgi?PAGE=Proteins&PROGRAM=blastp&BLAST_PROGRAMS=blastp&QUERY=MTAALTDPRPEPRTVGQWWVLTNRLVSPTLRNGEVATALVASVVFTVGWYIPLNNILGPRSGMSSYAQFLMPLVALQGISFAAITGALRAATDSVKGINR RFDSMPIPAFTPLAARMSAGLYRCAVGTFAALVSGHIIGFRFHGGLVNAVLFCVLLLAIGLVLSFLADLLGSNSKNPEATSQWLMLPQLIFGLISVGIQP AENFPEWIQPIVRNQPISQFIYALRALGGDTTPGAGEVSWPVIGPSVAWLVGVMVIMVPVSVMLLRRRS&LINK_LOC=protein&PAGE_TYPE=BlastSearch', 'BLAST this protein','Mkms_2874')" />
   RFDSMPIPAFTPLAARMSAGLYRCAVGTFAALVSGHIIGFRFHGGLVNAVLFCVLLLAIGLVLSFLADLLGSNSKNPEATSQWLMLPQLIFGLISVGIQP AENFPEWIQPIVRNQPISQFIYALRALGGDTTPGAGEVSWPVIGPSVAWLVGVMVIMVPVSVMLLRRRS', 'http://blast.ncbi.nlm.nih.gov/Blast.cgi?PAGE=Proteins&PROGRAM=blastp&BLAST_PROGRAMS=blastp&QUERY=MTAALTDPRPEPRTVGQWWVLTNRLVSPTLRNGEVATALVASVVFTVGWYIPLNNILGPRSGMSSYAQFLMPLVALQGISFAAITGALRAATDSVKGINR RFDSMPIPAFTPLAARMSAGLYRCAVGTFAALVSGHIIGFRFHGGLVNAVLFCVLLLAIGLVLSFLADLLGSNSKNPEATSQWLMLPQLIFGLISVGIQP AENFPEWIQPIVRNQPISQFIYALRALGGDTTPGAGEVSWPVIGPSVAWLVGVMVIMVPVSVMLLRRRS&LINK_LOC=protein&PAGE_TYPE=BlastSearch', 'BLAST this protein','Mmcs_2830')" />
   RSATDSVQGINRRFRAMPIPSSTPLAARMTASMYRCCIALAVSVGCGYVIGFRFEAGVLGILGFVGLVLLIGAALAIIGDLIGVATQNPEATAPMMLIPQ LTLGLASVGLQPVEQFPDWIQGFVRNQPLSQWVYALQALAGDSTDAAPDATVAVLGSAVAWAAGCIVVALVLHVWVSRRRRS', 'http://blast.ncbi.nlm.nih.gov/Blast.cgi?PAGE=Proteins&PROGRAM=blastp&BLAST_PROGRAMS=blastp&QUERY=MTAQSAVKTAPRQAAIPHAVRPPVSAIQQWWVLTVRMIIPTLRNGELATQVVGSIVFTVGYYLPLKQMMGAVQPLSSYAQYLTPLIMLQAIWFAAISAAF RSATDSVQGINRRFRAMPIPSSTPLAARMTASMYRCCIALAVSVGCGYVIGFRFEAGVLGILGFVGLVLLIGAALAIIGDLIGVATQNPEATAPMMLIPQ LTLGLASVGLQPVEQFPDWIQGFVRNQPLSQWVYALQALAGDSTDAAPDATVAVLGSAVAWAAGCIVVALVLHVWVSRRRRS&LINK_LOC=protein&PAGE_TYPE=BlastSearch', 'BLAST this protein','Mspyr1_27190')" />
   ARYRSMPVNRGALLVGRFLADTVWALVSVAVILLCGFVAGFRFGGGVPAALGFVALAVLFGLVLTAGTSAVGLASRDPESVSAVLNLVYLPLLMLSTAFV PAEAFPGWLEPVIAASPVSVVIDALRALATGEGVAGAVAPALVWTAALGAVFSWAAVRSFRKAV', 'http://blast.ncbi.nlm.nih.gov/Blast.cgi?PAGE=Proteins&PROGRAM=blastp&BLAST_PROGRAMS=blastp&QUERY=MSAPPTAAATERGLGNLLAQGAATAGRNIRARADAGALVTLGVFPAVFVFGFLLLFGRLLGQQGVDYAQFLPPAIIVQWMFSVANSAAPMLAADRRDGLI ARYRSMPVNRGALLVGRFLADTVWALVSVAVILLCGFVAGFRFGGGVPAALGFVALAVLFGLVLTAGTSAVGLASRDPESVSAVLNLVYLPLLMLSTAFV PAEAFPGWLEPVIAASPVSVVIDALRALATGEGVAGAVAPALVWTAALGAVFSWAAVRSFRKAV&LINK_LOC=protein&PAGE_TYPE=BlastSearch', 'BLAST this protein','Ndas_1755')" />
   FSSYAQFMMPLVILQAAAFTAIGAAFRSATDAVAGLDRRFGSMPIGKLVPFGARMSGNVFRLAIALTAALVCGHVIGFRFRLDALHTLGFLALALAIGIA FTVGADVIGTASKSPEATTQALVLPPLILGMLSTALAPATQFPQWVQPFVRNQPISQFAIGLRALAGDTAGNAGVVSWSLLGPSLLWLAGILALALPLAV RFATRRS', 'http://blast.ncbi.nlm.nih.gov/Blast.cgi?PAGE=Proteins&PROGRAM=blastp&BLAST_PROGRAMS=blastp&QUERY=MSGPTPGGGAASARGAARAGAVPVIDAVATEPATPAHRGAWLTDLRARPVRARQWWVLTTRLITPSVKTGEVLTSVFAPAAFTASFYIPLKTVMTFAGTG FSSYAQFMMPLVILQAAAFTAIGAAFRSATDAVAGLDRRFGSMPIGKLVPFGARMSGNVFRLAIALTAALVCGHVIGFRFRLDALHTLGFLALALAIGIA FTVGADVIGTASKSPEATTQALVLPPLILGMLSTALAPATQFPQWVQPFVRNQPISQFAIGLRALAGDTAGNAGVVSWSLLGPSLLWLAGILALALPLAV RFATRRS&LINK_LOC=protein&PAGE_TYPE=BlastSearch', 'BLAST this protein','NFA_11070')" />

 
 
   RVNVGLMHDLVGALHDRRRSTPPVLLYASTAQAANPSAASRYAQQKTEAERILRKATDEGRVRGVILRLPAVYGQSGPSGPMGRGVVAAMIRRALAGEPL TMWHDGGVRRDLLHVEDVATAFAAALEHHDALAGGTWALGADRSEPLGDIFRAVSGSVARQTGSPAVDVVTVPAPEHAEANDFRSDDIDSTEFRSRTGWR PRVSLTDGIDRTVAALTPTEEH', 'http://blast.ncbi.nlm.nih.gov/Blast.cgi?PAGE=Proteins&PROGRAM=blastp&BLAST_PROGRAMS=blastp&QUERY=MNGISDSPRQLITLLGASGFVGSAVLRELRDHPVRLRAVSRGGAPAVPPGAAEVEDLRADLLEPGRAAAAIEDADVIVHLVAHAAGGSTWRSATSDPEAE RVNVGLMHDLVGALHDRRRSTPPVLLYASTAQAANPSAASRYAQQKTEAERILRKATDEGRVRGVILRLPAVYGQSGPSGPMGRGVVAAMIRRALAGEPL TMWHDGGVRRDLLHVEDVATAFAAALEHHDALAGGTWALGADRSEPLGDIFRAVSGSVARQTGSPAVDVVTVPAPEHAEANDFRSDDIDSTEFRSRTGWR PRVSLTDGIDRTVAALTPTEEH&LINK_LOC=protein&PAGE_TYPE=BlastSearch', 'BLAST this protein','SACE_0720')" />

 
 
   VALYVALTAAARFTNRLNTYAVQLLQHRLRATLSTRVLHSDGSAVRAPADNVVSAMTNDVFRLANAGLLVVLPISRIAAIMFIAVSLLVMHWPLGVMVLL GAPVAVWLMGLLSERLSRDTREYQDLLADTVGRATDLVAGYRVIKGVRAETEATRRYRQASRETLVGAGRNAGLLGRFLVGSGAVNGVFVAAVTGLAGWF AVNGQLSVGELIAAGGLTQALLPQMQAIASYSIPNLAGARASAARIMDVFRNAGTAASGHDDAIRQRCTPPPVQDVSAPYVGVLDIGVPSASIRVEPGEL VGVHADDRIAARIADALLNPWAANDIEVRLDGRPAHELTPAEYRSLVTAAPHRATLFTGTIRDNLAVTACLPERMDSAVWAAACEDFAADLDTPVGENGN RLSGGQRQRVALARAFATDTPVLVLHDPTTAVDSVTEQTIAKRLVGIRIDRSTLLIASSPALLGCCDRVVGLLDNMPVLGKAAL', 'http://blast.ncbi.nlm.nih.gov/Blast.cgi?PAGE=Proteins&PROGRAM=blastp&BLAST_PROGRAMS=blastp&QUERY=MRTDPMELGRGPLTRWLPVLGQAAPEPPDVRSFEVREGDRPGQFVARVIFSLPRITIPAMLLAIVWQVGESAVPVVMGLAIDRALVTRDAGQLVLWLGVL VALYVALTAAARFTNRLNTYAVQLLQHRLRATLSTRVLHSDGSAVRAPADNVVSAMTNDVFRLANAGLLVVLPISRIAAIMFIAVSLLVMHWPLGVMVLL GAPVAVWLMGLLSERLSRDTREYQDLLADTVGRATDLVAGYRVIKGVRAETEATRRYRQASRETLVGAGRNAGLLGRFLVGSGAVNGVFVAAVTGLAGWF AVNGQLSVGELIAAGGLTQALLPQMQAIASYSIPNLAGARASAARIMDVFRNAGTAASGHDDAIRQRCTPPPVQDVSAPYVGVLDIGVPSASIRVEPGEL VGVHADDRIAARIADALLNPWAANDIEVRLDGRPAHELTPAEYRSLVTAAPHRATLFTGTIRDNLAVTACLPERMDSAVWAAACEDFAADLDTPVGENGN RLSGGQRQRVALARAFATDTPVLVLHDPTTAVDSVTEQTIAKRLVGIRIDRSTLLIASSPALLGCCDRVVGLLDNMPVLGKAAL&LINK_LOC=protein&PAGE_TYPE=BlastSearch', 'BLAST this protein','Franean1_3048')" />

 
 
   ANTAMQTAIGESTYPVMSSMKWQKTFHASIASPLGSFDVFVGRLLFVGFRVLMNCAIFLAVMALFGAVHAAGPGGLGPVLAVPAAALTGLAFAAPVIAWA VTQDRDTGFSVVFRFVMIPLFLFSGTFFPVTQLPAAIRPLAYATPLWHGVDLCRGLALGTASAGSVLLHLAYLFAVIGAGLWYGAHTFRRRLNP', 'http://blast.ncbi.nlm.nih.gov/Blast.cgi?PAGE=Proteins&PROGRAM=blastp&BLAST_PROGRAMS=blastp&QUERY=MSTLTPASSGPPVRSAPPGSAASVRGTPLMAVRELRGLLTNYCRTWRGSIISSVLAPLLSLVALGMSLGKIVDAGPGAHSFGTVDGEPVSYLLFLAPALL ANTAMQTAIGESTYPVMSSMKWQKTFHASIASPLGSFDVFVGRLLFVGFRVLMNCAIFLAVMALFGAVHAAGPGGLGPVLAVPAAALTGLAFAAPVIAWA VTQDRDTGFSVVFRFVMIPLFLFSGTFFPVTQLPAAIRPLAYATPLWHGVDLCRGLALGTASAGSVLLHLAYLFAVIGAGLWYGAHTFRRRLNP&LINK_LOC=protein&PAGE_TYPE=BlastSearch', 'BLAST this protein','Caci_1803')" />

 
 
   QVEPDEDVLEDGIAFAGVAKYPARVKCALLGWMAFKDAVSRSEGAKS', 'http://blast.ncbi.nlm.nih.gov/Blast.cgi?PAGE=Proteins&PROGRAM=blastp&BLAST_PROGRAMS=blastp&QUERY=MQLQQMYQEIILDHYKNPHGRGLRDPYDAESHQINPTCGDEVTLRVKLDGPLVADVSYDGQGCSISQAATSVLTDLVVGKPVEQAMGKLDAFVELMQGRG QVEPDEDVLEDGIAFAGVAKYPARVKCALLGWMAFKDAVSRSEGAKS&LINK_LOC=protein&PAGE_TYPE=BlastSearch', 'BLAST this protein','Amir_5149')" />
   TIEGDEDLLGDGIAFAGVAKYPARVKCALLGWMAFKDAVVQITEGTSREGQQ', 'http://blast.ncbi.nlm.nih.gov/Blast.cgi?PAGE=Proteins&PROGRAM=blastp&BLAST_PROGRAMS=blastp&QUERY=MRLDQMYQEVILDHYKHPHHRGLREPFGAEVHHVNPTCGDEITLRVHVERDAVADISYDGQGCSISQASTSVLTDQLIGLPLGDALKIVGAFQEMIGSRG TIEGDEDLLGDGIAFAGVAKYPARVKCALLGWMAFKDAVVQITEGTSREGQQ&LINK_LOC=protein&PAGE_TYPE=BlastSearch', 'BLAST this protein','AS9A_1907')" />
   RGRIEGDDEIIGDGVAFSGVSKYPARVKCALLGWKAFEAASIDAGVEPPASE', 'http://blast.ncbi.nlm.nih.gov/Blast.cgi?PAGE=Proteins&PROGRAM=blastp&BLAST_PROGRAMS=blastp&QUERY=MKLEQMYQEVILDHYKHPQHAGLRDPFDAEVHHVNTSCGDELTLRVKISEDGKTVEDVSYEAIGCSISQASTSVMAEEIVGQPVEEAFAKLAEFEKMVTS RGRIEGDDEIIGDGVAFSGVSKYPARVKCALLGWKAFEAASIDAGVEPPASE&LINK_LOC=protein&PAGE_TYPE=BlastSearch', 'BLAST this protein','CRES_1062')" />
   RGKLEGDEDLIGDGIAFSGVSKYPARVKCALLGWKAFEAAAIDAGVTPPEQSEDA', 'http://blast.ncbi.nlm.nih.gov/Blast.cgi?PAGE=Proteins&PROGRAM=blastp&BLAST_PROGRAMS=blastp&QUERY=MRMEQMYQEVILDHYKHPEHAGLREPYNAEVFHVNTSCGDELTLRVHLSEDHKTVQDISYDAVGCSISQASTSVMADEIVGKPVTEAFAKLAEFEKMITS RGKLEGDEDLIGDGIAFSGVSKYPARVKCALLGWKAFEAAAIDAGVTPPEQSEDA&LINK_LOC=protein&PAGE_TYPE=BlastSearch', 'BLAST this protein','cu_1011')" />
   GDEDLIGDGIAFAGVSRYPARVKCALLGWKAFEAATLDAGIPHPSPAH', 'http://blast.ncbi.nlm.nih.gov/Blast.cgi?PAGE=Proteins&PROGRAM=blastp&BLAST_PROGRAMS=blastp&QUERY=MYQEVILDHYKNPQHAGLREPFEAEVHHVNTSCGDEVTLRVHLSDDQKTVEDVSYDAQGCSISQASTSVMAEEIIGLPVEQAFAKLAEFEKMVTSRGEEE GDEDLIGDGIAFAGVSRYPARVKCALLGWKAFEAATLDAGIPHPSPAH&LINK_LOC=protein&PAGE_TYPE=BlastSearch', 'BLAST this protein','CVAR_1463')" />
   AFTELMQSKGKGEPDEDVLEDAVAFVGVSKYPARIKCALLGWMAWKDAVSQSLEKEAV', 'http://blast.ncbi.nlm.nih.gov/Blast.cgi?PAGE=Proteins&PROGRAM=blastp&BLAST_PROGRAMS=blastp&QUERY=MQLDAMYQEIILDHYRNPHHKGLRDPHNAEAHHINPTCGDEVTLRVALTPAEGSGGLDREAVVSDVSYEGMGCSISQASTSVLTDLLIGRTVAEGMTTLD AFTELMQSKGKGEPDEDVLEDAVAFVGVSKYPARIKCALLGWMAWKDAVSQSLEKEAV&LINK_LOC=protein&PAGE_TYPE=BlastSearch', 'BLAST this protein','Ndas_2958')" />
   VSSRGTVEGDEDVLGDGIAFSGVSKYPARVKCALLGWMAFKDAVVRIVDDENDLARTGGQTS', 'http://blast.ncbi.nlm.nih.gov/Blast.cgi?PAGE=Proteins&PROGRAM=blastp&BLAST_PROGRAMS=blastp&QUERY=MRLEQMYQEVILDHYKHPHGRGLRDPFGAEVHHVNPTCGDEITLRAHLTEGEDGRPVVADISYDGQGCSISQASTSVLFDQIVGMPLDEALRTVDAFNEM VSSRGTVEGDEDVLGDGIAFSGVSKYPARVKCALLGWMAFKDAVVRIVDDENDLARTGGQTS&LINK_LOC=protein&PAGE_TYPE=BlastSearch', 'BLAST this protein','REQ_22310')" />
   GTVEGDEDVLGDGIAFVGVSKYPARVKCALLGWMAFKDAVVQIVDGSAVTTADSSSELVDTTEGQGS', 'http://blast.ncbi.nlm.nih.gov/Blast.cgi?PAGE=Proteins&PROGRAM=blastp&BLAST_PROGRAMS=blastp&QUERY=MRMEQMYQEVILDHYKHPHGRGLREPFGAEVHHVNPTCGDEVTLRVQISEDGTVSDVSYDGQGCSISQASTSVLNDLVIGMQVTDALKTVTAFNEMVSSR GTVEGDEDVLGDGIAFVGVSKYPARVKCALLGWMAFKDAVVQIVDGSAVTTADSSSELVDTTEGQGS&LINK_LOC=protein&PAGE_TYPE=BlastSearch', 'BLAST this protein','RER_30590')" />
   GTVEGDEDVLGDGIAFVGVSKYPARVKCALLGWMAFKDAVVQIVDDRVVDERDSAVPTADGEPARIGGQAS', 'http://blast.ncbi.nlm.nih.gov/Blast.cgi?PAGE=Proteins&PROGRAM=blastp&BLAST_PROGRAMS=blastp&QUERY=MRMEQMYQEVILDHYKHPHGRGLREPFGAEVHHVNPTCGDEVTLRVQLADDGTVVDVSYDGQGCSISQASTSVLTDQVVGMPVEQALQTVTAFNEMVSSR GTVEGDEDVLGDGIAFVGVSKYPARVKCALLGWMAFKDAVVQIVDDRVVDERDSAVPTADGEPARIGGQAS&LINK_LOC=protein&PAGE_TYPE=BlastSearch', 'BLAST this protein','RHA1_ro07200')" />
   GTVEGDEDVLGDGIAFVGVSKYPARVKCALLGWMAFKDAVVQIVDEQDSAVPTADGEPARIGGQAS', 'http://blast.ncbi.nlm.nih.gov/Blast.cgi?PAGE=Proteins&PROGRAM=blastp&BLAST_PROGRAMS=blastp&QUERY=MRMEQMYQEVILDHYKHPHGRGLREPFGAEVHHVNPTCGDEVTLRVQLADDGTVVDVSYDGQGCSISQASTSVLTDQVVGMPVEQALQTVAAFNEMVSSR GTVEGDEDVLGDGIAFVGVSKYPARVKCALLGWMAFKDAVVQIVDEQDSAVPTADGEPARIGGQAS&LINK_LOC=protein&PAGE_TYPE=BlastSearch', 'BLAST this protein','ROP_69880')" />
   QGRGQVEPDEDVLEDGIAFAGVAKYPMRVKCALLGWMAFKDAVSSVGVEEAS', 'http://blast.ncbi.nlm.nih.gov/Blast.cgi?PAGE=Proteins&PROGRAM=blastp&BLAST_PROGRAMS=blastp&QUERY=MQLEQMYQEIILDHYKNPHRHGLREPYDAESFQVNPTCGDEITLRVRLDGEGSDAIVEDVSYAGQGCSISQASASVLTDLVVGRTVGEAFKTQAAFGEMM QGRGQVEPDEDVLEDGIAFAGVAKYPMRVKCALLGWMAFKDAVSSVGVEEAS&LINK_LOC=protein&PAGE_TYPE=BlastSearch', 'BLAST this protein','SACE_2178')" />
   TALMQSKGQGEPDEDVLQDAVAFAGVSKYPARVKCALLAWMAWKDATAKSLATQRQEEAV', 'http://blast.ncbi.nlm.nih.gov/Blast.cgi?PAGE=Proteins&PROGRAM=blastp&BLAST_PROGRAMS=blastp&QUERY=MRLDAMYQEIILDHYRNPHHKGLREPFDAESHHVNPTCGDEITLRVLLKPADKESDGSAAIADISYDSMGCSISQASASVMADLLIGKTVDEAMAILDEF TALMQSKGQGEPDEDVLQDAVAFAGVSKYPARVKCALLAWMAWKDATAKSLATQRQEEAV&LINK_LOC=protein&PAGE_TYPE=BlastSearch', 'BLAST this protein','Tfu_1982')" />

 
 
   LGTSLGGARSGEEFHRQWIQDGLRKANTGLLRQYPLHSVADHLASEFQLFGPRSVQSNACAAGAVAIAYGIELLESDAADMVLAGGVDPLALLSFGGFSS LKALDPLHCAPYTRSSGLNLGEGAGFLVLESEASAVGRGAAIYAEIAGYGLSADAYHATAPDPMGRGALRAMHAALHMAGNTVEDVDYVNGHGTGTAAND SVEAKAITHLREGAAPPASSTKSMIGHTLGAAGAIEAVTSVLAIQNQEMPPTVTPDGLRSPTGLDIVAETAQRARLKTVLSNSFAFGGNNASLAIREYTK DASPRAPKTVRPVVITGIGALAGDAVTTEEVKTALFEGRPVYGDGTVEVDGFGSFPFGDIPTDKLKKGIDPKYLRHMDTLGRRSALAVSQLLKQRGLSRA ESTSTGLLFATGTGPISTVEAFERELLTTGSGNALLFPNTVMNAAPGHVALLNKLQGPTATICAGNTGAITALHFAQNLISNGVVDRMIVLAADEAPHAM LAAYAPIPGYLARSACLPYSNTGRIISGAAVAILLESEDSVDPGRVLGRIEHFGMTGDGSGPSRLRRGSEAWVRSFRLALGSNPPETVDAVVAAACGRDA VDSLEVEALAAMGLGGRPVSTPKAIFGDAGASGGLLGVAQAVWMSQEKFIPGTAGVVASGPEGLVPPSGKAGEVNRTLVSTCEAGGSFQSVTIAT', 'http://blast.ncbi.nlm.nih.gov/Blast.cgi?PAGE=Proteins&PROGRAM=blastp&BLAST_PROGRAMS=blastp&QUERY=MKIVVTGFGLRAAVGSNAEQSWESISNGKSGIVRTTVVPTEGLVSSMGGQVESEMETEPPSSRTTYVDRCHRLASAAATEALTHAGANQAPVDRTRIALS LGTSLGGARSGEEFHRQWIQDGLRKANTGLLRQYPLHSVADHLASEFQLFGPRSVQSNACAAGAVAIAYGIELLESDAADMVLAGGVDPLALLSFGGFSS LKALDPLHCAPYTRSSGLNLGEGAGFLVLESEASAVGRGAAIYAEIAGYGLSADAYHATAPDPMGRGALRAMHAALHMAGNTVEDVDYVNGHGTGTAAND SVEAKAITHLREGAAPPASSTKSMIGHTLGAAGAIEAVTSVLAIQNQEMPPTVTPDGLRSPTGLDIVAETAQRARLKTVLSNSFAFGGNNASLAIREYTK DASPRAPKTVRPVVITGIGALAGDAVTTEEVKTALFEGRPVYGDGTVEVDGFGSFPFGDIPTDKLKKGIDPKYLRHMDTLGRRSALAVSQLLKQRGLSRA ESTSTGLLFATGTGPISTVEAFERELLTTGSGNALLFPNTVMNAAPGHVALLNKLQGPTATICAGNTGAITALHFAQNLISNGVVDRMIVLAADEAPHAM LAAYAPIPGYLARSACLPYSNTGRIISGAAVAILLESEDSVDPGRVLGRIEHFGMTGDGSGPSRLRRGSEAWVRSFRLALGSNPPETVDAVVAAACGRDA VDSLEVEALAAMGLGGRPVSTPKAIFGDAGASGGLLGVAQAVWMSQEKFIPGTAGVVASGPEGLVPPSGKAGEVNRTLVSTCEAGGSFQSVTIAT&LINK_LOC=protein&PAGE_TYPE=BlastSearch', 'BLAST this protein','AAur_2336')" />
   DTAGVGTTGVGTTSVTGVGTAAGAGGTAGAAGAPVTLLPAQRWGVVLGSCNGGLVSGERALRDELAGRQPDWRHTLVVSPQAIAHATELIVTGQADAVLV GGTDAFSDVVFAGFNSLESLAVGPAAPYSKDRDGLSLGEGAGMLVLVRADIARAAGARVFAEVLGYGFSADGYHPTAPHPQGAGAARAITAALSRAGVAA DEVGYVNGHGTGTPKNDPAESNAIRAALGEAAGTVALSSTKSMVGHLLGAAGAVESIVTILALDEQVAPPTAGFTGVDPQCGLDVVPNEARPLAMDVAVS NNFAFAGANASVVFGRPGRRAAAPAGQDRDRQRQREREPIVITGLAALTPAGTSVAELWEAYQAAGSASGADASQAAGHIGVGHVEFDPAAYLPARQRRR LDRLGLLAVASCQDALADAGLADVARPATGIGVILGTGLGPMESMERFTTPLLTEGPAAANPAVFPNTVYNAAAGQVAMLLGLTGVTSTVTAMHAAGAAA LCVAADLLRAGAADAIVCPAVDVLPPAVVNAYRRTPLFAGAGGAYTLAEAGFALVLERLSVARARGARIRAVVRGHGIASDARGVGRWDARGRGVEQAVR TALAAAGVEPGALSAVWANAAGIRAVDRPERAALGRVFGPAGPRVETPKRSLGEPVGAGAHLSAVLAVAGWEAGGAAGPVLVNSSSLGGTHVSLVLTGPG HGDHAGPEVNDHVFHAADAAGSRKEQ', 'http://blast.ncbi.nlm.nih.gov/Blast.cgi?PAGE=Proteins&PROGRAM=blastp&BLAST_PROGRAMS=blastp&QUERY=MAEGTQTAGDADRVVVTGLGAVTAQGVGVAKLWEGVRAGQVAIRPVKNLPMDGYRTALGGEVQEPAIPAHPWPLEVSREPALDFALVAAEEAMAACGIGV DTAGVGTTGVGTTSVTGVGTAAGAGGTAGAAGAPVTLLPAQRWGVVLGSCNGGLVSGERALRDELAGRQPDWRHTLVVSPQAIAHATELIVTGQADAVLV GGTDAFSDVVFAGFNSLESLAVGPAAPYSKDRDGLSLGEGAGMLVLVRADIARAAGARVFAEVLGYGFSADGYHPTAPHPQGAGAARAITAALSRAGVAA DEVGYVNGHGTGTPKNDPAESNAIRAALGEAAGTVALSSTKSMVGHLLGAAGAVESIVTILALDEQVAPPTAGFTGVDPQCGLDVVPNEARPLAMDVAVS NNFAFAGANASVVFGRPGRRAAAPAGQDRDRQRQREREPIVITGLAALTPAGTSVAELWEAYQAAGSASGADASQAAGHIGVGHVEFDPAAYLPARQRRR LDRLGLLAVASCQDALADAGLADVARPATGIGVILGTGLGPMESMERFTTPLLTEGPAAANPAVFPNTVYNAAAGQVAMLLGLTGVTSTVTAMHAAGAAA LCVAADLLRAGAADAIVCPAVDVLPPAVVNAYRRTPLFAGAGGAYTLAEAGFALVLERLSVARARGARIRAVVRGHGIASDARGVGRWDARGRGVEQAVR TALAAAGVEPGALSAVWANAAGIRAVDRPERAALGRVFGPAGPRVETPKRSLGEPVGAGAHLSAVLAVAGWEAGGAAGPVLVNSSSLGGTHVSLVLTGPG HGDHAGPEVNDHVFHAADAAGSRKEQ&LINK_LOC=protein&PAGE_TYPE=BlastSearch', 'BLAST this protein','FsymDg_3052')" />
   LGTCNAGLLSARAWLDAARRGAAPDPRLPLLATPQALAESVAAAYGLRGPVLAVNTACASGANAIGLGADLVRQGRADAVLAGGTDAFSDVVFAGFNALE SLSPEPAAPYSADRQGLSLGEGSGMVLLVSTGFAAAHGLRPLAEVAGYGLSADGYHVTAPHPEGAGAARAIDSALRRSGVAPEDIGYVNGHGTGTPKNDP AESAAIARALGESAAHTPVSSTKSVIGHLLGAAGAVEAIVTAHALDEQLAPPTAGYTRSAPECPLDYVPGAARPLHTDAALSNNFAFAGANAALVLTRPG THTPPVPDWDRVVVTGVSVLGPAGDGIQAARRALRAGQEAGSLEGGLRLGRVALGDEEIAPYLTRRQRRRMDRLAVLSIVATAKALADAGLSADESVGVV FGTGTGPMEAMEKFVLPLLEEGAAAADPSVFPNTVYNQAAGQVATHLGLRGPTSTLSVGHATGAAAVAYTADLLAAGHADALVCTVTDTLTEQVARAYTA TGAASTRPPGAPADGRFTLAEGSVALVMERLSAARARGATVLGEVLGHGMASDASRARLWDPRGRGMELAMRRALADAGLTPRDVGGVWLSAAGLTAADR AESAAVERVFGSDTAPPSHAPKSVLGEPMGVGGALCLALSLWSGDTRPAGPAVVNSSSLGGSHVSLVVAAGDPHGTGDPGGPDNDEGVNQ', 'http://blast.ncbi.nlm.nih.gov/Blast.cgi?PAGE=Proteins&PROGRAM=blastp&BLAST_PROGRAMS=blastp&QUERY=MAVVGTGALTSQGAGAAALWEGARAGRVAIRPVQHIDMRGLATKLGGEVEGARTAPRGYRRPEGHRERALDLALAAAEEALAALAALPEGTVAPTRFGLV LGTCNAGLLSARAWLDAARRGAAPDPRLPLLATPQALAESVAAAYGLRGPVLAVNTACASGANAIGLGADLVRQGRADAVLAGGTDAFSDVVFAGFNALE SLSPEPAAPYSADRQGLSLGEGSGMVLLVSTGFAAAHGLRPLAEVAGYGLSADGYHVTAPHPEGAGAARAIDSALRRSGVAPEDIGYVNGHGTGTPKNDP AESAAIARALGESAAHTPVSSTKSVIGHLLGAAGAVEAIVTAHALDEQLAPPTAGYTRSAPECPLDYVPGAARPLHTDAALSNNFAFAGANAALVLTRPG THTPPVPDWDRVVVTGVSVLGPAGDGIQAARRALRAGQEAGSLEGGLRLGRVALGDEEIAPYLTRRQRRRMDRLAVLSIVATAKALADAGLSADESVGVV FGTGTGPMEAMEKFVLPLLEEGAAAADPSVFPNTVYNQAAGQVATHLGLRGPTSTLSVGHATGAAAVAYTADLLAAGHADALVCTVTDTLTEQVARAYTA TGAASTRPPGAPADGRFTLAEGSVALVMERLSAARARGATVLGEVLGHGMASDASRARLWDPRGRGMELAMRRALADAGLTPRDVGGVWLSAAGLTAADR AESAAVERVFGSDTAPPSHAPKSVLGEPMGVGGALCLALSLWSGDTRPAGPAVVNSSSLGGSHVSLVVAAGDPHGTGDPGGPDNDEGVNQ&LINK_LOC=protein&PAGE_TYPE=BlastSearch', 'BLAST this protein','SBI_02599')" />
[truncated: 15,849,048 more chars]
