# Supplementary material for: Comparative genomics of actinomycetes with a focus on natural product biosynthetic genes
Source: BMC Genomics. 2013 Sep 11;14:611. doi: 10.1186/1471-2164-14-611 (PMC3848822; doi:10.1186/1471-2164-14-611)
Supplement: Additional file 1 — A stand-alone website showing all natural product gene clusters analyzed in this study, along with separate files for conserved clusters mentioned in the text and pHMM files. Use of the HTML files requires Javascript. Homologous genes are shown in the same color. All homologous genes on a page are highlighted upon mouseover of any of them. Mouseover also produces a description containing the locus tag and annotation for each gene. Mouseover for a domain box above the gene arrows shows the domain name. Clicking on a gene arrow produces a page with the amino acid sequence and a link to BLAST the nr protein database. [file 1471-2164-14-611-S1.zip › website/allcompletelarge.html]

allcompletelarge


AMED\_2
AMED\_7
Amir\_5
Amir\_10
Amir\_13
Caci\_6
Caci\_27
FRAAL\_1
FRAAL\_8
FRAAL\_15
FraEuI1c\_10
FraEuI1c\_15
Francci3\_15
Gbro\_2
Gbro\_9
Kfla\_7
MAB\_7
MAF\_10
MAV\_11
Mb\_10
MCAN\_10
Micau\_3
Micau\_11
ML5\_10
ML5\_18
MLBr\_5
MMAR\_10
MMAR\_20
MMAR\_24
MUL\_5
Namu\_3
NFA\_2
NFA\_16
NFA\_17
RAM\_2
RAM\_7
REQ\_11
RER\_5
RHA1\_ro\_4
RHA1\_ro\_19
RHA1\_ro\_21
ROP\_1
ROP\_18
Rv\_10
SACE\_10
SACE\_21
SACE\_23
SACTE\_18
Sare\_1
Sare\_5
Sare\_21
SAV\_3
SAV\_6
SAV\_19
SBI\_6
SBI\_9
SBI\_19
SBI\_25
SBI\_47
SBI\_54
SCAB\_29
SCO\_11
SCO\_21
Sfla\_9
SGR\_4
SGR\_22
SGR\_31
SGR\_32
SGR\_33
SGR\_39
Sros\_10
Srot\_6
Strop\_12
Strvi\_7
Strvi\_11
Strvi\_13
Strvi\_14
Strvi\_21
Strvi\_24
Strvi\_25
Strvi\_30
Strvi\_37
Strvi\_40
Svir\_3
VAB18032\_8
VAB18032\_19

Tooltip
