# Supplementary material for: Comparative genomics of actinomycetes with a focus on natural product biosynthetic genes
Source: BMC Genomics. 2013 Sep 11;14:611. doi: 10.1186/1471-2164-14-611 (PMC3848822; doi:10.1186/1471-2164-14-611)
Supplement: Additional file 1 — A stand-alone website showing all natural product gene clusters analyzed in this study, along with separate files for conserved clusters mentioned in the text and pHMM files. Use of the HTML files requires Javascript. Homologous genes are shown in the same color. All homologous genes on a page are highlighted upon mouseover of any of them. Mouseover also produces a description containing the locus tag and annotation for each gene. Mouseover for a domain box above the gene arrows shows the domain name. Clicking on a gene arrow produces a page with the amino acid sequence and a link to BLAST the nr protein database. [file 1471-2164-14-611-S1.zip › website/Conserved Clusters/mycolicacid.html]

mycolicacid.txt


MAF\_12
Mb\_12
Rv\_12
MCAN\_12
MLBr\_1
MMAR\_27
MUL\_9
MAV\_1
MAP\_1
JDM601\_8
MSMEG\_11
Mspyr1\_10
Mflv\_3
Mjls\_10
Mkms\_10
Mmcs\_10
MAB\_1
Gbro\_1
Tpau\_1
NFA\_22
REQ\_1
RER\_1
ROP\_13
RHA1\_ro\_8
Cgl\_3
CE\_2
DIP\_5
CULC22\_3
cauri\_2
CVAR\_1
cu\_1
CRES\_2
jk\_1
AS9A\_1
Srot\_8

Tooltip
