# Supplementary figures and images for: Comparative genomics of actinomycetes with a focus on natural product biosynthetic genes
Source: BMC Genomics. 2013 Sep 11;14:611. doi: 10.1186/1471-2164-14-611 (PMC3848822; doi:10.1186/1471-2164-14-611)

0.01

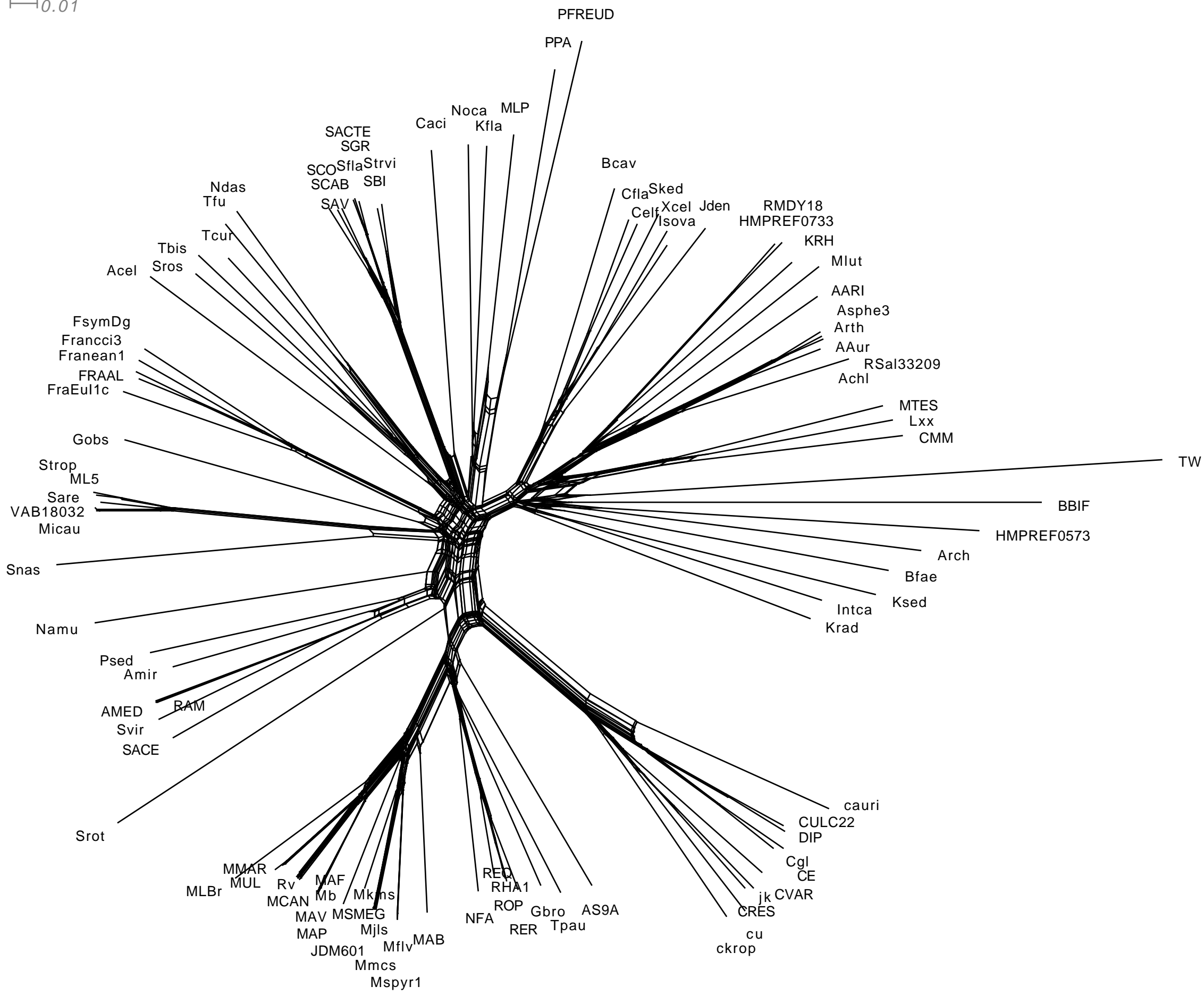

Supplement: Additional file 2 — A NeighborNet analysis on concatenated ribosomal proteins. [file 1471-2164-14-611-S2.pdf]
