# Supplementary material for: Comparative genomics of actinomycetes with a focus on natural product biosynthetic genes
Source: BMC Genomics. 2013 Sep 11;14:611. doi: 10.1186/1471-2164-14-611 (PMC3848822; doi:10.1186/1471-2164-14-611)
Supplement: Additional file 3 — List of genes grouped together within the genera of interest, gene range is separated by commas and gene groups are separated by semicolons. [file 1471-2164-14-611-S3.docx]

List of genes grouped together within the genera of interest, gene range is separated by commas and gene groups are separated by semicolons: jk0132-jk0144, CULC22_02180-CULC22_02192, DIP_2181-DIP_2195, cu_0154-cu_0166, CVAR_0248-CVAR_0259, Cgl_2865-Cgl_2876, cauri_2362-cauri_2374, CE_2695-CE_2707, CRES_0529-CRES_0542; DIP_0580-DIP_0594, CULC22_01053-CULC22_01065, CRES_0446-CRES_0458; cu_0323-cu_0335, jk1773-jk1785; SBI_06737-SBI_06753, SAV_3654-SAV_3671, Strvi_8796-Strvi_8809, Strvi_8191-Strvi_8207; SGR_1713-SGR_1727, SACTE_4955-SACTE_4969, Sfla_1550-Sfla_1564, SBI_03310-SBI_03322, SAV_2460-SAV_2472, SCO_5794-SCO_5806, SCAB_24621-SCAB_24751, Strvi_2120-Strvi_2132; SCAB_43231-SCAB_43371, SBI_02758-SBI_02771, Strvi_2536-Strvi_2549, SCO_5311-SCO_5324, SAV_2832-SAV_2845, Sfla_6013-Sfla_6034, SACTE_0239-SACTE_0262; Strvi_1054-Strvi_1072, SAV_5263-SAV_5275, SGR_4741-SGR_4753, SCO_2779-SCO_2791, Sfla_4098-Sfla_4111, SBI_06439-SBI_06459, SCAB_57851-SCAB_57981; SGR_1338-SGR_1350, SACTE_5415-SACTE_5427, Sfla_1074-Sfla_1086; SGR_4799-SGR_4815, Sfla_4154-Sfla_4170, SACTE_2210-SACTE_2226; SCAB_1411-SCAB_1551, SACTE_0734-SACTE_0747, SCO_7665-SCO_7689; SCAB_69761-SCAB_69881, SBI_01603-SBI_01627; SAV_7497-SAV_7509, SGR_2391-SGR_2403, SCAB_8551-SCAB_8681, SCO_6675-SCO_6687; SAV_7125-SAV_7137, SCO_1200-SCO_1212; SCO_5081-SCO_5094, SACTE_6340-SACTE_6353; SCO_6220-SCO_6233, SAV_1997-SAV_2010, SCAB_18331-SCAB_18461; SGR_3840-SGR_3853, SCAB_31981-SCAB_32101, Strvi_1308-Strvi_1322; Sfla_0301-Sfla_0314, SGR_6710-SGR_6722, SCO_0486-SCO_0498, SACTE_0626-SACTE_0638; SACTE_5195-SACTE_5216, SAV_2362-SAV_2382; SACTE_5570-SACTE_5588, Sfla_0972-Sfla_0990; SACTE_6100-SACTE_6112, Sfla_0136-Sfla_0148, SGR_808-SGR_820; SBI_09189-SBI_09201, SAV_1545-SAV_1557; SACTE_5054-SACTE_5068, Sfla_1328-Sfla_1341; SAV_7315-SAV_7328, Strvi_6796-Strvi_6809, SBI_08286-SBI_08299; SAV_7355-SAV_7367, Strvi_6294-Strvi_6307, SBI_00974-SBI_00986, Strvi_5226-Strvi_5238; SCO_0263-SCO_0276, SBI_06943-SBI_06956; SBI_08394-SBI_08425, Strvi_3951-Strvi_3978; SAV_932-SAV_949, SBI_00720-SBI_00739; FraEuI1c_6939-FraEuI1c_6952, Francci3_2026-Francci3_2039, Francci3_1120-Francci3_1134, Franean1_6748-Franean1_6761, FraEuI1c_0873-FraEuI1c_0885, Francci3_1815-Francci3_1828, Franean1_1326-Franean1_1339, Franean1_0049-Franean1_0062, FRAAL_6336-FRAAL_6350, Francci3_3308-Francci3_3321, FRAAL_2693-FRAAL_2707, Franean1_2793-Franean1_2806; Franean1_5934-Franean1_5948, FraEuI1c_4523-FraEuI1c_4541; FraEuI1c_1311-FraEuI1c_1324, Francci3_4094-Francci3_4107; FsymDg_1311-FsymDg_1325, FraEuI1c_1553-FraEuI1c_1566, FRAAL_2907-FRAAL_2919, FRAAL_1542-FRAAL_1555, Francci3_0920-Francci3_0932, Franean1_5607-Franean1_5621, FraEuI1c_1209-FraEuI1c_1221; FsymDg_2761-FsymDg_2774, Francci3_2844-Francci3_2857, FraEuI1c_4746-FraEuI1c_4759, Franean1_2387-Franean1_2400, FRAAL_4381-FRAAL_4393; FRAAL_6417-FRAAL_6431, Francci3_4050-Francci3_4064, Franean1_0652-Franean1_0666, FsymDg_0703-FsymDg_0716; RHA1_ro05446-RHA1_ro05458, ROP_55160-ROP_55280; ROP_51570-ROP_51700, RHA1_ro05096-RHA1_ro05109; RHA1_ro02391-RHA1_ro02403, ROP_21030-ROP_21150; ROP_20310-ROP_20430, RHA1_ro02313-RHA1_ro02325; RHA1_ro06097-RHA1_ro06109, ROP_61520-ROP_61640; ROP_48070-ROP_48190, REQ_07570-REQ_07690, RHA1_ro04709-RHA1_ro04722; ROP_02520-ROP_02670, RHA1_ro00135-RHA1_ro00150; ROP_48810-ROP_48950, REQ_08080-REQ_08220; RER_35670-RER_35790, REQ_27950-REQ_28061, ROP_08310-ROP_08430, RHA1_ro01103-RHA1_ro01115; RHA1_ro04225-RHA1_ro04237, ROP_41490-ROP_41610; RER_02150-RER_02270, REQ_01990-REQ_02110, ROP_39370-ROP_39490, RHA1_ro04059-RHA1_ro04071; RER_36680-RER_36800, ROP_09180-ROP_09300, REQ_28910-REQ_29030, RHA1_ro01195-RHA1_ro01207; RHA1_ro02201-RHA1_ro02216, ROP_19150-ROP_19300; AARI_09490-AARI_09610, AAur_0263-AAur_0275, Achl_0509-Achl_0521, Arth_0282-Arth_0294, Asphe3_04140-Asphe3_04260; Asphe3_19150-Asphe3_19270, Arth_2192-Arth_2204, AAur_2192-AAur_2205, Achl_1933-Achl_1946; MAV_2363-MAV_2377, MAP_1861c-MAP_1877c; MLBr_02347-MLBr_02379, Mb_2950c-Mb_2977, MUL_1997-MUL_2027, MLBr_00129-MLBr_00153, MCAN_29471-MCAN_29731, MAF_29300-MAF_29580, Rv_2925c-Rv_2953, Mflv_3384-Mflv_3401, MMAR_1756-MMAR_1782; Mflv_0399-Mflv_0411, MAP_2224-MAP_2236, MLBr_01221-MLBr_01241, Mspyr1_03430-Mspyr1_03560, MAB_0933-MAB_0945, MAV_1757-MAV_1769, Mkms_0248-Mkms_0260, Mjls_0228-Mjls_0240, Mmcs_0238-Mmcs_0250; MAF_11930-MAF_12060, Rv_1174c-Rv_1187, Mb_1207c-Mb_1219, MCAN_11851-MCAN_11981; MAF_38340-MAF_38460, Mb_3849-Mb_3861, Rv_3819-Rv_3831, MCAN_38381-MCAN_38501; MMAR_0701-MMAR_0713, MCAN_03971-MCAN_04101, Rv_0399c-Rv_0411c, MAF_04010-MAF_04130, Mb_0406c-Mb_0419c; MAF_16670-MAF_16880, Rv_1654-Rv_1671, MAP_1363-MAP_1378, MCAN_16631-MCAN_16801, MUL_1646-MUL_1664, MMAR_2464-MMAR_2480, MAV_3100-MAV_3116, Mb_1682-Mb_1699c; Rv_2042c-Rv_2054, MAP_1790-MAP_1802c, MAF_20570-MAF_20690, MAV_2443-MAV_2457, Mb_2068c-Mb_2080, MMAR_3019-MMAR_3031, MUL_2259-MUL_2271, MCAN_20651-MCAN_20771; MCAN_15421-MCAN_15561, Rv_1521-Rv_1533, Mb_1548-Mb_1560, MAF_15470-MAF_15600; Mspyr1_27150-Mspyr1_27330, Mmcs_2826-Mmcs_2841, JDM601_2205-JDM601_2221, Mjls_2855-Mjls_2872, Mkms_2870-Mkms_2885; Mkms_3173-Mkms_3185, Mjls_3124-Mjls_3136, Mmcs_3113-Mmcs_3125; MAP_2597c-MAP_2610c, MAV_1314-MAV_1326; MUL_2632-MUL_2643, MMAR_1244-MMAR_1256; MAV_3049-MAV_3063, MAP_1414-MAP_1426c; MAB_2114c-MAB_2130, MAV_2003-MAV_2020, Mmcs_3459-Mmcs_3478, Mb_2394c-Mb_2410c, Mkms_3522-Mkms_3541, Mspyr1_21210-Mspyr1_21400, JDM601_1521-JDM601_1549, MMAR_3683-MMAR_3700, Mjls_3470-Mjls_3491, MUL_3625-MUL_3643, MAP_2165-MAP_2183c, MCAN_24051-MCAN_24231, MSMEG_4504-MSMEG_4521, Mflv_2684-Mflv_2703, Rv_2373c-Rv_2389c, MAF_23870-MAF_24030; MAV_3232-MAV_3250, MAP_1236c-MAP_1248; MMAR_2184-MMAR_2196, MUL_1767-MUL_1782, Mb_1399c-Mb_1416, MCAN_13821-MCAN_13971, Rv_1366-Rv_1381, MAF_13880-MAF_14030; Mb_0098c-Mb_0111c, MCAN_00981-MCAN_01111, MAF_00960-MAF_01080, Rv_0095c-Rv_0107c; Mjls_2361-Mjls_2374, Mmcs_2322-Mmcs_2334, Mkms_2369-Mkms_2381; MAV_1274-MAV_1286, Mflv_5134-Mflv_5146, MMAR_4307-MMAR_4319, Mjls_1080-Mjls_1092, MSMEG_0802-MSMEG_0815, Mkms_1069-Mkms_1081, Mspyr1_45540-Mspyr1_45660, Mmcs_1053-Mmcs_1065, JDM601_1754-JDM601_1766, MAP_2636-MAP_2648c; Mspyr1_33300-Mspyr1_33420, Mflv_3985-Mflv_3997; MUL_4973-MUL_4990, Mb_3824-Mb_3836c, MAV_0212-MAV_0225, MAP_0214-MAP_0226, Mspyr1_50120-Mspyr1_50240, Rv_3794-Rv_3806c, MAB_0174-MAB_0186c, MCAN_38131-MCAN_38251, JDM601_4110-JDM601_4123, MAF_38080-MAF_38210, Mjls_5385-Mjls_5397, Mkms_5092-Mkms_5104, Mflv_1163-Mflv_1175, MSMEG_6386-MSMEG_6398, Mmcs_5004-Mmcs_5016, MLBr_00095-MLBr_00108, MMAR_5358-MMAR_5370
